# A Gene Expression and Pre-mRNA Splicing Signature That Marks the Adenoma-Adenocarcinoma Progression in Colorectal Cancer

**DOI:** 10.1371/journal.pone.0087761

**Published:** 2014-02-06

**Authors:** Marine Pesson, Alain Volant, Arnaud Uguen, Kilian Trillet, Pierre De La Grange, Marc Aubry, Mélanie Daoulas, Michel Robaszkiewicz, Gérald Le Gac, Alain Morel, Brigitte Simon, Laurent Corcos

**Affiliations:** 1 Institut National de la Santé Et de la Recherche Médicale, Unité Mixte de Recherche 1078, Université de Bretagne Occidentale, Structure Fédérative de Recherche ScInBioS, Faculté de Médecine, Brest, France; 2 Centre Hospitalier Régional Universitaire de Brest, Brest, France; 3 GenoSplice technology, Hôpital Saint-Louis, Institut Universitaire d’Hématologie, Centre Hayem, Paris, France; 4 Plateforme de Génomique Santé, Laboratoire de Génomique Médicale, Centre National de la Recherche Scientifique, Unité Mixte de Recherche 6290, Institut de Génétique et Développement de Rennes, Unité Mixte de Services 3480 Biosit, Université de Rennes 1, Rennes, France; 5 Institut de Recherche contre le Cancer Nantes Atlantique, Institut National de la Santé Et de la Recherche Médicale, Unité Mixte de Recherche 892, Centre Paul Papin, Angers, France; Ohio State University Medical Center, United States of America

## Abstract

It is widely accepted that most colorectal cancers (CRCs) arise from colorectal adenomas (CRAs), but transcriptomic data characterizing the progression from colorectal normal mucosa to adenoma, and then to adenocarcinoma are scarce. These transition steps were investigated using microarrays, both at the level of gene expression and alternative pre-mRNA splicing. Many genes and exons were abnormally expressed in CRAs, even more than in CRCs, as compared to normal mucosae. Known biological pathways involved in CRC were altered in CRA, but several new enriched pathways were also recognized, such as the complement and coagulation cascades. We also identified four intersectional transcriptional signatures that could distinguish CRAs from normal mucosae or CRCs, including a signature of 40 genes differentially deregulated in both CRA and CRC samples. A majority of these genes had been described in different cancers, including *FBLN1* or *INHBA*, but only a few in CRC. Several of these changes were also observed at the protein level. In addition, 20% of these genes (*i.e. CFH*, *CRYAB*, *DPT*, *FBLN1*, *ITIH5*, *NR3C2*, *SLIT3* and *TIMP1*) showed altered pre-mRNA splicing in CRAs. As a global variation occurring since the CRA stage, and maintained in CRC, the expression and splicing changes of this 40-gene set may mark the risk of cancer occurrence from analysis of CRA biopsies.

## Introduction

Colorectal cancer (CRC) is one of the most prevalent cancers in developed countries, and is a major leading cause of cancer-related mortality worldwide. The most common type of CRC is adenocarcinoma (>95%), which is an invasive neoplasm of the glandular epithelium of the colon or rectum. It is accepted that adenocarcinomas may likely arise from colorectal adenomas (CRAs), as inferred from specific phenotypic features, such as size and histology.

Colorectal lesions are classified at endoscopy as non-polypoid (flat) and polypoid, which are separated into tubular, tubulovillous or villous, with different grades of dysplasia. CRAs are often referred to as adenomatous polyps that represent the lesions most frequently associated with neoplastic outcome, and it was shown that their removal was linked to a decrease in the incidence of CRC [1]. While tubular adenomas are the most common, villous adenomas are the least frequent, but they may transform into cancer with high frequency [2]. In addition, patients with previous multiple polyps had adenomas with advanced pathological features [3].

Several driver mutations have been identified during the progression from CRA to CRC [4], together with other molecular events, such as microRNA modulation [5] or pre-mRNA splicing alterations [6]. In addition, several gene expression profiles have been reported in CRC [7], [8]. Some studies also surveyed gene expression in CRA, and analyzed the lineage with CRC [9], [10], [11], [12], [13], [14]. Nevertheless, most analyses were performed from a limited number of CRA samples. Moreover, only a few studies have looked at the genome-wide alternative pre-mRNA splicing profiles of CRA samples [15] and their link with CRC, even though alternative splicing occurs for an estimated 90% of genes in the human genome [16].The aim of this study was to analyze, with microarrays, gene expression and alternative splicing in CRAs, in comparison with normal mucosae, but also with CRCs. We report here a comprehensive picture of the modifications that occurred in CRAs, some of which were specific for CRAs, while others were shared in CRCs. Importantly, we identified a 40-gene set (32 down- and 8 up-regulated genes), from an intersectional analysis of side-by-side comparisons, considering normal mucosae, CRAs and CRCs, that could mark the main regulatory events characterizing the stepwise progression in colorectal cancer.

## Materials and Methods

### Tissue Sample Processing

A written informed consent form was elaborated together with the Ethics Committee of Brest University Hospital (headed by Pr. J.M. Boles). Patients signed the form, which was returned to the Anatomy and Pathology department of Brest University Hospital. Hence, this study was approved by the Ethics committee of Brest University Hospital. Colon or rectum biopsy samples were obtained after surgical removal. The samples were then processed anonymously. The tissue fragments derived from biopsies were stored in RNAlater (Ambion, France): 55 CRAs, 25 CRCs and 27 colorectal normal mucosae (NOR; paired with CRAs or CRCs) were collected between 2006 and 2012, the majority as of 2009. From CRA or CRC biopsies, a surface fragment was collected from the tumor region, comprising on average 90% tumor cells, 5% lymphocytes and 5% stromal cells. These percentages were very homogenous between independent samples. Three subgroups (A1, A2 and A3) of CRAs could be distinguished according to histological data. Detailed patient information is presented in Table 1 and Table S1. DNA and total RNA were extracted with the AllPrep DNA/RNA Mini kit (Qiagen, Courtabœuf, France) from homogenized tissue samples (20 mg), according to the manufacturer’s instructions. RNA purity and integrity were determined by measuring the optical density ratio (A260/A280) and the RNA integrity number (RIN) was obtained using the RNA 6000 Nano LabChip (Agilent, Massy, France) and the 2100 Bioanalyzer (Agilent). Only RNA samples with a 28S/18S ratio >1.0 and RIN ≥7.0 were used for microarray analyses.

**Table 1:** Characteristics of colorectal biopsy samples used in the present study.

		CRA					
	Group	Subgroup A1	Subgroup A2	Subgroup A3	Out-of-Class	CRC	NOR
Agilent Whole Human Genome Microarray	Number of Samples	9	13	15	0	9	9
	Gender (male/female)	(6/3)	(10/3)	(11/4)		(5/4)	(5/4)
	Mean Age (range, years)	73 (58–92)	63 (52–77)	64 (46–88)		71 (48–92)	71 (48–92)
Affymetrix HumanExon 1.0 ST Array	Number of Samples	7	7	9	1	0	0
	Gender (male/female)	(2/5)	(6/1)	(6/3)	(0/1)		
	Mean Age (range, years)	71 (58–84)	68 (52–92)	62 (46–82)	50		

Abbreviations: NOR: colorectal normal mucosa; CRA: colorectal adenoma; CRC: colorectal cancer.

### Whole-Genome Microarray

An analysis of 55 RNA samples derived from colorectal tissue, consisting of three sample groups (NOR, CRA and CRC) with varying numbers of biological replicates, was performed on 44k Whole Human Genome microarrays (Agilent) that contain 41,093 probes, providing full coverage of human transcripts. Double-stranded cDNA was synthesized from 500 ng of total RNA using the Quick Amp Labeling kit, One-color, as instructed by the manufacturer (Agilent). Labeling with cyanine3-CTP, fragmentation of cRNA, hybridization, and washing were performed according to the manufacturer’s instructions (Agilent). The microarrays were scanned and the data were extracted with the Agilent Feature Extraction Software.

### Gene Expression Analysis

Raw gene expression data were imported into the GeneSpring GX 11.0.2 software program (Agilent). Side-by-side comparisons were performed for gene expression alterations: CRC *vs.* paired NOR, CRA *vs.* NOR, and CRC *vs.* CRA. Genes with missing values in more than 25% of the samples were excluded from the analysis. These data have been deposited in NCBI’s Gene Expression Omnibus and are accessible through GEO Series accession numbers GSE50114, GSE50115 and GSE50117. A 2-fold cut-off difference was applied to select the up- and down-regulated genes (P-value ≤0.01 by *t*-test with Benjamini-Hochberg false discovery rate, FDR). Hierarchical clustering of the expression data was performed using Euclidean distance with average linkage.

### Gene Set Enrichment Analysis

The publicly available software, Database for Annotation, Visualization and Integrated Discovery [17], was used to analyze the gene set enrichment in colorectal lesions. A 2-fold cut-off difference was applied to select the list of deregulated genes (P-value ≤0.01 by *t*-test with FDR). Only the pathways from the Kyoto Encyclopedia of Genes and Genomes (KEGG) will be described [18].

### Alternative Splicing Analysis

A pooled RNA, assayed in duplicate, from 3 colorectal normal mucosae and 24 CRA RNA samples were analyzed on Human Exon 1.0 ST arrays (Affymetrix, Paris, France), which enabled analysis of both gene expression and alternative splicing. Microarray hybridization was performed at the Curie Institute facility (Paris, France). The raw data were analyzed by GenoSplice technology. These data are accessible through GEO Series accession number GSE50592. A 1.5-fold cut-off difference was applied to select the up- and down-regulated genes and exons (P-value ≤0.05).

### Real-Time Polymerase Chain Reaction Validation

As a validation step of microarray results, quantitative RT-PCR was performed on three groups (NOR, CRA and CRC) of at least 8 samples, including some of the samples hybridized on microarrays, or on an independent set of 14 CRAs and 8 paired tumor-normal CRC samples. Total RNA (200 ng) was used for first-strand cDNA synthesis with the High-Capacity cDNA Reverse Transcription kit (Applied Biosystems). Quantitative RT-PCR was performed using the Power SYBR Green PCR Master Mix (Applied Biosystems) according to the manufacturer’s instructions with an ABI 7000 or 7300 real-time PCR system (Applied Biosystems). All determinations were performed in duplicate and normalized against *beta*-2-microglobulin as an internal control gene. The results were expressed as the relative gene expression using the ΔΔCt method [19]. All of the tested genes were selected based on the microarray analyses, in order to validate the biological pathway enrichment and a gene signature in CRAs and CRCs. The primer sequences and reaction conditions will be provided upon request. In addition, a PCR array setup (Qiagen) was used to analyze, in NOR and CRC samples, the expression of genes with primers present among the PCR array multiwell plates (Apoptosis, Cancer Pathway Finder, Drug Metabolism, Lipoprotein Signaling and Cholesterol Metabolism, *Wnt* Signaling Pathway).

## Results

### Comparison of Colorectal Adenoma Morphological Subgroups

Several mutational landmarks have been described in the progression to colorectal cancer, such as *KRAS*, *BRAF* and *PI3K* mutations [4], [20], and were analyzed in our samples (Supporting Information). In addition, the microsatellite instability status (Supporting Information) was determined in 12 CRA samples, but all were negative. The Vienna classification allowed to group adenomas into two classes: a minor group of lower grade (3) with 11 (22%) samples and a major group of 40 (78%) samples of higher grade (>3) (Table S1). This classification did not match with the tubular/villous/tubulovillous lesion types, since CRAs with both low grade and high grade dysplasia were evenly distributed into the tubullovillous and tubular groups (only one CRA was from the villous type). This separation in tubular, villous or tubulovillous was therefore not adopted. We decided to rely on a precise morphology analysis and applied an anatomical grouping, which led to the distinction of three morphological subgroups: adenomas with areas of micro-invasive adenocarcinomas (A1; 10 samples), degenerated adenomas, *i.e*. adenomas with areas of *in situ* (intra-mucosa) adenocarcinomas (A2; 17 samples), and adenomas with areas of dysplasia (A3; 24 samples). In order to determine if CRAs could also be distinguished by molecular means, a one-way ANOVA was performed to compare CRA subgroups to CRC and NOR groups, with “tissue type” as an ANOVA factor (data not shown). The analysis revealed that CRA subgroups were very close with one another. There was no difference between subgroups A2 and A3, and the maximum number of deregulated probes was found for the subgroup A1 *vs*. subgroup A2 comparison (49 probes, corresponding to 0.12% of total number of probes, P-value ≤0.01). Moreover, while the comparisons between CRA subgroups and normal mucosae showed the largest numbers of distinctive probes (up to 4,382 probes in subgroup A2 *vs*. NOR), the comparisons between CRA subgroups and CRCs showed the smallest (up to 1,424 probes in CRC *vs*. subgroup A2). CRAs as a whole were thus more distinct from normal mucosae than from CRCs. The three CRA subgroups were also compared to each other, and no difference was observed in side-by-side comparisons (P-value of ≤0.01 by *t*-test with FDR). Consequently, CRAs were considered collectively as a single group for further side-by-side comparisons by Student’s *t*-test.

### Gene Expression Profiling in Colorectal Lesions in Comparison with Normal Mucosae

**Figure 1:**
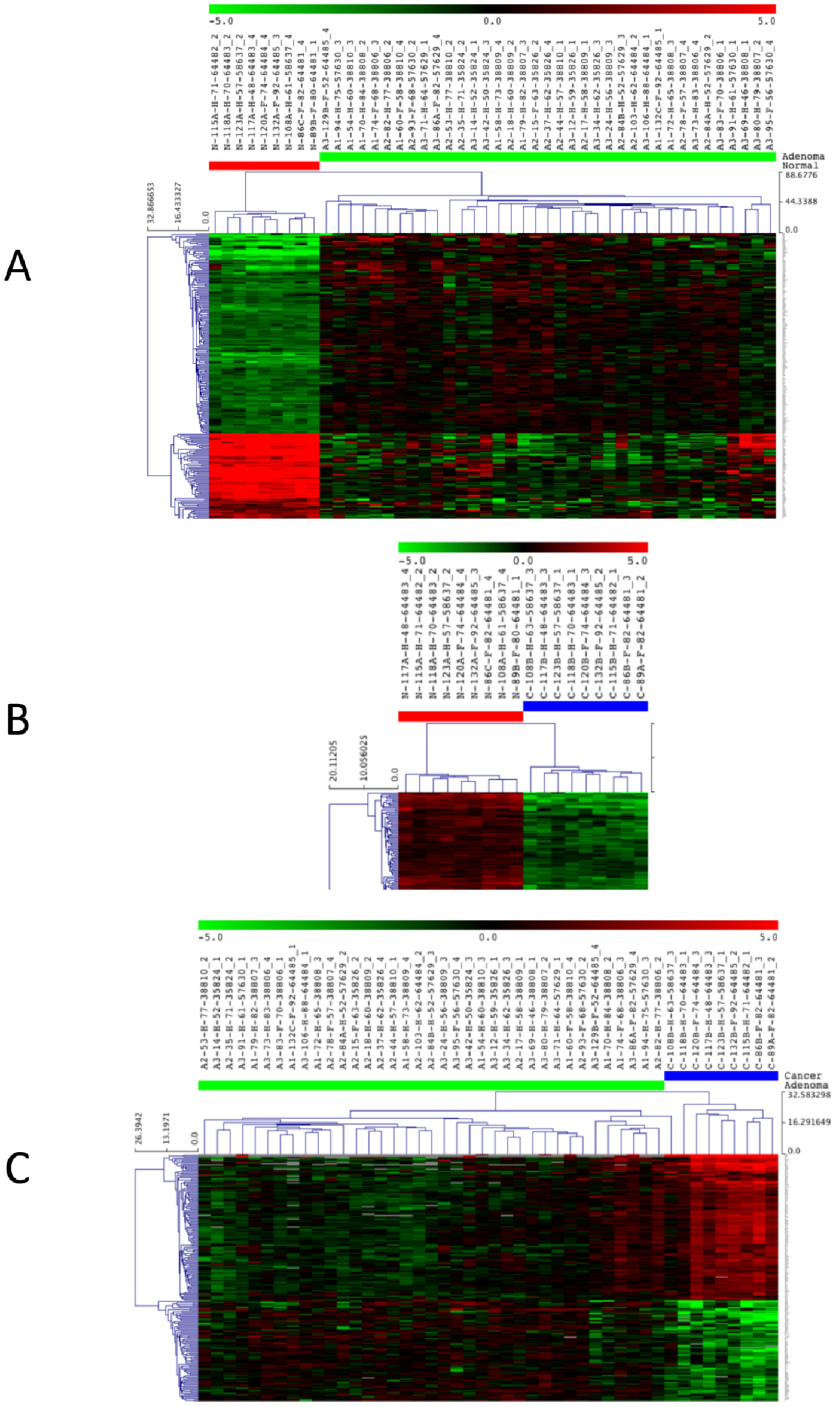
Hierarchical clustering considering the gene expression in colorectal lesions. Heat map of the expression data was constructed using Euclidean distance with average linkage. The heat map of the deregulated probes with a fold-change ≥3.0 and a P-value ≤0.001 is shown for CRA *vs*. NOR (**A**; complete heat map in Figure S1), for CRC *vs*. NOR (**B**), and CRC *vs*. CRA (**C**).

In order to identify genes that could participate in the progression from normal mucosa to CRA, we performed a CRA *vs*. NOR comparison, and found that 2,393 probes were deregulated in CRAs (≥2.0 fold-change (FC), P-value of ≤0.01 by *t*-test with FDR), corresponding to 32% up- and 68% down-regulations. The CRC *vs*. NOR comparison showed that 1,805 probes were deregulated in CRCs (≥2.0 FC, P-value ≤0.01 by paired *t*-test with FDR), corresponding to 46% up- and 54% down-regulations. The heat maps of the deregulated probes with a fold-change ≥3.0 and a P-value ≤0.001 are shown in Figures 1A (CRA *vs*. NOR) and 1B (CRC *vs*. NOR), and Figure S1 (CRA *vs*. NOR, full image). Complete lists of the differentially expressed probes in CRA *vs*. NOR and CRC *vs*. NOR are presented in Tables S2 and S3, respectively. A set of deregulation events in CRA *vs*. NOR was analyzed by quantitative RT-PCR, and the validation rate of Agilent microarray results was 78% (50 out of 64 transcripts; Table S4). In addition, Qiagen PCR array experiments were performed on an independent set of 96 CRC and 20 NOR samples (from Brest tumor bank). Among the deregulated probes in CRC *vs*. NOR on microarrays, 41 primer pairs corresponding to the same genes that were present in the PCR arrays. Twenty-eight were also deregulated in PCR arrays (≥2.0 FC, P-value ≤0.01), corresponding to 68% cross validation (Table S5).

**Figure 2:**
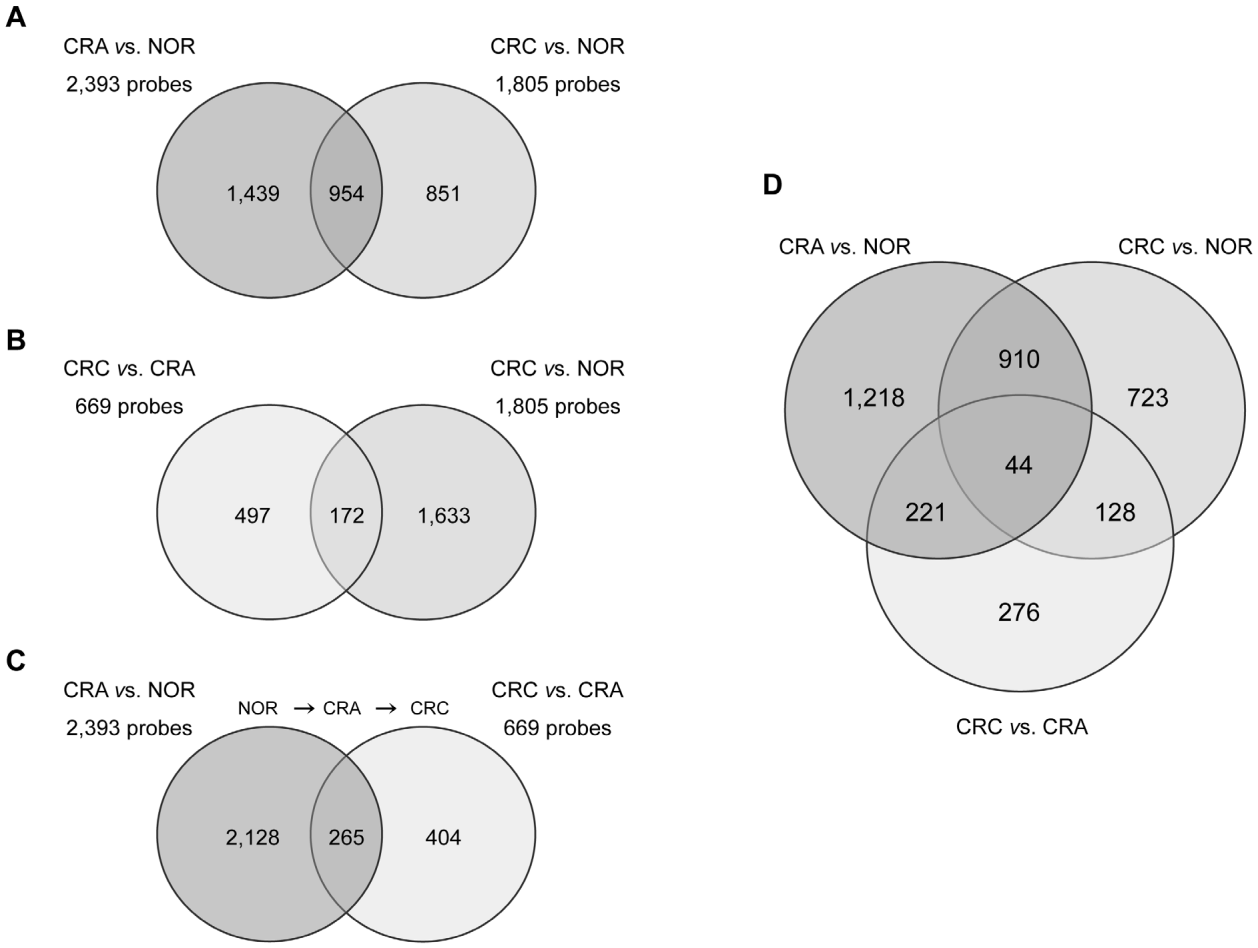
Venn diagrams of probe level alterations in colorectal lesions. An intersectional analysis of probe level alterations was performed. Cut-off values were P-value ≤0.01 and fold-change ≥2. The CRA *vs*. NOR comparison showed the largest number of probe level changes (2,393 deregulated probes), while the CRC *vs*. CRA comparison showed the lowest (669 deregulated probes). The probes that showed alterations in two or in the three comparisons were of interest. (**A**) Signature of 954 probes deregulated in both CRA and CRC lesions as compared to NOR. (**B**) Signature of 172 probes deregulated in CRC in comparison to both CRA and NOR. (**C**) Signature of 265 probes deregulated in CRC as compared to CRA, which levels were already abnormal in CRA as compared to NOR. (**D**) Signature of 44 probes showing alterations in the three comparisons (CRA *vs*. NOR, CRC *vs*. CRA and CRC *vs*. NOR). Abbreviations: NOR: colorectal normal mucosa; CRA: colorectal adenoma; CRC: colorectal cancer.

The CRA *vs*. NOR comparison showed more differences than the CRC *vs*. NOR comparison, and there were more down-regulations (68% in CRA *vs*. 54% in CRC) than up-regulations (32% in CRA *vs*. 46% in CRC). An intersectional analysis of probe level alterations was performed (Figure 2A), showing a signature of 954 probes deregulated in both CRA and CRC samples as compared to normal mucosae (Table S6 and Figure S2), corresponding to 40% and 53% deregulated probes in CRA and CRC, respectively. All commonly deregulated probes followed the same type of variation in both comparisons, *i.e.* were up- or down-regulated similarly.

### Pathway Enrichment in Colorectal Lesions in Comparison with Normal Mucosae

The KEGG pathway analysis showed 25 gene sets distinguishing CRA from NOR, and 20 distinguishing CRC from NOR (P-value ≤0.05; Table 2), considering deregulated probes with a 2-fold cut-off (P-value ≤0.01 by *t*-test with FDR). The complement and coagulation cascades, cytokine-cytokine receptor interaction, and chemokine signaling pathways were among the top of enriched pathways in CRA *vs*. NOR, while cell cycle and DNA replication were pathways most affected in CRC *vs*. NOR, according to the P-value. Seven pathways were enriched in both CRA *vs*. NOR and CRC *vs*. NOR comparisons, among which the p53 signaling pathway was part of already described enriched pathways in CRA [14]. Nitrogen metabolism was also a commonly enriched pathway between both analyses, and included the carbonic anhydrases (*CA1* and *CA4*) that were part of the most down-regulated probes in CRA and CRC.

**Table 2:** KEGG gene sets enriched in colorectal lesions.

KEGG Enriched Pathway	P-value	Benjamini-Hochberg	FoldEnrichment	Number ofGenes in thePathway	Number ofDeregulatedGenes	Percentage ofDeregulatedGenes
**Colorectal Adenoma ** ***vs*** **. Normal**						
Complement and coagulation cascades	5.6E-08	1.0E-05	3.29	69	26	38%
Cytokine-cytokine receptor interaction	3.4E-05	3.1E-03	1.76	262	53	20%
Chemokine signaling pathway	5.0E-04	3.0E-02	1.77	187	38	20%
Viral myocarditis	2.1E-03	9.3E-02	2.21	71	18	25%
Drug metabolism	2.4E-03	8.4E-02	2.64	43	13	30%
Intestinal immune network for IgA production	2.7E-03	7.7E-02	2.49	49	14	29%
Hematopoietic cell lineage	3.3E-03	8.1E-02	2.03	86	20	23%
Focal adhesion	3.7E-03	8.1E-02	1.61	201	37	18%
Aldosterone-regulated sodium reabsorption	5.1E-03	9.7E-02	2.55	41	12	29%
Axon guidance	5.3E-03	9.1E-02	1.76	129	26	20%
Androgen and oestrogen metabolism	7.0E-03	1.1E-01	2.59	37	11	30%
Cell adhesion molecules (CAMs)	7.1E-03	1.0E-01	1.72	132	26	20%
Pentose and glucuronate interconversions	1.2E-02	1.6E-01	3.39	18	7	39%
ECM-receptor interaction	1.3E-02	1.5E-01	1.87	84	18	21%
Asthma	1.4E-02	1.5E-01	2.71	29	9	31%
Pathways in cancer	1.9E-02	1.9E-01	1.36	328	51	16%
Basal cell carcinoma	1.9E-02	1.9E-01	2.06	55	13	24%
Leukocyte transendothelial migration	2.5E-02	2.3E-01	1.63	118	22	19%
Colorectal cancer	2.7E-02	2.3E-01	1.77	84	17	20%
Folate biosynthesis	2.9E-02	2.3E-01	3.96	11	5	45%
Ascorbate and aldarate metabolism	3.7E-02	2.8E-01	3.08	17	6	35%
Sulfur metabolism	4.0E-02	2.8E-01	3.63	12	5	42%
Prion diseases	4.0E-02	2.8E-01	2.24	35	9	26%
Nitrogen metabolism	4.0E-02	2.7E-01	2.65	23	7	30%
p53 signaling pathway	4.2E-02	2.7E-01	1.80	68	14	21%
**Colorectal Cancer ** ***vs*** **. Normal**						
Cell cycle	2.2E-07	3.9E-05	2.96	125	29	23%
DNA replication	9.9E-06	8.9E-04	4.60	36	13	36%
Pentose and glucuronate interconversions	2.5E-04	1.5E-02	5.66	18	8	44%
Purine metabolism	7.0E-04	3.1E-02	2.08	153	25	16%
Oocyte meiosis	2.0E-03	6.9E-02	2.20	110	19	17%
p53 signaling pathway	2.0E-03	5.8E-02	2.62	68	14	21%
Drug metabolism	2.7E-03	6.6E-02	2.67	62	13	21%
Starch and sucrose metabolism	4.4E-03	9.4E-02	3.03	42	10	24%
Mismatch repair	7.1E-03	1.3E-01	3.88	23	7	30%
Nitrogen metabolism	7.1E-03	1.3E-01	3.88	23	7	30%
Ascorbate and aldarate metabolism	8.1E-03	1.4E-01	4.50	17	6	35%
Sulfur metabolism	1.1E-02	1.7E-01	5.31	12	5	42%
Pyramiding metabolism	1.5E-02	2.0E-01	2.01	95	15	16%
Progesterone-mediated acolyte maturation	1.5E-02	1.9E-01	2.07	86	14	16%
Drug metabolism	1.7E-02	1.9E-01	2.67	43	9	21%
Metabolism of xenobiotics by cytochrome P	1.7E-02	1.8E-01	2.34	60	11	18%
Androgen and estrogen metabolism	2.3E-02	2.3E-01	2.76	37	8	22%
Retinol metabolism	2.3E-02	2.2E-01	2.36	54	10	19%
Steroid hormone biosynthesis	2.5E-02	2.2E-01	2.49	46	9	20%
Glycine, serine and threonine metabolism	3.0E-02	2.5E-01	2.88	31	7	23%
**Colorectal Cancer ** ***vs*** **. Colorectal Adenoma**						
ECM-receptor interaction	5.6E-05	7.8E-03	4.12	84	13	15%
TGF-*beta* signaling pathway	7.9E-05	5.6E-03	3.98	87	13	15%
Focal adhesion	1.5E-04	7.2E-03	2.65	201	20	10%
Complement and coagulation cascades	3.9E-03	1.3E-01	3.47	69	9	13%
Arginine and proline metabolism	4.7E-02	7.4E-01	3.01	53	6	11%

The KEGG pathway analysis showed 25 gene sets distinguishing CRA from NOR, 20 distinguishing CRC from NOR, and five distinguishing CRC from CRA (P-value ≤0.05), considering deregulated genes with a 2-fold cut-off difference (P-value ≤0.01 by *t*-test with FDR).

If a 1.1-fold cut-off difference instead of 2.0 was applied to select deregulated probes (P-value ≤0.01), *i.e*. if all deregulated probes were considered (5 733 probes), 18 gene sets instead of 25 were altered in CRA *vs*. NOR according to KEGG (P-value ≤0.05; Table S7). Only the complement and coagulation cascades pathway was common between both the 18 and 25 gene lists. Therefore, 17 new pathways were enriched in CRA, such as DNA replication, cell cycle, spliceosome or mismatch repair.

### Gene Expression Profiling in Colorectal Adenocarcinomas in Comparison with Colorectal Adenomas

An analysis of differentially detected probes between CRC and CRA identified 669 deregulated probes (≥2.0 FC, P-value of ≤0.01 by *t*-test with FDR), corresponding to 55% up- and 45% down-regulations. The heat map of the deregulated probes with a fold-change ≥3.0 and a P-value ≤0.001 is shown in Figure 1C. The complete list of the differential probe signals in CRC *vs*. CRA is presented in Table S8. The CRC *vs*. CRA comparison showed fewer probe level differences with much lower fold-changes than the CRC *vs*. NOR and CRA *vs*. NOR comparisons. The intersectional analysis of probe signals showed a signature of 172 probes deregulated in CRC as compared to both CRA and NOR samples (Figure 2B, Table S9 and Figure S3), corresponding to 26% deregulated probes in CRC *vs*. CRA, and less than 10% deregulated probes in CRC *vs*. NOR. As these modifications were not present in CRA, they could be markers of CRC aggressiveness.

### Pathway Enrichment in Colorectal Adenocarcinomas in Comparison with Colorectal Adenomas

The KEGG pathway analysis revealed five gene sets distinguishing CRC from CRA (P-value ≤0.05; Table 2), considering deregulated probes with a 2-fold cut-off (P-value ≤0.01 by *t*-test with FDR). Two enriched pathways were specific for the CRC *vs*. CRA comparison: arginine and proline metabolism, and TGF-*beta* signaling pathway that has been already described as an altered pathway between CRA and CRC [9]. Moreover, the CRA *vs*. NOR and CRC *vs*. CRA comparisons had three commonly enriched pathways, among which focal adhesion and ECM-receptor interaction were part of already reported pathways enriched in colon carcinogenesis [21]. These pathways could play an important role in the progression of CRC, because they were enriched from NOR to CRA, and then from CRA to CRC.

### Intermediate Signature of Progression from Colorectal Adenoma to Colorectal Adenocarcinoma

The evidence for the progression from NOR to CRA, and then to CRC, was investigated with an intersectional analysis of probe level alterations. A signature of 265 probes, corresponding to 215 genes, was identified (Figure 2C, Table S10 and Figure S4), which was coincidental in lists of the 2,393 and 669 deregulated probes, corresponding to the CRA *vs*. NOR and CRC *vs*. CRA comparisons, respectively. It included deregulated probes in CRC *vs*. CRA, which were already distinct in the CRA *vs*. NOR analysis. The distributions of up- and down-regulated events in CRC *vs*. CRA were 69% and 31%, respectively. An enrichment analysis of the signature of 265 probes was performed using KEGG pathways, and revealed that 41 genes were part of eight enriched gene sets, including focal adhesion, ECM-receptor interaction or TGF-*beta* signaling pathway (Table S11). Moreover, an intermediate gene expression signature of 44 probes (corresponding to 40 genes) was identified (Figure 2D and Table 3), which was coincidental in the three lists of deregulated probes, and then was part of all signatures that we previously described (signatures of 954, 172 and 265 probes). It corresponded to 8 up- and 32 down-regulated genes in both CRA and CRC samples, as compared to normal mucosae. Eight probes demonstrated progressively increased signals from NOR to CRA, and then to CRC; 23 probes revealed gradually decreased signals. In addition, 13 probes were less suppressed in CRC than in CRA, as compared to NOR.

**Table 3:** List of the up- and down-regulated genes of the gene expression signature of 44 probes.

		Colorectal Adenoma *vs*. Normal			Colorectal Cancer *vs*. Normal			Colorectal Cancer *vs*. Colorectal Adenoma		
Gene Symbol	Probe Name	P-value	Fold-Change	Regulation	P-value	Fold-Change	Regulation	P-value	Fold-Change	Regulation
*SCARA5*	A_23_P94103	1.04E-14	11.54	down	1.11E-03	36.56	down	1.27E-03	3.17	down
*IGHA2*	A_23_P61042	7.04E-03	3.77	down	3.43E-03	21.85	down	7.54E-03	5.80	down
*BEST2*	A_23_P16225	4.59E-03	4.25	down	1.11E-03	20.25	down	9.80E-03	4.76	down
*C6orf105*	A_23_P156826	3.72E-03	2.78	down	1.86E-03	18.54	down	9.22E-05	6.66	down
*FAM55D*	A_23_P320216	1.00E-02	2.25	down	3.65E-03	17.45	down	2.00E-05	7.76	down
*DNASE1L3*	A_23_P257993	2.08E-04	2.39	down	1.94E-03	12.77	down	8.39E-06	5.34	down
*UGT1A6*	A_23_P60599	2.65E-03	2.33	down	3.46E-03	10.89	down	1.43E-04	4.67	down
*LRRC19*	A_23_P364625	1.38E-07	3.55	down	6.01E-03	9.42	down	3.52E-03	2.65	down
*IGJ*	A_23_P167168	5.24E-03	2.50	down	3.42E-03	8.87	down	2.42E-03	3.55	down
*ISX*	A_32_P217140	3.53E-04	2.65	down	4.80E-03	8.32	down	2.19E-03	3.14	down
*NR3C2*	A_23_P392470	3.98E-06	2.58	down	6.39E-03	7.53	down	3.79E-04	2.92	down
*SMPDL3A*	A_23_P72117	1.20E-06	3.08	down	1.90E-03	7.07	down	2.30E-03	2.29	down
*HSD11B2*	A_23_P14986	2.52E-06	2.64	down	1.16E-03	7.07	down	6.38E-05	2.68	down
*RDH5*	A_24_P218814	2.54E-03	2.22	down	8.51E-04	6.34	down	7.18E-04	2.85	down
*SEPP1*	A_23_P121926	3.11E-06	2.95	down	2.45E-03	6.27	down	6.02E-03	2.13	down
*ITM2C*	A_24_P379820	3.47E-03	2.04	down	2.84E-03	5.85	down	1.14E-03	2.87	down
*ITM2C*	A_24_P402690	5.68E-04	2.08	down	5.51E-03	5.62	down	3.14E-04	2.70	down
*PBLD*	A_23_P149998	3.95E-05	2.35	down	9.07E-03	5.58	down	5.90E-03	2.38	down
*PBLD*	A_24_P112395	1.11E-04	2.21	down	7.65E-03	5.31	down	3.69E-03	2.40	down
*LOC400573*	A_32_P515920	7.71E-06	2.50	down	1.24E-03	5.17	down	3.48E-03	2.06	down
*ASAP3*	A_23_P114689	5.04E-05	2.37	down	7.64E-04	5.03	down	2.44E-03	2.13	down
*C1orf115*	A_23_P160433	1.89E-04	2.23	down	4.35E-03	4.83	down	2.85E-03	2.16	down
*C1orf115*	A_24_P131173	8.42E-04	2.08	down	6.01E-03	4.46	down	3.10E-03	2.15	down
*DPT*	A_23_P200741	6.20E-18	27.31	down	6.92E-03	5.36	down	3.56E-07	5.09	up
*SLIT3*	A_23_P58588	3.42E-14	12.05	down	8.11E-04	5.19	down	2.55E-03	2.32	up
*CFHR3*	A_23_P103256	8.67E-16	9.06	down	5.95E-03	4.02	down	3.63E-04	2.25	up
*CFH*	A_23_P200160	3.65E-11	8.65	down	3.43E-03	3.90	down	6.86E-03	2.22	up
*FBLN1*	A_23_P433016	8.05E-13	12.99	down	5.53E-03	3.40	down	9.13E-05	3.82	up
*CCDC80*	A_23_P58082	7.18E-14	15.11	down	5.62E-03	3.35	down	5.61E-06	4.52	up
*FBLN1*	A_23_P211631	1.36E-11	13.36	down	7.13E-03	3.20	down	1.02E-04	4.18	up
*SPARCL1*	A_23_P113351	2.28E-11	6.11	down	6.48E-03	3.01	down	4.95E-03	2.03	up
*CRYAB*	A_24_P206776	2.31E-15	5.69	down	2.93E-03	2.72	down	1.96E-04	2.09	up
*VSIG4*	A_23_P217269	3.01E-10	8.43	down	2.61E-03	2.65	down	3.99E-04	3.18	up
*PLN*	A_23_P30614	2.07E-07	8.08	down	3.37E-03	2.61	down	6.24E-03	3.10	up
*ITIH5*	A_23_P411993	3.16E-12	5.07	down	6.28E-03	2.40	down	2.69E-04	2.11	up
*DACT3*	A_23_P360964	3.82E-12	6.29	down	9.46E-03	2.30	down	1.11E-04	2.73	up
*INHBA*	A_23_P122924	1.66E-06	5.72	up	6.54E-04	41.06	up	1.22E-05	7.18	up
*TRIB3*	A_23_P210690	1.73E-04	3.17	up	1.45E-03	9.88	up	3.10E-03	3.11	up
*JUB*	A_23_P54055	2.03E-08	4.24	up	1.11E-03	8.77	up	2.75E-03	2.07	up
*PSAT1*	A_23_P259692	4.48E-03	2.47	up	5.72E-03	6.95	up	1.47E-03	2.81	up
*MYBL2*	A_23_P143190	3.96E-05	2.40	up	8.37E-03	5.19	up	2.58E-03	2.16	up
*SKA3*	A_23_P340909	1.05E-04	2.15	up	1.86E-03	4.65	up	3.87E-04	2.16	up
*UBE2S*	A_32_P184933	2.74E-05	2.01	up	1.70E-03	4.56	up	4.22E-05	2.27	up
*TIMP1*	A_23_P62115	3.34E-04	2.11	up	2.25E-03	4.39	up	4.62E-03	2.08	up

Signature of 44 probes corresponding to genes showing alterations in the three comparisons (CRA *vs*. NOR, CRC *vs*. CRA and CRC *vs*. NOR; ≥2.0 FC, P-value ≤0.01 by *t*-test with FDR).

### Classification of Colorectal Adenomas in Comparison with Normal Mucosae and Colorectal Adenocarcinomas

**Figure 3:**
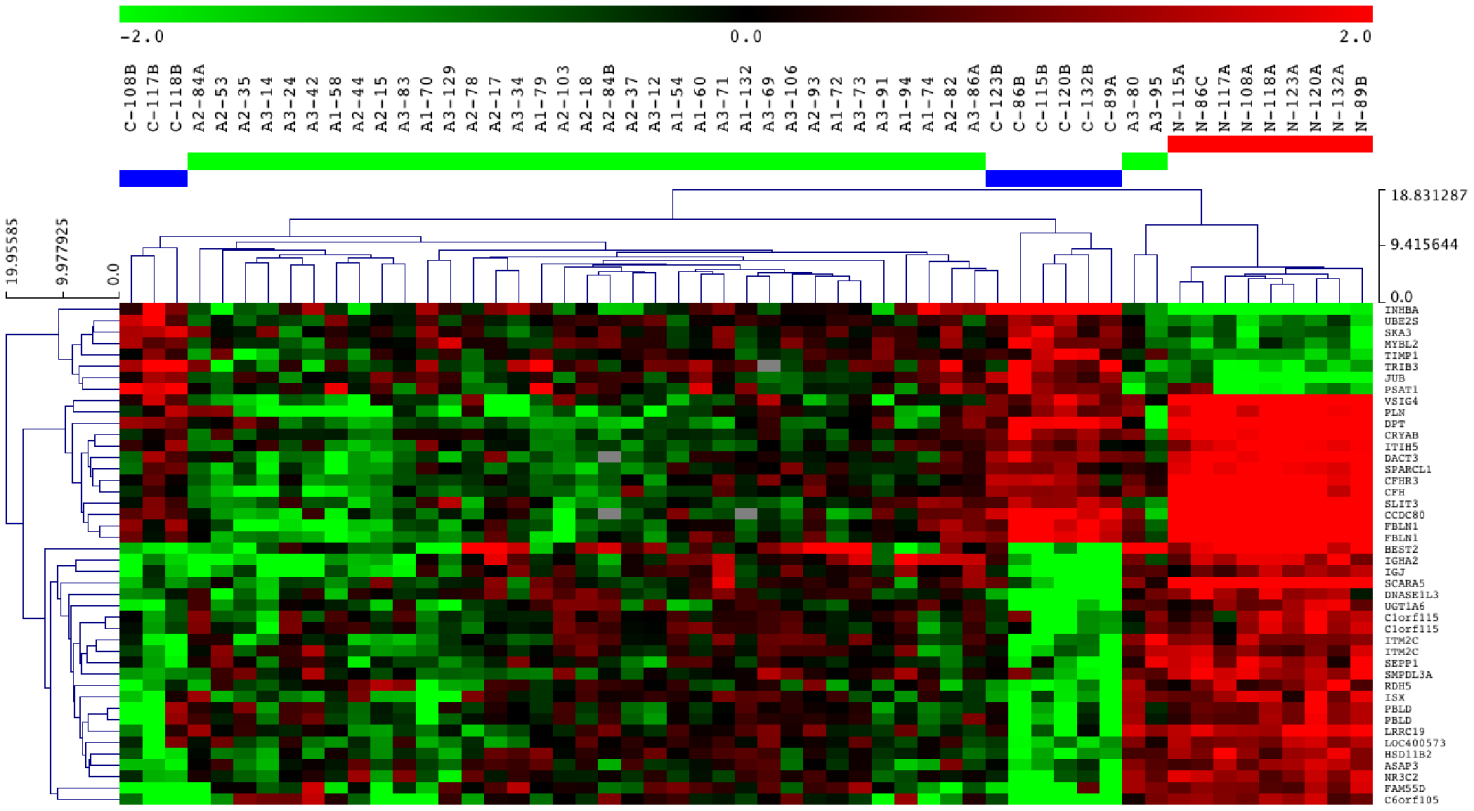
Hierarchical clustering (Euclidean, average linkage) considering the expression signature of 44 probes. Branches represent individual colorectal samples. Different colors were used to identify the sample groups: red, group of normal mucosae (N: normal); green, group of adenomas (A: adenoma); blue, group of adenocarcinomas (C: cancer). The first sample annotation corresponds to the sample group. The subgroups of adenomas are specified: A1, adenomas with areas of micro-invasive adenocarcinomas; A2, adenomas with areas of intra-mucosa adenocarcinomas; A3, adenomas with areas of dysplasia. The second sample annotation corresponds to the sample number.

A classification of the colorectal tissues was performed using hierarchical clustering of probe signal alterations corresponding to the four signatures previously described. Only two groups were distinguished considering the signature of 954 probes (Figure S2): one was composed of normal mucosae and the other contained a mix of colorectal lesions. By contrast, the clustering considering the signature of 172 probes allowed to distinguish the three types of colorectal tissues (Figure S3): one group was only composed of CRCs, and the other was divided into a CRA subgroup and a NOR subgroup. Similarly, the clustering with the signature of 265 probes enabled to distinguish the three sample types (Figure S4), but one group was only composed of CRAs, and the other grouped together the NOR and CRC samples that were distributed into two distinct subgroups. Finally, the signature of 44 probes showed that the majority of CRAs clustered with CRCs, a few CRAs (showing the least affected histology) being grouped with NOR samples (Figure 3). For the majority of samples, no strict concordance between histological (morphological subgroups or localization) and molecular data was recognized concerning the distribution of CRAs into subgroups. Similarly, the specifics of CRC clustering were not explained by tumor localization (Table S1). Molecular data could thus give supplementary information to classify the colorectal lesions.

### Exon-Level Analysis in Colorectal Adenomas

A CRA *vs*. NOR comparison was performed on Human Exon 1.0 ST arrays (Affymetrix), and showed that 1,484 genes were deregulated in CRA (590 up- and 894 down-regulated genes; ≥1.5 FC, P-value ≤0.05; Table S12). A corresponding heat map is shown in Figure S5. A set of deregulated transcripts in CRA *vs*. NOR was analyzed by quantitative RT-PCR, and the validation rate of Affymetrix microarray results was 83% (24 out of 29 transcripts, also validated for the Agilent analysis). In addition, the CRA *vs*. NOR comparison showed extensive changes in alternative splicing profiles: 1,852 exons were deregulated in CRA (862 up- and 990 down-regulated exons; ≥1.5 FC, P-value ≤0.05; Table S13). A publicly available microarray expression data set from 10 paired tumor-normal CRC samples [6] was downloaded from the Affymetrix web site in order to compare alternative splicing profiling in CRA and CRC. The CRA *vs*. NOR and CRC *vs*. NOR comparisons had 100 deregulated exons in common. While 47 up- and 47 down-regulated splicing events followed the same type of variation in the two comparisons, few regulations were opposite in CRA and CRC, corresponding to 6% of common deregulated exons (data not shown). We found that 296 deregulated (102 up- and 194 down-regulated) probes in CRA *vs*. NOR from the Agilent analysis showed deregulated exons in the Affymetrix analysis (data not shown). A lot of genes that were part of altered pathways had deregulated exons. Among the 40 genes of the Agilent transcriptional signature of 44 probes, 8 (*CFH*, *CRYAB*, *DPT*, *FBLN1*, *ITIH5*, *NR3C2*, *SLIT3* and *TIMP1*), *i.e*. 20%, had deregulated exons (Table S14).

## Discussion

The aim of this study was to investigate, at the whole-transcriptome level, the extent of variations that occur in human colorectal adenomas in comparison to adenocarcinomas, taking the normal epithelium as a reference. Many changes were apparent in CRA *vs*. NOR, even more so than in CRC *vs*. NOR. Hence, CRA, as a type of intermediary lesion, already exhibited strong signs of alterations. From the molecular changes evidenced in CRA, it is clear that CRAs are not merely accumulating alterations that will all be found in CRCs. Possibly, the evolution to CRCs follows a more strictly clonal expansion, which may lead to select for gene changes important for clonal growth while eliminating less relevant modifications. According to this hypothesis, CRAs may have different outcomes, some evolving towards cancer, while others could be prone to disappearance. We identified four signatures distinguishing the types of colorectal tissues, and showed that a 40-gene set could be of specific interest, marking the molecular changes that distinguish the normal mucosa from CRA and CRC. Importantly, several alternative pre-mRNA splicing events were also characteristic of the CRA to CRC progression.

Several genes implicated in CRC were deregulated in CRA *vs*. NOR. The highest increases in probe levels included *KIA1199* that had already been found deregulated in CRA [22], or the matrix metalloproteinase *MMP7* which over-expression is known to influence early colorectal carcinogenesis [23]. Fifteen gene sets, such as those involved in cytokine-cytokine receptor interaction, chemokine signaling pathway, or cell adhesion molecules, were specific for CRA *vs*. NOR. Importantly, several new enriched biological pathways were identified, among which the complement and coagulation cascades pathway was the most significantly affected in the Agilent analysis, and was also identified as altered in the Affymetrix analysis (data not shown). This agrees with a recent report suggesting that components from the coagulation cascade could influence cancer progression [24].

A number of genes were also differentially expressed in CRC *vs*. CRA. Most of these genes have not been described in previous microarray studies, although several of the changes agreed to previous reports, including variations in the expression levels of AMN, THBS2, SPP1 or TIMP1 [25], [26], [27]. In addition, 58 probes (19 up- and 39 down-regulated) from the CRC *vs*. CRA comparison were among a list of 248 probes previously identified [11], including that for *AURKA*, which encodes a cell cycle-regulated kinase involved in CRC [28], and was over-expressed in CRC, as compared to CRA and NOR. In addition, among our top deregulated probes, *SPON2*, *RGS16*, *SFRP4* and *CTHRC1* have already been found among the most up-regulated probes in CRC as compared to CRA, and *FAM55D*, *ATOH8*, *RETNLB*, *ID4*, *UGT1A6*, and *VSIG2*, among the most down-regulated probes [11]. It was already shown that some of these genes were deregulated in epithelial cancers or associated with, such as *SFRP4*, *SPON2* [29], *RGS16* [30], or *UGT1A6* [31].

Specific gene expression alterations in either type of colorectal lesions were identified, thanks to intersectional analyses (Figure 2). Firstly, 1,218 (51%) deregulated probes were specific for the NOR to CRA transition, and then, could mark low-risk CRA, because there was no link with CRC. Secondly, 723 (40%) deregulated probes were specific for CRC *vs*. NOR, and then could mark specifically CRC. Finally, 276 (41%) deregulated probes were specific for the CRA to CRC transition. The latter probe set could be interesting to define events specific for the final steps of cancer progression.

The signature of 954 probes corresponded to genes showing expression alterations in both CRA and CRC samples, as compared to normal mucosae. As these deregulated probes in CRC were also abnormally expressed in CRA, they were unlikely candidate markers of the progression from CRA to CRC. Accordingly, the hierarchical clustering did not allow distinguishing CRAs from CRCs. The signature of 172 probes, corresponding to genes deregulated in CRC in comparison to both CRA and NOR, could mark specifically CRC and, supporting this hypothesis, the hierarchical clustering identified the CRCs as a single group. The signature of 265 probes corresponding to genes deregulated in CRC *vs*. CRA, which were already abnormally expressed in CRA *vs*. NOR, was of specific interest because it could mark the progression from NOR to CRA, and then to CRC.

A small number of studies have analyzed the lineage between NOR, CRA and CRC, and the genes differentially expressed between CRA and CRC [13], [25], [26], [27]. One of these studies identified, on an Asian population, an intermediate gene expression signature composed of 463 deregulated probe sets [13]. Twenty seven % (57 out of 215) of the transcripts from our list of 265 probes were identified in this previous signature (45 up- and 12 down-regulated). The limited overlap between both studies could be related to differences between human populations, as already alluded to in a previous study [32]. In order to narrow down this signature of 265 probes, we considered the 44 probes that showed alterations in the three comparisons (CRA *vs*. NOR, CRC *vs*. CRA and CRC *vs*. NOR), and then, were part of all signatures that we identified. The 44 probes corresponded to 8 up- and 32 down-regulated transcripts in both CRA and CRC samples, as compared to normal mucosae. At least 35 out of the 40 transcripts of the signature were previously described in cancer, but only 17 were already associated with colorectal cancer.

Among the over-expressed transcripts in colorectal lesions, *INHBA* has been already identified in the transition from CRA to CRC [13], and its expression has been associated with different cancers, especially with gastric cancer [33]. *PSAT1* was over-expressed in colon tumors, and may be a new target for CRC therapy [34]. It was demonstrated that *TIMP1* increased cell proliferation [35], and may be a CRC candidate marker in serum [36]. The MMP/TIMP system plays a major role in tumor invasion and metastasis, and increased expression of MMPs and TIMPs (observed in our analyses in CRA and CRC) occurred at an early stage of colorectal neoplasia [37]. *SKA3* was required for the maintenance of chromosome cohesion in mitosis [38]. *UBE2S* played a role in the promotion of mitotic exit [39], and *JUB* encodes a cell cycle regulator that interacts with Aurora-A [40].

Among the down-regulated transcripts in colorectal lesions, 20 showed a gradual expression alteration from NOR to CRA, and then, from CRA to CRC, and 12 showed an opposite regulation in the two transition steps, *i.e*. were down-regulated in the NOR to CRA step, and up-regulated in the CRA to CRC step, and then, were less down-regulated in CRC than in CRA, as compared to NOR. Among the transcripts with gradually decreased expression, only *UGT1A6* had been already identified [13]. *SCARA5*, which was proposed as a tumor suppressor gene in hepatocellular carcinoma [41], was down-regulated in various tumor samples [42], and may play a role in colorectal carcinogenesis [43]. Reduction of *NR3C2/MR* expression was already described as a potential early event involved in CRC progression [44]. Five (*CCDC80*, *DPT*, *FBLN1*, *PLN* and *VSIG4*) out of 12 transcripts with increased expression in CRC *vs*. CRA were already found to be up-regulated in CRC as compared to CRA [13]. Reduction of *CCDC80* expression has been observed in colorectal carcinogenesis [45]. *FBLN1* was down-regulated in prostate cancer and in hepatocellular cancer, in which it was proposed as a novel candidate tumor suppressor [46]. *CFH* (complement factor H) might be a novel diagnostic marker for human lung adenocarcinoma [47]. *DACT3* was identified as an epigenetic regulator of the *Wnt* pathway in CRC [48]. *ITIH* genes were down-regulated in multiple human solid tumors, including colon cancer, and may represent a family of putative tumor suppressor genes [49]. *SPARCL1* was associated with a poor prognosis in CRC, and might be a valuable marker for early diagnosis in CRC [50].

The impact of the mRNA expression alteration on the protein level was analyzed by western blotting for a few selected genes among the 40-gene set in both CRA and CRC samples (Supporting Information). The regulation of one up-regulated gene (*TRIB3*), that was already described as a CRC biomarker [51], and four down-regulated genes (*DPT*, *HSD11B2*, *RDH5* and *SMPDL3A*) resulted in a similar regulation of the proteins (Figure S6), showing the potential of these genes as biomarkers. An expected heterogeneity in mRNA and protein expression across colorectal lesions was observed (data not shown), indicating that the expression analysis of these genes could be used to classify CRAs as low- or high-risk to transform into CRC. Nevertheless, it will require several more years to get an appreciation of the functional links between our gene signatures and cancer progression, as our tissue samples have been sampled mostly less than 4 years ago.

Defects in alternative splicing have been implicated in cancer, and alterations in the expression of genes involved in spliceosome assembly were already described in precancerous breast lesions [52]. Our results indicate that changes in splicing profiles in CRA, possibly contributed by modifications in splicing factors, may also be found in CRC, and could define a splicing signature set that could mark the potential for CRA to evolve towards CRC. The alternative splicing events of two genes (*FBLN1* and *ITIH5*) from the 40-gene set (Table S14) were confirmed by quantitative RT-PCR in CRA *vs*. NOR. Specifically, we validated the over-expression of exon 3 and exon e16 for *FBLN1*, and the over-expression of the last exons 13 and 14 for *ITIH5,* in CRAs as compared to normal mucosae (data not shown). Both fibulin-1 (encoded by *FBLN1*) and inter-alpha-trypsin inhibitor heavy chain (encoded by *ITIH5*) are involved in extracellular matrix associations, and both are suppressed in many cancers, including colon cancer, as a consequence of promoter methylation, making the genes putative tumor suppressor genes. The roles played by these alternative splice products occurring in CRAs will require further investigations, together with the other alternative transcripts detected.

In conclusion, our study showed that genes were differentially expressed between colorectal adenomas and adenocarcinomas but, also, to a large extent, between colorectal adenomas and the normal epithelium. We could identify different gene expression signatures, among which one (signature of 44 probes) could be indicative of the CRA patients with the highest potential for developing CRC. The observation that several splicing factors were deregulated in CRA (and CRC) is certainly in line with the recent observations showing that the pre-mRNA splicing machinery may be profoundly remodeled during cancer progression, and may, therefore, play a major role in cancer outcome [53]. Further analyses will be required to determine if these modifications may be predictive markers of the pathological evolution in CRC. Finally, from a systems biology standpoint, it will also be interesting to try to determine if our various gene expression signatures are under some kind of coordination control. This would allow deriving predictive indexes. At a practical level, such indexes could be used to classify patients, at time of adenoma ablation, according to their risk for developing CRC.

## Supporting Information

Figure S1 **Hierarchical clustering considering the gene expression in colorectal adenomas.** Heat map of the expression data was constructed using Euclidean distance with average linkage. The complete heat map of the deregulated probes with a fold-change ≥3.0 and a P-value ≤0.001 is shown for CRA vs. NOR.
(JPG)

Figure S2 **Hierarchical clustering (Euclidean, average linkage) of the colorectal tissues considering the gene expression signature of 954 probes.** Branches represent individual colorectal samples. Different colors were used to identify the sample groups: red, group of normal mucosae (N: normal); green, group of adenomas (A: adenoma); blue, group of adenocarcinomas (C: cancer). The first sample annotation corresponds to the sample group. The subgroups of adenomas are specified: A1, adenomas with areas of micro-invasive adenocarcinomas; A2, adenomas with areas of intra-mucosa adenocarcinomas; A3, adenomas with areas of dysplasia. The second sample annotation corresponds to the sample number. The hierarchical clustering allows distinguishing normal mucosae from colorectal lesions, but not adenomas from adenocarcinomas.
(JPG)

Figure S3 **Hierarchical clustering (Euclidean, average linkage) of the colorectal tissues considering the gene expression signature of 172 probes.** Branches represent individual colorectal samples. Different colors were used to identify the sample groups: red, group of normal mucosae (N: normal); green, group of adenomas (A: adenoma); blue, group of adenocarcinomas (C: cancer). The first sample annotation corresponds to the sample group. The subgroups of adenomas are specified: A1, adenomas with areas of micro-invasive adenocarcinomas; A2, adenomas with areas of intra-mucosa adenocarcinomas; A3, adenomas with areas of dysplasia. The second sample annotation corresponds to the sample number. The hierarchical clustering allowsdistinguishing adenocarcinomas from normal mucosae and adenomas.
(JPG)

Figure S4 **Hierarchical clustering (Euclidean, average linkage) of the colorectal tissues considering the gene expression signature of 265 probes.** Branches represent individual colorectal samples. Different colors were used to identify the sample groups: red, group of normal mucosae (N: normal); green, group of adenomas (A: adenoma); blue, group of adenocarcinomas (C: cancer). The first sample annotation corresponds to the sample group. The subgroups of adenomas are specified: A1, adenomas with areas of micro-invasive adenocarcinomas; A2, adenomas with areas of intra-mucosa adenocarcinomas; A3, adenomas with areas of dysplasia. The second sample annotation corresponds to the sample number. The hierarchical clustering allows distinguishing the three types of colorectal tissues.
(JPG)

Figure S5 **Hierarchical clustering by distance to mean for the Affymetrix analysis.** Twenty four adenoma samples (polyps) were compared to a pool of normal mucosa sample analyzed in duplicate. The hierarchical clustering allows distinguishing the two types of colorectal tissues.
(JPG)

Figure S6 **Western blot analysis of NOR, CRA and CRC samples.** HSD11B2, SMPDL3A, RDH5, Dermatopontin (DPT) and TRIB3 protein levels were analyzed in colorectal adenomas and adenocarcinomas by western blotting. The mRNA levels were analyzed in colorectal lesion samples by quantitative RT-PCR (data not shown), and also validated the results of the Agilent™ microarrays.
(JPG)

Table S1 **Detailed characteristics of colorectal biopsy samples used in the present study.**
(DOC)

Table S2 **Significantly up- and down-regulated genes in colorectal adenoma samples in comparison to normal mucosae.**
(DOC)

Table S3 **Significantly up- and down-regulated genes in colorectal cancer samples in comparison to paired normal mucosae.**
(DOC)

Table S4 **Validation by quantitative Real-Time Polymerase Chain Reaction.**
(DOC)

Table S5 **Validation by PCR arrays of regulations in colorectal cancer samples in comparison to normal mucosae.**
(DOC)

Table S6 **List of the up- and down-regulated genes of the gene expression signature of 954 probes.**
(DOC)

Table S7 **KEGG gene sets enriched in colorectal adenoma samples in comparison to normal mucosae.**
(DOC)

Table S8 **Significantly up- and down-regulated genes in colorectal cancer samples in comparison to colorectal adenoma samples.**
(DOC)

Table S9 **List of the up- and down-regulated genes of the gene expression signature of 172 probes.**
(DOC)

Table S10 **List of the up- and down-regulated genes of the gene expression signature of 265 probes.**
(DOC)

Table S11 **KEGG gene sets enriched in the gene expression signature of 265 probes.**
(DOC)

Table S12 **List of the up- and down-regulated genes in colorectal adenomas in comparison with normal mucosae on Affymetrix™ Human Exon 1.0 ST arrays.**
(DOC)

Table S13 **List of the up- and down-regulated exons in colorectal adenomas in comparison with normal mucosae on Affymetrix Human Exon 1.0 ST arrays.**
(DOC)

Table S14 **List of the deregulated exons in colorectal adenomas in comparison with normal mucosae, for the genes from the Agilent™ gene expression signature of 44 probes.**
(DOC)

File S1 **Supplementary Methods.** MSI, mutation and protein analysis methods.
(DOC)

File S2 **Supplementary Results.** MSI status and mutation analyses.
(DOC)
