# Supplementary figures and images for: A Gene Expression and Pre-mRNA Splicing Signature That Marks the Adenoma-Adenocarcinoma Progression in Colorectal Cancer
Source: PLoS One. 2014 Feb 6;9(2):e87761. doi: 10.1371/journal.pone.0087761 (PMC3916340; doi:10.1371/journal.pone.0087761)

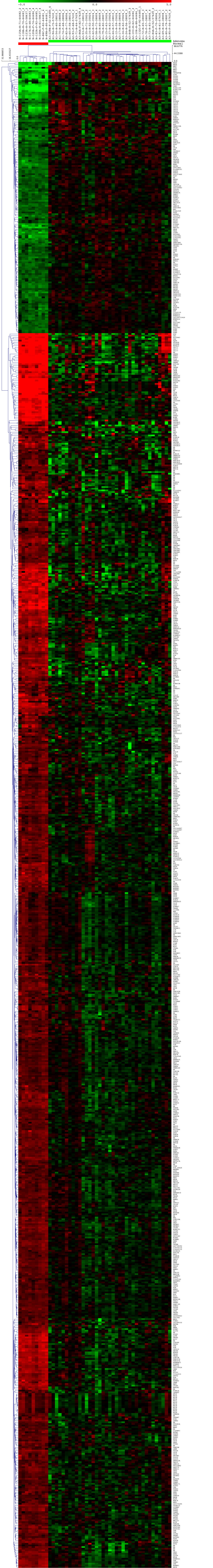

Supplement: Figure S1 — Hierarchical clustering considering the gene expression in colorectal adenomas. Heat map of the expression data was constructed using Euclidean distance with average linkage. The complete heat map of the deregulated probes with a fold-change ≥3.0 and a P-value ≤0.001 is shown for CRA vs. NOR. (JPG) [file pone.0087761.s001.jpg]

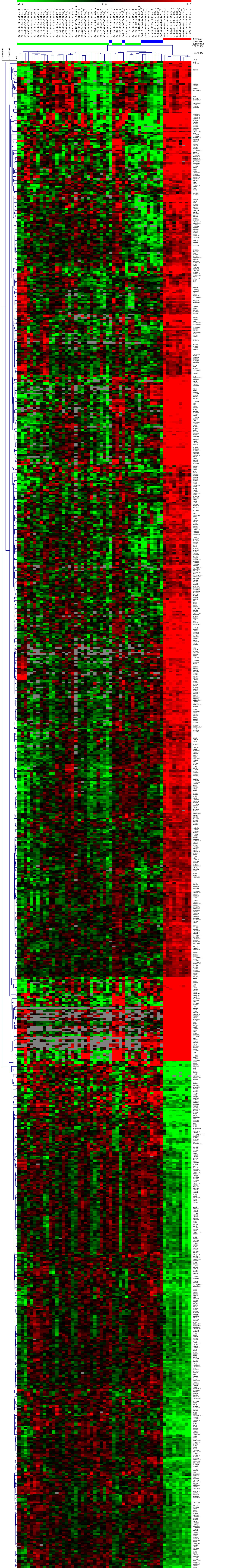

Supplement: Figure S2 — Hierarchical clustering (Euclidean, average linkage) of the colorectal tissues considering the gene expression signature of 954 probes. Branches represent individual colorectal samples. Different colors were used to identify the sample groups: red, group of normal mucosae (N: normal); green, group of adenomas (A: adenoma); blue, group of adenocarcinomas (C: cancer). The first sample annotation corresponds to the sample group. The subgroups of adenomas are specified: A1, adenomas with areas of micro-invasive adenocarcinomas; A2, adenomas with areas of intra-mucosa adenocarcinomas; A3, adenomas with areas of dysplasia. The second sample annotation corresponds to the sample number. The hierarchical clustering allows distinguishing normal mucosae from colorectal lesions, but not adenomas from adenocarcinomas. (JPG) [file pone.0087761.s002.jpg]

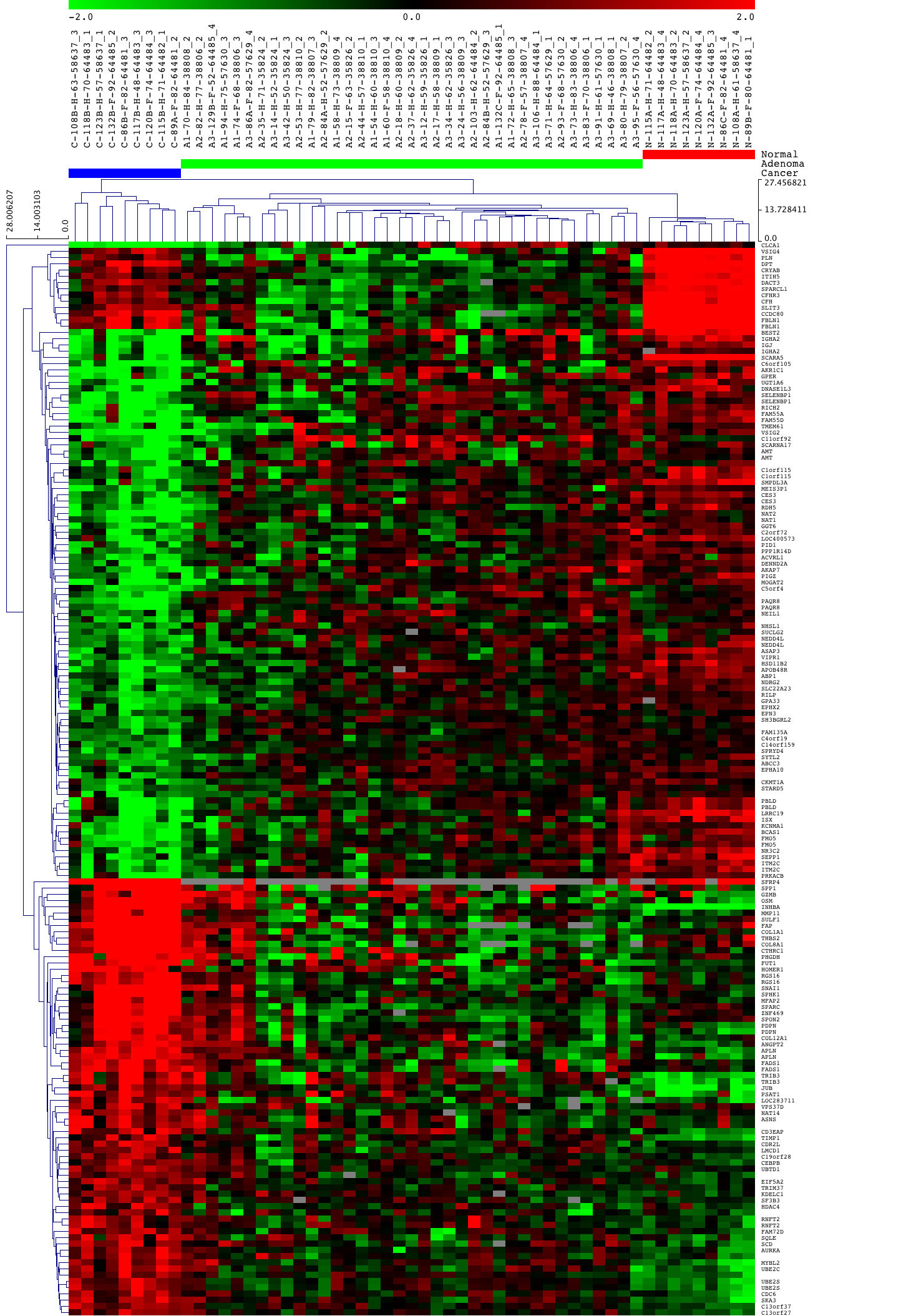

Supplement: Figure S3 — Hierarchical clustering (Euclidean, average linkage) of the colorectal tissues considering the gene expression signature of 172 probes. Branches represent individual colorectal samples. Different colors were used to identify the sample groups: red, group of normal mucosae (N: normal); green, group of adenomas (A: adenoma); blue, group of adenocarcinomas (C: cancer). The first sample annotation corresponds to the sample group. The subgroups of adenomas are specified: A1, adenomas with areas of micro-invasive adenocarcinomas; A2, adenomas with areas of intra-mucosa adenocarcinomas; A3, adenomas with areas of dysplasia. The second sample annotation corresponds to the sample number. The hierarchical clustering allowsdistinguishing adenocarcinomas from normal mucosae and adenomas. (JPG) [file pone.0087761.s003.jpg]

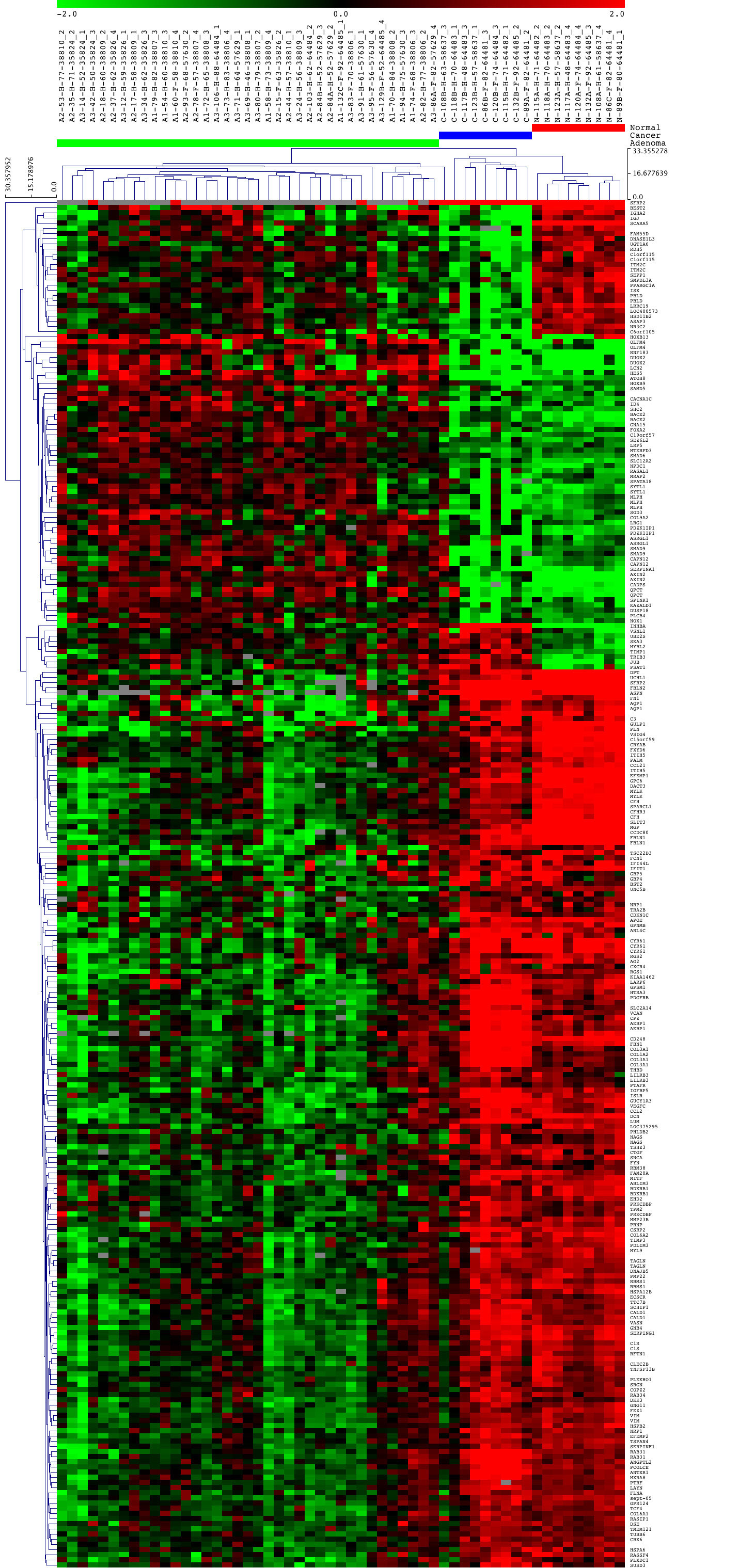

Supplement: Figure S4 — Hierarchical clustering (Euclidean, average linkage) of the colorectal tissues considering the gene expression signature of 265 probes. Branches represent individual colorectal samples. Different colors were used to identify the sample groups: red, group of normal mucosae (N: normal); green, group of adenomas (A: adenoma); blue, group of adenocarcinomas (C: cancer). The first sample annotation corresponds to the sample group. The subgroups of adenomas are specified: A1, adenomas with areas of micro-invasive adenocarcinomas; A2, adenomas with areas of intra-mucosa adenocarcinomas; A3, adenomas with areas of dysplasia. The second sample annotation corresponds to the sample number. The hierarchical clustering allows distinguishing the three types of colorectal tissues. (JPG) [file pone.0087761.s004.jpg]

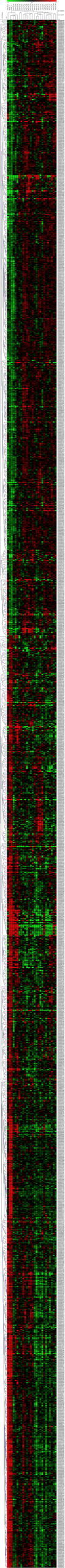

Supplement: Figure S5 — Hierarchical clustering by distance to mean for the Affymetrix analysis. Twenty four adenoma samples (polyps) were compared to a pool of normal mucosa sample analyzed in duplicate. The hierarchical clustering allows distinguishing the two types of colorectal tissues. (JPG) [file pone.0087761.s005.jpg]

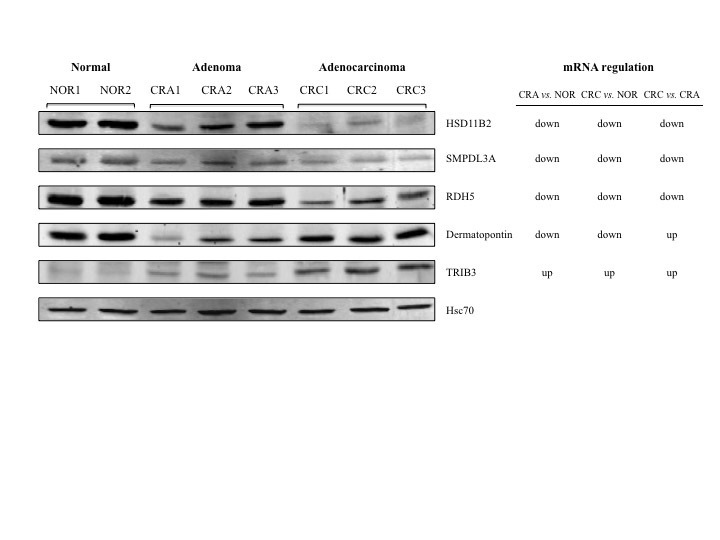

Supplement: Figure S6 — Western blot analysis of NOR, CRA and CRC samples. HSD11B2, SMPDL3A, RDH5, Dermatopontin (DPT) and TRIB3 protein levels were analyzed in colorectal adenomas and adenocarcinomas by western blotting. The mRNA levels were analyzed in colorectal lesion samples by quantitative RT-PCR (data not shown), and also validated the results of the Agilent™ microarrays. (JPG) [file pone.0087761.s006.jpg]
