# Supplementary material for: A Gene Expression and Pre-mRNA Splicing Signature That Marks the Adenoma-Adenocarcinoma Progression in Colorectal Cancer
Source: PLoS One. 2014 Feb 6;9(2):e87761. doi: 10.1371/journal.pone.0087761 (PMC3916340; doi:10.1371/journal.pone.0087761)
Supplement: Table S1 — Detailed characteristics of colorectal biopsy samples used in the present study. (DOC) [file pone.0087761.s007.doc]

**Table S1.** **Detailed characteristics of colorectal biopsy samples used in the present study.** The detailed patient information, such as sample number, patient age, biopsy localization and lesion classification, are presented. The Vienna classification was used for colorectal adenomas, and the TNM (tumors/nodes/metastases) system for colorectal adenocarcinomas. n/a: not available.

| Sample N° | Sample Group | Sample Subgroup | Agilent^TM^ Microarray | Affymetrix^TM^ Microarray | Mutation Analysis | RT-PCR | Western Blotting | Surgery Year | Age (years) | Sex | Biopsy Localization | Vienna Classification or TNM Staging | Lesion Morphology | Lesion Type | Grade of Dysplasia |
| --- | --- | --- | --- | --- | --- | --- | --- | --- | --- | --- | --- | --- | --- | --- | --- |
| 17 | Adenoma | A2 | yes | no | no | no | no | 2006 | 58 | M | Rectosigmoid junction | 4.1 | Pedunculated | Tubular | High |
| 18 | Adenoma | A2 | yes | no | no | no | no | 2006 | 60 | M | Rectum | 4.1 | Pedunculated / Serrated | Tubulovillous | High |
| 14 | Adenoma | A3 | yes | yes | no | no | no | 2006 | 52 | M | Rectum | 4.1 | Pedunculated / Serrated | Tubular | High |
| 15 | Adenoma | A2 | yes | yes | no | no | no | 2006 | 63 | F | Sigmoid colon | 4.1 | Pedunculated | Tubular | High |
| 12 | Adenoma | A3 | yes | yes | no | yes | no | 2006 | 59 | M | Cecum | 3 | Pedunculated | Tubular | Low |
| 24 | Adenoma | A3 | yes | yes | no | yes | no | 2006 | 56 | M | Right colon | 3 | Pedunculated | Tubular | Low |
| 35 | Adenoma | A2 | yes | yes | no | no | no | 2007 | 71 | M | Rectum | 4.1 | Pedunculated | Tubulovillous | High |
| 34 | Adenoma | A3 | yes | no | no | no | no | 2007 | 62 | M | Sigmoid colon | 3 | Pedunculated | Tubular | Low |
| 37 | Adenoma | A2 | yes | no | no | no | no | 2007 | 62 | M | Right colon | 3 / 4.1 | Pedunculated | Tubulovillous | Low / High |
| 42 | Adenoma | A3 | yes | no | no | yes | no | 2007 | 50 | M | Sigmoid colon | 3 | Pedunculated | Tubulovillous | Low |
| 44 | Adenoma | A2 | yes | yes | no | yes | no | 2007 | 57 | M | Sigmoid colon | 4.1 | Flat | Tubulovillous | High |
| 53 | Adenoma | A2 | yes | no | no | no | yes | 2008 | 77 | M | Rectum | 3 / 4.1 | Flat | Tubulovillous | Low / High |
| 54 | Adenoma | A1 | yes | yes | no | yes | no | 2008 | 60 | M | Sigmoid colon | 3 / 4.1 | Pedunculated | Tubulovillous | Low / High |
| 58 | Adenoma | A1 | yes | yes | yes | yes | yes | 2008 | 73 | M | Transversal colon | 3 / 4.1 | Pedunculated | Tubular | Low / High |
| 60 | Adenoma | A1 | yes | yes | yes | yes | no | 2008 | 58 | F | Rectum | 3 / 4.1 | Flat | Tubulovillous | Low / High |
| 80 | Adenoma | A3 | yes | yes | no | yes | no | 2009 | 79 | M | Rectosigmoid junction | 3 | Pedunculated | Tubulovillous | Low |
| 69 | Adenoma | A3 | yes | yes | no | yes | no | 2009 | 46 | M | Rectosigmoid junction | 3 | Pedunculated | Tubulovillous | Low |
| 70 | Adenoma | A1 | yes | yes | no | yes | no | 2009 | 84 | M | Rectosigmoid junction | 4.1 | Pedunculated | Tubular | High |
| 71 | Adenoma | A3 | yes | no | yes | no | yes | 2009 | 64 | M | Rectum | 3 / 4.1 | Pedunculated | Tubulovillous | Low / High |
| 72 | Adenoma | A1 | yes | no | no | yes | no | 2009 | 65 | M | Bauhin's valve | 3 / 4.1 | Pedunculated | Tubulovillous | Low / High |
| 73 | Adenoma | A3 | yes | no | yes | no | yes | 2009 | 83 | M | Left colon | 3 | Pedunculated | Tubulovillous | Low |
| 74 | Adenoma | A1 | yes | yes | yes | yes | no | 2009 | 68 | F | Rectosigmoid junction | 3 / 4.1 | Pedunculated | Tubulovillous | Low / High |
| 76 | Adenoma | A3 | no | no | yes | yes | no | 2009 | 59 | M | Sigmoid colon | 3 | Pedunculated | Tubulovillous | Low |
| 78 | Adenoma | A2 | yes | no | yes | yes | no | 2009 | 57 | F | Rectum | 3 / 4.1 | Sessile | Tubulovillous | Low / High |
| 79 | Adenoma | A1 | yes | yes | yes | yes | yes | 2009 | 82 | M | Right colon | 3 / 4.1 | Sessile | Tubulovillous | Low / High |
| 81 | Adenoma | Out-of-Class | no | yes | yes | no | no | 2009 | 50 | F | Transversal colon | No | Pedunculated | Juvenile Polyposis | No |
| 82 | Adenoma | A2 | yes | yes | yes | yes | no | 2009 | 77 | M | Sigmoid colon | 3 / 4.1 | Pedunculated | Tubulovillous | Low / High |
| 83 | Adenoma | A3 | yes | yes | yes | yes | no | 2009 | 70 | F | Right colon | 3 | Pedunculated | Villous | Low |
| 84A | Adenoma | A2 | yes | no | yes | yes | no | 2009 | 52 | M | Left colon | 4.1 | Pedunculated | Tubulovillous | High |
| 84B | Adenoma | A2 | yes | yes | yes | yes | no | 2009 | 52 | M | Sigmoid colon | 4.1 | Pedunculated | Tubular | High |
| 86A | Adenoma | A3 | yes | yes | yes | yes | no | 2010 | 82 | F | Right colon | 3 / 4.1 | Pedunculated | Tubular | Low / High |
| 91 | Adenoma | A3 | yes | yes | yes | yes | no | 2010 | 61 | M | Rectosigmoid junction | 3 / 4.1 | Pedunculated | Tubulovillous | Low / High |
| 88 | Adenoma | A2 | no | yes | yes | yes | no | 2010 | 92 | M | Rectum | 4.1 | Pedunculated | Tubulovillous | Low / High |
| 94 | Adenoma | A1 | yes | yes | yes | yes | no | 2010 | 75 | M | Cecum | 4.1 | Sessile | Tubulovillous | High |
| 93 | Adenoma | A2 | yes | no | no | no | no | 2010 | 68 | F | Rectum | 4.1 / 4.2 / pTis | Pedunculated | Tubulovillous | High / Carcinoma *in situ* |
| 95 | Adenoma | A3 | yes | yes | yes | yes | no | 2010 | 56 | F | Rectum | 4.1 | Pedunculated | Tubular | High |
| 103 | Adenoma | A2 | yes | yes | no | yes | no | 2010 | 62 | M | Left colon | 4.1 | Pedunculated | Tubulovillous | High |
| 106 | Adenoma | A3 | yes | no | no | yes | no | 2010 | 88 | M | Left colon | 3 | Pedunculated | Tubular | Low |
| 126 | Adenoma | A2 | no | no | no | yes | no | 2011 | 68 | M | Rectum | 3 / 4.1 | Pedunculated | Tubulovillous | Low / High |
| 129B | Adenoma | A3 | yes | no | yes | yes | no | 2011 | 52 | F | Left colon | 4.1 | Pedunculated | Tubular | High |
| 132C | Adenoma | A1 | yes | no | no | yes | no | 2011 | 92 | F | Cecum | n/a | n/a | Tubular | High |
| 139 | Adenoma | A3 | no | no | yes | yes | no | 2011 | 73 | F | Rectosigmoid junction | 3 / 4.1 | Pedunculated | Tubulovillous | Low / High |
| 140B | Adenoma | A3 | no | no | yes | yes | no | 2011 | 75 | F | Right colon | 3 / 4.1 | Sessile | Tubulovillous | Low / High |
| 141 | Adenoma | A3 | no | no | yes | yes | no | 2011 | 53 | M | Sigmoid colon | 3 | Pedunculated | Tubulovillous | Low |
| 144 | Adenoma | A3 | no | no | yes | yes | no | 2011 | 73 | F | Rectosigmoid junction | 4.1 | Pedunculated | Tubular | High |
| 146 | Adenoma | A2 | no | no | yes | yes | no | 2011 | 63 | M | Transversal colon | 3 / 4.1 | Pedunculated | Tubular | Low / High |
| 153 | Adenoma | A3 | no | no | yes | yes | no | 2011 | 82 | F | Rectosigmoid junction | 4.1 | Pedunculated | Tubulovillous | High |
| 154B | Adenoma | n/a | no | no | no | yes | no | 2011 | 48 | F | Left colon | 3 / 4.1 | Pedunculated | Tubulovillous | Low / High |
| 155B | Adenoma | A3 | no | no | yes | yes | no | 2011 | 70 | M | Bauhin's valve | 3 / 4.1 | Sessile | Tubulovillous | Low / High |
| 157C | Adenoma | A2 | no | no | yes | yes | no | 2011 | 76 | M | Bauhin's valve | n/a | n/a | Tubulovillous | Low / High |
| 162 | Adenoma | A1 | no | no | yes | yes | no | 2011 | 74 | M | Left colon | 4.1 | Pedunculated | Villous | High |
| 163 | Adenoma | A3 | no | no | yes | yes | no | 2012 | 60 | M | Right angle | 4.1 | Pedunculated | Villous | High |
| 165C | Adenoma | A3 | no | no | yes | yes | no | 2012 | 70 | M | Sigmoid colon | 4.2 | n/a | Tubulovillous | High |
| 185 | Adenoma | n/a | no | no | no | yes | no | 2012 | 88 | M | Right colon | n/a | n/a | n/a | n/a |
| 192 | Adenoma | n/a | no | no | no | yes | no | 2012 | 58 | M | Sigmoid colon | 3 / 4.1 | Sessile | Tubular | Low / High |
| 86B | Cancer |  | yes | no | no | yes | no | 2010 | 82 | F | Right colon | pT1 N0 Mx (R0) |  |  |  |
| 89A | Cancer |  | yes | no | no | yes | no | 2010 | 80 | F | Rectum | pT4a N2 Mx (R1) |  |  |  |
| 108B | Cancer |  | yes | no | no | yes | no | 2010 | 63 | M | Left colon | pT2 N1 Mx (R0) |  |  |  |
| 115B | Cancer |  | yes | no | no | yes | no | 2010 | 71 | M | Sigmoid colon | pT2 N1 Mx (R0) |  |  |  |
| 117B | Cancer |  | yes | no | no | yes | no | 2010 | 49 | M | Right colon | pT3 N1 Mx (R0) |  |  |  |
| 118B | Cancer |  | yes | no | no | yes | yes | 2010 | 70 | M | Right angle | pT3 N0 Mx (R0) |  |  |  |
| 120B | Cancer |  | yes | no | no | yes | no | 2010 | 74 | F | Right colon | pT3 N0 Mx (R0) |  |  |  |
| 123B | Cancer |  | yes | no | no | yes | yes | 2011 | 57 | M | Cecum | pT3 N2 Mx (R0) |  |  |  |
| 132B | Cancer |  | yes | no | yes | yes | no | 2011 | 92 | F | Cecum | pT3 N0 Mx (R0) |  |  |  |
| 133B | Cancer |  | no | no | yes | no | no | 2011 | 93 | F | Cecum | pT4b N1 Mx (R+) |  |  |  |
| 134B | Cancer |  | no | no | yes | no | no | 2011 | 83 | M | Cecum | pT3 N1 Mx (R0) |  |  |  |
| 135B | Cancer |  | no | no | yes | no | yes | 2011 | 76 | M | Right colon | pT3 N0 Mx (R0) |  |  |  |
| 136B | Cancer |  | no | no | yes | no | yes | 2011 | 73 | M | Sigmoid colon | pT3 N1b Mx (R0) |  |  |  |
| 142B | Cancer |  | no | no | yes | yes | no | 2011 | 86 | M | Transversal colon | pT3 N0 Mx (R0) |  |  |  |
| 147B | Cancer |  | no | no | yes | no | no | 2011 | 60 | M | Right colon | pT3 N0 Mx (R0) |  |  |  |
| 150B | Cancer |  | no | no | yes | yes | no | 2011 | 66 | M | Left colon | pT3 N0 Mx (R0) |  |  |  |
| 156B | Cancer |  | no | no | yes | no | no | 2011 | 64 | M | Cecum | pT2 N0 Mx (R0) |  |  |  |
| 157B | Cancer |  | no | no | yes | no | no | 2011 | 76 | M | Left angle | pT3 N0 Mx (R0) |  |  |  |
| 160B | Cancer |  | no | no | no | yes | no | 2011 | 81 | M | Rectum | pT3 N0 Mx (R0) |  |  |  |
| 164B | Cancer |  | no | no | yes | yes | no | 2012 | 78 | M | Left colon | pT3 N0 Mx (R0) |  |  |  |
| 165B | Cancer |  | no | no | yes | no | no | 2012 | 70 | M | Left colon | pT3 N2a Mx (R0) |  |  |  |
| 179B | Cancer |  | no | no | no | yes | no | 2012 | 74 | F | Left colon | pT4 N0 Mx (R1) |  |  |  |
| 191B | Cancer |  | no | no | no | yes | no | 2012 | 76 | F | Rectum | pT3 N2a Mx (R0) |  |  |  |
| 193B | Cancer |  | no | no | no | yes | no | 2012 | 63 | M | Right colon | pT4 N1b Mx (R0) |  |  |  |
| 190B | Cancer |  | no | no | no | yes | no | 2012 | 50 | M | Right colon | pT3 N2a Mx (R0) |  |  |  |
| 86C | Normal |  | yes | no | no | yes | no | 2010 | 82 | F | Right colon |  |  |  |  |
| 89B | Normal |  | yes | no | no | yes | no | 2010 | 80 | F | Rectum |  |  |  |  |
| 108A | Normal |  | yes | no | no | yes | no | 2010 | 63 | M | Left colon |  |  |  |  |
| 115A | Normal |  | yes | no | no | yes | no | 2010 | 71 | M | Sigmoid colon |  |  |  |  |
| 117A | Normal |  | yes | no | no | yes | no | 2010 | 49 | M | Right colon |  |  |  |  |
| 118A | Normal |  | yes | no | no | yes | no | 2010 | 70 | M | Right angle |  |  |  |  |
| 120A | Normal |  | yes | no | no | yes | no | 2010 | 74 | F | Right colon |  |  |  |  |
| 123A | Normal |  | yes | no | no | yes | no | 2011 | 57 | M | Cecum |  |  |  |  |
| 132A | Normal |  | yes | no | yes | yes | no | 2011 | 92 | F | Cecum |  |  |  |  |
| 133A | Normal |  | no | no | yes | no | no | 2011 | 93 | F | Cecum |  |  |  |  |
| 134A | Normal |  | no | no | yes | no | no | 2011 | 83 | M | Cecum |  |  |  |  |
| 135A | Normal |  | no | no | yes | no | no | 2011 | 76 | M | Right colon |  |  |  |  |
| 136A | Normal |  | no | no | yes | no | yes | 2011 | 73 | M | Sigmoid colon |  |  |  |  |
| 140A | Normal |  | no | no | yes | no | no | 2011 | 75 | F | Right colon |  |  |  |  |
| 142A | Normal |  | no | no | yes | yes | no | 2011 | 86 | M | Transversal colon |  |  |  |  |
| 147A | Normal |  | no | no | yes | no | no | 2011 | 60 | M | Right colon |  |  |  |  |
| 150A | Normal |  | no | no | yes | yes | no | 2011 | 66 | M | Left colon |  |  |  |  |
| 155A | Normal |  | no | no | yes | no | no | 2011 | 70 | M | Left angle |  |  |  |  |
| 156A | Normal |  | no | no | yes | no | no | 2011 | 64 | M | Cecum |  |  |  |  |
| 157A | Normal |  | no | no | yes | no | no | 2011 | 76 | M | Left angle |  |  |  |  |
| 160A | Normal |  | no | no | no | yes | no | 2011 | 81 | M | Rectum |  |  |  |  |
| 164A | Normal |  | no | no | yes | yes | no | 2012 | 78 | M | Left colon |  |  |  |  |
| 165A | Normal |  | no | no | yes | no | no | 2012 | 70 | M | Left colon |  |  |  |  |
| 179A | Normal |  | no | no | no | yes | no | 2012 | 74 | F | Left colon |  |  |  |  |
| 191A | Normal |  | no | no | no | yes | no | 2012 | 76 | F | Rectum |  |  |  |  |
| 193A | Normal |  | no | no | no | yes | yes | 2012 | 63 | M | Right colon |  |  |  |  |
| 190A | Normal |  | no | no | no | yes | no | 2012 | 50 | M | Right colon |  |  |  |  |
