# Supplementary material for: A Gene Expression and Pre-mRNA Splicing Signature That Marks the Adenoma-Adenocarcinoma Progression in Colorectal Cancer
Source: PLoS One. 2014 Feb 6;9(2):e87761. doi: 10.1371/journal.pone.0087761 (PMC3916340; doi:10.1371/journal.pone.0087761)
Supplement: Table S2 — Significantly up- and down-regulated genes in colorectal adenoma samples in comparison to normal mucosae. (DOC) [file pone.0087761.s008.doc]

**Table S2. Significantly up- and down-regulated genes in colorectal adenoma samples in comparison to normal mucosae.** The results of 44k Whole Human Genome microarrays (Agilent) for the deregulated genes in CRA *vs*. NOR are presented (≥ 2.0 FC, P-value ≤ 0.01 by *t*-test with FDR).

| Probe Name | Gene Symbol | P-value | Fold-Change | Regulation |
| --- | --- | --- | --- | --- |
| A_23_P168916 | *CA1* | 1.04E-09 | 294.76 | down |
| A_23_P63032 | *GUCA2B* | 3.67E-10 | 79.97 | down |
| A_23_P11968 | *GUCA2A* | 5.15E-11 | 68.47 | down |
| A_23_P4096 | *CA4* | 2.01E-08 | 58.35 | down |
| A_23_P10127 | *SFRP1* | 1.78E-12 | 56.63 | down |
| A_23_P259863 | *CD177* | 2.25E-08 | 54.58 | down |
| A_23_P102611 | *WISP2* | 6.37E-17 | 53.11 | down |
| A_23_P123228 | *SLC26A3* | 2.31E-08 | 47.04 | down |
| A_32_P143589 | *CD177* | 1.70E-07 | 44.91 | down |
| A_24_P40626 | *GREM2* | 2.04E-12 | 44.43 | down |
| A_23_P251412 | *SCGN* | 1.84E-10 | 43.96 | down |
| A_23_P151895 | *CILP* | 8.95E-20 | 42.73 | down |
| A_23_P26522 | *AQP8* | 8.66E-09 | 38.04 | down |
| A_32_P157213 |  | 8.23E-09 | 36.14 | down |
| A_24_P934546 | *SFRP2* | 5.04E-16 | 34.57 | down |
| A_23_P156708 | *TNXB* | 2.16E-15 | 33.72 | down |
| A_23_P88626 | *ANPEP* | 2.38E-08 | 31.07 | down |
| A_23_P105012 | *HRASLS2* | 4.52E-11 | 30.06 | down |
| A_23_P10121 | *SFRP1* | 6.26E-11 | 30.04 | down |
| A_23_P8812 |  | 1.70E-14 | 29.42 | down |
| A_23_P259868 | *CD177* | 1.88E-08 | 28.30 | down |
| A_23_P200741 | *DPT* | 6.20E-18 | 27.31 | down |
| A_32_P27046 | *CHGA* | 2.04E-14 | 26.40 | down |
| A_23_P18672 | *GBA3* | 1.74E-11 | 25.94 | down |
| A_23_P13713 | *PRPH* | 1.77E-14 | 25.32 | down |
| A_24_P168925 | *CHRDL1* | 3.67E-18 | 24.87 | down |
| A_23_P8913 | *CA2* | 6.97E-09 | 24.72 | down |
| A_23_P19650 | *VIP* | 5.44E-10 | 24.20 | down |
| A_23_P106656 | *CA7* | 1.77E-14 | 23.63 | down |
| A_23_P26965 | *CCL13* | 4.93E-09 | 23.08 | down |
| A_23_P130573 | *CEACAM7* | 1.52E-07 | 22.60 | down |
| A_24_P137501 | *SFRP2* | 2.27E-11 | 22.56 | down |
| A_24_P300379 | *PI16* | 4.59E-18 | 22.20 | down |
| A_23_P207456 | *CCL8* | 9.11E-16 | 21.32 | down |
| A_24_P125335 | *CCL13* | 3.64E-10 | 21.02 | down |
| A_23_P18713 | *ABCG2* | 1.09E-13 | 20.93 | down |
| A_23_P4112 | *PYY* | 1.27E-12 | 20.89 | down |
| A_23_P127760 | *MS4A12* | 1.31E-06 | 20.67 | down |
| A_24_P364263 | *HRASLS2* | 2.88E-09 | 20.05 | down |
| A_23_P57709 | *PCOLCE2* | 1.81E-13 | 20.01 | down |
| A_32_P358887 | *SLC4A4* | 1.90E-08 | 19.80 | down |
| A_23_P436284 | *OSTBETA* | 6.73E-11 | 19.63 | down |
| A_23_P216712 | *TRPM6* | 1.32E-08 | 19.46 | down |
| A_23_P254664 | *GCG* | 1.75E-06 | 19.25 | down |
| A_23_P140384 | *CTSG* | 6.29E-10 | 17.55 | down |
| A_23_P143981 | *FBLN2* | 3.31E-15 | 17.21 | down |
| A_23_P97181 | *GREM2* | 2.36E-15 | 17.02 | down |
| A_23_P150457 | *LYVE1* | 4.59E-18 | 16.20 | down |
| A_23_P202448 | *CXCL12* | 3.92E-14 | 15.80 | down |
| A_23_P148879 | *ATP1A2* | 8.68E-17 | 15.35 | down |
| A_23_P58082 | *CCDC80* | 7.18E-14 | 15.11 | down |
| A_32_P140139 | *F13A1* | 9.48E-11 | 14.75 | down |
| A_23_P250951 | *SLC26A2* | 9.25E-07 | 14.72 | down |
| A_32_P166653 |  | 1.24E-08 | 14.67 | down |
| A_24_P360674 | *CDKN2B* | 9.94E-11 | 14.64 | down |
| A_23_P212968 | *UGT2B11* | 5.07E-07 | 14.55 | down |
| A_24_P17691 | *UGT2B17* | 8.11E-05 | 14.42 | down |
| A_23_P382584 | *CHGB* | 1.03E-09 | 14.31 | down |
| A_23_P58407 | *UGT2B15* | 4.94E-05 | 13.82 | down |
| A_23_P256425 | *ADAMDEC1* | 2.51E-14 | 13.63 | down |
| A_23_P407695 | *FAM151A* | 2.68E-15 | 13.53 | down |
| A_23_P211631 | *FBLN1* | 1.36E-11 | 13.36 | down |
| A_23_P69497 | *CLEC3B* | 1.79E-13 | 13.29 | down |
| A_24_P643776 |  | 1.45E-14 | 13.22 | down |
| A_23_P132956 | *UCHL1* | 1.11E-10 | 13.21 | down |
| A_24_P131622 | *FAM107A* | 2.84E-12 | 13.01 | down |
| A_23_P433016 | *FBLN1* | 8.05E-13 | 12.99 | down |
| A_23_P81103 | *SFRP2* | 6.95E-10 | 12.95 | down |
| A_23_P414793 | *CP* | 3.72E-17 | 12.58 | down |
| A_23_P408249 | *PCK1* | 6.39E-07 | 12.40 | down |
| A_23_P58588 | *SLIT3* | 3.42E-14 | 12.05 | down |
| A_23_P146134 | *DUSP26* | 1.25E-11 | 11.98 | down |
| A_24_P70183 | *MYH11* | 5.00E-08 | 11.65 | down |
| A_23_P212050 | *BCHE* | 6.31E-08 | 11.64 | down |
| A_23_P39067 | *SPIB* | 5.40E-14 | 11.62 | down |
| A_23_P94103 | *SCARA5* | 1.04E-14 | 11.54 | down |
| A_23_P303087 | *PTN* | 1.32E-10 | 11.47 | down |
| A_23_P164057 | *MFAP4* | 5.90E-10 | 11.43 | down |
| A_23_P58359 | *ADH1A* | 1.57E-05 | 11.37 | down |
| A_23_P21092 | *CALB2* | 7.65E-12 | 11.32 | down |
| A_23_P206920 | *MYH11* | 1.55E-07 | 11.13 | down |
| A_24_P395415 |  | 2.61E-06 | 11.06 | down |
| A_32_P524904 | *C11orf86* | 5.21E-06 | 11.02 | down |
| A_24_P325992 | *LIFR* | 6.65E-11 | 10.93 | down |
| A_23_P146274 | *STMN2* | 1.41E-07 | 10.90 | down |
| A_23_P500400 | *ABCA6* | 1.10E-17 | 10.90 | down |
| A_23_P165457 | *CRYBA2* | 6.32E-12 | 10.65 | down |
| A_23_P90710 | *DES* | 7.76E-07 | 10.64 | down |
| A_24_P291658 | *ADH1A* | 9.46E-06 | 10.60 | down |
| A_23_P84860 | *FAM107A* | 3.78E-14 | 10.50 | down |
| A_23_P372308 | *RGMA* | 2.78E-12 | 10.36 | down |
| A_23_P349463 | *CHP2* | 1.45E-06 | 10.35 | down |
| A_23_P17438 | *EDN3* | 1.24E-07 | 10.32 | down |
| A_23_P101992 | *MARCO* | 1.36E-10 | 10.24 | down |
| A_32_P197561 | *EBF1* | 2.15E-11 | 10.16 | down |
| A_23_P81590 | *PDE6A* | 8.94E-08 | 10.12 | down |
| A_23_P56559 | *DHRS9* | 1.31E-06 | 10.11 | down |
| A_23_P217498 | *GDPD2* | 1.75E-09 | 9.65 | down |
| A_23_P338919 | *SPEG* | 1.02E-06 | 9.63 | down |
| A_24_P870620 | *PTN* | 1.88E-11 | 9.60 | down |
| A_23_P369994 | *DCLK1* | 2.38E-20 | 9.57 | down |
| A_23_P22205 | *SLC4A4* | 2.11E-08 | 9.57 | down |
| A_23_P501007 | *EFEMP1* | 2.77E-12 | 9.54 | down |
| A_23_P163336 | *CA12* | 6.16E-11 | 9.54 | down |
| A_23_P204286 | *MGP* | 1.77E-14 | 9.50 | down |
| A_23_P111531 | *GLI3* | 5.42E-13 | 9.40 | down |
| A_23_P81158 | *ADH1C* | 5.12E-05 | 9.37 | down |
| A_32_P48256 |  | 2.83E-10 | 9.28 | down |
| A_23_P164436 | *ASPA* | 2.76E-13 | 9.22 | down |
| A_23_P103256 | *CFHR3* | 8.67E-16 | 9.06 | down |
| A_23_P7727 | *HAPLN1* | 1.75E-09 | 9.00 | down |
| A_23_P56433 | *C2orf40* | 1.60E-20 | 8.91 | down |
| A_32_P169353 |  | 1.62E-05 | 8.83 | down |
| A_23_P399265 | *STMN2* | 3.52E-07 | 8.78 | down |
| A_32_P171043 | *CCBE1* | 7.51E-20 | 8.71 | down |
| A_24_P74828 | *MT1JP* | 1.05E-04 | 8.65 | down |
| A_23_P200160 | *CFH* | 3.65E-11 | 8.65 | down |
| A_23_P41528 | *FGFBP2* | 3.47E-14 | 8.64 | down |
| A_23_P87700 | *MFAP5* | 1.80E-08 | 8.61 | down |
| A_23_P139635 | *DAO* | 2.68E-09 | 8.52 | down |
| A_23_P123853 | *CCL19* | 2.56E-06 | 8.51 | down |
| A_23_P103812 | *FAM5C* | 4.11E-08 | 8.47 | down |
| A_23_P217269 | *VSIG4* | 3.01E-10 | 8.43 | down |
| A_23_P15450 | *TMEM100* | 1.75E-13 | 8.36 | down |
| A_23_P56197 | *CRLF1* | 1.62E-10 | 8.35 | down |
| A_24_P319736 | *MEIS1* | 1.77E-14 | 8.30 | down |
| A_23_P7342 | *UGT2B10* | 5.37E-07 | 8.29 | down |
| A_23_P253911 | *CHRNA3* | 2.57E-06 | 8.25 | down |
| A_23_P64873 | *DCN* | 3.30E-09 | 8.23 | down |
| A_23_P500421 | *EYA2* | 1.37E-03 | 8.21 | down |
| A_32_P97169 | *GPC6* | 1.05E-12 | 8.14 | down |
| A_32_P86578 | *LOC389023* | 2.19E-03 | 8.13 | down |
| A_23_P107116 | *RNF112* | 3.21E-14 | 8.12 | down |
| A_23_P104252 | *ITIH5* | 4.52E-10 | 8.12 | down |
| A_23_P30614 | *PLN* | 2.07E-07 | 8.08 | down |
| A_23_P57155 | *CHGB* | 7.07E-09 | 8.05 | down |
| A_23_P420442 | *SEMA6D* | 2.31E-08 | 8.03 | down |
| A_23_P96383 | *SRPX* | 3.78E-14 | 8.00 | down |
| A_32_P310335 | *JAM2* | 5.95E-13 | 7.93 | down |
| A_23_P153390 | *CLEC4G* | 1.45E-14 | 7.92 | down |
| A_32_P176819 | *CMAH* | 4.79E-11 | 7.91 | down |
| A_23_P96501 | *TEX11* | 1.54E-09 | 7.81 | down |
| A_24_P8116 | *CCDC80* | 7.49E-19 | 7.80 | down |
| A_23_P110764 | *MYOT* | 6.62E-17 | 7.72 | down |
| A_23_P114740 | *CFH* | 2.96E-11 | 7.69 | down |
| A_32_P210168 | *C15orf59* | 1.30E-15 | 7.69 | down |
| A_32_P88965 | *DDR2* | 2.35E-12 | 7.67 | down |
| A_23_P145529 | *PKIB* | 6.31E-06 | 7.64 | down |
| A_32_P112592 | *LOC339524* | 5.62E-18 | 7.61 | down |
| A_23_P136753 |  | 8.92E-06 | 7.61 | down |
| A_23_P17130 | *C2orf88* | 3.83E-10 | 7.58 | down |
| A_24_P48204 | *SECTM1* | 1.20E-07 | 7.56 | down |
| A_23_P125233 | *CNN1* | 1.79E-07 | 7.49 | down |
| A_23_P216429 | *ASPN* | 5.29E-12 | 7.49 | down |
| A_23_P130333 | *TTR* | 9.95E-07 | 7.48 | down |
| A_24_P709844 |  | 6.74E-09 | 7.46 | down |
| A_23_P106773 | *SULT1A2* | 3.96E-09 | 7.45 | down |
| A_23_P120667 | *JAM2* | 2.06E-11 | 7.44 | down |
| A_23_P139146 | *MS4A8B* | 5.61E-06 | 7.43 | down |
| A_23_P27306 | *COLEC12* | 6.54E-12 | 7.34 | down |
| A_23_P209232 | *CLIP4* | 9.05E-10 | 7.31 | down |
| A_23_P6321 | *CLDN5* | 3.96E-10 | 7.30 | down |
| A_23_P144348 | *SLIT2* | 5.33E-10 | 7.30 | down |
| A_23_P46426 | *CYR61* | 1.09E-08 | 7.19 | down |
| A_24_P20292 | *B3GNT7* | 7.18E-05 | 7.15 | down |
| A_23_P17134 | *MAL* | 4.64E-11 | 7.14 | down |
| A_24_P56240 | *CPNE8* | 3.68E-08 | 7.10 | down |
| A_23_P51787 | *AMPD1* | 3.78E-07 | 7.09 | down |
| A_23_P38735 | *CDH19* | 1.35E-06 | 7.05 | down |
| A_23_P26511 | *GDPD3* | 3.55E-08 | 7.05 | down |
| A_23_P332399 | *GULP1* | 7.81E-07 | 7.04 | down |
| A_23_P426305 | *AOC3* | 1.79E-13 | 6.96 | down |
| A_32_P44878 | *ITIH5* | 2.43E-16 | 6.90 | down |
| A_23_P46429 | *CYR61* | 1.64E-09 | 6.87 | down |
| A_24_P71904 | *HPGD* | 3.12E-07 | 6.78 | down |
| A_24_P164998 | *LOC646627* | 2.86E-06 | 6.78 | down |
| A_23_P66241 | *MT1M* | 3.62E-04 | 6.77 | down |
| A_23_P216361 | *COL14A1* | 2.72E-09 | 6.77 | down |
| A_24_P165864 | *P2RY14* | 1.03E-09 | 6.76 | down |
| A_32_P106615 |  | 7.90E-10 | 6.75 | down |
| A_23_P86461 | *PLAC9* | 1.38E-17 | 6.73 | down |
| A_24_P379413 | *IL6R* | 1.52E-08 | 6.72 | down |
| A_24_P370946 | *CYR61* | 6.49E-08 | 6.71 | down |
| A_23_P211468 |  | 5.96E-07 | 6.68 | down |
| A_23_P134854 | *CLDN23* | 1.80E-13 | 6.67 | down |
| A_32_P53524 | *NTN1* | 7.66E-09 | 6.66 | down |
| A_32_P171225 |  | 1.78E-08 | 6.66 | down |
| A_23_P378416 | *GPM6B* | 3.40E-32 | 6.65 | down |
| A_24_P260443 | *THBS4* | 4.16E-11 | 6.65 | down |
| A_23_P4387 | *KRT24* | 6.34E-10 | 6.58 | down |
| A_23_P68669 | *CHODL* | 6.90E-09 | 6.56 | down |
| A_23_P39294 | *PLAC2* | 7.49E-06 | 6.54 | down |
| A_23_P94434 | *HRCT1* | 6.83E-07 | 6.46 | down |
| A_32_P11262 |  | 2.69E-08 | 6.46 | down |
| A_32_P136295 | *GNG7* | 2.45E-09 | 6.45 | down |
| A_23_P310094 | *SYNPO2* | 5.30E-11 | 6.44 | down |
| A_23_P51499 | *PRELP* | 4.17E-14 | 6.43 | down |
| A_23_P206733 | *CES1* | 4.11E-08 | 6.43 | down |
| A_24_P310256 | *LGI4* | 5.13E-07 | 6.42 | down |
| A_23_P66637 | *SGCA* | 1.89E-12 | 6.41 | down |
| A_23_P155688 | *SPINK2* | 1.32E-07 | 6.39 | down |
| A_23_P210158 |  | 9.52E-07 | 6.37 | down |
| A_23_P108216 | *FXYD1* | 1.09E-09 | 6.35 | down |
| A_23_P162668 | *CPM* | 1.27E-09 | 6.33 | down |
| A_23_P118065 | *HSD17B2* | 2.08E-06 | 6.33 | down |
| A_23_P10506 | *HPGDS* | 2.32E-08 | 6.29 | down |
| A_23_P360964 | *DACT3* | 3.82E-12 | 6.29 | down |
| A_23_P300220 | *YPEL4* | 4.08E-11 | 6.28 | down |
| A_23_P256470 | *NPY* | 1.81E-12 | 6.26 | down |
| A_23_P112470 | *CCL21* | 1.04E-09 | 6.20 | down |
| A_23_P119562 | *CFD* | 2.72E-05 | 6.19 | down |
| A_24_P79300 | *CLDN5* | 1.75E-09 | 6.17 | down |
| A_23_P105803 | *FGF9* | 7.57E-09 | 6.17 | down |
| A_24_P106542 | *RSPO3* | 5.05E-05 | 6.15 | down |
| A_23_P26640 | *C16orf89* | 1.44E-06 | 6.12 | down |
| A_24_P267523 | *COX6B2* | 7.26E-06 | 6.11 | down |
| A_23_P113351 | *SPARCL1* | 2.28E-11 | 6.11 | down |
| A_23_P76749 | *GALNTL1* | 1.15E-08 | 6.06 | down |
| A_23_P160159 | *SLC2A5* | 1.06E-07 | 6.04 | down |
| A_23_P52697 | *CD248* | 1.79E-09 | 6.02 | down |
| A_23_P158297 | *BTNL3* | 2.56E-05 | 6.01 | down |
| A_24_P938352 | *CPM* | 5.88E-09 | 6.00 | down |
| A_23_P156890 | *TCF21* | 3.01E-10 | 5.96 | down |
| A_23_P216307 | *RUNX1T1* | 2.42E-09 | 5.96 | down |
| A_23_P111583 | *CD36* | 5.23E-11 | 5.95 | down |
| A_23_P217688 | *TSC22D3* | 1.24E-06 | 5.95 | down |
| A_23_P12363 | *ROR1* | 9.55E-09 | 5.94 | down |
| A_23_P101407 | *C3* | 5.13E-05 | 5.88 | down |
| A_24_P113926 | *DPT* | 4.65E-18 | 5.87 | down |
| A_24_P119745 | *FN1* | 3.36E-04 | 5.86 | down |
| A_23_P218858 | *ABI3BP* | 5.29E-12 | 5.85 | down |
| A_23_P389987 | *TLX2* | 3.69E-15 | 5.84 | down |
| A_23_P110319 | *CWH43* | 1.03E-03 | 5.79 | down |
| A_24_P319923 | *MYLK* | 6.98E-10 | 5.79 | down |
| A_32_P76137 |  | 6.22E-04 | 5.78 | down |
| A_23_P144916 | *GFPT2* | 1.29E-08 | 5.77 | down |
| A_24_P34865 | *BTNL3* | 6.00E-06 | 5.77 | down |
| A_23_P81219 | *PLAC8* | 1.84E-06 | 5.75 | down |
| A_23_P133474 | *GPX3* | 8.85E-17 | 5.75 | down |
| A_24_P233786 | *FAM129A* | 1.31E-09 | 5.75 | down |
| A_23_P111860 | *RADIL* | 2.55E-05 | 5.74 | down |
| A_24_P183128 | *PLAC8* | 1.72E-06 | 5.72 | down |
| A_23_P55738 | *CEACAM1* | 3.61E-06 | 5.70 | down |
| A_24_P206776 | *CRYAB* | 2.31E-15 | 5.69 | down |
| A_23_P142560 | *ZEB2* | 2.05E-13 | 5.68 | down |
| A_32_P32413 | *SETBP1* | 3.79E-09 | 5.66 | down |
| A_32_P61298 |  | 7.61E-11 | 5.65 | down |
| A_24_P382319 | *CEACAM1* | 2.55E-08 | 5.65 | down |
| A_32_P213103 |  | 1.55E-09 | 5.64 | down |
| A_23_P164958 | *CCDC8* | 1.08E-13 | 5.64 | down |
| A_23_P70719 | *LAMA2* | 5.43E-08 | 5.59 | down |
| A_23_P250102 | *CAND2* | 5.45E-10 | 5.58 | down |
| A_23_P49192 | *CES2* | 2.95E-08 | 5.58 | down |
| A_23_P21324 | *TWIST2* | 7.82E-09 | 5.58 | down |
| A_23_P99515 | *C13orf33* | 8.78E-09 | 5.55 | down |
| A_24_P228302 | *CEACAM7* | 2.99E-06 | 5.53 | down |
| A_32_P78681 | *GLP2R* | 9.14E-09 | 5.53 | down |
| A_24_P413286 |  | 1.99E-04 | 5.52 | down |
| A_23_P418234 | *PHLPP2* | 1.28E-12 | 5.51 | down |
| A_23_P21907 |  | 3.14E-08 | 5.48 | down |
| A_23_P434118 | *CEACAM1* | 2.34E-06 | 5.47 | down |
| A_32_P208403 | *GNG2* | 6.89E-13 | 5.44 | down |
| A_23_P319859 | *EYA2* | 1.23E-03 | 5.41 | down |
| A_23_P4773 | *LILRB5* | 5.38E-09 | 5.38 | down |
| A_24_P263786 |  | 3.74E-03 | 5.38 | down |
| A_23_P11843 | *LRRN2* | 6.45E-07 | 5.38 | down |
| A_23_P41987 | *GFRA3* | 1.83E-09 | 5.38 | down |
| A_32_P30966 |  | 8.49E-06 | 5.38 | down |
| A_23_P13907 | *IGF1* | 2.17E-11 | 5.37 | down |
| A_23_P120243 | *HOXD1* | 1.08E-06 | 5.37 | down |
| A_23_P141505 | *CLEC10A* | 1.02E-10 | 5.33 | down |
| A_24_P104407 | *SYNM* | 1.74E-10 | 5.32 | down |
| A_23_P117662 | *HDC* | 3.38E-06 | 5.27 | down |
| A_24_P85539 | *FN1* | 3.43E-04 | 5.27 | down |
| A_24_P120907 | *PGM5* | 1.99E-06 | 5.26 | down |
| A_32_P42329 |  | 9.40E-11 | 5.25 | down |
| A_32_P213418 |  | 7.04E-10 | 5.25 | down |
| A_23_P21393 |  | 4.46E-07 | 5.24 | down |
| A_23_P139912 | *IGFBP6* | 4.84E-09 | 5.23 | down |
| A_23_P95640 | *C1orf186* | 2.40E-09 | 5.21 | down |
| A_32_P46594 | *LOC145837* | 4.52E-05 | 5.17 | down |
| A_24_P308506 | *NAT8B* | 1.02E-06 | 5.16 | down |
| A_23_P96965 | *SYNC* | 1.51E-10 | 5.16 | down |
| A_23_P371495 | *TMTC1* | 1.30E-10 | 5.15 | down |
| A_23_P143817 | *MYLK* | 8.44E-10 | 5.12 | down |
| A_23_P19182 | *REEP2* | 5.29E-12 | 5.12 | down |
| A_24_P357465 | *TP53INP2* | 2.83E-09 | 5.11 | down |
| A_23_P48561 | *EFS* | 3.57E-13 | 5.10 | down |
| A_23_P52266 | *IFIT1* | 2.42E-07 | 5.10 | down |
| A_23_P4551 | *SETBP1* | 5.86E-08 | 5.09 | down |
| A_24_P16004 |  | 2.55E-03 | 5.09 | down |
| A_24_P152845 | *LOC340888* | 5.13E-05 | 5.09 | down |
| A_23_P135548 | *DPYD* | 1.01E-11 | 5.07 | down |
| A_23_P411993 | *ITIH5* | 3.16E-12 | 5.07 | down |
| A_23_P151915 | *GCNT3* | 6.39E-06 | 5.07 | down |
| A_23_P212500 | *TF* | 3.05E-13 | 5.05 | down |
| A_23_P66854 | *KRT20* | 2.31E-06 | 5.05 | down |
| A_23_P83028 | *RECK* | 2.30E-10 | 5.05 | down |
| A_23_P19673 | *SGK1* | 3.88E-08 | 5.05 | down |
| A_23_P170830 |  | 4.66E-03 | 5.04 | down |
| A_32_P46214 | *SLC9A9* | 4.70E-11 | 5.04 | down |
| A_24_P538459 |  | 1.33E-03 | 5.03 | down |
| A_32_P30004 |  | 2.89E-09 | 5.03 | down |
| A_32_P152437 | *AKAP12* | 2.00E-11 | 5.02 | down |
| A_23_P27005 | *DHRS11* | 8.19E-12 | 5.02 | down |
| A_32_P83098 | *SCNN1B* | 6.06E-06 | 5.01 | down |
| A_24_P384604 |  | 1.15E-03 | 4.99 | down |
| A_24_P409971 | *NEXN* | 1.08E-06 | 4.97 | down |
| A_23_P22735 | *BEX2* | 3.91E-03 | 4.96 | down |
| A_24_P154868 | *MEP1A* | 6.26E-06 | 4.96 | down |
| A_23_P138352 | *WNT2B* | 4.32E-11 | 4.96 | down |
| A_24_P304419 | *IGF1* | 6.66E-11 | 4.95 | down |
| A_24_P177844 |  | 1.60E-05 | 4.94 | down |
| A_23_P136724 | *LOC344887* | 9.40E-08 | 4.93 | down |
| A_23_P105144 | *SCUBE2* | 8.15E-05 | 4.93 | down |
| A_23_P95851 | *TUBAL3* | 3.16E-06 | 4.93 | down |
| A_23_P85240 | *TLR7* | 2.46E-09 | 4.92 | down |
| A_24_P272310 | *MUSTN1* | 6.76E-10 | 4.92 | down |
| A_23_P416774 | *CLIC5* | 1.56E-09 | 4.92 | down |
| A_24_P203886 |  | 4.34E-04 | 4.89 | down |
| A_23_P45324 | *TMEM35* | 4.47E-05 | 4.87 | down |
| A_24_P575267 |  | 5.51E-05 | 4.87 | down |
| A_23_P52425 | *NKX2-3* | 8.00E-09 | 4.86 | down |
| A_23_P115417 | *RGL1* | 6.17E-11 | 4.85 | down |
| A_23_P212354 | *CCR2* | 6.25E-07 | 4.84 | down |
| A_23_P356494 | *SPINK5* | 6.63E-04 | 4.83 | down |
| A_23_P420209 | *GCNT3* | 2.95E-05 | 4.82 | down |
| A_24_P592400 | *LOC553137* | 2.86E-07 | 4.81 | down |
| A_23_P1904 | *MS4A2* | 5.33E-08 | 4.81 | down |
| A_24_P548866 | *HIGD1A* | 2.13E-06 | 4.81 | down |
| A_23_P164650 | *APOE* | 1.46E-06 | 4.80 | down |
| A_23_P310956 | *COL6A2* | 1.16E-09 | 4.80 | down |
| A_23_P414343 | *MT1H* | 9.82E-07 | 4.79 | down |
| A_23_P61466 | *CD163L1* | 3.83E-10 | 4.77 | down |
| A_23_P127911 | *PAMR1* | 2.87E-08 | 4.76 | down |
| A_23_P42975 | *PRKAR2B* | 3.88E-08 | 4.76 | down |
| A_23_P107351 | *LOC728392* | 2.12E-10 | 4.74 | down |
| A_24_P307375 |  | 1.26E-03 | 4.74 | down |
| A_23_P97841 | *ITIH5* | 1.28E-12 | 4.74 | down |
| A_24_P816844 |  | 9.47E-05 | 4.74 | down |
| A_24_P914669 |  | 2.24E-11 | 4.73 | down |
| A_23_P162579 | *HSPB8* | 1.61E-11 | 4.72 | down |
| A_24_P503710 | *TLCD2* | 1.36E-07 | 4.72 | down |
| A_23_P93641 | *AKR1B10* | 3.84E-04 | 4.72 | down |
| A_23_P48585 | *SALL2* | 1.24E-11 | 4.71 | down |
| A_23_P427703 | *MT1L* | 1.36E-06 | 4.70 | down |
| A_23_P66017 | *PRRT2* | 1.78E-08 | 4.70 | down |
| A_24_P788772 |  | 1.67E-05 | 4.69 | down |
| A_23_P150394 | *FXYD6* | 1.43E-14 | 4.69 | down |
| A_23_P153616 | *MADCAM1* | 3.22E-04 | 4.69 | down |
| A_23_P39955 | *ACTG2* | 1.27E-05 | 4.69 | down |
| A_24_P129107 | *NKD2* | 9.46E-03 | 4.68 | down |
| A_24_P11825 | *CCR2* | 2.98E-06 | 4.68 | down |
| A_23_P60933 | *MT1G* | 5.60E-06 | 4.68 | down |
| A_24_P80204 | *MALL* | 1.85E-06 | 4.67 | down |
| A_24_P163237 | *STOX2* | 3.37E-07 | 4.66 | down |
| A_23_P47924 | *PTPRR* | 4.41E-05 | 4.65 | down |
| A_23_P85441 | *IGSF9* | 1.15E-07 | 4.65 | down |
| A_23_P37736 | *TNFRSF17* | 1.32E-03 | 4.64 | down |
| A_32_P212024 |  | 2.83E-10 | 4.63 | down |
| A_23_P58993 | *MOCS1* | 2.85E-06 | 4.62 | down |
| A_24_P129341 | *AKR1B10* | 5.26E-04 | 4.62 | down |
| A_23_P22487 | *PCSK1N* | 4.40E-04 | 4.60 | down |
| A_24_P204374 |  | 2.79E-03 | 4.60 | down |
| A_23_P303833 | *SCN4B* | 3.83E-10 | 4.59 | down |
| A_24_P233078 | *PYY2* | 4.28E-04 | 4.59 | down |
| A_23_P22682 | *ARMCX1* | 1.03E-11 | 4.58 | down |
| A_23_P17481 | *SIGLEC1* | 9.20E-13 | 4.57 | down |
| A_23_P207905 | *SECTM1* | 5.75E-08 | 4.57 | down |
| A_23_P257003 | *PCSK5* | 2.75E-10 | 4.57 | down |
| A_23_P211039 | *ADAMTS1* | 9.72E-08 | 4.57 | down |
| A_32_P57486 |  | 1.72E-05 | 4.55 | down |
| A_24_P234768 | *HTR4* | 3.99E-05 | 4.53 | down |
| A_23_P210060 | *C2orf88* | 3.39E-11 | 4.53 | down |
| A_23_P378364 | *PCDH7* | 3.36E-12 | 4.52 | down |
| A_23_P54918 | *LDHD* | 1.65E-04 | 4.51 | down |
| A_23_P392541 | *SPG20* | 7.47E-09 | 4.51 | down |
| A_23_P337658 | *ALPI* | 1.72E-08 | 4.50 | down |
| A_23_P69810 | *AGPAT9* | 1.48E-08 | 4.50 | down |
| A_23_P122068 | *C1QTNF3* | 6.77E-10 | 4.50 | down |
| A_24_P1054 | *NFKBIL2* | 2.12E-04 | 4.50 | down |
| A_32_P74409 | *AG2* | 1.45E-06 | 4.49 | down |
| A_23_P301304 | *FGFR1* | 3.98E-07 | 4.48 | down |
| A_23_P72697 | *GPIHBP1* | 2.79E-10 | 4.47 | down |
| A_23_P85140 | *TCEAL2* | 5.34E-07 | 4.47 | down |
| A_23_P106405 | *NDN* | 3.08E-05 | 4.46 | down |
| A_23_P70307 | *SMOC2* | 3.62E-03 | 4.45 | down |
| A_23_P214059 | *NR3C1* | 6.14E-08 | 4.45 | down |
| A_32_P218707 |  | 2.70E-06 | 4.45 | down |
| A_23_P84084 | *GFRA2* | 4.06E-10 | 4.43 | down |
| A_24_P561165 |  | 5.15E-10 | 4.42 | down |
| A_23_P331928 | *CD109* | 1.05E-07 | 4.41 | down |
| A_23_P210623 | *PCK1* | 1.53E-06 | 4.39 | down |
| A_23_P372834 | *AQP1* | 1.01E-04 | 4.38 | down |
| A_23_P303242 | *MT1X* | 2.22E-06 | 4.38 | down |
| A_32_P171313 | *GNB4* | 9.03E-09 | 4.36 | down |
| A_23_P110624 | *CTNND2* | 1.86E-07 | 4.34 | down |
| A_24_P596251 |  | 1.12E-03 | 4.33 | down |
| A_24_P13790 | *CES2* | 8.37E-08 | 4.33 | down |
| A_32_P156851 | *RCAN2* | 2.79E-07 | 4.32 | down |
| A_23_P138524 | *CPXM2* | 5.04E-05 | 4.32 | down |
| A_24_P561341 | *FAM150B* | 4.69E-07 | 4.31 | down |
| A_24_P933319 | *RAB3B* | 3.92E-07 | 4.31 | down |
| A_23_P213050 | *HPGD* | 3.55E-07 | 4.31 | down |
| A_23_P69573 | *GUCY1A3* | 4.53E-08 | 4.31 | down |
| A_24_P746314 |  | 1.21E-09 | 4.30 | down |
| A_24_P637651 |  | 1.85E-07 | 4.29 | down |
| A_24_P197537 | *PDE8B* | 1.79E-07 | 4.29 | down |
| A_23_P34644 | *FCGR2B* | 1.74E-07 | 4.29 | down |
| A_23_P168388 | *GIMAP8* | 2.80E-11 | 4.29 | down |
| A_32_P133072 | *SPON1* | 1.79E-03 | 4.28 | down |
| A_23_P401106 | *PDE2A* | 8.53E-10 | 4.28 | down |
| A_23_P45871 | *IFI44L* | 1.23E-04 | 4.28 | down |
| A_23_P138706 | *ADRA2A* | 5.94E-05 | 4.27 | down |
| A_23_P47709 | *FOLR2* | 2.12E-08 | 4.27 | down |
| A_23_P134426 | *GPNMB* | 2.34E-07 | 4.26 | down |
| A_23_P140748 | *NDRG4* | 3.03E-07 | 4.26 | down |
| A_23_P320739 | *MEF2C* | 1.32E-07 | 4.26 | down |
| A_24_P600036 |  | 4.77E-07 | 4.26 | down |
| A_23_P6818 | *SEMA3G* | 2.74E-08 | 4.26 | down |
| A_32_P164477 |  | 2.28E-07 | 4.26 | down |
| A_23_P75260 | *RASSF4* | 7.18E-08 | 4.26 | down |
| A_23_P84791 |  | 7.13E-03 | 4.26 | down |
| A_23_P16225 | *BEST2* | 4.59E-03 | 4.25 | down |
| A_23_P209700 | *NMUR1* | 6.99E-07 | 4.25 | down |
| A_23_P4536 | *EPB41L3* | 1.93E-05 | 4.25 | down |
| A_32_P213521 | *EMCN* | 1.59E-07 | 4.24 | down |
| A_32_P228618 | *RBMS3* | 5.52E-10 | 4.24 | down |
| A_24_P190804 | *AP1S2* | 1.79E-13 | 4.23 | down |
| A_24_P306905 |  | 3.03E-03 | 4.23 | down |
| A_24_P920447 | *C14orf132* | 1.83E-09 | 4.22 | down |
| A_23_P65678 | *FBN1* | 1.04E-09 | 4.22 | down |
| A_23_P201808 | *PPAP2B* | 8.31E-11 | 4.22 | down |
| A_23_P151805 | *FBLN5* | 2.32E-10 | 4.22 | down |
| A_24_P125096 | *MT1X* | 3.32E-06 | 4.22 | down |
| A_23_P417951 | *TSPYL5* | 7.31E-09 | 4.21 | down |
| A_23_P213857 | *C7* | 1.54E-09 | 4.21 | down |
| A_23_P102551 | *MALL* | 1.50E-05 | 4.20 | down |
| A_23_P7752 | *SEMA6A* | 2.12E-05 | 4.20 | down |
| A_24_P214754 | *NR3C1* | 7.76E-07 | 4.18 | down |
| A_32_P48279 |  | 2.38E-09 | 4.18 | down |
| A_32_P74477 |  | 1.03E-13 | 4.18 | down |
| A_23_P1331 | *COL13A1* | 2.70E-06 | 4.16 | down |
| A_24_P68908 | *LOC344887* | 1.20E-06 | 4.15 | down |
| A_23_P311895 | *CLIC5* | 1.50E-08 | 4.15 | down |
| A_23_P132845 | *CLCN2* | 1.68E-08 | 4.15 | down |
| A_24_P942648 |  | 6.91E-06 | 4.15 | down |
| A_32_P3214 | *GXYLT2* | 7.32E-05 | 4.14 | down |
| A_23_P216596 | *SVEP1* | 6.76E-10 | 4.14 | down |
| A_23_P215913 | *CLU* | 2.27E-05 | 4.14 | down |
| A_23_P97606 | *GSTM5* | 1.24E-07 | 4.13 | down |
| A_23_P89431 | *CCL2* | 9.29E-06 | 4.13 | down |
| A_23_P104804 | *ZBTB16* | 2.26E-06 | 4.12 | down |
| A_23_P206707 | *MT1G* | 8.57E-05 | 4.12 | down |
| A_23_P214897 | *AKAP12* | 3.29E-07 | 4.12 | down |
| A_23_P41487 | *TBC1D9* | 4.82E-07 | 4.11 | down |
| A_24_P96505 | *GFRA2* | 1.75E-09 | 4.10 | down |
| A_23_P31177 | *TMEM140* | 8.38E-08 | 4.10 | down |
| A_23_P144827 | *FBXL7* | 4.86E-10 | 4.10 | down |
| A_23_P167096 | *VEGFC* | 7.23E-07 | 4.09 | down |
| A_32_P2452 | *TMTC1* | 5.45E-10 | 4.08 | down |
| A_23_P201747 | *PADI2* | 2.40E-04 | 4.07 | down |
| A_23_P4069 | *TMEM220* | 2.71E-04 | 4.07 | down |
| A_24_P334208 |  | 3.49E-03 | 4.06 | down |
| A_24_P272993 | *MEG3* | 1.59E-04 | 4.06 | down |
| A_24_P919850 | *BDKRB1* | 8.42E-08 | 4.05 | down |
| A_24_P246361 | *IQGAP2* | 4.52E-05 | 4.05 | down |
| A_23_P171074 | *ITM2A* | 2.65E-05 | 4.05 | down |
| A_23_P117782 | *LARP6* | 4.41E-05 | 4.05 | down |
| A_23_P63209 | *HSD11B1* | 3.12E-05 | 4.05 | down |
| A_23_P12746 | *MRC1L1* | 1.79E-07 | 4.05 | down |
| A_23_P19624 | *BMP6* | 2.18E-06 | 4.05 | down |
| A_23_P205177 | *F10* | 7.87E-05 | 4.04 | down |
| A_24_P578437 |  | 1.42E-04 | 4.04 | down |
| A_24_P925505 | *CD36* | 1.71E-08 | 4.03 | down |
| A_23_P408094 | *MXD1* | 5.13E-07 | 4.03 | down |
| A_24_P412156 | *CXCL12* | 1.35E-06 | 4.03 | down |
| A_23_P161727 | *HSPB2* | 3.74E-09 | 4.02 | down |
| A_32_P157927 |  | 8.41E-03 | 4.02 | down |
| A_23_P139123 | *SERPING1* | 3.59E-09 | 4.02 | down |
| A_23_P150609 | *IGF2* | 9.70E-03 | 4.02 | down |
| A_23_P131899 | *SDCBP2* | 2.01E-06 | 4.02 | down |
| A_23_P35977 | *PDZD3* | 6.16E-06 | 4.02 | down |
| A_23_P34597 | *CDA* | 3.08E-05 | 4.02 | down |
| A_23_P151529 | *C14orf132* | 1.79E-09 | 4.00 | down |
| A_23_P46936 | *EGR2* | 1.20E-06 | 4.00 | down |
| A_24_P419300 | *LOC25845* | 1.33E-04 | 4.00 | down |
| A_24_P355246 | *PCSK5* | 1.20E-10 | 4.00 | down |
| A_32_P202502 | *EFHA2* | 3.83E-10 | 3.99 | down |
| A_24_P940426 | *QKI* | 8.46E-10 | 3.99 | down |
| A_23_P206724 | *MT1E* | 2.55E-04 | 3.99 | down |
| A_23_P157136 | *SCIN* | 3.66E-05 | 3.99 | down |
| A_23_P399078 | *TIMP3* | 2.79E-08 | 3.99 | down |
| A_23_P208991 | *PALM* | 1.47E-09 | 3.98 | down |
| A_23_P120125 | *COLEC11* | 2.26E-06 | 3.98 | down |
| A_24_P117782 | *SCRT2* | 4.71E-03 | 3.97 | down |
| A_23_P87379 | *PDE2A* | 1.95E-09 | 3.96 | down |
| A_23_P422933 | *ARHGAP20* | 2.54E-05 | 3.96 | down |
| A_23_P120902 | *LGALS2* | 7.04E-03 | 3.96 | down |
| A_23_P114185 | *TSPAN7* | 7.24E-06 | 3.95 | down |
| A_32_P65022 |  | 4.87E-03 | 3.95 | down |
| A_24_P257224 | *TPO* | 1.60E-03 | 3.95 | down |
| A_32_P230736 | *LOC389033* | 4.41E-06 | 3.94 | down |
| A_23_P396328 | *MPDZ* | 6.59E-10 | 3.93 | down |
| A_23_P35414 | *PPP1R3C* | 5.04E-06 | 3.93 | down |
| A_24_P303874 | *C9orf62* | 8.77E-03 | 3.92 | down |
| A_32_P123527 |  | 2.67E-11 | 3.92 | down |
| A_23_P397376 | *MAF* | 2.24E-11 | 3.92 | down |
| A_23_P394395 | *JPH2* | 3.15E-12 | 3.91 | down |
| A_32_P30075 |  | 2.53E-03 | 3.91 | down |
| A_23_P44155 | *CD96* | 5.63E-07 | 3.90 | down |
| A_23_P87879 | *CD69* | 2.53E-08 | 3.90 | down |
| A_24_P935986 | *BCAT1* | 1.06E-06 | 3.89 | down |
| A_23_P112452 | *GGTA1* | 8.11E-09 | 3.89 | down |
| A_23_P203920 | *SSPN* | 2.60E-07 | 3.89 | down |
| A_24_P851254 |  | 1.58E-03 | 3.88 | down |
| A_23_P42969 | *FGL2* | 2.16E-09 | 3.88 | down |
| A_23_P86079 | *hCG_20426* | 2.33E-10 | 3.87 | down |
| A_23_P256205 | *ABLIM3* | 1.49E-09 | 3.87 | down |
| A_23_P35995 | *ASAM* | 5.87E-07 | 3.86 | down |
| A_24_P330303 | *FRMD6* | 7.18E-14 | 3.85 | down |
| A_24_P48723 | *PTGIS* | 1.86E-06 | 3.85 | down |
| A_23_P93122 | *MEP1A* | 1.38E-04 | 3.84 | down |
| A_23_P150018 | *DUSP5* | 2.50E-07 | 3.84 | down |
| A_23_P434212 | *SULT1A1* | 5.86E-07 | 3.83 | down |
| A_23_P19894 | *AQP1* | 3.25E-04 | 3.83 | down |
| A_32_P52785 | *DAAM2* | 2.27E-11 | 3.82 | down |
| A_23_P154115 | *IGFBP5* | 8.53E-06 | 3.82 | down |
| A_32_P188143 |  | 4.08E-03 | 3.81 | down |
| A_23_P96590 | *GPRASP1* | 3.34E-08 | 3.81 | down |
| A_24_P15550 |  | 4.38E-03 | 3.79 | down |
| A_23_P209564 | *CYBRD1* | 4.02E-09 | 3.79 | down |
| A_23_P144668 | *CMBL* | 6.10E-03 | 3.78 | down |
| A_24_P321525 | *RERG* | 1.90E-15 | 3.78 | down |
| A_23_P85008 | *MAOB* | 5.36E-07 | 3.78 | down |
| A_23_P163782 | *MT1H* | 4.90E-05 | 3.78 | down |
| A_23_P258088 | *PACSIN1* | 7.98E-07 | 3.78 | down |
| A_23_P210425 | *MYL9* | 5.00E-09 | 3.78 | down |
| A_23_P100711 | *PMP22* | 2.91E-09 | 3.78 | down |
| A_32_P132317 | *GPR155* | 3.08E-08 | 3.78 | down |
| A_24_P132383 | *GIMAP8* | 6.77E-10 | 3.77 | down |
| A_23_P205746 | *EML1* | 8.42E-08 | 3.77 | down |
| A_23_P61042 | *IGHA2* | 7.04E-03 | 3.77 | down |
| A_23_P99063 | *LUM* | 1.83E-06 | 3.77 | down |
| A_23_P2492 | *C1S* | 2.62E-08 | 3.76 | down |
| A_24_P484904 |  | 7.34E-03 | 3.76 | down |
| A_23_P205370 | *ASB2* | 8.33E-07 | 3.76 | down |
| A_32_P118942 |  | 7.18E-05 | 3.76 | down |
| A_23_P201940 | *LMOD1* | 1.09E-12 | 3.76 | down |
| A_23_P325690 | *ANKRD35* | 9.21E-10 | 3.75 | down |
| A_23_P21548 | *ARHGAP10* | 1.80E-07 | 3.75 | down |
| A_23_P128744 | *BDKRB1* | 2.85E-07 | 3.75 | down |
| A_23_P50815 | *TTYH1* | 8.80E-09 | 3.74 | down |
| A_23_P202881 | *FEZ1* | 1.71E-08 | 3.74 | down |
| A_24_P358321 |  | 3.30E-03 | 3.73 | down |
| A_23_P252413 | *MT2A* | 1.34E-06 | 3.73 | down |
| A_23_P76480 |  | 2.69E-08 | 3.72 | down |
| A_23_P111724 | *RUNDC3B* | 7.76E-07 | 3.72 | down |
| A_23_P103765 | *FCER1A* | 9.41E-05 | 3.72 | down |
| A_23_P401084 | *ZNF575* | 2.57E-08 | 3.72 | down |
| A_23_P106844 | *MT2A* | 1.12E-05 | 3.72 | down |
| A_23_P372848 | *P2RX1* | 1.22E-06 | 3.72 | down |
| A_23_P86931 | *SLC37A2* | 4.09E-06 | 3.71 | down |
| A_23_P163251 | *PAQR5* | 2.22E-06 | 3.71 | down |
| A_32_P722809 |  | 5.20E-03 | 3.70 | down |
| A_23_P114947 | *RGS2* | 6.25E-07 | 3.70 | down |
| A_23_P203023 | *RDX* | 8.63E-06 | 3.69 | down |
| A_23_P73787 | *NUDT10* | 1.33E-06 | 3.69 | down |
| A_23_P33723 | *CD163* | 4.85E-08 | 3.69 | down |
| A_23_P10980 | *LPHN3* | 4.82E-06 | 3.68 | down |
| A_23_P3312 | *ISLR* | 1.18E-07 | 3.68 | down |
| A_24_P828125 |  | 7.02E-03 | 3.68 | down |
| A_23_P329353 | *CNRIP1* | 1.61E-09 | 3.66 | down |
| A_32_P16204 | *LOC375295* | 5.97E-08 | 3.64 | down |
| A_24_P101282 |  | 2.83E-04 | 3.64 | down |
| A_24_P225845 |  | 3.55E-09 | 3.63 | down |
| A_32_P48536 |  | 2.76E-06 | 3.63 | down |
| A_23_P431410 | *RBMS1* | 2.81E-07 | 3.62 | down |
| A_23_P119006 |  | 6.22E-09 | 3.62 | down |
| A_23_P65278 | *NBEA* | 1.06E-06 | 3.62 | down |
| A_23_P112957 |  | 3.11E-03 | 3.62 | down |
| A_24_P541919 | *DENND5B* | 3.31E-07 | 3.62 | down |
| A_23_P15174 | *MT1F* | 6.78E-04 | 3.61 | down |
| A_23_P98844 | *GEFT* | 1.90E-08 | 3.61 | down |
| A_23_P110403 | *PDLIM3* | 6.66E-07 | 3.61 | down |
| A_24_P143171 | *TMEM47* | 8.16E-06 | 3.60 | down |
| A_24_P31275 | *ATP1B2* | 2.56E-08 | 3.60 | down |
| A_23_P49546 | *GRIN2C* | 2.72E-07 | 3.59 | down |
| A_23_P84154 | *ARHGAP15* | 5.55E-07 | 3.59 | down |
| A_24_P209455 | *GIMAP4* | 3.69E-08 | 3.58 | down |
| A_24_P243749 | *PDK4* | 3.03E-06 | 3.58 | down |
| A_23_P415021 | *METTL7A* | 6.33E-08 | 3.58 | down |
| A_23_P44724 | *CSRP2* | 1.24E-07 | 3.58 | down |
| A_24_P944253 | *KLHL6* | 8.50E-06 | 3.57 | down |
| A_23_P32500 | *STAB1* | 1.71E-08 | 3.57 | down |
| A_23_P145054 | *FAM162B* | 1.76E-06 | 3.56 | down |
| A_24_P289665 | *LOC389332* | 5.05E-04 | 3.56 | down |
| A_23_P372234 | *CA12* | 3.56E-05 | 3.56 | down |
| A_23_P364625 | *LRRC19* | 1.38E-07 | 3.55 | down |
| A_32_P194423 |  | 2.41E-04 | 3.55 | down |
| A_32_P227870 | *SLC30A4* | 2.64E-09 | 3.55 | down |
| A_32_P50066 | *MAP9* | 3.86E-05 | 3.55 | down |
| A_23_P398460 | *HK2* | 7.14E-06 | 3.54 | down |
| A_23_P157879 | *FCN1* | 3.25E-04 | 3.54 | down |
| A_23_P74088 | *MMP23B* | 5.89E-09 | 3.53 | down |
| A_23_P25187 | *KRT81* | 6.05E-03 | 3.52 | down |
| A_24_P187970 | *PADI2* | 1.07E-04 | 3.52 | down |
| A_32_P110390 | *TMEM171* | 6.39E-07 | 3.52 | down |
| A_23_P217326 | *FHL1* | 5.13E-08 | 3.52 | down |
| A_23_P206585 | *PRKCB* | 1.54E-05 | 3.51 | down |
| A_23_P202683 | *CDHR5* | 2.20E-06 | 3.51 | down |
| A_24_P72518 | *AHCYL2* | 7.66E-07 | 3.50 | down |
| A_32_P167631 |  | 2.78E-03 | 3.50 | down |
| A_23_P42746 | *NCF1* | 1.08E-06 | 3.50 | down |
| A_23_P154037 | *AOX1* | 8.38E-08 | 3.49 | down |
| A_23_P20713 | *C8G* | 8.31E-08 | 3.49 | down |
| A_23_P52336 | *UNC5B* | 1.22E-05 | 3.49 | down |
| A_23_P432947 | *GREM1* | 2.38E-04 | 3.49 | down |
| A_24_P938284 | *FCGR2B* | 3.08E-08 | 3.48 | down |
| A_23_P76488 | *EMP1* | 2.84E-06 | 3.48 | down |
| A_24_P220485 | *OLFML2A* | 8.53E-06 | 3.48 | down |
| A_23_P55682 | *ZSCAN18* | 1.34E-03 | 3.48 | down |
| A_24_P928052 | *NRP1* | 4.89E-08 | 3.48 | down |
| A_32_P100379 | *PDGFRA* | 7.76E-07 | 3.48 | down |
| A_24_P215765 | *ATP10A* | 1.15E-08 | 3.48 | down |
| A_23_P118025 | *DPEP2* | 1.65E-08 | 3.48 | down |
| A_24_P646168 |  | 3.58E-10 | 3.48 | down |
| A_32_P220798 | *CD34* | 1.42E-05 | 3.47 | down |
| A_23_P344531 | *SYNPO* | 8.52E-09 | 3.47 | down |
| A_23_P104741 | *KIRREL3* | 1.85E-07 | 3.47 | down |
| A_24_P116700 | *TMEM220* | 1.40E-04 | 3.47 | down |
| A_23_P116898 | *A2M* | 7.69E-07 | 3.47 | down |
| A_24_P222655 | *C1QA* | 8.11E-09 | 3.46 | down |
| A_24_P139901 | *GYPC* | 2.77E-07 | 3.46 | down |
| A_23_P361654 |  | 4.79E-03 | 3.45 | down |
| A_23_P347070 | *PAG1* | 1.21E-09 | 3.45 | down |
| A_23_P139786 | *OASL* | 7.86E-05 | 3.45 | down |
| A_23_P76136 | *TSPAN11* | 9.38E-07 | 3.44 | down |
| A_23_P102113 | *WNT10A* | 5.18E-06 | 3.44 | down |
| A_23_P19142 | *KCNMB1* | 3.75E-08 | 3.44 | down |
| A_23_P146554 | *PTGDS* | 8.52E-04 | 3.43 | down |
| A_23_P98910 | *LRMP* | 5.12E-06 | 3.43 | down |
| A_23_P159163 |  | 5.12E-03 | 3.43 | down |
| A_32_P199998 | *C10orf75* | 7.83E-04 | 3.42 | down |
| A_24_P32935 | *FOLR2* | 8.23E-09 | 3.42 | down |
| A_32_P4018 | *ROR1* | 5.90E-07 | 3.42 | down |
| A_23_P29124 | *38596* | 3.79E-09 | 3.41 | down |
| A_24_P40551 | *BEX4* | 1.93E-04 | 3.41 | down |
| A_24_P409013 | *WDR78* | 1.73E-06 | 3.41 | down |
| A_23_P37702 | *TPSAB1* | 1.09E-03 | 3.40 | down |
| A_23_P19723 | *BMP5* | 4.41E-06 | 3.40 | down |
| A_32_P175934 | *CD48* | 3.07E-06 | 3.40 | down |
| A_23_P24433 | *CTSF* | 1.63E-06 | 3.40 | down |
| A_23_P75769 | *MS4A4A* | 4.75E-09 | 3.39 | down |
| A_23_P335452 | *ZCCHC24* | 2.89E-07 | 3.39 | down |
| A_24_P346859 |  | 3.26E-06 | 3.39 | down |
| A_32_P164203 |  | 3.89E-03 | 3.38 | down |
| A_24_P353638 | *SLAMF7* | 1.37E-04 | 3.38 | down |
| A_23_P252541 | *RAB7B* | 4.67E-09 | 3.38 | down |
| A_23_P361014 | *TSHZ3* | 7.49E-06 | 3.37 | down |
| A_23_P165657 | *SLC20A1* | 8.10E-08 | 3.37 | down |
| A_32_P72611 |  | 4.75E-08 | 3.37 | down |
| A_23_P206806 | *ITGAL* | 1.41E-05 | 3.36 | down |
| A_24_P230916 | *MIER1* | 4.54E-07 | 3.36 | down |
| A_23_P131676 | *CXCR7* | 1.72E-06 | 3.36 | down |
| A_23_P94380 | *AIF1L* | 8.97E-06 | 3.36 | down |
| A_24_P63019 | *IL1R2* | 6.40E-04 | 3.35 | down |
| A_32_P75867 |  | 5.59E-05 | 3.35 | down |
| A_23_P343865 | *FLJ32063* | 2.56E-04 | 3.35 | down |
| A_24_P100996 | *ANO5* | 5.29E-03 | 3.34 | down |
| A_23_P386942 | *DIRAS1* | 3.88E-05 | 3.34 | down |
| A_24_P352388 | *CDHR5* | 7.37E-06 | 3.34 | down |
| A_24_P935103 | *ADCY9* | 1.20E-10 | 3.34 | down |
| A_23_P218369 | *CCL14* | 4.81E-08 | 3.33 | down |
| A_32_P115749 |  | 8.08E-05 | 3.32 | down |
| A_32_P108254 | *FAM20A* | 5.35E-08 | 3.32 | down |
| A_32_P101313 | *PTPLAD2* | 5.08E-08 | 3.32 | down |
| A_24_P296808 | *PNMAL1* | 1.23E-04 | 3.32 | down |
| A_23_P74145 | *CD48* | 1.34E-05 | 3.32 | down |
| A_24_P153568 | *MPEG1* | 5.67E-10 | 3.32 | down |
| A_23_P61057 | *IL16* | 2.65E-05 | 3.31 | down |
| A_23_P21976 | *CSPG4* | 3.99E-06 | 3.31 | down |
| A_23_P92042 | *ITPR1* | 1.52E-08 | 3.31 | down |
| A_24_P361896 | *MT2A* | 3.44E-06 | 3.31 | down |
| A_23_P374695 | *TEK* | 2.88E-06 | 3.31 | down |
| A_24_P274831 | *GIMAP7* | 4.43E-05 | 3.30 | down |
| A_23_P66635 | *CCL11* | 5.70E-03 | 3.30 | down |
| A_23_P254626 | *SGCE* | 1.11E-08 | 3.30 | down |
| A_23_P156218 | *GZMK* | 9.10E-04 | 3.30 | down |
| A_23_P89589 | *PER1* | 1.02E-06 | 3.30 | down |
| A_24_P393740 | *FYB* | 3.25E-07 | 3.29 | down |
| A_24_P240166 | *PHLDB2* | 4.28E-07 | 3.29 | down |
| A_23_P64058 | *RASGRP2* | 8.36E-06 | 3.28 | down |
| A_24_P852756 | *HLA-DQA2* | 5.69E-03 | 3.28 | down |
| A_24_P204574 |  | 7.86E-03 | 3.28 | down |
| A_24_P381604 | *ITM2B* | 7.92E-03 | 3.28 | down |
| A_23_P105461 | *CMKLR1* | 1.24E-07 | 3.27 | down |
| A_23_P110791 | *CSF1R* | 3.24E-09 | 3.27 | down |
| A_23_P91334 | *HSPA12B* | 2.29E-06 | 3.27 | down |
| A_32_P215938 | *GPSM1* | 1.18E-05 | 3.27 | down |
| A_32_P116206 | *RELL1* | 3.58E-11 | 3.26 | down |
| A_23_P96271 | *MYOM1* | 8.52E-06 | 3.26 | down |
| A_23_P97141 | *RGS1* | 1.85E-04 | 3.25 | down |
| A_24_P267664 | *C21orf88* | 3.62E-04 | 3.25 | down |
| A_23_P79398 | *IL1R2* | 8.28E-04 | 3.25 | down |
| A_23_P300600 | *NEFH* | 4.67E-07 | 3.25 | down |
| A_23_P108823 | *OSBPL6* | 3.54E-06 | 3.24 | down |
| A_23_P49816 | *ADAP2* | 6.08E-07 | 3.24 | down |
| A_24_P124349 | *PDGFD* | 2.44E-06 | 3.24 | down |
| A_23_P317620 | *ARL4C* | 3.74E-05 | 3.24 | down |
| A_32_P113887 |  | 9.47E-03 | 3.24 | down |
| A_24_P239076 | *IGLL1* | 5.92E-03 | 3.24 | down |
| A_23_P18017 | *CPA3* | 3.16E-04 | 3.23 | down |
| A_23_P392384 | *AIF1L* | 3.41E-05 | 3.23 | down |
| A_23_P203173 | *IL10RA* | 3.39E-08 | 3.22 | down |
| A_23_P217384 | *AP1S2* | 3.61E-12 | 3.22 | down |
| A_24_P365975 | *COL8A2* | 3.04E-06 | 3.21 | down |
| A_24_P270424 | *DPF3* | 3.49E-05 | 3.21 | down |
| A_32_P50655 |  | 1.45E-05 | 3.21 | down |
| A_23_P122127 | *FYB* | 1.54E-07 | 3.20 | down |
| A_24_P542291 |  | 3.85E-03 | 3.20 | down |
| A_24_P168495 | *CYP4F2* | 2.93E-03 | 3.20 | down |
| A_23_P29939 | *SNCA* | 3.55E-08 | 3.20 | down |
| A_23_P128235 | *KRT1* | 8.03E-04 | 3.19 | down |
| A_23_P157299 | *AEBP1* | 5.19E-06 | 3.19 | down |
| A_23_P105251 | *GLI1* | 2.84E-07 | 3.19 | down |
| A_23_P87853 | *TMCC3* | 1.07E-05 | 3.19 | down |
| A_23_P428887 | *KLHL34* | 2.20E-04 | 3.18 | down |
| A_23_P58251 | *CPZ* | 3.25E-05 | 3.18 | down |
| A_24_P201702 | *CLEC2B* | 2.83E-06 | 3.18 | down |
| A_23_P257649 | *RBP1* | 4.53E-06 | 3.18 | down |
| A_23_P16722 | *DOCK10* | 6.37E-09 | 3.17 | down |
| A_23_P414913 | *GLIPR2* | 2.73E-05 | 3.17 | down |
| A_23_P60856 | *TSPAN4* | 7.66E-09 | 3.17 | down |
| A_23_P217528 | *KLF8* | 6.48E-06 | 3.16 | down |
| A_24_P104115 | *RHOF* | 1.70E-06 | 3.16 | down |
| A_23_P129856 | *HIC1* | 3.63E-09 | 3.16 | down |
| A_23_P125423 | *C1R* | 6.66E-07 | 3.16 | down |
| A_23_P61945 | *MITF* | 1.32E-06 | 3.16 | down |
| A_23_P146339 | *GPT* | 1.51E-03 | 3.15 | down |
| A_24_P295590 | *RASSF4* | 1.09E-07 | 3.15 | down |
| A_32_P42895 |  | 1.76E-05 | 3.15 | down |
| A_23_P79572 | *MGC16025* | 1.50E-03 | 3.15 | down |
| A_32_P132194 |  | 1.90E-03 | 3.15 | down |
| A_23_P152838 | *CCL5* | 3.22E-04 | 3.15 | down |
| A_23_P79562 | *FABP1* | 7.13E-03 | 3.15 | down |
| A_23_P217570 | *CAPN6* | 4.64E-03 | 3.15 | down |
| A_23_P100660 | *SERPINF1* | 1.52E-07 | 3.14 | down |
| A_23_P312920 | *POU2AF1* | 2.75E-03 | 3.14 | down |
| A_23_P141367 | *CCR10* | 5.42E-04 | 3.14 | down |
| A_32_P214860 |  | 4.82E-04 | 3.13 | down |
| A_23_P161190 | *VIM* | 1.16E-07 | 3.13 | down |
| A_23_P153897 | *GNG7* | 2.20E-05 | 3.13 | down |
| A_23_P34144 | *MAGEH1* | 9.25E-07 | 3.13 | down |
| A_23_P10401 | *PPP2R3A* | 1.06E-08 | 3.13 | down |
| A_32_P40288 | *TMEM200A* | 7.96E-03 | 3.13 | down |
| A_23_P159027 | *ZNF521* | 5.70E-08 | 3.13 | down |
| A_23_P119196 | *KLF2* | 1.03E-05 | 3.12 | down |
| A_24_P83102 | *IGLL1* | 5.77E-03 | 3.12 | down |
| A_23_P89871 | *ZNF415* | 8.89E-03 | 3.12 | down |
| A_23_P83857 | *MAOA* | 5.89E-08 | 3.11 | down |
| A_23_P157736 | *PPAPDC3* | 5.55E-07 | 3.11 | down |
| A_23_P415401 | *KLF9* | 9.61E-06 | 3.11 | down |
| A_23_P395438 | *HTRA3* | 2.20E-05 | 3.11 | down |
| A_23_P382065 | *EMCN* | 5.61E-06 | 3.11 | down |
| A_23_P325924 | *FAM59B* | 4.05E-05 | 3.11 | down |
| A_23_P53126 | *LMO2* | 7.98E-09 | 3.09 | down |
| A_32_P162494 |  | 5.91E-03 | 3.09 | down |
| A_24_P912985 |  | 3.37E-08 | 3.09 | down |
| A_23_P102117 | *WNT10A* | 2.00E-06 | 3.09 | down |
| A_24_P330518 | *CA12* | 1.12E-04 | 3.09 | down |
| A_24_P81789 | *RHOF* | 5.94E-05 | 3.09 | down |
| A_23_P72117 | *SMPDL3A* | 1.20E-06 | 3.08 | down |
| A_23_P71480 | *DEFB1* | 1.27E-03 | 3.08 | down |
| A_23_P92903 | *C1QTNF2* | 7.04E-09 | 3.08 | down |
| A_24_P316046 | *LOC25845* | 6.35E-03 | 3.08 | down |
| A_23_P133445 | *GZMA* | 6.13E-05 | 3.08 | down |
| A_32_P80850 | *COL14A1* | 8.80E-09 | 3.08 | down |
| A_23_P28906 |  | 5.84E-06 | 3.07 | down |
| A_32_P169406 | *LOC400043* | 1.12E-13 | 3.07 | down |
| A_23_P111701 | *GNG11* | 2.59E-06 | 3.07 | down |
| A_23_P142974 | *ARHGAP25* | 5.13E-06 | 3.07 | down |
| A_23_P253602 | *BMX* | 1.61E-03 | 3.07 | down |
| A_32_P33913 |  | 9.65E-11 | 3.07 | down |
| A_23_P151426 | *FOXO1* | 6.59E-08 | 3.06 | down |
| A_24_P618928 |  | 3.77E-08 | 3.06 | down |
| A_23_P166207 | *ABCC13* | 5.07E-04 | 3.06 | down |
| A_23_P324340 | *DISP2* | 2.53E-05 | 3.06 | down |
| A_23_P126266 | *HLX* | 3.17E-07 | 3.05 | down |
| A_23_P26890 | *MMP28* | 6.39E-03 | 3.05 | down |
| A_23_P71328 | *MATN2* | 1.91E-03 | 3.05 | down |
| A_23_P391711 | *FABP2* | 7.57E-04 | 3.05 | down |
| A_32_P69136 |  | 1.40E-04 | 3.05 | down |
| A_23_P128084 | *ITGA7* | 1.44E-07 | 3.04 | down |
| A_24_P117620 | *CAMK2N1* | 2.04E-05 | 3.04 | down |
| A_23_P326319 | *C16orf45* | 3.74E-07 | 3.04 | down |
| A_24_P315256 |  | 2.85E-06 | 3.04 | down |
| A_32_P181166 | *C1orf162* | 1.06E-06 | 3.04 | down |
| A_23_P363255 | *CCDC68* | 7.82E-03 | 3.03 | down |
| A_23_P426663 | *MITF* | 2.89E-05 | 3.03 | down |
| A_23_P253145 | *TAGAP* | 4.14E-06 | 3.03 | down |
| A_23_P28334 | *IL18RAP* | 2.75E-06 | 3.03 | down |
| A_23_P109143 | *PRNP* | 8.79E-06 | 3.03 | down |
| A_24_P364591 | *FBLN5* | 2.65E-09 | 3.03 | down |
| A_24_P296772 | *PPP1R14A* | 7.76E-07 | 3.02 | down |
| A_23_P133338 | *CDHR2* | 3.60E-06 | 3.02 | down |
| A_24_P205604 | *PADI2* | 1.16E-03 | 3.02 | down |
| A_32_P4595 | *SGCD* | 6.27E-06 | 3.02 | down |
| A_23_P131089 | *KANK3* | 8.88E-08 | 3.02 | down |
| A_24_P158946 | *FGD4* | 4.23E-07 | 3.02 | down |
| A_23_P73114 | *PROS1* | 3.74E-03 | 3.02 | down |
| A_23_P42588 | *GIMAP5* | 9.95E-07 | 3.01 | down |
| A_23_P22422 | *PNMA3* | 6.94E-04 | 3.01 | down |
| A_32_P116203 | *NCF1* | 1.21E-06 | 3.01 | down |
| A_24_P54390 | *RASGRP3* | 3.30E-06 | 3.00 | down |
| A_23_P20285 | *PDLIM2* | 2.25E-06 | 3.00 | down |
| A_24_P921366 | *CALD1* | 1.59E-06 | 3.00 | down |
| A_23_P155666 | *NAAA* | 2.14E-04 | 3.00 | down |
| A_24_P16541 | *NBPF9* | 2.96E-04 | 3.00 | down |
| A_23_P56868 |  | 6.56E-03 | 3.00 | down |
| A_23_P17269 | *CCDC88A* | 4.57E-05 | 2.99 | down |
| A_23_P301530 | *ANK3* | 7.32E-09 | 2.99 | down |
| A_23_P112241 | *DNAJB5* | 1.18E-06 | 2.99 | down |
| A_24_P104119 | *RHOF* | 5.12E-05 | 2.99 | down |
| A_24_P119141 | *PROS1* | 3.81E-03 | 2.98 | down |
| A_23_P87013 | *TAGLN* | 1.42E-07 | 2.98 | down |
| A_24_P297182 | *GGT5* | 6.64E-06 | 2.98 | down |
| A_24_P921103 |  | 1.17E-05 | 2.98 | down |
| A_24_P388433 | *PPP2R3A* | 1.78E-07 | 2.97 | down |
| A_23_P147641 | *TCEA2* | 6.60E-05 | 2.97 | down |
| A_24_P928017 |  | 1.34E-03 | 2.97 | down |
| A_23_P355244 | *SAMD9* | 1.20E-06 | 2.97 | down |
| A_23_P10902 | *FRZB* | 2.79E-03 | 2.97 | down |
| A_23_P371966 | *FAM171B* | 3.02E-06 | 2.97 | down |
| A_23_P15247 | *C16orf5* | 7.25E-05 | 2.97 | down |
| A_23_P258190 | *AKR1B1* | 4.13E-05 | 2.97 | down |
| A_23_P12549 | *APBB1IP* | 1.54E-04 | 2.96 | down |
| A_23_P252075 | *AHCYL2* | 6.10E-06 | 2.96 | down |
| A_24_P345451 | *CYBRD1* | 7.96E-04 | 2.96 | down |
| A_32_P61684 | *PAG1* | 1.66E-07 | 2.96 | down |
| A_23_P87011 | *TAGLN* | 6.69E-07 | 2.96 | down |
| A_24_P262201 | *SULT1A4* | 1.50E-06 | 2.96 | down |
| A_23_P202269 | *ANK3* | 3.16E-04 | 2.95 | down |
| A_23_P357717 | *TCL1A* | 7.44E-03 | 2.95 | down |
| A_24_P570378 |  | 4.53E-04 | 2.95 | down |
| A_23_P121926 | *SEPP1* | 3.11E-06 | 2.95 | down |
| A_23_P427023 | *GIMAP1* | 2.72E-07 | 2.94 | down |
| A_24_P941773 | *METTL7A* | 4.77E-04 | 2.94 | down |
| A_23_P342138 | *ADAMTSL1* | 2.90E-04 | 2.94 | down |
| A_32_P17484 |  | 1.24E-06 | 2.93 | down |
| A_23_P79587 | *ALPP* | 1.26E-06 | 2.93 | down |
| A_24_P918147 |  | 7.43E-05 | 2.93 | down |
| A_32_P24832 | *OLFML3* | 2.86E-06 | 2.93 | down |
| A_24_P288836 | *HLA-DPB2* | 4.18E-05 | 2.93 | down |
| A_23_P413585 | *FOXD2* | 2.11E-04 | 2.93 | down |
| A_23_P67799 | *TMEM37* | 5.73E-06 | 2.93 | down |
| A_23_P39840 | *VAMP5* | 1.01E-06 | 2.92 | down |
| A_23_P140190 | *KIAA0125* | 6.61E-03 | 2.92 | down |
| A_23_P432573 | *MRGPRF* | 2.70E-06 | 2.92 | down |
| A_24_P324405 | *ANKRD11* | 8.37E-03 | 2.92 | down |
| A_23_P200001 | *NEXN* | 1.65E-05 | 2.92 | down |
| A_23_P82775 | *SOX17* | 4.62E-05 | 2.92 | down |
| A_23_P23279 | *RCSD1* | 3.65E-07 | 2.92 | down |
| A_23_P24414 | *EFEMP2* | 4.58E-07 | 2.92 | down |
| A_32_P207789 |  | 5.43E-05 | 2.92 | down |
| A_23_P21758 | *ADAM28* | 1.40E-06 | 2.92 | down |
| A_24_P110983 |  | 7.07E-07 | 2.92 | down |
| A_23_P383986 | *CHST15* | 1.01E-04 | 2.91 | down |
| A_23_P338981 | *CYGB* | 2.69E-08 | 2.91 | down |
| A_23_P315571 | *RFTN1* | 3.21E-06 | 2.91 | down |
| A_32_P30898 |  | 1.94E-07 | 2.91 | down |
| A_23_P103371 | *ADC* | 2.26E-06 | 2.91 | down |
| A_23_P14174 | *TNFSF13B* | 4.52E-07 | 2.91 | down |
| A_23_P333029 | *C8orf47* | 7.95E-03 | 2.90 | down |
| A_24_P943393 | *AHNAK* | 1.92E-06 | 2.90 | down |
| A_23_P31064 | *MOXD1* | 1.06E-04 | 2.90 | down |
| A_23_P356581 | *ROBO3* | 1.73E-04 | 2.90 | down |
| A_32_P218785 |  | 3.51E-03 | 2.90 | down |
| A_23_P35617 | *PLCE1* | 1.62E-06 | 2.90 | down |
| A_23_P137366 | *C1QB* | 5.46E-06 | 2.90 | down |
| A_23_P340848 | *PTGIR* | 1.49E-05 | 2.90 | down |
| A_23_P118122 | *RGS11* | 4.50E-04 | 2.89 | down |
| A_23_P144807 | *39692* | 5.33E-10 | 2.89 | down |
| A_32_P179646 |  | 8.03E-03 | 2.89 | down |
| A_24_P11462 | *ADC* | 3.80E-08 | 2.89 | down |
| A_23_P8906 | *LRP12* | 4.84E-05 | 2.89 | down |
| A_23_P302005 | *STON1* | 5.02E-05 | 2.89 | down |
| A_24_P913716 | *B3GNT7* | 3.52E-03 | 2.89 | down |
| A_32_P356316 | *HLA-DOA* | 2.14E-06 | 2.88 | down |
| A_23_P116942 | *LAG3* | 1.26E-04 | 2.88 | down |
| A_24_P319088 | *CCL23* | 2.33E-06 | 2.88 | down |
| A_24_P355649 | *FLI1* | 1.42E-06 | 2.88 | down |
| A_23_P102160 | *FAM82A1* | 4.39E-10 | 2.88 | down |
| A_24_P372134 | *TMEM140* | 1.29E-08 | 2.88 | down |
| A_23_P41365 | *SMR3A* | 8.54E-03 | 2.87 | down |
| A_23_P300150 | *NFATC1* | 6.17E-06 | 2.87 | down |
| A_24_P927886 | *GNA11* | 3.76E-06 | 2.87 | down |
| A_23_P145916 | *AEBP1* | 5.29E-05 | 2.87 | down |
| A_23_P43283 | *GPR124* | 2.50E-06 | 2.86 | down |
| A_23_P360804 | *CPNE5* | 4.62E-04 | 2.86 | down |
| A_23_P166248 | *RCAN1* | 7.46E-09 | 2.86 | down |
| A_23_P43977 |  | 4.56E-04 | 2.86 | down |
| A_23_P213620 | *PPP2R2B* | 2.41E-06 | 2.86 | down |
| A_24_P75008 | *LOC346329* | 1.67E-06 | 2.86 | down |
| A_23_P203351 | *MS4A7* | 1.20E-06 | 2.86 | down |
| A_32_P62863 | *SCHIP1* | 4.66E-05 | 2.86 | down |
| A_23_P80739 | *PLCD1* | 4.41E-06 | 2.86 | down |
| A_23_P17345 | *MAFB* | 1.22E-09 | 2.86 | down |
| A_24_P334361 | *DDX60* | 1.47E-07 | 2.85 | down |
| A_24_P340128 | *P2RY8* | 7.54E-04 | 2.85 | down |
| A_23_P24616 | *SIAE* | 1.09E-03 | 2.85 | down |
| A_23_P43276 | *GPR124* | 9.70E-07 | 2.84 | down |
| A_23_P111206 | *FKBP5* | 6.71E-06 | 2.84 | down |
| A_24_P95154 | *TUSC3* | 1.43E-03 | 2.84 | down |
| A_24_P252739 | *KLF6* | 3.25E-03 | 2.84 | down |
| A_23_P140427 | *EVL* | 1.41E-06 | 2.84 | down |
| A_23_P301521 | *KIAA1462* | 5.13E-04 | 2.84 | down |
| A_23_P25974 | *TTC7B* | 1.34E-04 | 2.84 | down |
| A_23_P217228 | *TRO* | 3.79E-07 | 2.83 | down |
| A_23_P348737 | *NR2F1* | 1.61E-03 | 2.83 | down |
| A_23_P412562 | *C1orf162* | 1.05E-07 | 2.83 | down |
| A_32_P104469 |  | 2.92E-03 | 2.83 | down |
| A_24_P391230 | *CYYR1* | 4.62E-06 | 2.83 | down |
| A_24_P74070 | *PARD6G* | 3.86E-06 | 2.83 | down |
| A_24_P236799 | *RAB31* | 5.27E-05 | 2.83 | down |
| A_32_P91273 |  | 6.88E-08 | 2.82 | down |
| A_24_P278603 | *MOGAT2* | 9.14E-05 | 2.82 | down |
| A_23_P50786 | *CLIP3* | 2.26E-06 | 2.82 | down |
| A_23_P92899 | *C1QTNF2* | 8.91E-09 | 2.82 | down |
| A_32_P193218 | *LPP* | 2.88E-04 | 2.81 | down |
| A_23_P161194 | *VIM* | 3.03E-06 | 2.81 | down |
| A_23_P96568 | *FLNA* | 1.92E-06 | 2.81 | down |
| A_23_P213959 | *PPARGC1B* | 4.83E-06 | 2.81 | down |
| A_24_P156113 | *EHD2* | 3.43E-06 | 2.81 | down |
| A_23_P2041 | *MICALCL* | 1.89E-06 | 2.81 | down |
| A_23_P36496 | *RBMS1* | 2.85E-05 | 2.81 | down |
| A_24_P659113 | *CCNYL1* | 8.90E-07 | 2.81 | down |
| A_32_P227930 | *C5orf52* | 1.26E-05 | 2.81 | down |
| A_23_P42575 | *CALD1* | 5.76E-05 | 2.81 | down |
| A_23_P142289 | *GNA11* | 6.18E-06 | 2.80 | down |
| A_23_P387630 | *STARD8* | 9.90E-10 | 2.80 | down |
| A_24_P363583 | *AGFG2* | 8.66E-07 | 2.80 | down |
| A_24_P11315 | *OLFML3* | 1.62E-05 | 2.80 | down |
| A_23_P85015 | *MAOB* | 3.76E-06 | 2.80 | down |
| A_23_P120644 |  | 1.36E-03 | 2.80 | down |
| A_23_P253345 | *C8orf4* | 1.47E-04 | 2.80 | down |
| A_32_P162524 |  | 4.48E-04 | 2.80 | down |
| A_23_P27332 | *TCF4* | 1.08E-05 | 2.80 | down |
| A_23_P59637 | *DOCK4* | 1.42E-06 | 2.80 | down |
| A_23_P206212 | *THBS1* | 3.26E-05 | 2.80 | down |
| A_32_P154361 |  | 9.19E-04 | 2.80 | down |
| A_23_P67661 | *COX7A1* | 4.21E-06 | 2.80 | down |
| A_32_P7721 | *RORA* | 4.81E-06 | 2.79 | down |
| A_23_P340019 | *NLRC3* | 1.70E-04 | 2.79 | down |
| A_23_P431305 | *FAM69B* | 3.33E-06 | 2.79 | down |
| A_23_P333640 | *PAPLN* | 1.21E-04 | 2.79 | down |
| A_24_P372223 | *MSR1* | 7.36E-06 | 2.79 | down |
| A_24_P361816 |  | 9.02E-03 | 2.79 | down |
| A_23_P203882 | *MMP19* | 3.25E-05 | 2.79 | down |
| A_32_P38637 | *KRBA1* | 6.65E-05 | 2.79 | down |
| A_24_P373152 | *CFL2* | 9.96E-06 | 2.78 | down |
| A_24_P766716 | *CMKLR1* | 4.15E-07 | 2.78 | down |
| A_23_P156826 | *C6orf105* | 3.72E-03 | 2.78 | down |
| A_23_P74575 | *CD1D* | 3.17E-04 | 2.78 | down |
| A_24_P346269 |  | 3.08E-03 | 2.78 | down |
| A_23_P57570 | *A4GALT* | 2.05E-07 | 2.78 | down |
| A_24_P118196 | *GXYLT2* | 9.48E-04 | 2.78 | down |
| A_32_P92840 |  | 5.49E-08 | 2.78 | down |
| A_23_P150053 | *ACTA2* | 5.03E-07 | 2.77 | down |
| A_23_P19663 | *CTGF* | 4.06E-06 | 2.77 | down |
| A_24_P135322 | *NRP1* | 1.21E-06 | 2.77 | down |
| A_23_P140876 | *ABCA3* | 1.65E-04 | 2.77 | down |
| A_23_P103601 | *MAN1C1* | 1.20E-06 | 2.77 | down |
| A_24_P400997 | *SMCHD1* | 4.22E-05 | 2.77 | down |
| A_23_P401700 | *APBB1IP* | 4.29E-05 | 2.77 | down |
| A_23_P251499 | *PCOLCE* | 1.78E-07 | 2.76 | down |
| A_23_P421401 | *PDGFRB* | 1.32E-05 | 2.76 | down |
| A_24_P940115 | *DLC1* | 8.39E-07 | 2.76 | down |
| A_23_P300033 | *PDGFRA* | 2.26E-06 | 2.76 | down |
| A_32_P169550 |  | 5.46E-06 | 2.76 | down |
| A_23_P169039 | *SNAI2* | 1.62E-05 | 2.76 | down |
| A_32_P22750 | *LOC100293875* | 1.97E-04 | 2.75 | down |
| A_24_P77968 | *FLNC* | 3.13E-05 | 2.75 | down |
| A_24_P22079 | *FOXO1* | 2.75E-09 | 2.75 | down |
| A_23_P103496 | *GBP4* | 5.93E-03 | 2.75 | down |
| A_23_P78092 | *EVI2A* | 1.16E-05 | 2.75 | down |
| A_32_P69956 |  | 7.22E-05 | 2.74 | down |
| A_32_P122715 |  | 2.51E-08 | 2.74 | down |
| A_23_P65022 | *ACADS* | 5.05E-05 | 2.74 | down |
| A_23_P70060 | *PPAP2A* | 7.21E-08 | 2.74 | down |
| A_23_P40415 | *ADAMTS5* | 4.72E-05 | 2.74 | down |
| A_23_P86171 | *FOXD2* | 6.23E-04 | 2.74 | down |
| A_23_P365685 | *LIMS3* | 6.29E-06 | 2.74 | down |
| A_32_P95894 |  | 3.84E-04 | 2.74 | down |
| A_23_P37317 |  | 1.60E-07 | 2.73 | down |
| A_23_P106675 | *PLCG2* | 5.14E-05 | 2.73 | down |
| A_24_P721699 | *NCRNA00181* | 2.61E-06 | 2.72 | down |
| A_24_P326511 | *SORBS1* | 3.61E-06 | 2.72 | down |
| A_24_P185854 | *DMD* | 1.61E-03 | 2.72 | down |
| A_24_P923271 |  | 6.31E-03 | 2.72 | down |
| A_24_P305933 | *TMCC3* | 8.32E-07 | 2.72 | down |
| A_32_P109495 |  | 1.43E-06 | 2.72 | down |
| A_23_P368805 | *HHLA2* | 1.20E-03 | 2.72 | down |
| A_23_P323761 | *TRAF3IP3* | 1.74E-04 | 2.71 | down |
| A_23_P411723 | *PLAG1* | 1.47E-04 | 2.71 | down |
| A_32_P216004 |  | 1.21E-04 | 2.71 | down |
| A_23_P352266 | *BCL2* | 4.91E-05 | 2.71 | down |
| A_24_P10731 | *MADCAM1* | 7.32E-04 | 2.71 | down |
| A_23_P134237 | *RARRES2* | 1.34E-03 | 2.71 | down |
| A_23_P151297 | *TENC1* | 3.34E-09 | 2.70 | down |
| A_24_P246626 |  | 1.04E-05 | 2.70 | down |
| A_24_P928522 | *DST* | 1.36E-03 | 2.70 | down |
| A_23_P32444 | *MXRA8* | 2.65E-06 | 2.70 | down |
| A_24_P937240 |  | 3.49E-06 | 2.70 | down |
| A_32_P163306 | *HHIPL1* | 1.70E-06 | 2.70 | down |
| A_23_P434430 | *ZNF439* | 8.42E-05 | 2.70 | down |
| A_23_P110253 | *KIT* | 7.41E-04 | 2.70 | down |
| A_24_P610945 |  | 2.49E-04 | 2.69 | down |
| A_23_P39766 | *GLS* | 7.34E-06 | 2.69 | down |
| A_23_P145024 | *ADRB2* | 9.09E-05 | 2.69 | down |
| A_23_P141376 | *PTRF* | 1.78E-06 | 2.69 | down |
| A_32_P162520 |  | 8.43E-03 | 2.69 | down |
| A_32_P74942 |  | 4.37E-03 | 2.69 | down |
| A_23_P149153 | *PDE4DIP* | 4.80E-05 | 2.68 | down |
| A_23_P315273 | *MT3* | 2.71E-03 | 2.68 | down |
| A_24_P931428 | *TCF4* | 3.16E-05 | 2.68 | down |
| A_23_P217009 | *C9orf24* | 7.41E-04 | 2.68 | down |
| A_23_P253982 | *HOXA4* | 5.74E-06 | 2.68 | down |
| A_32_P222684 | *PRDM6* | 3.54E-07 | 2.67 | down |
| A_32_P143824 |  | 5.06E-07 | 2.67 | down |
| A_23_P123086 | *KIAA1908* | 5.10E-05 | 2.67 | down |
| A_23_P137751 | *FAM46C* | 3.55E-04 | 2.67 | down |
| A_24_P415012 | *LRP12* | 4.87E-04 | 2.67 | down |
| A_23_P46045 | *RGS5* | 9.41E-05 | 2.67 | down |
| A_23_P54840 | *MT1A* | 3.54E-05 | 2.67 | down |
| A_24_P100387 | *GK* | 8.33E-06 | 2.67 | down |
| A_23_P90634 | *CCNYL1* | 8.42E-07 | 2.67 | down |
| A_23_P41470 | *DDX60* | 1.69E-06 | 2.67 | down |
| A_24_P170136 |  | 7.97E-05 | 2.67 | down |
| A_24_P90005 | *COL13A1* | 1.88E-04 | 2.67 | down |
| A_32_P185229 | *ATP8B2* | 9.39E-06 | 2.66 | down |
| A_23_P203488 | *SMPD1* | 6.05E-05 | 2.66 | down |
| A_24_P397294 | *LTC4S* | 5.46E-08 | 2.66 | down |
| A_24_P262127 | *RRAD* | 9.17E-04 | 2.66 | down |
| A_23_P203376 | *MS4A6A* | 2.31E-06 | 2.66 | down |
| A_24_P305345 | *CD209* | 1.09E-06 | 2.66 | down |
| A_24_P159434 | *CD300A* | 7.04E-03 | 2.65 | down |
| A_23_P129925 | *SLFN11* | 9.38E-05 | 2.65 | down |
| A_23_P86653 | *SRGN* | 7.84E-06 | 2.65 | down |
| A_23_P88767 | *PLA2G10* | 4.00E-04 | 2.65 | down |
| A_24_P379750 | *MXD1* | 1.34E-05 | 2.65 | down |
| A_32_P217140 | *ISX* | 3.53E-04 | 2.65 | down |
| A_23_P57323 | *ERG* | 1.75E-04 | 2.65 | down |
| A_23_P33791 | *SSBP2* | 9.89E-06 | 2.65 | down |
| A_23_P65388 | *CLEC14A* | 2.02E-06 | 2.65 | down |
| A_23_P135990 | *SLCO2A1* | 6.15E-05 | 2.65 | down |
| A_23_P170713 |  | 6.14E-05 | 2.65 | down |
| A_23_P65629 | *KCNK10* | 5.70E-03 | 2.64 | down |
| A_24_P944154 | *MCTP2* | 2.04E-03 | 2.64 | down |
| A_23_P414654 | *RAB37* | 1.86E-04 | 2.64 | down |
| A_23_P411246 | *TMC8* | 2.28E-05 | 2.64 | down |
| A_23_P57036 | *CD40* | 1.26E-04 | 2.64 | down |
| A_23_P14986 | *HSD11B2* | 2.52E-06 | 2.64 | down |
| A_23_P138125 | *FAIM3* | 1.61E-04 | 2.64 | down |
| A_32_P59302 | *HIVEP3* | 4.24E-04 | 2.64 | down |
| A_32_P144421 | *ZNF518B* | 2.75E-03 | 2.63 | down |
| A_23_P302914 | *ZFYVE28* | 3.17E-06 | 2.63 | down |
| A_23_P209944 | *RETSAT* | 2.57E-06 | 2.63 | down |
| A_23_P127789 | *AHNAK* | 4.32E-04 | 2.63 | down |
| A_23_P161837 | *MRVI1* | 5.11E-04 | 2.63 | down |
| A_24_P51115 | *MFSD4* | 7.30E-03 | 2.63 | down |
| A_32_P159651 | *KAT2B* | 9.53E-07 | 2.63 | down |
| A_23_P95619 | *GLOD5* | 3.29E-07 | 2.63 | down |
| A_23_P155057 | *CYTH4* | 6.40E-05 | 2.63 | down |
| A_23_P334414 | *TRAF3IP3* | 6.05E-04 | 2.63 | down |
| A_32_P120484 |  | 1.62E-05 | 2.62 | down |
| A_23_P122724 | *VNN2* | 4.75E-05 | 2.62 | down |
| A_23_P210100 | *CYP26B1* | 6.27E-03 | 2.62 | down |
| A_24_P192914 | *AMICA1* | 2.63E-06 | 2.62 | down |
| A_23_P99141 | *GPR162* | 8.68E-08 | 2.62 | down |
| A_32_P70519 | *LPP* | 5.02E-04 | 2.61 | down |
| A_32_P140501 |  | 7.44E-07 | 2.61 | down |
| A_23_P129695 | *VASN* | 2.33E-05 | 2.61 | down |
| A_23_P404606 | *C5orf41* | 7.45E-04 | 2.61 | down |
| A_23_P26865 | *MYH3* | 4.32E-04 | 2.61 | down |
| A_24_P77432 | *ROBO1* | 5.50E-04 | 2.61 | down |
| A_24_P45476 | *XCL1* | 2.25E-03 | 2.61 | down |
| A_24_P497244 | *MALAT1* | 8.79E-03 | 2.61 | down |
| A_23_P259621 | *LAT2* | 1.55E-07 | 2.60 | down |
| A_23_P130974 | *KIAA1683* | 1.98E-04 | 2.60 | down |
| A_24_P200854 | *HOXA2* | 4.13E-05 | 2.60 | down |
| A_23_P10442 | *OSBPL1A* | 3.26E-05 | 2.60 | down |
| A_23_P150064 | *MMRN2* | 2.47E-07 | 2.59 | down |
| A_23_P143331 | *BMP2* | 1.52E-05 | 2.59 | down |
| A_23_P99747 | *CDKL1* | 1.13E-07 | 2.59 | down |
| A_23_P122443 | *HIST1H1C* | 1.11E-05 | 2.59 | down |
| A_23_P500886 | *CLDN15* | 9.57E-03 | 2.59 | down |
| A_23_P24774 | *ABCC8* | 5.93E-05 | 2.59 | down |
| A_32_P215676 |  | 5.36E-05 | 2.58 | down |
| A_23_P405175 | *CNST* | 2.30E-06 | 2.58 | down |
| A_23_P392470 | *NR3C2* | 3.98E-06 | 2.58 | down |
| A_24_P227971 | *TP53TG3* | 5.16E-03 | 2.58 | down |
| A_24_P136484 |  | 5.87E-04 | 2.58 | down |
| A_23_P127727 | *MPEG1* | 2.97E-08 | 2.58 | down |
| A_23_P416711 | *ST6GALNAC3* | 7.77E-05 | 2.58 | down |
| A_23_P55179 | *ARL4D* | 3.10E-06 | 2.58 | down |
| A_23_P252721 | *DLC1* | 2.09E-05 | 2.58 | down |
| A_23_P122852 | *SMARCD3* | 7.74E-08 | 2.57 | down |
| A_23_P258151 | *FGD5* | 3.76E-06 | 2.57 | down |
| A_23_P152876 | *RAB34* | 6.50E-06 | 2.57 | down |
| A_24_P131392 | *FAM82A1* | 1.90E-07 | 2.57 | down |
| A_24_P369898 | *MYO15B* | 2.48E-07 | 2.57 | down |
| A_23_P211910 | *PLOD2* | 1.28E-03 | 2.56 | down |
| A_32_P58215 | *CD84* | 3.45E-05 | 2.56 | down |
| A_23_P331748 | *CD33* | 1.47E-07 | 2.56 | down |
| A_24_P85775 | *C1orf38* | 4.12E-06 | 2.56 | down |
| A_23_P16283 | *CPT1C* | 7.95E-05 | 2.56 | down |
| A_23_P258769 | *HLA-DPB1* | 5.60E-05 | 2.56 | down |
| A_23_P32233 | *KLF4* | 3.41E-04 | 2.55 | down |
| A_32_P6408 | *ZNF853* | 2.03E-03 | 2.55 | down |
| A_23_P316741 | *TSPAN4* | 8.11E-09 | 2.55 | down |
| A_24_P645914 | *EFNA5* | 9.86E-04 | 2.55 | down |
| A_23_P46131 | *GRRP1* | 2.08E-06 | 2.55 | down |
| A_23_P433690 | *ZNF331* | 5.09E-03 | 2.55 | down |
| A_24_P282108 | *ZZEF1* | 5.40E-10 | 2.55 | down |
| A_32_P32463 |  | 3.96E-04 | 2.55 | down |
| A_23_P312840 | *SEMA6A* | 1.35E-04 | 2.55 | down |
| A_24_P142973 | *PEX26* | 1.14E-10 | 2.55 | down |
| A_24_P107303 | *IL10RA* | 6.45E-08 | 2.55 | down |
| A_24_P171268 | *RASSF5* | 6.56E-05 | 2.55 | down |
| A_23_P208389 | *AXL* | 1.59E-07 | 2.55 | down |
| A_24_P892402 |  | 3.64E-04 | 2.55 | down |
| A_23_P131801 | *SGK2* | 1.05E-04 | 2.54 | down |
| A_23_P351275 | *UPP1* | 2.13E-05 | 2.54 | down |
| A_23_P94338 | *ENPP2* | 5.29E-04 | 2.54 | down |
| A_24_P246891 | *NEU4* | 6.98E-03 | 2.54 | down |
| A_23_P1759 | *AMICA1* | 1.15E-05 | 2.54 | down |
| A_23_P428129 | *CDKN1C* | 1.97E-04 | 2.54 | down |
| A_23_P67198 | *CPAMD8* | 2.86E-04 | 2.54 | down |
| A_23_P166087 | *RASSF2* | 3.05E-05 | 2.54 | down |
| A_23_P49610 | *C17orf91* | 1.98E-03 | 2.54 | down |
| A_24_P71244 | *PIK3CD* | 1.60E-05 | 2.53 | down |
| A_23_P407565 | *CX3CR1* | 2.17E-04 | 2.53 | down |
| A_24_P402242 | *COL3A1* | 9.75E-04 | 2.53 | down |
| A_24_P166443 | *HLA-DPB1* | 2.80E-05 | 2.53 | down |
| A_23_P98876 | *SLC39A5* | 7.54E-04 | 2.53 | down |
| A_23_P78018 | *ABCA5* | 2.33E-06 | 2.53 | down |
| A_24_P127159 |  | 1.19E-05 | 2.53 | down |
| A_23_P142533 | *COL3A1* | 1.14E-04 | 2.52 | down |
| A_23_P133656 | *LAMA4* | 3.71E-07 | 2.52 | down |
| A_24_P354488 | *NAAA* | 8.18E-06 | 2.52 | down |
| A_32_P163036 | *C2orf74* | 1.22E-03 | 2.52 | down |
| A_23_P65918 | *ITPKA* | 1.10E-03 | 2.52 | down |
| A_32_P75792 | *FAM132A* | 9.66E-03 | 2.52 | down |
| A_23_P44335 | *ENTPD8* | 4.21E-03 | 2.52 | down |
| A_23_P48088 | *CD27* | 8.90E-03 | 2.52 | down |
| A_24_P331882 | *KIAA1211* | 1.48E-03 | 2.52 | down |
| A_23_P9883 | *NLRP3* | 1.02E-07 | 2.52 | down |
| A_23_P149975 | *FAM107B* | 2.80E-05 | 2.51 | down |
| A_32_P166693 | *HEG1* | 1.60E-05 | 2.51 | down |
| A_23_P64617 | *FZD4* | 3.14E-09 | 2.51 | down |
| A_23_P71867 | *IL11RA* | 5.02E-07 | 2.51 | down |
| A_23_P88069 | *LHFP* | 3.79E-04 | 2.51 | down |
| A_23_P212655 | *KLHL6* | 5.19E-03 | 2.51 | down |
| A_23_P50217 | *ZNF671* | 1.10E-03 | 2.50 | down |
| A_32_P515920 | *LOC400573* | 7.71E-06 | 2.50 | down |
| A_23_P413641 | *PREX1* | 1.09E-05 | 2.50 | down |
| A_23_P363647 | *DDX26B* | 5.29E-05 | 2.50 | down |
| A_23_P324813 | *BCL6B* | 6.24E-05 | 2.50 | down |
| A_23_P167168 | *IGJ* | 5.24E-03 | 2.50 | down |
| A_24_P141214 | *STOM* | 8.99E-05 | 2.50 | down |
| A_23_P141688 | *RAB31* | 9.73E-05 | 2.50 | down |
| A_32_P66625 |  | 9.40E-04 | 2.49 | down |
| A_23_P150057 | *MMRN2* | 2.73E-04 | 2.49 | down |
| A_23_P101642 | *PTPRH* | 4.67E-06 | 2.49 | down |
| A_23_P13822 | *STYK1* | 3.47E-07 | 2.49 | down |
| A_23_P408996 | *MBOAT1* | 1.15E-05 | 2.49 | down |
| A_24_P109432 | *NBEAL1* | 4.49E-04 | 2.49 | down |
| A_24_P131522 | *ANTXR1* | 2.91E-07 | 2.49 | down |
| A_32_P216602 |  | 8.29E-03 | 2.48 | down |
| A_24_P151356 | *SGK2* | 3.15E-04 | 2.48 | down |
| A_23_P99275 | *KLRB1* | 3.66E-04 | 2.48 | down |
| A_23_P145631 | *GIMAP6* | 5.47E-05 | 2.48 | down |
| A_23_P124108 | *ITGAM* | 6.10E-03 | 2.47 | down |
| A_24_P673968 | *TTC22* | 2.66E-07 | 2.47 | down |
| A_23_P110569 | *TRIM36* | 5.51E-06 | 2.47 | down |
| A_32_P104746 | *ZFYVE28* | 1.18E-05 | 2.47 | down |
| A_32_P130641 | *STARD9* | 1.61E-05 | 2.47 | down |
| A_23_P35045 | *ARHGAP30* | 6.85E-05 | 2.47 | down |
| A_23_P165848 | *EMILIN1* | 5.63E-06 | 2.47 | down |
| A_32_P46495 |  | 4.43E-03 | 2.46 | down |
| A_24_P372643 | *SLC22A18AS* | 1.01E-04 | 2.46 | down |
| A_24_P173823 | *PBX1* | 7.98E-06 | 2.46 | down |
| A_23_P35114 | *PLEKHO1* | 2.02E-06 | 2.46 | down |
| A_23_P211561 | *MEI1* | 7.58E-03 | 2.46 | down |
| A_24_P335656 | *SECTM1* | 4.26E-06 | 2.46 | down |
| A_24_P106166 |  | 4.96E-04 | 2.46 | down |
| A_23_P405754 | *CACNB1* | 2.95E-04 | 2.46 | down |
| A_24_P254949 | *PGM5* | 9.88E-04 | 2.45 | down |
| A_23_P74290 | *GBP5* | 9.06E-03 | 2.45 | down |
| A_23_P78265 | *KRT33A* | 1.35E-04 | 2.45 | down |
| A_23_P143526 | *S100B* | 3.11E-03 | 2.45 | down |
| A_23_P314101 | *SUSD2* | 2.55E-04 | 2.45 | down |
| A_32_P351968 | *HLA-DMB* | 6.22E-05 | 2.45 | down |
| A_23_P150325 | *TMEM133* | 5.71E-09 | 2.45 | down |
| A_23_P18447 | *PPARGC1A* | 1.11E-05 | 2.44 | down |
| A_23_P142796 | *LIMS2* | 1.71E-03 | 2.44 | down |
| A_23_P400378 | *GPBAR1* | 1.75E-07 | 2.44 | down |
| A_32_P226786 | *FAM126B* | 1.15E-08 | 2.44 | down |
| A_23_P500861 | *SYNE1* | 3.00E-06 | 2.44 | down |
| A_23_P67339 | *RCN3* | 4.33E-05 | 2.44 | down |
| A_23_P122007 | *C5orf30* | 3.46E-10 | 2.44 | down |
| A_24_P45728 | *CGN* | 3.81E-05 | 2.43 | down |
| A_23_P202334 | *FGFR2* | 2.44E-04 | 2.43 | down |
| A_24_P283189 | *CD14* | 5.53E-06 | 2.43 | down |
| A_23_P7325 | *BST1* | 3.02E-03 | 2.43 | down |
| A_32_P104432 | *NCRNA00087* | 3.39E-04 | 2.43 | down |
| A_23_P251937 | *CPEB4* | 4.75E-06 | 2.43 | down |
| A_23_P166376 | *GGT5* | 9.36E-06 | 2.43 | down |
| A_23_P203983 | *RHOF* | 4.78E-05 | 2.43 | down |
| A_23_P88849 | *RRAD* | 3.91E-04 | 2.43 | down |
| A_23_P99741 | *CDKL1* | 2.83E-06 | 2.43 | down |
| A_24_P88850 | *MRAS* | 2.83E-05 | 2.43 | down |
| A_23_P397480 | *ABCC13* | 5.16E-03 | 2.43 | down |
| A_23_P873 | *C1orf38* | 6.41E-05 | 2.42 | down |
| A_23_P62967 | *DISC1* | 1.58E-04 | 2.42 | down |
| A_32_P194563 |  | 1.69E-03 | 2.42 | down |
| A_23_P158330 | *UGT1A8* | 2.59E-03 | 2.42 | down |
| A_23_P163467 | *C15orf52* | 1.91E-05 | 2.42 | down |
| A_23_P376557 | *MMP25* | 1.12E-05 | 2.42 | down |
| A_23_P27994 | *TYROBP* | 7.46E-05 | 2.42 | down |
| A_24_P148796 | *MST1* | 4.44E-03 | 2.42 | down |
| A_23_P72651 | *ECSCR* | 2.85E-04 | 2.41 | down |
| A_23_P344673 | *LOC401022* | 2.86E-03 | 2.41 | down |
| A_24_P301655 | *CD33* | 1.31E-06 | 2.41 | down |
| A_23_P205074 | *SLC46A3* | 1.72E-05 | 2.41 | down |
| A_24_P69654 | *KLF6* | 7.59E-07 | 2.41 | down |
| A_24_P254437 | *CBX6* | 2.42E-05 | 2.41 | down |
| A_23_P13382 | *LSP1* | 4.61E-04 | 2.41 | down |
| A_32_P226907 | *LOC284112* | 1.77E-08 | 2.41 | down |
| A_23_P336708 | *DTX3* | 1.09E-03 | 2.41 | down |
| A_24_P926993 |  | 1.78E-04 | 2.41 | down |
| A_23_P105442 | *GRASP* | 1.38E-04 | 2.41 | down |
| A_24_P625683 |  | 2.16E-03 | 2.41 | down |
| A_24_P316059 |  | 9.04E-05 | 2.40 | down |
| A_24_P188975 | *FLJ11235* | 5.78E-03 | 2.40 | down |
| A_23_P159907 | *MAGED4B* | 7.62E-04 | 2.40 | down |
| A_24_P328504 | *SP140* | 1.18E-03 | 2.40 | down |
| A_24_P103004 | *SLC20A1* | 8.36E-04 | 2.40 | down |
| A_23_P202219 | *CALHM2* | 4.30E-06 | 2.40 | down |
| A_32_P72758 | *MCTP2* | 5.24E-03 | 2.40 | down |
| A_23_P254079 | *STBD1* | 5.44E-05 | 2.39 | down |
| A_23_P102000 | *CXCR4* | 9.12E-04 | 2.39 | down |
| A_24_P243528 | *HLA-DPA1* | 8.02E-06 | 2.39 | down |
| A_32_P31832 |  | 3.99E-03 | 2.39 | down |
| A_23_P258136 | *MXRA5* | 4.13E-03 | 2.39 | down |
| A_32_P47874 |  | 5.27E-05 | 2.39 | down |
| A_23_P257993 | *DNASE1L3* | 2.08E-04 | 2.39 | down |
| A_23_P339818 | *ARRDC4* | 7.18E-05 | 2.39 | down |
| A_23_P92025 | *CIDEC* | 7.11E-07 | 2.39 | down |
| A_23_P201790 | *PPP1R12B* | 5.12E-05 | 2.39 | down |
| A_23_P434919 | *RAB42* | 5.84E-04 | 2.39 | down |
| A_23_P390032 | *TMEM20* | 1.55E-06 | 2.39 | down |
| A_24_P634768 | *FLJ22763* | 8.94E-03 | 2.39 | down |
| A_24_P68079 | *TRANK1* | 1.12E-04 | 2.39 | down |
| A_23_P159237 | *GPR20* | 1.47E-03 | 2.39 | down |
| A_23_P114903 | *HSPA6* | 1.74E-06 | 2.39 | down |
| A_24_P345081 | *NBPF3* | 3.36E-04 | 2.38 | down |
| A_23_P73429 | *HCLS1* | 6.77E-04 | 2.38 | down |
| A_23_P419239 | *ETNK1* | 9.48E-04 | 2.38 | down |
| A_24_P50666 |  | 8.92E-04 | 2.38 | down |
| A_24_P922261 | *SRGAP1* | 9.08E-03 | 2.38 | down |
| A_23_P25336 | *GLTP* | 9.37E-06 | 2.38 | down |
| A_23_P92161 | *ARL14* | 4.11E-05 | 2.38 | down |
| A_23_P145874 | *SAMD9L* | 7.42E-04 | 2.37 | down |
| A_32_P99171 | *CHST11* | 1.75E-04 | 2.37 | down |
| A_32_P163469 | *NFE2L1* | 2.07E-03 | 2.37 | down |
| A_23_P83277 | *IL11RA* | 6.89E-06 | 2.37 | down |
| A_24_P205130 | *FNBP1* | 2.57E-06 | 2.37 | down |
| A_23_P251118 | *LPP* | 7.57E-07 | 2.37 | down |
| A_23_P114689 | *ASAP3* | 5.04E-05 | 2.37 | down |
| A_23_P113161 | *C1orf21* | 1.05E-05 | 2.37 | down |
| A_23_P256735 | *PGCP* | 1.35E-04 | 2.37 | down |
| A_23_P77073 | *SPPL2A* | 1.54E-06 | 2.36 | down |
| A_23_P304110 | *ANKRD43* | 1.73E-04 | 2.36 | down |
| A_24_P370372 | *CBX6* | 1.77E-08 | 2.36 | down |
| A_23_P110961 | *BRP44L* | 1.60E-06 | 2.36 | down |
| A_23_P61447 | *ETFDH* | 3.69E-07 | 2.35 | down |
| A_24_P23625 | *HS3ST3B1* | 1.89E-06 | 2.35 | down |
| A_23_P118254 | *FOXF1* | 3.09E-06 | 2.35 | down |
| A_23_P149998 | *PBLD* | 3.95E-05 | 2.35 | down |
| A_24_P467073 | *hCG_1643808* | 2.02E-04 | 2.35 | down |
| A_32_P198412 |  | 2.62E-04 | 2.35 | down |
| A_23_P217258 | *CYBB* | 6.96E-06 | 2.35 | down |
| A_24_P263144 | *BMX* | 7.83E-03 | 2.34 | down |
| A_23_P17095 | *TFPI* | 6.87E-04 | 2.34 | down |
| A_23_P131990 | *VSX1* | 2.15E-04 | 2.34 | down |
| A_23_P216845 | *GFI1B* | 3.23E-05 | 2.34 | down |
| A_23_P108280 | *CYP4F12* | 5.48E-05 | 2.34 | down |
| A_23_P302550 | *RGS18* | 1.54E-05 | 2.34 | down |
| A_23_P20864 | *ANGPTL2* | 5.94E-05 | 2.34 | down |
| A_23_P33256 | *GP9* | 5.69E-05 | 2.34 | down |
| A_23_P394064 | *PTRF* | 3.76E-06 | 2.34 | down |
| A_24_P650482 | *LOC400960* | 5.53E-06 | 2.34 | down |
| A_23_P24515 | *ACAT1* | 2.13E-07 | 2.34 | down |
| A_23_P91910 | *PLSCR4* | 4.51E-06 | 2.33 | down |
| A_23_P24077 | *C10orf54* | 6.40E-12 | 2.33 | down |
| A_23_P60599 | *UGT1A6* | 2.65E-03 | 2.33 | down |
| A_23_P80594 | *PLCL2* | 2.61E-06 | 2.33 | down |
| A_23_P502343 | *ADAM33* | 7.69E-07 | 2.33 | down |
| A_23_P110957 | *FOXF2* | 1.24E-07 | 2.33 | down |
| A_23_P32175 | *LHX6* | 1.94E-04 | 2.33 | down |
| A_32_P32254 | *COL6A1* | 1.75E-04 | 2.33 | down |
| A_23_P253350 | *C8orf4* | 5.68E-03 | 2.33 | down |
| A_23_P23346 | *MLLT11* | 2.19E-06 | 2.33 | down |
| A_23_P211233 | *COL6A2* | 4.37E-03 | 2.33 | down |
| A_32_P47754 | *SLC2A14* | 6.54E-04 | 2.32 | down |
| A_23_P23457 | *FBLIM1* | 4.52E-07 | 2.32 | down |
| A_24_P828496 |  | 1.83E-04 | 2.32 | down |
| A_24_P398370 |  | 4.27E-05 | 2.32 | down |
| A_23_P334864 | *FAM126B* | 6.74E-09 | 2.32 | down |
| A_23_P5281 | *LYL1* | 3.49E-07 | 2.32 | down |
| A_32_P9368 |  | 1.26E-04 | 2.32 | down |
| A_24_P161293 |  | 5.42E-04 | 2.32 | down |
| A_23_P131060 | *CYP4F8* | 2.57E-04 | 2.32 | down |
| A_23_P50508 | *PLA2G4C* | 2.23E-04 | 2.32 | down |
| A_23_P259561 |  | 1.66E-05 | 2.31 | down |
| A_23_P412321 | *CCR5* | 1.61E-05 | 2.31 | down |
| A_23_P125977 | *C1QC* | 2.77E-04 | 2.31 | down |
| A_24_P896205 | *LOC645722* | 4.61E-04 | 2.31 | down |
| A_23_P377957 | *KCTD12* | 4.87E-03 | 2.31 | down |
| A_24_P829261 | *MALAT1* | 9.42E-04 | 2.31 | down |
| A_23_P119923 | *CNNM4* | 6.15E-08 | 2.31 | down |
| A_24_P324787 | *KANK2* | 3.29E-04 | 2.30 | down |
| A_23_P17663 | *MX1* | 6.80E-04 | 2.30 | down |
| A_23_P433758 | *SPN* | 9.94E-05 | 2.30 | down |
| A_23_P87742 | *IFFO1* | 1.66E-07 | 2.30 | down |
| A_23_P1962 | *RARRES3* | 5.37E-03 | 2.30 | down |
| A_23_P253317 | *GPR171* | 2.82E-04 | 2.30 | down |
| A_23_P502142 | *FYN* | 4.23E-05 | 2.30 | down |
| A_23_P105562 | *VWF* | 2.11E-03 | 2.30 | down |
| A_24_P586264 |  | 1.06E-03 | 2.30 | down |
| A_24_P277934 | *COL1A2* | 5.58E-04 | 2.30 | down |
| A_23_P24044 | *CNNM2* | 1.72E-06 | 2.29 | down |
| A_23_P160177 | *ATP1A4* | 8.47E-03 | 2.29 | down |
| A_24_P373562 | *ADAP2* | 1.61E-06 | 2.29 | down |
| A_23_P11800 | *CAMK2N1* | 1.54E-04 | 2.29 | down |
| A_23_P39465 | *BST2* | 1.84E-03 | 2.29 | down |
| A_24_P208345 | *SLC45A3* | 1.73E-04 | 2.29 | down |
| A_23_P119353 | *RASIP1* | 6.43E-05 | 2.29 | down |
| A_32_P202708 |  | 4.80E-03 | 2.28 | down |
| A_24_P237443 | *SASH3* | 1.68E-03 | 2.28 | down |
| A_23_P11201 | *GPR34* | 7.14E-06 | 2.28 | down |
| A_32_P83465 | *NBPF10* | 3.76E-04 | 2.28 | down |
| A_24_P294832 | *PTP4A1* | 1.58E-05 | 2.28 | down |
| A_23_P20566 | *TPM2* | 1.42E-05 | 2.27 | down |
| A_23_P86390 | *NRP1* | 4.44E-03 | 2.27 | down |
| A_24_P182122 |  | 2.33E-05 | 2.27 | down |
| A_23_P50376 | *ZNF331* | 1.26E-04 | 2.27 | down |
| A_23_P5983 | *PLTP* | 6.78E-04 | 2.27 | down |
| A_23_P29096 | *PDE9A* | 2.32E-03 | 2.27 | down |
| A_24_P845631 |  | 9.81E-04 | 2.27 | down |
| A_23_P252471 | *PECAM1* | 1.39E-03 | 2.27 | down |
| A_23_P104346 | *PIP4K2A* | 2.98E-05 | 2.27 | down |
| A_23_P90357 | *TBXA2R* | 5.62E-03 | 2.26 | down |
| A_23_P254271 | *TUBB6* | 9.52E-06 | 2.26 | down |
| A_24_P71373 | *SLC9A1* | 4.93E-07 | 2.26 | down |
| A_24_P842872 |  | 6.70E-04 | 2.26 | down |
| A_24_P843921 |  | 3.49E-03 | 2.25 | down |
| A_23_P22460 | *XIAP* | 1.77E-04 | 2.25 | down |
| A_23_P127565 | *LAYN* | 4.12E-06 | 2.25 | down |
| A_24_P135061 | *RP11-94I2.2* | 8.72E-04 | 2.25 | down |
| A_23_P25030 | *HSD17B6* | 9.66E-05 | 2.25 | down |
| A_23_P156880 | *ENPP1* | 1.35E-04 | 2.25 | down |
| A_24_P280029 | *PDXP* | 1.02E-05 | 2.25 | down |
| A_23_P320216 | *FAM55D* | 1.00E-02 | 2.25 | down |
| A_23_P354805 | *KLF12* | 3.75E-03 | 2.25 | down |
| A_23_P252052 | *FILIP1L* | 3.15E-04 | 2.25 | down |
| A_23_P72001 | *PHYHD1* | 2.99E-04 | 2.24 | down |
| A_23_P51926 | *PTAFR* | 3.48E-04 | 2.24 | down |
| A_23_P329573 | *ITGB2* | 1.86E-04 | 2.24 | down |
| A_24_P215653 | *CLEC14A* | 1.99E-03 | 2.24 | down |
| A_23_P152505 | *ABAT* | 1.26E-03 | 2.24 | down |
| A_32_P171793 |  | 3.80E-04 | 2.24 | down |
| A_23_P253046 | *UGP2* | 1.28E-09 | 2.24 | down |
| A_24_P365526 | *HCK* | 1.80E-04 | 2.23 | down |
| A_23_P160433 | *C1orf115* | 1.89E-04 | 2.23 | down |
| A_23_P72025 | *SLC25A20* | 8.46E-05 | 2.23 | down |
| A_24_P189533 | *ENDOD1* | 4.25E-05 | 2.23 | down |
| A_24_P145911 | *TRA2B* | 5.17E-04 | 2.23 | down |
| A_24_P62530 | *RHOU* | 3.73E-05 | 2.23 | down |
| A_32_P107029 | *NAPSA* | 2.14E-04 | 2.23 | down |
| A_23_P360329 | *AIM1L* | 1.85E-04 | 2.23 | down |
| A_24_P249824 | *DYRK2* | 8.01E-04 | 2.23 | down |
| A_23_P14302 | *C14orf139* | 2.24E-04 | 2.23 | down |
| A_23_P162589 | *VDR* | 2.12E-07 | 2.22 | down |
| A_23_P203629 | *IL18BP* | 5.04E-05 | 2.22 | down |
| A_24_P218814 | *RDH5* | 2.54E-03 | 2.22 | down |
| A_23_P155477 | *C3orf18* | 4.99E-06 | 2.22 | down |
| A_32_P179686 |  | 1.86E-04 | 2.22 | down |
| A_32_P135336 | *LOC388242* | 6.81E-03 | 2.22 | down |
| A_23_P200670 | *WDR78* | 1.60E-04 | 2.22 | down |
| A_23_P162288 | *MYO1A* | 6.68E-04 | 2.22 | down |
| A_24_P330385 | *SLC22A17* | 4.78E-05 | 2.22 | down |
| A_23_P308519 | *SLC9A3R1* | 1.32E-06 | 2.22 | down |
| A_23_P101093 | *COPZ2* | 7.49E-06 | 2.21 | down |
| A_23_P104624 | *ENDOD1* | 5.63E-06 | 2.21 | down |
| A_23_P208450 | *SLC25A23* | 1.13E-07 | 2.21 | down |
| A_23_P5051 | *GNA11* | 1.37E-09 | 2.21 | down |
| A_23_P24004 | *IFIT2* | 8.40E-04 | 2.21 | down |
| A_24_P112395 | *PBLD* | 1.11E-04 | 2.21 | down |
| A_23_P62647 | *SLAMF1* | 1.00E-03 | 2.21 | down |
| A_32_P42236 |  | 4.37E-04 | 2.21 | down |
| A_24_P200162 | *HIGD1A* | 5.07E-06 | 2.21 | down |
| A_23_P983 | *PRDX6* | 7.98E-08 | 2.20 | down |
| A_23_P164451 | *TBX2* | 2.62E-04 | 2.20 | down |
| A_24_P201739 | *SH2B3* | 1.74E-05 | 2.20 | down |
| A_23_P50710 | *CYP4F2* | 1.72E-04 | 2.20 | down |
| A_23_P210900 | *ACSS2* | 1.76E-06 | 2.20 | down |
| A_23_P144959 | *VCAN* | 6.70E-04 | 2.20 | down |
| A_24_P365767 | *CYBB* | 2.96E-06 | 2.19 | down |
| A_23_P152428 | *MARVELD3* | 1.41E-04 | 2.19 | down |
| A_23_P90125 | *NAPSB* | 2.16E-03 | 2.19 | down |
| A_23_P81441 | *C5orf20* | 1.82E-03 | 2.19 | down |
| A_23_P252201 | *EAF2* | 4.53E-04 | 2.19 | down |
| A_32_P35595 |  | 1.50E-04 | 2.19 | down |
| A_23_P203475 | *PRKCDBP* | 9.08E-04 | 2.19 | down |
| A_23_P210708 | *SIRPA* | 3.61E-05 | 2.19 | down |
| A_23_P34915 | *ATF3* | 2.53E-03 | 2.19 | down |
| A_23_P401076 | *SUSD3* | 4.82E-04 | 2.19 | down |
| A_23_P93722 | *SDK1* | 1.76E-05 | 2.19 | down |
| A_24_P32085 | *MOBKL2B* | 1.87E-05 | 2.19 | down |
| A_32_P57717 |  | 1.45E-04 | 2.18 | down |
| A_23_P19226 | *DSE* | 1.89E-04 | 2.18 | down |
| A_23_P163787 | *MMP2* | 7.67E-04 | 2.18 | down |
| A_23_P343104 | *FLJ30901* | 1.30E-03 | 2.18 | down |
| A_23_P19095 | *SNX24* | 1.24E-04 | 2.18 | down |
| A_23_P28507 | *MGAT4A* | 3.35E-04 | 2.18 | down |
| A_23_P164528 | *WDR7* | 2.28E-09 | 2.18 | down |
| A_23_P79069 | *RASAL3* | 1.57E-03 | 2.17 | down |
| A_23_P203115 | *TMEM25* | 3.41E-03 | 2.17 | down |
| A_23_P213745 | *CXCL14* | 1.06E-04 | 2.17 | down |
| A_23_P214627 | *AIF1* | 2.92E-07 | 2.17 | down |
| A_32_P167239 | *AFAP1L1* | 6.69E-04 | 2.17 | down |
| A_32_P223504 |  | 4.39E-03 | 2.17 | down |
| A_32_P39049 | *LPP* | 7.22E-04 | 2.17 | down |
| A_23_P85800 | *CD52* | 9.11E-04 | 2.17 | down |
| A_23_P24234 | *OPN4* | 1.94E-05 | 2.16 | down |
| A_23_P160167 | *TSPAN1* | 1.19E-04 | 2.16 | down |
| A_24_P634530 | *CPPED1* | 2.84E-04 | 2.16 | down |
| A_23_P29237 | *APOL3* | 1.81E-03 | 2.16 | down |
| A_23_P142255 | *SHD* | 1.86E-04 | 2.16 | down |
| A_32_P4934 |  | 2.21E-04 | 2.16 | down |
| A_23_P104224 | *A1CF* | 1.37E-03 | 2.16 | down |
| A_32_P31785 |  | 8.49E-05 | 2.16 | down |
| A_23_P76529 | *ITGB7* | 4.90E-05 | 2.16 | down |
| A_24_P937765 |  | 4.96E-04 | 2.16 | down |
| A_23_P167129 | *HHIP* | 1.80E-04 | 2.16 | down |
| A_24_P215352 | *PRKCDBP* | 8.37E-06 | 2.15 | down |
| A_32_P61145 |  | 6.13E-06 | 2.15 | down |
| A_24_P485742 |  | 2.40E-04 | 2.15 | down |
| A_24_P59361 | *KIF16B* | 3.34E-03 | 2.15 | down |
| A_32_P21646 | *FAM120AOS* | 1.39E-05 | 2.15 | down |
| A_23_P56356 | *PLB1* | 6.28E-08 | 2.15 | down |
| A_32_P42367 |  | 2.79E-03 | 2.14 | down |
| A_24_P6903 | *ACTBL2* | 2.96E-05 | 2.14 | down |
| A_23_P207911 | *TRPV2* | 7.07E-04 | 2.14 | down |
| A_32_P203939 |  | 1.48E-04 | 2.14 | down |
| A_23_P30913 | *HLA-DPA1* | 9.40E-05 | 2.14 | down |
| A_32_P224522 | *SLC25A23* | 2.66E-07 | 2.14 | down |
| A_23_P163567 | *SMPD3* | 2.86E-06 | 2.14 | down |
| A_23_P125451 | *PTPRC* | 1.20E-03 | 2.14 | down |
| A_32_P140656 | *IL6ST* | 2.39E-07 | 2.14 | down |
| A_32_P177725 |  | 1.47E-03 | 2.14 | down |
| A_32_P25397 | *PBX1* | 5.52E-03 | 2.14 | down |
| A_23_P74042 | *LPHN2* | 5.30E-06 | 2.14 | down |
| A_23_P354074 | *LYST* | 6.15E-06 | 2.14 | down |
| A_23_P68006 | *IL1R1* | 8.98E-05 | 2.14 | down |
| A_23_P90497 | *LILRA4* | 3.74E-05 | 2.14 | down |
| A_24_P943613 | *TBC1D1* | 4.17E-05 | 2.14 | down |
| A_32_P475513 | *MYO15B* | 3.45E-04 | 2.13 | down |
| A_23_P130598 | *SLC25A23* | 4.57E-07 | 2.13 | down |
| A_32_P119949 |  | 5.64E-05 | 2.13 | down |
| A_23_P41992 | *GFRA3* | 6.36E-06 | 2.13 | down |
| A_23_P117580 | *ENTPD5* | 2.27E-03 | 2.13 | down |
| A_23_P5002 | *MAP4K1* | 4.10E-04 | 2.12 | down |
| A_24_P659415 |  | 1.99E-04 | 2.12 | down |
| A_23_P204847 | *LCP1* | 7.11E-04 | 2.12 | down |
| A_32_P163247 | *CD8A* | 4.75E-03 | 2.12 | down |
| A_24_P179183 | *ANKRD12* | 5.67E-04 | 2.12 | down |
| A_23_P211207 | *ADARB1* | 1.35E-04 | 2.12 | down |
| A_24_P139120 | *PDE4C* | 4.72E-04 | 2.12 | down |
| A_23_P147805 | *UPP1* | 7.63E-05 | 2.12 | down |
| A_32_P23360 | *YPEL2* | 8.23E-04 | 2.12 | down |
| A_24_P392201 | *TBC1D10C* | 1.94E-03 | 2.12 | down |
| A_23_P32404 | *ISG20* | 2.79E-04 | 2.12 | down |
| A_24_P235429 | *ABCA1* | 6.40E-04 | 2.12 | down |
| A_23_P106761 | *CORO1A* | 8.38E-04 | 2.12 | down |
| A_32_P47543 |  | 8.75E-05 | 2.11 | down |
| A_24_P754185 | *KIAA1109* | 1.20E-04 | 2.11 | down |
| A_24_P261417 | *DKK3* | 5.48E-04 | 2.11 | down |
| A_23_P61987 | *TMEM121* | 2.20E-05 | 2.11 | down |
| A_23_P136173 | *CSF2RA* | 4.63E-04 | 2.11 | down |
| A_24_P59430 | *FAM55B* | 1.58E-04 | 2.11 | down |
| A_23_P81770 | *PTP4A1* | 3.69E-05 | 2.11 | down |
| A_24_P102821 | *PTAFR* | 1.66E-04 | 2.11 | down |
| A_23_P319423 | *KCNK5* | 1.07E-04 | 2.11 | down |
| A_32_P210642 | *EGFL7* | 6.35E-04 | 2.11 | down |
| A_32_P184888 |  | 9.47E-05 | 2.11 | down |
| A_23_P17430 | *RBM38* | 7.89E-04 | 2.11 | down |
| A_23_P211522 | *SYNGR1* | 2.12E-05 | 2.11 | down |
| A_24_P372613 | *APBB1* | 1.84E-07 | 2.11 | down |
| A_32_P32739 | *NAGS* | 2.79E-05 | 2.10 | down |
| A_23_P64611 | *P2RY6* | 1.15E-03 | 2.10 | down |
| A_23_P58132 | *RHOH* | 2.15E-03 | 2.10 | down |
| A_23_P29005 | *SAMSN1* | 9.92E-04 | 2.10 | down |
| A_24_P182461 | *IGSF3* | 1.74E-05 | 2.10 | down |
| A_24_P151582 | *TEF* | 5.21E-05 | 2.10 | down |
| A_23_P216468 | *SLC1A1* | 2.53E-03 | 2.10 | down |
| A_23_P330070 | *TFPI* | 1.73E-03 | 2.10 | down |
| A_32_P30905 | *WDFY4* | 1.33E-03 | 2.10 | down |
| A_23_P109636 | *LRIG1* | 3.04E-03 | 2.09 | down |
| A_32_P174083 | *CYCS* | 1.53E-04 | 2.09 | down |
| A_23_P3911 | *PLXDC1* | 5.72E-03 | 2.09 | down |
| A_24_P94222 | *FBLIM1* | 2.54E-05 | 2.09 | down |
| A_32_P70158 | *LILRB3* | 9.58E-03 | 2.09 | down |
| A_24_P190190 | *CACNB1* | 3.17E-03 | 2.09 | down |
| A_23_P132185 | *SCARF2* | 3.83E-09 | 2.09 | down |
| A_23_P82979 | *LAMC3* | 5.28E-04 | 2.09 | down |
| A_23_P368711 | *LILRB3* | 2.82E-04 | 2.09 | down |
| A_23_P77859 | *TMEM88* | 2.14E-06 | 2.09 | down |
| A_23_P21560 | *FAM49A* | 1.10E-03 | 2.09 | down |
| A_23_P309261 | *AKAP9* | 8.50E-06 | 2.09 | down |
| A_23_P111981 | *LYNX1* | 5.10E-05 | 2.08 | down |
| A_24_P150466 | *SMOC1* | 3.87E-03 | 2.08 | down |
| A_23_P74547 | *CD53* | 1.64E-03 | 2.08 | down |
| A_24_P396702 | *CD302* | 2.54E-05 | 2.08 | down |
| A_23_P29922 | *TLR3* | 1.04E-03 | 2.08 | down |
| A_24_P402690 | *ITM2C* | 5.68E-04 | 2.08 | down |
| A_23_P156811 | *FAM119A* | 4.39E-03 | 2.08 | down |
| A_32_P232825 |  | 3.96E-04 | 2.08 | down |
| A_23_P152970 | *RAPGEFL1* | 3.79E-04 | 2.08 | down |
| A_24_P131173 | *C1orf115* | 8.42E-04 | 2.08 | down |
| A_24_P373174 | *RAB27A* | 4.66E-04 | 2.07 | down |
| A_24_P174755 | *SLC22A5* | 2.79E-06 | 2.07 | down |
| A_23_P166371 | *VPREB3* | 1.35E-03 | 2.07 | down |
| A_24_P148717 | *CCR1* | 3.03E-03 | 2.07 | down |
| A_23_P82868 | *PLAT* | 4.14E-04 | 2.07 | down |
| A_23_P4190 | *ACSF2* | 3.43E-04 | 2.07 | down |
| A_32_P59475 |  | 1.23E-03 | 2.07 | down |
| A_23_P97123 | *ANKRD36BP1* | 4.40E-03 | 2.07 | down |
| A_23_P503072 | *CCL28* | 1.20E-03 | 2.07 | down |
| A_32_P18470 | *TCEAL5* | 1.37E-04 | 2.06 | down |
| A_23_P40718 | *PARVB* | 3.45E-03 | 2.06 | down |
| A_24_P101072 | *NBPF10* | 1.89E-03 | 2.06 | down |
| A_23_P36120 | *MS4A6A* | 3.87E-05 | 2.06 | down |
| A_23_P128319 | *ATP2B1* | 1.29E-05 | 2.06 | down |
| A_23_P91390 | *THBD* | 5.78E-03 | 2.06 | down |
| A_23_P126825 | *SLC16A1* | 2.89E-03 | 2.06 | down |
| A_23_P39386 | *HCST* | 2.13E-05 | 2.06 | down |
| A_24_P12521 | *C7orf31* | 6.37E-04 | 2.06 | down |
| A_24_P935491 | *COL3A1* | 4.19E-04 | 2.06 | down |
| A_24_P294233 | *GLS* | 1.30E-04 | 2.06 | down |
| A_32_P185766 |  | 1.33E-03 | 2.06 | down |
| A_24_P931955 |  | 8.66E-03 | 2.06 | down |
| A_23_P112634 | *C4orf34* | 5.52E-04 | 2.05 | down |
| A_23_P38816 | *A1BG* | 4.08E-05 | 2.05 | down |
| A_24_P149036 | *DPYSL3* | 5.90E-03 | 2.05 | down |
| A_23_P208866 | *GMFG* | 9.64E-04 | 2.05 | down |
| A_24_P229766 |  | 1.45E-03 | 2.05 | down |
| A_23_P405794 | *NAGS* | 1.32E-04 | 2.05 | down |
| A_23_P55936 | *FCGRT* | 3.43E-04 | 2.05 | down |
| A_23_P116512 | *PRR5L* | 2.28E-07 | 2.05 | down |
| A_24_P398810 | *EIF5* | 4.31E-03 | 2.05 | down |
| A_24_P38276 | *FZD1* | 2.09E-08 | 2.05 | down |
| A_32_P465065 | *C20orf135* | 5.42E-04 | 2.05 | down |
| A_24_P367666 | *ASH1L* | 4.37E-03 | 2.05 | down |
| A_23_P81717 | *FRMD1* | 4.23E-04 | 2.05 | down |
| A_23_P311912 | *AHNAK2* | 8.44E-03 | 2.04 | down |
| A_23_P74241 | *MTF1* | 5.93E-04 | 2.04 | down |
| A_24_P213478 | *SEMA6A* | 2.39E-03 | 2.04 | down |
| A_24_P64167 | *PTGS1* | 3.88E-03 | 2.04 | down |
| A_23_P353056 | *C2CD2L* | 5.90E-11 | 2.04 | down |
| A_23_P98410 | *CD3G* | 6.50E-03 | 2.04 | down |
| A_24_P224926 | *MFNG* | 1.42E-04 | 2.04 | down |
| A_24_P459621 | *FLJ34515* | 8.67E-04 | 2.04 | down |
| A_23_P104493 | *PAPSS2* | 9.54E-04 | 2.04 | down |
| A_23_P69531 | *KLB* | 2.44E-04 | 2.04 | down |
| A_24_P379820 | *ITM2C* | 3.47E-03 | 2.04 | down |
| A_23_P38959 | *VAV1* | 6.10E-03 | 2.03 | down |
| A_23_P305723 | *MIER1* | 1.56E-10 | 2.03 | down |
| A_32_P126362 |  | 2.89E-03 | 2.03 | down |
| A_24_P67096 | *ABCA5* | 2.18E-03 | 2.03 | down |
| A_23_P401547 | *PVRL3* | 6.91E-04 | 2.03 | down |
| A_24_P798431 |  | 1.83E-04 | 2.03 | down |
| A_23_P129246 | *PLEKHO2* | 3.13E-05 | 2.03 | down |
| A_23_P217109 | *SLC31A2* | 4.29E-05 | 2.03 | down |
| A_23_P86470 | *CH25H* | 3.74E-03 | 2.03 | down |
| A_24_P930927 |  | 4.33E-04 | 2.02 | down |
| A_23_P257155 | *ATXN7* | 7.23E-04 | 2.02 | down |
| A_23_P50646 | *LOC390940* | 4.65E-03 | 2.02 | down |
| A_23_P433760 | *SPN* | 4.46E-03 | 2.02 | down |
| A_32_P170406 | *TLN2* | 7.50E-03 | 2.02 | down |
| A_24_P153713 | *MARVELD3* | 7.69E-03 | 2.02 | down |
| A_23_P119362 | *EMP3* | 1.22E-03 | 2.02 | down |
| A_32_P524014 | *UTRN* | 6.58E-04 | 2.02 | down |
| A_23_P314250 | *FAM78A* | 3.20E-03 | 2.02 | down |
| A_32_P121085 | *DOK3* | 5.31E-03 | 2.02 | down |
| A_24_P403959 | *RNASE1* | 9.92E-03 | 2.02 | down |
| A_24_P819729 |  | 9.56E-03 | 2.02 | down |
| A_23_P22350 | *GRAMD3* | 9.15E-06 | 2.02 | down |
| A_24_P169234 | *ZAP70* | 2.14E-03 | 2.02 | down |
| A_32_P147651 |  | 7.54E-04 | 2.02 | down |
| A_23_P253012 | *GRAMD1C* | 6.51E-05 | 2.02 | down |
| A_23_P124642 | *RASGRP1* | 2.52E-03 | 2.01 | down |
| A_23_P406187 | *NAGS* | 2.60E-04 | 2.01 | down |
| A_23_P147869 | *KIAA1109* | 9.18E-04 | 2.01 | down |
| A_24_P150665 | *RUNDC2A* | 1.53E-04 | 2.01 | down |
| A_23_P137470 | *SIPA1L2* | 1.47E-04 | 2.01 | down |
| A_23_P48596 | *RNASE1* | 8.01E-03 | 2.01 | down |
| A_23_P50426 | *KANK2* | 9.39E-07 | 2.01 | down |
| A_32_P190613 |  | 2.66E-04 | 2.00 | down |
| A_23_P119916 | *WNT6* | 7.32E-03 | 2.00 | down |
| A_24_P239183 | *MUC4* | 8.00E-03 | 2.00 | down |
| A_32_P103815 |  | 2.11E-06 | 2.00 | down |
| A_23_P103532 | *GPR161* | 7.71E-03 | 2.00 | down |
| A_23_P48455 | *AMN* | 2.57E-03 | 2.00 | down |
| A_23_P52761 | *MMP7* | 1.74E-15 | 116.11 | up |
| A_23_P324754 | *KIAA1199* | 4.96E-17 | 45.89 | up |
| A_32_P161855 | *KIAA1199* | 8.20E-17 | 42.64 | up |
| A_23_P161698 | *MMP3* | 2.34E-08 | 42.07 | up |
| A_23_P49155 | *CDH3* | 6.25E-18 | 39.62 | up |
| A_23_P151851 | *DUOX2* | 7.04E-09 | 27.02 | up |
| A_32_P164246 | *FOXQ1* | 1.14E-13 | 23.58 | up |
| A_23_P169437 | *LCN2* | 5.92E-09 | 18.89 | up |
| A_24_P58673 | *REG4* | 5.76E-09 | 17.47 | up |
| A_23_P149529 | *TACSTD2* | 2.13E-08 | 16.42 | up |
| A_32_P200238 | *UCA1* | 1.01E-06 | 15.96 | up |
| A_24_P35905 | *DUOX2* | 2.11E-08 | 15.12 | up |
| A_24_P183150 | *CXCL3* | 6.41E-08 | 14.67 | up |
| A_23_P2789 | *OLFM4* | 5.56E-06 | 13.34 | up |
| A_23_P135381 | *SP5* | 5.21E-14 | 13.26 | up |
| A_23_P400310 | *REG4* | 1.08E-06 | 13.19 | up |
| A_32_P183718 | *SP5* | 2.64E-14 | 12.59 | up |
| A_23_P83339 | *RNF183* | 5.48E-14 | 12.43 | up |
| A_32_P171061 | *ASCL2* | 6.82E-12 | 10.80 | up |
| A_23_P57784 | *CLDN1* | 3.34E-12 | 10.19 | up |
| A_23_P201706 | *S100A2* | 3.46E-10 | 9.92 | up |
| A_23_P315364 | *CXCL2* | 2.24E-06 | 9.56 | up |
| A_23_P148015 | *AXIN2* | 7.71E-15 | 9.38 | up |
| A_23_P1691 | *MMP1* | 6.33E-06 | 9.31 | up |
| A_23_P58266 | *S100P* | 1.71E-12 | 9.17 | up |
| A_24_P257416 | *CXCL2* | 1.08E-06 | 8.37 | up |
| A_24_P174793 | *PCSK1* | 3.39E-03 | 7.80 | up |
| A_23_P32454 | *TG* | 4.41E-07 | 7.48 | up |
| A_23_P218646 | *TNFRSF6B* | 1.81E-04 | 7.42 | up |
| A_23_P159395 | *AXIN2* | 2.38E-12 | 7.09 | up |
| A_23_P207850 | *TNS4* | 9.76E-09 | 7.07 | up |
| A_23_P5903 | *SLCO4A1* | 6.19E-13 | 6.89 | up |
| A_23_P23980 | *C10orf81* | 4.64E-11 | 6.88 | up |
| A_24_P286951 | *C10orf81* | 4.06E-10 | 6.85 | up |
| A_23_P76538 | *TESC* | 1.03E-06 | 6.82 | up |
| A_24_P398147 | *NEBL* | 1.57E-18 | 6.72 | up |
| A_23_P72411 | *CYP4X1* | 6.02E-06 | 6.56 | up |
| A_23_P16915 | *QPCT* | 3.28E-08 | 6.52 | up |
| A_23_P90032 | *LRRC8E* | 2.80E-11 | 6.07 | up |
| A_23_P65696 | *DUOXA2* | 1.85E-06 | 5.99 | up |
| A_24_P788878 | *C2CD4B* | 8.65E-06 | 5.84 | up |
| A_23_P4212 | *HOXB13* | 3.66E-03 | 5.77 | up |
| A_23_P7144 | *CXCL1* | 1.14E-05 | 5.74 | up |
| A_23_P122924 | *INHBA* | 1.66E-06 | 5.72 | up |
| A_32_P50603 | *C2orf70* | 4.50E-08 | 5.51 | up |
| A_23_P69326 | *CADPS* | 5.44E-10 | 5.46 | up |
| A_23_P366376 | *TDGF1* | 4.44E-09 | 5.45 | up |
| A_24_P251764 | *CXCL3* | 1.38E-05 | 5.34 | up |
| A_24_P348203 | *LRRC8E* | 1.50E-10 | 5.34 | up |
| A_24_P703830 | *NANOS3* | 1.47E-05 | 5.28 | up |
| A_24_P71468 | *QPCT* | 2.83E-07 | 5.26 | up |
| A_23_P428298 | *UNC5CL* | 6.63E-08 | 5.25 | up |
| A_23_P204133 | *GALNT6* | 4.53E-08 | 4.85 | up |
| A_23_P118042 | *LRRC36* | 1.18E-12 | 4.81 | up |
| A_23_P121716 | *ANXA3* | 1.77E-15 | 4.70 | up |
| A_23_P102731 | *SMOX* | 7.66E-09 | 4.66 | up |
| A_23_P154875 | *BACE2* | 3.58E-13 | 4.64 | up |
| A_23_P53198 | *DGAT2* | 4.99E-10 | 4.62 | up |
| A_24_P42264 | *LYZ* | 3.38E-04 | 4.62 | up |
| A_24_P295791 | *DGAT2* | 3.43E-10 | 4.61 | up |
| A_23_P14019 | *APOBEC1* | 2.46E-05 | 4.54 | up |
| A_32_P158181 |  | 3.96E-10 | 4.54 | up |
| A_23_P56213 | *GRAMD1A* | 1.56E-09 | 4.53 | up |
| A_23_P142878 | *ATOH8* | 7.02E-06 | 4.51 | up |
| A_24_P14584 | *BACE2* | 1.50E-12 | 4.50 | up |
| A_24_P46093 | *SLC6A6* | 1.17E-08 | 4.48 | up |
| A_32_P183904 | *SHF* | 2.01E-06 | 4.41 | up |
| A_23_P16523 | *GDF15* | 1.21E-07 | 4.38 | up |
| A_23_P356684 | *ANLN* | 1.53E-08 | 4.31 | up |
| A_23_P49338 | *TNFRSF12A* | 4.62E-08 | 4.31 | up |
| A_23_P403445 | *CGREF1* | 1.56E-03 | 4.29 | up |
| A_23_P160920 | *PDZK1IP1* | 7.39E-08 | 4.25 | up |
| A_23_P54055 | *JUB* | 2.03E-08 | 4.24 | up |
| A_23_P218111 | *SERPINA1* | 2.17E-07 | 4.24 | up |
| A_32_P127153 | *SORD* | 3.82E-12 | 4.23 | up |
| A_32_P77742 |  | 1.77E-03 | 4.23 | up |
| A_23_P69537 | *NMU* | 1.68E-03 | 4.22 | up |
| A_23_P77493 | *TUBB3* | 2.12E-03 | 4.16 | up |
| A_24_P234196 | *RRM2* | 1.17E-08 | 4.13 | up |
| A_23_P257457 | *SDR16C5* | 1.37E-04 | 4.11 | up |
| A_23_P380298 | *ProSAPiP1* | 1.79E-09 | 4.10 | up |
| A_23_P145863 | *S100A11* | 6.43E-11 | 4.08 | up |
| A_23_P109171 | *BFSP1* | 3.34E-11 | 4.05 | up |
| A_23_P70827 | *KIAA1549* | 2.61E-12 | 4.02 | up |
| A_23_P76450 | *PHLDA1* | 1.32E-06 | 4.02 | up |
| A_23_P394304 | *PDZK1IP1* | 6.28E-08 | 3.98 | up |
| A_24_P181254 | *OLFM4* | 4.23E-05 | 3.98 | up |
| A_24_P69095 | *ENC1* | 4.46E-10 | 3.98 | up |
| A_23_P77103 | *SORD* | 1.01E-09 | 3.97 | up |
| A_23_P42718 | *NFE2L3* | 4.22E-10 | 3.97 | up |
| A_23_P92349 | *FGFRL1* | 2.60E-07 | 3.94 | up |
| A_23_P156327 | *TGFBI* | 6.22E-09 | 3.94 | up |
| A_32_P234184 | *HES5* | 5.54E-04 | 3.92 | up |
| A_23_P3934 | *RNF43* | 8.25E-12 | 3.89 | up |
| A_23_P46390 | *SYTL1* | 4.28E-07 | 3.84 | up |
| A_23_P257694 | *GTF2IRD1* | 1.81E-09 | 3.84 | up |
| A_23_P19733 | *SLC22A3* | 3.43E-07 | 3.83 | up |
| A_24_P20806 | *PRR7* | 3.64E-08 | 3.82 | up |
| A_23_P133606 | *SLC12A2* | 3.62E-11 | 3.81 | up |
| A_24_P911676 | *SOX4* | 5.38E-09 | 3.79 | up |
| A_23_P170667 | *ASPHD1* | 6.02E-06 | 3.79 | up |
| A_23_P398854 | *DOK7* | 6.55E-06 | 3.78 | up |
| A_23_P133694 | *SLC29A1* | 5.17E-11 | 3.78 | up |
| A_32_P217773 | *SYTL1* | 1.58E-06 | 3.76 | up |
| A_32_P205624 | *SHC2* | 3.03E-06 | 3.71 | up |
| A_23_P101374 | *CYP2S1* | 5.02E-09 | 3.70 | up |
| A_23_P166508 |  | 1.04E-09 | 3.70 | up |
| A_23_P146572 | *NPDC1* | 1.32E-09 | 3.68 | up |
| A_23_P373031 | *CACNA1C* | 4.83E-06 | 3.67 | up |
| A_23_P63402 | *GPSM2* | 1.30E-10 | 3.67 | up |
| A_32_P135243 | *MTHFD1L* | 2.91E-09 | 3.67 | up |
| A_32_P25437 | *SLC12A2* | 2.54E-12 | 3.63 | up |
| A_23_P126593 | *S100A11* | 3.59E-09 | 3.63 | up |
| A_32_P89691 | *SORD* | 9.03E-09 | 3.61 | up |
| A_23_P24716 | *TMEM132A* | 5.50E-06 | 3.60 | up |
| A_32_P399546 | *ARNTL2* | 2.91E-08 | 3.58 | up |
| A_32_P8221 | *GRM8* | 1.23E-03 | 3.57 | up |
| A_23_P160537 | *C1orf135* | 2.07E-09 | 3.57 | up |
| A_24_P331128 | *GNA15* | 1.18E-08 | 3.57 | up |
| A_23_P7582 | *TCF7* | 8.77E-07 | 3.56 | up |
| A_23_P134935 | *DUSP4* | 3.02E-05 | 3.55 | up |
| A_23_P71268 | *AZGP1* | 1.58E-04 | 3.53 | up |
| A_23_P76078 | *IL23A* | 9.38E-04 | 3.53 | up |
| A_32_P142459 |  | 2.04E-08 | 3.51 | up |
| A_24_P335620 | *SLC7A5* | 5.49E-04 | 3.50 | up |
| A_23_P168443 | *EPHB4* | 6.58E-07 | 3.50 | up |
| A_24_P751220 | *LOC157860* | 2.21E-06 | 3.50 | up |
| A_23_P331895 | *TTYH3* | 8.02E-08 | 3.49 | up |
| A_23_P50000 | *FAM57A* | 1.97E-09 | 3.49 | up |
| A_23_P153197 | *TGIF1* | 1.54E-09 | 3.47 | up |
| A_23_P112774 | *PTP4A3* | 4.43E-04 | 3.46 | up |
| A_23_P315386 | *RHPN1* | 2.85E-07 | 3.44 | up |
| A_32_P152696 | *LOC729983* | 3.78E-09 | 3.43 | up |
| A_24_P379233 | *GJB3* | 1.02E-04 | 3.43 | up |
| A_24_P602871 | *SAMD5* | 5.11E-05 | 3.42 | up |
| A_23_P111621 | *GTF2IRD1* | 6.40E-12 | 3.40 | up |
| A_23_P427502 | *ZNRF3* | 4.56E-07 | 3.39 | up |
| A_32_P206899 | *DNAH2* | 5.71E-06 | 3.36 | up |
| A_24_P8371 | *SPNS2* | 5.24E-07 | 3.33 | up |
| A_23_P150316 | *MMP12* | 2.98E-03 | 3.30 | up |
| A_23_P206077 | *AEN* | 2.57E-08 | 3.30 | up |
| A_23_P81298 | *RGNEF* | 9.84E-10 | 3.29 | up |
| A_24_P235049 | *MTHFD1L* | 5.06E-07 | 3.29 | up |
| A_24_P924484 |  | 2.78E-05 | 3.26 | up |
| A_23_P10194 | *SEZ6L2* | 3.87E-07 | 3.26 | up |
| A_24_P100517 | *C9orf140* | 1.09E-07 | 3.25 | up |
| A_23_P30464 | *PRR7* | 3.85E-08 | 3.25 | up |
| A_23_P201758 | *CD46* | 2.00E-05 | 3.22 | up |
| A_24_P392496 | *LOC100133920* | 1.55E-09 | 3.19 | up |
| A_23_P210690 | *TRIB3* | 1.73E-04 | 3.17 | up |
| A_23_P78405 | *LIPG* | 2.83E-07 | 3.15 | up |
| A_32_P157671 |  | 1.43E-06 | 3.15 | up |
| A_23_P362719 | *LSM14B* | 6.17E-03 | 3.14 | up |
| A_23_P35230 | *CD46* | 2.29E-05 | 3.13 | up |
| A_23_P115261 | *AGT* | 3.76E-05 | 3.13 | up |
| A_23_P259586 | *TTK* | 3.73E-07 | 3.13 | up |
| A_23_P71270 | *AZGP1* | 2.74E-04 | 3.12 | up |
| A_23_P211504 | *KDELR3* | 1.03E-06 | 3.12 | up |
| A_23_P256956 | *KIF20A* | 2.69E-08 | 3.12 | up |
| A_23_P201459 | *IFI6* | 3.86E-05 | 3.12 | up |
| A_32_P209094 | *FGGY* | 1.77E-06 | 3.12 | up |
| A_23_P340698 | *MMP12* | 3.13E-03 | 3.11 | up |
| A_23_P254741 | *SOD3* | 3.29E-04 | 3.09 | up |
| A_23_P359245 | *MET* | 8.65E-10 | 3.09 | up |
| A_23_P26847 | *SOX9* | 8.15E-09 | 3.09 | up |
| A_23_P106814 |  | 9.17E-09 | 3.08 | up |
| A_23_P214908 | *MTHFD1L* | 4.07E-07 | 3.06 | up |
| A_23_P428326 | *TBC1D16* | 2.32E-10 | 3.05 | up |
| A_23_P32577 | *DACH1* | 2.26E-07 | 3.04 | up |
| A_24_P406006 | *LPCAT1* | 1.34E-05 | 3.03 | up |
| A_23_P41804 | *NKD2* | 4.38E-03 | 3.03 | up |
| A_23_P157404 | *AP1S1* | 5.74E-05 | 3.02 | up |
| A_23_P210726 | *CDC25B* | 2.23E-06 | 3.02 | up |
| A_23_P422115 | *C9orf116* | 5.86E-08 | 3.01 | up |
| A_23_P131935 | *FERMT1* | 2.60E-09 | 3.00 | up |
| A_23_P160968 | *LAMC2* | 1.06E-06 | 3.00 | up |
| A_23_P208880 | *UHRF1* | 1.68E-05 | 2.98 | up |
| A_24_P151622 | *IMMP2L* | 1.98E-07 | 2.97 | up |
| A_23_P387552 | *RARG* | 3.18E-09 | 2.97 | up |
| A_23_P152678 | *B9D1* | 2.20E-08 | 2.96 | up |
| A_23_P143958 | *RPL22L1* | 4.45E-06 | 2.95 | up |
| A_23_P146456 | *CTSL2* | 1.77E-05 | 2.95 | up |
| A_23_P139600 | *RASAL1* | 6.13E-08 | 2.94 | up |
| A_24_P42495 |  | 1.34E-05 | 2.94 | up |
| A_24_P79040 | *CAPN12* | 5.35E-06 | 2.93 | up |
| A_23_P162165 | *KCTD14* | 1.09E-09 | 2.93 | up |
| A_23_P45786 | *COL9A2* | 3.80E-05 | 2.93 | up |
| A_23_P156687 | *CFB* | 3.50E-03 | 2.92 | up |
| A_23_P139820 | *SLC11A2* | 5.70E-08 | 2.92 | up |
| A_23_P401472 | *CHRM3* | 1.86E-05 | 2.91 | up |
| A_24_P322771 | *TFF1* | 7.87E-03 | 2.90 | up |
| A_24_P382661 | *ETS2* | 3.63E-07 | 2.89 | up |
| A_23_P67042 | *MOCOS* | 1.48E-05 | 2.89 | up |
| A_23_P344481 | *STOX1* | 1.42E-06 | 2.89 | up |
| A_23_P28898 | *PLCB4* | 3.82E-05 | 2.88 | up |
| A_23_P129903 | *TRIM16L* | 9.99E-06 | 2.88 | up |
| A_23_P94795 | *TEAD4* | 2.42E-07 | 2.87 | up |
| A_23_P54517 | *TYRO3* | 9.83E-07 | 2.86 | up |
| A_23_P319598 | *C4BPB* | 2.66E-03 | 2.85 | up |
| A_24_P67494 |  | 1.35E-07 | 2.85 | up |
| A_23_P23839 | *LGR6* | 3.99E-06 | 2.85 | up |
| A_23_P134910 | *GGH* | 3.41E-06 | 2.84 | up |
| A_23_P215956 | *MYC* | 1.31E-06 | 2.83 | up |
| A_23_P145786 | *MLXIPL* | 5.92E-04 | 2.83 | up |
| A_23_P38677 | *SLMO1* | 9.62E-06 | 2.82 | up |
| A_23_P98282 | *SPTBN2* | 6.67E-07 | 2.81 | up |
| A_23_P95029 | *SNTB1* | 2.59E-06 | 2.81 | up |
| A_23_P95060 | *EPHB3* | 5.89E-05 | 2.81 | up |
| A_23_P24870 | *CD44* | 3.36E-12 | 2.80 | up |
| A_23_P401718 | *CCDC74B* | 3.35E-05 | 2.80 | up |
| A_23_P369328 | *C10orf35* | 2.74E-11 | 2.80 | up |
| A_24_P571937 |  | 1.49E-03 | 2.80 | up |
| A_24_P377499 | *OSBPL3* | 8.29E-11 | 2.79 | up |
| A_24_P212834 |  | 6.46E-07 | 2.79 | up |
| A_23_P164814 | *C19orf57* | 4.30E-06 | 2.79 | up |
| A_24_P122137 | *LIF* | 4.09E-06 | 2.78 | up |
| A_23_P366253 | *PATZ1* | 6.08E-10 | 2.77 | up |
| A_23_P100963 | *SPNS3* | 8.58E-09 | 2.77 | up |
| A_23_P59375 | *ID4* | 2.62E-04 | 2.76 | up |
| A_24_P257099 | *HJURP* | 7.39E-07 | 2.75 | up |
| A_23_P256890 | *PTRH1* | 9.55E-08 | 2.75 | up |
| A_23_P71530 | *TNFRSF11B* | 2.17E-05 | 2.74 | up |
| A_23_P369899 | *TMEM158* | 3.77E-03 | 2.74 | up |
| A_32_P103695 | *FAM92A1* | 4.48E-05 | 2.73 | up |
| A_23_P99292 | *RAD51AP1* | 1.49E-06 | 2.73 | up |
| A_32_P95147 |  | 6.15E-10 | 2.73 | up |
| A_23_P429491 | *C11orf82* | 5.87E-07 | 2.73 | up |
| A_23_P111297 | *RPP40* | 1.06E-09 | 2.72 | up |
| A_24_P194081 | *FXYD5* | 4.99E-05 | 2.72 | up |
| A_24_P416079 | *NUSAP1* | 2.43E-07 | 2.72 | up |
| A_24_P181672 | *B3GNTL1* | 8.54E-09 | 2.72 | up |
| A_23_P130995 | *FXYD5* | 1.85E-04 | 2.71 | up |
| A_24_P413884 | *CENPA* | 4.70E-06 | 2.71 | up |
| A_23_P213424 | *ENC1* | 2.90E-04 | 2.71 | up |
| A_23_P310 | *MARCKSL1* | 2.90E-05 | 2.70 | up |
| A_32_P34522 |  | 9.87E-05 | 2.70 | up |
| A_32_P209230 | *CITED4* | 7.59E-04 | 2.70 | up |
| A_24_P873688 | *CENPN* | 1.17E-06 | 2.70 | up |
| A_23_P168556 | *STX1A* | 7.50E-06 | 2.69 | up |
| A_24_P109614 |  | 2.13E-06 | 2.68 | up |
| A_23_P44684 | *ECT2* | 5.38E-08 | 2.68 | up |
| A_24_P287941 | *PSMC3IP* | 1.20E-07 | 2.68 | up |
| A_23_P20022 | *C7orf68* | 1.06E-09 | 2.67 | up |
| A_23_P17826 | *SLC5A1* | 2.08E-06 | 2.67 | up |
| A_24_P383450 | *IER5L* | 6.74E-06 | 2.67 | up |
| A_23_P118834 | *TOP2A* | 7.71E-06 | 2.66 | up |
| A_23_P118203 | *ZG16B* | 1.44E-04 | 2.66 | up |
| A_23_P103756 | *OVGP1* | 1.11E-05 | 2.65 | up |
| A_24_P339272 | *NCRNA00188* | 6.96E-05 | 2.65 | up |
| A_23_P21706 | *CTPS* | 2.35E-07 | 2.65 | up |
| A_23_P92441 | *MAD2L1* | 4.30E-07 | 2.65 | up |
| A_32_P163858 | *SCD* | 4.71E-04 | 2.64 | up |
| A_24_P170203 |  | 1.31E-06 | 2.64 | up |
| A_23_P27656 | *C19orf48* | 6.44E-07 | 2.63 | up |
| A_23_P65812 | *SMAD6* | 4.14E-08 | 2.63 | up |
| A_23_P42331 | *HMGA1* | 3.83E-10 | 2.63 | up |
| A_32_P19840 |  | 1.94E-03 | 2.63 | up |
| A_23_P23303 | *EXO1* | 2.52E-07 | 2.63 | up |
| A_24_P188377 | *CD55* | 1.22E-03 | 2.63 | up |
| A_32_P63324 |  | 8.97E-07 | 2.63 | up |
| A_24_P413126 | *PMEPA1* | 3.16E-05 | 2.63 | up |
| A_23_P22224 | *EIF4EBP1* | 2.91E-05 | 2.62 | up |
| A_32_P226768 |  | 6.12E-06 | 2.62 | up |
| A_24_P225616 | *RRM2* | 4.05E-05 | 2.62 | up |
| A_24_P161403 | *LOC131055* | 6.88E-04 | 2.62 | up |
| A_32_P177955 | *LOC441461* | 3.43E-07 | 2.62 | up |
| A_24_P255663 |  | 2.93E-04 | 2.61 | up |
| A_23_P52207 | *BAMBI* | 1.47E-04 | 2.61 | up |
| A_23_P132175 | *RTN4R* | 4.60E-07 | 2.61 | up |
| A_23_P306211 | *FAM84A* | 1.56E-04 | 2.61 | up |
| A_23_P86731 | *ZNF239* | 7.74E-06 | 2.61 | up |
| A_24_P322354 | *SKA1* | 3.42E-07 | 2.61 | up |
| A_23_P16409 | *CAPN12* | 1.52E-04 | 2.61 | up |
| A_23_P16516 | *ARID3A* | 4.53E-05 | 2.60 | up |
| A_24_P314179 | *ETS2* | 9.37E-09 | 2.60 | up |
| A_23_P313632 | *FUT8* | 5.75E-07 | 2.60 | up |
| A_23_P52278 | *KIF11* | 1.89E-06 | 2.59 | up |
| A_32_P76720 | *NT5DC3* | 3.24E-05 | 2.59 | up |
| A_23_P207537 | *DUSP14* | 7.47E-09 | 2.59 | up |
| A_23_P82420 | *STX1A* | 7.10E-06 | 2.59 | up |
| A_32_P44316 | *EEF1A1* | 2.74E-03 | 2.59 | up |
| A_23_P57379 | *CDC45L* | 7.93E-06 | 2.59 | up |
| A_23_P19657 | *LRP11* | 2.14E-06 | 2.59 | up |
| A_23_P169030 | *TNFRSF10B* | 3.81E-09 | 2.58 | up |
| A_24_P10214 | *STXBP6* | 4.92E-07 | 2.58 | up |
| A_23_P165783 | *MLPH* | 1.50E-05 | 2.58 | up |
| A_23_P401904 | *PHF19* | 5.02E-07 | 2.58 | up |
| A_24_P274814 | *TBXAS1* | 4.46E-05 | 2.58 | up |
| A_24_P116587 | *SEZ6L2* | 1.11E-03 | 2.57 | up |
| A_23_P202206 | *GSTO2* | 6.11E-08 | 2.57 | up |
| A_23_P88331 | *DLGAP5* | 9.12E-07 | 2.57 | up |
| A_23_P9574 | *ECT2* | 1.22E-06 | 2.57 | up |
| A_23_P154400 | *MLPH* | 5.82E-06 | 2.57 | up |
| A_23_P116235 | *MDK* | 3.68E-04 | 2.57 | up |
| A_23_P44836 | *NT5DC2* | 9.84E-06 | 2.56 | up |
| A_24_P171549 | *CDCA7* | 2.40E-06 | 2.56 | up |
| A_24_P387158 | *THOC4* | 9.32E-05 | 2.56 | up |
| A_23_P82738 | *RAD54B* | 9.74E-08 | 2.56 | up |
| A_23_P36464 | *C12orf11* | 1.35E-07 | 2.56 | up |
| A_24_P176714 | *B9D1* | 3.07E-07 | 2.55 | up |
| A_23_P88522 | *NMB* | 2.50E-08 | 2.55 | up |
| A_32_P87872 | *IMMP2L* | 8.76E-07 | 2.55 | up |
| A_24_P32895 |  | 2.25E-04 | 2.55 | up |
| A_23_P156957 | *NCOA7* | 9.62E-04 | 2.54 | up |
| A_32_P162709 |  | 5.77E-04 | 2.54 | up |
| A_23_P206661 | *NQO1* | 1.20E-04 | 2.54 | up |
| A_23_P333951 | *DNAH14* | 4.67E-07 | 2.54 | up |
| A_23_P26557 | *C16orf59* | 3.21E-05 | 2.54 | up |
| A_23_P38505 | *CXCL16* | 4.92E-08 | 2.54 | up |
| A_23_P500936 | *FOXA2* | 9.39E-06 | 2.54 | up |
| A_23_P110802 | *CENPH* | 5.47E-08 | 2.54 | up |
| A_23_P70249 | *CDC25C* | 2.04E-05 | 2.53 | up |
| A_24_P915692 | *PHLDA1* | 5.27E-05 | 2.53 | up |
| A_23_P27947 | *PDCD2L* | 6.08E-10 | 2.53 | up |
| A_23_P306215 | *FAM84A* | 2.32E-10 | 2.52 | up |
| A_23_P52298 | *NPM3* | 5.76E-06 | 2.52 | up |
| A_23_P121783 | *SRD5A3* | 4.05E-06 | 2.52 | up |
| A_24_P43391 | *TMEM165* | 3.73E-07 | 2.51 | up |
| A_23_P38388 | *TMEM92* | 3.88E-05 | 2.51 | up |
| A_23_P323930 | *TSPAN5* | 4.81E-05 | 2.51 | up |
| A_32_P62997 | *PBK* | 1.52E-06 | 2.51 | up |
| A_23_P48001 |  | 3.38E-04 | 2.50 | up |
| A_23_P106145 | *ERO1L* | 1.18E-04 | 2.50 | up |
| A_23_P413051 | *NOXO1* | 2.48E-05 | 2.50 | up |
| A_23_P349676 | *FBXO41* | 3.67E-04 | 2.50 | up |
| A_24_P731549 |  | 3.32E-04 | 2.50 | up |
| A_24_P58727 |  | 1.49E-03 | 2.50 | up |
| A_23_P140725 | *IFT140* | 1.19E-06 | 2.49 | up |
| A_24_P289208 | *TFF3* | 1.33E-03 | 2.49 | up |
| A_23_P134419 | *ZP3* | 1.49E-03 | 2.49 | up |
| A_23_P167674 | *F12* | 2.88E-05 | 2.49 | up |
| A_23_P58642 | *PITX1* | 1.58E-04 | 2.49 | up |
| A_23_P60405 | *DDX31* | 1.45E-06 | 2.48 | up |
| A_23_P256051 | *RPL23AP7* | 1.80E-08 | 2.48 | up |
| A_23_P124417 | *BUB1* | 3.00E-08 | 2.48 | up |
| A_32_P171328 | *UBE2S* | 6.95E-06 | 2.47 | up |
| A_23_P259692 | *PSAT1* | 4.48E-03 | 2.47 | up |
| A_32_P140262 |  | 4.96E-06 | 2.47 | up |
| A_24_P192485 | *TNFRSF11B* | 1.21E-04 | 2.47 | up |
| A_23_P66608 | *KAT2A* | 6.10E-06 | 2.47 | up |
| A_23_P49878 | *FAM64A* | 1.89E-06 | 2.47 | up |
| A_23_P16078 | *PAFAH1B3* | 8.42E-07 | 2.46 | up |
| A_23_P200067 | *EPHB2* | 2.49E-06 | 2.46 | up |
| A_24_P74981 | *KDM1A* | 2.15E-05 | 2.46 | up |
| A_23_P399726 | *C22orf29* | 1.39E-07 | 2.46 | up |
| A_24_P166042 | *IMPDH2* | 2.51E-07 | 2.46 | up |
| A_32_P80068 | *CLEC2D* | 1.20E-06 | 2.46 | up |
| A_23_P130194 | *PYCR1* | 5.32E-07 | 2.46 | up |
| A_23_P44195 | *MSI2* | 1.23E-07 | 2.45 | up |
| A_23_P53763 | *C13orf18* | 2.39E-03 | 2.45 | up |
| A_32_P96272 | *PPIAL4A* | 2.28E-04 | 2.45 | up |
| A_23_P123193 | *ACTR3B* | 1.06E-07 | 2.45 | up |
| A_24_P323778 |  | 2.76E-04 | 2.45 | up |
| A_24_P89701 | *IMPDH1* | 5.30E-06 | 2.45 | up |
| A_24_P826046 |  | 1.62E-05 | 2.45 | up |
| A_23_P107087 | *TSR1* | 6.58E-07 | 2.45 | up |
| A_23_P138507 | *CDK1* | 1.51E-05 | 2.45 | up |
| A_24_P413920 | *FAM84A* | 8.02E-08 | 2.44 | up |
| A_23_P204751 | *ACCN2* | 9.61E-03 | 2.44 | up |
| A_23_P345460 | *PLEKHG4* | 2.82E-04 | 2.44 | up |
| A_23_P370989 | *MCM4* | 3.03E-07 | 2.44 | up |
| A_23_P90510 | *REEP6* | 1.59E-04 | 2.44 | up |
| A_23_P122197 | *CCNB1* | 8.77E-07 | 2.43 | up |
| A_24_P227831 | *ABCC1* | 2.59E-06 | 2.43 | up |
| A_23_P326160 | *ZC3H12A* | 4.65E-03 | 2.43 | up |
| A_23_P131202 | *HES6* | 4.25E-03 | 2.43 | up |
| A_24_P15821 |  | 2.29E-04 | 2.43 | up |
| A_23_P3038 | *GPX2* | 5.16E-06 | 2.42 | up |
| A_23_P130182 | *AURKB* | 3.69E-06 | 2.42 | up |
| A_24_P315184 | *RPL12* | 3.88E-06 | 2.42 | up |
| A_24_P392109 | *CENPN* | 1.62E-06 | 2.42 | up |
| A_23_P14508 | *TTC9* | 6.27E-05 | 2.40 | up |
| A_23_P421011 | *KAZALD1* | 8.24E-06 | 2.40 | up |
| A_23_P71727 | *CKS2* | 3.02E-06 | 2.40 | up |
| A_23_P68610 | *TPX2* | 6.12E-06 | 2.40 | up |
| A_23_P80032 | *E2F1* | 1.10E-04 | 2.40 | up |
| A_32_P231617 | *TM4SF1* | 3.05E-04 | 2.40 | up |
| A_23_P145606 | *CHRM2* | 9.66E-05 | 2.40 | up |
| A_23_P143190 | *MYBL2* | 3.96E-05 | 2.40 | up |
| A_23_P74950 | *RCC2* | 4.62E-08 | 2.40 | up |
| A_23_P378526 | *RTEL1* | 3.51E-03 | 2.40 | up |
| A_23_P46309 | *RCC1* | 1.75E-06 | 2.40 | up |
| A_32_P153781 |  | 2.50E-04 | 2.39 | up |
| A_23_P411335 | *SGOL2* | 7.59E-07 | 2.39 | up |
| A_23_P407112 | *SPATA18* | 2.07E-04 | 2.39 | up |
| A_23_P61487 | *LRRC20* | 1.36E-07 | 2.39 | up |
| A_23_P104617 | *GYLTL1B* | 1.82E-04 | 2.39 | up |
| A_23_P14432 |  | 4.65E-06 | 2.39 | up |
| A_32_P71447 | *NCAPD3* | 1.51E-07 | 2.39 | up |
| A_23_P72737 | *IFITM1* | 1.52E-05 | 2.39 | up |
| A_24_P268676 | *BHLHE40* | 6.13E-04 | 2.39 | up |
| A_24_P505255 |  | 1.03E-04 | 2.38 | up |
| A_23_P133956 | *KIFC1* | 3.15E-05 | 2.38 | up |
| A_24_P190168 | *TMEM97* | 6.88E-08 | 2.37 | up |
| A_24_P230130 | *LOC401859* | 3.45E-04 | 2.37 | up |
| A_23_P39445 | *MEX3D* | 1.29E-04 | 2.37 | up |
| A_24_P320284 | *DHFR* | 4.23E-05 | 2.37 | up |
| A_23_P31584 | *RABL5* | 2.20E-06 | 2.37 | up |
| A_23_P14193 | *RFC3* | 4.57E-07 | 2.37 | up |
| A_23_P35219 | *NEK2* | 2.20E-06 | 2.37 | up |
| A_23_P250607 | *PLS3* | 2.09E-04 | 2.37 | up |
| A_32_P406142 |  | 1.04E-03 | 2.37 | up |
| A_23_P75790 | *C11orf9* | 1.04E-05 | 2.37 | up |
| A_24_P20630 | *LEF1* | 9.66E-04 | 2.37 | up |
| A_23_P13222 | *RCN1* | 3.84E-06 | 2.36 | up |
| A_23_P115872 | *CEP55* | 1.54E-03 | 2.36 | up |
| A_23_P370097 | *ALS2CR4* | 1.27E-09 | 2.36 | up |
| A_23_P323751 | *FAM83D* | 1.93E-05 | 2.36 | up |
| A_23_P397341 | *PAQR4* | 1.86E-04 | 2.36 | up |
| A_23_P36689 | *LRRC23* | 4.27E-06 | 2.36 | up |
| A_23_P38154 | *FDXR* | 1.99E-06 | 2.36 | up |
| A_23_P399501 | *PKM2* | 4.93E-06 | 2.35 | up |
| A_23_P150935 | *TROAP* | 6.46E-05 | 2.35 | up |
| A_23_P304921 | *NOX1* | 1.83E-03 | 2.35 | up |
| A_23_P66682 | *HOXB6* | 6.12E-03 | 2.35 | up |
| A_24_P918436 | *CSNK1E* | 1.61E-05 | 2.35 | up |
| A_23_P22169 | *FGGY* | 9.68E-04 | 2.35 | up |
| A_32_P109296 | *C15orf42* | 5.04E-05 | 2.35 | up |
| A_23_P382775 | *BBC3* | 6.28E-07 | 2.35 | up |
| A_24_P82142 | *TCF12* | 6.09E-05 | 2.34 | up |
| A_24_P94351 | *C1orf56* | 1.13E-05 | 2.34 | up |
| A_23_P105535 | *WDR51B* | 2.66E-06 | 2.34 | up |
| A_24_P832113 | *NPM1* | 1.58E-06 | 2.34 | up |
| A_24_P218979 | *CDCA3* | 1.36E-06 | 2.34 | up |
| A_23_P422026 | *ME1* | 4.24E-03 | 2.34 | up |
| A_23_P66211 | *PAQR4* | 2.90E-05 | 2.34 | up |
| A_23_P118815 | *BIRC5* | 1.46E-06 | 2.34 | up |
| A_23_P123039 | *NUPL2* | 1.03E-05 | 2.34 | up |
| A_24_P385341 | *C1orf107* | 4.72E-09 | 2.34 | up |
| A_23_P104651 | *CDCA5* | 5.96E-07 | 2.34 | up |
| A_23_P251421 | *CDCA7* | 4.71E-05 | 2.34 | up |
| A_23_P6802 | *RRP9* | 1.05E-06 | 2.34 | up |
| A_24_P4054 | *TRIP6* | 3.95E-03 | 2.33 | up |
| A_23_P217049 | *NCS1* | 2.14E-04 | 2.33 | up |
| A_23_P201636 | *LAMC2* | 1.87E-05 | 2.33 | up |
| A_23_P58321 | *CCNA2* | 4.06E-05 | 2.33 | up |
| A_23_P151634 | *SUPT16H* | 3.17E-06 | 2.33 | up |
| A_23_P309361 | *C1orf59* | 6.55E-06 | 2.32 | up |
| A_32_P327750 |  | 4.02E-04 | 2.32 | up |
| A_23_P103011 | *RAB36* | 9.56E-03 | 2.32 | up |
| A_24_P101722 | *LOC126170* | 6.97E-04 | 2.32 | up |
| A_23_P206059 | *PRC1* | 6.21E-06 | 2.32 | up |
| A_24_P124550 | *CCND1* | 1.97E-05 | 2.32 | up |
| A_23_P164826 | *RNASEH2A* | 4.51E-06 | 2.31 | up |
| A_23_P109017 | *SFRS6* | 5.31E-03 | 2.31 | up |
| A_23_P214079 | *SPINK1* | 4.58E-04 | 2.31 | up |
| A_23_P165778 | *MLPH* | 8.64E-06 | 2.31 | up |
| A_23_P83838 | *CA8* | 8.18E-03 | 2.31 | up |
| A_23_P420981 | *C14orf79* | 1.41E-07 | 2.31 | up |
| A_23_P415443 | *NCAPH* | 1.47E-06 | 2.31 | up |
| A_32_P171923 | *MACC1* | 1.53E-04 | 2.31 | up |
| A_23_P112481 | *AQP3* | 2.89E-03 | 2.31 | up |
| A_24_P343095 | *DHFR* | 2.95E-05 | 2.30 | up |
| A_23_P160742 | *GLMN* | 9.52E-07 | 2.30 | up |
| A_23_P24997 | *CDK4* | 4.03E-05 | 2.30 | up |
| A_23_P168747 | *NCAPG2* | 8.34E-05 | 2.29 | up |
| A_23_P121898 | *PARP8* | 1.99E-04 | 2.29 | up |
| A_23_P140821 | *PARD6A* | 5.45E-05 | 2.29 | up |
| A_23_P150789 | *PRSS23* | 5.96E-04 | 2.29 | up |
| A_24_P89708 | *IMPDH1* | 1.45E-05 | 2.29 | up |
| A_32_P88892 | *RPL7A* | 2.76E-06 | 2.29 | up |
| A_24_P210829 | *NME4* | 5.56E-05 | 2.29 | up |
| A_23_P314151 | *NOLC1* | 2.38E-07 | 2.29 | up |
| A_23_P117852 | *KIAA0101* | 7.05E-05 | 2.29 | up |
| A_23_P52082 | *INTS7* | 3.22E-12 | 2.28 | up |
| A_23_P166453 | *CDC42EP1* | 9.40E-06 | 2.28 | up |
| A_23_P17739 | *ZNF74* | 9.83E-07 | 2.28 | up |
| A_23_P154675 | *SNRPB* | 7.42E-05 | 2.28 | up |
| A_32_P751535 |  | 5.52E-03 | 2.28 | up |
| A_24_P193295 | *RAB15* | 3.26E-06 | 2.28 | up |
| A_23_P57089 | *PMEPA1* | 1.64E-04 | 2.27 | up |
| A_24_P135515 |  | 9.15E-04 | 2.27 | up |
| A_23_P379864 | *ASRGL1* | 2.05E-04 | 2.27 | up |
| A_23_P27013 | *HOXB9* | 2.61E-07 | 2.27 | up |
| A_23_P206830 | *TIMM16* | 2.55E-05 | 2.27 | up |
| A_24_P323598 | *ESCO2* | 2.46E-05 | 2.27 | up |
| A_32_P211708 | *FZD3* | 4.02E-05 | 2.27 | up |
| A_23_P141315 | *NLE1* | 1.70E-08 | 2.27 | up |
| A_23_P145514 | *IL20RA* | 5.78E-05 | 2.27 | up |
| A_23_P146637 | *SIGMAR1* | 1.99E-07 | 2.26 | up |
| A_24_P178415 |  | 8.02E-04 | 2.26 | up |
| A_23_P205713 | *STXBP6* | 6.46E-07 | 2.26 | up |
| A_24_P7040 |  | 2.71E-04 | 2.26 | up |
| A_23_P138465 | *NOLC1* | 3.47E-05 | 2.26 | up |
| A_23_P106822 | *NOB1* | 2.46E-09 | 2.25 | up |
| A_23_P52286 | *DPCD* | 1.04E-07 | 2.25 | up |
| A_23_P51085 | *SPC25* | 1.97E-04 | 2.25 | up |
| A_24_P83678 | *C6orf167* | 3.69E-08 | 2.25 | up |
| A_24_P105102 | *PKMYT1* | 2.45E-05 | 2.25 | up |
| A_23_P166336 | *TMEM191A* | 7.38E-05 | 2.25 | up |
| A_23_P134734 | *GOLSYN* | 2.57E-08 | 2.24 | up |
| A_24_P204358 | *PYCR1* | 2.38E-04 | 2.24 | up |
| A_24_P115199 | *FOXK1* | 4.69E-05 | 2.24 | up |
| A_23_P112482 | *AQP3* | 7.31E-03 | 2.24 | up |
| A_32_P5976 | *NUDT14* | 2.07E-06 | 2.24 | up |
| A_23_P80098 | *GART* | 1.10E-09 | 2.24 | up |
| A_23_P200489 | *TMEM63A* | 2.82E-08 | 2.24 | up |
| A_24_P772061 | *PPIA* | 2.90E-04 | 2.24 | up |
| A_23_P163099 | *POLE2* | 2.92E-06 | 2.24 | up |
| A_24_P340866 |  | 1.49E-06 | 2.24 | up |
| A_23_P385322 | *STAMBPL1* | 1.73E-08 | 2.24 | up |
| A_24_P916195 |  | 5.88E-05 | 2.24 | up |
| A_23_P47788 | *METTL1* | 1.32E-05 | 2.24 | up |
| A_23_P40880 | *CMTM8* | 1.52E-09 | 2.24 | up |
| A_32_P155416 | *ERI3* | 5.52E-08 | 2.24 | up |
| A_23_P158053 | *C9orf16* | 7.75E-07 | 2.24 | up |
| A_24_P76521 | *GSG2* | 1.56E-06 | 2.23 | up |
| A_23_P36928 | *POLR1D* | 6.35E-07 | 2.23 | up |
| A_23_P50638 | *LRG1* | 3.23E-04 | 2.23 | up |
| A_24_P332482 |  | 2.79E-04 | 2.23 | up |
| A_23_P300728 | *LMBR1* | 1.24E-07 | 2.23 | up |
| A_23_P333420 | *RANGAP1* | 2.77E-06 | 2.23 | up |
| A_24_P301846 | *GART* | 1.65E-06 | 2.23 | up |
| A_23_P161338 | *PPA1* | 2.72E-09 | 2.23 | up |
| A_23_P40657 | *GCAT* | 9.67E-04 | 2.22 | up |
| A_23_P79259 | *SH3BP4* | 9.81E-06 | 2.22 | up |
| A_23_P7030 | *MRPL3* | 9.21E-06 | 2.22 | up |
| A_32_P2730 | *BCAN* | 1.50E-03 | 2.22 | up |
| A_23_P100344 | *ORC6L* | 3.24E-05 | 2.22 | up |
| A_24_P200427 | *PAICS* | 4.36E-06 | 2.22 | up |
| A_24_P222126 | *TH1L* | 4.29E-03 | 2.22 | up |
| A_32_P147241 | *PKM2* | 4.86E-06 | 2.21 | up |
| A_23_P51718 | *PABPC4* | 5.79E-05 | 2.21 | up |
| A_24_P817236 | *CHRM3* | 1.47E-04 | 2.21 | up |
| A_24_P392540 |  | 9.46E-03 | 2.21 | up |
| A_24_P375813 | *RPL7A* | 1.36E-04 | 2.21 | up |
| A_24_P57367 | *AHCY* | 5.14E-04 | 2.21 | up |
| A_24_P178643 |  | 7.18E-05 | 2.21 | up |
| A_23_P133174 | *TMEM165* | 4.14E-07 | 2.21 | up |
| A_23_P57667 | *PLXNA1* | 2.82E-07 | 2.21 | up |
| A_23_P140738 | *FAM38A* | 9.56E-06 | 2.21 | up |
| A_23_P32861 | *NMD3* | 1.78E-06 | 2.21 | up |
| A_23_P100469 | *TXNL4B* | 1.66E-08 | 2.21 | up |
| A_24_P12438 | *NCOA7* | 3.92E-03 | 2.21 | up |
| A_23_P367405 | *PCBD1* | 8.54E-09 | 2.21 | up |
| A_24_P341476 | *LOC729313* | 1.44E-03 | 2.21 | up |
| A_23_P216920 | *NEK6* | 1.75E-05 | 2.20 | up |
| A_23_P200940 | *PPIH* | 1.52E-03 | 2.20 | up |
| A_23_P143650 | *DUSP18* | 2.13E-06 | 2.20 | up |
| A_24_P516215 | *NOB1* | 3.19E-05 | 2.20 | up |
| A_23_P215735 | *ST7* | 3.80E-08 | 2.20 | up |
| A_24_P162373 | *ZNRF3* | 1.48E-03 | 2.20 | up |
| A_23_P158596 | *AGTRAP* | 1.43E-06 | 2.20 | up |
| A_23_P71146 | *POLD2* | 8.44E-06 | 2.20 | up |
| A_23_P93562 | *SESN1* | 1.75E-04 | 2.20 | up |
| A_24_P333445 | *MORF4L2* | 6.97E-04 | 2.20 | up |
| A_23_P160240 | *ACP6* | 6.17E-06 | 2.20 | up |
| A_23_P58819 | *RANBP17* | 8.77E-07 | 2.20 | up |
| A_32_P24585 | *SH3PXD2B* | 2.55E-05 | 2.20 | up |
| A_23_P86089 | *VWA1* | 8.93E-04 | 2.19 | up |
| A_23_P91328 | *NOP56* | 3.50E-05 | 2.19 | up |
| A_24_P381494 | *SLC11A2* | 4.76E-05 | 2.19 | up |
| A_24_P151920 | *TMEM97* | 5.71E-07 | 2.19 | up |
| A_23_P146584 | *C9orf30* | 6.15E-08 | 2.19 | up |
| A_24_P341427 |  | 9.87E-04 | 2.19 | up |
| A_23_P66473 | *PITPNC1* | 1.23E-05 | 2.19 | up |
| A_23_P2181 | *CYB5R2* | 1.35E-03 | 2.19 | up |
| A_23_P389118 | *ANO6* | 7.65E-03 | 2.18 | up |
| A_24_P409881 | *LOC338756* | 2.42E-05 | 2.18 | up |
| A_23_P215419 | *ICA1* | 1.86E-06 | 2.18 | up |
| A_24_P859431 |  | 3.63E-07 | 2.18 | up |
| A_23_P28927 |  | 2.69E-04 | 2.18 | up |
| A_24_P626931 | *UBE2CBP* | 1.09E-09 | 2.18 | up |
| A_23_P414978 | *NUDT14* | 6.26E-04 | 2.18 | up |
| A_23_P37704 | *CDT1* | 2.50E-05 | 2.18 | up |
| A_23_P18579 | *PTTG2* | 1.83E-04 | 2.17 | up |
| A_23_P92261 | *ECE2* | 1.70E-05 | 2.17 | up |
| A_23_P157333 | *EPHA1* | 2.24E-05 | 2.17 | up |
| A_23_P67529 | *KCNN4* | 8.75E-05 | 2.17 | up |
| A_23_P404667 | *BIK* | 2.35E-05 | 2.17 | up |
| A_23_P82478 | *PUS7* | 3.70E-06 | 2.17 | up |
| A_32_P169735 | *TTC8* | 8.85E-06 | 2.17 | up |
| A_23_P144465 | *PAPSS1* | 3.02E-06 | 2.17 | up |
| A_24_P41180 | *RPS5* | 5.57E-04 | 2.17 | up |
| A_23_P202837 | *CCND1* | 6.40E-07 | 2.17 | up |
| A_23_P152984 | *THOC4* | 5.15E-04 | 2.17 | up |
| A_32_P158272 | *MACC1* | 2.57E-04 | 2.17 | up |
| A_23_P65110 | *RACGAP1* | 3.65E-06 | 2.17 | up |
| A_23_P67708 | *TCF3* | 3.87E-07 | 2.16 | up |
| A_24_P365515 | *FOXA2* | 3.56E-04 | 2.16 | up |
| A_24_P761727 |  | 1.28E-04 | 2.16 | up |
| A_23_P133995 | *PPIL1* | 2.35E-08 | 2.16 | up |
| A_24_P110082 |  | 3.54E-04 | 2.16 | up |
| A_32_P201521 | *TMEM97* | 1.44E-05 | 2.16 | up |
| A_24_P33156 | *AFMID* | 8.68E-05 | 2.16 | up |
| A_23_P115482 | *UBE2T* | 1.72E-06 | 2.16 | up |
| A_32_P116813 | *LYRM4* | 6.02E-06 | 2.15 | up |
| A_23_P2814 | *SMAD9* | 1.42E-04 | 2.15 | up |
| A_23_P153086 | *C18orf22* | 1.67E-05 | 2.15 | up |
| A_23_P34788 | *KIF2C* | 8.42E-05 | 2.15 | up |
| A_24_P317135 | *EXOSC7* | 4.99E-09 | 2.15 | up |
| A_23_P135294 | *ALDH1B1* | 1.52E-03 | 2.15 | up |
| A_23_P340909 | *SKA3* | 1.05E-04 | 2.15 | up |
| A_23_P209978 | *VSNL1* | 7.50E-04 | 2.15 | up |
| A_24_P506977 | *C7orf40* | 4.64E-05 | 2.15 | up |
| A_23_P87591 | *YEATS4* | 8.42E-08 | 2.14 | up |
| A_24_P92183 | *PABPC1L* | 4.51E-03 | 2.14 | up |
| A_23_P385861 | *CDCA2* | 5.71E-05 | 2.14 | up |
| A_23_P143047 | *ATP6V1E2* | 1.83E-05 | 2.14 | up |
| A_24_P107208 | *CAPRIN1* | 1.27E-05 | 2.14 | up |
| A_23_P106998 | *MRPS23* | 7.08E-08 | 2.14 | up |
| A_23_P62959 | *PHLDA3* | 3.07E-04 | 2.14 | up |
| A_23_P124855 | *ZCCHC7* | 1.12E-04 | 2.14 | up |
| A_23_P46539 | *PSRC1* | 1.42E-05 | 2.13 | up |
| A_23_P57306 | *CHAF1B* | 9.70E-06 | 2.13 | up |
| A_23_P82169 | *SOX4* | 2.31E-05 | 2.13 | up |
| A_23_P61268 | *C8orf30A* | 2.38E-06 | 2.13 | up |
| A_32_P61061 | *PPIAL4A* | 1.88E-04 | 2.13 | up |
| A_24_P146575 | *SHB* | 4.05E-05 | 2.13 | up |
| A_23_P206899 | *TMEM159* | 1.93E-04 | 2.13 | up |
| A_24_P399680 | *C20orf108* | 6.17E-06 | 2.13 | up |
| A_24_P413437 | *NONO* | 1.08E-06 | 2.13 | up |
| A_23_P74349 | *NUF2* | 5.28E-05 | 2.13 | up |
| A_23_P145197 | *BYSL* | 9.44E-07 | 2.13 | up |
| A_23_P52610 | *DDB2* | 2.69E-05 | 2.12 | up |
| A_23_P32328 | *LYRM4* | 2.27E-06 | 2.12 | up |
| A_23_P24444 | *DHCR7* | 3.76E-06 | 2.12 | up |
| A_23_P359497 | *TMEM231* | 7.36E-06 | 2.12 | up |
| A_23_P363896 | *NCRNA00188* | 7.40E-05 | 2.12 | up |
| A_23_P31143 | *TPD52L1* | 2.17E-03 | 2.12 | up |
| A_23_P119789 | *TMEM185B* | 4.67E-07 | 2.12 | up |
| A_32_P170925 | *TXNRD3* | 6.08E-06 | 2.12 | up |
| A_24_P787889 | *TMEM191A* | 4.80E-04 | 2.12 | up |
| A_24_P254933 |  | 3.69E-04 | 2.12 | up |
| A_23_P1505 | *LRP5* | 1.14E-05 | 2.12 | up |
| A_23_P54605 | *RSL1D1* | 3.81E-05 | 2.11 | up |
| A_24_P289188 |  | 2.10E-04 | 2.11 | up |
| A_23_P137830 |  | 1.89E-03 | 2.11 | up |
| A_24_P89509 | *TRMT11* | 2.36E-05 | 2.11 | up |
| A_23_P62115 | *TIMP1* | 3.34E-04 | 2.11 | up |
| A_23_P71904 | *METTL11A* | 5.74E-07 | 2.11 | up |
| A_24_P266037 | *DIMT1L* | 3.64E-08 | 2.11 | up |
| A_32_P143245 | *C6orf173* | 8.84E-05 | 2.11 | up |
| A_23_P357207 | *MRAP2* | 3.82E-04 | 2.11 | up |
| A_24_P193011 | *CCND1* | 1.16E-04 | 2.10 | up |
| A_23_P71644 | *FANCG* | 1.33E-06 | 2.10 | up |
| A_23_P165636 | *CAPG* | 5.37E-06 | 2.10 | up |
| A_24_P314571 |  | 3.47E-04 | 2.10 | up |
| A_24_P218265 | *TNFRSF10B* | 4.60E-06 | 2.10 | up |
| A_24_P365523 | *FOXA2* | 8.49E-05 | 2.10 | up |
| A_23_P436484 |  | 4.82E-03 | 2.10 | up |
| A_24_P42517 | *KDELR2* | 3.59E-05 | 2.10 | up |
| A_23_P429977 | *KCNQ1* | 1.41E-05 | 2.10 | up |
| A_23_P336854 | *LOC648771* | 1.85E-06 | 2.10 | up |
| A_23_P162476 | *CDCA3* | 1.55E-05 | 2.10 | up |
| A_24_P340679 | *PPIA* | 4.22E-03 | 2.09 | up |
| A_23_P205216 | *UTP14A* | 9.84E-06 | 2.09 | up |
| A_23_P257924 | *ETS2* | 1.15E-04 | 2.09 | up |
| A_24_P211151 | *EXOSC5* | 1.38E-06 | 2.09 | up |
| A_24_P203056 | *BCL7A* | 1.33E-05 | 2.09 | up |
| A_24_P305312 | *BBC3* | 1.53E-06 | 2.09 | up |
| A_24_P682601 |  | 1.71E-06 | 2.09 | up |
| A_23_P156739 | *C6orf125* | 7.85E-06 | 2.09 | up |
| A_23_P134225 | *LRRC61* | 1.89E-04 | 2.09 | up |
| A_24_P16124 | *IFITM4P* | 3.39E-04 | 2.09 | up |
| A_23_P205489 | *SLC7A8* | 6.16E-05 | 2.09 | up |
| A_23_P358628 | *DPY19L1* | 8.83E-07 | 2.08 | up |
| A_24_P84711 |  | 1.16E-03 | 2.08 | up |
| A_23_P415511 | *C2orf15* | 4.57E-07 | 2.08 | up |
| A_24_P398585 | *UNG* | 1.00E-03 | 2.08 | up |
| A_32_P64919 | *DIAPH3* | 1.03E-04 | 2.08 | up |
| A_23_P353436 | *CEP78* | 1.57E-06 | 2.08 | up |
| A_23_P258340 | *PPIA* | 1.12E-04 | 2.08 | up |
| A_23_P135364 | *DTYMK* | 7.76E-07 | 2.08 | up |
| A_23_P28878 | *C20orf27* | 5.62E-06 | 2.08 | up |
| A_23_P257743 | *SHB* | 1.34E-04 | 2.08 | up |
| A_32_P24165 | *FANCD2* | 1.47E-05 | 2.08 | up |
| A_23_P53530 | *MTERFD3* | 3.15E-07 | 2.07 | up |
| A_23_P209778 | *POLR2D* | 3.76E-08 | 2.07 | up |
| A_23_P37892 | *GPT2* | 3.69E-04 | 2.07 | up |
| A_23_P54597 | *RSL1D1* | 1.52E-05 | 2.07 | up |
| A_23_P252783 | *SLC2A8* | 6.92E-05 | 2.07 | up |
| A_24_P246351 | *FAM71E1* | 4.65E-05 | 2.07 | up |
| A_23_P361405 | *HYAL3* | 8.00E-04 | 2.07 | up |
| A_24_P677783 | *LOC152024* | 3.31E-04 | 2.07 | up |
| A_23_P315286 | *C19orf22* | 1.41E-05 | 2.07 | up |
| A_23_P151405 | *CKAP2* | 3.17E-04 | 2.07 | up |
| A_23_P208706 | *BAX* | 8.06E-04 | 2.07 | up |
| A_32_P193646 | *RBMX* | 1.12E-08 | 2.07 | up |
| A_23_P74954 | *RCC2* | 1.24E-08 | 2.07 | up |
| A_23_P141126 | *GALK1* | 1.33E-05 | 2.07 | up |
| A_23_P254888 | *ZYX* | 5.54E-05 | 2.07 | up |
| A_23_P133123 | *MND1* | 7.06E-05 | 2.07 | up |
| A_23_P309224 | *AGK* | 7.53E-07 | 2.07 | up |
| A_23_P9465 | *FPGS* | 3.42E-07 | 2.06 | up |
| A_23_P115149 | *WDR77* | 1.34E-05 | 2.06 | up |
| A_23_P335428 | *HIBADH* | 1.82E-05 | 2.06 | up |
| A_23_P200843 | *CHRM3* | 7.67E-04 | 2.06 | up |
| A_23_P332326 | *ARHGEF19* | 2.29E-04 | 2.06 | up |
| A_23_P208265 | *RPS5* | 6.93E-04 | 2.06 | up |
| A_24_P83118 | *DUSP18* | 1.64E-06 | 2.06 | up |
| A_23_P166135 | *XRN2* | 1.08E-04 | 2.06 | up |
| A_23_P102183 |  | 2.65E-05 | 2.06 | up |
| A_23_P258964 | *IARS* | 1.34E-06 | 2.06 | up |
| A_23_P9894 | *PRMT3* | 3.47E-06 | 2.05 | up |
| A_23_P202964 | *TMEM123* | 3.63E-04 | 2.05 | up |
| A_23_P145584 | *UBE2H* | 1.46E-06 | 2.05 | up |
| A_23_P17393 | *CSE1L* | 2.51E-08 | 2.05 | up |
| A_23_P162970 | *IPO4* | 4.51E-05 | 2.05 | up |
| A_23_P115861 | *ZNF485* | 1.84E-08 | 2.05 | up |
| A_23_P42802 | *PDIA4* | 4.23E-06 | 2.05 | up |
| A_23_P2066 | *APIP* | 8.50E-05 | 2.05 | up |
| A_24_P273679 | *YAP1* | 3.31E-04 | 2.05 | up |
| A_23_P118246 | *GINS2* | 6.78E-04 | 2.05 | up |
| A_23_P165840 | *ODC1* | 1.64E-04 | 2.05 | up |
| A_23_P9458 | *POLR1E* | 1.61E-06 | 2.05 | up |
| A_23_P155815 | *NCAPG* | 9.74E-05 | 2.05 | up |
| A_23_P312646 | *C9orf142* | 2.48E-04 | 2.05 | up |
| A_23_P203391 | *ASRGL1* | 8.27E-04 | 2.05 | up |
| A_24_P148811 | *RUVBL1* | 1.48E-05 | 2.05 | up |
| A_23_P21436 | *PHF19* | 2.65E-05 | 2.04 | up |
| A_23_P130764 | *KCNJ14* | 6.66E-03 | 2.04 | up |
| A_24_P73669 | *GSPT1* | 2.41E-05 | 2.04 | up |
| A_24_P393838 | *TOMM20* | 5.32E-07 | 2.04 | up |
| A_23_P156996 | *tcag7.1196* | 1.97E-04 | 2.04 | up |
| A_23_P60899 | *TGS1* | 3.11E-05 | 2.04 | up |
| A_23_P717 | *TMEM206* | 8.15E-09 | 2.04 | up |
| A_24_P248053 | *TOP1MT* | 4.15E-06 | 2.04 | up |
| A_23_P46871 | *SLC29A3* | 1.17E-05 | 2.04 | up |
| A_23_P60271 | *SMC2* | 2.40E-05 | 2.04 | up |
| A_23_P344853 | *WDR43* | 4.26E-06 | 2.03 | up |
| A_24_P258073 | *C10orf2* | 3.43E-06 | 2.03 | up |
| A_24_P655849 | *SMAD9* | 1.13E-03 | 2.03 | up |
| A_24_P7330 |  | 2.15E-05 | 2.03 | up |
| A_23_P90612 | *MCM6* | 9.15E-06 | 2.03 | up |
| A_23_P119095 | *PPP1R13L* | 2.58E-04 | 2.03 | up |
| A_23_P251387 | *REPS2* | 5.27E-05 | 2.03 | up |
| A_23_P345212 | *BOD1P* | 3.29E-07 | 2.03 | up |
| A_23_P86855 | *MACROD1* | 2.87E-04 | 2.03 | up |
| A_24_P161973 | *ATP11A* | 6.15E-05 | 2.03 | up |
| A_24_P119567 | *CCDC52* | 3.71E-06 | 2.03 | up |
| A_23_P17575 | *AHCY* | 2.54E-08 | 2.03 | up |
| A_32_P54018 | *KIAA1143* | 1.58E-06 | 2.02 | up |
| A_23_P156970 | *MEST* | 9.11E-05 | 2.02 | up |
| A_32_P122754 | *C9orf30* | 8.29E-08 | 2.02 | up |
| A_32_P44274 | *CHTF18* | 5.43E-05 | 2.02 | up |
| A_24_P823684 | *HSP90AB1* | 2.18E-05 | 2.02 | up |
| A_23_P129014 | *C14orf143* | 8.37E-06 | 2.02 | up |
| A_23_P123974 | *DTYMK* | 6.99E-07 | 2.02 | up |
| A_23_P151506 | *PLEK2* | 4.53E-07 | 2.02 | up |
| A_23_P10305 | *CTPS2* | 8.79E-06 | 2.02 | up |
| A_23_P57588 | *GTSE1* | 3.57E-04 | 2.02 | up |
| A_32_P230720 | *E2F6* | 1.49E-10 | 2.02 | up |
| A_24_P176374 | *CDT1* | 1.97E-04 | 2.02 | up |
| A_23_P411922 | *PRPF40B* | 1.45E-05 | 2.02 | up |
| A_23_P333498 | *EEPD1* | 1.15E-04 | 2.01 | up |
| A_32_P162306 | *LOC440577* | 4.12E-03 | 2.01 | up |
| A_23_P50477 | *BCL2L12* | 2.00E-07 | 2.01 | up |
| A_23_P76882 | *CCNB1IP1* | 6.17E-05 | 2.01 | up |
| A_23_P31721 | *E2F5* | 9.05E-05 | 2.01 | up |
| A_23_P87902 | *DYRK4* | 2.71E-04 | 2.01 | up |
| A_24_P50890 | *PVRL1* | 2.03E-05 | 2.01 | up |
| A_23_P88740 | *CENPN* | 2.40E-05 | 2.01 | up |
| A_23_P156842 | *EEF1E1* | 7.86E-06 | 2.01 | up |
| A_23_P354297 | *CHTF18* | 7.28E-05 | 2.01 | up |
| A_32_P184933 | *UBE2S* | 2.74E-05 | 2.01 | up |
| A_23_P137031 | *EIF2S3* | 9.47E-06 | 2.01 | up |
| A_23_P253752 | *FAM54A* | 1.26E-04 | 2.01 | up |
| A_32_P113436 | *HNRNPA1L2* | 2.50E-06 | 2.01 | up |
| A_23_P8400 | *CDK5* | 9.55E-06 | 2.01 | up |
| A_23_P71014 | *WBSCR22* | 1.08E-06 | 2.00 | up |
| A_24_P212605 |  | 2.84E-03 | 2.00 | up |
| A_23_P107421 | *TK1* | 1.99E-04 | 2.00 | up |
| A_24_P64182 | *SLC2A8* | 5.59E-05 | 2.00 | up |
| A_23_P253029 | *BOK* | 1.53E-04 | 2.00 | up |
| A_23_P52058 | *TARBP1* | 1.51E-05 | 2.00 | up |
