# Supplementary material for: A Gene Expression and Pre-mRNA Splicing Signature That Marks the Adenoma-Adenocarcinoma Progression in Colorectal Cancer
Source: PLoS One. 2014 Feb 6;9(2):e87761. doi: 10.1371/journal.pone.0087761 (PMC3916340; doi:10.1371/journal.pone.0087761)
Supplement: Table S3 — Significantly up- and down-regulated genes in colorectal cancer samples in comparison to paired normal mucosae. (DOC) [file pone.0087761.s009.doc]

**Table S3.** **Significantly up- and down-regulated genes in colorectal cancer samples in comparison to paired normal mucosae.** The results of 44k Whole Human Genome microarrays (Agilent) for the deregulated genes in are presented (≥ 2.0 FC, P-value ≤ 0.01 by paired *t*-test with FDR).

| Probe Name | Gene Symbol | P-value | Fold-Change | Regulation |
| --- | --- | --- | --- | --- |
| A_23_P168916 | *CA1* | 1.05E-03 | 328.30 | down |
| A_23_P4096 | *CA4* | 2.50E-03 | 70.36 | down |
| A_23_P51217 | *CLCA1* | 3.51E-03 | 64.56 | down |
| A_23_P63032 | *GUCA2B* | 7.54E-03 | 57.01 | down |
| A_23_P11968 | *GUCA2A* | 1.37E-03 | 54.18 | down |
| A_32_P358887 | *SLC4A4* | 2.14E-03 | 50.33 | down |
| A_23_P251412 | *SCGN* | 6.52E-04 | 45.91 | down |
| A_23_P10127 | *SFRP1* | 4.66E-04 | 45.53 | down |
| A_23_P26522 | *AQP8* | 1.93E-03 | 44.01 | down |
| A_23_P102611 | *WISP2* | 5.51E-04 | 43.97 | down |
| A_23_P8913 | *CA2* | 8.72E-04 | 40.12 | down |
| A_23_P95790 | *ITLN1* | 6.23E-03 | 39.77 | down |
| A_23_P145529 | *PKIB* | 1.04E-03 | 39.74 | down |
| A_23_P94103 | *SCARA5* | 1.11E-03 | 36.56 | down |
| A_23_P58359 | *ADH1A* | 3.52E-03 | 35.39 | down |
| A_23_P81158 | *ADH1C* | 3.46E-03 | 34.56 | down |
| A_23_P501624 | *UGT2B17* | 3.22E-03 | 33.67 | down |
| A_23_P259863 | *CD177* | 2.54E-03 | 31.60 | down |
| A_24_P40626 | *GREM2* | 6.54E-04 | 31.20 | down |
| A_23_P58407 | *UGT2B15* | 1.20E-03 | 28.94 | down |
| A_24_P395415 |  | 1.60E-03 | 28.58 | down |
| A_24_P17691 | *UGT2B17* | 1.20E-03 | 28.26 | down |
| A_24_P291658 | *ADH1A* | 2.35E-03 | 25.89 | down |
| A_23_P97181 | *GREM2* | 5.37E-04 | 25.56 | down |
| A_23_P10121 | *SFRP1* | 4.66E-04 | 25.52 | down |
| A_32_P27046 | *CHGA* | 8.53E-04 | 25.01 | down |
| A_32_P157213 |  | 1.59E-03 | 24.04 | down |
| A_32_P143589 | *CD177* | 7.42E-03 | 24.04 | down |
| A_24_P300379 | *PI16* | 6.54E-04 | 22.45 | down |
| A_23_P110319 | *CWH43* | 4.97E-03 | 22.44 | down |
| A_23_P61042 | *IGHA2* | 3.43E-03 | 21.85 | down |
| A_23_P106656 | *CA7* | 3.01E-03 | 21.58 | down |
| A_23_P202448 | *CXCL12* | 6.52E-04 | 21.03 | down |
| A_23_P16225 | *BEST2* | 1.11E-03 | 20.25 | down |
| A_23_P259868 | *CD177* | 3.84E-03 | 19.98 | down |
| A_23_P36018 | *VSIG2* | 1.54E-03 | 19.80 | down |
| A_23_P18713 | *ABCG2* | 1.27E-03 | 19.31 | down |
| A_23_P250951 | *SLC26A2* | 4.61E-03 | 18.93 | down |
| A_23_P156826 | *C6orf105* | 1.86E-03 | 18.54 | down |
| A_23_P156708 | *TNXB* | 8.07E-04 | 18.23 | down |
| A_23_P320216 | *FAM55D* | 3.65E-03 | 17.45 | down |
| A_23_P56559 | *DHRS9* | 2.48E-03 | 16.64 | down |
| A_23_P140384 | *CTSG* | 2.84E-03 | 16.37 | down |
| A_23_P146274 | *STMN2* | 7.64E-04 | 16.14 | down |
| A_23_P212968 | *UGT2B11* | 2.26E-03 | 15.84 | down |
| A_23_P123228 | *SLC26A3* | 5.10E-03 | 15.74 | down |
| A_23_P4112 | *PYY* | 1.54E-03 | 15.44 | down |
| A_23_P37736 | *TNFRSF17* | 3.13E-03 | 15.18 | down |
| A_23_P17130 | *C2orf88* | 2.55E-03 | 15.13 | down |
| A_23_P51787 | *AMPD1* | 6.15E-03 | 15.06 | down |
| A_23_P407695 | *FAM151A* | 2.46E-03 | 14.61 | down |
| A_32_P78681 | *GLP2R* | 5.25E-04 | 14.59 | down |
| A_32_P118397 | *HEPACAM2* | 8.40E-03 | 14.22 | down |
| A_23_P18672 | *GBA3* | 1.38E-03 | 14.13 | down |
| A_23_P39067 | *SPIB* | 2.63E-03 | 13.84 | down |
| A_24_P263786 |  | 1.96E-03 | 13.77 | down |
| A_23_P256425 | *ADAMDEC1* | 1.20E-03 | 13.71 | down |
| A_32_P169353 |  | 6.54E-03 | 13.60 | down |
| A_23_P118065 | *HSD17B2* | 1.96E-03 | 13.56 | down |
| A_24_P20292 | *B3GNT7* | 7.16E-03 | 13.43 | down |
| A_24_P168925 | *CHRDL1* | 3.18E-03 | 13.18 | down |
| A_24_P164998 | *LOC646627* | 7.31E-03 | 13.02 | down |
| A_23_P257993 | *DNASE1L3* | 1.94E-03 | 12.77 | down |
| A_24_P234768 | *HTR4* | 7.01E-03 | 12.49 | down |
| A_23_P120902 | *LGALS2* | 3.91E-03 | 12.48 | down |
| A_23_P69497 | *CLEC3B* | 7.64E-04 | 12.34 | down |
| A_23_P105012 | *HRASLS2* | 1.40E-03 | 12.25 | down |
| A_23_P158297 | *BTNL3* | 8.21E-03 | 12.22 | down |
| A_23_P93641 | *AKR1B10* | 4.02E-03 | 12.21 | down |
| A_24_P129341 | *AKR1B10* | 3.95E-03 | 12.16 | down |
| A_23_P151895 | *CILP* | 1.78E-03 | 12.07 | down |
| A_23_P436284 | *OSTBETA* | 5.34E-03 | 11.90 | down |
| A_23_P136026 |  | 2.17E-03 | 11.75 | down |
| A_23_P130573 | *CEACAM7* | 2.79E-03 | 11.73 | down |
| A_24_P131622 | *FAM107A* | 6.65E-04 | 11.72 | down |
| A_23_P103812 | *FAM5C* | 7.27E-03 | 11.70 | down |
| A_23_P201747 | *PADI2* | 1.18E-03 | 11.51 | down |
| A_23_P15450 | *TMEM100* | 8.51E-04 | 11.32 | down |
| A_23_P54918 | *LDHD* | 1.20E-03 | 11.24 | down |
| A_23_P105144 | *SCUBE2* | 2.66E-03 | 11.14 | down |
| A_32_P11262 |  | 3.28E-03 | 11.06 | down |
| A_23_P212050 | *BCHE* | 6.54E-04 | 10.96 | down |
| A_23_P7342 | *UGT2B10* | 3.52E-03 | 10.95 | down |
| A_24_P270424 | *DPF3* | 6.52E-04 | 10.91 | down |
| A_23_P60599 | *UGT1A6* | 3.46E-03 | 10.89 | down |
| A_23_P163336 | *CA12* | 1.48E-03 | 10.88 | down |
| A_23_P17438 | *EDN3* | 1.96E-03 | 10.83 | down |
| A_23_P17134 | *MAL* | 5.25E-04 | 10.77 | down |
| A_23_P399265 | *STMN2* | 5.25E-04 | 10.64 | down |
| A_23_P19650 | *VIP* | 9.32E-03 | 10.60 | down |
| A_23_P356494 | *SPINK5* | 3.83E-03 | 10.59 | down |
| A_24_P152845 | *LOC340888* | 3.80E-03 | 10.59 | down |
| A_23_P164436 | *ASPA* | 1.49E-03 | 10.56 | down |
| A_23_P218858 | *ABI3BP* | 4.98E-03 | 10.31 | down |
| A_23_P96501 | *TEX11* | 2.48E-03 | 10.20 | down |
| A_23_P13713 | *PRPH* | 4.66E-04 | 9.94 | down |
| A_23_P8812 |  | 3.13E-03 | 9.86 | down |
| A_23_P114185 | *TSPAN7* | 5.21E-03 | 9.86 | down |
| A_23_P146134 | *DUSP26* | 9.62E-04 | 9.86 | down |
| A_32_P140139 | *F13A1* | 1.38E-03 | 9.67 | down |
| A_24_P364263 | *HRASLS2* | 1.86E-03 | 9.43 | down |
| A_23_P364625 | *LRRC19* | 6.01E-03 | 9.42 | down |
| A_23_P84860 | *FAM107A* | 4.66E-04 | 9.31 | down |
| A_23_P136753 |  | 1.41E-03 | 9.28 | down |
| A_23_P369994 | *DCLK1* | 2.24E-03 | 9.17 | down |
| A_23_P24543 | *FAM55A* | 8.19E-03 | 9.13 | down |
| A_23_P150457 | *LYVE1* | 3.52E-03 | 8.89 | down |
| A_32_P133072 | *SPON1* | 1.16E-03 | 8.88 | down |
| A_23_P119562 | *CFD* | 9.85E-04 | 8.88 | down |
| A_23_P167168 | *IGJ* | 3.42E-03 | 8.87 | down |
| A_23_P19723 | *BMP5* | 1.16E-03 | 8.65 | down |
| A_23_P39294 | *PLAC2* | 2.69E-03 | 8.59 | down |
| A_24_P300394 | *GSTA2* | 3.28E-03 | 8.56 | down |
| A_24_P325992 | *LIFR* | 8.53E-04 | 8.41 | down |
| A_32_P217140 | *ISX* | 4.80E-03 | 8.32 | down |
| A_24_P360674 | *CDKN2B* | 1.34E-03 | 8.25 | down |
| A_23_P312920 | *POU2AF1* | 6.24E-03 | 8.19 | down |
| A_32_P46594 | *LOC145837* | 2.95E-03 | 8.17 | down |
| A_23_P106773 | *SULT1A2* | 2.63E-03 | 8.08 | down |
| A_23_P26511 | *GDPD3* | 4.36E-03 | 7.98 | down |
| A_24_P709844 |  | 7.86E-04 | 7.98 | down |
| A_23_P61466 | *CD163L1* | 6.54E-04 | 7.96 | down |
| A_23_P90710 | *DES* | 9.73E-03 | 7.94 | down |
| A_32_P136295 | *GNG7* | 4.29E-03 | 7.92 | down |
| A_24_P538459 |  | 4.50E-03 | 7.77 | down |
| A_24_P177844 |  | 5.95E-03 | 7.75 | down |
| A_23_P66854 | *KRT20* | 4.12E-03 | 7.71 | down |
| A_23_P26854 | *RICH2* | 1.74E-03 | 7.65 | down |
| A_24_P592400 | *LOC553137* | 4.82E-03 | 7.65 | down |
| A_24_P417352 |  | 9.07E-03 | 7.63 | down |
| A_23_P166207 | *ABCC13* | 3.28E-03 | 7.61 | down |
| A_23_P392470 | *NR3C2* | 6.39E-03 | 7.53 | down |
| A_32_P48256 |  | 3.66E-03 | 7.44 | down |
| A_24_P16004 |  | 9.31E-03 | 7.42 | down |
| A_24_P643776 |  | 1.23E-03 | 7.40 | down |
| A_23_P136724 | *LOC344887* | 4.08E-03 | 7.31 | down |
| A_24_P222872 | *UGT1A6* | 7.56E-03 | 7.30 | down |
| A_23_P420442 | *SEMA6D* | 5.58E-03 | 7.29 | down |
| A_23_P10506 | *HPGDS* | 6.52E-04 | 7.26 | down |
| A_24_P352388 | *CDHR5* | 2.31E-03 | 7.21 | down |
| A_24_P524462 |  | 5.51E-04 | 7.16 | down |
| A_23_P415021 | *METTL7A* | 1.86E-03 | 7.12 | down |
| A_23_P253602 | *BMX* | 6.03E-03 | 7.09 | down |
| A_23_P72117 | *SMPDL3A* | 1.90E-03 | 7.07 | down |
| A_23_P14986 | *HSD11B2* | 1.16E-03 | 7.07 | down |
| A_23_P21907 |  | 1.07E-03 | 7.04 | down |
| A_24_P278603 | *MOGAT2* | 1.53E-03 | 7.00 | down |
| A_23_P134854 | *CLDN23* | 9.62E-04 | 6.99 | down |
| A_24_P243749 | *PDK4* | 4.18E-03 | 6.99 | down |
| A_23_P111583 | *CD36* | 3.01E-03 | 6.93 | down |
| A_23_P202683 | *CDHR5* | 1.77E-03 | 6.91 | down |
| A_24_P510357 | *LOC100293440* | 8.06E-03 | 6.89 | down |
| A_24_P116700 | *TMEM220* | 1.42E-03 | 6.87 | down |
| A_23_P17420 | *BCAS1* | 1.82E-03 | 6.87 | down |
| A_23_P95640 | *C1orf186* | 2.32E-03 | 6.82 | down |
| A_24_P169873 | *IGHA2* | 9.02E-03 | 6.82 | down |
| A_24_P70183 | *MYH11* | 2.25E-03 | 6.81 | down |
| A_24_P205604 | *PADI2* | 4.28E-03 | 6.78 | down |
| A_23_P158330 | *UGT1A8* | 1.12E-03 | 6.73 | down |
| A_23_P141505 | *CLEC10A* | 1.16E-03 | 6.72 | down |
| A_23_P8640 | *GPER* | 6.08E-03 | 6.69 | down |
| A_24_P938352 | *CPM* | 1.44E-03 | 6.66 | down |
| A_32_P76137 |  | 3.23E-03 | 6.65 | down |
| A_24_P220947 | *AKR1C1* | 7.64E-04 | 6.64 | down |
| A_23_P68669 | *CHODL* | 4.66E-04 | 6.62 | down |
| A_23_P74619 | *SELENBP1* | 4.69E-03 | 6.60 | down |
| A_23_P213050 | *HPGD* | 1.20E-03 | 6.55 | down |
| A_24_P384604 |  | 7.69E-03 | 6.53 | down |
| A_24_P315941 |  | 8.41E-03 | 6.52 | down |
| A_32_P310335 | *JAM2* | 3.84E-03 | 6.49 | down |
| A_24_P165864 | *P2RY14* | 5.37E-04 | 6.46 | down |
| A_23_P45324 | *TMEM35* | 1.16E-03 | 6.46 | down |
| A_23_P13907 | *IGF1* | 6.52E-04 | 6.46 | down |
| A_24_P409013 | *WDR78* | 6.08E-03 | 6.45 | down |
| A_23_P203698 | *MOGAT2* | 2.54E-03 | 6.40 | down |
| A_23_P397455 | *ACVR1C* | 9.46E-03 | 6.39 | down |
| A_24_P218814 | *RDH5* | 8.51E-04 | 6.34 | down |
| A_24_P15550 |  | 7.67E-03 | 6.31 | down |
| A_32_P120484 |  | 1.16E-03 | 6.28 | down |
| A_23_P121926 | *SEPP1* | 2.45E-03 | 6.27 | down |
| A_24_P1054 | *NFKBIL2* | 4.31E-03 | 6.24 | down |
| A_24_P72518 | *AHCYL2* | 2.94E-03 | 6.21 | down |
| A_24_P68908 | *LOC344887* | 8.39E-03 | 6.21 | down |
| A_23_P303833 | *SCN4B* | 8.51E-04 | 6.21 | down |
| A_23_P206920 | *MYH11* | 2.87E-03 | 6.19 | down |
| A_23_P98910 | *LRMP* | 1.47E-03 | 6.19 | down |
| A_23_P257971 | *AKR1C1* | 8.51E-04 | 6.18 | down |
| A_24_P941773 | *METTL7A* | 2.66E-03 | 6.14 | down |
| A_23_P107116 | *RNF112* | 3.17E-03 | 6.12 | down |
| A_23_P10980 | *LPHN3* | 8.01E-03 | 6.06 | down |
| A_23_P343104 | *FLJ30901* | 8.72E-04 | 6.03 | down |
| A_32_P106615 |  | 1.77E-03 | 6.02 | down |
| A_23_P153390 | *CLEC4G* | 4.43E-03 | 6.01 | down |
| A_23_P86021 | *SELENBP1* | 4.50E-03 | 6.00 | down |
| A_23_P4773 | *LILRB5* | 4.66E-04 | 6.00 | down |
| A_32_P194423 |  | 6.28E-03 | 5.98 | down |
| A_32_P156851 | *RCAN2* | 7.64E-04 | 5.95 | down |
| A_32_P53524 | *NTN1* | 9.00E-04 | 5.90 | down |
| A_24_P913716 | *B3GNT7* | 5.58E-03 | 5.89 | down |
| A_24_P304419 | *IGF1* | 1.77E-03 | 5.89 | down |
| A_23_P27005 | *DHRS11* | 2.11E-03 | 5.89 | down |
| A_23_P112452 | *GGTA1* | 7.64E-04 | 5.88 | down |
| A_23_P342138 | *ADAMTSL1* | 6.54E-04 | 5.88 | down |
| A_23_P54770 | *APOB48R* | 2.25E-03 | 5.86 | down |
| A_24_P379820 | *ITM2C* | 2.84E-03 | 5.85 | down |
| A_23_P4069 | *TMEM220* | 1.27E-03 | 5.83 | down |
| A_23_P66637 | *SGCA* | 6.54E-04 | 5.82 | down |
| A_23_P31798 | *NAT2* | 1.01E-03 | 5.82 | down |
| A_24_P318990 |  | 5.58E-03 | 5.77 | down |
| A_24_P204574 |  | 6.09E-03 | 5.76 | down |
| A_32_P227930 | *C5orf52* | 2.32E-03 | 5.76 | down |
| A_32_P94 | *LHFPL4* | 1.18E-03 | 5.75 | down |
| A_23_P86461 | *PLAC9* | 3.52E-03 | 5.73 | down |
| A_24_P330518 | *CA12* | 2.54E-03 | 5.72 | down |
| A_23_P84596 | *MGC29506* | 8.21E-03 | 5.71 | down |
| A_23_P156890 | *TCF21* | 3.57E-03 | 5.71 | down |
| A_23_P88767 | *PLA2G10* | 6.28E-03 | 5.70 | down |
| A_23_P140190 | *KIAA0125* | 6.54E-03 | 5.70 | down |
| A_24_P56240 | *CPNE8* | 4.87E-03 | 5.69 | down |
| A_24_P626951 |  | 5.68E-03 | 5.69 | down |
| A_23_P112957 |  | 7.48E-03 | 5.66 | down |
| A_23_P352266 | *BCL2* | 9.56E-04 | 5.62 | down |
| A_24_P402690 | *ITM2C* | 5.51E-03 | 5.62 | down |
| A_23_P170830 |  | 5.01E-03 | 5.61 | down |
| A_32_P148122 |  | 5.64E-03 | 5.60 | down |
| A_23_P69531 | *KLB* | 1.72E-03 | 5.60 | down |
| A_23_P149998 | *PBLD* | 9.07E-03 | 5.58 | down |
| A_23_P218247 | *CES3* | 2.85E-03 | 5.58 | down |
| A_23_P21092 | *CALB2* | 4.56E-03 | 5.56 | down |
| A_23_P21260 |  | 6.69E-03 | 5.56 | down |
| A_32_P722809 |  | 9.41E-03 | 5.51 | down |
| A_23_P52425 | *NKX2-3* | 2.76E-03 | 5.49 | down |
| A_24_P169713 |  | 7.30E-03 | 5.48 | down |
| A_23_P102160 | *FAM82A1* | 8.81E-04 | 5.46 | down |
| A_32_P30075 |  | 7.44E-03 | 5.45 | down |
| A_23_P65629 | *KCNK10* | 3.79E-03 | 5.42 | down |
| A_23_P162668 | *CPM* | 1.96E-03 | 5.41 | down |
| A_23_P35617 | *PLCE1* | 4.44E-03 | 5.40 | down |
| A_24_P379413 | *IL6R* | 5.25E-04 | 5.39 | down |
| A_23_P144348 | *SLIT2* | 1.11E-03 | 5.37 | down |
| A_23_P200741 | *DPT* | 6.92E-03 | 5.36 | down |
| A_24_P472081 |  | 6.70E-03 | 5.34 | down |
| A_23_P1904 | *MS4A2* | 8.54E-03 | 5.33 | down |
| A_23_P96383 | *SRPX* | 2.64E-03 | 5.33 | down |
| A_23_P372234 | *CA12* | 2.88E-03 | 5.32 | down |
| A_24_P112395 | *PBLD* | 7.65E-03 | 5.31 | down |
| A_24_P187970 | *PADI2* | 4.17E-03 | 5.31 | down |
| A_23_P44335 | *ENTPD8* | 7.14E-03 | 5.30 | down |
| A_23_P303087 | *PTN* | 3.65E-03 | 5.29 | down |
| A_23_P85008 | *MAOB* | 2.67E-03 | 5.26 | down |
| A_23_P84084 | *GFRA2* | 6.63E-03 | 5.24 | down |
| A_24_P361816 |  | 7.47E-03 | 5.22 | down |
| A_23_P131899 | *SDCBP2* | 4.33E-03 | 5.21 | down |
| A_23_P67799 | *TMEM37* | 5.48E-03 | 5.20 | down |
| A_23_P310094 | *SYNPO2* | 3.84E-03 | 5.20 | down |
| A_23_P26024 | *C15orf48* | 9.69E-03 | 5.19 | down |
| A_23_P418234 | *PHLPP2* | 1.01E-03 | 5.19 | down |
| A_23_P58588 | *SLIT3* | 8.11E-04 | 5.19 | down |
| A_32_P515920 | *LOC400573* | 1.24E-03 | 5.17 | down |
| A_24_P124349 | *PDGFD* | 1.20E-03 | 5.15 | down |
| A_23_P65022 | *ACADS* | 1.38E-03 | 5.15 | down |
| A_23_P103765 | *FCER1A* | 4.45E-03 | 5.15 | down |
| A_23_P80739 | *PLCD1* | 8.53E-04 | 5.13 | down |
| A_24_P605563 | *IGL@* | 7.23E-03 | 5.12 | down |
| A_24_P357465 | *TP53INP2* | 1.18E-03 | 5.05 | down |
| A_24_P204374 |  | 3.09E-03 | 5.04 | down |
| A_23_P217326 | *FHL1* | 8.54E-04 | 5.04 | down |
| A_32_P167631 |  | 4.67E-03 | 5.04 | down |
| A_23_P114689 | *ASAP3* | 7.64E-04 | 5.03 | down |
| A_23_P210158 |  | 7.64E-04 | 5.03 | down |
| A_23_P154358 | *PROM2* | 4.59E-03 | 5.02 | down |
| A_23_P69810 | *AGPAT9* | 9.73E-03 | 5.02 | down |
| A_23_P92161 | *ARL14* | 6.74E-03 | 5.02 | down |
| A_23_P119006 |  | 6.74E-03 | 4.99 | down |
| A_24_P71904 | *HPGD* | 5.67E-03 | 4.99 | down |
| A_23_P159163 |  | 8.84E-03 | 4.99 | down |
| A_24_P548866 | *HIGD1A* | 4.66E-04 | 4.98 | down |
| A_23_P47709 | *FOLR2* | 1.70E-03 | 4.97 | down |
| A_32_P46214 | *SLC9A9* | 8.53E-04 | 4.96 | down |
| A_23_P372848 | *P2RX1* | 3.01E-03 | 4.94 | down |
| A_23_P372308 | *RGMA* | 3.31E-03 | 4.94 | down |
| A_23_P41528 | *FGFBP2* | 6.76E-03 | 4.94 | down |
| A_24_P131392 | *FAM82A1* | 1.00E-03 | 4.92 | down |
| A_24_P870620 | *PTN* | 3.18E-03 | 4.90 | down |
| A_23_P205370 | *ASB2* | 1.48E-03 | 4.87 | down |
| A_24_P925505 | *CD36* | 3.84E-03 | 4.86 | down |
| A_32_P171225 |  | 1.45E-03 | 4.85 | down |
| A_23_P96271 | *MYOM1* | 3.23E-03 | 4.84 | down |
| A_23_P160433 | *C1orf115* | 4.35E-03 | 4.83 | down |
| A_23_P56868 |  | 1.16E-03 | 4.82 | down |
| A_24_P12521 | *C7orf31* | 3.38E-03 | 4.76 | down |
| A_23_P233 | *FMO5* | 2.92E-03 | 4.73 | down |
| A_23_P55738 | *CEACAM1* | 8.93E-03 | 4.72 | down |
| A_23_P110569 | *TRIM36* | 5.45E-03 | 4.72 | down |
| A_23_P434118 | *CEACAM1* | 9.48E-03 | 4.70 | down |
| A_23_P256470 | *NPY* | 1.34E-03 | 4.69 | down |
| A_23_P28906 |  | 5.88E-03 | 4.66 | down |
| A_23_P32233 | *KLF4* | 1.48E-03 | 4.65 | down |
| A_23_P360804 | *CPNE5* | 2.52E-03 | 4.64 | down |
| A_23_P80594 | *PLCL2* | 7.02E-03 | 4.60 | down |
| A_23_P159435 |  | 7.35E-03 | 4.58 | down |
| A_23_P110253 | *KIT* | 4.98E-03 | 4.57 | down |
| A_24_P156490 | *KCNMA1* | 9.10E-03 | 4.56 | down |
| A_24_P90881 | *CES3* | 9.95E-04 | 4.55 | down |
| A_23_P104741 | *KIRREL3* | 2.10E-03 | 4.55 | down |
| A_23_P66767 | *GGT6* | 6.06E-03 | 4.54 | down |
| A_23_P41987 | *GFRA3* | 1.12E-03 | 4.53 | down |
| A_24_P272310 | *MUSTN1* | 2.26E-03 | 4.52 | down |
| A_23_P311895 | *CLIC5* | 1.86E-03 | 4.51 | down |
| A_32_P118942 |  | 8.93E-03 | 4.50 | down |
| A_24_P62708 | *PRKACB* | 9.25E-03 | 4.50 | down |
| A_24_P648880 | *MEIS3P1* | 1.48E-03 | 4.49 | down |
| A_24_P163237 | *STOX2* | 1.48E-03 | 4.49 | down |
| A_23_P21758 | *ADAM28* | 7.23E-03 | 4.49 | down |
| A_23_P85240 | *TLR7* | 2.74E-03 | 4.47 | down |
| A_23_P416774 | *CLIC5* | 3.52E-03 | 4.46 | down |
| A_24_P131173 | *C1orf115* | 6.01E-03 | 4.46 | down |
| A_24_P353638 | *SLAMF7* | 8.20E-03 | 4.45 | down |
| A_23_P25187 | *KRT81* | 7.30E-03 | 4.44 | down |
| A_23_P113161 | *C1orf21* | 4.69E-03 | 4.43 | down |
| A_23_P218626 | *NEU4* | 7.95E-03 | 4.42 | down |
| A_32_P48279 |  | 2.94E-03 | 4.41 | down |
| A_23_P164057 | *MFAP4* | 4.45E-03 | 4.40 | down |
| A_32_P218025 |  | 7.41E-03 | 4.39 | down |
| A_24_P153568 | *MPEG1* | 8.53E-04 | 4.39 | down |
| A_24_P388433 | *PPP2R3A* | 2.25E-03 | 4.38 | down |
| A_23_P324340 | *DISP2* | 7.24E-03 | 4.37 | down |
| A_23_P257583 | *DENND2A* | 2.00E-03 | 4.35 | down |
| A_24_P937240 |  | 2.69E-03 | 4.35 | down |
| A_23_P32500 | *STAB1* | 9.62E-04 | 4.33 | down |
| A_23_P11629 | *TMEM61* | 9.72E-03 | 4.31 | down |
| A_23_P426305 | *AOC3* | 3.28E-03 | 4.31 | down |
| A_23_P4536 | *EPB41L3* | 1.36E-03 | 4.27 | down |
| A_23_P162288 | *MYO1A* | 6.76E-03 | 4.27 | down |
| A_23_P250102 | *CAND2* | 2.05E-03 | 4.27 | down |
| A_23_P171074 | *ITM2A* | 3.18E-03 | 4.27 | down |
| A_23_P117662 | *HDC* | 5.95E-03 | 4.27 | down |
| A_23_P10902 | *FRZB* | 9.99E-03 | 4.24 | down |
| A_23_P253321 | *PNOC* | 7.73E-03 | 4.24 | down |
| A_23_P41365 | *SMR3A* | 6.52E-03 | 4.23 | down |
| A_23_P219161 | *OLFM1* | 6.54E-04 | 4.22 | down |
| A_23_P79572 | *MGC16025* | 7.63E-03 | 4.21 | down |
| A_23_P135990 | *SLCO2A1* | 1.05E-03 | 4.21 | down |
| A_24_P68079 | *TRANK1* | 2.19E-03 | 4.19 | down |
| A_23_P386942 | *DIRAS1* | 9.58E-04 | 4.19 | down |
| A_23_P160167 | *TSPAN1* | 5.24E-03 | 4.19 | down |
| A_23_P212354 | *CCR2* | 4.50E-03 | 4.19 | down |
| A_24_P104407 | *SYNM* | 7.45E-03 | 4.18 | down |
| A_23_P71328 | *MATN2* | 7.63E-03 | 4.14 | down |
| A_23_P214281 | *PAQR8* | 8.51E-04 | 4.14 | down |
| A_23_P133474 | *GPX3* | 8.93E-03 | 4.13 | down |
| A_23_P218369 | *CCL14* | 3.79E-03 | 4.11 | down |
| A_23_P202334 | *FGFR2* | 6.12E-03 | 4.10 | down |
| A_24_P246891 | *NEU4* | 6.01E-03 | 4.09 | down |
| A_23_P163567 | *SMPD3* | 7.89E-03 | 4.08 | down |
| A_23_P434212 | *SULT1A1* | 2.87E-03 | 4.07 | down |
| A_23_P217319 | *FGF13* | 2.19E-03 | 4.04 | down |
| A_23_P67198 | *CPAMD8* | 7.04E-03 | 4.04 | down |
| A_23_P74575 | *CD1D* | 1.63E-03 | 4.03 | down |
| A_23_P252075 | *AHCYL2* | 4.08E-03 | 4.03 | down |
| A_32_P72611 |  | 1.92E-03 | 4.03 | down |
| A_32_P44878 | *ITIH5* | 9.40E-03 | 4.03 | down |
| A_23_P59452 | *ABP1* | 2.35E-03 | 4.02 | down |
| A_23_P103256 | *CFHR3* | 5.95E-03 | 4.02 | down |
| A_23_P110624 | *CTNND2* | 2.85E-03 | 4.02 | down |
| A_23_P85441 | *IGSF9* | 1.59E-03 | 4.01 | down |
| A_24_P305345 | *CD209* | 1.05E-03 | 4.01 | down |
| A_23_P29096 | *PDE9A* | 4.83E-03 | 4.01 | down |
| A_23_P143935 | *PIGZ* | 4.68E-03 | 4.01 | down |
| A_23_P83857 | *MAOA* | 8.22E-03 | 3.98 | down |
| A_23_P21485 | *PID1* | 7.84E-03 | 3.96 | down |
| A_23_P5536 | *SLC9A2* | 1.48E-03 | 3.95 | down |
| A_24_P174755 | *SLC22A5* | 1.00E-03 | 3.93 | down |
| A_23_P145024 | *ADRB2* | 5.11E-03 | 3.93 | down |
| A_24_P105933 | *VIPR1* | 3.13E-03 | 3.93 | down |
| A_32_P37592 | *SCARNA17* | 5.86E-03 | 3.93 | down |
| A_23_P135123 |  | 7.50E-03 | 3.92 | down |
| A_23_P104804 | *ZBTB16* | 9.56E-04 | 3.91 | down |
| A_23_P414654 | *RAB37* | 3.36E-03 | 3.91 | down |
| A_23_P48088 | *CD27* | 3.83E-03 | 3.91 | down |
| A_23_P53588 | *WNT5B* | 2.11E-03 | 3.91 | down |
| A_23_P200160 | *CFH* | 3.43E-03 | 3.90 | down |
| A_24_P11825 | *CCR2* | 9.02E-03 | 3.90 | down |
| A_23_P153897 | *GNG7* | 3.55E-03 | 3.90 | down |
| A_32_P44210 |  | 7.94E-03 | 3.89 | down |
| A_32_P48536 |  | 1.05E-03 | 3.89 | down |
| A_23_P338919 | *SPEG* | 1.72E-03 | 3.89 | down |
| A_24_P503710 | *TLCD2* | 1.53E-03 | 3.89 | down |
| A_23_P258190 | *AKR1B1* | 3.36E-03 | 3.89 | down |
| A_23_P19624 | *BMP6* | 2.53E-03 | 3.88 | down |
| A_24_P67096 | *ABCA5* | 8.36E-03 | 3.87 | down |
| A_32_P218707 |  | 5.35E-03 | 3.86 | down |
| A_23_P17481 | *SIGLEC1* | 8.19E-03 | 3.83 | down |
| A_23_P94434 | *HRCT1* | 9.44E-03 | 3.83 | down |
| A_23_P335495 | *ANO7* | 2.70E-03 | 3.83 | down |
| A_24_P650482 | *LOC400960* | 1.77E-03 | 3.83 | down |
| A_23_P8834 | *EPHX2* | 7.01E-03 | 3.83 | down |
| A_23_P106675 | *PLCG2* | 2.34E-03 | 3.83 | down |
| A_24_P80204 | *MALL* | 6.88E-03 | 3.82 | down |
| A_24_P32935 | *FOLR2* | 1.18E-03 | 3.81 | down |
| A_32_P110390 | *TMEM171* | 1.54E-03 | 3.81 | down |
| A_23_P12363 | *ROR1* | 8.50E-03 | 3.79 | down |
| A_24_P373174 | *RAB27A* | 4.18E-03 | 3.78 | down |
| A_23_P117580 | *ENTPD5* | 5.08E-03 | 3.78 | down |
| A_23_P95619 | *GLOD5* | 2.88E-03 | 3.78 | down |
| A_23_P42969 | *FGL2* | 1.05E-03 | 3.78 | down |
| A_23_P163251 | *PAQR5* | 2.16E-03 | 3.76 | down |
| A_24_P901986 | *LOC100133019* | 1.86E-03 | 3.75 | down |
| A_32_P9127 |  | 7.50E-03 | 3.73 | down |
| A_23_P70060 | *PPAP2A* | 1.34E-03 | 3.72 | down |
| A_32_P132194 |  | 7.42E-03 | 3.70 | down |
| A_23_P94319 | *KBTBD11* | 6.00E-03 | 3.70 | down |
| A_23_P120667 | *JAM2* | 4.78E-03 | 3.70 | down |
| A_23_P337658 | *ALPI* | 1.17E-03 | 3.69 | down |
| A_32_P74477 |  | 3.22E-03 | 3.67 | down |
| A_23_P201808 | *PPAP2B* | 9.62E-04 | 3.66 | down |
| A_23_P19673 | *SGK1* | 5.02E-03 | 3.65 | down |
| A_24_P252739 | *KLF6* | 1.92E-03 | 3.65 | down |
| A_23_P7684 | *CCNJL* | 2.69E-03 | 3.65 | down |
| A_23_P143331 | *BMP2* | 4.30E-03 | 3.65 | down |
| A_24_P382319 | *CEACAM1* | 6.36E-03 | 3.65 | down |
| A_23_P213745 | *CXCL14* | 9.25E-03 | 3.64 | down |
| A_23_P257164 | *AMT* | 6.18E-03 | 3.63 | down |
| A_23_P167129 | *HHIP* | 4.33E-03 | 3.63 | down |
| A_23_P78018 | *ABCA5* | 4.84E-03 | 3.63 | down |
| A_23_P213857 | *C7* | 8.09E-03 | 3.62 | down |
| A_23_P204885 | *PCDH20* | 6.18E-03 | 3.62 | down |
| A_23_P209232 | *CLIP4* | 2.46E-03 | 3.62 | down |
| A_24_P646168 |  | 1.96E-03 | 3.62 | down |
| A_23_P4551 | *SETBP1* | 6.54E-04 | 3.60 | down |
| A_23_P209700 | *NMUR1* | 8.25E-03 | 3.60 | down |
| A_23_P24616 | *SIAE* | 5.72E-03 | 3.60 | down |
| A_23_P129312 | *PPP1R14D* | 9.58E-03 | 3.59 | down |
| A_32_P91273 |  | 2.32E-03 | 3.58 | down |
| A_23_P344531 | *SYNPO* | 3.22E-03 | 3.58 | down |
| A_24_P244800 | *NDRG2* | 4.78E-03 | 3.57 | down |
| A_23_P371107 | *D4S234E* | 4.03E-03 | 3.56 | down |
| A_23_P37205 | *NDRG2* | 6.78E-03 | 3.56 | down |
| A_24_P460763 |  | 6.73E-03 | 3.55 | down |
| A_23_P66017 | *PRRT2* | 2.80E-03 | 3.54 | down |
| A_23_P160226 | *C1orf175* | 1.22E-03 | 3.54 | down |
| A_23_P401084 | *ZNF575* | 5.19E-03 | 3.53 | down |
| A_32_P197561 | *EBF1* | 3.25E-03 | 3.53 | down |
| A_23_P110957 | *FOXF2* | 5.46E-03 | 3.53 | down |
| A_24_P917492 | *TTLL3* | 3.56E-03 | 3.52 | down |
| A_23_P206585 | *PRKCB* | 7.60E-03 | 3.52 | down |
| A_32_P150876 | *LOC339290* | 3.18E-03 | 3.52 | down |
| A_23_P91910 | *PLSCR4* | 6.85E-03 | 3.52 | down |
| A_32_P120791 | *LOC727916* | 3.32E-03 | 3.51 | down |
| A_32_P50655 |  | 1.07E-03 | 3.50 | down |
| A_23_P7882 | *SLC22A23* | 3.02E-03 | 3.50 | down |
| A_23_P130974 | *KIAA1683* | 4.29E-03 | 3.50 | down |
| A_23_P10753 |  | 4.33E-03 | 3.49 | down |
| A_23_P120103 | *KCNS3* | 4.82E-03 | 3.49 | down |
| A_32_P228618 | *RBMS3* | 3.15E-03 | 3.49 | down |
| A_23_P253012 | *GRAMD1C* | 6.09E-03 | 3.49 | down |
| A_23_P141367 | *CCR10* | 9.95E-04 | 3.46 | down |
| A_24_P71341 | *FMO5* | 3.17E-03 | 3.45 | down |
| A_24_P40551 | *BEX4* | 1.34E-03 | 3.45 | down |
| A_23_P10442 | *OSBPL1A* | 5.20E-03 | 3.44 | down |
| A_24_P233786 | *FAM129A* | 2.87E-03 | 3.44 | down |
| A_23_P101642 | *PTPRH* | 6.03E-03 | 3.44 | down |
| A_24_P32085 | *MOBKL2B* | 6.77E-03 | 3.44 | down |
| A_24_P930088 |  | 8.90E-03 | 3.44 | down |
| A_32_P123527 |  | 1.92E-03 | 3.44 | down |
| A_32_P76602 |  | 8.14E-03 | 3.43 | down |
| A_23_P422071 | *B3GALT4* | 1.19E-03 | 3.42 | down |
| A_32_P184888 |  | 1.86E-03 | 3.41 | down |
| A_24_P637651 |  | 1.38E-03 | 3.40 | down |
| A_23_P433016 | *FBLN1* | 5.53E-03 | 3.40 | down |
| A_23_P85015 | *MAOB* | 9.07E-03 | 3.39 | down |
| A_23_P95594 | *NAT1* | 3.79E-03 | 3.38 | down |
| A_24_P354488 | *NAAA* | 3.61E-03 | 3.38 | down |
| A_24_P102865 | *TMEM61* | 5.05E-03 | 3.37 | down |
| A_23_P61057 | *IL16* | 8.30E-03 | 3.37 | down |
| A_23_P258862 | *PDCD4* | 6.54E-04 | 3.36 | down |
| A_32_P146844 |  | 7.22E-03 | 3.36 | down |
| A_24_P64167 | *PTGS1* | 3.56E-03 | 3.35 | down |
| A_23_P58082 | *CCDC80* | 5.62E-03 | 3.35 | down |
| A_24_P227971 | *TP53TG3* | 1.27E-03 | 3.34 | down |
| A_23_P312840 | *SEMA6A* | 9.02E-03 | 3.34 | down |
| A_24_P363583 | *AGFG2* | 4.36E-03 | 3.34 | down |
| A_32_P32413 | *SETBP1* | 5.58E-03 | 3.33 | down |
| A_24_P174503 | *AMT* | 5.34E-03 | 3.33 | down |
| A_23_P363647 | *DDX26B* | 4.12E-03 | 3.32 | down |
| A_23_P45365 | *COL4A5* | 6.01E-03 | 3.32 | down |
| A_23_P2283 | *TAC3* | 1.54E-03 | 3.32 | down |
| A_23_P120125 | *COLEC11* | 7.64E-04 | 3.31 | down |
| A_23_P115726 | *SLC16A9* | 8.83E-03 | 3.31 | down |
| A_23_P111171 | *B3GALT4* | 1.05E-03 | 3.31 | down |
| A_24_P29665 | *CYCS* | 9.58E-03 | 3.30 | down |
| A_32_P121674 |  | 3.59E-03 | 3.30 | down |
| A_32_P167471 | *CLMN* | 6.47E-03 | 3.30 | down |
| A_24_P940166 | *PAPSS2* | 1.12E-03 | 3.30 | down |
| A_23_P142560 | *ZEB2* | 4.14E-03 | 3.30 | down |
| A_23_P97606 | *GSTM5* | 8.72E-04 | 3.29 | down |
| A_23_P11543 | *FUCA1* | 3.26E-03 | 3.29 | down |
| A_24_P372643 | *SLC22A18AS* | 9.56E-04 | 3.29 | down |
| A_32_P14737 |  | 2.11E-03 | 3.28 | down |
| A_24_P319374 | *GPA33* | 8.41E-03 | 3.28 | down |
| A_24_P324783 | *ACVRL1* | 2.19E-03 | 3.28 | down |
| A_32_P52785 | *DAAM2* | 2.95E-03 | 3.27 | down |
| A_23_P99275 | *KLRB1* | 4.09E-03 | 3.27 | down |
| A_23_P23279 | *RCSD1* | 4.65E-03 | 3.27 | down |
| A_24_P491923 |  | 7.94E-03 | 3.26 | down |
| A_24_P934135 |  | 7.35E-03 | 3.26 | down |
| A_24_P928522 | *DST* | 1.54E-03 | 3.25 | down |
| A_23_P408996 | *MBOAT1* | 4.81E-03 | 3.25 | down |
| A_24_P282108 | *ZZEF1* | 1.34E-03 | 3.24 | down |
| A_24_P623734 | *C2orf72* | 8.54E-03 | 3.24 | down |
| A_23_P203488 | *SMPD1* | 1.18E-03 | 3.24 | down |
| A_23_P131060 | *CYP4F8* | 8.48E-03 | 3.24 | down |
| A_24_P930927 |  | 2.36E-03 | 3.23 | down |
| A_23_P156852 | *PECI* | 1.42E-03 | 3.22 | down |
| A_24_P262201 | *SULT1A4* | 4.17E-03 | 3.22 | down |
| A_24_P396702 | *CD302* | 1.53E-03 | 3.22 | down |
| A_23_P2041 | *MICALCL* | 2.87E-03 | 3.22 | down |
| A_23_P201940 | *LMOD1* | 1.12E-03 | 3.21 | down |
| A_24_P109432 | *NBEAL1* | 6.26E-03 | 3.21 | down |
| A_24_P944154 | *MCTP2* | 8.46E-03 | 3.21 | down |
| A_23_P414913 | *GLIPR2* | 7.16E-03 | 3.20 | down |
| A_23_P216361 | *COL14A1* | 1.58E-03 | 3.20 | down |
| A_23_P211631 | *FBLN1* | 7.13E-03 | 3.20 | down |
| A_24_P945113 | *ACVRL1* | 5.43E-03 | 3.19 | down |
| A_23_P48585 | *SALL2* | 4.42E-03 | 3.19 | down |
| A_23_P87853 | *TMCC3* | 5.06E-03 | 3.18 | down |
| A_24_P296772 | *PPP1R14A* | 6.98E-03 | 3.18 | down |
| A_23_P163235 | *CKMT1A* | 9.23E-03 | 3.17 | down |
| A_24_P566916 | *NEAT1* | 9.56E-03 | 3.17 | down |
| A_23_P9932 | *PDCD4* | 9.68E-04 | 3.17 | down |
| A_23_P216596 | *SVEP1* | 9.56E-04 | 3.16 | down |
| A_23_P254917 | *LGALS4* | 3.80E-03 | 3.15 | down |
| A_24_P683905 | *HSBP1L1* | 4.64E-03 | 3.14 | down |
| A_23_P151529 | *C14orf132* | 4.99E-03 | 3.14 | down |
| A_32_P80809 |  | 2.18E-03 | 3.14 | down |
| A_24_P108311 | *NEDD4L* | 7.75E-03 | 3.14 | down |
| A_23_P97841 | *ITIH5* | 2.74E-03 | 3.13 | down |
| A_24_P153713 | *MARVELD3* | 3.43E-03 | 3.13 | down |
| A_23_P202269 | *ANK3* | 6.49E-03 | 3.12 | down |
| A_32_P227870 | *SLC30A4* | 4.92E-03 | 3.12 | down |
| A_23_P59718 | *SRI* | 1.38E-03 | 3.12 | down |
| A_24_P8116 | *CCDC80* | 9.41E-03 | 3.12 | down |
| A_24_P136484 |  | 2.41E-03 | 3.12 | down |
| A_24_P930741 | *EPHA10* | 6.64E-03 | 3.11 | down |
| A_32_P80597 | *ELOVL6* | 6.01E-03 | 3.11 | down |
| A_24_P69379 | *CASD1* | 2.11E-03 | 3.11 | down |
| A_24_P185117 | *RILP* | 1.13E-03 | 3.10 | down |
| A_23_P206310 | *KIAA0513* | 7.48E-03 | 3.10 | down |
| A_24_P254551 | *ARHGEF9* | 7.64E-04 | 3.08 | down |
| A_32_P115749 |  | 5.19E-03 | 3.08 | down |
| A_32_P45168 | *MEIS3P1* | 9.62E-04 | 3.08 | down |
| A_23_P143526 | *S100B* | 9.48E-03 | 3.06 | down |
| A_24_P132383 | *GIMAP8* | 5.60E-03 | 3.06 | down |
| A_23_P4494 | *DSC2* | 3.17E-03 | 3.06 | down |
| A_23_P257003 | *PCSK5* | 4.38E-03 | 3.05 | down |
| A_24_P192914 | *AMICA1* | 8.11E-04 | 3.05 | down |
| A_24_P497244 | *MALAT1* | 6.40E-03 | 3.05 | down |
| A_23_P415401 | *KLF9* | 4.36E-03 | 3.05 | down |
| A_23_P85140 | *TCEAL2* | 1.47E-03 | 3.05 | down |
| A_23_P10401 | *PPP2R3A* | 6.90E-03 | 3.04 | down |
| A_23_P26815 | *RILP* | 8.51E-04 | 3.04 | down |
| A_23_P53193 | *SYTL2* | 1.16E-03 | 3.04 | down |
| A_32_P46495 |  | 8.25E-03 | 3.04 | down |
| A_23_P123086 | *KIAA1908* | 2.31E-03 | 3.03 | down |
| A_24_P178723 | *LOC100132644* | 2.00E-03 | 3.01 | down |
| A_23_P113351 | *SPARCL1* | 6.48E-03 | 3.01 | down |
| A_23_P83277 | *IL11RA* | 1.16E-03 | 3.00 | down |
| A_23_P3221 | *SQRDL* | 2.51E-03 | 3.00 | down |
| A_32_P122715 |  | 3.95E-03 | 3.00 | down |
| A_23_P19095 | *SNX24* | 3.74E-03 | 3.00 | down |
| A_23_P163227 | *CKMT1A* | 8.57E-03 | 3.00 | down |
| A_23_P254165 | *RAI2* | 7.35E-03 | 2.99 | down |
| A_24_P459621 | *FLJ34515* | 2.50E-03 | 2.99 | down |
| A_24_P334640 | *PAQR8* | 6.65E-04 | 2.99 | down |
| A_23_P213959 | *PPARGC1B* | 3.74E-03 | 2.98 | down |
| A_23_P26865 | *MYH3* | 7.69E-03 | 2.98 | down |
| A_23_P259594 | *AKAP7* | 4.79E-03 | 2.97 | down |
| A_23_P50217 | *ZNF671* | 7.63E-03 | 2.97 | down |
| A_23_P5392 | *TP53I3* | 9.56E-04 | 2.97 | down |
| A_24_P239183 | *MUC4* | 9.67E-03 | 2.97 | down |
| A_23_P83028 | *RECK* | 3.51E-03 | 2.97 | down |
| A_32_P164477 |  | 3.67E-03 | 2.96 | down |
| A_24_P173823 | *PBX1* | 8.79E-03 | 2.96 | down |
| A_23_P401106 | *PDE2A* | 8.99E-03 | 2.96 | down |
| A_32_P208403 | *GNG2* | 1.53E-03 | 2.95 | down |
| A_23_P320739 | *MEF2C* | 9.34E-03 | 2.95 | down |
| A_24_P31275 | *ATP1B2* | 9.56E-04 | 2.94 | down |
| A_23_P116898 | *A2M* | 1.80E-03 | 2.93 | down |
| A_32_P49748 | *B4GALNT3* | 3.73E-03 | 2.93 | down |
| A_32_P207789 |  | 3.65E-03 | 2.93 | down |
| A_23_P77073 | *SPPL2A* | 2.31E-03 | 2.93 | down |
| A_24_P766716 | *CMKLR1* | 1.38E-03 | 2.92 | down |
| A_23_P254079 | *STBD1* | 2.43E-03 | 2.92 | down |
| A_23_P81441 | *C5orf20* | 2.14E-03 | 2.92 | down |
| A_32_P69956 |  | 8.53E-03 | 2.92 | down |
| A_23_P96590 | *GPRASP1* | 2.74E-03 | 2.91 | down |
| A_24_P782308 | *NEDD4L* | 4.88E-03 | 2.90 | down |
| A_23_P127911 | *PAMR1* | 2.13E-03 | 2.89 | down |
| A_23_P301530 | *ANK3* | 6.05E-03 | 2.89 | down |
| A_32_P147651 |  | 8.27E-03 | 2.89 | down |
| A_23_P6818 | *SEMA3G* | 1.20E-03 | 2.89 | down |
| A_23_P1759 | *AMICA1* | 1.25E-03 | 2.89 | down |
| A_23_P127727 | *MPEG1* | 6.52E-04 | 2.89 | down |
| A_23_P200685 | *MOSC2* | 2.85E-03 | 2.89 | down |
| A_23_P56197 | *CRLF1* | 8.88E-03 | 2.88 | down |
| A_32_P475513 | *MYO15B* | 9.07E-03 | 2.87 | down |
| A_23_P302914 | *ZFYVE28* | 2.16E-03 | 2.87 | down |
| A_32_P2452 | *TMTC1* | 1.12E-03 | 2.87 | down |
| A_23_P104493 | *PAPSS2* | 7.64E-04 | 2.87 | down |
| A_23_P51538 | *GPA33* | 4.88E-03 | 2.86 | down |
| A_24_P319088 | *CCL23* | 6.90E-03 | 2.86 | down |
| A_23_P71867 | *IL11RA* | 1.16E-03 | 2.86 | down |
| A_32_P213418 |  | 4.21E-03 | 2.86 | down |
| A_23_P73787 | *NUDT10* | 3.12E-03 | 2.86 | down |
| A_23_P300033 | *PDGFRA* | 6.64E-03 | 2.85 | down |
| A_32_P159289 |  | 8.87E-03 | 2.85 | down |
| A_32_P6139 | *C4orf19* | 2.99E-03 | 2.85 | down |
| A_24_P71373 | *SLC9A1* | 6.54E-04 | 2.85 | down |
| A_23_P72025 | *SLC25A20* | 6.35E-03 | 2.85 | down |
| A_32_P218785 |  | 8.10E-03 | 2.85 | down |
| A_23_P217528 | *KLF8* | 1.05E-03 | 2.85 | down |
| A_32_P42946 | *C1orf210* | 3.31E-03 | 2.84 | down |
| A_23_P167067 | *UGDH* | 2.42E-03 | 2.83 | down |
| A_24_P922261 | *SRGAP1* | 5.04E-03 | 2.83 | down |
| A_32_P214284 |  | 6.34E-03 | 2.82 | down |
| A_24_P920447 | *C14orf132* | 3.75E-03 | 2.82 | down |
| A_32_P174083 | *CYCS* | 4.30E-03 | 2.82 | down |
| A_23_P339818 | *ARRDC4* | 3.50E-03 | 2.81 | down |
| A_24_P305933 | *TMCC3* | 2.54E-03 | 2.81 | down |
| A_24_P101282 |  | 9.99E-03 | 2.81 | down |
| A_23_P101141 | *RNF125* | 3.63E-03 | 2.81 | down |
| A_23_P61447 | *ETFDH* | 5.86E-03 | 2.81 | down |
| A_32_P104746 | *ZFYVE28* | 3.14E-03 | 2.80 | down |
| A_23_P101380 | *B3GNT8* | 9.30E-03 | 2.80 | down |
| A_32_P211683 |  | 3.86E-03 | 2.80 | down |
| A_23_P309261 | *AKAP9* | 1.16E-03 | 2.80 | down |
| A_23_P42746 | *NCF1* | 6.05E-03 | 2.79 | down |
| A_23_P105747 | *APPL2* | 1.20E-03 | 2.79 | down |
| A_24_P943393 | *AHNAK* | 4.41E-03 | 2.79 | down |
| A_32_P226907 | *LOC284112* | 6.52E-04 | 2.78 | down |
| A_23_P500861 | *SYNE1* | 4.87E-03 | 2.78 | down |
| A_23_P146551 | *MOBKL2B* | 1.94E-03 | 2.77 | down |
| A_24_P106166 |  | 5.20E-03 | 2.77 | down |
| A_23_P168388 | *GIMAP8* | 5.47E-03 | 2.77 | down |
| A_23_P122007 | *C5orf30* | 3.65E-03 | 2.76 | down |
| A_32_P181107 |  | 2.38E-03 | 2.76 | down |
| A_23_P120644 |  | 8.11E-04 | 2.76 | down |
| A_23_P49610 | *C17orf91* | 2.13E-03 | 2.76 | down |
| A_32_P198071 |  | 2.38E-03 | 2.76 | down |
| A_23_P104624 | *ENDOD1* | 3.13E-03 | 2.76 | down |
| A_32_P82293 |  | 5.23E-03 | 2.75 | down |
| A_23_P318284 | *GPD1L* | 1.66E-03 | 2.75 | down |
| A_23_P155666 | *NAAA* | 7.76E-03 | 2.75 | down |
| A_23_P66948 | *FAM59A* | 6.11E-03 | 2.75 | down |
| A_23_P29684 | *VILL* | 8.69E-03 | 2.75 | down |
| A_32_P156564 |  | 5.02E-03 | 2.74 | down |
| A_23_P155335 | *PLD1* | 2.35E-03 | 2.73 | down |
| A_23_P144807 | *sept-08* | 8.51E-04 | 2.73 | down |
| A_23_P371495 | *TMTC1* | 1.94E-03 | 2.73 | down |
| A_23_P211039 | *ADAMTS1* | 5.49E-03 | 2.73 | down |
| A_32_P19101 |  | 8.70E-03 | 2.73 | down |
| A_24_P798431 |  | 7.59E-03 | 2.72 | down |
| A_24_P206776 | *CRYAB* | 2.93E-03 | 2.72 | down |
| A_23_P397376 | *MAF* | 9.07E-03 | 2.72 | down |
| A_23_P251316 | *CTNND1* | 1.92E-03 | 2.71 | down |
| A_23_P35564 | *SEC31B* | 6.47E-03 | 2.71 | down |
| A_23_P325924 | *FAM59B* | 8.54E-03 | 2.71 | down |
| A_24_P189533 | *ENDOD1* | 1.53E-03 | 2.71 | down |
| A_23_P41888 | *AGXT2L2* | 4.66E-04 | 2.70 | down |
| A_23_P110961 | *BRP44L* | 4.67E-03 | 2.70 | down |
| A_32_P103131 | *SOAT1* | 2.29E-03 | 2.69 | down |
| A_23_P16722 | *DOCK10* | 9.86E-03 | 2.69 | down |
| A_24_P943113 | *EIF4E3* | 8.02E-03 | 2.69 | down |
| A_23_P351757 | *PLCD3* | 9.89E-03 | 2.68 | down |
| A_23_P259090 | *NUDT12* | 2.00E-03 | 2.68 | down |
| A_23_P125233 | *CNN1* | 8.98E-03 | 2.68 | down |
| A_23_P433760 | *SPN* | 7.77E-03 | 2.68 | down |
| A_24_P208091 | *SLC35D1* | 1.86E-03 | 2.68 | down |
| A_23_P100111 | *CHP* | 4.63E-03 | 2.68 | down |
| A_23_P253345 | *C8orf4* | 6.25E-03 | 2.67 | down |
| A_32_P101313 | *PTPLAD2* | 1.38E-03 | 2.67 | down |
| A_23_P135548 | *DPYD* | 2.40E-03 | 2.67 | down |
| A_23_P12572 | *CASP7* | 7.07E-03 | 2.66 | down |
| A_23_P116512 | *PRR5L* | 1.79E-03 | 2.66 | down |
| A_23_P92903 | *C1QTNF2* | 4.72E-03 | 2.66 | down |
| A_32_P72758 | *MCTP2* | 5.58E-03 | 2.66 | down |
| A_23_P115417 | *RGL1* | 1.20E-03 | 2.66 | down |
| A_23_P217269 | *VSIG4* | 2.61E-03 | 2.65 | down |
| A_32_P130641 | *STARD9* | 3.46E-03 | 2.65 | down |
| A_23_P162386 | *BIN2* | 9.85E-04 | 2.65 | down |
| A_32_P194563 |  | 2.79E-03 | 2.65 | down |
| A_23_P501831 | *C5orf4* | 8.99E-03 | 2.65 | down |
| A_24_P200162 | *HIGD1A* | 9.05E-04 | 2.65 | down |
| A_23_P99747 | *CDKL1* | 5.51E-04 | 2.64 | down |
| A_23_P99853 | *KIAA1370* | 3.52E-03 | 2.64 | down |
| A_23_P167066 | *UGDH* | 1.00E-02 | 2.63 | down |
| A_32_P17484 |  | 5.98E-03 | 2.63 | down |
| A_23_P62133 | *MTM1* | 1.95E-03 | 2.63 | down |
| A_24_P205130 | *FNBP1* | 7.23E-03 | 2.62 | down |
| A_23_P152970 | *RAPGEFL1* | 6.16E-03 | 2.61 | down |
| A_24_P355246 | *PCSK5* | 6.46E-03 | 2.61 | down |
| A_23_P203376 | *MS4A6A* | 1.68E-03 | 2.61 | down |
| A_23_P30614 | *PLN* | 3.37E-03 | 2.61 | down |
| A_23_P14798 | *NEO1* | 4.17E-03 | 2.60 | down |
| A_23_P255884 | *GSN* | 2.35E-03 | 2.60 | down |
| A_23_P19142 | *KCNMB1* | 6.52E-03 | 2.60 | down |
| A_32_P73413 |  | 7.30E-03 | 2.60 | down |
| A_32_P180336 | *C11orf92* | 8.54E-03 | 2.59 | down |
| A_23_P387184 | *NHSL1* | 3.28E-03 | 2.59 | down |
| A_32_P224522 | *SLC25A23* | 2.87E-03 | 2.59 | down |
| A_23_P119923 | *CNNM4* | 4.57E-03 | 2.58 | down |
| A_32_P180825 | *FMN1* | 3.22E-03 | 2.58 | down |
| A_23_P394395 | *JPH2* | 4.97E-03 | 2.58 | down |
| A_23_P48455 | *AMN* | 4.89E-03 | 2.57 | down |
| A_23_P2831 | *EDNRB* | 3.08E-03 | 2.57 | down |
| A_24_P555510 | *PCM1* | 8.19E-03 | 2.57 | down |
| A_23_P151267 | *LIMA1* | 6.05E-03 | 2.57 | down |
| A_24_P396660 | *GSTM4* | 9.44E-03 | 2.57 | down |
| A_23_P103601 | *MAN1C1* | 8.54E-03 | 2.57 | down |
| A_24_P560519 | *PPARGC1B* | 1.94E-03 | 2.57 | down |
| A_23_P112801 | *CHP* | 3.90E-03 | 2.56 | down |
| A_23_P29975 | *C4orf19* | 1.85E-03 | 2.56 | down |
| A_23_P142239 | *YIF1B* | 7.69E-03 | 2.56 | down |
| A_24_P148503 | *FZD5* | 2.79E-03 | 2.56 | down |
| A_32_P216004 |  | 4.48E-03 | 2.56 | down |
| A_24_P366777 | *NOTCH2NL* | 6.63E-03 | 2.55 | down |
| A_32_P70420 |  | 7.99E-03 | 2.55 | down |
| A_24_P673968 | *TTC22* | 3.22E-03 | 2.55 | down |
| A_23_P146512 | *GOLM1* | 8.25E-03 | 2.55 | down |
| A_23_P110791 | *CSF1R* | 5.21E-03 | 2.54 | down |
| A_23_P110473 | *NAIP* | 5.36E-03 | 2.54 | down |
| A_23_P300714 | *SORBS3* | 6.76E-03 | 2.54 | down |
| A_23_P92899 | *C1QTNF2* | 3.01E-03 | 2.54 | down |
| A_23_P32249 | *FNBP1* | 2.13E-03 | 2.54 | down |
| A_23_P22444 | *CFP* | 3.50E-03 | 2.54 | down |
| A_23_P72697 | *GPIHBP1* | 9.29E-03 | 2.53 | down |
| A_32_P108722 | *LOC100288196* | 5.42E-03 | 2.53 | down |
| A_23_P118025 | *DPEP2* | 6.43E-03 | 2.53 | down |
| A_24_P357576 | *KIAA1370* | 8.86E-03 | 2.53 | down |
| A_24_P684183 | *SLC44A4* | 1.72E-03 | 2.52 | down |
| A_23_P106405 | *NDN* | 2.96E-03 | 2.51 | down |
| A_23_P93349 | *SLC44A4* | 1.70E-03 | 2.51 | down |
| A_24_P935103 | *ADCY9* | 5.72E-03 | 2.51 | down |
| A_24_P118171 | *MAGI3* | 2.25E-03 | 2.51 | down |
| A_23_P62128 | *MTM1* | 1.58E-03 | 2.51 | down |
| A_32_P109495 |  | 3.89E-03 | 2.51 | down |
| A_24_P196592 | *MMP28* | 5.97E-03 | 2.50 | down |
| A_24_P147169 | *PLA2G4F* | 5.02E-03 | 2.50 | down |
| A_32_P140656 | *IL6ST* | 2.49E-03 | 2.49 | down |
| A_23_P11201 | *GPR34* | 5.08E-03 | 2.49 | down |
| A_23_P13382 | *LSP1* | 8.50E-03 | 2.49 | down |
| A_23_P208450 | *SLC25A23* | 3.91E-03 | 2.49 | down |
| A_23_P328323 | *RAVER2* | 3.27E-03 | 2.48 | down |
| A_24_P924932 | *BCL2L15* | 7.35E-03 | 2.48 | down |
| A_23_P87379 | *PDE2A* | 9.41E-03 | 2.48 | down |
| A_23_P55578 | *RIOK3* | 3.17E-03 | 2.48 | down |
| A_24_P746314 |  | 8.52E-03 | 2.48 | down |
| A_23_P49816 | *ADAP2* | 1.59E-03 | 2.48 | down |
| A_23_P325690 | *ANKRD35* | 7.81E-03 | 2.48 | down |
| A_24_P294832 | *PTP4A1* | 1.54E-03 | 2.48 | down |
| A_23_P43455 | *PNPLA7* | 8.51E-04 | 2.47 | down |
| A_23_P127789 | *AHNAK* | 9.21E-03 | 2.47 | down |
| A_23_P52031 | *PGM1* | 3.39E-03 | 2.46 | down |
| A_23_P62768 | *TMEM54* | 4.34E-03 | 2.46 | down |
| A_23_P203173 | *IL10RA* | 8.63E-03 | 2.46 | down |
| A_23_P99141 | *GPR162* | 2.54E-03 | 2.46 | down |
| A_23_P83094 | *TLE4* | 5.92E-03 | 2.45 | down |
| A_23_P24077 | *C10orf54* | 1.36E-03 | 2.45 | down |
| A_32_P30898 |  | 8.86E-03 | 2.45 | down |
| A_24_P295633 | *PRR5L* | 1.51E-03 | 2.45 | down |
| A_32_P211188 | *PPARGC1B* | 2.38E-03 | 2.44 | down |
| A_32_P116203 | *NCF1* | 6.22E-03 | 2.44 | down |
| A_23_P132910 | *RBM47* | 7.34E-03 | 2.43 | down |
| A_32_P230736 | *LOC389033* | 6.52E-04 | 2.43 | down |
| A_23_P214222 | *MARCKS* | 1.54E-03 | 2.43 | down |
| A_23_P209944 | *RETSAT* | 4.80E-03 | 2.43 | down |
| A_32_P213930 |  | 7.76E-03 | 2.42 | down |
| A_23_P138352 | *WNT2B* | 5.73E-03 | 2.42 | down |
| A_32_P116206 | *RELL1* | 3.86E-03 | 2.42 | down |
| A_23_P253046 | *UGP2* | 2.51E-03 | 2.42 | down |
| A_24_P209171 | *SH3BGRL2* | 3.63E-03 | 2.42 | down |
| A_32_P95894 |  | 3.18E-03 | 2.42 | down |
| A_24_P413669 | *PFKFB2* | 8.85E-03 | 2.42 | down |
| A_23_P150379 | *MPZL2* | 8.42E-03 | 2.42 | down |
| A_23_P101025 | *LGALS9C* | 5.81E-03 | 2.41 | down |
| A_23_P382188 | *STAP2* | 7.73E-03 | 2.41 | down |
| A_23_P331748 | *CD33* | 3.46E-03 | 2.41 | down |
| A_24_P397294 | *LTC4S* | 6.24E-03 | 2.41 | down |
| A_24_P142973 | *PEX26* | 9.23E-03 | 2.41 | down |
| A_24_P330773 | *CALCOCO2* | 6.60E-03 | 2.41 | down |
| A_23_P10211 | *SLC2A13* | 7.71E-03 | 2.41 | down |
| A_23_P329353 | *CNRIP1* | 8.89E-03 | 2.41 | down |
| A_23_P411993 | *ITIH5* | 6.28E-03 | 2.40 | down |
| A_23_P207507 | *ABCC3* | 6.63E-03 | 2.40 | down |
| A_23_P213518 | *CAST* | 2.49E-03 | 2.40 | down |
| A_23_P214969 | *CITED2* | 9.36E-03 | 2.40 | down |
| A_24_P222655 | *C1QA* | 5.48E-03 | 2.40 | down |
| A_23_P22350 | *GRAMD3* | 6.85E-03 | 2.40 | down |
| A_23_P411612 | *SPRYD4* | 4.28E-03 | 2.40 | down |
| A_23_P252201 | *EAF2* | 3.65E-03 | 2.38 | down |
| A_23_P8311 | *TTRAP* | 3.99E-03 | 2.38 | down |
| A_24_P373152 | *CFL2* | 3.18E-03 | 2.37 | down |
| A_24_P188975 | *FLJ11235* | 8.58E-03 | 2.36 | down |
| A_23_P142289 | *GNA11* | 2.45E-03 | 2.35 | down |
| A_23_P206120 |  | 4.59E-03 | 2.35 | down |
| A_23_P149390 | *CGN* | 2.25E-03 | 2.35 | down |
| A_23_P209426 | *TRAK2* | 5.19E-03 | 2.34 | down |
| A_23_P48771 | *C14orf159* | 5.84E-03 | 2.34 | down |
| A_23_P116557 | *LGALS9* | 6.85E-03 | 2.34 | down |
| A_32_P100379 | *PDGFRA* | 4.54E-03 | 2.34 | down |
| A_32_P49854 |  | 9.35E-03 | 2.34 | down |
| A_24_P488105 | *ETNK1* | 4.61E-03 | 2.34 | down |
| A_32_P138557 | *RRN3P3* | 8.18E-03 | 2.34 | down |
| A_24_P927886 | *GNA11* | 2.35E-03 | 2.32 | down |
| A_23_P108157 | *TJP3* | 1.42E-03 | 2.32 | down |
| A_23_P158880 | *STARD5* | 1.00E-02 | 2.31 | down |
| A_32_P13823 |  | 2.40E-03 | 2.31 | down |
| A_23_P99741 | *CDKL1* | 2.14E-03 | 2.31 | down |
| A_23_P151805 | *FBLN5* | 5.55E-03 | 2.31 | down |
| A_23_P23194 | *PINK1* | 5.63E-03 | 2.31 | down |
| A_23_P411806 | *SLC44A1* | 1.16E-03 | 2.30 | down |
| A_23_P53623 | *P2RX4* | 7.04E-03 | 2.30 | down |
| A_23_P360964 | *DACT3* | 9.46E-03 | 2.30 | down |
| A_32_P81173 | *USP34* | 5.87E-03 | 2.30 | down |
| A_23_P89589 | *PER1* | 4.38E-03 | 2.30 | down |
| A_23_P256542 | *FAM162A* | 7.66E-03 | 2.30 | down |
| A_23_P368484 | *C17orf76* | 7.50E-03 | 2.29 | down |
| A_23_P105461 | *CMKLR1* | 9.41E-04 | 2.29 | down |
| A_24_P301655 | *CD33* | 3.07E-03 | 2.28 | down |
| A_23_P303718 | *DST* | 1.16E-03 | 2.28 | down |
| A_32_P175739 | *HK2* | 5.08E-03 | 2.28 | down |
| A_23_P82402 | *GLCCI1* | 2.33E-03 | 2.28 | down |
| A_23_P410859 | *ZSWIM6* | 3.80E-03 | 2.28 | down |
| A_23_P390172 | *RNASEL* | 7.00E-03 | 2.28 | down |
| A_23_P206945 | *ACOX1* | 6.22E-03 | 2.28 | down |
| A_24_P325176 | *KIAA1109* | 3.31E-03 | 2.27 | down |
| A_24_P75008 | *LOC346329* | 2.64E-03 | 2.27 | down |
| A_23_P5051 | *GNA11* | 2.32E-03 | 2.27 | down |
| A_24_P190804 | *AP1S2* | 4.29E-03 | 2.26 | down |
| A_23_P108437 | *FZD5* | 3.15E-03 | 2.26 | down |
| A_23_P25336 | *GLTP* | 1.18E-03 | 2.26 | down |
| A_32_P27991 | *BUB3* | 7.97E-03 | 2.25 | down |
| A_23_P149975 | *FAM107B* | 5.00E-03 | 2.25 | down |
| A_32_P211386 |  | 6.54E-04 | 2.25 | down |
| A_32_P166733 |  | 5.72E-03 | 2.25 | down |
| A_23_P130886 | *MPND* | 1.90E-03 | 2.25 | down |
| A_24_P311845 | *PANK3* | 8.86E-03 | 2.25 | down |
| A_23_P46618 | *PLXNA2* | 8.18E-03 | 2.24 | down |
| A_23_P252541 | *RAB7B* | 9.03E-03 | 2.24 | down |
| A_24_P317827 | *TMEM8B* | 7.71E-03 | 2.24 | down |
| A_32_P129752 | *TMEM30B* | 3.06E-03 | 2.24 | down |
| A_24_P273647 | *CCDC64B* | 3.63E-03 | 2.24 | down |
| A_23_P368558 | *TMEM131* | 1.16E-03 | 2.24 | down |
| A_32_P75772 |  | 1.48E-03 | 2.24 | down |
| A_32_P24832 | *OLFML3* | 9.84E-03 | 2.23 | down |
| A_23_P502343 | *ADAM33* | 1.27E-03 | 2.23 | down |
| A_23_P48596 | *RNASE1* | 9.50E-03 | 2.22 | down |
| A_32_P193218 | *LPP* | 8.88E-03 | 2.22 | down |
| A_23_P400580 | *FNIP2* | 2.68E-03 | 2.22 | down |
| A_32_P226786 | *FAM126B* | 8.34E-03 | 2.22 | down |
| A_24_P127159 |  | 3.67E-03 | 2.22 | down |
| A_23_P142796 | *LIMS2* | 9.23E-03 | 2.22 | down |
| A_23_P131990 | *VSX1* | 4.44E-03 | 2.22 | down |
| A_32_P206839 |  | 2.63E-03 | 2.21 | down |
| A_32_P85539 | *HCFC2* | 7.50E-03 | 2.21 | down |
| A_24_P321525 | *RERG* | 4.32E-03 | 2.21 | down |
| A_23_P58835 | *F2RL1* | 9.78E-03 | 2.20 | down |
| A_23_P150325 | *TMEM133* | 5.21E-03 | 2.20 | down |
| A_23_P129157 | *NEIL1* | 1.47E-03 | 2.19 | down |
| A_23_P250735 | *CBX7* | 5.19E-03 | 2.19 | down |
| A_23_P118254 | *FOXF1* | 9.14E-03 | 2.18 | down |
| A_23_P39602 | *NCOA1* | 4.68E-03 | 2.18 | down |
| A_32_P190737 | *FNIP2* | 4.67E-03 | 2.18 | down |
| A_23_P24044 | *CNNM2* | 2.82E-03 | 2.18 | down |
| A_32_P38637 | *KRBA1* | 6.91E-03 | 2.18 | down |
| A_24_P226008 | *MGLL* | 5.02E-03 | 2.18 | down |
| A_23_P211878 | *FLNB* | 3.61E-03 | 2.17 | down |
| A_23_P23457 | *FBLIM1* | 1.54E-03 | 2.17 | down |
| A_24_P19410 | *CBX7* | 9.28E-03 | 2.17 | down |
| A_24_P403959 | *RNASE1* | 8.89E-03 | 2.17 | down |
| A_24_P659415 |  | 8.64E-03 | 2.17 | down |
| A_23_P210330 | *HSPC159* | 6.22E-03 | 2.17 | down |
| A_24_P341187 | *GBA2* | 2.65E-03 | 2.17 | down |
| A_23_P70719 | *LAMA2* | 2.89E-03 | 2.17 | down |
| A_23_P133799 | *KLC4* | 6.00E-03 | 2.17 | down |
| A_23_P128215 | *SOCS2* | 1.48E-03 | 2.17 | down |
| A_23_P110879 | *TRAF3IP2* | 8.04E-03 | 2.16 | down |
| A_24_P306063 | *FAM135A* | 6.48E-03 | 2.16 | down |
| A_32_P174385 |  | 5.50E-03 | 2.16 | down |
| A_23_P105307 | *DGKA* | 1.18E-03 | 2.16 | down |
| A_23_P40611 | *TCN2* | 8.38E-03 | 2.16 | down |
| A_23_P254193 | *TTC38* | 8.85E-03 | 2.16 | down |
| A_32_P92840 |  | 7.68E-03 | 2.16 | down |
| A_23_P29067 | *TMPRSS2* | 8.25E-03 | 2.15 | down |
| A_23_P99249 | *SUCLG2* | 2.80E-03 | 2.15 | down |
| A_23_P397999 | *FZD5* | 1.49E-03 | 2.15 | down |
| A_23_P48747 | *DHRS1* | 4.17E-03 | 2.15 | down |
| A_24_P778741 | *LPP* | 7.54E-03 | 2.15 | down |
| A_24_P97221 | *MRPL35* | 2.38E-03 | 2.15 | down |
| A_32_P171903 |  | 3.01E-03 | 2.15 | down |
| A_23_P138524 | *CPXM2* | 7.50E-03 | 2.15 | down |
| A_23_P334864 | *FAM126B* | 2.35E-03 | 2.14 | down |
| A_23_P128084 | *ITGA7* | 2.88E-03 | 2.14 | down |
| A_23_P407565 | *CX3CR1* | 6.64E-03 | 2.14 | down |
| A_23_P123692 | *C9orf7* | 1.65E-03 | 2.14 | down |
| A_24_P387321 | *ZNF44* | 6.77E-03 | 2.14 | down |
| A_23_P305723 | *MIER1* | 1.16E-03 | 2.14 | down |
| A_32_P198731 | *NEURL1B* | 7.84E-03 | 2.13 | down |
| A_32_P464568 | *GBA2* | 2.84E-03 | 2.13 | down |
| A_23_P257155 | *ATXN7* | 6.19E-03 | 2.12 | down |
| A_32_P80850 | *COL14A1* | 3.37E-03 | 2.11 | down |
| A_23_P203702 |  | 6.88E-03 | 2.11 | down |
| A_23_P41992 | *GFRA3* | 2.33E-03 | 2.11 | down |
| A_23_P168836 | *PTK2B* | 1.16E-03 | 2.11 | down |
| A_24_P114551 | *LPP* | 5.95E-03 | 2.10 | down |
| A_23_P201764 | *DHDDS* | 1.40E-03 | 2.10 | down |
| A_23_P215024 | *LRRC1* | 1.54E-03 | 2.10 | down |
| A_23_P37317 |  | 2.74E-03 | 2.10 | down |
| A_23_P250571 | *DMXL1* | 6.19E-03 | 2.10 | down |
| A_23_P416711 | *ST6GALNAC3* | 8.79E-03 | 2.09 | down |
| A_32_P175667 |  | 3.81E-03 | 2.09 | down |
| A_23_P21644 | *HSD17B11* | 9.70E-03 | 2.09 | down |
| A_23_P310560 | *NUDT16* | 3.57E-03 | 2.09 | down |
| A_24_P373562 | *ADAP2* | 1.48E-03 | 2.09 | down |
| A_24_P90216 | *LGR4* | 8.54E-03 | 2.09 | down |
| A_23_P49009 | *LPCAT4* | 3.03E-03 | 2.09 | down |
| A_23_P119196 | *KLF2* | 3.11E-03 | 2.09 | down |
| A_23_P164528 | *WDR7* | 5.07E-03 | 2.09 | down |
| A_23_P87810 |  | 7.14E-03 | 2.09 | down |
| A_23_P24774 | *ABCC8* | 2.38E-03 | 2.09 | down |
| A_24_P122874 | *TRAK2* | 3.52E-03 | 2.08 | down |
| A_24_P600036 |  | 2.85E-03 | 2.08 | down |
| A_23_P336198 | *GLCCI1* | 1.14E-03 | 2.08 | down |
| A_23_P207650 | *ACADVL* | 7.42E-03 | 2.08 | down |
| A_23_P210731 | *ADAM33* | 1.40E-03 | 2.08 | down |
| A_23_P33791 | *SSBP2* | 1.78E-03 | 2.08 | down |
| A_24_P19884 | *ZBTB7B* | 3.70E-03 | 2.08 | down |
| A_32_P6415 | *TNRC6B* | 5.42E-03 | 2.07 | down |
| A_23_P151565 | *RALGAPA1* | 3.77E-03 | 2.07 | down |
| A_24_P89987 | *EPB41L4B* | 6.59E-03 | 2.07 | down |
| A_32_P56415 |  | 1.16E-03 | 2.07 | down |
| A_23_P398172 | *FAM135A* | 2.98E-03 | 2.06 | down |
| A_23_P27556 | *EMR1* | 7.65E-03 | 2.06 | down |
| A_23_P501547 | *ADCY6* | 8.21E-03 | 2.06 | down |
| A_24_P254949 | *PGM5* | 2.25E-03 | 2.06 | down |
| A_23_P107597 | *ATP8B1* | 4.20E-03 | 2.05 | down |
| A_23_P61426 | *MSRA* | 7.35E-03 | 2.05 | down |
| A_32_P121059 |  | 5.09E-03 | 2.05 | down |
| A_24_P893239 | *COX7B* | 9.66E-03 | 2.05 | down |
| A_23_P251705 | *ARHGEF9* | 7.76E-03 | 2.05 | down |
| A_32_P182395 |  | 8.86E-03 | 2.05 | down |
| A_23_P300826 | *C6orf136* | 2.45E-03 | 2.05 | down |
| A_24_P102343 | *EPN3* | 5.19E-03 | 2.05 | down |
| A_24_P216654 | *SOAT1* | 4.95E-03 | 2.04 | down |
| A_24_P329924 | *SIK3* | 4.78E-03 | 2.04 | down |
| A_32_P23838 | *CYP4V2* | 3.38E-03 | 2.04 | down |
| A_24_P133475 | *DHRS1* | 4.57E-03 | 2.04 | down |
| A_32_P47543 |  | 8.63E-03 | 2.04 | down |
| A_23_P337934 | *FBLIM1* | 1.20E-03 | 2.04 | down |
| A_24_P32560 | *ENHO* | 2.25E-03 | 2.04 | down |
| A_23_P159237 | *GPR20* | 3.13E-03 | 2.04 | down |
| A_23_P111267 | *SH3BGRL2* | 8.69E-03 | 2.04 | down |
| A_23_P58117 | *SHROOM3* | 2.90E-03 | 2.03 | down |
| A_32_P31785 |  | 6.13E-03 | 2.03 | down |
| A_24_P134356 | *BTBD3* | 9.16E-03 | 2.03 | down |
| A_24_P252043 | *PTP4A1* | 4.67E-03 | 2.02 | down |
| A_23_P70785 | *AIM1* | 7.73E-03 | 2.01 | down |
| A_24_P910050 |  | 2.45E-03 | 2.01 | down |
| A_23_P152428 | *MARVELD3* | 2.17E-03 | 2.01 | down |
| A_32_P103558 | *STON2* | 5.72E-03 | 2.01 | down |
| A_23_P217367 | *ATG4A* | 8.50E-03 | 2.00 | down |
| A_24_P203678 | *ACAT1* | 6.19E-03 | 2.00 | down |
| A_23_P161698 | *MMP3* | 2.93E-03 | 72.31 | up |
| A_23_P49155 | *CDH3* | 6.65E-04 | 52.90 | up |
| A_23_P122924 | *INHBA* | 6.54E-04 | 41.06 | up |
| A_32_P164246 | *FOXQ1* | 2.25E-03 | 39.58 | up |
| A_32_P161855 | *KIAA1199* | 6.52E-04 | 30.53 | up |
| A_23_P324754 | *KIAA1199* | 2.70E-03 | 29.17 | up |
| A_23_P203267 | *TRIM29* | 1.96E-03 | 28.56 | up |
| A_23_P1691 | *MMP1* | 2.07E-03 | 26.30 | up |
| A_23_P56746 | *FAP* | 2.05E-03 | 21.71 | up |
| A_23_P57784 | *CLDN1* | 1.53E-03 | 21.64 | up |
| A_23_P431776 | *ETV4* | 5.92E-03 | 20.28 | up |
| A_24_P416346 | *ETV4* | 1.90E-03 | 15.96 | up |
| A_23_P107911 | *KLK10* | 5.01E-03 | 15.27 | up |
| A_23_P7313 | *SPP1* | 8.29E-03 | 13.96 | up |
| A_23_P57417 | *MMP11* | 6.01E-03 | 13.37 | up |
| A_23_P166408 | *OSM* | 3.52E-03 | 11.83 | up |
| A_23_P111888 | *CTHRC1* | 1.66E-03 | 11.70 | up |
| A_23_P215328 | *SFRP4* | 1.84E-03 | 11.39 | up |
| A_23_P201706 | *S100A2* | 1.65E-03 | 11.16 | up |
| A_23_P135381 | *SP5* | 2.68E-03 | 11.13 | up |
| A_32_P183718 | *SP5* | 3.22E-03 | 11.00 | up |
| A_23_P207520 | *COL1A1* | 2.37E-03 | 10.65 | up |
| A_23_P210690 | *TRIB3* | 1.45E-03 | 9.88 | up |
| A_23_P5903 | *SLCO4A1* | 8.53E-04 | 9.79 | up |
| A_23_P76538 | *TESC* | 5.04E-03 | 9.76 | up |
| A_23_P403445 | *CGREF1* | 5.01E-03 | 9.72 | up |
| A_23_P200222 | *LRP8* | 2.05E-03 | 9.71 | up |
| A_23_P41804 | *NKD2* | 2.02E-03 | 9.45 | up |
| A_24_P305541 | *TRIB3* | 2.49E-03 | 9.21 | up |
| A_23_P154688 | *SLC4A11* | 2.49E-03 | 9.02 | up |
| A_23_P400298 | *PRSS22* | 6.52E-04 | 9.01 | up |
| A_23_P54055 | *JUB* | 1.11E-03 | 8.77 | up |
| A_32_P142440 | *PCSK9* | 4.50E-03 | 8.68 | up |
| A_23_P49338 | *TNFRSF12A* | 2.07E-03 | 8.44 | up |
| A_24_P335620 | *SLC7A5* | 2.25E-03 | 8.43 | up |
| A_23_P113393 | *APLN* | 8.51E-04 | 8.33 | up |
| A_23_P104522 | *NEBL* | 6.54E-04 | 7.26 | up |
| A_23_P108673 | *FAM176A* | 6.64E-03 | 7.23 | up |
| A_23_P107963 | *FUT1* | 8.51E-04 | 7.22 | up |
| A_23_P62021 | *THBS2* | 3.60E-03 | 7.19 | up |
| A_23_P79518 | *IL1B* | 8.78E-03 | 7.15 | up |
| A_23_P107981 | *SULT2B1* | 1.65E-03 | 7.03 | up |
| A_23_P92860 | *CCNO* | 7.93E-03 | 6.98 | up |
| A_23_P259692 | *PSAT1* | 5.72E-03 | 6.95 | up |
| A_23_P43164 | *SULF1* | 4.67E-03 | 6.88 | up |
| A_23_P351837 | *KLHL35* | 2.66E-03 | 6.77 | up |
| A_24_P295791 | *DGAT2* | 1.16E-03 | 6.56 | up |
| A_24_P404822 | *APLN* | 6.54E-04 | 6.27 | up |
| A_23_P69030 | *COL8A1* | 2.25E-03 | 6.18 | up |
| A_23_P42718 | *NFE2L3* | 3.36E-03 | 6.07 | up |
| A_23_P90032 | *LRRC8E* | 8.68E-04 | 6.01 | up |
| A_23_P207850 | *TNS4* | 2.66E-03 | 5.98 | up |
| A_32_P50603 | *C2orf70* | 1.42E-03 | 5.73 | up |
| A_24_P20806 | *PRR7* | 9.62E-04 | 5.68 | up |
| A_24_P348203 | *LRRC8E* | 6.65E-04 | 5.57 | up |
| A_23_P76450 | *PHLDA1* | 9.85E-04 | 5.56 | up |
| A_23_P26426 | *CPNE7* | 1.95E-03 | 5.53 | up |
| A_23_P150316 | *MMP12* | 3.22E-03 | 5.52 | up |
| A_23_P150693 | *FJX1* | 2.50E-03 | 5.47 | up |
| A_23_P24716 | *TMEM132A* | 7.96E-04 | 5.47 | up |
| A_23_P77493 | *TUBB3* | 8.95E-04 | 5.41 | up |
| A_24_P46093 | *SLC6A6* | 1.32E-03 | 5.29 | up |
| A_23_P160537 | *C1orf135* | 1.27E-03 | 5.26 | up |
| A_23_P145786 | *MLXIPL* | 2.84E-03 | 5.25 | up |
| A_32_P152696 | *LOC729983* | 1.58E-03 | 5.20 | up |
| A_23_P143190 | *MYBL2* | 8.37E-03 | 5.19 | up |
| A_23_P214168 | *COL12A1* | 6.47E-03 | 5.17 | up |
| A_24_P306896 | *LOC283711* | 4.62E-03 | 5.16 | up |
| A_23_P42168 | *MDFI* | 3.22E-03 | 5.12 | up |
| A_24_P297539 | *UBE2C* | 4.17E-03 | 5.12 | up |
| A_24_P385739 | *EIF5A2* | 2.26E-03 | 5.10 | up |
| A_23_P30464 | *PRR7* | 8.11E-04 | 5.07 | up |
| A_24_P235049 | *MTHFD1L* | 1.63E-03 | 5.04 | up |
| A_23_P340698 | *MMP12* | 4.63E-03 | 5.02 | up |
| A_24_P200831 | *MLXIPL* | 5.32E-03 | 5.01 | up |
| A_23_P356684 | *ANLN* | 1.66E-03 | 5.00 | up |
| A_23_P70827 | *KIAA1549* | 4.66E-04 | 4.94 | up |
| A_23_P1029 | *MFAP2* | 6.25E-03 | 4.94 | up |
| A_23_P166508 |  | 1.48E-03 | 4.91 | up |
| A_24_P100517 | *C9orf140* | 2.57E-03 | 4.91 | up |
| A_23_P156327 | *TGFBI* | 9.56E-04 | 4.90 | up |
| A_23_P41917 | *HOMER1* | 2.45E-03 | 4.86 | up |
| A_23_P430068 | *PDPN* | 2.69E-03 | 4.85 | up |
| A_23_P16523 | *GDF15* | 7.13E-03 | 4.85 | up |
| A_24_P299685 | *PDPN* | 2.37E-03 | 4.77 | up |
| A_32_P135243 | *MTHFD1L* | 1.27E-03 | 4.77 | up |
| A_23_P366394 | *ZAK* | 1.16E-03 | 4.76 | up |
| A_23_P53198 | *DGAT2* | 3.43E-03 | 4.74 | up |
| A_23_P117602 | *GZMB* | 6.62E-03 | 4.72 | up |
| A_24_P398147 | *NEBL* | 6.65E-04 | 4.71 | up |
| A_23_P38106 | *SPHK1* | 9.59E-03 | 4.70 | up |
| A_32_P72447 | *UBE2S* | 1.38E-03 | 4.65 | up |
| A_23_P340909 | *SKA3* | 1.86E-03 | 4.65 | up |
| A_24_P413126 | *PMEPA1* | 5.31E-03 | 4.64 | up |
| A_23_P132378 | *CELSR1* | 7.03E-03 | 4.61 | up |
| A_32_P171328 | *UBE2S* | 9.85E-04 | 4.61 | up |
| A_23_P252740 | *DSCC1* | 1.17E-03 | 4.59 | up |
| A_23_P207537 | *DUSP14* | 1.03E-03 | 4.59 | up |
| A_24_P358591 | *C2orf70* | 1.94E-03 | 4.57 | up |
| A_32_P184933 | *UBE2S* | 1.70E-03 | 4.56 | up |
| A_23_P216068 | *ATAD2* | 2.47E-03 | 4.54 | up |
| A_23_P102731 | *SMOX* | 1.72E-03 | 4.53 | up |
| A_23_P106024 | *JAG2* | 3.17E-03 | 4.51 | up |
| A_24_P8371 | *SPNS2* | 7.33E-04 | 4.50 | up |
| A_23_P57089 | *PMEPA1* | 3.92E-03 | 4.47 | up |
| A_23_P60079 | *ANGPT2* | 2.13E-03 | 4.47 | up |
| A_23_P320578 | *RGS16* | 3.57E-03 | 4.46 | up |
| A_23_P85783 | *PHGDH* | 5.42E-03 | 4.46 | up |
| A_24_P392496 | *LOC100133920* | 1.48E-03 | 4.45 | up |
| A_23_P131846 | *SNAI1* | 3.38E-03 | 4.40 | up |
| A_23_P62115 | *TIMP1* | 2.25E-03 | 4.39 | up |
| A_23_P80032 | *E2F1* | 1.48E-03 | 4.39 | up |
| A_24_P192994 | *FADS1* | 2.57E-03 | 4.37 | up |
| A_32_P399546 | *ARNTL2* | 5.70E-03 | 4.28 | up |
| A_23_P257694 | *GTF2IRD1* | 4.82E-03 | 4.25 | up |
| A_23_P94795 | *TEAD4* | 3.24E-03 | 4.24 | up |
| A_23_P323751 | *FAM83D* | 2.25E-03 | 4.23 | up |
| A_23_P63402 | *GPSM2* | 3.39E-03 | 4.19 | up |
| A_23_P161474 | *MCM10* | 6.18E-03 | 4.17 | up |
| A_24_P267452 | *CD3EAP* | 1.16E-03 | 4.16 | up |
| A_23_P203419 | *FADS1* | 2.08E-03 | 4.14 | up |
| A_23_P369899 | *TMEM158* | 9.34E-03 | 4.13 | up |
| A_23_P68610 | *TPX2* | 2.63E-03 | 4.13 | up |
| A_23_P63618 | *SCD* | 1.18E-03 | 4.11 | up |
| A_32_P163858 | *SCD* | 4.55E-03 | 4.09 | up |
| A_23_P388146 |  | 2.92E-03 | 4.08 | up |
| A_24_P383450 | *IER5L* | 1.68E-03 | 4.07 | up |
| A_23_P95029 | *SNTB1* | 3.28E-03 | 4.05 | up |
| A_23_P204751 | *ACCN2* | 6.47E-03 | 4.04 | up |
| A_23_P115872 | *CEP55* | 7.76E-03 | 4.03 | up |
| A_23_P100344 | *ORC6L* | 1.92E-03 | 4.02 | up |
| A_24_P234196 | *RRM2* | 6.88E-03 | 4.01 | up |
| A_32_P58606 | *ONECUT2* | 2.25E-03 | 4.01 | up |
| A_23_P9574 | *ECT2* | 9.62E-04 | 3.98 | up |
| A_23_P138507 | *CDK1* | 1.56E-03 | 3.97 | up |
| A_24_P67494 |  | 1.80E-03 | 3.97 | up |
| A_23_P388812 | *CKAP2L* | 1.90E-03 | 3.95 | up |
| A_32_P158181 |  | 9.56E-04 | 3.95 | up |
| A_23_P401904 | *PHF19* | 3.37E-03 | 3.94 | up |
| A_23_P217845 | *RGS16* | 3.62E-03 | 3.89 | up |
| A_23_P214908 | *MTHFD1L* | 7.64E-04 | 3.89 | up |
| A_32_P209230 | *CITED4* | 4.97E-03 | 3.88 | up |
| A_23_P52278 | *KIF11* | 1.12E-03 | 3.88 | up |
| A_23_P44684 | *ECT2* | 3.38E-03 | 3.87 | up |
| A_23_P429491 | *C11orf82* | 2.22E-03 | 3.86 | up |
| A_23_P256956 | *KIF20A* | 2.43E-03 | 3.85 | up |
| A_23_P49972 | *CDC6* | 1.16E-03 | 3.83 | up |
| A_23_P20022 | *C7orf68* | 3.11E-03 | 3.80 | up |
| A_23_P14193 | *RFC3* | 1.11E-03 | 3.80 | up |
| A_24_P915692 | *PHLDA1* | 1.54E-03 | 3.79 | up |
| A_24_P319613 | *NEK2* | 3.02E-03 | 3.74 | up |
| A_32_P116556 | *ZNF469* | 9.66E-03 | 3.74 | up |
| A_23_P92441 | *MAD2L1* | 1.16E-03 | 3.74 | up |
| A_23_P118815 | *BIRC5* | 1.34E-03 | 3.72 | up |
| A_24_P703830 | *NANOS3* | 7.51E-03 | 3.71 | up |
| A_23_P124417 | *BUB1* | 1.20E-03 | 3.71 | up |
| A_24_P257099 | *HJURP* | 3.06E-03 | 3.70 | up |
| A_23_P71558 | *RECQL4* | 1.48E-03 | 3.70 | up |
| A_23_P104617 | *GYLTL1B* | 1.86E-03 | 3.69 | up |
| A_23_P146284 | *SQLE* | 2.74E-03 | 3.65 | up |
| A_23_P422115 | *C9orf116* | 1.86E-03 | 3.63 | up |
| A_23_P35219 | *NEK2* | 2.88E-03 | 3.63 | up |
| A_23_P51085 | *SPC25* | 4.54E-03 | 3.63 | up |
| A_24_P693986 | *TRNP1* | 4.69E-03 | 3.62 | up |
| A_23_P131866 | *AURKA* | 4.24E-03 | 3.61 | up |
| A_23_P111621 | *GTF2IRD1* | 5.88E-03 | 3.59 | up |
| A_32_P226768 |  | 3.60E-03 | 3.59 | up |
| A_23_P23303 | *EXO1* | 5.19E-03 | 3.57 | up |
| A_23_P315386 | *RHPN1* | 1.81E-03 | 3.57 | up |
| A_23_P104318 | *DDIT4* | 3.92E-03 | 3.53 | up |
| A_23_P217049 | *NCS1* | 3.61E-03 | 3.53 | up |
| A_23_P115482 | *UBE2T* | 3.03E-03 | 3.52 | up |
| A_24_P356373 | *HAGHL* | 3.63E-03 | 3.52 | up |
| A_23_P74349 | *NUF2* | 2.19E-03 | 3.52 | up |
| A_24_P313504 | *PLK1* | 3.09E-03 | 3.52 | up |
| A_23_P27656 | *C19orf48* | 2.39E-03 | 3.51 | up |
| A_23_P104651 | *CDCA5* | 1.20E-03 | 3.49 | up |
| A_24_P195454 |  | 6.22E-03 | 3.49 | up |
| A_23_P34788 | *KIF2C* | 8.53E-04 | 3.48 | up |
| A_23_P121533 | *SPON2* | 7.93E-03 | 3.48 | up |
| A_32_P140262 |  | 2.98E-03 | 3.48 | up |
| A_32_P96719 | *SHCBP1* | 4.25E-03 | 3.48 | up |
| A_23_P115261 | *AGT* | 4.20E-03 | 3.47 | up |
| A_23_P92349 | *FGFRL1* | 1.12E-03 | 3.47 | up |
| A_32_P171923 | *MACC1* | 8.86E-03 | 3.46 | up |
| A_23_P58321 | *CCNA2* | 2.63E-03 | 3.45 | up |
| A_23_P126593 | *S100A11* | 1.55E-03 | 3.45 | up |
| A_23_P44836 | *NT5DC2* | 2.34E-03 | 3.43 | up |
| A_23_P57379 | *CDC45L* | 4.08E-03 | 3.43 | up |
| A_23_P168556 | *STX1A* | 2.17E-03 | 3.43 | up |
| A_23_P204158 | *RNFT2* | 5.01E-03 | 3.42 | up |
| A_23_P96325 | *ERCC6L* | 3.99E-03 | 3.42 | up |
| A_23_P88522 | *NMB* | 8.53E-04 | 3.42 | up |
| A_24_P13390 | *RNFT2* | 4.49E-03 | 3.42 | up |
| A_24_P65803 | *C20orf20* | 2.89E-03 | 3.41 | up |
| A_24_P392109 | *CENPN* | 7.31E-03 | 3.40 | up |
| A_23_P44195 | *MSI2* | 2.87E-03 | 3.39 | up |
| A_23_P145863 | *S100A11* | 1.45E-03 | 3.38 | up |
| A_23_P61487 | *LRRC20* | 4.50E-04 | 3.35 | up |
| A_23_P166526 | *RIBC2* | 1.48E-03 | 3.35 | up |
| A_23_P7642 | *SPARC* | 6.88E-03 | 3.34 | up |
| A_32_P119174 | *CENPP* | 1.78E-03 | 3.33 | up |
| A_23_P22086 |  | 9.69E-03 | 3.33 | up |
| A_23_P16078 | *PAFAH1B3* | 1.77E-03 | 3.33 | up |
| A_24_P412088 | *MCM10* | 4.97E-03 | 3.30 | up |
| A_23_P349676 | *FBXO41* | 3.25E-03 | 3.30 | up |
| A_23_P397341 | *PAQR4* | 2.54E-03 | 3.29 | up |
| A_23_P208880 | *UHRF1* | 6.16E-03 | 3.29 | up |
| A_24_P680947 | *KIF18B* | 8.53E-04 | 3.29 | up |
| A_23_P17393 | *CSE1L* | 2.32E-03 | 3.28 | up |
| A_23_P170667 | *ASPHD1* | 7.56E-03 | 3.28 | up |
| A_23_P27947 | *PDCD2L* | 1.16E-03 | 3.28 | up |
| A_24_P413884 | *CENPA* | 3.31E-03 | 3.27 | up |
| A_23_P88331 | *DLGAP5* | 2.48E-03 | 3.27 | up |
| A_23_P48669 | *CDKN3* | 7.84E-03 | 3.27 | up |
| A_23_P338912 | *PHLDA1* | 2.74E-03 | 3.26 | up |
| A_23_P106145 | *ERO1L* | 1.81E-03 | 3.26 | up |
| A_23_P17307 | *C20orf20* | 1.76E-03 | 3.26 | up |
| A_23_P31143 | *TPD52L1* | 7.68E-03 | 3.25 | up |
| A_32_P9382 | *C13orf37* | 7.05E-04 | 3.24 | up |
| A_23_P71727 | *CKS2* | 2.25E-03 | 3.24 | up |
| A_23_P161481 | *KIAA1274* | 1.86E-03 | 3.24 | up |
| A_23_P121716 | *ANXA3* | 3.91E-03 | 3.24 | up |
| A_23_P55421 | *CBX8* | 1.38E-03 | 3.23 | up |
| A_24_P16124 | *IFITM4P* | 9.62E-04 | 3.23 | up |
| A_23_P122197 | *CCNB1* | 4.01E-03 | 3.23 | up |
| A_24_P161973 | *ATP11A* | 1.94E-03 | 3.22 | up |
| A_23_P72737 | *IFITM1* | 1.03E-03 | 3.21 | up |
| A_24_P194081 | *FXYD5* | 5.45E-03 | 3.21 | up |
| A_23_P26557 | *C16orf59* | 3.03E-03 | 3.19 | up |
| A_23_P208310 | *CD3EAP* | 5.01E-03 | 3.19 | up |
| A_23_P345460 | *PLEKHG4* | 3.54E-03 | 3.18 | up |
| A_23_P3963 | *CDR2L* | 3.22E-03 | 3.18 | up |
| A_24_P218979 | *CDCA3* | 3.99E-03 | 3.18 | up |
| A_23_P92093 | *CELSR3* | 7.31E-03 | 3.18 | up |
| A_23_P214907 | *MTHFD1L* | 9.62E-04 | 3.18 | up |
| A_23_P380298 | *ProSAPiP1* | 3.02E-03 | 3.18 | up |
| A_23_P82420 | *STX1A* | 1.99E-03 | 3.17 | up |
| A_23_P132175 | *RTN4R* | 1.94E-03 | 3.17 | up |
| A_23_P118174 | *PLK1* | 4.68E-03 | 3.16 | up |
| A_23_P49878 | *FAM64A* | 2.70E-03 | 3.16 | up |
| A_23_P88740 | *CENPN* | 6.31E-03 | 3.15 | up |
| A_23_P252106 | *RIPK2* | 1.45E-03 | 3.15 | up |
| A_23_P259586 | *TTK* | 1.86E-03 | 3.13 | up |
| A_23_P18579 | *PTTG2* | 4.63E-03 | 3.12 | up |
| A_24_P204358 | *PYCR1* | 2.87E-03 | 3.12 | up |
| A_23_P162476 | *CDCA3* | 4.98E-03 | 3.11 | up |
| A_32_P226186 | *KIAA1549* | 7.64E-04 | 3.11 | up |
| A_23_P52298 | *NPM3* | 1.11E-03 | 3.10 | up |
| A_23_P370989 | *MCM4* | 8.51E-04 | 3.10 | up |
| A_24_P57367 | *AHCY* | 9.93E-03 | 3.09 | up |
| A_23_P130194 | *PYCR1* | 3.37E-03 | 3.08 | up |
| A_24_P69095 | *ENC1* | 8.68E-04 | 3.08 | up |
| A_23_P205449 | *CDCA4* | 1.94E-03 | 3.08 | up |
| A_23_P415443 | *NCAPH* | 1.12E-03 | 3.08 | up |
| A_24_P105102 | *PKMYT1* | 4.48E-03 | 3.07 | up |
| A_23_P362046 | *C13orf27* | 1.16E-03 | 3.07 | up |
| A_23_P150667 | *KIF18A* | 2.93E-03 | 3.05 | up |
| A_23_P133694 | *SLC29A1* | 4.17E-03 | 3.05 | up |
| A_23_P345707 | *C15orf42* | 1.45E-03 | 3.04 | up |
| A_24_P7040 |  | 1.02E-03 | 3.04 | up |
| A_24_P860703 | *LOC388796* | 6.01E-03 | 3.04 | up |
| A_23_P302654 | *CEP72* | 8.89E-03 | 3.04 | up |
| A_32_P210202 | *E2F7* | 2.64E-03 | 3.04 | up |
| A_23_P132874 | *C3orf26* | 1.22E-03 | 3.04 | up |
| A_23_P360240 | *MYEOV* | 9.95E-04 | 3.03 | up |
| A_23_P213424 | *ENC1* | 1.47E-03 | 3.03 | up |
| A_32_P142459 |  | 6.19E-03 | 3.03 | up |
| A_24_P850187 |  | 2.92E-03 | 3.03 | up |
| A_23_P43800 | *BOP1* | 7.66E-03 | 3.03 | up |
| A_24_P254933 |  | 9.84E-04 | 3.02 | up |
| A_23_P50477 | *BCL2L12* | 1.13E-03 | 3.02 | up |
| A_23_P84929 | *SLC38A5* | 4.66E-03 | 3.02 | up |
| A_23_P253752 | *FAM54A* | 4.95E-03 | 3.02 | up |
| A_23_P207783 | *TBC1D16* | 6.09E-03 | 3.02 | up |
| A_32_P109296 | *C15orf42* | 6.54E-04 | 3.02 | up |
| A_23_P7636 | *PTTG1* | 4.07E-03 | 3.01 | up |
| A_24_P273014 |  | 1.02E-03 | 3.01 | up |
| A_23_P112774 | *PTP4A3* | 6.61E-03 | 3.00 | up |
| A_24_P122137 | *LIF* | 6.54E-03 | 3.00 | up |
| A_24_P211151 | *EXOSC5* | 3.84E-03 | 2.99 | up |
| A_23_P151405 | *CKAP2* | 7.64E-04 | 2.98 | up |
| A_23_P65240 | *COL4A1* | 8.25E-03 | 2.98 | up |
| A_24_P418408 | *FAM89A* | 3.75E-03 | 2.97 | up |
| A_23_P376870 | *C14orf79* | 1.33E-03 | 2.97 | up |
| A_23_P167674 | *F12* | 4.36E-03 | 2.97 | up |
| A_24_P161463 | *C20orf199* | 2.08E-03 | 2.97 | up |
| A_23_P57588 | *GTSE1* | 3.18E-03 | 2.96 | up |
| A_23_P139820 | *SLC11A2* | 6.28E-03 | 2.96 | up |
| A_23_P66211 | *PAQR4* | 4.76E-03 | 2.96 | up |
| A_23_P155815 | *NCAPG* | 8.51E-04 | 2.95 | up |
| A_23_P102183 |  | 1.78E-03 | 2.95 | up |
| A_23_P717 | *TMEM206* | 8.53E-04 | 2.94 | up |
| A_23_P118834 | *TOP2A* | 9.54E-03 | 2.94 | up |
| A_23_P57306 | *CHAF1B* | 2.70E-03 | 2.93 | up |
| A_23_P60016 | *PTTG3P* | 6.12E-03 | 2.93 | up |
| A_23_P141893 | *PPM1N* | 3.82E-03 | 2.93 | up |
| A_23_P211926 | *WNT5A* | 8.38E-03 | 2.92 | up |
| A_23_P70249 | *CDC25C* | 3.65E-03 | 2.92 | up |
| A_23_P250313 | *KIAA1524* | 2.50E-03 | 2.92 | up |
| A_23_P98282 | *SPTBN2* | 2.70E-03 | 2.92 | up |
| A_23_P107942 | *ZNF473* | 2.89E-03 | 2.92 | up |
| A_23_P52410 | *RTKN2* | 9.26E-03 | 2.91 | up |
| A_24_P225970 | *SGOL1* | 3.70E-03 | 2.90 | up |
| A_23_P15727 | *FKBP10* | 4.56E-03 | 2.90 | up |
| A_23_P16673 | *CNN2* | 1.11E-03 | 2.89 | up |
| A_23_P127533 | *DCUN1D5* | 1.17E-03 | 2.89 | up |
| A_23_P133956 | *KIFC1* | 5.78E-03 | 2.89 | up |
| A_24_P287941 | *PSMC3IP* | 5.92E-03 | 2.88 | up |
| A_23_P125265 | *KPNA2* | 2.58E-03 | 2.88 | up |
| A_23_P82738 | *RAD54B* | 7.64E-04 | 2.87 | up |
| A_32_P206698 | *CKS1B* | 1.16E-03 | 2.87 | up |
| A_23_P206059 | *PRC1* | 2.92E-03 | 2.87 | up |
| A_32_P64919 | *DIAPH3* | 6.03E-03 | 2.87 | up |
| A_23_P339705 | *WDR62* | 5.42E-03 | 2.86 | up |
| A_23_P50990 | *CENPO* | 1.38E-03 | 2.86 | up |
| A_23_P37954 | *CCNF* | 1.11E-03 | 2.85 | up |
| A_32_P127153 | *SORD* | 7.64E-04 | 2.85 | up |
| A_24_P123347 | *PPAT* | 8.72E-04 | 2.85 | up |
| A_23_P24192 | *RRP12* | 3.56E-03 | 2.84 | up |
| A_23_P428326 | *TBC1D16* | 3.46E-03 | 2.84 | up |
| A_23_P206077 | *AEN* | 6.05E-03 | 2.84 | up |
| A_23_P56213 | *GRAMD1A* | 4.95E-03 | 2.84 | up |
| A_23_P54517 | *TYRO3* | 5.00E-03 | 2.84 | up |
| A_23_P369328 | *C10orf35* | 2.91E-03 | 2.84 | up |
| A_23_P131289 | *CHPF* | 9.84E-04 | 2.83 | up |
| A_23_P149200 | *CDC20* | 9.28E-03 | 2.81 | up |
| A_23_P23206 | *MAD2L2* | 2.85E-03 | 2.81 | up |
| A_32_P151800 | *FAM72D* | 7.01E-03 | 2.81 | up |
| A_23_P331895 | *TTYH3* | 4.83E-03 | 2.81 | up |
| A_23_P346206 | *RAE1* | 3.46E-03 | 2.81 | up |
| A_23_P48835 | *KIF23* | 2.31E-03 | 2.81 | up |
| A_23_P24444 | *DHCR7* | 1.63E-03 | 2.80 | up |
| A_24_P142743 | *CNN2* | 1.32E-03 | 2.79 | up |
| A_24_P359856 | *HDAC4* | 5.00E-03 | 2.79 | up |
| A_24_P911179 | *ASPM* | 3.65E-03 | 2.78 | up |
| A_24_P190168 | *TMEM97* | 2.49E-03 | 2.78 | up |
| A_23_P129014 | *C14orf143* | 2.92E-03 | 2.78 | up |
| A_24_P868905 |  | 1.60E-03 | 2.78 | up |
| A_23_P256890 | *PTRH1* | 2.05E-03 | 2.78 | up |
| A_32_P49284 |  | 6.32E-03 | 2.77 | up |
| A_24_P148811 | *RUVBL1* | 2.70E-03 | 2.76 | up |
| A_24_P628384 | *ALKBH2* | 6.54E-04 | 2.76 | up |
| A_23_P119254 | *ASF1B* | 8.95E-03 | 2.75 | up |
| A_32_P19840 |  | 1.94E-03 | 2.75 | up |
| A_23_P138465 | *NOLC1* | 6.76E-03 | 2.75 | up |
| A_32_P95729 | *FANCI* | 2.94E-03 | 2.75 | up |
| A_24_P377499 | *OSBPL3* | 2.94E-03 | 2.74 | up |
| A_23_P375104 | *FANCI* | 1.20E-03 | 2.74 | up |
| A_23_P314151 | *NOLC1* | 3.89E-03 | 2.74 | up |
| A_23_P252125 |  | 5.68E-03 | 2.73 | up |
| A_24_P873688 | *CENPN* | 7.93E-03 | 2.73 | up |
| A_23_P128613 | *KDELC1* | 1.16E-03 | 2.73 | up |
| A_23_P150935 | *TROAP* | 1.27E-03 | 2.73 | up |
| A_23_P119095 | *PPP1R13L* | 2.11E-03 | 2.73 | up |
| A_23_P94422 | *MELK* | 9.00E-03 | 2.73 | up |
| A_24_P176714 | *B9D1* | 9.07E-03 | 2.72 | up |
| A_23_P87545 | *IFITM3* | 1.38E-03 | 2.72 | up |
| A_23_P116123 | *CHEK1* | 6.22E-03 | 2.71 | up |
| A_24_P287043 | *IFITM2* | 4.72E-03 | 2.71 | up |
| A_23_P129967 | *C17orf53* | 1.20E-03 | 2.71 | up |
| A_23_P401 | *CENPF* | 3.01E-03 | 2.71 | up |
| A_23_P123343 | *NUDCD1* | 1.07E-03 | 2.70 | up |
| A_24_P4054 | *TRIP6* | 9.18E-03 | 2.70 | up |
| A_32_P201521 | *TMEM97* | 2.58E-03 | 2.70 | up |
| A_24_P193011 | *CCND1* | 8.54E-04 | 2.70 | up |
| A_23_P99292 | *RAD51AP1* | 1.86E-03 | 2.70 | up |
| A_23_P130995 | *FXYD5* | 8.08E-03 | 2.70 | up |
| A_23_P89621 | *CBX4* | 2.11E-03 | 2.69 | up |
| A_23_P41674 | *GRPEL2* | 1.41E-03 | 2.69 | up |
| A_24_P380022 | *EIF5A2* | 7.54E-03 | 2.68 | up |
| A_23_P92261 | *ECE2* | 4.20E-03 | 2.68 | up |
| A_23_P218523 | *C19orf28* | 2.38E-03 | 2.68 | up |
| A_23_P166023 | *PFDN4* | 4.59E-03 | 2.68 | up |
| A_32_P188921 |  | 3.88E-03 | 2.68 | up |
| A_24_P124550 | *CCND1* | 1.36E-03 | 2.68 | up |
| A_23_P74115 | *RAD54L* | 1.90E-03 | 2.68 | up |
| A_23_P138461 | *C10orf2* | 1.45E-03 | 2.68 | up |
| A_23_P28153 | *SCLY* | 1.78E-03 | 2.68 | up |
| A_24_P532589 | *C13orf37* | 8.53E-04 | 2.68 | up |
| A_23_P77103 | *SORD* | 1.89E-03 | 2.68 | up |
| A_32_P185317 | *LOC645249* | 5.10E-03 | 2.68 | up |
| A_23_P369634 | *C8orf73* | 9.98E-03 | 2.68 | up |
| A_24_P151920 | *TMEM97* | 1.95E-03 | 2.67 | up |
| A_23_P345139 | *C19orf28* | 3.65E-03 | 2.67 | up |
| A_23_P41280 | *PAICS* | 1.47E-03 | 2.67 | up |
| A_23_P52017 | *ASPM* | 4.67E-03 | 2.66 | up |
| A_23_P65110 | *RACGAP1* | 1.75E-03 | 2.65 | up |
| A_24_P506977 | *C7orf40* | 6.52E-04 | 2.64 | up |
| A_23_P151150 | *FOXM1* | 7.23E-03 | 2.64 | up |
| A_23_P76006 | *SERPINH1* | 6.19E-03 | 2.64 | up |
| A_23_P168443 | *EPHB4* | 6.52E-04 | 2.63 | up |
| A_32_P13719 | *XPOT* | 7.03E-03 | 2.63 | up |
| A_23_P31721 | *E2F5* | 3.60E-03 | 2.62 | up |
| A_23_P57667 | *PLXNA1* | 6.54E-04 | 2.62 | up |
| A_24_P406693 | *P4HA1* | 2.26E-03 | 2.60 | up |
| A_24_P916195 |  | 5.73E-03 | 2.60 | up |
| A_32_P184937 |  | 5.51E-03 | 2.59 | up |
| A_32_P170925 | *TXNRD3* | 1.16E-03 | 2.59 | up |
| A_23_P136805 | *ARHGAP11A* | 8.10E-03 | 2.59 | up |
| A_32_P89691 | *SORD* | 2.11E-03 | 2.59 | up |
| A_23_P164427 | *C17orf39* | 2.88E-03 | 2.59 | up |
| A_23_P67725 | *LMNB2* | 3.03E-03 | 2.58 | up |
| A_24_P4212 | *PYCRL* | 5.68E-03 | 2.58 | up |
| A_24_P76142 |  | 7.64E-04 | 2.58 | up |
| A_23_P6771 | *LMCD1* | 2.87E-03 | 2.58 | up |
| A_23_P418031 | *IFFO2* | 5.50E-03 | 2.57 | up |
| A_23_P425502 | *DONSON* | 3.27E-03 | 2.57 | up |
| A_32_P24165 | *FANCD2* | 5.08E-03 | 2.56 | up |
| A_23_P18196 | *RFC4* | 8.53E-04 | 2.56 | up |
| A_24_P315405 |  | 2.66E-03 | 2.56 | up |
| A_23_P145694 | *ASNS* | 7.89E-03 | 2.56 | up |
| A_23_P164826 | *RNASEH2A* | 2.19E-03 | 2.56 | up |
| A_23_P158596 | *AGTRAP* | 7.64E-04 | 2.56 | up |
| A_23_P51966 |  | 5.04E-03 | 2.55 | up |
| A_23_P87769 | *C12orf48* | 9.27E-03 | 2.55 | up |
| A_32_P186474 | *RACGAP1* | 2.78E-03 | 2.54 | up |
| A_23_P15123 | *UBFD1* | 1.92E-03 | 2.54 | up |
| A_23_P420981 | *C14orf79* | 9.95E-04 | 2.54 | up |
| A_23_P25525 | *GTF3A* | 5.47E-03 | 2.54 | up |
| A_23_P45917 | *CKS1B* | 2.92E-03 | 2.54 | up |
| A_23_P310068 | *C12orf29* | 2.85E-03 | 2.54 | up |
| A_23_P154488 | *PNPT1* | 4.76E-03 | 2.53 | up |
| A_24_P375962 |  | 9.30E-03 | 2.53 | up |
| A_24_P258073 | *C10orf2* | 8.51E-04 | 2.52 | up |
| A_23_P70007 | *HMMR* | 3.45E-03 | 2.52 | up |
| A_24_P340866 |  | 1.38E-03 | 2.51 | up |
| A_23_P37704 | *CDT1* | 3.60E-03 | 2.51 | up |
| A_23_P200866 | *STMN1* | 2.92E-03 | 2.51 | up |
| A_32_P192430 | *CKS1B* | 2.69E-03 | 2.51 | up |
| A_23_P74663 | *TAF1A* | 7.84E-03 | 2.51 | up |
| A_24_P176374 | *CDT1* | 5.21E-03 | 2.50 | up |
| A_23_P71146 | *POLD2* | 2.87E-03 | 2.50 | up |
| A_23_P426398 | *PGAM5* | 4.62E-03 | 2.50 | up |
| A_23_P340722 | *XPOT* | 3.13E-03 | 2.50 | up |
| A_23_P122815 | *CALU* | 5.51E-04 | 2.50 | up |
| A_23_P313223 | *C11orf84* | 3.86E-03 | 2.49 | up |
| A_23_P215875 | *DCAF13* | 3.57E-03 | 2.49 | up |
| A_32_P158376 |  | 8.64E-03 | 2.49 | up |
| A_24_P158385 | *ZMYND19* | 4.78E-03 | 2.49 | up |
| A_23_P44132 | *FASN* | 4.26E-03 | 2.48 | up |
| A_23_P82478 | *PUS7* | 6.85E-03 | 2.48 | up |
| A_23_P170491 | *TRAIP* | 7.64E-04 | 2.48 | up |
| A_32_P12610 | *E2F6* | 2.15E-03 | 2.48 | up |
| A_23_P46539 | *PSRC1* | 4.13E-03 | 2.48 | up |
| A_23_P17826 | *SLC5A1* | 2.05E-03 | 2.48 | up |
| A_32_P148476 | *LOC389842* | 4.97E-03 | 2.48 | up |
| A_24_P227831 | *ABCC1* | 1.01E-03 | 2.48 | up |
| A_23_P50389 | *NAT14* | 3.12E-03 | 2.47 | up |
| A_23_P168747 | *NCAPG2* | 2.25E-03 | 2.47 | up |
| A_32_P71447 | *NCAPD3* | 2.27E-03 | 2.47 | up |
| A_23_P42257 | *IER3* | 9.70E-03 | 2.47 | up |
| A_23_P214950 | *PERP* | 3.56E-03 | 2.47 | up |
| A_23_P215517 | *KLHL7* | 6.85E-03 | 2.47 | up |
| A_23_P111297 | *RPP40* | 4.97E-03 | 2.47 | up |
| A_24_P258051 | *MASTL* | 8.81E-04 | 2.47 | up |
| A_23_P113634 | *CBFB* | 7.27E-04 | 2.46 | up |
| A_24_P942481 | *GPR180* | 2.00E-03 | 2.46 | up |
| A_23_P74914 | *URB2* | 3.29E-03 | 2.46 | up |
| A_23_P110802 | *CENPH* | 2.29E-03 | 2.46 | up |
| A_24_P195621 | *LOC341056* | 8.43E-03 | 2.46 | up |
| A_24_P945396 | *SF3B3* | 2.70E-03 | 2.46 | up |
| A_23_P62959 | *PHLDA3* | 9.33E-03 | 2.46 | up |
| A_24_P385341 | *C1orf107* | 8.51E-04 | 2.45 | up |
| A_23_P34741 | *ZNF593* | 7.60E-03 | 2.45 | up |
| A_23_P202837 | *CCND1* | 8.51E-04 | 2.45 | up |
| A_23_P158239 | *SHMT2* | 6.82E-03 | 2.45 | up |
| A_23_P202206 | *GSTO2* | 1.86E-03 | 2.44 | up |
| A_23_P161338 | *PPA1* | 2.84E-03 | 2.44 | up |
| A_23_P24997 | *CDK4* | 1.13E-03 | 2.44 | up |
| A_23_P119789 | *TMEM185B* | 9.48E-03 | 2.44 | up |
| A_24_P15502 |  | 2.05E-03 | 2.43 | up |
| A_23_P353436 | *CEP78* | 2.96E-03 | 2.43 | up |
| A_23_P354297 | *CHTF18* | 3.36E-03 | 2.43 | up |
| A_23_P119778 | *SLC39A10* | 6.22E-03 | 2.43 | up |
| A_23_P162719 | *DIAPH3* | 4.77E-03 | 2.43 | up |
| A_23_P370097 | *ALS2CR4* | 3.25E-03 | 2.43 | up |
| A_23_P161501 | *UBTD1* | 3.52E-03 | 2.43 | up |
| A_23_P50167 | *SLC39A6* | 5.03E-03 | 2.42 | up |
| A_32_P76720 | *NT5DC3* | 7.65E-03 | 2.42 | up |
| A_23_P411296 | *CEBPB* | 4.20E-03 | 2.42 | up |
| A_24_P856273 |  | 1.36E-03 | 2.42 | up |
| A_32_P120084 | *CCDC59* | 1.18E-03 | 2.41 | up |
| A_23_P17575 | *AHCY* | 7.74E-03 | 2.41 | up |
| A_24_P407311 | *ERO1L* | 8.62E-04 | 2.41 | up |
| A_24_P25346 | *CIRH1A* | 2.48E-03 | 2.41 | up |
| A_23_P250607 | *PLS3* | 7.61E-03 | 2.41 | up |
| A_23_P375 | *CDCA8* | 3.28E-03 | 2.41 | up |
| A_23_P133995 | *PPIL1* | 5.45E-03 | 2.41 | up |
| A_23_P25873 | *WDHD1* | 2.47E-03 | 2.41 | up |
| A_24_P761727 |  | 5.51E-03 | 2.41 | up |
| A_23_P32913 | *ARMC10* | 2.59E-03 | 2.41 | up |
| A_32_P514599 |  | 1.14E-03 | 2.40 | up |
| A_23_P200310 | *DEPDC1* | 6.10E-03 | 2.40 | up |
| A_32_P44274 | *CHTF18* | 3.86E-03 | 2.40 | up |
| A_23_P134295 | *NUDT1* | 9.28E-03 | 2.39 | up |
| A_23_P359497 | *TMEM231* | 7.25E-03 | 2.39 | up |
| A_23_P145584 | *UBE2H* | 1.74E-03 | 2.39 | up |
| A_24_P736638 |  | 8.53E-04 | 2.39 | up |
| A_23_P122863 | *GRB10* | 9.08E-03 | 2.39 | up |
| A_32_P157965 | *EIF2S2* | 1.44E-03 | 2.39 | up |
| A_23_P316960 | *GRINA* | 6.09E-03 | 2.39 | up |
| A_23_P135364 | *DTYMK* | 3.17E-03 | 2.39 | up |
| A_23_P398515 | *PKMYT1* | 3.65E-03 | 2.39 | up |
| A_23_P12874 | *GTPBP4* | 1.34E-03 | 2.38 | up |
| A_23_P82169 | *SOX4* | 6.22E-03 | 2.38 | up |
| A_23_P215208 | *FIGNL1* | 4.77E-03 | 2.38 | up |
| A_24_P289636 |  | 2.54E-03 | 2.38 | up |
| A_23_P202392 | *SUV39H2* | 2.38E-03 | 2.38 | up |
| A_23_P41327 | *LYAR* | 2.05E-03 | 2.38 | up |
| A_32_P103633 | *MCM2* | 1.20E-03 | 2.38 | up |
| A_23_P169629 | *SHMT2* | 5.20E-03 | 2.38 | up |
| A_23_P63038 | *LEPRE1* | 1.36E-03 | 2.38 | up |
| A_23_P52286 | *DPCD* | 4.21E-03 | 2.38 | up |
| A_23_P152136 | *GINS3* | 1.27E-03 | 2.37 | up |
| A_23_P121222 | *RAD18* | 1.46E-03 | 2.37 | up |
| A_23_P120458 | *RNF114* | 6.11E-03 | 2.37 | up |
| A_23_P88119 | *HSPH1* | 7.50E-03 | 2.37 | up |
| A_23_P39088 | *PRMT1* | 3.53E-03 | 2.37 | up |
| A_23_P71904 | *METTL11A* | 2.91E-03 | 2.36 | up |
| A_23_P166159 | *PDRG1* | 3.09E-03 | 2.36 | up |
| A_24_P47547 | *RAN* | 1.84E-03 | 2.36 | up |
| A_23_P139983 | *XPO4* | 1.16E-03 | 2.36 | up |
| A_23_P145197 | *BYSL* | 5.95E-03 | 2.36 | up |
| A_23_P310317 | *TCOF1* | 7.64E-04 | 2.35 | up |
| A_24_P366535 | *LOC100128760* | 5.20E-03 | 2.35 | up |
| A_32_P28939 | *ALKBH2* | 9.62E-04 | 2.35 | up |
| A_23_P51269 |  | 1.53E-03 | 2.35 | up |
| A_32_P37733 |  | 4.92E-03 | 2.35 | up |
| A_23_P91590 | *RANBP1* | 3.28E-03 | 2.35 | up |
| A_32_P167592 |  | 1.77E-03 | 2.35 | up |
| A_23_P54626 | *CIRH1A* | 2.80E-03 | 2.34 | up |
| A_23_P3849 | *TRAP1* | 6.12E-03 | 2.34 | up |
| A_23_P156842 | *EEF1E1* | 3.79E-03 | 2.34 | up |
| A_23_P91870 | *CHCHD6* | 3.50E-03 | 2.34 | up |
| A_23_P163099 | *POLE2* | 7.87E-03 | 2.34 | up |
| A_24_P171549 | *CDCA7* | 3.01E-03 | 2.34 | up |
| A_32_P49334 | *LOC284889* | 8.11E-04 | 2.34 | up |
| A_23_P35021 | *B3GALT6* | 3.84E-03 | 2.34 | up |
| A_23_P112159 | *EIF2C2* | 5.36E-03 | 2.34 | up |
| A_24_P516215 | *NOB1* | 1.18E-03 | 2.33 | up |
| A_23_P382775 | *BBC3* | 8.58E-03 | 2.32 | up |
| A_32_P72341 | *TRIM59* | 9.30E-03 | 2.32 | up |
| A_23_P123974 | *DTYMK* | 2.25E-03 | 2.31 | up |
| A_23_P91619 | *MIF* | 8.81E-04 | 2.31 | up |
| A_23_P254612 | *DBF4* | 1.72E-03 | 2.30 | up |
| A_23_P38505 | *CXCL16* | 6.54E-03 | 2.30 | up |
| A_32_P201496 | *POLR3H* | 1.09E-03 | 2.30 | up |
| A_23_P415984 | *NPAS2* | 4.14E-03 | 2.30 | up |
| A_23_P31584 | *RABL5* | 9.62E-04 | 2.30 | up |
| A_23_P25019 | *PRIM1* | 5.51E-03 | 2.30 | up |
| A_23_P67708 | *TCF3* | 3.82E-03 | 2.30 | up |
| A_23_P31315 | *CBX3* | 2.92E-03 | 2.30 | up |
| A_23_P162970 | *IPO4* | 9.44E-03 | 2.30 | up |
| A_23_P88630 | *BLM* | 7.19E-03 | 2.30 | up |
| A_23_P361419 | *DEPDC1B* | 6.39E-03 | 2.29 | up |
| A_32_P192823 | *PRPS1L1* | 5.30E-03 | 2.29 | up |
| A_23_P46309 | *RCC1* | 3.65E-03 | 2.29 | up |
| A_24_P793228 | *LOC442517* | 3.82E-03 | 2.29 | up |
| A_24_P200427 | *PAICS* | 3.21E-03 | 2.29 | up |
| A_23_P32707 | *ESPL1* | 8.41E-03 | 2.29 | up |
| A_24_P268676 | *BHLHE40* | 3.24E-03 | 2.29 | up |
| A_32_P76091 | *HSPD1* | 7.64E-04 | 2.29 | up |
| A_23_P99920 | *PTPLAD1* | 2.19E-03 | 2.29 | up |
| A_23_P361405 | *HYAL3* | 5.86E-03 | 2.29 | up |
| A_23_P212844 | *TACC3* | 5.86E-03 | 2.28 | up |
| A_23_P93690 | *MCM7* | 7.49E-03 | 2.28 | up |
| A_23_P146584 | *C9orf30* | 2.50E-03 | 2.28 | up |
| A_23_P40049 | *CAD* | 4.64E-03 | 2.28 | up |
| A_23_P17739 | *ZNF74* | 3.18E-03 | 2.28 | up |
| A_23_P35230 | *CD46* | 5.73E-03 | 2.28 | up |
| A_23_P60405 | *DDX31* | 2.15E-03 | 2.28 | up |
| A_23_P96542 | *VMA21* | 3.29E-03 | 2.28 | up |
| A_24_P335358 | *PUS1* | 6.15E-03 | 2.28 | up |
| A_24_P295245 | *ASPH* | 4.62E-03 | 2.28 | up |
| A_23_P120270 | *MCFD2* | 9.77E-03 | 2.28 | up |
| A_23_P118038 | *NUTF2* | 8.93E-03 | 2.27 | up |
| A_23_P76882 | *CCNB1IP1* | 2.56E-03 | 2.27 | up |
| A_23_P160968 | *LAMC2* | 3.57E-03 | 2.27 | up |
| A_23_P253524 | *CENPE* | 3.52E-03 | 2.27 | up |
| A_32_P230720 | *E2F6* | 8.51E-04 | 2.27 | up |
| A_23_P78888 | *FBL* | 7.32E-03 | 2.27 | up |
| A_24_P187948 | *BID* | 1.23E-03 | 2.27 | up |
| A_23_P344853 | *WDR43* | 9.53E-04 | 2.27 | up |
| A_23_P21033 | *GMPS* | 6.54E-04 | 2.27 | up |
| A_23_P144622 | *GNPDA1* | 3.42E-03 | 2.26 | up |
| A_24_P239140 | *GART* | 9.89E-03 | 2.26 | up |
| A_24_P153853 | *TRIM37* | 3.91E-03 | 2.26 | up |
| A_24_P247616 |  | 3.24E-03 | 2.26 | up |
| A_24_P213206 | *ZNF259P1* | 4.34E-03 | 2.25 | up |
| A_24_P316364 | *REXO2* | 3.95E-03 | 2.25 | up |
| A_24_P752362 |  | 1.03E-03 | 2.25 | up |
| A_23_P75811 | *SLC3A2* | 7.65E-03 | 2.25 | up |
| A_23_P319719 | *PGAM5* | 4.71E-03 | 2.25 | up |
| A_24_P891276 |  | 9.40E-03 | 2.24 | up |
| A_23_P329870 | *RHBDF2* | 2.34E-03 | 2.24 | up |
| A_24_P117964 | *BOD1* | 4.92E-03 | 2.24 | up |
| A_24_P290013 | *hCG_1990547* | 1.66E-03 | 2.24 | up |
| A_23_P77048 | *SLC25A29* | 4.49E-03 | 2.23 | up |
| A_23_P118061 | *CKLF* | 6.51E-03 | 2.23 | up |
| A_24_P217572 | *EDNRA* | 7.94E-03 | 2.23 | up |
| A_23_P6802 | *RRP9* | 4.07E-03 | 2.23 | up |
| A_24_P153324 |  | 2.19E-03 | 2.23 | up |
| A_24_P195327 |  | 5.06E-03 | 2.23 | up |
| A_23_P399726 | *C22orf29* | 7.65E-03 | 2.23 | up |
| A_23_P11461 | *UBE2V1* | 6.39E-03 | 2.23 | up |
| A_32_P108474 | *ABCE1* | 3.36E-03 | 2.23 | up |
| A_23_P218685 | *UBE2V1* | 6.00E-03 | 2.23 | up |
| A_23_P415882 | *TIMM50* | 2.62E-03 | 2.22 | up |
| A_32_P96752 |  | 4.35E-03 | 2.22 | up |
| A_32_P90080 | *ARMC10* | 1.20E-03 | 2.22 | up |
| A_23_P53276 | *TIMELESS* | 2.25E-03 | 2.22 | up |
| A_23_P399501 | *PKM2* | 3.60E-03 | 2.21 | up |
| A_23_P218751 | *GNB1L* | 3.59E-03 | 2.21 | up |
| A_23_P318300 | *ZAK* | 3.96E-03 | 2.21 | up |
| A_24_P263543 | *LOC119358* | 1.16E-03 | 2.21 | up |
| A_23_P90419 | *PBX4* | 4.67E-03 | 2.21 | up |
| A_23_P11862 | *C1orf112* | 6.01E-03 | 2.21 | up |
| A_23_P26375 | *ACD* | 1.54E-03 | 2.20 | up |
| A_23_P345212 | *BOD1P* | 3.98E-03 | 2.20 | up |
| A_23_P209200 | *CCNE1* | 3.85E-03 | 2.20 | up |
| A_23_P303329 | *CTU1* | 1.56E-03 | 2.20 | up |
| A_23_P4679 | *ERF* | 4.27E-03 | 2.20 | up |
| A_24_P218757 | *FAM86C* | 3.52E-03 | 2.20 | up |
| A_23_P32463 | *EXOSC4* | 6.63E-03 | 2.20 | up |
| A_23_P89509 | *SPAG5* | 1.72E-03 | 2.20 | up |
| A_32_P11723 | *EIF2S2* | 5.26E-03 | 2.20 | up |
| A_24_P415260 |  | 4.93E-03 | 2.20 | up |
| A_23_P70328 | *CENPQ* | 6.54E-04 | 2.20 | up |
| A_24_P74160 | *SNRPD2* | 9.56E-04 | 2.20 | up |
| A_24_P193582 | *DEF8* | 3.82E-03 | 2.20 | up |
| A_32_P122754 | *C9orf30* | 2.22E-03 | 2.20 | up |
| A_23_P251421 | *CDCA7* | 4.45E-03 | 2.19 | up |
| A_24_P170295 | *HSP90AB1* | 4.54E-03 | 2.19 | up |
| A_24_P75019 | *RP9P* | 1.05E-03 | 2.19 | up |
| A_23_P112412 | *TEX10* | 1.38E-03 | 2.19 | up |
| A_23_P141315 | *NLE1* | 4.98E-03 | 2.19 | up |
| A_23_P146997 | *CXorf15* | 8.51E-04 | 2.19 | up |
| A_23_P214411 | *GLO1* | 2.31E-03 | 2.19 | up |
| A_32_P42213 |  | 5.41E-03 | 2.19 | up |
| A_23_P36928 | *POLR1D* | 4.21E-03 | 2.18 | up |
| A_23_P252711 | *LACTB2* | 7.08E-03 | 2.18 | up |
| A_23_P64232 | *ZNF259* | 3.91E-03 | 2.18 | up |
| A_32_P206401 | *QSOX2* | 7.97E-03 | 2.18 | up |
| A_23_P46337 | *C1orf107* | 1.18E-03 | 2.18 | up |
| A_32_P34552 | *POLB* | 3.17E-03 | 2.18 | up |
| A_24_P181672 | *B3GNTL1* | 1.13E-03 | 2.18 | up |
| A_23_P161918 | *CCDC86* | 7.59E-03 | 2.18 | up |
| A_23_P383278 | *PYCRL* | 9.41E-03 | 2.18 | up |
| A_24_P266037 | *DIMT1L* | 2.40E-03 | 2.18 | up |
| A_24_P367397 |  | 5.95E-03 | 2.18 | up |
| A_32_P458096 | *TBC1D24* | 8.58E-03 | 2.18 | up |
| A_23_P157072 | *EIF3B* | 9.49E-03 | 2.18 | up |
| A_24_P38944 | *CCDC86* | 7.33E-03 | 2.17 | up |
| A_23_P170774 | *E2F6* | 1.86E-03 | 2.17 | up |
| A_23_P99927 | *PTPLAD1* | 2.70E-03 | 2.17 | up |
| A_23_P140705 | *C15orf23* | 8.70E-03 | 2.17 | up |
| A_23_P252855 | *BRIX1* | 5.01E-03 | 2.16 | up |
| A_32_P34589 | *RSRC1* | 4.68E-03 | 2.16 | up |
| A_24_P159648 | *BAIAP2* | 6.42E-03 | 2.16 | up |
| A_23_P216355 | *NFKBIL2* | 2.53E-03 | 2.16 | up |
| A_23_P500390 | *DONSON* | 9.33E-03 | 2.16 | up |
| A_23_P131754 | *C20orf195* | 9.98E-03 | 2.16 | up |
| A_24_P346587 | *MTFR1* | 5.71E-03 | 2.16 | up |
| A_24_P376229 | *UBA2* | 1.16E-03 | 2.16 | up |
| A_23_P133146 | *H2AFZ* | 5.91E-03 | 2.15 | up |
| A_32_P12388 |  | 3.37E-03 | 2.15 | up |
| A_23_P7679 | *NUP155* | 8.11E-04 | 2.15 | up |
| A_23_P117623 | *TDP1* | 2.94E-03 | 2.15 | up |
| A_24_P6921 | *LOC541471* | 7.27E-03 | 2.15 | up |
| A_23_P252857 | *BRIX1* | 6.09E-03 | 2.15 | up |
| A_23_P61810 | *BAIAP2* | 7.36E-03 | 2.15 | up |
| A_24_P461389 |  | 6.03E-03 | 2.15 | up |
| A_23_P77049 | *SLC25A29* | 3.49E-03 | 2.15 | up |
| A_32_P8120 | *GNL3* | 5.63E-04 | 2.14 | up |
| A_24_P392271 |  | 3.52E-03 | 2.14 | up |
| A_24_P161525 | *HSP90AB3P* | 4.93E-03 | 2.14 | up |
| A_23_P58953 | *NQO2* | 1.17E-03 | 2.14 | up |
| A_23_P409417 | *VPS37D* | 1.27E-03 | 2.14 | up |
| A_23_P152804 | *NME1* | 2.67E-03 | 2.14 | up |
| A_23_P76761 | *VRK1* | 4.68E-03 | 2.14 | up |
| A_23_P41948 | *CCDC99* | 6.85E-03 | 2.14 | up |
| A_23_P162822 | *EXOSC8* | 1.92E-03 | 2.14 | up |
| A_32_P21993 | *TPM4* | 9.77E-03 | 2.14 | up |
| A_24_P273143 | *NCRNA00152* | 4.97E-03 | 2.14 | up |
| A_23_P80902 | *KIF15* | 3.22E-03 | 2.14 | up |
| A_23_P134247 | *RHEB* | 3.53E-03 | 2.14 | up |
| A_23_P25626 | *C13orf34* | 5.02E-03 | 2.13 | up |
| A_23_P205216 | *UTP14A* | 7.96E-03 | 2.13 | up |
| A_32_P27271 | *E2F6* | 1.72E-03 | 2.13 | up |
| A_32_P147241 | *PKM2* | 3.32E-03 | 2.13 | up |
| A_23_P57836 |  | 5.02E-03 | 2.13 | up |
| A_23_P66608 | *KAT2A* | 4.48E-03 | 2.13 | up |
| A_24_P416301 | *FOXK2* | 5.92E-03 | 2.13 | up |
| A_23_P36464 | *C12orf11* | 2.49E-03 | 2.13 | up |
| A_23_P21436 | *PHF19* | 4.47E-03 | 2.13 | up |
| A_23_P201636 | *LAMC2* | 2.05E-03 | 2.13 | up |
| A_23_P88893 | *DEF8* | 3.56E-03 | 2.13 | up |
| A_23_P258964 | *IARS* | 4.14E-03 | 2.13 | up |
| A_23_P362183 | *ANKS6* | 5.19E-03 | 2.13 | up |
| A_23_P58102 | *EXOSC7* | 6.65E-04 | 2.12 | up |
| A_23_P215525 | *OSBPL3* | 8.97E-03 | 2.12 | up |
| A_23_P68665 | *ADRM1* | 9.44E-03 | 2.12 | up |
| A_24_P99071 | *IPO5* | 2.49E-03 | 2.12 | up |
| A_24_P381029 | *GLRX3* | 2.69E-03 | 2.12 | up |
| A_23_P14083 | *AMIGO2* | 2.11E-03 | 2.12 | up |
| A_24_P110541 |  | 5.37E-03 | 2.12 | up |
| A_23_P333420 | *RANGAP1* | 2.79E-03 | 2.12 | up |
| A_24_P146603 | *DDX21* | 5.51E-03 | 2.12 | up |
| A_23_P143047 | *ATP6V1E2* | 8.93E-03 | 2.12 | up |
| A_23_P165937 | *DSN1* | 7.32E-03 | 2.11 | up |
| A_23_P98042 |  | 5.10E-03 | 2.11 | up |
| A_23_P332439 |  | 3.69E-03 | 2.11 | up |
| A_23_P39116 | *LIG1* | 3.91E-03 | 2.11 | up |
| A_23_P258272 | *SDCCAG3* | 3.60E-03 | 2.11 | up |
| A_23_P50456 | *POLD1* | 3.25E-03 | 2.11 | up |
| A_23_P140738 | *FAM38A* | 1.17E-03 | 2.11 | up |
| A_24_P41570 | *H2AFZ* | 5.42E-03 | 2.11 | up |
| A_24_P124032 | *RIPK2* | 1.54E-03 | 2.11 | up |
| A_24_P305597 | *ADRM1* | 9.51E-03 | 2.11 | up |
| A_23_P51410 | *SMYD3* | 6.81E-03 | 2.11 | up |
| A_23_P55149 | *AP2B1* | 2.85E-03 | 2.11 | up |
| A_23_P55998 | *SLC1A5* | 7.93E-03 | 2.11 | up |
| A_23_P209987 | *POLR1B* | 1.16E-03 | 2.11 | up |
| A_23_P205789 | *GABPB1* | 6.52E-03 | 2.10 | up |
| A_23_P28625 | *WDR12* | 1.48E-03 | 2.10 | up |
| A_23_P43071 | *MTERFD1* | 2.32E-03 | 2.10 | up |
| A_24_P401670 |  | 9.08E-03 | 2.10 | up |
| A_24_P923142 | *ZC3HAV1L* | 2.07E-03 | 2.10 | up |
| A_23_P106998 | *MRPS23* | 3.18E-03 | 2.10 | up |
| A_23_P207400 | *BRCA1* | 4.82E-03 | 2.10 | up |
| A_23_P114232 | *PRDX4* | 7.69E-03 | 2.10 | up |
| A_24_P358305 |  | 9.08E-03 | 2.10 | up |
| A_23_P68717 | *PSMG1* | 4.20E-03 | 2.10 | up |
| A_23_P138426 | *USP6NL* | 4.69E-03 | 2.09 | up |
| A_23_P12292 | *C1orf116* | 4.90E-03 | 2.09 | up |
| A_23_P52082 | *INTS7* | 3.52E-03 | 2.09 | up |
| A_23_P45517 | *PPP2R3B* | 6.19E-03 | 2.09 | up |
| A_24_P339611 | *PDCD5* | 1.88E-03 | 2.09 | up |
| A_24_P684799 |  | 3.69E-03 | 2.09 | up |
| A_24_P711050 |  | 1.72E-03 | 2.09 | up |
| A_24_P317622 | *AGAP3* | 7.14E-03 | 2.09 | up |
| A_24_P161914 |  | 4.78E-03 | 2.09 | up |
| A_23_P93823 | *RFC2* | 3.36E-03 | 2.09 | up |
| A_24_P194000 | *SAE1* | 1.58E-03 | 2.08 | up |
| A_23_P41025 | *GNL3* | 6.52E-04 | 2.08 | up |
| A_23_P1199 | *NUDT5* | 7.78E-03 | 2.08 | up |
| A_24_P243834 | *MTERFD1* | 2.86E-03 | 2.08 | up |
| A_23_P411335 | *SGOL2* | 7.42E-03 | 2.08 | up |
| A_23_P165171 | *TMEM161A* | 3.22E-03 | 2.08 | up |
| A_23_P103628 | *HEATR1* | 1.62E-03 | 2.08 | up |
| A_23_P90612 | *MCM6* | 8.55E-03 | 2.08 | up |
| A_24_P92367 | *YDJC* | 3.99E-03 | 2.08 | up |
| A_24_P706901 |  | 9.51E-03 | 2.08 | up |
| A_23_P106822 | *NOB1* | 1.18E-03 | 2.07 | up |
| A_23_P28420 | *OLA1* | 1.96E-03 | 2.07 | up |
| A_23_P150365 | *REXO2* | 5.86E-03 | 2.07 | up |
| A_24_P305312 | *BBC3* | 6.60E-03 | 2.07 | up |
| A_23_P80839 | *MAP6D1* | 8.54E-03 | 2.06 | up |
| A_23_P155316 | *NCBP2* | 1.48E-03 | 2.06 | up |
| A_24_P359191 | *SLC6A6* | 6.39E-03 | 2.06 | up |
| A_23_P39110 | *RUVBL2* | 3.43E-03 | 2.06 | up |
| A_23_P35099 | *MRTO4* | 8.53E-04 | 2.06 | up |
| A_24_P266035 | *DIMT1L* | 3.03E-03 | 2.06 | up |
| A_23_P320658 | *BUB3* | 7.64E-04 | 2.06 | up |
| A_32_P44568 | *LDHA* | 6.05E-03 | 2.06 | up |
| A_23_P202143 | *NOLC1* | 1.72E-03 | 2.06 | up |
| A_24_P298420 | *PRMT5* | 1.48E-03 | 2.06 | up |
| A_24_P280868 | *FAM86B2* | 5.58E-03 | 2.05 | up |
| A_24_P48177 | *ST3GAL2* | 7.14E-03 | 2.05 | up |
| A_23_P170058 | *PSMB2* | 5.28E-03 | 2.05 | up |
| A_23_P202138 | *SFXN3* | 2.82E-03 | 2.05 | up |
| A_23_P91468 | *PSMA7* | 3.46E-03 | 2.05 | up |
| A_23_P115861 | *ZNF485* | 7.30E-03 | 2.05 | up |
| A_23_P341223 | *KLHL21* | 5.15E-03 | 2.05 | up |
| A_23_P3775 | *OGFOD1* | 1.79E-03 | 2.05 | up |
| A_23_P168629 | *RBM28* | 2.11E-03 | 2.05 | up |
| A_32_P506600 | *RAN* | 3.87E-03 | 2.05 | up |
| A_23_P215341 | *FKBP14* | 4.55E-03 | 2.05 | up |
| A_24_P626931 | *UBE2CBP* | 6.19E-03 | 2.05 | up |
| A_24_P84428 | *CACYBP* | 2.49E-03 | 2.05 | up |
| A_24_P83968 |  | 4.92E-03 | 2.04 | up |
| A_23_P97161 | *TTF2* | 1.10E-03 | 2.04 | up |
| A_23_P89884 | *TRIM28* | 8.21E-03 | 2.04 | up |
| A_23_P110108 | *SLC12A8* | 8.38E-03 | 2.04 | up |
| A_24_P83544 |  | 4.38E-03 | 2.04 | up |
| A_23_P379550 | *YARS* | 8.81E-03 | 2.04 | up |
| A_23_P163992 | *GRB7* | 7.03E-03 | 2.04 | up |
| A_24_P241276 | *EXOSC8* | 2.87E-03 | 2.04 | up |
| A_23_P218827 | *POLQ* | 5.86E-03 | 2.04 | up |
| A_23_P68072 | *WDR54* | 4.68E-03 | 2.04 | up |
| A_23_P158053 | *C9orf16* | 6.00E-03 | 2.04 | up |
| A_23_P50455 | *POLD1* | 4.38E-03 | 2.04 | up |
| A_23_P80098 | *GART* | 6.29E-03 | 2.03 | up |
| A_24_P69439 | *SLC25A32* | 5.58E-03 | 2.03 | up |
| A_24_P179033 |  | 6.01E-03 | 2.03 | up |
| A_23_P87216 | *SLC3A2* | 3.80E-03 | 2.03 | up |
| A_24_P261259 | *PFKFB3* | 4.12E-03 | 2.03 | up |
| A_24_P307126 |  | 5.79E-03 | 2.03 | up |
| A_23_P54597 | *RSL1D1* | 1.16E-03 | 2.03 | up |
| A_23_P65217 | *TGDS* | 1.68E-03 | 2.03 | up |
| A_24_P317135 | *EXOSC7* | 7.33E-04 | 2.02 | up |
| A_23_P366253 | *PATZ1* | 6.63E-03 | 2.02 | up |
| A_24_P261083 | *OSGIN2* | 2.91E-03 | 2.02 | up |
| A_24_P24982 |  | 2.80E-03 | 2.02 | up |
| A_24_P204165 |  | 2.33E-03 | 2.02 | up |
| A_23_P117163 | *RCBTB1* | 3.74E-03 | 2.02 | up |
| A_23_P91900 | *SMC4* | 3.92E-03 | 2.02 | up |
| A_23_P165691 | *PSMD14* | 3.52E-03 | 2.02 | up |
| A_23_P69249 | *ACTL6A* | 1.08E-03 | 2.02 | up |
| A_32_P42574 | *C1orf198* | 1.20E-03 | 2.02 | up |
| A_24_P827491 | *PA2G4* | 3.60E-03 | 2.02 | up |
| A_23_P115149 | *WDR77* | 3.13E-03 | 2.02 | up |
| A_32_P3290 | *HAUS6* | 5.02E-03 | 2.02 | up |
| A_23_P92082 | *TKT* | 9.48E-03 | 2.02 | up |
| A_24_P84698 |  | 7.64E-04 | 2.02 | up |
| A_32_P1614 |  | 5.08E-03 | 2.01 | up |
| A_23_P14432 |  | 6.77E-03 | 2.01 | up |
| A_23_P60899 | *TGS1* | 5.92E-03 | 2.01 | up |
| A_23_P79692 | *ABI2* | 5.81E-03 | 2.01 | up |
| A_23_P119130 | *RPS19* | 2.35E-03 | 2.01 | up |
| A_32_P32923 |  | 2.74E-03 | 2.01 | up |
| A_32_P96134 | *DPY19L1* | 5.20E-03 | 2.01 | up |
| A_23_P151368 | *N6AMT2* | 9.91E-03 | 2.01 | up |
| A_23_P79043 | *TMEM147* | 5.04E-03 | 2.01 | up |
| A_32_P112104 | *SMARCC1* | 5.48E-03 | 2.01 | up |
| A_23_P305981 | *LOC388152* | 4.33E-03 | 2.01 | up |
| A_24_P253251 | *SLC7A1* | 4.97E-03 | 2.01 | up |
| A_24_P418590 |  | 2.92E-03 | 2.00 | up |
| A_24_P219024 | *DIS3* | 2.79E-03 | 2.00 | up |
| A_24_P384411 |  | 3.70E-03 | 2.00 | up |
| A_23_P40347 | *HM13* | 3.43E-03 | 2.00 | up |
| A_23_P123193 | *ACTR3B* | 2.88E-03 | 2.00 | up |
