# Supplementary material for: A Gene Expression and Pre-mRNA Splicing Signature That Marks the Adenoma-Adenocarcinoma Progression in Colorectal Cancer
Source: PLoS One. 2014 Feb 6;9(2):e87761. doi: 10.1371/journal.pone.0087761 (PMC3916340; doi:10.1371/journal.pone.0087761)
Supplement: Table S4 — Validation by quantitative Real-Time Polymerase Chain Reaction. (DOC) [file pone.0087761.s010.doc]

**Table S4. Validation by quantitative Real-Time Polymerase Chain Reaction.** The quantitative RT-PCR assay was performed on groups of at least eight samples, including some of samples hybridized on microarrays, or on an independent set of 14 CRAs and 8 paired tumor-normal CRC samples. Relative mRNA levels were normalized to that of *beta*-2-microglobulin. The cut-off of delta-delta-Ct was equal to 1.5.

| Comparison | Category | Gene Symbol | Probe Name | Microarray Fold-Change | Microarray Regulation | delta-delta-Ct | Validation |
| --- | --- | --- | --- | --- | --- | --- | --- |
| CRA *vs*. NOR | Calcium Signaling Pathway | *PLCE1* | A_23_P35617 | 2.90 | down | 0.29 | yes |
|  | Cell Adhesion Molecules | *CDH3* | A_23_P49155 | 39.62 | up | 7.56 | yes |
|  |  | *CLDN1* | A_23_P57784 | 10.19 | up | 3.11 | yes |
|  |  | *JAM2* | A_23_P120667 | 7.44 | down | 0.52 | yes |
|  |  | *CLDN23* | A_23_P134854 | 6.67 | down | 0.09 | yes |
|  |  | *CD34* | A_32_P220798 | 3.47 | down | 0.64 | yes |
|  |  | *CD40* | A_23_P57036 | 2.64 | down | 0.53 | yes |
|  |  | *ITGB7* | A_23_P76529 | 2.16 | down | 0.05 | yes |
|  |  | *CD8A* | A_32_P163247 | 2.12 | down | 0.08 | yes |
|  |  | *PVRL3* | A_23_P401547 | 2.03 | down | 1.10 | no |
|  | Cell Cycle | *CCNA2* | A_23_P58321 | 2.33 | up | 2.09 | yes |
|  |  | *E2F5* | A_23_P31721 | 2.01 | up | 1.83 | yes |
|  | Cytokine-Cytokine Receptor Interaction | *TNFRSF12A* | A_23_P49338 | 4.31 | up | 1.75 | yes |
|  |  | *TNFRSF10B* | A_23_P169030 | 2.58 | up | 2.47 | yes |
|  |  | *TNFRSF17* | A_23_P37736 | 4.64 | down | 0.31 | yes |
|  | Drug Metabolism | *IMPDH2* | A_24_P166042 | 2.46 | up | 3.70 | yes |
|  |  | *IMPDH1* | A_24_P89701 | 2.45 | up | 0.31 | no |
|  |  | *UGT2B15* | A_23_P58407 | 13.82 | down | 0.24 | yes |
|  |  | *CES1* | A_23_P206733 | 6.43 | down | 0.24 | yes |
|  |  | *CES2* | A_23_P49192 | 5.58 | down | 1.07 | no |
|  | Focal Adhesion | *BCL2* | A_23_P352266 | 2.71 | down | 0.76 | no |
|  |  | *COL1A2* | A_24_P277934 | 2.30 | down | 0.85 | no |
|  | List of 288 Splicing Factors | *SFRS6* | A_23_P109017 | 2.31 | up | 2.13 | yes |
|  |  | *SNRPB* | A_23_P154675 | 2.28 | up | 2.11 | yes |
|  |  | *PPIL1* | A_23_P133995 | 2.16 | up | 5.30 | yes |
|  |  | *NONO* | A_24_P413437 | 2.13 | up | 3.40 | yes |
|  |  | *RBMX* | A_32_P193646 | 2.07 | up | 19.92 | yes |
|  |  | *WDR77* | A_23_P115149 | 2.06 | up | 4.34 | yes |
|  |  | *DDX26B* | A_23_P363647 | 2.50 | down | 1.26 | no |
|  | Other Category | *BACE2* | A_24_P14584 | 4.50 | up | 5.07 | yes |
|  |  | *PKIB* | A_23_P145529 | 7.64 | down | 0.10 | yes |
|  |  | *SEPP1* | A_23_P121926 | 2.95 | down | 0.40 | yes |
|  | p53 Signaling Pathway | *CDK1* | A_23_P138507 | 2.45 | up | 0.40 | no |
|  |  | *CCNB1* | A_23_P122197 | 2.43 | up | 0.30 | no |
|  |  | *BAX* | A_23_P208706 | 2.07 | up | 3.29 | yes |
|  |  | *IGF1* | A_24_P304419 | 4.95 | down | 0.35 | yes |
|  |  | *THBS1* | A_23_P206212 | 2.80 | down | 0.11 | yes |
|  | Signature of 44 entities | *INHBA* | A_23_P122924 | 5.72 | up | 3.35 | yes |
|  |  | *AJUBA* | A_23_P54055 | 4.24 | up | 9.35 | yes |
|  |  | *PSAT1* | A_23_P259692 | 2.47 | up | 6.09 | yes |
|  |  | *SKA3* | A_23_P340909 | 2.15 | up | 3.05 | yes |
|  |  | *TIMP1* | A_23_P62115 | 2.11 | up | 30.41 | yes |
|  |  | *UBE2S* | A_32_P184933 | 2.01 | up | 3.94 | yes |
|  |  | *DPT* | A_23_P200741 | 27.31 | down | 0.004 | yes |
|  |  | *FBLN1* | A_23_P211631 | 13.36 | down | 0.03 | yes |
|  |  | *SLIT3* | A_23_P58588 | 12.05 | down | 1.37 | no |
|  |  | *SCARA5* | A_23_P94103 | 11.54 | down | 0.3 | yes |
|  |  | *CFH* | A_23_P200160 | 8.65 | down | 0.27 | yes |
|  |  | *CRYAB* | A_24_P206776 | 5.69 | down | 0.19 | yes |
|  |  | *ITIH5* | A_23_P411993 | 5.07 | down | 0.24 | yes |
|  |  | *LRRC19* | A_23_P364625 | 3.55 | down | 0.59 | yes |
|  |  | *SMPDL3A* | A_23_P72117 | 3.08 | down | 0.26 | yes |
|  |  | *ISX* | A_32_P217140 | 2.65 | down | 1.29 | no |
|  |  | *HSD11B2* | A_23_P14986 | 2.64 | down | 0.53 | yes |
|  |  | *NR3C2* | A_23_P392470 | 2.58 | down | 0.84 | no |
|  |  | *IGJ* | A_23_P167168 | 2.50 | down | 0.42 | yes |
|  |  | *ASAP3* | A_23_P114689 | 2.37 | down | 1.09 | no |
|  |  | *NXPE4* | A_23_P320216 | 2.25 | down | 0.74 | no |
|  |  | *RDH5* | A_24_P218814 | 2.22 | down | 0.53 | yes |
|  |  | *ITM2C* | A_24_P402690 | 2.08 | down | 1.61 | no |
|  | *Wnt* Signaling Pathway | *MMP7* | A_23_P52761 | 116.11 | up | 40.82 | yes |
|  |  | *TCF7* | A_23_P7582 | 3.56 | up | 16.46 | yes |
|  |  | *PLCB4* | A_23_P28898 | 2.88 | up | 5.15 | yes |
|  |  | *SFRP2* | A_24_P934546 | 34.57 | down | 0.02 | yes |
| CRC *vs*. NOR | List of 288 Splicing Factors | *PPIL1* | A_23_P133995 | 2.41 | up | 11.44 | yes |
|  |  | *PRMT5* | A_24_P298420 | 2.06 | up | 1.99 | yes |
|  |  | *WDR77* | A_23_P115149 | 2.02 | up | 21.8 | yes |
|  |  | *DDX26B* | A_23_P363647 | 3.32 | down | 2.74 | no |
|  | Signature of 44 entities | *INHBA* | A_23_P122924 | 41.06 | up | 13.24 | yes |
|  |  | *AJUBA* | A_23_P54055 | 8.77 | up | 6.83 | yes |
|  |  | *PSAT1* | A_23_P259692 | 6.95 | up | 5.31 | yes |
|  |  | *SKA3* | A_23_P340909 | 4.65 | up | 4.09 | yes |
|  |  | *UBE2S* | A_32_P184933 | 4.56 | up | 3.99 | yes |
|  |  | *TIMP1* | A_23_P62115 | 4.39 | up | 5.57 | yes |
|  |  | *SCARA5* | A_23_P94103 | 36.56 | down | 0.19 | yes |
|  |  | *NXPE4* | A_23_P320216 | 17.45 | down | 0.28 | yes |
|  |  | *LRRC19* | A_23_P364625 | 9.42 | down | 0.26 | yes |
|  |  | *IGJ* | A_23_P167168 | 8.87 | down | 0.75 | no |
|  |  | *ISX* | A_32_P217140 | 8.32 | down | 0.63 | yes |
|  |  | *NR3C2* | A_23_P392470 | 7.53 | down | 0.51 | yes |
|  |  | *SMPDL3A* | A_23_P72117 | 7.07 | down | 0.29 | yes |
|  |  | *HSD11B2* | A_23_P14986 | 7.07 | down | 0.22 | yes |
|  |  | *RDH5* | A_24_P218814 | 6.34 | down | 0.30 | yes |
|  |  | *ITM2C* | A_24_P402690 | 5.62 | down | 0.37 | yes |
|  |  | *DPT* | A_23_P200741 | 5.36 | down | 0.01 | yes |
|  |  | *SLIT3* | A_23_P58588 | 5.19 | down | 1.12 | no |
|  |  | *ASAP3* | A_23_P114689 | 5.03 | down | 0.49 | yes |
|  |  | *CFH* | A_23_P200160 | 3.90 | down | 0.67 | yes |
|  |  | *FBLN1* | A_23_P211631 | 3.20 | down | 2.91 | no |
|  |  | *CRYAB* | A_24_P206776 | 2.72 | down | 2.82 | no |
|  |  | *ITIH5* | A_23_P411993 | 2.40 | down | 1.47 | no |
