# Supplementary material for: A Gene Expression and Pre-mRNA Splicing Signature That Marks the Adenoma-Adenocarcinoma Progression in Colorectal Cancer
Source: PLoS One. 2014 Feb 6;9(2):e87761. doi: 10.1371/journal.pone.0087761 (PMC3916340; doi:10.1371/journal.pone.0087761)
Supplement: Table S5 — Validation by PCR arrays of regulations in colorectal cancer samples in comparison to normal mucosae. (DOC) [file pone.0087761.s011.doc]

**Table S5. Validation by PCR arrays of regulations in colorectal cancer samples in comparison to normal mucosae.** The results of cross validation between 44k Whole Human Genome microarray (Agilent) and PCR array (Qiagen) experiments are presented.

| Gene Symbol | Microarray Probe Name | Microarray Fold-Change | Microarray Regulation | PCR Array Fold-Change | PCR Array P-value | Validation |
| --- | --- | --- | --- | --- | --- | --- |
| *ABCC1* | A_24_P227831 | 2.48 | up | 2.89 | 0.00E+00 | yes |
| *BRCA1* | A_23_P207400 | 2.10 | up | 2.04 | 1.80E-05 | yes |
| *CCND1* | A_24_P193011 | 2.70 | up | 3.73 | 0.00E+00 | yes |
|  | A_24_P124550 | 2.68 | up |  |  |  |
|  | A_23_P202837 | 2.45 | up |  |  |  |
| *CDK4* | A_23_P24997 | 2.44 | up | 2.69 | 0.00E+00 | yes |
| *CXCL16* | A_23_P38505 | 2.30 | up | 2.94 | 0.00E+00 | yes |
| *DHCR7* | A_23_P24444 | 2.80 | up | 3.61 | 1.00E-06 | yes |
| *MMP1* | A_23_P1691 | 26.30 | up | 4.60 | 7.48E-03 | yes |
| *NME1* | A_23_P152804 | 2.14 | up | 4.58 | 0.00E+00 | yes |
| *PCSK9* | A_32_P142440 | 8.68 | up | 5.46 | 6.00E-06 | yes |
| *PKM2* | A_23_P399501 | 2.21 | up | 2.83 | 0.00E+00 | yes |
|  | A_32_P147241 | 2.13 | up |  |  |  |
| *RIPK2* | A_23_P252106 | 3.15 | up | 2.13 | 1.30E-05 | yes |
|  | A_24_P124032 | 2.11 | up |  |  |  |
| *TIMP1* | A_23_P62115 | 4.39 | up | 4.01 | 0.00E+00 | yes |
| *ADH1C* | A_23_P81158 | 34.56 | down | -11.27 | 0.00E+00 | yes |
| *BCL2* | A_23_P352266 | 5.62 | down | -3.18 | 0.00E+00 | yes |
| *CASP7* | A_23_P12572 | 2.66 | down | -2.15 | 0.00E+00 | yes |
| *CD27* | A_23_P48088 | 3.91 | down | -3.00 | 0.00E+00 | yes |
| *FGFR2* | A_23_P202334 | 4.10 | down | -4.29 | 0.00E+00 | yes |
| *FRZB* | A_23_P10902 | 4.24 | down | -2.61 | 8.38E-03 | yes |
| *GPX3* | A_23_P133474 | 4.13 | down | -3.67 | 1.00E-06 | yes |
| *GSTM5* | A_23_P97606 | 3.29 | down | -4.59 | 0.00E+00 | yes |
| *HSD17B2* | A_23_P118065 | 13.56 | down | -5.42 | 0.00E+00 | yes |
| *IGF1* | A_23_P13907 | 6.46 | down | -5.65 | 0.00E+00 | yes |
|  | A_24_P304419 | 5.90 | down |  |  |  |
| *NAIP* | A_23_P110473 | 2.54 | down | -2.28 | 3.00E-06 | yes |
| *OSBPL1A* | A_23_P10442 | 3.44 | down | -2.31 | 0.00E+00 | yes |
| *SFRP1* | A_23_P10127 | 45.53 | down | -21.76 | 0.00E+00 | yes |
|  | A_23_P10121 | 25.52 | down |  |  |  |
| *STAB1* | A_23_P32500 | 4.33 | down | -3.59 | 0.00E+00 | yes |
| *WNT2B* | A_23_P138352 | 2.42 | down | -2.82 | 0.00E+00 | yes |
| *WNT5B* | A_23_P53588 | 3.91 | down | -2.30 | 3.80E-05 | yes |
