# Supplementary material for: A Gene Expression and Pre-mRNA Splicing Signature That Marks the Adenoma-Adenocarcinoma Progression in Colorectal Cancer
Source: PLoS One. 2014 Feb 6;9(2):e87761. doi: 10.1371/journal.pone.0087761 (PMC3916340; doi:10.1371/journal.pone.0087761)
Supplement: Table S6 — List of the up- and down-regulated genes of the gene expression signature of 954 probes. (DOC) [file pone.0087761.s012.doc]

**Table S6. List of the up- and down-regulated genes of the gene expression signature of 954 probes.** The common deregulated genes between both the CRA *vs*. NOR and CRC *vs*. NOR analyses on 44k Whole Human Genome microarrays (Agilent) are presented (≥ 2.0 FC, P-value ≤ 0.01 by *t*-test with FDR). All common genes followed the same type of variation in both comparisons, *i.e*. were up- or down-regulated similarly in CRA and CRC in comparison with NOR.

|  |  | Colorectal Adenoma *vs*. Normal | | | Colorectal Cancer *vs*. Normal | | |
| --- | --- | --- | --- | --- | --- | --- | --- |
| Probe Name | Gene Symbol | P-value | Fold-Change | Regulation | P-value | Fold-Change | Regulation |
| A_23_P168916 | *CA1* | 1.04E-09 | 294.76 | down | 1.05E-03 | 328.30 | down |
| A_23_P63032 | *GUCA2B* | 3.67E-10 | 79.97 | down | 7.54E-03 | 57.01 | down |
| A_23_P11968 | *GUCA2A* | 5.15E-11 | 68.47 | down | 1.37E-03 | 54.18 | down |
| A_23_P4096 | *CA4* | 2.01E-08 | 58.35 | down | 2.50E-03 | 70.36 | down |
| A_23_P10127 | *SFRP1* | 1.78E-12 | 56.63 | down | 4.66E-04 | 45.53 | down |
| A_23_P259863 | *CD177* | 2.25E-08 | 54.58 | down | 2.54E-03 | 31.60 | down |
| A_23_P102611 | *WISP2* | 6.37E-17 | 53.11 | down | 5.51E-04 | 43.97 | down |
| A_23_P123228 | *SLC26A3* | 2.31E-08 | 47.04 | down | 5.10E-03 | 15.74 | down |
| A_32_P143589 | *CD177* | 1.70E-07 | 44.91 | down | 7.42E-03 | 24.04 | down |
| A_24_P40626 | *GREM2* | 2.04E-12 | 44.43 | down | 6.54E-04 | 31.20 | down |
| A_23_P251412 | *SCGN* | 1.84E-10 | 43.96 | down | 6.52E-04 | 45.91 | down |
| A_23_P151895 | *CILP* | 8.95E-20 | 42.73 | down | 1.78E-03 | 12.07 | down |
| A_23_P26522 | *AQP8* | 8.66E-09 | 38.04 | down | 1.93E-03 | 44.01 | down |
| A_32_P157213 |  | 8.23E-09 | 36.14 | down | 1.59E-03 | 24.04 | down |
| A_23_P156708 | *TNXB* | 2.16E-15 | 33.72 | down | 8.07E-04 | 18.23 | down |
| A_23_P105012 | *HRASLS2* | 4.52E-11 | 30.06 | down | 1.40E-03 | 12.25 | down |
| A_23_P10121 | *SFRP1* | 6.26E-11 | 30.04 | down | 4.66E-04 | 25.52 | down |
| A_23_P8812 |  | 1.70E-14 | 29.42 | down | 3.13E-03 | 9.86 | down |
| A_23_P259868 | *CD177* | 1.88E-08 | 28.30 | down | 3.84E-03 | 19.98 | down |
| A_23_P200741 | *DPT* | 6.20E-18 | 27.31 | down | 6.92E-03 | 5.36 | down |
| A_32_P27046 | *CHGA* | 2.04E-14 | 26.40 | down | 8.53E-04 | 25.01 | down |
| A_23_P18672 | *GBA3* | 1.74E-11 | 25.94 | down | 1.38E-03 | 14.13 | down |
| A_23_P13713 | *PRPH* | 1.77E-14 | 25.32 | down | 4.66E-04 | 9.94 | down |
| A_24_P168925 | *CHRDL1* | 3.67E-18 | 24.87 | down | 3.18E-03 | 13.18 | down |
| A_23_P8913 | *CA2* | 6.97E-09 | 24.72 | down | 8.72E-04 | 40.12 | down |
| A_23_P19650 | *VIP* | 5.44E-10 | 24.20 | down | 9.32E-03 | 10.60 | down |
| A_23_P106656 | *CA7* | 1.77E-14 | 23.63 | down | 3.01E-03 | 21.58 | down |
| A_23_P130573 | *CEACAM7* | 1.52E-07 | 22.60 | down | 2.79E-03 | 11.73 | down |
| A_24_P300379 | *PI16* | 4.59E-18 | 22.20 | down | 6.54E-04 | 22.45 | down |
| A_23_P18713 | *ABCG2* | 1.09E-13 | 20.93 | down | 1.27E-03 | 19.31 | down |
| A_23_P4112 | *PYY* | 1.27E-12 | 20.89 | down | 1.54E-03 | 15.44 | down |
| A_24_P364263 | *HRASLS2* | 2.88E-09 | 20.05 | down | 1.86E-03 | 9.43 | down |
| A_32_P358887 | *SLC4A4* | 1.90E-08 | 19.80 | down | 2.14E-03 | 50.33 | down |
| A_23_P436284 | *OSTBETA* | 6.73E-11 | 19.63 | down | 5.34E-03 | 11.90 | down |
| A_23_P140384 | *CTSG* | 6.29E-10 | 17.55 | down | 2.84E-03 | 16.37 | down |
| A_23_P97181 | *GREM2* | 2.36E-15 | 17.02 | down | 5.37E-04 | 25.56 | down |
| A_23_P150457 | *LYVE1* | 4.59E-18 | 16.20 | down | 3.52E-03 | 8.89 | down |
| A_23_P202448 | *CXCL12* | 3.92E-14 | 15.80 | down | 6.52E-04 | 21.03 | down |
| A_23_P58082 | *CCDC80* | 7.18E-14 | 15.11 | down | 5.62E-03 | 3.35 | down |
| A_32_P140139 | *F13A1* | 9.48E-11 | 14.75 | down | 1.38E-03 | 9.67 | down |
| A_23_P250951 | *SLC26A2* | 9.25E-07 | 14.72 | down | 4.61E-03 | 18.93 | down |
| A_24_P360674 | *CDKN2B* | 9.94E-11 | 14.64 | down | 1.34E-03 | 8.25 | down |
| A_23_P212968 | *UGT2B11* | 5.07E-07 | 14.55 | down | 2.26E-03 | 15.84 | down |
| A_24_P17691 | *UGT2B17* | 8.11E-05 | 14.42 | down | 1.20E-03 | 28.26 | down |
| A_23_P58407 | *UGT2B15* | 4.94E-05 | 13.82 | down | 1.20E-03 | 28.94 | down |
| A_23_P256425 | *ADAMDEC1* | 2.51E-14 | 13.63 | down | 1.20E-03 | 13.71 | down |
| A_23_P407695 | *FAM151A* | 2.68E-15 | 13.53 | down | 2.46E-03 | 14.61 | down |
| A_23_P211631 | *FBLN1* | 1.36E-11 | 13.36 | down | 7.13E-03 | 3.20 | down |
| A_23_P69497 | *CLEC3B* | 1.79E-13 | 13.29 | down | 7.64E-04 | 12.34 | down |
| A_24_P643776 |  | 1.45E-14 | 13.22 | down | 1.23E-03 | 7.40 | down |
| A_24_P131622 | *FAM107A* | 2.84E-12 | 13.01 | down | 6.65E-04 | 11.72 | down |
| A_23_P433016 | *FBLN1* | 8.05E-13 | 12.99 | down | 5.53E-03 | 3.40 | down |
| A_23_P58588 | *SLIT3* | 3.42E-14 | 12.05 | down | 8.11E-04 | 5.19 | down |
| A_23_P146134 | *DUSP26* | 1.25E-11 | 11.98 | down | 9.62E-04 | 9.86 | down |
| A_24_P70183 | *MYH11* | 5.00E-08 | 11.65 | down | 2.25E-03 | 6.81 | down |
| A_23_P212050 | *BCHE* | 6.31E-08 | 11.64 | down | 6.54E-04 | 10.96 | down |
| A_23_P39067 | *SPIB* | 5.40E-14 | 11.62 | down | 2.63E-03 | 13.84 | down |
| A_23_P94103 | *SCARA5* | 1.04E-14 | 11.54 | down | 1.11E-03 | 36.56 | down |
| A_23_P303087 | *PTN* | 1.32E-10 | 11.47 | down | 3.65E-03 | 5.29 | down |
| A_23_P164057 | *MFAP4* | 5.90E-10 | 11.43 | down | 4.45E-03 | 4.40 | down |
| A_23_P58359 | *ADH1A* | 1.57E-05 | 11.37 | down | 3.52E-03 | 35.39 | down |
| A_23_P21092 | *CALB2* | 7.65E-12 | 11.32 | down | 4.56E-03 | 5.56 | down |
| A_23_P206920 | *MYH11* | 1.55E-07 | 11.13 | down | 2.87E-03 | 6.19 | down |
| A_24_P395415 |  | 2.61E-06 | 11.06 | down | 1.60E-03 | 28.58 | down |
| A_24_P325992 | *LIFR* | 6.65E-11 | 10.93 | down | 8.53E-04 | 8.41 | down |
| A_23_P146274 | *STMN2* | 1.41E-07 | 10.90 | down | 7.64E-04 | 16.14 | down |
| A_23_P90710 | *DES* | 7.76E-07 | 10.64 | down | 9.73E-03 | 7.94 | down |
| A_24_P291658 | *ADH1A* | 9.46E-06 | 10.60 | down | 2.35E-03 | 25.89 | down |
| A_23_P84860 | *FAM107A* | 3.78E-14 | 10.50 | down | 4.66E-04 | 9.31 | down |
| A_23_P372308 | *RGMA* | 2.78E-12 | 10.36 | down | 3.31E-03 | 4.94 | down |
| A_23_P17438 | *EDN3* | 1.24E-07 | 10.32 | down | 1.96E-03 | 10.83 | down |
| A_32_P197561 | *EBF1* | 2.15E-11 | 10.16 | down | 3.25E-03 | 3.53 | down |
| A_23_P56559 | *DHRS9* | 1.31E-06 | 10.11 | down | 2.48E-03 | 16.64 | down |
| A_23_P338919 | *SPEG* | 1.02E-06 | 9.63 | down | 1.72E-03 | 3.89 | down |
| A_24_P870620 | *PTN* | 1.88E-11 | 9.60 | down | 3.18E-03 | 4.90 | down |
| A_23_P369994 | *DCLK1* | 2.38E-20 | 9.57 | down | 2.24E-03 | 9.17 | down |
| A_23_P163336 | *CA12* | 6.16E-11 | 9.54 | down | 1.48E-03 | 10.88 | down |
| A_23_P81158 | *ADH1C* | 5.12E-05 | 9.37 | down | 3.46E-03 | 34.56 | down |
| A_32_P48256 |  | 2.83E-10 | 9.28 | down | 3.66E-03 | 7.44 | down |
| A_23_P164436 | *ASPA* | 2.76E-13 | 9.22 | down | 1.49E-03 | 10.56 | down |
| A_23_P103256 | *CFHR3* | 8.67E-16 | 9.06 | down | 5.95E-03 | 4.02 | down |
| A_32_P169353 |  | 1.62E-05 | 8.83 | down | 6.54E-03 | 13.60 | down |
| A_23_P399265 | *STMN2* | 3.52E-07 | 8.78 | down | 5.25E-04 | 10.64 | down |
| A_23_P200160 | *CFH* | 3.65E-11 | 8.65 | down | 3.43E-03 | 3.90 | down |
| A_23_P41528 | *FGFBP2* | 3.47E-14 | 8.64 | down | 6.76E-03 | 4.94 | down |
| A_23_P103812 | *FAM5C* | 4.11E-08 | 8.47 | down | 7.27E-03 | 11.70 | down |
| A_23_P217269 | *VSIG4* | 3.01E-10 | 8.43 | down | 2.61E-03 | 2.65 | down |
| A_23_P15450 | *TMEM100* | 1.75E-13 | 8.36 | down | 8.51E-04 | 11.32 | down |
| A_23_P56197 | *CRLF1* | 1.62E-10 | 8.35 | down | 8.88E-03 | 2.88 | down |
| A_23_P7342 | *UGT2B10* | 5.37E-07 | 8.29 | down | 3.52E-03 | 10.95 | down |
| A_23_P107116 | *RNF112* | 3.21E-14 | 8.12 | down | 3.17E-03 | 6.12 | down |
| A_23_P30614 | *PLN* | 2.07E-07 | 8.08 | down | 3.37E-03 | 2.61 | down |
| A_23_P420442 | *SEMA6D* | 2.31E-08 | 8.03 | down | 5.58E-03 | 7.29 | down |
| A_23_P96383 | *SRPX* | 3.78E-14 | 8.00 | down | 2.64E-03 | 5.33 | down |
| A_32_P310335 | *JAM2* | 5.95E-13 | 7.93 | down | 3.84E-03 | 6.49 | down |
| A_23_P153390 | *CLEC4G* | 1.45E-14 | 7.92 | down | 4.43E-03 | 6.01 | down |
| A_23_P96501 | *TEX11* | 1.54E-09 | 7.81 | down | 2.48E-03 | 10.20 | down |
| A_24_P8116 | *CCDC80* | 7.49E-19 | 7.80 | down | 9.41E-03 | 3.12 | down |
| A_23_P145529 | *PKIB* | 6.31E-06 | 7.64 | down | 1.04E-03 | 39.74 | down |
| A_23_P136753 |  | 8.92E-06 | 7.61 | down | 1.41E-03 | 9.28 | down |
| A_23_P17130 | *C2orf88* | 3.83E-10 | 7.58 | down | 2.55E-03 | 15.13 | down |
| A_23_P125233 | *CNN1* | 1.79E-07 | 7.49 | down | 8.98E-03 | 2.68 | down |
| A_24_P709844 |  | 6.74E-09 | 7.46 | down | 7.86E-04 | 7.98 | down |
| A_23_P106773 | *SULT1A2* | 3.96E-09 | 7.45 | down | 2.63E-03 | 8.08 | down |
| A_23_P120667 | *JAM2* | 2.06E-11 | 7.44 | down | 4.78E-03 | 3.70 | down |
| A_23_P209232 | *CLIP4* | 9.05E-10 | 7.31 | down | 2.46E-03 | 3.62 | down |
| A_23_P144348 | *SLIT2* | 5.33E-10 | 7.30 | down | 1.11E-03 | 5.37 | down |
| A_24_P20292 | *B3GNT7* | 7.18E-05 | 7.15 | down | 7.16E-03 | 13.43 | down |
| A_23_P17134 | *MAL* | 4.64E-11 | 7.14 | down | 5.25E-04 | 10.77 | down |
| A_24_P56240 | *CPNE8* | 3.68E-08 | 7.10 | down | 4.87E-03 | 5.69 | down |
| A_23_P51787 | *AMPD1* | 3.78E-07 | 7.09 | down | 6.15E-03 | 15.06 | down |
| A_23_P26511 | *GDPD3* | 3.55E-08 | 7.05 | down | 4.36E-03 | 7.98 | down |
| A_23_P426305 | *AOC3* | 1.79E-13 | 6.96 | down | 3.28E-03 | 4.31 | down |
| A_32_P44878 | *ITIH5* | 2.43E-16 | 6.90 | down | 9.40E-03 | 4.03 | down |
| A_24_P71904 | *HPGD* | 3.12E-07 | 6.78 | down | 5.67E-03 | 4.99 | down |
| A_24_P164998 | *LOC646627* | 2.86E-06 | 6.78 | down | 7.31E-03 | 13.02 | down |
| A_23_P216361 | *COL14A1* | 2.72E-09 | 6.77 | down | 1.58E-03 | 3.20 | down |
| A_24_P165864 | *P2RY14* | 1.03E-09 | 6.76 | down | 5.37E-04 | 6.46 | down |
| A_32_P106615 |  | 7.90E-10 | 6.75 | down | 1.77E-03 | 6.02 | down |
| A_23_P86461 | *PLAC9* | 1.38E-17 | 6.73 | down | 3.52E-03 | 5.73 | down |
| A_24_P379413 | *IL6R* | 1.52E-08 | 6.72 | down | 5.25E-04 | 5.39 | down |
| A_23_P134854 | *CLDN23* | 1.80E-13 | 6.67 | down | 9.62E-04 | 6.99 | down |
| A_32_P53524 | *NTN1* | 7.66E-09 | 6.66 | down | 9.00E-04 | 5.90 | down |
| A_32_P171225 |  | 1.78E-08 | 6.66 | down | 1.45E-03 | 4.85 | down |
| A_23_P68669 | *CHODL* | 6.90E-09 | 6.56 | down | 4.66E-04 | 6.62 | down |
| A_23_P39294 | *PLAC2* | 7.49E-06 | 6.54 | down | 2.69E-03 | 8.59 | down |
| A_23_P94434 | *HRCT1* | 6.83E-07 | 6.46 | down | 9.44E-03 | 3.83 | down |
| A_32_P11262 |  | 2.69E-08 | 6.46 | down | 3.28E-03 | 11.06 | down |
| A_32_P136295 | *GNG7* | 2.45E-09 | 6.45 | down | 4.29E-03 | 7.92 | down |
| A_23_P310094 | *SYNPO2* | 5.30E-11 | 6.44 | down | 3.84E-03 | 5.20 | down |
| A_23_P66637 | *SGCA* | 1.89E-12 | 6.41 | down | 6.54E-04 | 5.82 | down |
| A_23_P210158 |  | 9.52E-07 | 6.37 | down | 7.64E-04 | 5.03 | down |
| A_23_P162668 | *CPM* | 1.27E-09 | 6.33 | down | 1.96E-03 | 5.41 | down |
| A_23_P118065 | *HSD17B2* | 2.08E-06 | 6.33 | down | 1.96E-03 | 13.56 | down |
| A_23_P10506 | *HPGDS* | 2.32E-08 | 6.29 | down | 6.52E-04 | 7.26 | down |
| A_23_P360964 | *DACT3* | 3.82E-12 | 6.29 | down | 9.46E-03 | 2.30 | down |
| A_23_P256470 | *NPY* | 1.81E-12 | 6.26 | down | 1.34E-03 | 4.69 | down |
| A_23_P119562 | *CFD* | 2.72E-05 | 6.19 | down | 9.85E-04 | 8.88 | down |
| A_23_P113351 | *SPARCL1* | 2.28E-11 | 6.11 | down | 6.48E-03 | 3.01 | down |
| A_23_P158297 | *BTNL3* | 2.56E-05 | 6.01 | down | 8.21E-03 | 12.22 | down |
| A_24_P938352 | *CPM* | 5.88E-09 | 6.00 | down | 1.44E-03 | 6.66 | down |
| A_23_P156890 | *TCF21* | 3.01E-10 | 5.96 | down | 3.57E-03 | 5.71 | down |
| A_23_P111583 | *CD36* | 5.23E-11 | 5.95 | down | 3.01E-03 | 6.93 | down |
| A_23_P12363 | *ROR1* | 9.55E-09 | 5.94 | down | 8.50E-03 | 3.79 | down |
| A_23_P218858 | *ABI3BP* | 5.29E-12 | 5.85 | down | 4.98E-03 | 10.31 | down |
| A_23_P110319 | *CWH43* | 1.03E-03 | 5.79 | down | 4.97E-03 | 22.44 | down |
| A_32_P76137 |  | 6.22E-04 | 5.78 | down | 3.23E-03 | 6.65 | down |
| A_23_P133474 | *GPX3* | 8.85E-17 | 5.75 | down | 8.93E-03 | 4.13 | down |
| A_24_P233786 | *FAM129A* | 1.31E-09 | 5.75 | down | 2.87E-03 | 3.44 | down |
| A_23_P55738 | *CEACAM1* | 3.61E-06 | 5.70 | down | 8.93E-03 | 4.72 | down |
| A_24_P206776 | *CRYAB* | 2.31E-15 | 5.69 | down | 2.93E-03 | 2.72 | down |
| A_23_P142560 | *ZEB2* | 2.05E-13 | 5.68 | down | 4.14E-03 | 3.30 | down |
| A_32_P32413 | *SETBP1* | 3.79E-09 | 5.66 | down | 5.58E-03 | 3.33 | down |
| A_24_P382319 | *CEACAM1* | 2.55E-08 | 5.65 | down | 6.36E-03 | 3.65 | down |
| A_23_P70719 | *LAMA2* | 5.43E-08 | 5.59 | down | 2.89E-03 | 2.17 | down |
| A_23_P250102 | *CAND2* | 5.45E-10 | 5.58 | down | 2.05E-03 | 4.27 | down |
| A_32_P78681 | *GLP2R* | 9.14E-09 | 5.53 | down | 5.25E-04 | 14.59 | down |
| A_23_P418234 | *PHLPP2* | 1.28E-12 | 5.51 | down | 1.01E-03 | 5.19 | down |
| A_23_P21907 |  | 3.14E-08 | 5.48 | down | 1.07E-03 | 7.04 | down |
| A_23_P434118 | *CEACAM1* | 2.34E-06 | 5.47 | down | 9.48E-03 | 4.70 | down |
| A_32_P208403 | *GNG2* | 6.89E-13 | 5.44 | down | 1.53E-03 | 2.95 | down |
| A_23_P4773 | *LILRB5* | 5.38E-09 | 5.38 | down | 4.66E-04 | 6.00 | down |
| A_24_P263786 |  | 3.74E-03 | 5.38 | down | 1.96E-03 | 13.77 | down |
| A_23_P41987 | *GFRA3* | 1.83E-09 | 5.38 | down | 1.12E-03 | 4.53 | down |
| A_23_P13907 | *IGF1* | 2.17E-11 | 5.37 | down | 6.52E-04 | 6.46 | down |
| A_23_P141505 | *CLEC10A* | 1.02E-10 | 5.33 | down | 1.16E-03 | 6.72 | down |
| A_24_P104407 | *SYNM* | 1.74E-10 | 5.32 | down | 7.45E-03 | 4.18 | down |
| A_23_P117662 | *HDC* | 3.38E-06 | 5.27 | down | 5.95E-03 | 4.27 | down |
| A_32_P213418 |  | 7.04E-10 | 5.25 | down | 4.21E-03 | 2.86 | down |
| A_23_P95640 | *C1orf186* | 2.40E-09 | 5.21 | down | 2.32E-03 | 6.82 | down |
| A_32_P46594 | *LOC145837* | 4.52E-05 | 5.17 | down | 2.95E-03 | 8.17 | down |
| A_23_P371495 | *TMTC1* | 1.30E-10 | 5.15 | down | 1.94E-03 | 2.73 | down |
| A_24_P357465 | *TP53INP2* | 2.83E-09 | 5.11 | down | 1.18E-03 | 5.05 | down |
| A_23_P4551 | *SETBP1* | 5.86E-08 | 5.09 | down | 6.54E-04 | 3.60 | down |
| A_24_P16004 |  | 2.55E-03 | 5.09 | down | 9.31E-03 | 7.42 | down |
| A_24_P152845 | *LOC340888* | 5.13E-05 | 5.09 | down | 3.80E-03 | 10.59 | down |
| A_23_P135548 | *DPYD* | 1.01E-11 | 5.07 | down | 2.40E-03 | 2.67 | down |
| A_23_P411993 | *ITIH5* | 3.16E-12 | 5.07 | down | 6.28E-03 | 2.40 | down |
| A_23_P66854 | *KRT20* | 2.31E-06 | 5.05 | down | 4.12E-03 | 7.71 | down |
| A_23_P83028 | *RECK* | 2.30E-10 | 5.05 | down | 3.51E-03 | 2.97 | down |
| A_23_P19673 | *SGK1* | 3.88E-08 | 5.05 | down | 5.02E-03 | 3.65 | down |
| A_23_P170830 |  | 4.66E-03 | 5.04 | down | 5.01E-03 | 5.61 | down |
| A_32_P46214 | *SLC9A9* | 4.70E-11 | 5.04 | down | 8.53E-04 | 4.96 | down |
| A_24_P538459 |  | 1.33E-03 | 5.03 | down | 4.50E-03 | 7.77 | down |
| A_23_P27005 | *DHRS11* | 8.19E-12 | 5.02 | down | 2.11E-03 | 5.89 | down |
| A_24_P384604 |  | 1.15E-03 | 4.99 | down | 7.69E-03 | 6.53 | down |
| A_23_P138352 | *WNT2B* | 4.32E-11 | 4.96 | down | 5.73E-03 | 2.42 | down |
| A_24_P304419 | *IGF1* | 6.66E-11 | 4.95 | down | 1.77E-03 | 5.89 | down |
| A_24_P177844 |  | 1.60E-05 | 4.94 | down | 5.95E-03 | 7.75 | down |
| A_23_P136724 | *LOC344887* | 9.40E-08 | 4.93 | down | 4.08E-03 | 7.31 | down |
| A_23_P105144 | *SCUBE2* | 8.15E-05 | 4.93 | down | 2.66E-03 | 11.14 | down |
| A_23_P85240 | *TLR7* | 2.46E-09 | 4.92 | down | 2.74E-03 | 4.47 | down |
| A_24_P272310 | *MUSTN1* | 6.76E-10 | 4.92 | down | 2.26E-03 | 4.52 | down |
| A_23_P416774 | *CLIC5* | 1.56E-09 | 4.92 | down | 3.52E-03 | 4.46 | down |
| A_23_P45324 | *TMEM35* | 4.47E-05 | 4.87 | down | 1.16E-03 | 6.46 | down |
| A_23_P52425 | *NKX2-3* | 8.00E-09 | 4.86 | down | 2.76E-03 | 5.49 | down |
| A_23_P115417 | *RGL1* | 6.17E-11 | 4.85 | down | 1.20E-03 | 2.66 | down |
| A_23_P212354 | *CCR2* | 6.25E-07 | 4.84 | down | 4.50E-03 | 4.19 | down |
| A_23_P356494 | *SPINK5* | 6.63E-04 | 4.83 | down | 3.83E-03 | 10.59 | down |
| A_24_P592400 | *LOC553137* | 2.86E-07 | 4.81 | down | 4.82E-03 | 7.65 | down |
| A_23_P1904 | *MS4A2* | 5.33E-08 | 4.81 | down | 8.54E-03 | 5.33 | down |
| A_24_P548866 | *HIGD1A* | 2.13E-06 | 4.81 | down | 4.66E-04 | 4.98 | down |
| A_23_P61466 | *CD163L1* | 3.83E-10 | 4.77 | down | 6.54E-04 | 7.96 | down |
| A_23_P127911 | *PAMR1* | 2.87E-08 | 4.76 | down | 2.13E-03 | 2.89 | down |
| A_23_P97841 | *ITIH5* | 1.28E-12 | 4.74 | down | 2.74E-03 | 3.13 | down |
| A_24_P503710 | *TLCD2* | 1.36E-07 | 4.72 | down | 1.53E-03 | 3.89 | down |
| A_23_P93641 | *AKR1B10* | 3.84E-04 | 4.72 | down | 4.02E-03 | 12.21 | down |
| A_23_P48585 | *SALL2* | 1.24E-11 | 4.71 | down | 4.42E-03 | 3.19 | down |
| A_23_P66017 | *PRRT2* | 1.78E-08 | 4.70 | down | 2.80E-03 | 3.54 | down |
| A_24_P11825 | *CCR2* | 2.98E-06 | 4.68 | down | 9.02E-03 | 3.90 | down |
| A_24_P80204 | *MALL* | 1.85E-06 | 4.67 | down | 6.88E-03 | 3.82 | down |
| A_24_P163237 | *STOX2* | 3.37E-07 | 4.66 | down | 1.48E-03 | 4.49 | down |
| A_23_P85441 | *IGSF9* | 1.15E-07 | 4.65 | down | 1.59E-03 | 4.01 | down |
| A_23_P37736 | *TNFRSF17* | 1.32E-03 | 4.64 | down | 3.13E-03 | 15.18 | down |
| A_24_P129341 | *AKR1B10* | 5.26E-04 | 4.62 | down | 3.95E-03 | 12.16 | down |
| A_24_P204374 |  | 2.79E-03 | 4.60 | down | 3.09E-03 | 5.04 | down |
| A_23_P303833 | *SCN4B* | 3.83E-10 | 4.59 | down | 8.51E-04 | 6.21 | down |
| A_23_P17481 | *SIGLEC1* | 9.20E-13 | 4.57 | down | 8.19E-03 | 3.83 | down |
| A_23_P257003 | *PCSK5* | 2.75E-10 | 4.57 | down | 4.38E-03 | 3.05 | down |
| A_23_P211039 | *ADAMTS1* | 9.72E-08 | 4.57 | down | 5.49E-03 | 2.73 | down |
| A_24_P234768 | *HTR4* | 3.99E-05 | 4.53 | down | 7.01E-03 | 12.49 | down |
| A_23_P54918 | *LDHD* | 1.65E-04 | 4.51 | down | 1.20E-03 | 11.24 | down |
| A_23_P337658 | *ALPI* | 1.72E-08 | 4.50 | down | 1.17E-03 | 3.69 | down |
| A_23_P69810 | *AGPAT9* | 1.48E-08 | 4.50 | down | 9.73E-03 | 5.02 | down |
| A_24_P1054 | *NFKBIL2* | 2.12E-04 | 4.50 | down | 4.31E-03 | 6.24 | down |
| A_23_P72697 | *GPIHBP1* | 2.79E-10 | 4.47 | down | 9.29E-03 | 2.53 | down |
| A_23_P85140 | *TCEAL2* | 5.34E-07 | 4.47 | down | 1.47E-03 | 3.05 | down |
| A_23_P106405 | *NDN* | 3.08E-05 | 4.46 | down | 2.96E-03 | 2.51 | down |
| A_32_P218707 |  | 2.70E-06 | 4.45 | down | 5.35E-03 | 3.86 | down |
| A_23_P84084 | *GFRA2* | 4.06E-10 | 4.43 | down | 6.63E-03 | 5.24 | down |
| A_23_P110624 | *CTNND2* | 1.86E-07 | 4.34 | down | 2.85E-03 | 4.02 | down |
| A_32_P156851 | *RCAN2* | 2.79E-07 | 4.32 | down | 7.64E-04 | 5.95 | down |
| A_23_P138524 | *CPXM2* | 5.04E-05 | 4.32 | down | 7.50E-03 | 2.15 | down |
| A_23_P213050 | *HPGD* | 3.55E-07 | 4.31 | down | 1.20E-03 | 6.55 | down |
| A_24_P746314 |  | 1.21E-09 | 4.30 | down | 8.52E-03 | 2.48 | down |
| A_24_P637651 |  | 1.85E-07 | 4.29 | down | 1.38E-03 | 3.40 | down |
| A_23_P168388 | *GIMAP8* | 2.80E-11 | 4.29 | down | 5.47E-03 | 2.77 | down |
| A_32_P133072 | *SPON1* | 1.79E-03 | 4.28 | down | 1.16E-03 | 8.88 | down |
| A_23_P401106 | *PDE2A* | 8.53E-10 | 4.28 | down | 8.99E-03 | 2.96 | down |
| A_23_P47709 | *FOLR2* | 2.12E-08 | 4.27 | down | 1.70E-03 | 4.97 | down |
| A_23_P320739 | *MEF2C* | 1.32E-07 | 4.26 | down | 9.34E-03 | 2.95 | down |
| A_24_P600036 |  | 4.77E-07 | 4.26 | down | 2.85E-03 | 2.08 | down |
| A_23_P6818 | *SEMA3G* | 2.74E-08 | 4.26 | down | 1.20E-03 | 2.89 | down |
| A_32_P164477 |  | 2.28E-07 | 4.26 | down | 3.67E-03 | 2.96 | down |
| A_23_P16225 | *BEST2* | 4.59E-03 | 4.25 | down | 1.11E-03 | 20.25 | down |
| A_23_P209700 | *NMUR1* | 6.99E-07 | 4.25 | down | 8.25E-03 | 3.60 | down |
| A_23_P4536 | *EPB41L3* | 1.93E-05 | 4.25 | down | 1.36E-03 | 4.27 | down |
| A_32_P228618 | *RBMS3* | 5.52E-10 | 4.24 | down | 3.15E-03 | 3.49 | down |
| A_24_P190804 | *AP1S2* | 1.79E-13 | 4.23 | down | 4.29E-03 | 2.26 | down |
| A_24_P920447 | *C14orf132* | 1.83E-09 | 4.22 | down | 3.75E-03 | 2.82 | down |
| A_23_P201808 | *PPAP2B* | 8.31E-11 | 4.22 | down | 9.62E-04 | 3.66 | down |
| A_23_P151805 | *FBLN5* | 2.32E-10 | 4.22 | down | 5.55E-03 | 2.31 | down |
| A_23_P213857 | *C7* | 1.54E-09 | 4.21 | down | 8.09E-03 | 3.62 | down |
| A_32_P48279 |  | 2.38E-09 | 4.18 | down | 2.94E-03 | 4.41 | down |
| A_32_P74477 |  | 1.03E-13 | 4.18 | down | 3.22E-03 | 3.67 | down |
| A_24_P68908 | *LOC344887* | 1.20E-06 | 4.15 | down | 8.39E-03 | 6.21 | down |
| A_23_P311895 | *CLIC5* | 1.50E-08 | 4.15 | down | 1.86E-03 | 4.51 | down |
| A_23_P216596 | *SVEP1* | 6.76E-10 | 4.14 | down | 9.56E-04 | 3.16 | down |
| A_23_P97606 | *GSTM5* | 1.24E-07 | 4.13 | down | 8.72E-04 | 3.29 | down |
| A_23_P104804 | *ZBTB16* | 2.26E-06 | 4.12 | down | 9.56E-04 | 3.91 | down |
| A_32_P2452 | *TMTC1* | 5.45E-10 | 4.08 | down | 1.12E-03 | 2.87 | down |
| A_23_P201747 | *PADI2* | 2.40E-04 | 4.07 | down | 1.18E-03 | 11.51 | down |
| A_23_P4069 | *TMEM220* | 2.71E-04 | 4.07 | down | 1.27E-03 | 5.83 | down |
| A_23_P171074 | *ITM2A* | 2.65E-05 | 4.05 | down | 3.18E-03 | 4.27 | down |
| A_23_P19624 | *BMP6* | 2.18E-06 | 4.05 | down | 2.53E-03 | 3.88 | down |
| A_24_P925505 | *CD36* | 1.71E-08 | 4.03 | down | 3.84E-03 | 4.86 | down |
| A_23_P131899 | *SDCBP2* | 2.01E-06 | 4.02 | down | 4.33E-03 | 5.21 | down |
| A_23_P151529 | *C14orf132* | 1.79E-09 | 4.00 | down | 4.99E-03 | 3.14 | down |
| A_24_P355246 | *PCSK5* | 1.20E-10 | 4.00 | down | 6.46E-03 | 2.61 | down |
| A_23_P120125 | *COLEC11* | 2.26E-06 | 3.98 | down | 7.64E-04 | 3.31 | down |
| A_23_P87379 | *PDE2A* | 1.95E-09 | 3.96 | down | 9.41E-03 | 2.48 | down |
| A_23_P120902 | *LGALS2* | 7.04E-03 | 3.96 | down | 3.91E-03 | 12.48 | down |
| A_23_P114185 | *TSPAN7* | 7.24E-06 | 3.95 | down | 5.21E-03 | 9.86 | down |
| A_32_P230736 | *LOC389033* | 4.41E-06 | 3.94 | down | 6.52E-04 | 2.43 | down |
| A_32_P123527 |  | 2.67E-11 | 3.92 | down | 1.92E-03 | 3.44 | down |
| A_23_P397376 | *MAF* | 2.24E-11 | 3.92 | down | 9.07E-03 | 2.72 | down |
| A_23_P394395 | *JPH2* | 3.15E-12 | 3.91 | down | 4.97E-03 | 2.58 | down |
| A_32_P30075 |  | 2.53E-03 | 3.91 | down | 7.44E-03 | 5.45 | down |
| A_23_P112452 | *GGTA1* | 8.11E-09 | 3.89 | down | 7.64E-04 | 5.88 | down |
| A_23_P42969 | *FGL2* | 2.16E-09 | 3.88 | down | 1.05E-03 | 3.78 | down |
| A_23_P434212 | *SULT1A1* | 5.86E-07 | 3.83 | down | 2.87E-03 | 4.07 | down |
| A_32_P52785 | *DAAM2* | 2.27E-11 | 3.82 | down | 2.95E-03 | 3.27 | down |
| A_23_P96590 | *GPRASP1* | 3.34E-08 | 3.81 | down | 2.74E-03 | 2.91 | down |
| A_24_P15550 |  | 4.38E-03 | 3.79 | down | 7.67E-03 | 6.31 | down |
| A_24_P321525 | *RERG* | 1.90E-15 | 3.78 | down | 4.32E-03 | 2.21 | down |
| A_23_P85008 | *MAOB* | 5.36E-07 | 3.78 | down | 2.67E-03 | 5.26 | down |
| A_24_P132383 | *GIMAP8* | 6.77E-10 | 3.77 | down | 5.60E-03 | 3.06 | down |
| A_23_P61042 | *IGHA2* | 7.04E-03 | 3.77 | down | 3.43E-03 | 21.85 | down |
| A_23_P205370 | *ASB2* | 8.33E-07 | 3.76 | down | 1.48E-03 | 4.87 | down |
| A_32_P118942 |  | 7.18E-05 | 3.76 | down | 8.93E-03 | 4.50 | down |
| A_23_P201940 | *LMOD1* | 1.09E-12 | 3.76 | down | 1.12E-03 | 3.21 | down |
| A_23_P325690 | *ANKRD35* | 9.21E-10 | 3.75 | down | 7.81E-03 | 2.48 | down |
| A_23_P103765 | *FCER1A* | 9.41E-05 | 3.72 | down | 4.45E-03 | 5.15 | down |
| A_23_P401084 | *ZNF575* | 2.57E-08 | 3.72 | down | 5.19E-03 | 3.53 | down |
| A_23_P372848 | *P2RX1* | 1.22E-06 | 3.72 | down | 3.01E-03 | 4.94 | down |
| A_23_P163251 | *PAQR5* | 2.22E-06 | 3.71 | down | 2.16E-03 | 3.76 | down |
| A_32_P722809 |  | 5.20E-03 | 3.70 | down | 9.41E-03 | 5.51 | down |
| A_23_P73787 | *NUDT10* | 1.33E-06 | 3.69 | down | 3.12E-03 | 2.86 | down |
| A_23_P10980 | *LPHN3* | 4.82E-06 | 3.68 | down | 8.01E-03 | 6.06 | down |
| A_23_P329353 | *CNRIP1* | 1.61E-09 | 3.66 | down | 8.89E-03 | 2.41 | down |
| A_24_P101282 |  | 2.83E-04 | 3.64 | down | 9.99E-03 | 2.81 | down |
| A_32_P48536 |  | 2.76E-06 | 3.63 | down | 1.05E-03 | 3.89 | down |
| A_23_P119006 |  | 6.22E-09 | 3.62 | down | 6.74E-03 | 4.99 | down |
| A_23_P112957 |  | 3.11E-03 | 3.62 | down | 7.48E-03 | 5.66 | down |
| A_24_P31275 | *ATP1B2* | 2.56E-08 | 3.60 | down | 9.56E-04 | 2.94 | down |
| A_24_P243749 | *PDK4* | 3.03E-06 | 3.58 | down | 4.18E-03 | 6.99 | down |
| A_23_P415021 | *METTL7A* | 6.33E-08 | 3.58 | down | 1.86E-03 | 7.12 | down |
| A_23_P32500 | *STAB1* | 1.71E-08 | 3.57 | down | 9.62E-04 | 4.33 | down |
| A_23_P372234 | *CA12* | 3.56E-05 | 3.56 | down | 2.88E-03 | 5.32 | down |
| A_23_P364625 | *LRRC19* | 1.38E-07 | 3.55 | down | 6.01E-03 | 9.42 | down |
| A_32_P194423 |  | 2.41E-04 | 3.55 | down | 6.28E-03 | 5.98 | down |
| A_32_P227870 | *SLC30A4* | 2.64E-09 | 3.55 | down | 4.92E-03 | 3.12 | down |
| A_23_P25187 | *KRT81* | 6.05E-03 | 3.52 | down | 7.30E-03 | 4.44 | down |
| A_24_P187970 | *PADI2* | 1.07E-04 | 3.52 | down | 4.17E-03 | 5.31 | down |
| A_32_P110390 | *TMEM171* | 6.39E-07 | 3.52 | down | 1.54E-03 | 3.81 | down |
| A_23_P217326 | *FHL1* | 5.13E-08 | 3.52 | down | 8.54E-04 | 5.04 | down |
| A_23_P206585 | *PRKCB* | 1.54E-05 | 3.51 | down | 7.60E-03 | 3.52 | down |
| A_23_P202683 | *CDHR5* | 2.20E-06 | 3.51 | down | 1.77E-03 | 6.91 | down |
| A_24_P72518 | *AHCYL2* | 7.66E-07 | 3.50 | down | 2.94E-03 | 6.21 | down |
| A_32_P167631 |  | 2.78E-03 | 3.50 | down | 4.67E-03 | 5.04 | down |
| A_23_P42746 | *NCF1* | 1.08E-06 | 3.50 | down | 6.05E-03 | 2.79 | down |
| A_32_P100379 | *PDGFRA* | 7.76E-07 | 3.48 | down | 4.54E-03 | 2.34 | down |
| A_23_P118025 | *DPEP2* | 1.65E-08 | 3.48 | down | 6.43E-03 | 2.53 | down |
| A_24_P646168 |  | 3.58E-10 | 3.48 | down | 1.96E-03 | 3.62 | down |
| A_23_P344531 | *SYNPO* | 8.52E-09 | 3.47 | down | 3.22E-03 | 3.58 | down |
| A_23_P104741 | *KIRREL3* | 1.85E-07 | 3.47 | down | 2.10E-03 | 4.55 | down |
| A_24_P116700 | *TMEM220* | 1.40E-04 | 3.47 | down | 1.42E-03 | 6.87 | down |
| A_23_P116898 | *A2M* | 7.69E-07 | 3.47 | down | 1.80E-03 | 2.93 | down |
| A_24_P222655 | *C1QA* | 8.11E-09 | 3.46 | down | 5.48E-03 | 2.40 | down |
| A_23_P19142 | *KCNMB1* | 3.75E-08 | 3.44 | down | 6.52E-03 | 2.60 | down |
| A_23_P98910 | *LRMP* | 5.12E-06 | 3.43 | down | 1.47E-03 | 6.19 | down |
| A_23_P159163 |  | 5.12E-03 | 3.43 | down | 8.84E-03 | 4.99 | down |
| A_24_P32935 | *FOLR2* | 8.23E-09 | 3.42 | down | 1.18E-03 | 3.81 | down |
| A_24_P40551 | *BEX4* | 1.93E-04 | 3.41 | down | 1.34E-03 | 3.45 | down |
| A_24_P409013 | *WDR78* | 1.73E-06 | 3.41 | down | 6.08E-03 | 6.45 | down |
| A_23_P19723 | *BMP5* | 4.41E-06 | 3.40 | down | 1.16E-03 | 8.65 | down |
| A_24_P353638 | *SLAMF7* | 1.37E-04 | 3.38 | down | 8.20E-03 | 4.45 | down |
| A_23_P252541 | *RAB7B* | 4.67E-09 | 3.38 | down | 9.03E-03 | 2.24 | down |
| A_32_P72611 |  | 4.75E-08 | 3.37 | down | 1.92E-03 | 4.03 | down |
| A_23_P386942 | *DIRAS1* | 3.88E-05 | 3.34 | down | 9.58E-04 | 4.19 | down |
| A_24_P352388 | *CDHR5* | 7.37E-06 | 3.34 | down | 2.31E-03 | 7.21 | down |
| A_24_P935103 | *ADCY9* | 1.20E-10 | 3.34 | down | 5.72E-03 | 2.51 | down |
| A_23_P218369 | *CCL14* | 4.81E-08 | 3.33 | down | 3.79E-03 | 4.11 | down |
| A_32_P115749 |  | 8.08E-05 | 3.32 | down | 5.19E-03 | 3.08 | down |
| A_32_P101313 | *PTPLAD2* | 5.08E-08 | 3.32 | down | 1.38E-03 | 2.67 | down |
| A_24_P153568 | *MPEG1* | 5.67E-10 | 3.32 | down | 8.53E-04 | 4.39 | down |
| A_23_P61057 | *IL16* | 2.65E-05 | 3.31 | down | 8.30E-03 | 3.37 | down |
| A_23_P89589 | *PER1* | 1.02E-06 | 3.30 | down | 4.38E-03 | 2.30 | down |
| A_24_P204574 |  | 7.86E-03 | 3.28 | down | 6.09E-03 | 5.76 | down |
| A_23_P105461 | *CMKLR1* | 1.24E-07 | 3.27 | down | 9.41E-04 | 2.29 | down |
| A_23_P110791 | *CSF1R* | 3.24E-09 | 3.27 | down | 5.21E-03 | 2.54 | down |
| A_32_P116206 | *RELL1* | 3.58E-11 | 3.26 | down | 3.86E-03 | 2.42 | down |
| A_23_P96271 | *MYOM1* | 8.52E-06 | 3.26 | down | 3.23E-03 | 4.84 | down |
| A_23_P49816 | *ADAP2* | 6.08E-07 | 3.24 | down | 1.59E-03 | 2.48 | down |
| A_24_P124349 | *PDGFD* | 2.44E-06 | 3.24 | down | 1.20E-03 | 5.15 | down |
| A_23_P203173 | *IL10RA* | 3.39E-08 | 3.22 | down | 8.63E-03 | 2.46 | down |
| A_24_P270424 | *DPF3* | 3.49E-05 | 3.21 | down | 6.52E-04 | 10.91 | down |
| A_32_P50655 |  | 1.45E-05 | 3.21 | down | 1.07E-03 | 3.50 | down |
| A_23_P87853 | *TMCC3* | 1.07E-05 | 3.19 | down | 5.06E-03 | 3.18 | down |
| A_23_P16722 | *DOCK10* | 6.37E-09 | 3.17 | down | 9.86E-03 | 2.69 | down |
| A_23_P414913 | *GLIPR2* | 2.73E-05 | 3.17 | down | 7.16E-03 | 3.20 | down |
| A_23_P217528 | *KLF8* | 6.48E-06 | 3.16 | down | 1.05E-03 | 2.85 | down |
| A_23_P79572 | *MGC16025* | 1.50E-03 | 3.15 | down | 7.63E-03 | 4.21 | down |
| A_32_P132194 |  | 1.90E-03 | 3.15 | down | 7.42E-03 | 3.70 | down |
| A_23_P312920 | *POU2AF1* | 2.75E-03 | 3.14 | down | 6.24E-03 | 8.19 | down |
| A_23_P141367 | *CCR10* | 5.42E-04 | 3.14 | down | 9.95E-04 | 3.46 | down |
| A_23_P153897 | *GNG7* | 2.20E-05 | 3.13 | down | 3.55E-03 | 3.90 | down |
| A_23_P10401 | *PPP2R3A* | 1.06E-08 | 3.13 | down | 6.90E-03 | 3.04 | down |
| A_23_P119196 | *KLF2* | 1.03E-05 | 3.12 | down | 3.11E-03 | 2.09 | down |
| A_23_P83857 | *MAOA* | 5.89E-08 | 3.11 | down | 8.22E-03 | 3.98 | down |
| A_23_P415401 | *KLF9* | 9.61E-06 | 3.11 | down | 4.36E-03 | 3.05 | down |
| A_23_P325924 | *FAM59B* | 4.05E-05 | 3.11 | down | 8.54E-03 | 2.71 | down |
| A_24_P330518 | *CA12* | 1.12E-04 | 3.09 | down | 2.54E-03 | 5.72 | down |
| A_23_P72117 | *SMPDL3A* | 1.20E-06 | 3.08 | down | 1.90E-03 | 7.07 | down |
| A_23_P92903 | *C1QTNF2* | 7.04E-09 | 3.08 | down | 4.72E-03 | 2.66 | down |
| A_32_P80850 | *COL14A1* | 8.80E-09 | 3.08 | down | 3.37E-03 | 2.11 | down |
| A_23_P28906 |  | 5.84E-06 | 3.07 | down | 5.88E-03 | 4.66 | down |
| A_23_P253602 | *BMX* | 1.61E-03 | 3.07 | down | 6.03E-03 | 7.09 | down |
| A_23_P166207 | *ABCC13* | 5.07E-04 | 3.06 | down | 3.28E-03 | 7.61 | down |
| A_23_P324340 | *DISP2* | 2.53E-05 | 3.06 | down | 7.24E-03 | 4.37 | down |
| A_23_P71328 | *MATN2* | 1.91E-03 | 3.05 | down | 7.63E-03 | 4.14 | down |
| A_23_P128084 | *ITGA7* | 1.44E-07 | 3.04 | down | 2.88E-03 | 2.14 | down |
| A_24_P296772 | *PPP1R14A* | 7.76E-07 | 3.02 | down | 6.98E-03 | 3.18 | down |
| A_24_P205604 | *PADI2* | 1.16E-03 | 3.02 | down | 4.28E-03 | 6.78 | down |
| A_32_P116203 | *NCF1* | 1.21E-06 | 3.01 | down | 6.22E-03 | 2.44 | down |
| A_23_P155666 | *NAAA* | 2.14E-04 | 3.00 | down | 7.76E-03 | 2.75 | down |
| A_23_P56868 |  | 6.56E-03 | 3.00 | down | 1.16E-03 | 4.82 | down |
| A_23_P301530 | *ANK3* | 7.32E-09 | 2.99 | down | 6.05E-03 | 2.89 | down |
| A_24_P388433 | *PPP2R3A* | 1.78E-07 | 2.97 | down | 2.25E-03 | 4.38 | down |
| A_23_P10902 | *FRZB* | 2.79E-03 | 2.97 | down | 9.99E-03 | 4.24 | down |
| A_23_P258190 | *AKR1B1* | 4.13E-05 | 2.97 | down | 3.36E-03 | 3.89 | down |
| A_23_P252075 | *AHCYL2* | 6.10E-06 | 2.96 | down | 4.08E-03 | 4.03 | down |
| A_24_P262201 | *SULT1A4* | 1.50E-06 | 2.96 | down | 4.17E-03 | 3.22 | down |
| A_23_P202269 | *ANK3* | 3.16E-04 | 2.95 | down | 6.49E-03 | 3.12 | down |
| A_23_P121926 | *SEPP1* | 3.11E-06 | 2.95 | down | 2.45E-03 | 6.27 | down |
| A_24_P941773 | *METTL7A* | 4.77E-04 | 2.94 | down | 2.66E-03 | 6.14 | down |
| A_23_P342138 | *ADAMTSL1* | 2.90E-04 | 2.94 | down | 6.54E-04 | 5.88 | down |
| A_32_P17484 |  | 1.24E-06 | 2.93 | down | 5.98E-03 | 2.63 | down |
| A_32_P24832 | *OLFML3* | 2.86E-06 | 2.93 | down | 9.84E-03 | 2.23 | down |
| A_23_P67799 | *TMEM37* | 5.73E-06 | 2.93 | down | 5.48E-03 | 5.20 | down |
| A_23_P140190 | *KIAA0125* | 6.61E-03 | 2.92 | down | 6.54E-03 | 5.70 | down |
| A_23_P23279 | *RCSD1* | 3.65E-07 | 2.92 | down | 4.65E-03 | 3.27 | down |
| A_32_P207789 |  | 5.43E-05 | 2.92 | down | 3.65E-03 | 2.93 | down |
| A_23_P21758 | *ADAM28* | 1.40E-06 | 2.92 | down | 7.23E-03 | 4.49 | down |
| A_32_P30898 |  | 1.94E-07 | 2.91 | down | 8.86E-03 | 2.45 | down |
| A_24_P943393 | *AHNAK* | 1.92E-06 | 2.90 | down | 4.41E-03 | 2.79 | down |
| A_32_P218785 |  | 3.51E-03 | 2.90 | down | 8.10E-03 | 2.85 | down |
| A_23_P35617 | *PLCE1* | 1.62E-06 | 2.90 | down | 4.44E-03 | 5.40 | down |
| A_23_P144807 | *39692* | 5.33E-10 | 2.89 | down | 8.51E-04 | 2.73 | down |
| A_24_P913716 | *B3GNT7* | 3.52E-03 | 2.89 | down | 5.58E-03 | 5.89 | down |
| A_24_P319088 | *CCL23* | 2.33E-06 | 2.88 | down | 6.90E-03 | 2.86 | down |
| A_23_P102160 | *FAM82A1* | 4.39E-10 | 2.88 | down | 8.81E-04 | 5.46 | down |
| A_23_P41365 | *SMR3A* | 8.54E-03 | 2.87 | down | 6.52E-03 | 4.23 | down |
| A_24_P927886 | *GNA11* | 3.76E-06 | 2.87 | down | 2.35E-03 | 2.32 | down |
| A_23_P360804 | *CPNE5* | 4.62E-04 | 2.86 | down | 2.52E-03 | 4.64 | down |
| A_24_P75008 | *LOC346329* | 1.67E-06 | 2.86 | down | 2.64E-03 | 2.27 | down |
| A_23_P80739 | *PLCD1* | 4.41E-06 | 2.86 | down | 8.53E-04 | 5.13 | down |
| A_23_P24616 | *SIAE* | 1.09E-03 | 2.85 | down | 5.72E-03 | 3.60 | down |
| A_24_P252739 | *KLF6* | 3.25E-03 | 2.84 | down | 1.92E-03 | 3.65 | down |
| A_32_P91273 |  | 6.88E-08 | 2.82 | down | 2.32E-03 | 3.58 | down |
| A_24_P278603 | *MOGAT2* | 9.14E-05 | 2.82 | down | 1.53E-03 | 7.00 | down |
| A_23_P92899 | *C1QTNF2* | 8.91E-09 | 2.82 | down | 3.01E-03 | 2.54 | down |
| A_32_P193218 | *LPP* | 2.88E-04 | 2.81 | down | 8.88E-03 | 2.22 | down |
| A_23_P213959 | *PPARGC1B* | 4.83E-06 | 2.81 | down | 3.74E-03 | 2.98 | down |
| A_23_P2041 | *MICALCL* | 1.89E-06 | 2.81 | down | 2.87E-03 | 3.22 | down |
| A_32_P227930 | *C5orf52* | 1.26E-05 | 2.81 | down | 2.32E-03 | 5.76 | down |
| A_23_P142289 | *GNA11* | 6.18E-06 | 2.80 | down | 2.45E-03 | 2.35 | down |
| A_24_P363583 | *AGFG2* | 8.66E-07 | 2.80 | down | 4.36E-03 | 3.34 | down |
| A_23_P85015 | *MAOB* | 3.76E-06 | 2.80 | down | 9.07E-03 | 3.39 | down |
| A_23_P120644 |  | 1.36E-03 | 2.80 | down | 8.11E-04 | 2.76 | down |
| A_23_P253345 | *C8orf4* | 1.47E-04 | 2.80 | down | 6.25E-03 | 2.67 | down |
| A_24_P361816 |  | 9.02E-03 | 2.79 | down | 7.47E-03 | 5.22 | down |
| A_32_P38637 | *KRBA1* | 6.65E-05 | 2.79 | down | 6.91E-03 | 2.18 | down |
| A_24_P373152 | *CFL2* | 9.96E-06 | 2.78 | down | 3.18E-03 | 2.37 | down |
| A_24_P766716 | *CMKLR1* | 4.15E-07 | 2.78 | down | 1.38E-03 | 2.92 | down |
| A_23_P156826 | *C6orf105* | 3.72E-03 | 2.78 | down | 1.86E-03 | 18.54 | down |
| A_23_P74575 | *CD1D* | 3.17E-04 | 2.78 | down | 1.63E-03 | 4.03 | down |
| A_32_P92840 |  | 5.49E-08 | 2.78 | down | 7.68E-03 | 2.16 | down |
| A_23_P103601 | *MAN1C1* | 1.20E-06 | 2.77 | down | 8.54E-03 | 2.57 | down |
| A_23_P300033 | *PDGFRA* | 2.26E-06 | 2.76 | down | 6.64E-03 | 2.85 | down |
| A_32_P69956 |  | 7.22E-05 | 2.74 | down | 8.53E-03 | 2.92 | down |
| A_32_P122715 |  | 2.51E-08 | 2.74 | down | 3.95E-03 | 3.00 | down |
| A_23_P65022 | *ACADS* | 5.05E-05 | 2.74 | down | 1.38E-03 | 5.15 | down |
| A_23_P70060 | *PPAP2A* | 7.21E-08 | 2.74 | down | 1.34E-03 | 3.72 | down |
| A_32_P95894 |  | 3.84E-04 | 2.74 | down | 3.18E-03 | 2.42 | down |
| A_23_P37317 |  | 1.60E-07 | 2.73 | down | 2.74E-03 | 2.10 | down |
| A_23_P106675 | *PLCG2* | 5.14E-05 | 2.73 | down | 2.34E-03 | 3.83 | down |
| A_24_P305933 | *TMCC3* | 8.32E-07 | 2.72 | down | 2.54E-03 | 2.81 | down |
| A_32_P109495 |  | 1.43E-06 | 2.72 | down | 3.89E-03 | 2.51 | down |
| A_32_P216004 |  | 1.21E-04 | 2.71 | down | 4.48E-03 | 2.56 | down |
| A_23_P352266 | *BCL2* | 4.91E-05 | 2.71 | down | 9.56E-04 | 5.62 | down |
| A_24_P928522 | *DST* | 1.36E-03 | 2.70 | down | 1.54E-03 | 3.25 | down |
| A_24_P937240 |  | 3.49E-06 | 2.70 | down | 2.69E-03 | 4.35 | down |
| A_23_P110253 | *KIT* | 7.41E-04 | 2.70 | down | 4.98E-03 | 4.57 | down |
| A_23_P145024 | *ADRB2* | 9.09E-05 | 2.69 | down | 5.11E-03 | 3.93 | down |
| A_23_P123086 | *KIAA1908* | 5.10E-05 | 2.67 | down | 2.31E-03 | 3.03 | down |
| A_23_P203488 | *SMPD1* | 6.05E-05 | 2.66 | down | 1.18E-03 | 3.24 | down |
| A_24_P397294 | *LTC4S* | 5.46E-08 | 2.66 | down | 6.24E-03 | 2.41 | down |
| A_23_P203376 | *MS4A6A* | 2.31E-06 | 2.66 | down | 1.68E-03 | 2.61 | down |
| A_24_P305345 | *CD209* | 1.09E-06 | 2.66 | down | 1.05E-03 | 4.01 | down |
| A_23_P88767 | *PLA2G10* | 4.00E-04 | 2.65 | down | 6.28E-03 | 5.70 | down |
| A_32_P217140 | *ISX* | 3.53E-04 | 2.65 | down | 4.80E-03 | 8.32 | down |
| A_23_P33791 | *SSBP2* | 9.89E-06 | 2.65 | down | 1.78E-03 | 2.08 | down |
| A_23_P135990 | *SLCO2A1* | 6.15E-05 | 2.65 | down | 1.05E-03 | 4.21 | down |
| A_23_P65629 | *KCNK10* | 5.70E-03 | 2.64 | down | 3.79E-03 | 5.42 | down |
| A_24_P944154 | *MCTP2* | 2.04E-03 | 2.64 | down | 8.46E-03 | 3.21 | down |
| A_23_P414654 | *RAB37* | 1.86E-04 | 2.64 | down | 3.36E-03 | 3.91 | down |
| A_23_P14986 | *HSD11B2* | 2.52E-06 | 2.64 | down | 1.16E-03 | 7.07 | down |
| A_23_P302914 | *ZFYVE28* | 3.17E-06 | 2.63 | down | 2.16E-03 | 2.87 | down |
| A_23_P209944 | *RETSAT* | 2.57E-06 | 2.63 | down | 4.80E-03 | 2.43 | down |
| A_23_P127789 | *AHNAK* | 4.32E-04 | 2.63 | down | 9.21E-03 | 2.47 | down |
| A_23_P95619 | *GLOD5* | 3.29E-07 | 2.63 | down | 2.88E-03 | 3.78 | down |
| A_32_P120484 |  | 1.62E-05 | 2.62 | down | 1.16E-03 | 6.28 | down |
| A_24_P192914 | *AMICA1* | 2.63E-06 | 2.62 | down | 8.11E-04 | 3.05 | down |
| A_23_P99141 | *GPR162* | 8.68E-08 | 2.62 | down | 2.54E-03 | 2.46 | down |
| A_23_P26865 | *MYH3* | 4.32E-04 | 2.61 | down | 7.69E-03 | 2.98 | down |
| A_24_P497244 | *MALAT1* | 8.79E-03 | 2.61 | down | 6.40E-03 | 3.05 | down |
| A_23_P130974 | *KIAA1683* | 1.98E-04 | 2.60 | down | 4.29E-03 | 3.50 | down |
| A_23_P10442 | *OSBPL1A* | 3.26E-05 | 2.60 | down | 5.20E-03 | 3.44 | down |
| A_23_P143331 | *BMP2* | 1.52E-05 | 2.59 | down | 4.30E-03 | 3.65 | down |
| A_23_P99747 | *CDKL1* | 1.13E-07 | 2.59 | down | 5.51E-04 | 2.64 | down |
| A_23_P24774 | *ABCC8* | 5.93E-05 | 2.59 | down | 2.38E-03 | 2.09 | down |
| A_23_P392470 | *NR3C2* | 3.98E-06 | 2.58 | down | 6.39E-03 | 7.53 | down |
| A_24_P227971 | *TP53TG3* | 5.16E-03 | 2.58 | down | 1.27E-03 | 3.34 | down |
| A_24_P136484 |  | 5.87E-04 | 2.58 | down | 2.41E-03 | 3.12 | down |
| A_23_P127727 | *MPEG1* | 2.97E-08 | 2.58 | down | 6.52E-04 | 2.89 | down |
| A_23_P416711 | *ST6GALNAC3* | 7.77E-05 | 2.58 | down | 8.79E-03 | 2.09 | down |
| A_24_P131392 | *FAM82A1* | 1.90E-07 | 2.57 | down | 1.00E-03 | 4.92 | down |
| A_23_P331748 | *CD33* | 1.47E-07 | 2.56 | down | 3.46E-03 | 2.41 | down |
| A_23_P32233 | *KLF4* | 3.41E-04 | 2.55 | down | 1.48E-03 | 4.65 | down |
| A_24_P282108 | *ZZEF1* | 5.40E-10 | 2.55 | down | 1.34E-03 | 3.24 | down |
| A_23_P312840 | *SEMA6A* | 1.35E-04 | 2.55 | down | 9.02E-03 | 3.34 | down |
| A_24_P142973 | *PEX26* | 1.14E-10 | 2.55 | down | 9.23E-03 | 2.41 | down |
| A_24_P246891 | *NEU4* | 6.98E-03 | 2.54 | down | 6.01E-03 | 4.09 | down |
| A_23_P1759 | *AMICA1* | 1.15E-05 | 2.54 | down | 1.25E-03 | 2.89 | down |
| A_23_P67198 | *CPAMD8* | 2.86E-04 | 2.54 | down | 7.04E-03 | 4.04 | down |
| A_23_P49610 | *C17orf91* | 1.98E-03 | 2.54 | down | 2.13E-03 | 2.76 | down |
| A_23_P407565 | *CX3CR1* | 2.17E-04 | 2.53 | down | 6.64E-03 | 2.14 | down |
| A_23_P78018 | *ABCA5* | 2.33E-06 | 2.53 | down | 4.84E-03 | 3.63 | down |
| A_24_P127159 |  | 1.19E-05 | 2.53 | down | 3.67E-03 | 2.22 | down |
| A_24_P354488 | *NAAA* | 8.18E-06 | 2.52 | down | 3.61E-03 | 3.38 | down |
| A_23_P44335 | *ENTPD8* | 4.21E-03 | 2.52 | down | 7.14E-03 | 5.30 | down |
| A_23_P48088 | *CD27* | 8.90E-03 | 2.52 | down | 3.83E-03 | 3.91 | down |
| A_23_P149975 | *FAM107B* | 2.80E-05 | 2.51 | down | 5.00E-03 | 2.25 | down |
| A_23_P71867 | *IL11RA* | 5.02E-07 | 2.51 | down | 1.16E-03 | 2.86 | down |
| A_23_P50217 | *ZNF671* | 1.10E-03 | 2.50 | down | 7.63E-03 | 2.97 | down |
| A_32_P515920 | *LOC400573* | 7.71E-06 | 2.50 | down | 1.24E-03 | 5.17 | down |
| A_23_P363647 | *DDX26B* | 5.29E-05 | 2.50 | down | 4.12E-03 | 3.32 | down |
| A_23_P167168 | *IGJ* | 5.24E-03 | 2.50 | down | 3.42E-03 | 8.87 | down |
| A_23_P101642 | *PTPRH* | 4.67E-06 | 2.49 | down | 6.03E-03 | 3.44 | down |
| A_23_P408996 | *MBOAT1* | 1.15E-05 | 2.49 | down | 4.81E-03 | 3.25 | down |
| A_24_P109432 | *NBEAL1* | 4.49E-04 | 2.49 | down | 6.26E-03 | 3.21 | down |
| A_23_P99275 | *KLRB1* | 3.66E-04 | 2.48 | down | 4.09E-03 | 3.27 | down |
| A_24_P673968 | *TTC22* | 2.66E-07 | 2.47 | down | 3.22E-03 | 2.55 | down |
| A_23_P110569 | *TRIM36* | 5.51E-06 | 2.47 | down | 5.45E-03 | 4.72 | down |
| A_32_P104746 | *ZFYVE28* | 1.18E-05 | 2.47 | down | 3.14E-03 | 2.80 | down |
| A_32_P130641 | *STARD9* | 1.61E-05 | 2.47 | down | 3.46E-03 | 2.65 | down |
| A_32_P46495 |  | 4.43E-03 | 2.46 | down | 8.25E-03 | 3.04 | down |
| A_24_P372643 | *SLC22A18AS* | 1.01E-04 | 2.46 | down | 9.56E-04 | 3.29 | down |
| A_24_P173823 | *PBX1* | 7.98E-06 | 2.46 | down | 8.79E-03 | 2.96 | down |
| A_24_P106166 |  | 4.96E-04 | 2.46 | down | 5.20E-03 | 2.77 | down |
| A_24_P254949 | *PGM5* | 9.88E-04 | 2.45 | down | 2.25E-03 | 2.06 | down |
| A_23_P143526 | *S100B* | 3.11E-03 | 2.45 | down | 9.48E-03 | 3.06 | down |
| A_23_P150325 | *TMEM133* | 5.71E-09 | 2.45 | down | 5.21E-03 | 2.20 | down |
| A_23_P142796 | *LIMS2* | 1.71E-03 | 2.44 | down | 9.23E-03 | 2.22 | down |
| A_32_P226786 | *FAM126B* | 1.15E-08 | 2.44 | down | 8.34E-03 | 2.22 | down |
| A_23_P500861 | *SYNE1* | 3.00E-06 | 2.44 | down | 4.87E-03 | 2.78 | down |
| A_23_P122007 | *C5orf30* | 3.46E-10 | 2.44 | down | 3.65E-03 | 2.76 | down |
| A_23_P202334 | *FGFR2* | 2.44E-04 | 2.43 | down | 6.12E-03 | 4.10 | down |
| A_23_P99741 | *CDKL1* | 2.83E-06 | 2.43 | down | 2.14E-03 | 2.31 | down |
| A_32_P194563 |  | 1.69E-03 | 2.42 | down | 2.79E-03 | 2.65 | down |
| A_23_P158330 | *UGT1A8* | 2.59E-03 | 2.42 | down | 1.12E-03 | 6.73 | down |
| A_24_P301655 | *CD33* | 1.31E-06 | 2.41 | down | 3.07E-03 | 2.28 | down |
| A_23_P13382 | *LSP1* | 4.61E-04 | 2.41 | down | 8.50E-03 | 2.49 | down |
| A_32_P226907 | *LOC284112* | 1.77E-08 | 2.41 | down | 6.52E-04 | 2.78 | down |
| A_24_P188975 | *FLJ11235* | 5.78E-03 | 2.40 | down | 8.58E-03 | 2.36 | down |
| A_32_P72758 | *MCTP2* | 5.24E-03 | 2.40 | down | 5.58E-03 | 2.66 | down |
| A_23_P254079 | *STBD1* | 5.44E-05 | 2.39 | down | 2.43E-03 | 2.92 | down |
| A_23_P257993 | *DNASE1L3* | 2.08E-04 | 2.39 | down | 1.94E-03 | 12.77 | down |
| A_23_P339818 | *ARRDC4* | 7.18E-05 | 2.39 | down | 3.50E-03 | 2.81 | down |
| A_24_P68079 | *TRANK1* | 1.12E-04 | 2.39 | down | 2.19E-03 | 4.19 | down |
| A_23_P159237 | *GPR20* | 1.47E-03 | 2.39 | down | 3.13E-03 | 2.04 | down |
| A_24_P922261 | *SRGAP1* | 9.08E-03 | 2.38 | down | 5.04E-03 | 2.83 | down |
| A_23_P25336 | *GLTP* | 9.37E-06 | 2.38 | down | 1.18E-03 | 2.26 | down |
| A_23_P92161 | *ARL14* | 4.11E-05 | 2.38 | down | 6.74E-03 | 5.02 | down |
| A_23_P83277 | *IL11RA* | 6.89E-06 | 2.37 | down | 1.16E-03 | 3.00 | down |
| A_24_P205130 | *FNBP1* | 2.57E-06 | 2.37 | down | 7.23E-03 | 2.62 | down |
| A_23_P114689 | *ASAP3* | 5.04E-05 | 2.37 | down | 7.64E-04 | 5.03 | down |
| A_23_P113161 | *C1orf21* | 1.05E-05 | 2.37 | down | 4.69E-03 | 4.43 | down |
| A_23_P77073 | *SPPL2A* | 1.54E-06 | 2.36 | down | 2.31E-03 | 2.93 | down |
| A_23_P110961 | *BRP44L* | 1.60E-06 | 2.36 | down | 4.67E-03 | 2.70 | down |
| A_23_P61447 | *ETFDH* | 3.69E-07 | 2.35 | down | 5.86E-03 | 2.81 | down |
| A_23_P118254 | *FOXF1* | 3.09E-06 | 2.35 | down | 9.14E-03 | 2.18 | down |
| A_23_P149998 | *PBLD* | 3.95E-05 | 2.35 | down | 9.07E-03 | 5.58 | down |
| A_23_P131990 | *VSX1* | 2.15E-04 | 2.34 | down | 4.44E-03 | 2.22 | down |
| A_24_P650482 | *LOC400960* | 5.53E-06 | 2.34 | down | 1.77E-03 | 3.83 | down |
| A_23_P91910 | *PLSCR4* | 4.51E-06 | 2.33 | down | 6.85E-03 | 3.52 | down |
| A_23_P24077 | *C10orf54* | 6.40E-12 | 2.33 | down | 1.36E-03 | 2.45 | down |
| A_23_P60599 | *UGT1A6* | 2.65E-03 | 2.33 | down | 3.46E-03 | 10.89 | down |
| A_23_P80594 | *PLCL2* | 2.61E-06 | 2.33 | down | 7.02E-03 | 4.60 | down |
| A_23_P502343 | *ADAM33* | 7.69E-07 | 2.33 | down | 1.27E-03 | 2.23 | down |
| A_23_P110957 | *FOXF2* | 1.24E-07 | 2.33 | down | 5.46E-03 | 3.53 | down |
| A_23_P23457 | *FBLIM1* | 4.52E-07 | 2.32 | down | 1.54E-03 | 2.17 | down |
| A_23_P334864 | *FAM126B* | 6.74E-09 | 2.32 | down | 2.35E-03 | 2.14 | down |
| A_23_P131060 | *CYP4F8* | 2.57E-04 | 2.32 | down | 8.48E-03 | 3.24 | down |
| A_23_P119923 | *CNNM4* | 6.15E-08 | 2.31 | down | 4.57E-03 | 2.58 | down |
| A_23_P24044 | *CNNM2* | 1.72E-06 | 2.29 | down | 2.82E-03 | 2.18 | down |
| A_24_P373562 | *ADAP2* | 1.61E-06 | 2.29 | down | 1.48E-03 | 2.09 | down |
| A_23_P11201 | *GPR34* | 7.14E-06 | 2.28 | down | 5.08E-03 | 2.49 | down |
| A_24_P294832 | *PTP4A1* | 1.58E-05 | 2.28 | down | 1.54E-03 | 2.48 | down |
| A_23_P29096 | *PDE9A* | 2.32E-03 | 2.27 | down | 4.83E-03 | 4.01 | down |
| A_24_P71373 | *SLC9A1* | 4.93E-07 | 2.26 | down | 6.54E-04 | 2.85 | down |
| A_23_P320216 | *FAM55D* | 1.00E-02 | 2.25 | down | 3.65E-03 | 17.45 | down |
| A_23_P253046 | *UGP2* | 1.28E-09 | 2.24 | down | 2.51E-03 | 2.42 | down |
| A_23_P160433 | *C1orf115* | 1.89E-04 | 2.23 | down | 4.35E-03 | 4.83 | down |
| A_23_P72025 | *SLC25A20* | 8.46E-05 | 2.23 | down | 6.35E-03 | 2.85 | down |
| A_24_P189533 | *ENDOD1* | 4.25E-05 | 2.23 | down | 1.53E-03 | 2.71 | down |
| A_24_P218814 | *RDH5* | 2.54E-03 | 2.22 | down | 8.51E-04 | 6.34 | down |
| A_23_P162288 | *MYO1A* | 6.68E-04 | 2.22 | down | 6.76E-03 | 4.27 | down |
| A_23_P104624 | *ENDOD1* | 5.63E-06 | 2.21 | down | 3.13E-03 | 2.76 | down |
| A_23_P208450 | *SLC25A23* | 1.13E-07 | 2.21 | down | 3.91E-03 | 2.49 | down |
| A_23_P5051 | *GNA11* | 1.37E-09 | 2.21 | down | 2.32E-03 | 2.27 | down |
| A_24_P112395 | *PBLD* | 1.11E-04 | 2.21 | down | 7.65E-03 | 5.31 | down |
| A_24_P200162 | *HIGD1A* | 5.07E-06 | 2.21 | down | 9.05E-04 | 2.65 | down |
| A_23_P152428 | *MARVELD3* | 1.41E-04 | 2.19 | down | 2.17E-03 | 2.01 | down |
| A_23_P81441 | *C5orf20* | 1.82E-03 | 2.19 | down | 2.14E-03 | 2.92 | down |
| A_23_P252201 | *EAF2* | 4.53E-04 | 2.19 | down | 3.65E-03 | 2.38 | down |
| A_24_P32085 | *MOBKL2B* | 1.87E-05 | 2.19 | down | 6.77E-03 | 3.44 | down |
| A_23_P343104 | *FLJ30901* | 1.30E-03 | 2.18 | down | 8.72E-04 | 6.03 | down |
| A_23_P19095 | *SNX24* | 1.24E-04 | 2.18 | down | 3.74E-03 | 3.00 | down |
| A_23_P164528 | *WDR7* | 2.28E-09 | 2.18 | down | 5.07E-03 | 2.09 | down |
| A_23_P213745 | *CXCL14* | 1.06E-04 | 2.17 | down | 9.25E-03 | 3.64 | down |
| A_23_P160167 | *TSPAN1* | 1.19E-04 | 2.16 | down | 5.24E-03 | 4.19 | down |
| A_32_P31785 |  | 8.49E-05 | 2.16 | down | 6.13E-03 | 2.03 | down |
| A_23_P167129 | *HHIP* | 1.80E-04 | 2.16 | down | 4.33E-03 | 3.63 | down |
| A_32_P224522 | *SLC25A23* | 2.66E-07 | 2.14 | down | 2.87E-03 | 2.59 | down |
| A_23_P163567 | *SMPD3* | 2.86E-06 | 2.14 | down | 7.89E-03 | 4.08 | down |
| A_32_P140656 | *IL6ST* | 2.39E-07 | 2.14 | down | 2.49E-03 | 2.49 | down |
| A_32_P475513 | *MYO15B* | 3.45E-04 | 2.13 | down | 9.07E-03 | 2.87 | down |
| A_23_P41992 | *GFRA3* | 6.36E-06 | 2.13 | down | 2.33E-03 | 2.11 | down |
| A_23_P117580 | *ENTPD5* | 2.27E-03 | 2.13 | down | 5.08E-03 | 3.78 | down |
| A_24_P659415 |  | 1.99E-04 | 2.12 | down | 8.64E-03 | 2.17 | down |
| A_32_P47543 |  | 8.75E-05 | 2.11 | down | 8.63E-03 | 2.04 | down |
| A_32_P184888 |  | 9.47E-05 | 2.11 | down | 1.86E-03 | 3.41 | down |
| A_32_P174083 | *CYCS* | 1.53E-04 | 2.09 | down | 4.30E-03 | 2.82 | down |
| A_23_P309261 | *AKAP9* | 8.50E-06 | 2.09 | down | 1.16E-03 | 2.80 | down |
| A_24_P396702 | *CD302* | 2.54E-05 | 2.08 | down | 1.53E-03 | 3.22 | down |
| A_24_P402690 | *ITM2C* | 5.68E-04 | 2.08 | down | 5.51E-03 | 5.62 | down |
| A_23_P152970 | *RAPGEFL1* | 3.79E-04 | 2.08 | down | 6.16E-03 | 2.61 | down |
| A_24_P131173 | *C1orf115* | 8.42E-04 | 2.08 | down | 6.01E-03 | 4.46 | down |
| A_24_P373174 | *RAB27A* | 4.66E-04 | 2.07 | down | 4.18E-03 | 3.78 | down |
| A_24_P174755 | *SLC22A5* | 2.79E-06 | 2.07 | down | 1.00E-03 | 3.93 | down |
| A_24_P12521 | *C7orf31* | 6.37E-04 | 2.06 | down | 3.38E-03 | 4.76 | down |
| A_23_P116512 | *PRR5L* | 2.28E-07 | 2.05 | down | 1.79E-03 | 2.66 | down |
| A_24_P64167 | *PTGS1* | 3.88E-03 | 2.04 | down | 3.56E-03 | 3.35 | down |
| A_24_P459621 | *FLJ34515* | 8.67E-04 | 2.04 | down | 2.50E-03 | 2.99 | down |
| A_23_P104493 | *PAPSS2* | 9.54E-04 | 2.04 | down | 7.64E-04 | 2.87 | down |
| A_23_P69531 | *KLB* | 2.44E-04 | 2.04 | down | 1.72E-03 | 5.60 | down |
| A_24_P379820 | *ITM2C* | 3.47E-03 | 2.04 | down | 2.84E-03 | 5.85 | down |
| A_23_P305723 | *MIER1* | 1.56E-10 | 2.03 | down | 1.16E-03 | 2.14 | down |
| A_24_P67096 | *ABCA5* | 2.18E-03 | 2.03 | down | 8.36E-03 | 3.87 | down |
| A_24_P798431 |  | 1.83E-04 | 2.03 | down | 7.59E-03 | 2.72 | down |
| A_24_P930927 |  | 4.33E-04 | 2.02 | down | 2.36E-03 | 3.23 | down |
| A_23_P257155 | *ATXN7* | 7.23E-04 | 2.02 | down | 6.19E-03 | 2.12 | down |
| A_23_P433760 | *SPN* | 4.46E-03 | 2.02 | down | 7.77E-03 | 2.68 | down |
| A_24_P153713 | *MARVELD3* | 7.69E-03 | 2.02 | down | 3.43E-03 | 3.13 | down |
| A_24_P403959 | *RNASE1* | 9.92E-03 | 2.02 | down | 8.89E-03 | 2.17 | down |
| A_23_P22350 | *GRAMD3* | 9.15E-06 | 2.02 | down | 6.85E-03 | 2.40 | down |
| A_32_P147651 |  | 7.54E-04 | 2.02 | down | 8.27E-03 | 2.89 | down |
| A_23_P253012 | *GRAMD1C* | 6.51E-05 | 2.02 | down | 6.09E-03 | 3.49 | down |
| A_23_P48596 | *RNASE1* | 8.01E-03 | 2.01 | down | 9.50E-03 | 2.22 | down |
| A_24_P239183 | *MUC4* | 8.00E-03 | 2.00 | down | 9.67E-03 | 2.97 | down |
| A_23_P48455 | *AMN* | 2.57E-03 | 2.00 | down | 4.89E-03 | 2.57 | down |
| A_23_P324754 | *KIAA1199* | 4.96E-17 | 45.89 | up | 2.70E-03 | 29.17 | up |
| A_32_P161855 | *KIAA1199* | 8.20E-17 | 42.64 | up | 6.52E-04 | 30.53 | up |
| A_23_P161698 | *MMP3* | 2.34E-08 | 42.07 | up | 2.93E-03 | 72.31 | up |
| A_23_P49155 | *CDH3* | 6.25E-18 | 39.62 | up | 6.65E-04 | 52.90 | up |
| A_32_P164246 | *FOXQ1* | 1.14E-13 | 23.58 | up | 2.25E-03 | 39.58 | up |
| A_23_P135381 | *SP5* | 5.21E-14 | 13.26 | up | 2.68E-03 | 11.13 | up |
| A_32_P183718 | *SP5* | 2.64E-14 | 12.59 | up | 3.22E-03 | 11.00 | up |
| A_23_P57784 | *CLDN1* | 3.34E-12 | 10.19 | up | 1.53E-03 | 21.64 | up |
| A_23_P201706 | *S100A2* | 3.46E-10 | 9.92 | up | 1.65E-03 | 11.16 | up |
| A_23_P1691 | *MMP1* | 6.33E-06 | 9.31 | up | 2.07E-03 | 26.30 | up |
| A_23_P207850 | *TNS4* | 9.76E-09 | 7.07 | up | 2.66E-03 | 5.98 | up |
| A_23_P5903 | *SLCO4A1* | 6.19E-13 | 6.89 | up | 8.53E-04 | 9.79 | up |
| A_23_P76538 | *TESC* | 1.03E-06 | 6.82 | up | 5.04E-03 | 9.76 | up |
| A_24_P398147 | *NEBL* | 1.57E-18 | 6.72 | up | 6.65E-04 | 4.71 | up |
| A_23_P90032 | *LRRC8E* | 2.80E-11 | 6.07 | up | 8.68E-04 | 6.01 | up |
| A_23_P122924 | *INHBA* | 1.66E-06 | 5.72 | up | 6.54E-04 | 41.06 | up |
| A_32_P50603 | *C2orf70* | 4.50E-08 | 5.51 | up | 1.42E-03 | 5.73 | up |
| A_24_P348203 | *LRRC8E* | 1.50E-10 | 5.34 | up | 6.65E-04 | 5.57 | up |
| A_24_P703830 | *NANOS3* | 1.47E-05 | 5.28 | up | 7.51E-03 | 3.71 | up |
| A_23_P121716 | *ANXA3* | 1.77E-15 | 4.70 | up | 3.91E-03 | 3.24 | up |
| A_23_P102731 | *SMOX* | 7.66E-09 | 4.66 | up | 1.72E-03 | 4.53 | up |
| A_23_P53198 | *DGAT2* | 4.99E-10 | 4.62 | up | 3.43E-03 | 4.74 | up |
| A_24_P295791 | *DGAT2* | 3.43E-10 | 4.61 | up | 1.16E-03 | 6.56 | up |
| A_32_P158181 |  | 3.96E-10 | 4.54 | up | 9.56E-04 | 3.95 | up |
| A_23_P56213 | *GRAMD1A* | 1.56E-09 | 4.53 | up | 4.95E-03 | 2.84 | up |
| A_24_P46093 | *SLC6A6* | 1.17E-08 | 4.48 | up | 1.32E-03 | 5.29 | up |
| A_23_P16523 | *GDF15* | 1.21E-07 | 4.38 | up | 7.13E-03 | 4.85 | up |
| A_23_P356684 | *ANLN* | 1.53E-08 | 4.31 | up | 1.66E-03 | 5.00 | up |
| A_23_P49338 | *TNFRSF12A* | 4.62E-08 | 4.31 | up | 2.07E-03 | 8.44 | up |
| A_23_P403445 | *CGREF1* | 1.56E-03 | 4.29 | up | 5.01E-03 | 9.72 | up |
| A_23_P54055 | *JUB* | 2.03E-08 | 4.24 | up | 1.11E-03 | 8.77 | up |
| A_32_P127153 | *SORD* | 3.82E-12 | 4.23 | up | 7.64E-04 | 2.85 | up |
| A_23_P77493 | *TUBB3* | 2.12E-03 | 4.16 | up | 8.95E-04 | 5.41 | up |
| A_24_P234196 | *RRM2* | 1.17E-08 | 4.13 | up | 6.88E-03 | 4.01 | up |
| A_23_P380298 | *ProSAPiP1* | 1.79E-09 | 4.10 | up | 3.02E-03 | 3.18 | up |
| A_23_P145863 | *S100A11* | 6.43E-11 | 4.08 | up | 1.45E-03 | 3.38 | up |
| A_23_P70827 | *KIAA1549* | 2.61E-12 | 4.02 | up | 4.66E-04 | 4.94 | up |
| A_23_P76450 | *PHLDA1* | 1.32E-06 | 4.02 | up | 9.85E-04 | 5.56 | up |
| A_24_P69095 | *ENC1* | 4.46E-10 | 3.98 | up | 8.68E-04 | 3.08 | up |
| A_23_P77103 | *SORD* | 1.01E-09 | 3.97 | up | 1.89E-03 | 2.68 | up |
| A_23_P42718 | *NFE2L3* | 4.22E-10 | 3.97 | up | 3.36E-03 | 6.07 | up |
| A_23_P92349 | *FGFRL1* | 2.60E-07 | 3.94 | up | 1.12E-03 | 3.47 | up |
| A_23_P156327 | *TGFBI* | 6.22E-09 | 3.94 | up | 9.56E-04 | 4.90 | up |
| A_23_P257694 | *GTF2IRD1* | 1.81E-09 | 3.84 | up | 4.82E-03 | 4.25 | up |
| A_24_P20806 | *PRR7* | 3.64E-08 | 3.82 | up | 9.62E-04 | 5.68 | up |
| A_23_P170667 | *ASPHD1* | 6.02E-06 | 3.79 | up | 7.56E-03 | 3.28 | up |
| A_23_P133694 | *SLC29A1* | 5.17E-11 | 3.78 | up | 4.17E-03 | 3.05 | up |
| A_23_P166508 |  | 1.04E-09 | 3.70 | up | 1.48E-03 | 4.91 | up |
| A_23_P63402 | *GPSM2* | 1.30E-10 | 3.67 | up | 3.39E-03 | 4.19 | up |
| A_32_P135243 | *MTHFD1L* | 2.91E-09 | 3.67 | up | 1.27E-03 | 4.77 | up |
| A_23_P126593 | *S100A11* | 3.59E-09 | 3.63 | up | 1.55E-03 | 3.45 | up |
| A_32_P89691 | *SORD* | 9.03E-09 | 3.61 | up | 2.11E-03 | 2.59 | up |
| A_23_P24716 | *TMEM132A* | 5.50E-06 | 3.60 | up | 7.96E-04 | 5.47 | up |
| A_32_P399546 | *ARNTL2* | 2.91E-08 | 3.58 | up | 5.70E-03 | 4.28 | up |
| A_23_P160537 | *C1orf135* | 2.07E-09 | 3.57 | up | 1.27E-03 | 5.26 | up |
| A_32_P142459 |  | 2.04E-08 | 3.51 | up | 6.19E-03 | 3.03 | up |
| A_24_P335620 | *SLC7A5* | 5.49E-04 | 3.50 | up | 2.25E-03 | 8.43 | up |
| A_23_P168443 | *EPHB4* | 6.58E-07 | 3.50 | up | 6.52E-04 | 2.63 | up |
| A_23_P331895 | *TTYH3* | 8.02E-08 | 3.49 | up | 4.83E-03 | 2.81 | up |
| A_23_P112774 | *PTP4A3* | 4.43E-04 | 3.46 | up | 6.61E-03 | 3.00 | up |
| A_23_P315386 | *RHPN1* | 2.85E-07 | 3.44 | up | 1.81E-03 | 3.57 | up |
| A_32_P152696 | *LOC729983* | 3.78E-09 | 3.43 | up | 1.58E-03 | 5.20 | up |
| A_23_P111621 | *GTF2IRD1* | 6.40E-12 | 3.40 | up | 5.88E-03 | 3.59 | up |
| A_24_P8371 | *SPNS2* | 5.24E-07 | 3.33 | up | 7.33E-04 | 4.50 | up |
| A_23_P150316 | *MMP12* | 2.98E-03 | 3.30 | up | 3.22E-03 | 5.52 | up |
| A_23_P206077 | *AEN* | 2.57E-08 | 3.30 | up | 6.05E-03 | 2.84 | up |
| A_24_P235049 | *MTHFD1L* | 5.06E-07 | 3.29 | up | 1.63E-03 | 5.04 | up |
| A_24_P100517 | *C9orf140* | 1.09E-07 | 3.25 | up | 2.57E-03 | 4.91 | up |
| A_23_P30464 | *PRR7* | 3.85E-08 | 3.25 | up | 8.11E-04 | 5.07 | up |
| A_24_P392496 | *LOC100133920* | 1.55E-09 | 3.19 | up | 1.48E-03 | 4.45 | up |
| A_23_P210690 | *TRIB3* | 1.73E-04 | 3.17 | up | 1.45E-03 | 9.88 | up |
| A_23_P35230 | *CD46* | 2.29E-05 | 3.13 | up | 5.73E-03 | 2.28 | up |
| A_23_P115261 | *AGT* | 3.76E-05 | 3.13 | up | 4.20E-03 | 3.47 | up |
| A_23_P259586 | *TTK* | 3.73E-07 | 3.13 | up | 1.86E-03 | 3.13 | up |
| A_23_P256956 | *KIF20A* | 2.69E-08 | 3.12 | up | 2.43E-03 | 3.85 | up |
| A_23_P340698 | *MMP12* | 3.13E-03 | 3.11 | up | 4.63E-03 | 5.02 | up |
| A_23_P214908 | *MTHFD1L* | 4.07E-07 | 3.06 | up | 7.64E-04 | 3.89 | up |
| A_23_P428326 | *TBC1D16* | 2.32E-10 | 3.05 | up | 3.46E-03 | 2.84 | up |
| A_23_P41804 | *NKD2* | 4.38E-03 | 3.03 | up | 2.02E-03 | 9.45 | up |
| A_23_P422115 | *C9orf116* | 5.86E-08 | 3.01 | up | 1.86E-03 | 3.63 | up |
| A_23_P160968 | *LAMC2* | 1.06E-06 | 3.00 | up | 3.57E-03 | 2.27 | up |
| A_23_P208880 | *UHRF1* | 1.68E-05 | 2.98 | up | 6.16E-03 | 3.29 | up |
| A_23_P139820 | *SLC11A2* | 5.70E-08 | 2.92 | up | 6.28E-03 | 2.96 | up |
| A_23_P94795 | *TEAD4* | 2.42E-07 | 2.87 | up | 3.24E-03 | 4.24 | up |
| A_23_P54517 | *TYRO3* | 9.83E-07 | 2.86 | up | 5.00E-03 | 2.84 | up |
| A_24_P67494 |  | 1.35E-07 | 2.85 | up | 1.80E-03 | 3.97 | up |
| A_23_P145786 | *MLXIPL* | 5.92E-04 | 2.83 | up | 2.84E-03 | 5.25 | up |
| A_23_P98282 | *SPTBN2* | 6.67E-07 | 2.81 | up | 2.70E-03 | 2.92 | up |
| A_23_P95029 | *SNTB1* | 2.59E-06 | 2.81 | up | 3.28E-03 | 4.05 | up |
| A_23_P369328 | *C10orf35* | 2.74E-11 | 2.80 | up | 2.91E-03 | 2.84 | up |
| A_24_P377499 | *OSBPL3* | 8.29E-11 | 2.79 | up | 2.94E-03 | 2.74 | up |
| A_24_P122137 | *LIF* | 4.09E-06 | 2.78 | up | 6.54E-03 | 3.00 | up |
| A_23_P366253 | *PATZ1* | 6.08E-10 | 2.77 | up | 6.63E-03 | 2.02 | up |
| A_24_P257099 | *HJURP* | 7.39E-07 | 2.75 | up | 3.06E-03 | 3.70 | up |
| A_23_P256890 | *PTRH1* | 9.55E-08 | 2.75 | up | 2.05E-03 | 2.78 | up |
| A_23_P369899 | *TMEM158* | 3.77E-03 | 2.74 | up | 9.34E-03 | 4.13 | up |
| A_23_P99292 | *RAD51AP1* | 1.49E-06 | 2.73 | up | 1.86E-03 | 2.70 | up |
| A_23_P429491 | *C11orf82* | 5.87E-07 | 2.73 | up | 2.22E-03 | 3.86 | up |
| A_23_P111297 | *RPP40* | 1.06E-09 | 2.72 | up | 4.97E-03 | 2.47 | up |
| A_24_P194081 | *FXYD5* | 4.99E-05 | 2.72 | up | 5.45E-03 | 3.21 | up |
| A_24_P181672 | *B3GNTL1* | 8.54E-09 | 2.72 | up | 1.13E-03 | 2.18 | up |
| A_23_P130995 | *FXYD5* | 1.85E-04 | 2.71 | up | 8.08E-03 | 2.70 | up |
| A_24_P413884 | *CENPA* | 4.70E-06 | 2.71 | up | 3.31E-03 | 3.27 | up |
| A_23_P213424 | *ENC1* | 2.90E-04 | 2.71 | up | 1.47E-03 | 3.03 | up |
| A_32_P209230 | *CITED4* | 7.59E-04 | 2.70 | up | 4.97E-03 | 3.88 | up |
| A_24_P873688 | *CENPN* | 1.17E-06 | 2.70 | up | 7.93E-03 | 2.73 | up |
| A_23_P168556 | *STX1A* | 7.50E-06 | 2.69 | up | 2.17E-03 | 3.43 | up |
| A_23_P44684 | *ECT2* | 5.38E-08 | 2.68 | up | 3.38E-03 | 3.87 | up |
| A_24_P287941 | *PSMC3IP* | 1.20E-07 | 2.68 | up | 5.92E-03 | 2.88 | up |
| A_23_P20022 | *C7orf68* | 1.06E-09 | 2.67 | up | 3.11E-03 | 3.80 | up |
| A_23_P17826 | *SLC5A1* | 2.08E-06 | 2.67 | up | 2.05E-03 | 2.48 | up |
| A_24_P383450 | *IER5L* | 6.74E-06 | 2.67 | up | 1.68E-03 | 4.07 | up |
| A_23_P118834 | *TOP2A* | 7.71E-06 | 2.66 | up | 9.54E-03 | 2.94 | up |
| A_23_P92441 | *MAD2L1* | 4.30E-07 | 2.65 | up | 1.16E-03 | 3.74 | up |
| A_32_P163858 | *SCD* | 4.71E-04 | 2.64 | up | 4.55E-03 | 4.09 | up |
| A_23_P27656 | *C19orf48* | 6.44E-07 | 2.63 | up | 2.39E-03 | 3.51 | up |
| A_32_P19840 |  | 1.94E-03 | 2.63 | up | 1.94E-03 | 2.75 | up |
| A_23_P23303 | *EXO1* | 2.52E-07 | 2.63 | up | 5.19E-03 | 3.57 | up |
| A_24_P413126 | *PMEPA1* | 3.16E-05 | 2.63 | up | 5.31E-03 | 4.64 | up |
| A_32_P226768 |  | 6.12E-06 | 2.62 | up | 3.60E-03 | 3.59 | up |
| A_23_P132175 | *RTN4R* | 4.60E-07 | 2.61 | up | 1.94E-03 | 3.17 | up |
| A_23_P52278 | *KIF11* | 1.89E-06 | 2.59 | up | 1.12E-03 | 3.88 | up |
| A_32_P76720 | *NT5DC3* | 3.24E-05 | 2.59 | up | 7.65E-03 | 2.42 | up |
| A_23_P207537 | *DUSP14* | 7.47E-09 | 2.59 | up | 1.03E-03 | 4.59 | up |
| A_23_P82420 | *STX1A* | 7.10E-06 | 2.59 | up | 1.99E-03 | 3.17 | up |
| A_23_P57379 | *CDC45L* | 7.93E-06 | 2.59 | up | 4.08E-03 | 3.43 | up |
| A_23_P401904 | *PHF19* | 5.02E-07 | 2.58 | up | 3.37E-03 | 3.94 | up |
| A_23_P202206 | *GSTO2* | 6.11E-08 | 2.57 | up | 1.86E-03 | 2.44 | up |
| A_23_P88331 | *DLGAP5* | 9.12E-07 | 2.57 | up | 2.48E-03 | 3.27 | up |
| A_23_P9574 | *ECT2* | 1.22E-06 | 2.57 | up | 9.62E-04 | 3.98 | up |
| A_23_P44836 | *NT5DC2* | 9.84E-06 | 2.56 | up | 2.34E-03 | 3.43 | up |
| A_24_P171549 | *CDCA7* | 2.40E-06 | 2.56 | up | 3.01E-03 | 2.34 | up |
| A_23_P82738 | *RAD54B* | 9.74E-08 | 2.56 | up | 7.64E-04 | 2.87 | up |
| A_23_P36464 | *C12orf11* | 1.35E-07 | 2.56 | up | 2.49E-03 | 2.13 | up |
| A_24_P176714 | *B9D1* | 3.07E-07 | 2.55 | up | 9.07E-03 | 2.72 | up |
| A_23_P88522 | *NMB* | 2.50E-08 | 2.55 | up | 8.53E-04 | 3.42 | up |
| A_23_P26557 | *C16orf59* | 3.21E-05 | 2.54 | up | 3.03E-03 | 3.19 | up |
| A_23_P38505 | *CXCL16* | 4.92E-08 | 2.54 | up | 6.54E-03 | 2.30 | up |
| A_23_P110802 | *CENPH* | 5.47E-08 | 2.54 | up | 2.29E-03 | 2.46 | up |
| A_23_P70249 | *CDC25C* | 2.04E-05 | 2.53 | up | 3.65E-03 | 2.92 | up |
| A_24_P915692 | *PHLDA1* | 5.27E-05 | 2.53 | up | 1.54E-03 | 3.79 | up |
| A_23_P27947 | *PDCD2L* | 6.08E-10 | 2.53 | up | 1.16E-03 | 3.28 | up |
| A_23_P52298 | *NPM3* | 5.76E-06 | 2.52 | up | 1.11E-03 | 3.10 | up |
| A_23_P106145 | *ERO1L* | 1.18E-04 | 2.50 | up | 1.81E-03 | 3.26 | up |
| A_23_P349676 | *FBXO41* | 3.67E-04 | 2.50 | up | 3.25E-03 | 3.30 | up |
| A_23_P167674 | *F12* | 2.88E-05 | 2.49 | up | 4.36E-03 | 2.97 | up |
| A_23_P60405 | *DDX31* | 1.45E-06 | 2.48 | up | 2.15E-03 | 2.28 | up |
| A_23_P124417 | *BUB1* | 3.00E-08 | 2.48 | up | 1.20E-03 | 3.71 | up |
| A_32_P171328 | *UBE2S* | 6.95E-06 | 2.47 | up | 9.85E-04 | 4.61 | up |
| A_23_P259692 | *PSAT1* | 4.48E-03 | 2.47 | up | 5.72E-03 | 6.95 | up |
| A_32_P140262 |  | 4.96E-06 | 2.47 | up | 2.98E-03 | 3.48 | up |
| A_23_P66608 | *KAT2A* | 6.10E-06 | 2.47 | up | 4.48E-03 | 2.13 | up |
| A_23_P49878 | *FAM64A* | 1.89E-06 | 2.47 | up | 2.70E-03 | 3.16 | up |
| A_23_P16078 | *PAFAH1B3* | 8.42E-07 | 2.46 | up | 1.77E-03 | 3.33 | up |
| A_23_P399726 | *C22orf29* | 1.39E-07 | 2.46 | up | 7.65E-03 | 2.23 | up |
| A_23_P130194 | *PYCR1* | 5.32E-07 | 2.46 | up | 3.37E-03 | 3.08 | up |
| A_23_P44195 | *MSI2* | 1.23E-07 | 2.45 | up | 2.87E-03 | 3.39 | up |
| A_23_P123193 | *ACTR3B* | 1.06E-07 | 2.45 | up | 2.88E-03 | 2.00 | up |
| A_23_P138507 | *CDK1* | 1.51E-05 | 2.45 | up | 1.56E-03 | 3.97 | up |
| A_23_P204751 | *ACCN2* | 9.61E-03 | 2.44 | up | 6.47E-03 | 4.04 | up |
| A_23_P345460 | *PLEKHG4* | 2.82E-04 | 2.44 | up | 3.54E-03 | 3.18 | up |
| A_23_P370989 | *MCM4* | 3.03E-07 | 2.44 | up | 8.51E-04 | 3.10 | up |
| A_23_P122197 | *CCNB1* | 8.77E-07 | 2.43 | up | 4.01E-03 | 3.23 | up |
| A_24_P227831 | *ABCC1* | 2.59E-06 | 2.43 | up | 1.01E-03 | 2.48 | up |
| A_24_P392109 | *CENPN* | 1.62E-06 | 2.42 | up | 7.31E-03 | 3.40 | up |
| A_23_P71727 | *CKS2* | 3.02E-06 | 2.40 | up | 2.25E-03 | 3.24 | up |
| A_23_P68610 | *TPX2* | 6.12E-06 | 2.40 | up | 2.63E-03 | 4.13 | up |
| A_23_P80032 | *E2F1* | 1.10E-04 | 2.40 | up | 1.48E-03 | 4.39 | up |
| A_23_P143190 | *MYBL2* | 3.96E-05 | 2.40 | up | 8.37E-03 | 5.19 | up |
| A_23_P46309 | *RCC1* | 1.75E-06 | 2.40 | up | 3.65E-03 | 2.29 | up |
| A_23_P411335 | *SGOL2* | 7.59E-07 | 2.39 | up | 7.42E-03 | 2.08 | up |
| A_23_P61487 | *LRRC20* | 1.36E-07 | 2.39 | up | 4.50E-04 | 3.35 | up |
| A_23_P104617 | *GYLTL1B* | 1.82E-04 | 2.39 | up | 1.86E-03 | 3.69 | up |
| A_23_P14432 |  | 4.65E-06 | 2.39 | up | 6.77E-03 | 2.01 | up |
| A_32_P71447 | *NCAPD3* | 1.51E-07 | 2.39 | up | 2.27E-03 | 2.47 | up |
| A_23_P72737 | *IFITM1* | 1.52E-05 | 2.39 | up | 1.03E-03 | 3.21 | up |
| A_24_P268676 | *BHLHE40* | 6.13E-04 | 2.39 | up | 3.24E-03 | 2.29 | up |
| A_23_P133956 | *KIFC1* | 3.15E-05 | 2.38 | up | 5.78E-03 | 2.89 | up |
| A_24_P190168 | *TMEM97* | 6.88E-08 | 2.37 | up | 2.49E-03 | 2.78 | up |
| A_23_P31584 | *RABL5* | 2.20E-06 | 2.37 | up | 9.62E-04 | 2.30 | up |
| A_23_P14193 | *RFC3* | 4.57E-07 | 2.37 | up | 1.11E-03 | 3.80 | up |
| A_23_P35219 | *NEK2* | 2.20E-06 | 2.37 | up | 2.88E-03 | 3.63 | up |
| A_23_P250607 | *PLS3* | 2.09E-04 | 2.37 | up | 7.61E-03 | 2.41 | up |
| A_23_P115872 | *CEP55* | 1.54E-03 | 2.36 | up | 7.76E-03 | 4.03 | up |
| A_23_P370097 | *ALS2CR4* | 1.27E-09 | 2.36 | up | 3.25E-03 | 2.43 | up |
| A_23_P323751 | *FAM83D* | 1.93E-05 | 2.36 | up | 2.25E-03 | 4.23 | up |
| A_23_P397341 | *PAQR4* | 1.86E-04 | 2.36 | up | 2.54E-03 | 3.29 | up |
| A_23_P399501 | *PKM2* | 4.93E-06 | 2.35 | up | 3.60E-03 | 2.21 | up |
| A_23_P150935 | *TROAP* | 6.46E-05 | 2.35 | up | 1.27E-03 | 2.73 | up |
| A_32_P109296 | *C15orf42* | 5.04E-05 | 2.35 | up | 6.54E-04 | 3.02 | up |
| A_23_P382775 | *BBC3* | 6.28E-07 | 2.35 | up | 8.58E-03 | 2.32 | up |
| A_24_P218979 | *CDCA3* | 1.36E-06 | 2.34 | up | 3.99E-03 | 3.18 | up |
| A_23_P66211 | *PAQR4* | 2.90E-05 | 2.34 | up | 4.76E-03 | 2.96 | up |
| A_23_P118815 | *BIRC5* | 1.46E-06 | 2.34 | up | 1.34E-03 | 3.72 | up |
| A_24_P385341 | *C1orf107* | 4.72E-09 | 2.34 | up | 8.51E-04 | 2.45 | up |
| A_23_P104651 | *CDCA5* | 5.96E-07 | 2.34 | up | 1.20E-03 | 3.49 | up |
| A_23_P251421 | *CDCA7* | 4.71E-05 | 2.34 | up | 4.45E-03 | 2.19 | up |
| A_23_P6802 | *RRP9* | 1.05E-06 | 2.34 | up | 4.07E-03 | 2.23 | up |
| A_24_P4054 | *TRIP6* | 3.95E-03 | 2.33 | up | 9.18E-03 | 2.70 | up |
| A_23_P217049 | *NCS1* | 2.14E-04 | 2.33 | up | 3.61E-03 | 3.53 | up |
| A_23_P201636 | *LAMC2* | 1.87E-05 | 2.33 | up | 2.05E-03 | 2.13 | up |
| A_23_P58321 | *CCNA2* | 4.06E-05 | 2.33 | up | 2.63E-03 | 3.45 | up |
| A_23_P206059 | *PRC1* | 6.21E-06 | 2.32 | up | 2.92E-03 | 2.87 | up |
| A_24_P124550 | *CCND1* | 1.97E-05 | 2.32 | up | 1.36E-03 | 2.68 | up |
| A_23_P164826 | *RNASEH2A* | 4.51E-06 | 2.31 | up | 2.19E-03 | 2.56 | up |
| A_23_P420981 | *C14orf79* | 1.41E-07 | 2.31 | up | 9.95E-04 | 2.54 | up |
| A_23_P415443 | *NCAPH* | 1.47E-06 | 2.31 | up | 1.12E-03 | 3.08 | up |
| A_32_P171923 | *MACC1* | 1.53E-04 | 2.31 | up | 8.86E-03 | 3.46 | up |
| A_23_P24997 | *CDK4* | 4.03E-05 | 2.30 | up | 1.13E-03 | 2.44 | up |
| A_23_P168747 | *NCAPG2* | 8.34E-05 | 2.29 | up | 2.25E-03 | 2.47 | up |
| A_23_P314151 | *NOLC1* | 2.38E-07 | 2.29 | up | 3.89E-03 | 2.74 | up |
| A_23_P52082 | *INTS7* | 3.22E-12 | 2.28 | up | 3.52E-03 | 2.09 | up |
| A_23_P17739 | *ZNF74* | 9.83E-07 | 2.28 | up | 3.18E-03 | 2.28 | up |
| A_23_P57089 | *PMEPA1* | 1.64E-04 | 2.27 | up | 3.92E-03 | 4.47 | up |
| A_23_P141315 | *NLE1* | 1.70E-08 | 2.27 | up | 4.98E-03 | 2.19 | up |
| A_24_P7040 |  | 2.71E-04 | 2.26 | up | 1.02E-03 | 3.04 | up |
| A_23_P138465 | *NOLC1* | 3.47E-05 | 2.26 | up | 6.76E-03 | 2.75 | up |
| A_23_P106822 | *NOB1* | 2.46E-09 | 2.25 | up | 1.18E-03 | 2.07 | up |
| A_23_P52286 | *DPCD* | 1.04E-07 | 2.25 | up | 4.21E-03 | 2.38 | up |
| A_23_P51085 | *SPC25* | 1.97E-04 | 2.25 | up | 4.54E-03 | 3.63 | up |
| A_24_P105102 | *PKMYT1* | 2.45E-05 | 2.25 | up | 4.48E-03 | 3.07 | up |
| A_24_P204358 | *PYCR1* | 2.38E-04 | 2.24 | up | 2.87E-03 | 3.12 | up |
| A_23_P80098 | *GART* | 1.10E-09 | 2.24 | up | 6.29E-03 | 2.03 | up |
| A_23_P163099 | *POLE2* | 2.92E-06 | 2.24 | up | 7.87E-03 | 2.34 | up |
| A_24_P340866 |  | 1.49E-06 | 2.24 | up | 1.38E-03 | 2.51 | up |
| A_24_P916195 |  | 5.88E-05 | 2.24 | up | 5.73E-03 | 2.60 | up |
| A_23_P158053 | *C9orf16* | 7.75E-07 | 2.24 | up | 6.00E-03 | 2.04 | up |
| A_23_P36928 | *POLR1D* | 6.35E-07 | 2.23 | up | 4.21E-03 | 2.18 | up |
| A_23_P333420 | *RANGAP1* | 2.77E-06 | 2.23 | up | 2.79E-03 | 2.12 | up |
| A_23_P161338 | *PPA1* | 2.72E-09 | 2.23 | up | 2.84E-03 | 2.44 | up |
| A_23_P100344 | *ORC6L* | 3.24E-05 | 2.22 | up | 1.92E-03 | 4.02 | up |
| A_24_P200427 | *PAICS* | 4.36E-06 | 2.22 | up | 3.21E-03 | 2.29 | up |
| A_32_P147241 | *PKM2* | 4.86E-06 | 2.21 | up | 3.32E-03 | 2.13 | up |
| A_24_P57367 | *AHCY* | 5.14E-04 | 2.21 | up | 9.93E-03 | 3.09 | up |
| A_23_P57667 | *PLXNA1* | 2.82E-07 | 2.21 | up | 6.54E-04 | 2.62 | up |
| A_23_P140738 | *FAM38A* | 9.56E-06 | 2.21 | up | 1.17E-03 | 2.11 | up |
| A_24_P516215 | *NOB1* | 3.19E-05 | 2.20 | up | 1.18E-03 | 2.33 | up |
| A_23_P158596 | *AGTRAP* | 1.43E-06 | 2.20 | up | 7.64E-04 | 2.56 | up |
| A_23_P71146 | *POLD2* | 8.44E-06 | 2.20 | up | 2.87E-03 | 2.50 | up |
| A_24_P151920 | *TMEM97* | 5.71E-07 | 2.19 | up | 1.95E-03 | 2.67 | up |
| A_23_P146584 | *C9orf30* | 6.15E-08 | 2.19 | up | 2.50E-03 | 2.28 | up |
| A_24_P626931 | *UBE2CBP* | 1.09E-09 | 2.18 | up | 6.19E-03 | 2.05 | up |
| A_23_P37704 | *CDT1* | 2.50E-05 | 2.18 | up | 3.60E-03 | 2.51 | up |
| A_23_P18579 | *PTTG2* | 1.83E-04 | 2.17 | up | 4.63E-03 | 3.12 | up |
| A_23_P92261 | *ECE2* | 1.70E-05 | 2.17 | up | 4.20E-03 | 2.68 | up |
| A_23_P82478 | *PUS7* | 3.70E-06 | 2.17 | up | 6.85E-03 | 2.48 | up |
| A_23_P202837 | *CCND1* | 6.40E-07 | 2.17 | up | 8.51E-04 | 2.45 | up |
| A_23_P65110 | *RACGAP1* | 3.65E-06 | 2.17 | up | 1.75E-03 | 2.65 | up |
| A_23_P67708 | *TCF3* | 3.87E-07 | 2.16 | up | 3.82E-03 | 2.30 | up |
| A_24_P761727 |  | 1.28E-04 | 2.16 | up | 5.51E-03 | 2.41 | up |
| A_23_P133995 | *PPIL1* | 2.35E-08 | 2.16 | up | 5.45E-03 | 2.41 | up |
| A_32_P201521 | *TMEM97* | 1.44E-05 | 2.16 | up | 2.58E-03 | 2.70 | up |
| A_23_P115482 | *UBE2T* | 1.72E-06 | 2.16 | up | 3.03E-03 | 3.52 | up |
| A_23_P34788 | *KIF2C* | 8.42E-05 | 2.15 | up | 8.53E-04 | 3.48 | up |
| A_24_P317135 | *EXOSC7* | 4.99E-09 | 2.15 | up | 7.33E-04 | 2.02 | up |
| A_23_P340909 | *SKA3* | 1.05E-04 | 2.15 | up | 1.86E-03 | 4.65 | up |
| A_24_P506977 | *C7orf40* | 4.64E-05 | 2.15 | up | 6.52E-04 | 2.64 | up |
| A_23_P143047 | *ATP6V1E2* | 1.83E-05 | 2.14 | up | 8.93E-03 | 2.12 | up |
| A_23_P106998 | *MRPS23* | 7.08E-08 | 2.14 | up | 3.18E-03 | 2.10 | up |
| A_23_P62959 | *PHLDA3* | 3.07E-04 | 2.14 | up | 9.33E-03 | 2.46 | up |
| A_23_P46539 | *PSRC1* | 1.42E-05 | 2.13 | up | 4.13E-03 | 2.48 | up |
| A_23_P57306 | *CHAF1B* | 9.70E-06 | 2.13 | up | 2.70E-03 | 2.93 | up |
| A_23_P82169 | *SOX4* | 2.31E-05 | 2.13 | up | 6.22E-03 | 2.38 | up |
| A_23_P74349 | *NUF2* | 5.28E-05 | 2.13 | up | 2.19E-03 | 3.52 | up |
| A_23_P145197 | *BYSL* | 9.44E-07 | 2.13 | up | 5.95E-03 | 2.36 | up |
| A_23_P24444 | *DHCR7* | 3.76E-06 | 2.12 | up | 1.63E-03 | 2.80 | up |
| A_23_P359497 | *TMEM231* | 7.36E-06 | 2.12 | up | 7.25E-03 | 2.39 | up |
| A_23_P31143 | *TPD52L1* | 2.17E-03 | 2.12 | up | 7.68E-03 | 3.25 | up |
| A_23_P119789 | *TMEM185B* | 4.67E-07 | 2.12 | up | 9.48E-03 | 2.44 | up |
| A_32_P170925 | *TXNRD3* | 6.08E-06 | 2.12 | up | 1.16E-03 | 2.59 | up |
| A_24_P254933 |  | 3.69E-04 | 2.12 | up | 9.84E-04 | 3.02 | up |
| A_23_P62115 | *TIMP1* | 3.34E-04 | 2.11 | up | 2.25E-03 | 4.39 | up |
| A_23_P71904 | *METTL11A* | 5.74E-07 | 2.11 | up | 2.91E-03 | 2.36 | up |
| A_24_P266037 | *DIMT1L* | 3.64E-08 | 2.11 | up | 2.40E-03 | 2.18 | up |
| A_24_P193011 | *CCND1* | 1.16E-04 | 2.10 | up | 8.54E-04 | 2.70 | up |
| A_23_P162476 | *CDCA3* | 1.55E-05 | 2.10 | up | 4.98E-03 | 3.11 | up |
| A_23_P205216 | *UTP14A* | 9.84E-06 | 2.09 | up | 7.96E-03 | 2.13 | up |
| A_24_P211151 | *EXOSC5* | 1.38E-06 | 2.09 | up | 3.84E-03 | 2.99 | up |
| A_24_P305312 | *BBC3* | 1.53E-06 | 2.09 | up | 6.60E-03 | 2.07 | up |
| A_24_P16124 | *IFITM4P* | 3.39E-04 | 2.09 | up | 9.62E-04 | 3.23 | up |
| A_32_P64919 | *DIAPH3* | 1.03E-04 | 2.08 | up | 6.03E-03 | 2.87 | up |
| A_23_P353436 | *CEP78* | 1.57E-06 | 2.08 | up | 2.96E-03 | 2.43 | up |
| A_23_P135364 | *DTYMK* | 7.76E-07 | 2.08 | up | 3.17E-03 | 2.39 | up |
| A_32_P24165 | *FANCD2* | 1.47E-05 | 2.08 | up | 5.08E-03 | 2.56 | up |
| A_23_P54597 | *RSL1D1* | 1.52E-05 | 2.07 | up | 1.16E-03 | 2.03 | up |
| A_23_P361405 | *HYAL3* | 8.00E-04 | 2.07 | up | 5.86E-03 | 2.29 | up |
| A_23_P151405 | *CKAP2* | 3.17E-04 | 2.07 | up | 7.64E-04 | 2.98 | up |
| A_23_P115149 | *WDR77* | 1.34E-05 | 2.06 | up | 3.13E-03 | 2.02 | up |
| A_23_P102183 |  | 2.65E-05 | 2.06 | up | 1.78E-03 | 2.95 | up |
| A_23_P258964 | *IARS* | 1.34E-06 | 2.06 | up | 4.14E-03 | 2.13 | up |
| A_23_P145584 | *UBE2H* | 1.46E-06 | 2.05 | up | 1.74E-03 | 2.39 | up |
| A_23_P17393 | *CSE1L* | 2.51E-08 | 2.05 | up | 2.32E-03 | 3.28 | up |
| A_23_P162970 | *IPO4* | 4.51E-05 | 2.05 | up | 9.44E-03 | 2.30 | up |
| A_23_P115861 | *ZNF485* | 1.84E-08 | 2.05 | up | 7.30E-03 | 2.05 | up |
| A_23_P155815 | *NCAPG* | 9.74E-05 | 2.05 | up | 8.51E-04 | 2.95 | up |
| A_24_P148811 | *RUVBL1* | 1.48E-05 | 2.05 | up | 2.70E-03 | 2.76 | up |
| A_23_P21436 | *PHF19* | 2.65E-05 | 2.04 | up | 4.47E-03 | 2.13 | up |
| A_23_P60899 | *TGS1* | 3.11E-05 | 2.04 | up | 5.92E-03 | 2.01 | up |
| A_23_P717 | *TMEM206* | 8.15E-09 | 2.04 | up | 8.53E-04 | 2.94 | up |
| A_23_P344853 | *WDR43* | 4.26E-06 | 2.03 | up | 9.53E-04 | 2.27 | up |
| A_24_P258073 | *C10orf2* | 3.43E-06 | 2.03 | up | 8.51E-04 | 2.52 | up |
| A_23_P90612 | *MCM6* | 9.15E-06 | 2.03 | up | 8.55E-03 | 2.08 | up |
| A_23_P119095 | *PPP1R13L* | 2.58E-04 | 2.03 | up | 2.11E-03 | 2.73 | up |
| A_23_P345212 | *BOD1P* | 3.29E-07 | 2.03 | up | 3.98E-03 | 2.20 | up |
| A_24_P161973 | *ATP11A* | 6.15E-05 | 2.03 | up | 1.94E-03 | 3.22 | up |
| A_23_P17575 | *AHCY* | 2.54E-08 | 2.03 | up | 7.74E-03 | 2.41 | up |
| A_32_P122754 | *C9orf30* | 8.29E-08 | 2.02 | up | 2.22E-03 | 2.20 | up |
| A_32_P44274 | *CHTF18* | 5.43E-05 | 2.02 | up | 3.86E-03 | 2.40 | up |
| A_23_P129014 | *C14orf143* | 8.37E-06 | 2.02 | up | 2.92E-03 | 2.78 | up |
| A_23_P123974 | *DTYMK* | 6.99E-07 | 2.02 | up | 2.25E-03 | 2.31 | up |
| A_23_P57588 | *GTSE1* | 3.57E-04 | 2.02 | up | 3.18E-03 | 2.96 | up |
| A_32_P230720 | *E2F6* | 1.49E-10 | 2.02 | up | 8.51E-04 | 2.27 | up |
| A_24_P176374 | *CDT1* | 1.97E-04 | 2.02 | up | 5.21E-03 | 2.50 | up |
| A_23_P50477 | *BCL2L12* | 2.00E-07 | 2.01 | up | 1.13E-03 | 3.02 | up |
| A_23_P76882 | *CCNB1IP1* | 6.17E-05 | 2.01 | up | 2.56E-03 | 2.27 | up |
| A_23_P31721 | *E2F5* | 9.05E-05 | 2.01 | up | 3.60E-03 | 2.62 | up |
| A_23_P88740 | *CENPN* | 2.40E-05 | 2.01 | up | 6.31E-03 | 3.15 | up |
| A_23_P156842 | *EEF1E1* | 7.86E-06 | 2.01 | up | 3.79E-03 | 2.34 | up |
| A_23_P354297 | *CHTF18* | 7.28E-05 | 2.01 | up | 3.36E-03 | 2.43 | up |
| A_32_P184933 | *UBE2S* | 2.74E-05 | 2.01 | up | 1.70E-03 | 4.56 | up |
| A_23_P253752 | *FAM54A* | 1.26E-04 | 2.01 | up | 4.95E-03 | 3.02 | up |
