# Supplementary material for: A Gene Expression and Pre-mRNA Splicing Signature That Marks the Adenoma-Adenocarcinoma Progression in Colorectal Cancer
Source: PLoS One. 2014 Feb 6;9(2):e87761. doi: 10.1371/journal.pone.0087761 (PMC3916340; doi:10.1371/journal.pone.0087761)
Supplement: Table S7 — KEGG gene sets enriched in colorectal adenoma samples in comparison to normal mucosae. (DOC) [file pone.0087761.s013.doc]

**Table S7. KEGG gene sets enriched in colorectal adenoma samples in comparison to normal mucosae.** The KEGG pathway analysis showed 18 gene sets distinguishing colorectal adenomas from normal mucosae (P-value ≤ 0.05), considering deregulated genes with a 1.1-fold cut-off difference (P-value ≤ 0.01 by *t*-test with FDR).

| Pathway in Colorectal Adenoma *vs*. Normal | P-value | Benjamini-Hochberg | Fold Enrichment | Number of Genes in the Pathway | Number of Deregulated Genes | Percentage of Deregulated Genes |
| --- | --- | --- | --- | --- | --- | --- |
| DNA replication | 4.2E-09 | 8.3E-07 | 2.96 | 36 | 27 | 75% |
| Cell cycle | 2.6E-08 | 2.6E-06 | 1.93 | 125 | 61 | 49% |
| Purine metabolism | 4.3E-05 | 2.8E-03 | 1.60 | 153 | 62 | 41% |
| Spliceosome | 5.3E-05 | 2.6E-03 | 1.66 | 126 | 53 | 42% |
| Pyrimidine metabolism | 1.0E-04 | 3.9E-03 | 1.74 | 95 | 42 | 44% |
| Nucleotide excision repair | 3.5E-04 | 1.1E-02 | 2.06 | 44 | 23 | 52% |
| Mismatch repair | 1.4E-03 | 3.8E-02 | 2.40 | 23 | 14 | 61% |
| RNA degradation | 1.7E-03 | 4.0E-02 | 1.80 | 57 | 26 | 46% |
| Base excision repair | 2.5E-03 | 5.2E-02 | 2.03 | 35 | 18 | 51% |
| Oocyte meiosis | 3.4E-03 | 6.5E-02 | 1.51 | 110 | 42 | 38% |
| Progesterone-mediated oocyte maturation | 4.9E-03 | 8.3E-02 | 1.56 | 86 | 34 | 40% |
| One carbon pool by folate | 8.1E-03 | 1.2E-01 | 2.47 | 16 | 10 | 63% |
| Ribosome | 1.1E-02 | 1.6E-01 | 1.50 | 87 | 33 | 38% |
| Circadian rhythm | 2.6E-02 | 3.1E-01 | 2.43 | 13 | 8 | 62% |
| NOD-like receptor signaling pathway | 2.6E-02 | 2.9E-01 | 1.53 | 62 | 24 | 39% |
| Complement and coagulation cascades | 2.8E-02 | 3.0E-01 | 1.49 | 69 | 26 | 38% |
| Glutathione metabolism | 3.2E-02 | 3.1E-01 | 1.58 | 50 | 20 | 40% |
| Glycosylphosphatidylinositol(GPI)-anchor biosynthesis | 3.2E-02 | 3.0E-01 | 1.89 | 25 | 12 | 48% |
