# Supplementary material for: A Gene Expression and Pre-mRNA Splicing Signature That Marks the Adenoma-Adenocarcinoma Progression in Colorectal Cancer
Source: PLoS One. 2014 Feb 6;9(2):e87761. doi: 10.1371/journal.pone.0087761 (PMC3916340; doi:10.1371/journal.pone.0087761)
Supplement: Table S8 — Significantly up- and down-regulated genes in colorectal cancer samples in comparison to colorectal adenoma samples. (DOC) [file pone.0087761.s014.doc]

**Table S8. Significantly up- and down-regulated genes in colorectal cancer samples in comparison to colorectal adenoma samples.** The results of 44k Whole Human Genome microarrays (Agilent) for the deregulated genes in CRC *vs*. CRA are presented (≥ 2.0 FC, P-value ≤ 0.01 by *t*-test with FDR).

| Probe Name | Gene Symbol | P-value | Fold-Change | Regulation |
| --- | --- | --- | --- | --- |
| A_23_P51217 | *CLCA1* | 1.81E-06 | 41.59 | down |
| A_23_P2789 | *OLFM4* | 4.43E-05 | 18.67 | down |
| A_23_P36018 | *VSIG2* | 1.16E-07 | 12.09 | down |
| A_23_P142878 | *ATOH8* | 1.19E-07 | 11.08 | down |
| A_32_P234184 | *HES5* | 2.67E-06 | 10.83 | down |
| A_23_P92196 | *RETNLB* | 1.97E-03 | 9.65 | down |
| A_23_P151851 | *DUOX2* | 2.51E-05 | 9.03 | down |
| A_32_P23125 | *C20orf56* | 2.72E-04 | 8.59 | down |
| A_23_P358714 | *KIAA1324* | 3.57E-06 | 8.44 | down |
| A_24_P35905 | *DUOX2* | 3.03E-05 | 8.00 | down |
| A_23_P320216 | *FAM55D* | 2.00E-05 | 7.76 | down |
| A_23_P156826 | *C6orf105* | 9.22E-05 | 6.66 | down |
| A_23_P256784 | *MUC2* | 2.01E-03 | 6.43 | down |
| A_24_P365015 | *HOXB13* | 3.54E-04 | 6.31 | down |
| A_23_P149427 | *CAPN9* | 1.07E-04 | 6.14 | down |
| A_24_P208825 | *MUC4* | 1.84E-04 | 5.98 | down |
| A_23_P160336 | *LEFTY1* | 7.18E-04 | 5.90 | down |
| A_23_P42811 | *AGR3* | 5.00E-05 | 5.85 | down |
| A_23_P61042 | *IGHA2* | 7.54E-03 | 5.80 | down |
| A_23_P31798 | *NAT2* | 4.61E-08 | 5.71 | down |
| A_24_P181254 | *OLFM4* | 3.09E-04 | 5.69 | down |
| A_23_P21495 | *FCGBP* | 3.87E-04 | 5.69 | down |
| A_23_P4212 | *HOXB13* | 2.83E-04 | 5.40 | down |
| A_23_P257993 | *DNASE1L3* | 8.39E-06 | 5.34 | down |
| A_23_P24543 | *FAM55A* | 1.42E-05 | 5.24 | down |
| A_23_P86021 | *SELENBP1* | 2.87E-07 | 5.15 | down |
| A_23_P103588 | *HMGCS2* | 6.21E-03 | 5.11 | down |
| A_23_P77731 | *CRYM* | 8.21E-06 | 4.98 | down |
| A_23_P74619 | *SELENBP1* | 8.45E-05 | 4.84 | down |
| A_32_P180336 | *C11orf92* | 5.92E-05 | 4.77 | down |
| A_23_P16225 | *BEST2* | 9.80E-03 | 4.76 | down |
| A_23_P430670 | *CHST5* | 5.29E-03 | 4.75 | down |
| A_23_P59375 | *ID4* | 3.78E-06 | 4.67 | down |
| A_23_P60599 | *UGT1A6* | 1.43E-04 | 4.67 | down |
| A_24_P655849 | *SMAD9* | 3.38E-05 | 4.63 | down |
| A_23_P149517 | *PIGR* | 1.80E-03 | 4.61 | down |
| A_23_P254741 | *SOD3* | 1.57E-04 | 4.58 | down |
| A_23_P6433 | *MB* | 4.02E-04 | 4.56 | down |
| A_23_P169437 | *LCN2* | 2.19E-03 | 4.51 | down |
| A_23_P25674 | *CKB* | 1.50E-03 | 4.45 | down |
| A_23_P8640 | *GPER* | 2.50E-04 | 4.44 | down |
| A_23_P2814 | *SMAD9* | 5.95E-06 | 4.39 | down |
| A_24_P225534 | *RHBDL2* | 5.83E-05 | 4.28 | down |
| A_23_P258194 | *AIFM3* | 8.98E-06 | 4.27 | down |
| A_23_P218111 | *SERPINA1* | 2.67E-04 | 4.25 | down |
| A_23_P27013 | *HOXB9* | 1.10E-03 | 4.20 | down |
| A_32_P39855 | *C1orf125* | 9.78E-03 | 4.19 | down |
| A_23_P16915 | *QPCT* | 7.18E-04 | 4.16 | down |
| A_23_P250164 | *HGD* | 1.11E-03 | 4.15 | down |
| A_32_P46571 | *RHBDL2* | 4.97E-05 | 4.04 | down |
| A_23_P233 | *FMO5* | 1.06E-05 | 4.02 | down |
| A_23_P101972 | *CAPN13* | 7.34E-03 | 3.97 | down |
| A_24_P220947 | *AKR1C1* | 2.37E-04 | 3.95 | down |
| A_24_P602871 | *SAMD5* | 1.16E-03 | 3.95 | down |
| A_23_P54968 | *ST6GALNAC1* | 1.42E-03 | 3.91 | down |
| A_23_P164814 | *C19orf57* | 1.05E-05 | 3.76 | down |
| A_23_P69326 | *CADPS* | 9.09E-04 | 3.72 | down |
| A_24_P79040 | *CAPN12* | 7.90E-04 | 3.72 | down |
| A_23_P203698 | *MOGAT2* | 1.57E-04 | 3.69 | down |
| A_23_P26854 | *RICH2* | 7.88E-04 | 3.68 | down |
| A_32_P14737 |  | 6.04E-06 | 3.68 | down |
| A_23_P63432 | *RHBDL2* | 1.92E-04 | 3.67 | down |
| A_23_P126248 | *RNF186* | 1.84E-04 | 3.66 | down |
| A_24_P340286 | *CHADL* | 9.85E-04 | 3.65 | down |
| A_23_P218247 | *CES3* | 8.80E-05 | 3.64 | down |
| A_23_P41145 | *FAM3D* | 8.25E-05 | 3.61 | down |
| A_23_P148015 | *AXIN2* | 6.85E-05 | 3.61 | down |
| A_24_P286687 | *ZDHHC2* | 1.12E-05 | 3.59 | down |
| A_23_P138492 | *NEURL* | 4.00E-04 | 3.58 | down |
| A_23_P165783 | *MLPH* | 1.69E-04 | 3.57 | down |
| A_23_P118392 | *RASD1* | 2.04E-03 | 3.55 | down |
| A_23_P167168 | *IGJ* | 2.42E-03 | 3.55 | down |
| A_23_P214281 | *PAQR8* | 1.19E-07 | 3.53 | down |
| A_32_P154361 |  | 3.02E-03 | 3.52 | down |
| A_23_P17420 | *BCAS1* | 2.26E-05 | 3.50 | down |
| A_24_P266048 | *FAM134B* | 1.02E-04 | 3.48 | down |
| A_23_P45786 | *COL9A2* | 2.83E-04 | 3.46 | down |
| A_24_P71468 | *QPCT* | 2.99E-03 | 3.44 | down |
| A_23_P104484 | *GPR120* | 7.01E-03 | 3.44 | down |
| A_23_P207632 | *ATP2A3* | 9.04E-05 | 3.42 | down |
| A_24_P169873 | *IGHA2* | 2.41E-03 | 3.41 | down |
| A_23_P28898 | *PLCB4* | 1.08E-03 | 3.37 | down |
| A_23_P357207 | *MRAP2* | 6.62E-04 | 3.36 | down |
| A_23_P363316 | *HOXB5* | 4.77E-03 | 3.33 | down |
| A_32_P190036 |  | 8.39E-06 | 3.33 | down |
| A_23_P135123 |  | 1.66E-04 | 3.32 | down |
| A_32_P462013 |  | 3.36E-05 | 3.28 | down |
| A_23_P379864 | *ASRGL1* | 2.09E-03 | 3.27 | down |
| A_23_P319583 | *RIMS3* | 5.05E-05 | 3.27 | down |
| A_24_P211106 | *TNFRSF11A* | 2.08E-04 | 3.26 | down |
| A_23_P121622 | *SULT1B1* | 4.49E-04 | 3.25 | down |
| A_23_P16409 | *CAPN12* | 2.55E-03 | 3.25 | down |
| A_23_P38334 | *WNK4* | 5.85E-05 | 3.23 | down |
| A_23_P54770 | *APOB48R* | 3.38E-06 | 3.23 | down |
| A_23_P202978 | *CASP1* | 2.73E-05 | 3.20 | down |
| A_23_P94103 | *SCARA5* | 1.27E-03 | 3.17 | down |
| A_32_P217140 | *ISX* | 2.19E-03 | 3.14 | down |
| A_23_P137381 | *ID3* | 2.40E-05 | 3.13 | down |
| A_24_P191781 | *PARM1* | 3.54E-04 | 3.12 | down |
| A_23_P165778 | *MLPH* | 8.36E-05 | 3.07 | down |
| A_23_P116173 | *C11orf93* | 9.46E-04 | 3.07 | down |
| A_23_P118633 | *SPATA20* | 9.82E-06 | 3.06 | down |
| A_23_P58796 | *RGMB* | 4.67E-03 | 3.06 | down |
| A_23_P259594 | *AKAP7* | 7.25E-04 | 3.05 | down |
| A_23_P257583 | *DENND2A* | 3.12E-04 | 3.04 | down |
| A_23_P16252 | *KLK1* | 2.91E-03 | 3.04 | down |
| A_23_P252306 | *ID1* | 3.02E-04 | 3.04 | down |
| A_24_P334640 | *PAQR8* | 4.61E-07 | 3.03 | down |
| A_24_P648880 | *MEIS3P1* | 8.03E-05 | 3.03 | down |
| A_23_P317756 | *ACSM3* | 1.11E-03 | 3.03 | down |
| A_23_P322562 | *NEURL* | 1.27E-03 | 3.03 | down |
| A_23_P167599 | *FAM134B* | 1.96E-04 | 2.99 | down |
| A_23_P215549 | *PON3* | 7.39E-03 | 2.97 | down |
| A_23_P83339 | *RNF183* | 2.11E-03 | 2.97 | down |
| A_23_P46390 | *SYTL1* | 1.12E-03 | 2.94 | down |
| A_23_P53530 | *MTERFD3* | 3.20E-09 | 2.93 | down |
| A_23_P304921 | *NOX1* | 3.58E-03 | 2.92 | down |
| A_23_P392470 | *NR3C2* | 3.79E-04 | 2.92 | down |
| A_23_P11629 | *TMEM61* | 8.69E-03 | 2.91 | down |
| A_23_P204630 | *NTN4* | 8.13E-05 | 2.89 | down |
| A_32_P149011 |  | 2.41E-03 | 2.89 | down |
| A_24_P379820 | *ITM2C* | 1.14E-03 | 2.87 | down |
| A_32_P205624 | *SHC2* | 2.36E-03 | 2.86 | down |
| A_24_P156490 | *KCNMA1* | 1.14E-03 | 2.85 | down |
| A_24_P218814 | *RDH5* | 7.18E-04 | 2.85 | down |
| A_23_P102391 | *SLC40A1* | 3.48E-05 | 2.85 | down |
| A_23_P45011 | *PPP1R14C* | 2.55E-03 | 2.80 | down |
| A_23_P209799 | *MYO7B* | 9.32E-04 | 2.79 | down |
| A_23_P21485 | *PID1* | 2.19E-04 | 2.77 | down |
| A_23_P146572 | *NPDC1* | 7.77E-06 | 2.77 | down |
| A_23_P217280 | *NOX1* | 8.74E-03 | 2.77 | down |
| A_23_P8834 | *EPHX2* | 4.36E-05 | 2.77 | down |
| A_23_P390518 | *TNFRSF11A* | 1.12E-03 | 2.77 | down |
| A_24_P71341 | *FMO5* | 2.62E-06 | 2.76 | down |
| A_24_P62708 | *PRKACB* | 6.40E-04 | 2.75 | down |
| A_24_P174503 | *AMT* | 2.46E-03 | 2.75 | down |
| A_23_P257164 | *AMT* | 3.00E-03 | 2.74 | down |
| A_23_P18447 | *PPARGC1A* | 2.40E-03 | 2.74 | down |
| A_32_P37592 | *SCARNA17* | 8.06E-03 | 2.73 | down |
| A_23_P256131 | *HR* | 3.16E-05 | 2.73 | down |
| A_32_P217773 | *SYTL1* | 3.49E-03 | 2.71 | down |
| A_23_P406025 | *PRUNE2* | 1.64E-03 | 2.71 | down |
| A_24_P402690 | *ITM2C* | 3.14E-04 | 2.70 | down |
| A_32_P146815 |  | 5.16E-03 | 2.69 | down |
| A_24_P354715 | *NT5E* | 3.46E-03 | 2.69 | down |
| A_23_P14986 | *HSD11B2* | 6.38E-05 | 2.68 | down |
| A_23_P94255 | *TRPA1* | 1.46E-03 | 2.67 | down |
| A_24_P129588 | *GPR120* | 6.82E-03 | 2.66 | down |
| A_23_P501831 | *C5orf4* | 1.40E-04 | 2.66 | down |
| A_23_P364625 | *LRRC19* | 3.52E-03 | 2.65 | down |
| A_23_P159395 | *AXIN2* | 2.02E-03 | 2.65 | down |
| A_23_P95594 | *NAT1* | 2.61E-05 | 2.64 | down |
| A_23_P1722 | *C11orf52* | 3.57E-06 | 2.64 | down |
| A_23_P10194 | *SEZ6L2* | 2.54E-04 | 2.62 | down |
| A_23_P128323 | *SCNN1A* | 1.08E-04 | 2.62 | down |
| A_23_P407112 | *SPATA18* | 1.75E-03 | 2.62 | down |
| A_23_P214079 | *SPINK1* | 2.64E-03 | 2.62 | down |
| A_24_P10233 | *DAPK2* | 4.02E-04 | 2.60 | down |
| A_23_P145824 | *PPP1R9A* | 4.95E-03 | 2.59 | down |
| A_32_P49854 |  | 1.47E-04 | 2.58 | down |
| A_23_P311901 | *ATP10B* | 1.27E-04 | 2.56 | down |
| A_23_P203391 | *ASRGL1* | 3.13E-03 | 2.53 | down |
| A_24_P815062 |  | 3.50E-04 | 2.53 | down |
| A_23_P404902 | *SHROOM1* | 2.44E-04 | 2.53 | down |
| A_23_P55518 | *SMAD7* | 1.82E-04 | 2.52 | down |
| A_24_P867111 | *LOC283177* | 2.80E-03 | 2.49 | down |
| A_32_P170664 |  | 2.01E-03 | 2.49 | down |
| A_24_P782308 | *NEDD4L* | 3.54E-04 | 2.48 | down |
| A_23_P161507 | *MTL5* | 2.29E-03 | 2.48 | down |
| A_24_P844984 | *PIGR* | 1.26E-03 | 2.48 | down |
| A_23_P23705 | *SPATA6* | 2.55E-04 | 2.48 | down |
| A_23_P417415 | *ACOT11* | 8.22E-06 | 2.47 | down |
| A_32_P130788 | *SAMD13* | 1.44E-03 | 2.45 | down |
| A_23_P130027 | *EPN3* | 8.99E-05 | 2.45 | down |
| A_32_P65473 | *KIAA1244* | 7.26E-03 | 2.45 | down |
| A_23_P66767 | *GGT6* | 6.19E-03 | 2.45 | down |
| A_23_P160920 | *PDZK1IP1* | 2.30E-03 | 2.44 | down |
| A_23_P305759 | *ABHD3* | 1.68E-03 | 2.44 | down |
| A_24_P393571 | *GDA* | 4.63E-04 | 2.43 | down |
| A_23_P59616 | *GTF2IRD2* | 2.23E-04 | 2.43 | down |
| A_23_P50638 | *LRG1* | 1.06E-03 | 2.43 | down |
| A_23_P139600 | *RASAL1* | 2.70E-03 | 2.42 | down |
| A_23_P99249 | *SUCLG2* | 1.39E-04 | 2.41 | down |
| A_23_P154400 | *MLPH* | 3.32E-03 | 2.41 | down |
| A_23_P11787 | *WNT4* | 3.52E-03 | 2.40 | down |
| A_24_P112395 | *PBLD* | 3.69E-03 | 2.40 | down |
| A_23_P92073 | *PARP3* | 7.02E-05 | 2.39 | down |
| A_24_P945113 | *ACVRL1* | 1.47E-03 | 2.38 | down |
| A_23_P149998 | *PBLD* | 5.90E-03 | 2.38 | down |
| A_23_P254353 | *NOXA1* | 1.86E-04 | 2.37 | down |
| A_24_P209171 | *SH3BGRL2* | 3.78E-06 | 2.36 | down |
| A_23_P129157 | *NEIL1* | 1.69E-03 | 2.34 | down |
| A_24_P765795 |  | 1.40E-03 | 2.34 | down |
| A_32_P222695 | *FLJ41603* | 4.28E-05 | 2.33 | down |
| A_24_P83118 | *DUSP18* | 3.14E-04 | 2.33 | down |
| A_23_P373031 | *CACNA1C* | 8.79E-03 | 2.32 | down |
| A_24_P90881 | *CES3* | 9.25E-04 | 2.32 | down |
| A_23_P65532 | *PELI2* | 1.42E-03 | 2.32 | down |
| A_23_P500936 | *FOXA2* | 2.60E-03 | 2.31 | down |
| A_23_P34375 | *TCEA3* | 1.50E-03 | 2.30 | down |
| A_23_P72117 | *SMPDL3A* | 2.30E-03 | 2.29 | down |
| A_24_P406034 | *SLC35A1* | 6.33E-05 | 2.29 | down |
| A_24_P105933 | *VIPR1* | 3.54E-04 | 2.29 | down |
| A_24_P623734 | *C2orf72* | 5.64E-03 | 2.28 | down |
| A_23_P205531 | *RNASE4* | 1.26E-03 | 2.28 | down |
| A_23_P70660 | *FAM46A* | 3.18E-03 | 2.28 | down |
| A_23_P98455 | *VWA5A* | 3.63E-04 | 2.27 | down |
| A_23_P48771 | *C14orf159* | 1.68E-05 | 2.26 | down |
| A_23_P129312 | *PPP1R14D* | 2.42E-03 | 2.24 | down |
| A_23_P143348 | *OVOL2* | 2.60E-06 | 2.24 | down |
| A_24_P938006 |  | 5.31E-04 | 2.24 | down |
| A_23_P7882 | *SLC22A23* | 2.83E-04 | 2.23 | down |
| A_24_P402779 | *PARP3* | 1.91E-04 | 2.22 | down |
| A_23_P66454 | *GSDMB* | 6.92E-03 | 2.22 | down |
| A_23_P394304 | *PDZK1IP1* | 3.67E-03 | 2.21 | down |
| A_23_P421011 | *KAZALD1* | 8.19E-04 | 2.21 | down |
| A_23_P34376 | *TCEA3* | 3.66E-03 | 2.21 | down |
| A_23_P307002 |  | 1.08E-03 | 2.21 | down |
| A_23_P77174 | *PIGB* | 1.95E-06 | 2.20 | down |
| A_24_P331128 | *GNA15* | 1.59E-03 | 2.20 | down |
| A_24_P130363 | *C18orf1* | 2.67E-04 | 2.20 | down |
| A_23_P86195 | *SLC44A3* | 8.34E-05 | 2.20 | down |
| A_23_P143935 | *PIGZ* | 4.40E-03 | 2.20 | down |
| A_32_P25437 | *SLC12A2* | 1.86E-04 | 2.19 | down |
| A_24_P66001 | *UQCR10* | 3.53E-04 | 2.19 | down |
| A_23_P167920 | *DLL1* | 2.81E-03 | 2.18 | down |
| A_23_P161686 | *ARHGAP32* | 2.26E-05 | 2.18 | down |
| A_32_P5376 | *NUDT16P1* | 1.88E-04 | 2.18 | down |
| A_32_P166733 |  | 1.83E-05 | 2.18 | down |
| A_32_P229493 |  | 3.90E-03 | 2.17 | down |
| A_23_P24586 | *ACCS* | 1.73E-03 | 2.17 | down |
| A_23_P160433 | *C1orf115* | 2.85E-03 | 2.16 | down |
| A_23_P53193 | *SYTL2* | 3.22E-04 | 2.16 | down |
| A_23_P411612 | *SPRYD4* | 4.42E-06 | 2.15 | down |
| A_24_P319374 | *GPA33* | 6.46E-04 | 2.15 | down |
| A_32_P100109 | *REPS2* | 3.68E-04 | 2.15 | down |
| A_24_P131173 | *C1orf115* | 3.10E-03 | 2.15 | down |
| A_23_P158880 | *STARD5* | 2.27E-04 | 2.15 | down |
| A_23_P212779 | *PARM1* | 7.99E-03 | 2.14 | down |
| A_23_P138541 | *AKR1C3* | 8.46E-03 | 2.14 | down |
| A_32_P153781 |  | 7.42E-03 | 2.14 | down |
| A_23_P215819 | *ZNF655* | 2.31E-03 | 2.14 | down |
| A_23_P50081 | *IMPA2* | 2.18E-03 | 2.14 | down |
| A_23_P136355 | *HHAT* | 6.47E-04 | 2.13 | down |
| A_23_P121926 | *SEPP1* | 6.02E-03 | 2.13 | down |
| A_23_P154875 | *BACE2* | 1.20E-04 | 2.13 | down |
| A_32_P108592 |  | 1.20E-04 | 2.13 | down |
| A_23_P114689 | *ASAP3* | 2.44E-03 | 2.13 | down |
| A_23_P76312 | *GUCY2C* | 2.58E-03 | 2.13 | down |
| A_32_P156564 |  | 5.83E-05 | 2.12 | down |
| A_24_P233995 | *MOSC1* | 1.77E-03 | 2.12 | down |
| A_23_P203095 | *PCSK7* | 2.38E-03 | 2.12 | down |
| A_32_P159289 |  | 5.74E-03 | 2.12 | down |
| A_32_P66974 | *PCSK7* | 1.91E-04 | 2.12 | down |
| A_24_P194508 | *SPIN2B* | 3.70E-04 | 2.11 | down |
| A_24_P327181 | *WNK4* | 5.60E-03 | 2.11 | down |
| A_24_P269598 | *ENTPD4* | 1.19E-07 | 2.11 | down |
| A_23_P65812 | *SMAD6* | 1.21E-04 | 2.10 | down |
| A_32_P91042 | *LOC100129034* | 3.96E-04 | 2.10 | down |
| A_23_P377616 | *CIRBP* | 5.20E-03 | 2.10 | down |
| A_23_P21473 | *CEP70* | 4.94E-04 | 2.10 | down |
| A_23_P210015 | *PTPN18* | 3.17E-05 | 2.10 | down |
| A_24_P14584 | *BACE2* | 1.86E-04 | 2.09 | down |
| A_24_P122337 | *SYTL4* | 7.81E-03 | 2.09 | down |
| A_23_P163227 | *CKMT1A* | 3.18E-03 | 2.09 | down |
| A_23_P41424 | *SLC39A8* | 1.53E-03 | 2.09 | down |
| A_24_P108311 | *NEDD4L* | 1.88E-03 | 2.08 | down |
| A_23_P59452 | *ABP1* | 1.93E-03 | 2.08 | down |
| A_23_P26815 | *RILP* | 5.33E-04 | 2.08 | down |
| A_23_P135239 | *TLE1* | 1.12E-04 | 2.07 | down |
| A_23_P1505 | *LRP5* | 6.84E-05 | 2.07 | down |
| A_23_P58912 | *SLC35A1* | 1.07E-03 | 2.07 | down |
| A_23_P37205 | *NDRG2* | 2.91E-03 | 2.07 | down |
| A_23_P392126 | *C17orf108* | 6.71E-05 | 2.07 | down |
| A_23_P343382 | *GPRIN2* | 4.97E-03 | 2.07 | down |
| A_32_P515920 | *LOC400573* | 3.48E-03 | 2.06 | down |
| A_24_P12413 | *TRAM2* | 1.12E-03 | 2.06 | down |
| A_23_P94819 | *RPH3AL* | 2.99E-04 | 2.06 | down |
| A_23_P363426 | *SFRS2B* | 2.42E-05 | 2.05 | down |
| A_23_P71790 | *MAMDC4* | 1.40E-03 | 2.05 | down |
| A_23_P206177 | *SNAP23* | 1.20E-03 | 2.04 | down |
| A_23_P42375 | *RAB32* | 4.45E-05 | 2.04 | down |
| A_23_P205910 | *SLC24A1* | 5.72E-05 | 2.04 | down |
| A_24_P33652 | *MUDENG* | 1.37E-04 | 2.04 | down |
| A_23_P145895 | *TP53TG1* | 2.90E-03 | 2.03 | down |
| A_23_P398172 | *FAM135A* | 1.14E-04 | 2.03 | down |
| A_23_P29975 | *C4orf19* | 4.58E-05 | 2.02 | down |
| A_23_P345081 | *ZNF655* | 1.26E-03 | 2.02 | down |
| A_24_P930741 | *EPHA10* | 2.16E-03 | 2.02 | down |
| A_24_P526623 | *LOC339290* | 2.03E-03 | 2.02 | down |
| A_23_P207507 | *ABCC3* | 1.02E-03 | 2.02 | down |
| A_23_P431569 | *LOC100049716* | 5.32E-03 | 2.02 | down |
| A_32_P195756 |  | 2.12E-03 | 2.02 | down |
| A_23_P219004 | *NT5DC1* | 1.47E-04 | 2.01 | down |
| A_24_P102343 | *EPN3* | 4.28E-05 | 2.01 | down |
| A_23_P387184 | *NHSL1* | 3.69E-03 | 2.01 | down |
| A_24_P114671 |  | 4.33E-04 | 2.01 | down |
| A_24_P157720 |  | 9.80E-03 | 2.01 | down |
| A_23_P56746 | *FAP* | 2.06E-11 | 29.21 | up |
| A_23_P215328 | *SFRP4* | 1.74E-10 | 22.97 | up |
| A_24_P934546 | *SFRP2* | 2.60E-09 | 15.08 | up |
| A_23_P62021 | *THBS2* | 4.36E-09 | 13.59 | up |
| A_23_P216429 | *ASPN* | 3.22E-12 | 12.48 | up |
| A_23_P7313 | *SPP1* | 1.13E-04 | 11.05 | up |
| A_23_P69030 | *COL8A1* | 9.04E-07 | 10.64 | up |
| A_23_P143981 | *FBLN2* | 3.20E-09 | 10.62 | up |
| A_23_P207520 | *COL1A1* | 2.87E-07 | 10.26 | up |
| A_23_P111888 | *CTHRC1* | 6.10E-07 | 10.14 | up |
| A_24_P119745 | *FN1* | 1.84E-04 | 9.38 | up |
| A_23_P43164 | *SULF1* | 8.86E-08 | 9.14 | up |
| A_24_P137501 | *SFRP2* | 9.82E-06 | 8.72 | up |
| A_23_P57417 | *MMP11* | 1.22E-07 | 8.14 | up |
| A_23_P360754 | *ADAMTS4* | 1.95E-05 | 8.04 | up |
| A_23_P3312 | *ISLR* | 2.18E-08 | 7.45 | up |
| A_23_P395438 | *HTRA3* | 5.39E-08 | 7.30 | up |
| A_23_P58251 | *CPZ* | 1.28E-07 | 7.20 | up |
| A_23_P122924 | *INHBA* | 1.22E-05 | 7.18 | up |
| A_23_P99063 | *LUM* | 1.28E-07 | 7.03 | up |
| A_23_P52697 | *CD248* | 1.86E-07 | 6.48 | up |
| A_23_P166408 | *OSM* | 3.29E-04 | 6.25 | up |
| A_24_P277934 | *COL1A2* | 6.71E-07 | 6.10 | up |
| A_23_P33196 | *COL5A2* | 3.54E-07 | 5.88 | up |
| A_23_P18452 | *CXCL9* | 1.13E-03 | 5.86 | up |
| A_23_P320578 | *RGS16* | 2.79E-07 | 5.80 | up |
| A_23_P121533 | *SPON2* | 7.57E-07 | 5.76 | up |
| A_24_P402242 | *COL3A1* | 6.09E-06 | 5.74 | up |
| A_23_P69573 | *GUCY1A3* | 1.22E-07 | 5.69 | up |
| A_23_P154115 | *IGFBP5* | 3.57E-06 | 5.65 | up |
| A_23_P142533 | *COL3A1* | 4.94E-07 | 5.65 | up |
| A_23_P107963 | *FUT1* | 3.23E-05 | 5.64 | up |
| A_32_P42895 |  | 6.10E-07 | 5.56 | up |
| A_23_P421401 | *PDGFRB* | 3.67E-07 | 5.32 | up |
| A_23_P203419 | *FADS1* | 3.48E-05 | 5.28 | up |
| A_23_P85783 | *PHGDH* | 2.94E-03 | 5.25 | up |
| A_24_P316059 |  | 6.10E-07 | 5.25 | up |
| A_32_P116556 | *ZNF469* | 3.57E-06 | 5.24 | up |
| A_23_P141688 | *RAB31* | 6.69E-07 | 5.23 | up |
| A_24_P236799 | *RAB31* | 2.12E-06 | 5.22 | up |
| A_23_P390504 | *FOXC1* | 5.25E-05 | 5.19 | up |
| A_23_P7642 | *SPARC* | 1.33E-06 | 5.12 | up |
| A_23_P200741 | *DPT* | 3.56E-07 | 5.09 | up |
| A_24_P935491 | *COL3A1* | 3.44E-07 | 5.08 | up |
| A_23_P46426 | *CYR61* | 2.19E-05 | 4.94 | up |
| A_23_P157299 | *AEBP1* | 2.22E-05 | 4.86 | up |
| A_23_P46429 | *CYR61* | 6.04E-06 | 4.81 | up |
| A_23_P301521 | *KIAA1462* | 3.48E-05 | 4.70 | up |
| A_23_P131846 | *SNAI1* | 2.62E-06 | 4.69 | up |
| A_23_P10391 | *COL5A2* | 2.06E-05 | 4.69 | up |
| A_24_P370946 | *CYR61* | 4.28E-05 | 4.67 | up |
| A_23_P206022 | *ITGA11* | 2.45E-07 | 4.66 | up |
| A_23_P38106 | *SPHK1* | 3.69E-06 | 4.64 | up |
| A_23_P217845 | *RGS16* | 2.27E-07 | 4.64 | up |
| A_23_P65678 | *FBN1* | 1.26E-07 | 4.63 | up |
| A_24_P192994 | *FADS1* | 5.83E-05 | 4.62 | up |
| A_23_P74609 | *G0S2* | 1.31E-03 | 4.56 | up |
| A_23_P165927 | *STMN3* | 6.60E-03 | 4.54 | up |
| A_23_P58082 | *CCDC80* | 5.61E-06 | 4.52 | up |
| A_23_P1029 | *MFAP2* | 3.73E-07 | 4.50 | up |
| A_24_P131522 | *ANTXR1* | 3.71E-07 | 4.39 | up |
| A_23_P214168 | *COL12A1* | 2.91E-04 | 4.38 | up |
| A_32_P74409 | *AG2* | 3.74E-05 | 4.36 | up |
| A_23_P254507 | *HOPX* | 6.89E-07 | 4.34 | up |
| A_24_P404822 | *APLN* | 3.44E-07 | 4.33 | up |
| A_23_P317620 | *ARL4C* | 2.42E-05 | 4.28 | up |
| A_23_P145916 | *AEBP1* | 1.95E-04 | 4.22 | up |
| A_23_P101407 | *C3* | 4.52E-03 | 4.18 | up |
| A_23_P211631 | *FBLN1* | 1.02E-04 | 4.18 | up |
| A_23_P399078 | *TIMP3* | 1.20E-05 | 4.16 | up |
| A_32_P97169 | *GPC6* | 6.40E-06 | 4.15 | up |
| A_23_P144959 | *VCAN* | 4.61E-06 | 4.13 | up |
| A_23_P132956 | *UCHL1* | 2.49E-04 | 4.09 | up |
| A_23_P64873 | *DCN* | 1.40E-04 | 4.08 | up |
| A_23_P398566 | *NR4A3* | 2.83E-04 | 4.04 | up |
| A_23_P167096 | *VEGFC* | 1.63E-05 | 4.03 | up |
| A_23_P251499 | *PCOLCE* | 5.90E-07 | 4.02 | up |
| A_32_P74942 |  | 1.26E-03 | 4.00 | up |
| A_23_P127584 | *NNMT* | 6.89E-07 | 3.99 | up |
| A_23_P158593 | *COL5A1* | 2.78E-05 | 3.98 | up |
| A_23_P372834 | *AQP1* | 2.71E-03 | 3.96 | up |
| A_23_P113393 | *APLN* | 2.42E-05 | 3.95 | up |
| A_23_P433016 | *FBLN1* | 9.13E-05 | 3.82 | up |
| A_32_P47754 | *SLC2A14* | 4.01E-05 | 3.79 | up |
| A_23_P41917 | *HOMER1* | 9.18E-05 | 3.78 | up |
| A_23_P42575 | *CALD1* | 4.82E-05 | 3.75 | up |
| A_23_P20864 | *ANGPTL2* | 1.53E-05 | 3.69 | up |
| A_23_P128663 | *SACS* | 1.33E-05 | 3.65 | up |
| A_23_P87752 | *LEPREL2* | 4.47E-05 | 3.63 | up |
| A_23_P44724 | *CSRP2* | 1.87E-06 | 3.61 | up |
| A_23_P430068 | *PDPN* | 8.99E-05 | 3.61 | up |
| A_23_P153964 | *INHBB* | 2.01E-03 | 3.60 | up |
| A_23_P32444 | *MXRA8* | 1.39E-05 | 3.58 | up |
| A_32_P162520 |  | 2.59E-03 | 3.57 | up |
| A_23_P332399 | *GULP1* | 6.67E-03 | 3.57 | up |
| A_23_P39465 | *BST2* | 8.49E-04 | 3.57 | up |
| A_23_P200728 | *FCGR3A* | 2.31E-04 | 3.57 | up |
| A_23_P19894 | *AQP1* | 5.35E-03 | 3.55 | up |
| A_23_P34744 | *CTSK* | 2.73E-05 | 3.55 | up |
| A_23_P211468 |  | 2.42E-03 | 3.54 | up |
| A_23_P205057 | *PCDH17* | 5.32E-04 | 3.53 | up |
| A_32_P210168 | *C15orf59* | 3.05E-07 | 3.53 | up |
| A_23_P383009 | *IGFBP5* | 1.54E-03 | 3.47 | up |
| A_23_P152305 | *CDH11* | 9.26E-06 | 3.46 | up |
| A_23_P103496 | *GBP4* | 2.54E-03 | 3.46 | up |
| A_23_P38795 | *FPR1* | 3.25E-04 | 3.44 | up |
| A_32_P108254 | *FAM20A* | 6.09E-06 | 3.44 | up |
| A_23_P129695 | *VASN* | 5.83E-05 | 3.43 | up |
| A_23_P45871 | *IFI44L* | 2.37E-03 | 3.43 | up |
| A_23_P25974 | *TTC7B* | 4.48E-04 | 3.41 | up |
| A_23_P102000 | *CXCR4* | 2.50E-04 | 3.38 | up |
| A_24_P109214 | *APOC1* | 1.45E-03 | 3.36 | up |
| A_23_P24104 | *PLAU* | 2.44E-03 | 3.35 | up |
| A_23_P117602 | *GZMB* | 6.10E-03 | 3.34 | up |
| A_23_P97141 | *RGS1* | 1.02E-03 | 3.34 | up |
| A_23_P131614 | *COL6A3* | 4.05E-04 | 3.33 | up |
| A_32_P215938 | *GPSM1* | 6.63E-04 | 3.32 | up |
| A_23_P207564 | *CCL4* | 1.38E-04 | 3.31 | up |
| A_23_P819 | *ISG15* | 2.40E-05 | 3.27 | up |
| A_24_P561165 |  | 7.91E-06 | 3.26 | up |
| A_23_P79482 | *CHN1* | 8.67E-05 | 3.23 | up |
| A_23_P204286 | *MGP* | 2.09E-05 | 3.23 | up |
| A_24_P921366 | *CALD1* | 4.28E-05 | 3.23 | up |
| A_32_P62863 | *SCHIP1* | 2.56E-04 | 3.22 | up |
| A_23_P24414 | *EFEMP2* | 1.23E-05 | 3.20 | up |
| A_23_P4649 | *APOC1* | 1.63E-03 | 3.18 | up |
| A_23_P434809 | *S100A8* | 9.19E-03 | 3.18 | up |
| A_23_P89431 | *CCL2* | 1.07E-03 | 3.18 | up |
| A_23_P217269 | *VSIG4* | 3.99E-04 | 3.18 | up |
| A_23_P127565 | *LAYN* | 7.59E-07 | 3.16 | up |
| A_24_P273378 | *CAMKK2* | 9.71E-03 | 3.15 | up |
| A_23_P74290 | *GBP5* | 1.62E-03 | 3.13 | up |
| A_23_P211212 | *COL18A1* | 4.75E-04 | 3.12 | up |
| A_23_P210690 | *TRIB3* | 3.10E-03 | 3.11 | up |
| A_23_P30614 | *PLN* | 6.24E-03 | 3.10 | up |
| A_23_P428129 | *CDKN1C* | 8.14E-04 | 3.10 | up |
| A_24_P135322 | *NRP1* | 2.38E-05 | 3.09 | up |
| A_23_P501007 | *EFEMP1* | 2.83E-04 | 3.09 | up |
| A_23_P210425 | *MYL9* | 3.95E-05 | 3.08 | up |
| A_24_P228130 | *CCL3L3* | 3.56E-03 | 3.07 | up |
| A_24_P339201 | *METRN* | 5.58E-03 | 3.06 | up |
| A_23_P97990 | *HTRA1* | 8.03E-05 | 3.06 | up |
| A_23_P259071 | *AREG* | 3.77E-03 | 3.05 | up |
| A_23_P124084 | *LOXL1* | 3.23E-04 | 3.03 | up |
| A_23_P373017 | *CCL3* | 3.82E-03 | 3.02 | up |
| A_32_P70158 | *LILRB3* | 9.49E-04 | 3.02 | up |
| A_23_P52266 | *IFIT1* | 1.92E-03 | 3.00 | up |
| A_24_P306896 | *LOC283711* | 2.52E-03 | 2.99 | up |
| A_23_P19663 | *CTGF* | 2.19E-05 | 2.99 | up |
| A_24_P305541 | *TRIB3* | 4.72E-03 | 2.99 | up |
| A_23_P255111 |  | 6.69E-05 | 2.99 | up |
| A_23_P215634 | *IGFBP3* | 8.98E-04 | 2.98 | up |
| A_24_P261417 | *DKK3* | 1.43E-04 | 2.98 | up |
| A_23_P416581 | *GNAZ* | 2.70E-03 | 2.98 | up |
| A_32_P86763 | *TGM2* | 7.40E-03 | 2.97 | up |
| A_23_P217688 | *TSC22D3* | 2.37E-03 | 2.97 | up |
| A_23_P259292 | *C1QTNF5* | 3.48E-05 | 2.97 | up |
| A_23_P139123 | *SERPING1* | 4.47E-05 | 2.95 | up |
| A_32_P171313 | *GNB4* | 1.07E-04 | 2.95 | up |
| A_24_P215352 | *PRKCDBP* | 2.94E-06 | 2.95 | up |
| A_23_P29124 | *38596* | 5.47E-06 | 2.94 | up |
| A_23_P212696 | *FSTL1* | 3.23E-04 | 2.93 | up |
| A_24_P876522 | *GPX8* | 2.00E-04 | 2.91 | up |
| A_23_P162047 | *DKK3* | 2.26E-04 | 2.91 | up |
| A_24_P148261 | *TGFB2* | 1.50E-03 | 2.88 | up |
| A_23_P374104 | *ANGPTL2* | 2.69E-04 | 2.87 | up |
| A_24_P173727 | *PEX10* | 7.00E-03 | 2.87 | up |
| A_24_P931443 | *GPR68* | 8.41E-04 | 2.84 | up |
| A_23_P117782 | *LARP6* | 9.43E-03 | 2.84 | up |
| A_23_P321920 | *CCL3L3* | 6.58E-03 | 2.84 | up |
| A_24_P380734 | *SDC2* | 1.40E-04 | 2.83 | up |
| A_23_P401076 | *SUSD3* | 7.20E-04 | 2.81 | up |
| A_23_P61945 | *MITF* | 1.88E-04 | 2.81 | up |
| A_23_P259692 | *PSAT1* | 1.47E-03 | 2.81 | up |
| A_23_P81898 | *UBD* | 7.71E-03 | 2.81 | up |
| A_23_P164650 | *APOE* | 9.61E-03 | 2.79 | up |
| A_24_P299685 | *PDPN* | 1.72E-03 | 2.79 | up |
| A_23_P12282 | *DYRK3* | 1.38E-04 | 2.79 | up |
| A_23_P60079 | *ANGPT2* | 1.73E-03 | 2.77 | up |
| A_24_P154037 | *IRS2* | 1.63E-03 | 2.76 | up |
| A_23_P36364 | *THY1* | 5.50E-03 | 2.74 | up |
| A_23_P112470 | *CCL21* | 5.32E-05 | 2.74 | up |
| A_24_P300777 | *ADAM8* | 8.67E-05 | 2.74 | up |
| A_23_P431410 | *RBMS1* | 4.42E-04 | 2.74 | up |
| A_23_P87013 | *TAGLN* | 4.43E-05 | 2.74 | up |
| A_23_P360964 | *DACT3* | 1.11E-04 | 2.73 | up |
| A_24_P359856 | *HDAC4* | 3.32E-05 | 2.73 | up |
| A_23_P153320 | *ICAM1* | 4.42E-04 | 2.71 | up |
| A_23_P203475 | *PRKCDBP* | 1.05E-03 | 2.71 | up |
| A_23_P571 | *SLC2A1* | 3.54E-04 | 2.71 | up |
| A_23_P85716 | *FCGR2A* | 2.21E-04 | 2.71 | up |
| A_23_P157879 | *FCN1* | 4.72E-03 | 2.70 | up |
| A_23_P134426 | *GPNMB* | 1.07E-03 | 2.69 | up |
| A_23_P161727 | *HSPB2* | 7.36E-05 | 2.68 | up |
| A_23_P87011 | *TAGLN* | 1.12E-04 | 2.68 | up |
| A_23_P104252 | *ITIH5* | 2.88E-03 | 2.67 | up |
| A_23_P128974 | *BATF* | 6.01E-03 | 2.67 | up |
| A_23_P409417 | *VPS37D* | 8.36E-05 | 2.66 | up |
| A_24_P57426 | *COL18A1* | 4.68E-04 | 2.65 | up |
| A_24_P82106 | *MMP14* | 2.19E-04 | 2.64 | up |
| A_23_P100660 | *SERPINF1* | 1.14E-04 | 2.62 | up |
| A_23_P43276 | *GPR124* | 2.75E-04 | 2.62 | up |
| A_24_P320699 | *IGFBP3* | 1.92E-03 | 2.61 | up |
| A_23_P146284 | *SQLE* | 1.42E-03 | 2.61 | up |
| A_24_P326660 | *MCAM* | 6.86E-03 | 2.60 | up |
| A_24_P297539 | *UBE2C* | 3.80E-04 | 2.60 | up |
| A_23_P152876 | *RAB34* | 2.48E-04 | 2.60 | up |
| A_23_P2492 | *C1S* | 3.38E-04 | 2.59 | up |
| A_23_P168306 | *WASF1* | 6.50E-04 | 2.59 | up |
| A_23_P86390 | *NRP1* | 2.70E-03 | 2.59 | up |
| A_23_P209978 | *VSNL1* | 4.93E-03 | 2.59 | up |
| A_23_P99642 | *SLC7A7* | 1.70E-04 | 2.58 | up |
| A_24_P319923 | *MYLK* | 1.12E-03 | 2.58 | up |
| A_24_P478940 |  | 4.74E-03 | 2.57 | up |
| A_23_P111995 | *LOXL2* | 8.50E-03 | 2.56 | up |
| A_23_P109143 | *PRNP* | 1.17E-03 | 2.56 | up |
| A_23_P80040 | *PROCR* | 1.14E-03 | 2.55 | up |
| A_32_P49284 |  | 4.55E-03 | 2.54 | up |
| A_24_P315256 |  | 7.90E-04 | 2.53 | up |
| A_23_P256158 | *ADRA2C* | 4.17E-03 | 2.52 | up |
| A_23_P20566 | *TPM2* | 1.04E-04 | 2.52 | up |
| A_23_P315571 | *RFTN1* | 4.75E-04 | 2.52 | up |
| A_23_P114740 | *CFH* | 1.72E-03 | 2.52 | up |
| A_23_P404259 | *GPX8* | 5.31E-03 | 2.51 | up |
| A_23_P361014 | *TSHZ3* | 3.18E-03 | 2.51 | up |
| A_23_P204958 | *LATS2* | 4.97E-05 | 2.50 | up |
| A_23_P8513 | *SNX10* | 1.31E-03 | 2.50 | up |
| A_23_P60856 | *TSPAN4* | 3.61E-05 | 2.50 | up |
| A_23_P96568 | *FLNA* | 1.09E-03 | 2.50 | up |
| A_23_P72651 | *ECSCR* | 9.09E-04 | 2.49 | up |
| A_24_P169688 | *MICB* | 4.28E-03 | 2.48 | up |
| A_23_P75260 | *RASSF4* | 1.39E-03 | 2.48 | up |
| A_23_P91334 | *HSPA12B* | 1.02E-03 | 2.46 | up |
| A_23_P145694 | *ASNS* | 6.60E-03 | 2.45 | up |
| A_24_P145911 | *TRA2B* | 2.83E-04 | 2.45 | up |
| A_23_P257043 | *GEM* | 3.67E-03 | 2.45 | up |
| A_23_P36496 | *RBMS1* | 2.54E-03 | 2.45 | up |
| A_32_P198412 |  | 1.31E-03 | 2.45 | up |
| A_23_P394064 | *PTRF* | 1.51E-04 | 2.45 | up |
| A_23_P256205 | *ABLIM3* | 3.25E-04 | 2.44 | up |
| A_23_P119353 | *RASIP1* | 2.45E-04 | 2.44 | up |
| A_23_P111701 | *GNG11* | 8.16E-04 | 2.43 | up |
| A_24_P686965 | *SH2D5* | 4.76E-03 | 2.43 | up |
| A_23_P310956 | *COL6A2* | 2.27E-03 | 2.43 | up |
| A_23_P3963 | *CDR2L* | 1.66E-04 | 2.42 | up |
| A_32_P143824 |  | 5.02E-05 | 2.42 | up |
| A_23_P114903 | *HSPA6* | 4.37E-05 | 2.42 | up |
| A_32_P72447 | *UBE2S* | 1.08E-05 | 2.41 | up |
| A_23_P3911 | *PLXDC1* | 9.57E-03 | 2.41 | up |
| A_24_P98109 | *SNX10* | 7.72E-04 | 2.40 | up |
| A_23_P48198 | *GLT8D2* | 1.64E-03 | 2.40 | up |
| A_23_P52336 | *UNC5B* | 7.79E-03 | 2.40 | up |
| A_23_P92499 | *TLR2* | 2.16E-03 | 2.39 | up |
| A_23_P144244 |  | 1.44E-03 | 2.39 | up |
| A_24_P918317 | *DKK3* | 4.31E-03 | 2.39 | up |
| A_23_P353035 | *IGFBP7* | 1.38E-04 | 2.39 | up |
| A_24_P298179 |  | 6.90E-03 | 2.38 | up |
| A_24_P925062 | *MXRA7* | 3.52E-04 | 2.38 | up |
| A_23_P131866 | *AURKA* | 1.12E-04 | 2.38 | up |
| A_24_P401051 |  | 8.28E-04 | 2.37 | up |
| A_23_P138885 | *FIBIN* | 7.62E-05 | 2.37 | up |
| A_24_P328668 | *ARL13B* | 3.94E-03 | 2.36 | up |
| A_23_P125423 | *C1R* | 1.27E-03 | 2.36 | up |
| A_23_P212159 | *NUP210* | 6.25E-03 | 2.35 | up |
| A_23_P92727 | *RAI14* | 2.38E-05 | 2.35 | up |
| A_23_P6771 | *LMCD1* | 4.81E-04 | 2.35 | up |
| A_24_P920207 | *ARHGAP23* | 2.88E-03 | 2.35 | up |
| A_23_P161501 | *UBTD1* | 8.22E-06 | 2.34 | up |
| A_23_P100711 | *PMP22* | 3.03E-04 | 2.34 | up |
| A_23_P344421 | *ROBO4* | 2.50E-03 | 2.34 | up |
| A_23_P112241 | *DNAJB5* | 6.32E-04 | 2.33 | up |
| A_23_P143817 | *MYLK* | 1.63E-03 | 2.33 | up |
| A_23_P76480 |  | 9.49E-04 | 2.33 | up |
| A_23_P58588 | *SLIT3* | 2.55E-03 | 2.32 | up |
| A_23_P128728 | *ARG2* | 7.24E-03 | 2.32 | up |
| A_24_P195454 |  | 2.85E-04 | 2.31 | up |
| A_23_P91390 | *THBD* | 8.86E-03 | 2.31 | up |
| A_23_P161190 | *VIM* | 7.23E-04 | 2.30 | up |
| A_23_P502142 | *FYN* | 6.82E-04 | 2.30 | up |
| A_23_P50389 | *NAT14* | 1.08E-03 | 2.30 | up |
| A_32_P32254 | *COL6A1* | 4.68E-03 | 2.29 | up |
| A_32_P16204 | *LOC375295* | 7.14E-04 | 2.29 | up |
| A_32_P184933 | *UBE2S* | 4.22E-05 | 2.27 | up |
| A_23_P166459 | *LGALS1* | 2.84E-03 | 2.27 | up |
| A_24_P16892 | *TAF2* | 3.74E-03 | 2.27 | up |
| A_23_P74088 | *MMP23B* | 6.30E-04 | 2.26 | up |
| A_32_P32739 | *NAGS* | 1.56E-03 | 2.26 | up |
| A_23_P103256 | *CFHR3* | 3.63E-04 | 2.25 | up |
| A_23_P388146 |  | 5.28E-05 | 2.25 | up |
| A_24_P298174 | *CBX1* | 7.24E-03 | 2.24 | up |
| A_32_P81334 | *LARP4* | 3.35E-03 | 2.24 | up |
| A_24_P267452 | *CD3EAP* | 3.14E-03 | 2.22 | up |
| A_23_P200160 | *CFH* | 6.86E-03 | 2.22 | up |
| A_32_P151800 | *FAM72D* | 1.92E-03 | 2.21 | up |
| A_32_P119348 | *BICC1* | 1.99E-03 | 2.20 | up |
| A_23_P21134 | *DDIT3* | 1.86E-04 | 2.20 | up |
| A_23_P86653 | *SRGN* | 1.94E-03 | 2.19 | up |
| A_23_P101093 | *COPZ2* | 8.38E-04 | 2.19 | up |
| A_23_P110403 | *PDLIM3* | 6.19E-03 | 2.18 | up |
| A_23_P88865 | *CMTM3* | 1.03E-03 | 2.18 | up |
| A_23_P21162 | *TCTEX1D2* | 5.22E-03 | 2.18 | up |
| A_23_P63618 | *SCD* | 1.44E-03 | 2.18 | up |
| A_24_P315306 | *ITPRIPL2* | 1.07E-03 | 2.18 | up |
| A_23_P374082 | *ADAM19* | 3.58E-03 | 2.18 | up |
| A_23_P14174 | *TNFSF13B* | 1.91E-04 | 2.17 | up |
| A_23_P308800 | *GLS* | 2.42E-05 | 2.17 | up |
| A_23_P208991 | *PALM* | 2.73E-03 | 2.17 | up |
| A_32_P514599 |  | 3.58E-03 | 2.17 | up |
| A_24_P13390 | *RNFT2* | 3.42E-03 | 2.17 | up |
| A_23_P143190 | *MYBL2* | 2.58E-03 | 2.16 | up |
| A_23_P340909 | *SKA3* | 3.87E-04 | 2.16 | up |
| A_23_P150394 | *FXYD6* | 8.99E-05 | 2.16 | up |
| A_24_P347378 | *ALOX5AP* | 1.50E-03 | 2.16 | up |
| A_23_P61987 | *TMEM121* | 2.37E-04 | 2.16 | up |
| A_24_P794122 |  | 2.82E-03 | 2.15 | up |
| A_23_P204158 | *RNFT2* | 5.45E-03 | 2.15 | up |
| A_23_P17430 | *RBM38* | 3.48E-03 | 2.15 | up |
| A_23_P502274 | *MAPK11* | 1.70E-04 | 2.14 | up |
| A_32_P164522 | *SKA2* | 1.40E-04 | 2.14 | up |
| A_32_P540991 | *DGKE* | 4.45E-05 | 2.14 | up |
| A_23_P254271 | *TUBB6* | 4.57E-04 | 2.14 | up |
| A_24_P355267 | *SLC25A25* | 7.55E-03 | 2.14 | up |
| A_23_P160934 | *ANP32E* | 4.31E-05 | 2.14 | up |
| A_24_P68631 | *HIST2H2AB* | 8.10E-03 | 2.13 | up |
| A_23_P153489 | *CLEC11A* | 1.91E-03 | 2.12 | up |
| A_23_P204810 | *OSBPL8* | 2.85E-03 | 2.12 | up |
| A_23_P51926 | *PTAFR* | 4.28E-03 | 2.12 | up |
| A_24_P919850 | *BDKRB1* | 3.49E-03 | 2.12 | up |
| A_24_P133584 | *MFGE8* | 3.47E-03 | 2.12 | up |
| A_24_P154948 | *GARS* | 4.89E-03 | 2.12 | up |
| A_24_P819729 |  | 8.66E-03 | 2.12 | up |
| A_24_P945396 | *SF3B3* | 2.37E-04 | 2.12 | up |
| A_23_P406187 | *NAGS* | 6.06E-03 | 2.11 | up |
| A_24_P380022 | *EIF5A2* | 2.66E-04 | 2.11 | up |
| A_23_P50368 | *OSCAR* | 1.79E-03 | 2.11 | up |
| A_23_P411993 | *ITIH5* | 2.69E-04 | 2.11 | up |
| A_24_P912985 |  | 2.50E-04 | 2.11 | up |
| A_24_P370372 | *CBX6* | 6.56E-05 | 2.10 | up |
| A_23_P29939 | *SNCA* | 1.02E-03 | 2.10 | up |
| A_23_P114947 | *RGS2* | 8.50E-03 | 2.10 | up |
| A_23_P76992 | *PGF* | 4.77E-03 | 2.10 | up |
| A_23_P161194 | *VIM* | 4.13E-03 | 2.09 | up |
| A_32_P9382 | *C13orf37* | 1.76E-05 | 2.09 | up |
| A_24_P206776 | *CRYAB* | 1.96E-04 | 2.09 | up |
| A_23_P200829 | *SRGAP2* | 4.08E-03 | 2.09 | up |
| A_23_P128613 | *KDELC1* | 9.04E-05 | 2.08 | up |
| A_24_P153853 | *TRIM37* | 4.76E-05 | 2.08 | up |
| A_23_P301079 | *SKA2* | 4.28E-04 | 2.08 | up |
| A_23_P62115 | *TIMP1* | 4.62E-03 | 2.08 | up |
| A_23_P49972 | *CDC6* | 2.44E-04 | 2.07 | up |
| A_23_P54055 | *JUB* | 2.75E-03 | 2.07 | up |
| A_24_P201702 | *CLEC2B* | 6.97E-03 | 2.07 | up |
| A_24_P156113 | *EHD2* | 8.89E-03 | 2.06 | up |
| A_24_P324314 | *MXRA7* | 6.69E-05 | 2.05 | up |
| A_23_P218523 | *C19orf28* | 2.91E-04 | 2.05 | up |
| A_23_P128744 | *BDKRB1* | 5.12E-03 | 2.05 | up |
| A_23_P35114 | *PLEKHO1* | 6.71E-04 | 2.05 | up |
| A_23_P141362 | *FZD2* | 1.75E-03 | 2.05 | up |
| A_32_P184937 |  | 4.00E-04 | 2.04 | up |
| A_23_P164258 | *PIPOX* | 3.83E-03 | 2.03 | up |
| A_23_P113351 | *SPARCL1* | 4.95E-03 | 2.03 | up |
| A_23_P138194 | *NCF2* | 2.29E-03 | 2.03 | up |
| A_23_P27332 | *TCF4* | 8.76E-03 | 2.02 | up |
| A_24_P756657 | *C6orf225* | 9.76E-03 | 2.01 | up |
| A_23_P202881 | *FEZ1* | 3.67E-03 | 2.01 | up |
| A_23_P411296 | *CEBPB* | 2.00E-03 | 2.01 | up |
| A_23_P111737 | *RAMP3* | 3.91E-03 | 2.00 | up |
| A_24_P240166 | *PHLDB2* | 8.12E-03 | 2.00 | up |
| A_23_P362046 | *C13orf27* | 2.42E-05 | 2.00 | up |
| A_23_P368711 | *LILRB3* | 4.58E-03 | 2.00 | up |
| A_23_P19226 | *DSE* | 5.92E-03 | 2.00 | up |
