# Supplementary material for: A Gene Expression and Pre-mRNA Splicing Signature That Marks the Adenoma-Adenocarcinoma Progression in Colorectal Cancer
Source: PLoS One. 2014 Feb 6;9(2):e87761. doi: 10.1371/journal.pone.0087761 (PMC3916340; doi:10.1371/journal.pone.0087761)
Supplement: Table S9 — List of the up- and down-regulated genes of the gene expression signature of 172 probes. (DOC) [file pone.0087761.s015.doc]

**Table S9. List of the up- and down-regulated genes of the gene expression signature of 172 probes.** Signature of 172 probes corresponding to genes deregulated in CRC in comparison to both CRA and NOR (≥ 2.0 FC, P-value ≤ 0.01 by *t*-test with FDR).

|  |  | Colorectal Cancer *vs*. Normal | | | Colorectal Cancer *vs*. Colorectal Adenoma | | |
| --- | --- | --- | --- | --- | --- | --- | --- |
| Probe Name | Gene Symbol | P-value | Fold-Change | Regulation | P-value | Fold-Change | Regulation |
| A_23_P51217 | *CLCA1* | 3.51E-03 | 64.56 | down | 1.81E-06 | 41.59 | down |
| A_23_P36018 | *VSIG2* | 1.54E-03 | 19.80 | down | 1.16E-07 | 12.09 | down |
| A_23_P320216 | *FAM55D* | 3.65E-03 | 17.45 | down | 2.00E-05 | 7.76 | down |
| A_23_P156826 | *C6orf105* | 1.86E-03 | 18.54 | down | 9.22E-05 | 6.66 | down |
| A_23_P61042 | *IGHA2* | 3.43E-03 | 21.85 | down | 7.54E-03 | 5.80 | down |
| A_23_P31798 | *NAT2* | 1.01E-03 | 5.82 | down | 4.61E-08 | 5.71 | down |
| A_23_P257993 | *DNASE1L3* | 1.94E-03 | 12.77 | down | 8.39E-06 | 5.34 | down |
| A_23_P24543 | *FAM55A* | 8.19E-03 | 9.13 | down | 1.42E-05 | 5.24 | down |
| A_23_P86021 | *SELENBP1* | 4.50E-03 | 6.00 | down | 2.87E-07 | 5.15 | down |
| A_23_P74619 | *SELENBP1* | 4.69E-03 | 6.60 | down | 8.45E-05 | 4.84 | down |
| A_32_P180336 | *C11orf92* | 8.54E-03 | 2.59 | down | 5.92E-05 | 4.77 | down |
| A_23_P16225 | *BEST2* | 1.11E-03 | 20.25 | down | 9.80E-03 | 4.76 | down |
| A_23_P60599 | *UGT1A6* | 3.46E-03 | 10.89 | down | 1.43E-04 | 4.67 | down |
| A_23_P8640 | *GPER* | 6.08E-03 | 6.69 | down | 2.50E-04 | 4.44 | down |
| A_23_P233 | *FMO5* | 2.92E-03 | 4.73 | down | 1.06E-05 | 4.02 | down |
| A_24_P220947 | *AKR1C1* | 7.64E-04 | 6.64 | down | 2.37E-04 | 3.95 | down |
| A_23_P203698 | *MOGAT2* | 2.54E-03 | 6.40 | down | 1.57E-04 | 3.69 | down |
| A_23_P26854 | *RICH2* | 1.74E-03 | 7.65 | down | 7.88E-04 | 3.68 | down |
| A_32_P14737 |  | 2.11E-03 | 3.28 | down | 6.04E-06 | 3.68 | down |
| A_23_P218247 | *CES3* | 2.85E-03 | 5.58 | down | 8.80E-05 | 3.64 | down |
| A_23_P167168 | *IGJ* | 3.42E-03 | 8.87 | down | 2.42E-03 | 3.55 | down |
| A_23_P214281 | *PAQR8* | 8.51E-04 | 4.14 | down | 1.19E-07 | 3.53 | down |
| A_23_P17420 | *BCAS1* | 1.82E-03 | 6.87 | down | 2.26E-05 | 3.50 | down |
| A_24_P169873 | *IGHA2* | 9.02E-03 | 6.82 | down | 2.41E-03 | 3.41 | down |
| A_23_P135123 |  | 7.50E-03 | 3.92 | down | 1.66E-04 | 3.32 | down |
| A_23_P54770 | *APOB48R* | 2.25E-03 | 5.86 | down | 3.38E-06 | 3.23 | down |
| A_23_P94103 | *SCARA5* | 1.11E-03 | 36.56 | down | 1.27E-03 | 3.17 | down |
| A_32_P217140 | *ISX* | 4.80E-03 | 8.32 | down | 2.19E-03 | 3.14 | down |
| A_23_P259594 | *AKAP7* | 4.79E-03 | 2.97 | down | 7.25E-04 | 3.05 | down |
| A_23_P257583 | *DENND2A* | 2.00E-03 | 4.35 | down | 3.12E-04 | 3.04 | down |
| A_24_P334640 | *PAQR8* | 6.65E-04 | 2.99 | down | 4.61E-07 | 3.03 | down |
| A_24_P648880 | *MEIS3P1* | 1.48E-03 | 4.49 | down | 8.03E-05 | 3.03 | down |
| A_23_P392470 | *NR3C2* | 6.39E-03 | 7.53 | down | 3.79E-04 | 2.92 | down |
| A_23_P11629 | *TMEM61* | 9.72E-03 | 4.31 | down | 8.69E-03 | 2.91 | down |
| A_24_P379820 | *ITM2C* | 2.84E-03 | 5.85 | down | 1.14E-03 | 2.87 | down |
| A_24_P156490 | *KCNMA1* | 9.10E-03 | 4.56 | down | 1.14E-03 | 2.85 | down |
| A_24_P218814 | *RDH5* | 8.51E-04 | 6.34 | down | 7.18E-04 | 2.85 | down |
| A_23_P21485 | *PID1* | 7.84E-03 | 3.96 | down | 2.19E-04 | 2.77 | down |
| A_23_P8834 | *EPHX2* | 7.01E-03 | 3.83 | down | 4.36E-05 | 2.77 | down |
| A_24_P71341 | *FMO5* | 3.17E-03 | 3.45 | down | 2.62E-06 | 2.76 | down |
| A_24_P62708 | *PRKACB* | 9.25E-03 | 4.50 | down | 6.40E-04 | 2.75 | down |
| A_24_P174503 | *AMT* | 5.34E-03 | 3.33 | down | 2.46E-03 | 2.75 | down |
| A_23_P257164 | *AMT* | 6.18E-03 | 3.63 | down | 3.00E-03 | 2.74 | down |
| A_32_P37592 | *SCARNA17* | 5.86E-03 | 3.93 | down | 8.06E-03 | 2.73 | down |
| A_24_P402690 | *ITM2C* | 5.51E-03 | 5.62 | down | 3.14E-04 | 2.70 | down |
| A_23_P14986 | *HSD11B2* | 1.16E-03 | 7.07 | down | 6.38E-05 | 2.68 | down |
| A_23_P501831 | *C5orf4* | 8.99E-03 | 2.65 | down | 1.40E-04 | 2.66 | down |
| A_23_P364625 | *LRRC19* | 6.01E-03 | 9.42 | down | 3.52E-03 | 2.65 | down |
| A_23_P95594 | *NAT1* | 3.79E-03 | 3.38 | down | 2.61E-05 | 2.64 | down |
| A_32_P49854 |  | 9.35E-03 | 2.34 | down | 1.47E-04 | 2.58 | down |
| A_24_P782308 | *NEDD4L* | 4.88E-03 | 2.90 | down | 3.54E-04 | 2.48 | down |
| A_23_P66767 | *GGT6* | 6.06E-03 | 4.54 | down | 6.19E-03 | 2.45 | down |
| A_23_P99249 | *SUCLG2* | 2.80E-03 | 2.15 | down | 1.39E-04 | 2.41 | down |
| A_24_P112395 | *PBLD* | 7.65E-03 | 5.31 | down | 3.69E-03 | 2.40 | down |
| A_24_P945113 | *ACVRL1* | 5.43E-03 | 3.19 | down | 1.47E-03 | 2.38 | down |
| A_23_P149998 | *PBLD* | 9.07E-03 | 5.58 | down | 5.90E-03 | 2.38 | down |
| A_24_P209171 | *SH3BGRL2* | 3.63E-03 | 2.42 | down | 3.78E-06 | 2.36 | down |
| A_23_P129157 | *NEIL1* | 1.47E-03 | 2.19 | down | 1.69E-03 | 2.34 | down |
| A_24_P90881 | *CES3* | 9.95E-04 | 4.55 | down | 9.25E-04 | 2.32 | down |
| A_23_P72117 | *SMPDL3A* | 1.90E-03 | 7.07 | down | 2.30E-03 | 2.29 | down |
| A_24_P105933 | *VIPR1* | 3.13E-03 | 3.93 | down | 3.54E-04 | 2.29 | down |
| A_24_P623734 | *C2orf72* | 8.54E-03 | 3.24 | down | 5.64E-03 | 2.28 | down |
| A_23_P48771 | *C14orf159* | 5.84E-03 | 2.34 | down | 1.68E-05 | 2.26 | down |
| A_23_P129312 | *PPP1R14D* | 9.58E-03 | 3.59 | down | 2.42E-03 | 2.24 | down |
| A_23_P7882 | *SLC22A23* | 3.02E-03 | 3.50 | down | 2.83E-04 | 2.23 | down |
| A_23_P143935 | *PIGZ* | 4.68E-03 | 4.01 | down | 4.40E-03 | 2.20 | down |
| A_32_P166733 |  | 5.72E-03 | 2.25 | down | 1.83E-05 | 2.18 | down |
| A_23_P160433 | *C1orf115* | 4.35E-03 | 4.83 | down | 2.85E-03 | 2.16 | down |
| A_23_P53193 | *SYTL2* | 1.16E-03 | 3.04 | down | 3.22E-04 | 2.16 | down |
| A_23_P411612 | *SPRYD4* | 4.28E-03 | 2.40 | down | 4.42E-06 | 2.15 | down |
| A_24_P319374 | *GPA33* | 8.41E-03 | 3.28 | down | 6.46E-04 | 2.15 | down |
| A_24_P131173 | *C1orf115* | 6.01E-03 | 4.46 | down | 3.10E-03 | 2.15 | down |
| A_23_P158880 | *STARD5* | 1.00E-02 | 2.31 | down | 2.27E-04 | 2.15 | down |
| A_23_P121926 | *SEPP1* | 2.45E-03 | 6.27 | down | 6.02E-03 | 2.13 | down |
| A_23_P114689 | *ASAP3* | 7.64E-04 | 5.03 | down | 2.44E-03 | 2.13 | down |
| A_32_P156564 |  | 5.02E-03 | 2.74 | down | 5.83E-05 | 2.12 | down |
| A_32_P159289 |  | 8.87E-03 | 2.85 | down | 5.74E-03 | 2.12 | down |
| A_23_P163227 | *CKMT1A* | 8.57E-03 | 3.00 | down | 3.18E-03 | 2.09 | down |
| A_24_P108311 | *NEDD4L* | 7.75E-03 | 3.14 | down | 1.88E-03 | 2.08 | down |
| A_23_P59452 | *ABP1* | 2.35E-03 | 4.02 | down | 1.93E-03 | 2.08 | down |
| A_23_P26815 | *RILP* | 8.51E-04 | 3.04 | down | 5.33E-04 | 2.08 | down |
| A_23_P37205 | *NDRG2* | 6.78E-03 | 3.56 | down | 2.91E-03 | 2.07 | down |
| A_32_P515920 | *LOC400573* | 1.24E-03 | 5.17 | down | 3.48E-03 | 2.06 | down |
| A_23_P398172 | *FAM135A* | 2.98E-03 | 2.06 | down | 1.14E-04 | 2.03 | down |
| A_23_P29975 | *C4orf19* | 1.85E-03 | 2.56 | down | 4.58E-05 | 2.02 | down |
| A_24_P930741 | *EPHA10* | 6.64E-03 | 3.11 | down | 2.16E-03 | 2.02 | down |
| A_23_P207507 | *ABCC3* | 6.63E-03 | 2.40 | down | 1.02E-03 | 2.02 | down |
| A_24_P102343 | *EPN3* | 5.19E-03 | 2.05 | down | 4.28E-05 | 2.01 | down |
| A_23_P387184 | *NHSL1* | 3.28E-03 | 2.59 | down | 3.69E-03 | 2.01 | down |
| A_23_P200741 | *DPT* | 6.92E-03 | 5.36 | down | 3.56E-07 | 5.09 | up |
| A_23_P58082 | *CCDC80* | 5.62E-03 | 3.35 | down | 5.61E-06 | 4.52 | up |
| A_23_P211631 | *FBLN1* | 7.13E-03 | 3.20 | down | 1.02E-04 | 4.18 | up |
| A_23_P433016 | *FBLN1* | 5.53E-03 | 3.40 | down | 9.13E-05 | 3.82 | up |
| A_23_P217269 | *VSIG4* | 2.61E-03 | 2.65 | down | 3.99E-04 | 3.18 | up |
| A_23_P30614 | *PLN* | 3.37E-03 | 2.61 | down | 6.24E-03 | 3.10 | up |
| A_23_P360964 | *DACT3* | 9.46E-03 | 2.30 | down | 1.11E-04 | 2.73 | up |
| A_23_P58588 | *SLIT3* | 8.11E-04 | 5.19 | down | 2.55E-03 | 2.32 | up |
| A_23_P103256 | *CFHR3* | 5.95E-03 | 4.02 | down | 3.63E-04 | 2.25 | up |
| A_23_P200160 | *CFH* | 3.43E-03 | 3.90 | down | 6.86E-03 | 2.22 | up |
| A_23_P411993 | *ITIH5* | 6.28E-03 | 2.40 | down | 2.69E-04 | 2.11 | up |
| A_24_P206776 | *CRYAB* | 2.93E-03 | 2.72 | down | 1.96E-04 | 2.09 | up |
| A_23_P113351 | *SPARCL1* | 6.48E-03 | 3.01 | down | 4.95E-03 | 2.03 | up |
| A_23_P56746 | *FAP* | 2.05E-03 | 21.71 | up | 2.06E-11 | 29.21 | up |
| A_23_P215328 | *SFRP4* | 1.84E-03 | 11.39 | up | 1.74E-10 | 22.97 | up |
| A_23_P62021 | *THBS2* | 3.60E-03 | 7.19 | up | 4.36E-09 | 13.59 | up |
| A_23_P7313 | *SPP1* | 8.29E-03 | 13.96 | up | 1.13E-04 | 11.05 | up |
| A_23_P69030 | *COL8A1* | 2.25E-03 | 6.18 | up | 9.04E-07 | 10.64 | up |
| A_23_P207520 | *COL1A1* | 2.37E-03 | 10.65 | up | 2.87E-07 | 10.26 | up |
| A_23_P111888 | *CTHRC1* | 1.66E-03 | 11.70 | up | 6.10E-07 | 10.14 | up |
| A_23_P43164 | *SULF1* | 4.67E-03 | 6.88 | up | 8.86E-08 | 9.14 | up |
| A_23_P57417 | *MMP11* | 6.01E-03 | 13.37 | up | 1.22E-07 | 8.14 | up |
| A_23_P122924 | *INHBA* | 6.54E-04 | 41.06 | up | 1.22E-05 | 7.18 | up |
| A_23_P166408 | *OSM* | 3.52E-03 | 11.83 | up | 3.29E-04 | 6.25 | up |
| A_23_P320578 | *RGS16* | 3.57E-03 | 4.46 | up | 2.79E-07 | 5.80 | up |
| A_23_P121533 | *SPON2* | 7.93E-03 | 3.48 | up | 7.57E-07 | 5.76 | up |
| A_23_P107963 | *FUT1* | 8.51E-04 | 7.22 | up | 3.23E-05 | 5.64 | up |
| A_23_P203419 | *FADS1* | 2.08E-03 | 4.14 | up | 3.48E-05 | 5.28 | up |
| A_23_P85783 | *PHGDH* | 5.42E-03 | 4.46 | up | 2.94E-03 | 5.25 | up |
| A_32_P116556 | *ZNF469* | 9.66E-03 | 3.74 | up | 3.57E-06 | 5.24 | up |
| A_23_P7642 | *SPARC* | 6.88E-03 | 3.34 | up | 1.33E-06 | 5.12 | up |
| A_23_P131846 | *SNAI1* | 3.38E-03 | 4.40 | up | 2.62E-06 | 4.69 | up |
| A_23_P38106 | *SPHK1* | 9.59E-03 | 4.70 | up | 3.69E-06 | 4.64 | up |
| A_23_P217845 | *RGS16* | 3.62E-03 | 3.89 | up | 2.27E-07 | 4.64 | up |
| A_24_P192994 | *FADS1* | 2.57E-03 | 4.37 | up | 5.83E-05 | 4.62 | up |
| A_23_P1029 | *MFAP2* | 6.25E-03 | 4.94 | up | 3.73E-07 | 4.50 | up |
| A_23_P214168 | *COL12A1* | 6.47E-03 | 5.17 | up | 2.91E-04 | 4.38 | up |
| A_24_P404822 | *APLN* | 6.54E-04 | 6.27 | up | 3.44E-07 | 4.33 | up |
| A_23_P113393 | *APLN* | 8.51E-04 | 8.33 | up | 2.42E-05 | 3.95 | up |
| A_23_P41917 | *HOMER1* | 2.45E-03 | 4.86 | up | 9.18E-05 | 3.78 | up |
| A_23_P430068 | *PDPN* | 2.69E-03 | 4.85 | up | 8.99E-05 | 3.61 | up |
| A_23_P117602 | *GZMB* | 6.62E-03 | 4.72 | up | 6.10E-03 | 3.34 | up |
| A_23_P210690 | *TRIB3* | 1.45E-03 | 9.88 | up | 3.10E-03 | 3.11 | up |
| A_24_P306896 | *LOC283711* | 4.62E-03 | 5.16 | up | 2.52E-03 | 2.99 | up |
| A_24_P305541 | *TRIB3* | 2.49E-03 | 9.21 | up | 4.72E-03 | 2.99 | up |
| A_23_P259692 | *PSAT1* | 5.72E-03 | 6.95 | up | 1.47E-03 | 2.81 | up |
| A_24_P299685 | *PDPN* | 2.37E-03 | 4.77 | up | 1.72E-03 | 2.79 | up |
| A_23_P60079 | *ANGPT2* | 2.13E-03 | 4.47 | up | 1.73E-03 | 2.77 | up |
| A_24_P359856 | *HDAC4* | 5.00E-03 | 2.79 | up | 3.32E-05 | 2.73 | up |
| A_23_P409417 | *VPS37D* | 1.27E-03 | 2.14 | up | 8.36E-05 | 2.66 | up |
| A_23_P146284 | *SQLE* | 2.74E-03 | 3.65 | up | 1.42E-03 | 2.61 | up |
| A_24_P297539 | *UBE2C* | 4.17E-03 | 5.12 | up | 3.80E-04 | 2.60 | up |
| A_32_P49284 |  | 6.32E-03 | 2.77 | up | 4.55E-03 | 2.54 | up |
| A_23_P145694 | *ASNS* | 7.89E-03 | 2.56 | up | 6.60E-03 | 2.45 | up |
| A_23_P3963 | *CDR2L* | 3.22E-03 | 3.18 | up | 1.66E-04 | 2.42 | up |
| A_32_P72447 | *UBE2S* | 1.38E-03 | 4.65 | up | 1.08E-05 | 2.41 | up |
| A_23_P131866 | *AURKA* | 4.24E-03 | 3.61 | up | 1.12E-04 | 2.38 | up |
| A_23_P6771 | *LMCD1* | 2.87E-03 | 2.58 | up | 4.81E-04 | 2.35 | up |
| A_23_P161501 | *UBTD1* | 3.52E-03 | 2.43 | up | 8.22E-06 | 2.34 | up |
| A_24_P195454 |  | 6.22E-03 | 3.49 | up | 2.85E-04 | 2.31 | up |
| A_23_P50389 | *NAT14* | 3.12E-03 | 2.47 | up | 1.08E-03 | 2.30 | up |
| A_32_P184933 | *UBE2S* | 1.70E-03 | 4.56 | up | 4.22E-05 | 2.27 | up |
| A_23_P388146 |  | 2.92E-03 | 4.08 | up | 5.28E-05 | 2.25 | up |
| A_24_P267452 | *CD3EAP* | 1.16E-03 | 4.16 | up | 3.14E-03 | 2.22 | up |
| A_32_P151800 | *FAM72D* | 7.01E-03 | 2.81 | up | 1.92E-03 | 2.21 | up |
| A_23_P63618 | *SCD* | 1.18E-03 | 4.11 | up | 1.44E-03 | 2.18 | up |
| A_32_P514599 |  | 1.14E-03 | 2.40 | up | 3.58E-03 | 2.17 | up |
| A_24_P13390 | *RNFT2* | 4.49E-03 | 3.42 | up | 3.42E-03 | 2.17 | up |
| A_23_P143190 | *MYBL2* | 8.37E-03 | 5.19 | up | 2.58E-03 | 2.16 | up |
| A_23_P340909 | *SKA3* | 1.86E-03 | 4.65 | up | 3.87E-04 | 2.16 | up |
| A_23_P204158 | *RNFT2* | 5.01E-03 | 3.42 | up | 5.45E-03 | 2.15 | up |
| A_24_P945396 | *SF3B3* | 2.70E-03 | 2.46 | up | 2.37E-04 | 2.12 | up |
| A_24_P380022 | *EIF5A2* | 7.54E-03 | 2.68 | up | 2.66E-04 | 2.11 | up |
| A_32_P9382 | *C13orf37* | 7.05E-04 | 3.24 | up | 1.76E-05 | 2.09 | up |
| A_23_P128613 | *KDELC1* | 1.16E-03 | 2.73 | up | 9.04E-05 | 2.08 | up |
| A_24_P153853 | *TRIM37* | 3.91E-03 | 2.26 | up | 4.76E-05 | 2.08 | up |
| A_23_P62115 | *TIMP1* | 2.25E-03 | 4.39 | up | 4.62E-03 | 2.08 | up |
| A_23_P49972 | *CDC6* | 1.16E-03 | 3.83 | up | 2.44E-04 | 2.07 | up |
| A_23_P54055 | *JUB* | 1.11E-03 | 8.77 | up | 2.75E-03 | 2.07 | up |
| A_23_P218523 | *C19orf28* | 2.38E-03 | 2.68 | up | 2.91E-04 | 2.05 | up |
| A_32_P184937 |  | 5.51E-03 | 2.59 | up | 4.00E-04 | 2.04 | up |
| A_23_P411296 | *CEBPB* | 4.20E-03 | 2.42 | up | 2.00E-03 | 2.01 | up |
| A_23_P362046 | *C13orf27* | 1.16E-03 | 3.07 | up | 2.42E-05 | 2.00 | up |
