# Supplementary material for: A Gene Expression and Pre-mRNA Splicing Signature That Marks the Adenoma-Adenocarcinoma Progression in Colorectal Cancer
Source: PLoS One. 2014 Feb 6;9(2):e87761. doi: 10.1371/journal.pone.0087761 (PMC3916340; doi:10.1371/journal.pone.0087761)
Supplement: Table S10 — List of the up- and down-regulated genes of the gene expression signature of 265 probes. (DOC) [file pone.0087761.s016.doc]

**Table S10. List of the up- and down-regulated genes of the gene expression signature of 265 probes.** Signature of 265 probes, corresponding to genes deregulated in CRC as compared to CRA, which were already abnormally expressed in CRA as compared to NOR (≥ 2.0 FC, P-value ≤ 0.01 by *t*-test with FDR).

|  |  | Colorectal Adenoma *vs*. Normal | | | Colorectal Cancer *vs*. Colorectal Adenoma | | |
| --- | --- | --- | --- | --- | --- | --- | --- |
| Probe Name | Gene Symbol | P-value | Fold-Change | Regulation | P-value | Fold-Change | Regulation |
| A_23_P94103 | *SCARA5* | 1.04E-14 | 11.54 | down | 1.27E-03 | 3.17 | down |
| A_23_P16225 | *BEST2* | 4.59E-03 | 4.25 | down | 9.80E-03 | 4.76 | down |
| A_23_P61042 | *IGHA2* | 7.04E-03 | 3.77 | down | 7.54E-03 | 5.80 | down |
| A_23_P364625 | *LRRC19* | 1.38E-07 | 3.55 | down | 3.52E-03 | 2.65 | down |
| A_23_P72117 | *SMPDL3A* | 1.20E-06 | 3.08 | down | 2.30E-03 | 2.29 | down |
| A_23_P121926 | *SEPP1* | 3.11E-06 | 2.95 | down | 6.02E-03 | 2.13 | down |
| A_32_P154361 |  | 9.19E-04 | 2.80 | down | 3.02E-03 | 3.52 | down |
| A_23_P156826 | *C6orf105* | 3.72E-03 | 2.78 | down | 9.22E-05 | 6.66 | down |
| A_32_P217140 | *ISX* | 3.53E-04 | 2.65 | down | 2.19E-03 | 3.14 | down |
| A_23_P14986 | *HSD11B2* | 2.52E-06 | 2.64 | down | 6.38E-05 | 2.68 | down |
| A_23_P392470 | *NR3C2* | 3.98E-06 | 2.58 | down | 3.79E-04 | 2.92 | down |
| A_32_P515920 | *LOC400573* | 7.71E-06 | 2.50 | down | 3.48E-03 | 2.06 | down |
| A_23_P167168 | *IGJ* | 5.24E-03 | 2.50 | down | 2.42E-03 | 3.55 | down |
| A_23_P18447 | *PPARGC1A* | 1.11E-05 | 2.44 | down | 2.40E-03 | 2.74 | down |
| A_23_P257993 | *DNASE1L3* | 2.08E-04 | 2.39 | down | 8.39E-06 | 5.34 | down |
| A_23_P114689 | *ASAP3* | 5.04E-05 | 2.37 | down | 2.44E-03 | 2.13 | down |
| A_23_P149998 | *PBLD* | 3.95E-05 | 2.35 | down | 5.90E-03 | 2.38 | down |
| A_23_P60599 | *UGT1A6* | 2.65E-03 | 2.33 | down | 1.43E-04 | 4.67 | down |
| A_23_P320216 | *FAM55D* | 1.00E-02 | 2.25 | down | 2.00E-05 | 7.76 | down |
| A_23_P160433 | *C1orf115* | 1.89E-04 | 2.23 | down | 2.85E-03 | 2.16 | down |
| A_24_P218814 | *RDH5* | 2.54E-03 | 2.22 | down | 7.18E-04 | 2.85 | down |
| A_24_P112395 | *PBLD* | 1.11E-04 | 2.21 | down | 3.69E-03 | 2.40 | down |
| A_24_P402690 | *ITM2C* | 5.68E-04 | 2.08 | down | 3.14E-04 | 2.70 | down |
| A_24_P131173 | *C1orf115* | 8.42E-04 | 2.08 | down | 3.10E-03 | 2.15 | down |
| A_24_P379820 | *ITM2C* | 3.47E-03 | 2.04 | down | 1.14E-03 | 2.87 | down |
| A_23_P151851 | *DUOX2* | 7.04E-09 | 27.02 | up | 2.51E-05 | 9.03 | down |
| A_23_P169437 | *LCN2* | 5.92E-09 | 18.89 | up | 2.19E-03 | 4.51 | down |
| A_24_P35905 | *DUOX2* | 2.11E-08 | 15.12 | up | 3.03E-05 | 8.00 | down |
| A_23_P2789 | *OLFM4* | 5.56E-06 | 13.34 | up | 4.43E-05 | 18.67 | down |
| A_23_P83339 | *RNF183* | 5.48E-14 | 12.43 | up | 2.11E-03 | 2.97 | down |
| A_23_P148015 | *AXIN2* | 7.71E-15 | 9.38 | up | 6.85E-05 | 3.61 | down |
| A_23_P159395 | *AXIN2* | 2.38E-12 | 7.09 | up | 2.02E-03 | 2.65 | down |
| A_23_P16915 | *QPCT* | 3.28E-08 | 6.52 | up | 7.18E-04 | 4.16 | down |
| A_23_P4212 | *HOXB13* | 3.66E-03 | 5.77 | up | 2.83E-04 | 5.40 | down |
| A_23_P69326 | *CADPS* | 5.44E-10 | 5.46 | up | 9.09E-04 | 3.72 | down |
| A_24_P71468 | *QPCT* | 2.83E-07 | 5.26 | up | 2.99E-03 | 3.44 | down |
| A_23_P154875 | *BACE2* | 3.58E-13 | 4.64 | up | 1.20E-04 | 2.13 | down |
| A_23_P142878 | *ATOH8* | 7.02E-06 | 4.51 | up | 1.19E-07 | 11.08 | down |
| A_24_P14584 | *BACE2* | 1.50E-12 | 4.50 | up | 1.86E-04 | 2.09 | down |
| A_23_P160920 | *PDZK1IP1* | 7.39E-08 | 4.25 | up | 2.30E-03 | 2.44 | down |
| A_23_P218111 | *SERPINA1* | 2.17E-07 | 4.24 | up | 2.67E-04 | 4.25 | down |
| A_23_P394304 | *PDZK1IP1* | 6.28E-08 | 3.98 | up | 3.67E-03 | 2.21 | down |
| A_24_P181254 | *OLFM4* | 4.23E-05 | 3.98 | up | 3.09E-04 | 5.69 | down |
| A_32_P234184 | *HES5* | 5.54E-04 | 3.92 | up | 2.67E-06 | 10.83 | down |
| A_23_P46390 | *SYTL1* | 4.28E-07 | 3.84 | up | 1.12E-03 | 2.94 | down |
| A_32_P217773 | *SYTL1* | 1.58E-06 | 3.76 | up | 3.49E-03 | 2.71 | down |
| A_32_P205624 | *SHC2* | 3.03E-06 | 3.71 | up | 2.36E-03 | 2.86 | down |
| A_23_P146572 | *NPDC1* | 1.32E-09 | 3.68 | up | 7.77E-06 | 2.77 | down |
| A_23_P373031 | *CACNA1C* | 4.83E-06 | 3.67 | up | 8.79E-03 | 2.32 | down |
| A_32_P25437 | *SLC12A2* | 2.54E-12 | 3.63 | up | 1.86E-04 | 2.19 | down |
| A_24_P331128 | *GNA15* | 1.18E-08 | 3.57 | up | 1.59E-03 | 2.20 | down |
| A_24_P602871 | *SAMD5* | 5.11E-05 | 3.42 | up | 1.16E-03 | 3.95 | down |
| A_23_P10194 | *SEZ6L2* | 3.87E-07 | 3.26 | up | 2.54E-04 | 2.62 | down |
| A_23_P254741 | *SOD3* | 3.29E-04 | 3.09 | up | 1.57E-04 | 4.58 | down |
| A_23_P139600 | *RASAL1* | 6.13E-08 | 2.94 | up | 2.70E-03 | 2.42 | down |
| A_24_P79040 | *CAPN12* | 5.35E-06 | 2.93 | up | 7.90E-04 | 3.72 | down |
| A_23_P45786 | *COL9A2* | 3.80E-05 | 2.93 | up | 2.83E-04 | 3.46 | down |
| A_23_P28898 | *PLCB4* | 3.82E-05 | 2.88 | up | 1.08E-03 | 3.37 | down |
| A_23_P164814 | *C19orf57* | 4.30E-06 | 2.79 | up | 1.05E-05 | 3.76 | down |
| A_23_P59375 | *ID4* | 2.62E-04 | 2.76 | up | 3.78E-06 | 4.67 | down |
| A_23_P65812 | *SMAD6* | 4.14E-08 | 2.63 | up | 1.21E-04 | 2.10 | down |
| A_23_P16409 | *CAPN12* | 1.52E-04 | 2.61 | up | 2.55E-03 | 3.25 | down |
| A_23_P165783 | *MLPH* | 1.50E-05 | 2.58 | up | 1.69E-04 | 3.57 | down |
| A_23_P154400 | *MLPH* | 5.82E-06 | 2.57 | up | 3.32E-03 | 2.41 | down |
| A_23_P500936 | *FOXA2* | 9.39E-06 | 2.54 | up | 2.60E-03 | 2.31 | down |
| A_23_P421011 | *KAZALD1* | 8.24E-06 | 2.40 | up | 8.19E-04 | 2.21 | down |
| A_32_P153781 |  | 2.50E-04 | 2.39 | up | 7.42E-03 | 2.14 | down |
| A_23_P407112 | *SPATA18* | 2.07E-04 | 2.39 | up | 1.75E-03 | 2.62 | down |
| A_23_P304921 | *NOX1* | 1.83E-03 | 2.35 | up | 3.58E-03 | 2.92 | down |
| A_23_P214079 | *SPINK1* | 4.58E-04 | 2.31 | up | 2.64E-03 | 2.62 | down |
| A_23_P165778 | *MLPH* | 8.64E-06 | 2.31 | up | 8.36E-05 | 3.07 | down |
| A_23_P379864 | *ASRGL1* | 2.05E-04 | 2.27 | up | 2.09E-03 | 3.27 | down |
| A_23_P27013 | *HOXB9* | 2.61E-07 | 2.27 | up | 1.10E-03 | 4.20 | down |
| A_23_P50638 | *LRG1* | 3.23E-04 | 2.23 | up | 1.06E-03 | 2.43 | down |
| A_23_P2814 | *SMAD9* | 1.42E-04 | 2.15 | up | 5.95E-06 | 4.39 | down |
| A_23_P1505 | *LRP5* | 1.14E-05 | 2.12 | up | 6.84E-05 | 2.07 | down |
| A_23_P357207 | *MRAP2* | 3.82E-04 | 2.11 | up | 6.62E-04 | 3.36 | down |
| A_23_P53530 | *MTERFD3* | 3.15E-07 | 2.07 | up | 3.20E-09 | 2.93 | down |
| A_24_P83118 | *DUSP18* | 1.64E-06 | 2.06 | up | 3.14E-04 | 2.33 | down |
| A_23_P203391 | *ASRGL1* | 8.27E-04 | 2.05 | up | 3.13E-03 | 2.53 | down |
| A_24_P655849 | *SMAD9* | 1.13E-03 | 2.03 | up | 3.38E-05 | 4.63 | down |
| A_24_P934546 | *SFRP2* | 5.04E-16 | 34.57 | down | 2.60E-09 | 15.08 | up |
| A_23_P200741 | *DPT* | 6.20E-18 | 27.31 | down | 3.56E-07 | 5.09 | up |
| A_24_P137501 | *SFRP2* | 2.27E-11 | 22.56 | down | 9.82E-06 | 8.72 | up |
| A_23_P143981 | *FBLN2* | 3.31E-15 | 17.21 | down | 3.20E-09 | 10.62 | up |
| A_23_P58082 | *CCDC80* | 7.18E-14 | 15.11 | down | 5.61E-06 | 4.52 | up |
| A_23_P211631 | *FBLN1* | 1.36E-11 | 13.36 | down | 1.02E-04 | 4.18 | up |
| A_23_P132956 | *UCHL1* | 1.11E-10 | 13.21 | down | 2.49E-04 | 4.09 | up |
| A_23_P433016 | *FBLN1* | 8.05E-13 | 12.99 | down | 9.13E-05 | 3.82 | up |
| A_23_P58588 | *SLIT3* | 3.42E-14 | 12.05 | down | 2.55E-03 | 2.32 | up |
| A_23_P501007 | *EFEMP1* | 2.77E-12 | 9.54 | down | 2.83E-04 | 3.09 | up |
| A_23_P204286 | *MGP* | 1.77E-14 | 9.50 | down | 2.09E-05 | 3.23 | up |
| A_23_P103256 | *CFHR3* | 8.67E-16 | 9.06 | down | 3.63E-04 | 2.25 | up |
| A_23_P200160 | *CFH* | 3.65E-11 | 8.65 | down | 6.86E-03 | 2.22 | up |
| A_23_P217269 | *VSIG4* | 3.01E-10 | 8.43 | down | 3.99E-04 | 3.18 | up |
| A_23_P64873 | *DCN* | 3.30E-09 | 8.23 | down | 1.40E-04 | 4.08 | up |
| A_32_P97169 | *GPC6* | 1.05E-12 | 8.14 | down | 6.40E-06 | 4.15 | up |
| A_23_P104252 | *ITIH5* | 4.52E-10 | 8.12 | down | 2.88E-03 | 2.67 | up |
| A_23_P30614 | *PLN* | 2.07E-07 | 8.08 | down | 6.24E-03 | 3.10 | up |
| A_23_P114740 | *CFH* | 2.96E-11 | 7.69 | down | 1.72E-03 | 2.52 | up |
| A_32_P210168 | *C15orf59* | 1.30E-15 | 7.69 | down | 3.05E-07 | 3.53 | up |
| A_23_P216429 | *ASPN* | 5.29E-12 | 7.49 | down | 3.22E-12 | 12.48 | up |
| A_23_P46426 | *CYR61* | 1.09E-08 | 7.19 | down | 2.19E-05 | 4.94 | up |
| A_23_P332399 | *GULP1* | 7.81E-07 | 7.04 | down | 6.67E-03 | 3.57 | up |
| A_23_P46429 | *CYR61* | 1.64E-09 | 6.87 | down | 6.04E-06 | 4.81 | up |
| A_24_P370946 | *CYR61* | 6.49E-08 | 6.71 | down | 4.28E-05 | 4.67 | up |
| A_23_P211468 |  | 5.96E-07 | 6.68 | down | 2.42E-03 | 3.54 | up |
| A_23_P360964 | *DACT3* | 3.82E-12 | 6.29 | down | 1.11E-04 | 2.73 | up |
| A_23_P112470 | *CCL21* | 1.04E-09 | 6.20 | down | 5.32E-05 | 2.74 | up |
| A_23_P113351 | *SPARCL1* | 2.28E-11 | 6.11 | down | 4.95E-03 | 2.03 | up |
| A_23_P52697 | *CD248* | 1.79E-09 | 6.02 | down | 1.86E-07 | 6.48 | up |
| A_23_P217688 | *TSC22D3* | 1.24E-06 | 5.95 | down | 2.37E-03 | 2.97 | up |
| A_23_P101407 | *C3* | 5.13E-05 | 5.88 | down | 4.52E-03 | 4.18 | up |
| A_24_P119745 | *FN1* | 3.36E-04 | 5.86 | down | 1.84E-04 | 9.38 | up |
| A_24_P319923 | *MYLK* | 6.98E-10 | 5.79 | down | 1.12E-03 | 2.58 | up |
| A_24_P206776 | *CRYAB* | 2.31E-15 | 5.69 | down | 1.96E-04 | 2.09 | up |
| A_23_P143817 | *MYLK* | 8.44E-10 | 5.12 | down | 1.63E-03 | 2.33 | up |
| A_23_P52266 | *IFIT1* | 2.42E-07 | 5.10 | down | 1.92E-03 | 3.00 | up |
| A_23_P411993 | *ITIH5* | 3.16E-12 | 5.07 | down | 2.69E-04 | 2.11 | up |
| A_23_P164650 | *APOE* | 1.46E-06 | 4.80 | down | 9.61E-03 | 2.79 | up |
| A_23_P310956 | *COL6A2* | 1.16E-09 | 4.80 | down | 2.27E-03 | 2.43 | up |
| A_23_P150394 | *FXYD6* | 1.43E-14 | 4.69 | down | 8.99E-05 | 2.16 | up |
| A_32_P74409 | *AG2* | 1.45E-06 | 4.49 | down | 3.74E-05 | 4.36 | up |
| A_24_P561165 |  | 5.15E-10 | 4.42 | down | 7.91E-06 | 3.26 | up |
| A_23_P372834 | *AQP1* | 1.01E-04 | 4.38 | down | 2.71E-03 | 3.96 | up |
| A_32_P171313 | *GNB4* | 9.03E-09 | 4.36 | down | 1.07E-04 | 2.95 | up |
| A_23_P69573 | *GUCY1A3* | 4.53E-08 | 4.31 | down | 1.22E-07 | 5.69 | up |
| A_23_P45871 | *IFI44L* | 1.23E-04 | 4.28 | down | 2.37E-03 | 3.43 | up |
| A_23_P134426 | *GPNMB* | 2.34E-07 | 4.26 | down | 1.07E-03 | 2.69 | up |
| A_23_P75260 | *RASSF4* | 7.18E-08 | 4.26 | down | 1.39E-03 | 2.48 | up |
| A_23_P65678 | *FBN1* | 1.04E-09 | 4.22 | down | 1.26E-07 | 4.63 | up |
| A_23_P89431 | *CCL2* | 9.29E-06 | 4.13 | down | 1.07E-03 | 3.18 | up |
| A_23_P167096 | *VEGFC* | 7.23E-07 | 4.09 | down | 1.63E-05 | 4.03 | up |
| A_24_P919850 | *BDKRB1* | 8.42E-08 | 4.05 | down | 3.49E-03 | 2.12 | up |
| A_23_P117782 | *LARP6* | 4.41E-05 | 4.05 | down | 9.43E-03 | 2.84 | up |
| A_23_P161727 | *HSPB2* | 3.74E-09 | 4.02 | down | 7.36E-05 | 2.68 | up |
| A_23_P139123 | *SERPING1* | 3.59E-09 | 4.02 | down | 4.47E-05 | 2.95 | up |
| A_23_P399078 | *TIMP3* | 2.79E-08 | 3.99 | down | 1.20E-05 | 4.16 | up |
| A_23_P208991 | *PALM* | 1.47E-09 | 3.98 | down | 2.73E-03 | 2.17 | up |
| A_23_P256205 | *ABLIM3* | 1.49E-09 | 3.87 | down | 3.25E-04 | 2.44 | up |
| A_23_P19894 | *AQP1* | 3.25E-04 | 3.83 | down | 5.35E-03 | 3.55 | up |
| A_23_P154115 | *IGFBP5* | 8.53E-06 | 3.82 | down | 3.57E-06 | 5.65 | up |
| A_23_P210425 | *MYL9* | 5.00E-09 | 3.78 | down | 3.95E-05 | 3.08 | up |
| A_23_P100711 | *PMP22* | 2.91E-09 | 3.78 | down | 3.03E-04 | 2.34 | up |
| A_23_P99063 | *LUM* | 1.83E-06 | 3.77 | down | 1.28E-07 | 7.03 | up |
| A_23_P2492 | *C1S* | 2.62E-08 | 3.76 | down | 3.38E-04 | 2.59 | up |
| A_23_P128744 | *BDKRB1* | 2.85E-07 | 3.75 | down | 5.12E-03 | 2.05 | up |
| A_23_P202881 | *FEZ1* | 1.71E-08 | 3.74 | down | 3.67E-03 | 2.01 | up |
| A_23_P76480 |  | 2.69E-08 | 3.72 | down | 9.49E-04 | 2.33 | up |
| A_23_P114947 | *RGS2* | 6.25E-07 | 3.70 | down | 8.50E-03 | 2.10 | up |
| A_23_P3312 | *ISLR* | 1.18E-07 | 3.68 | down | 2.18E-08 | 7.45 | up |
| A_32_P16204 | *LOC375295* | 5.97E-08 | 3.64 | down | 7.14E-04 | 2.29 | up |
| A_23_P431410 | *RBMS1* | 2.81E-07 | 3.62 | down | 4.42E-04 | 2.74 | up |
| A_23_P110403 | *PDLIM3* | 6.66E-07 | 3.61 | down | 6.19E-03 | 2.18 | up |
| A_23_P44724 | *CSRP2* | 1.24E-07 | 3.58 | down | 1.87E-06 | 3.61 | up |
| A_23_P157879 | *FCN1* | 3.25E-04 | 3.54 | down | 4.72E-03 | 2.70 | up |
| A_23_P74088 | *MMP23B* | 5.89E-09 | 3.53 | down | 6.30E-04 | 2.26 | up |
| A_23_P52336 | *UNC5B* | 1.22E-05 | 3.49 | down | 7.79E-03 | 2.40 | up |
| A_23_P29124 | *38596* | 3.79E-09 | 3.41 | down | 5.47E-06 | 2.94 | up |
| A_23_P361014 | *TSHZ3* | 7.49E-06 | 3.37 | down | 3.18E-03 | 2.51 | up |
| A_32_P108254 | *FAM20A* | 5.35E-08 | 3.32 | down | 6.09E-06 | 3.44 | up |
| A_24_P240166 | *PHLDB2* | 4.28E-07 | 3.29 | down | 8.12E-03 | 2.00 | up |
| A_23_P91334 | *HSPA12B* | 2.29E-06 | 3.27 | down | 1.02E-03 | 2.46 | up |
| A_32_P215938 | *GPSM1* | 1.18E-05 | 3.27 | down | 6.63E-04 | 3.32 | up |
| A_23_P97141 | *RGS1* | 1.85E-04 | 3.25 | down | 1.02E-03 | 3.34 | up |
| A_23_P317620 | *ARL4C* | 3.74E-05 | 3.24 | down | 2.42E-05 | 4.28 | up |
| A_23_P29939 | *SNCA* | 3.55E-08 | 3.20 | down | 1.02E-03 | 2.10 | up |
| A_23_P157299 | *AEBP1* | 5.19E-06 | 3.19 | down | 2.22E-05 | 4.86 | up |
| A_23_P58251 | *CPZ* | 3.25E-05 | 3.18 | down | 1.28E-07 | 7.20 | up |
| A_24_P201702 | *CLEC2B* | 2.83E-06 | 3.18 | down | 6.97E-03 | 2.07 | up |
| A_23_P60856 | *TSPAN4* | 7.66E-09 | 3.17 | down | 3.61E-05 | 2.50 | up |
| A_23_P125423 | *C1R* | 6.66E-07 | 3.16 | down | 1.27E-03 | 2.36 | up |
| A_23_P61945 | *MITF* | 1.32E-06 | 3.16 | down | 1.88E-04 | 2.81 | up |
| A_32_P42895 |  | 1.76E-05 | 3.15 | down | 6.10E-07 | 5.56 | up |
| A_23_P100660 | *SERPINF1* | 1.52E-07 | 3.14 | down | 1.14E-04 | 2.62 | up |
| A_23_P161190 | *VIM* | 1.16E-07 | 3.13 | down | 7.23E-04 | 2.30 | up |
| A_23_P395438 | *HTRA3* | 2.20E-05 | 3.11 | down | 5.39E-08 | 7.30 | up |
| A_24_P912985 |  | 3.37E-08 | 3.09 | down | 2.50E-04 | 2.11 | up |
| A_23_P111701 | *GNG11* | 2.59E-06 | 3.07 | down | 8.16E-04 | 2.43 | up |
| A_24_P315256 |  | 2.85E-06 | 3.04 | down | 7.90E-04 | 2.53 | up |
| A_23_P109143 | *PRNP* | 8.79E-06 | 3.03 | down | 1.17E-03 | 2.56 | up |
| A_24_P921366 | *CALD1* | 1.59E-06 | 3.00 | down | 4.28E-05 | 3.23 | up |
| A_23_P112241 | *DNAJB5* | 1.18E-06 | 2.99 | down | 6.32E-04 | 2.33 | up |
| A_23_P87013 | *TAGLN* | 1.42E-07 | 2.98 | down | 4.43E-05 | 2.74 | up |
| A_23_P87011 | *TAGLN* | 6.69E-07 | 2.96 | down | 1.12E-04 | 2.68 | up |
| A_23_P24414 | *EFEMP2* | 4.58E-07 | 2.92 | down | 1.23E-05 | 3.20 | up |
| A_23_P315571 | *RFTN1* | 3.21E-06 | 2.91 | down | 4.75E-04 | 2.52 | up |
| A_23_P14174 | *TNFSF13B* | 4.52E-07 | 2.91 | down | 1.91E-04 | 2.17 | up |
| A_23_P145916 | *AEBP1* | 5.29E-05 | 2.87 | down | 1.95E-04 | 4.22 | up |
| A_32_P62863 | *SCHIP1* | 4.66E-05 | 2.86 | down | 2.56E-04 | 3.22 | up |
| A_23_P43276 | *GPR124* | 9.70E-07 | 2.84 | down | 2.75E-04 | 2.62 | up |
| A_23_P301521 | *KIAA1462* | 5.13E-04 | 2.84 | down | 3.48E-05 | 4.70 | up |
| A_23_P25974 | *TTC7B* | 1.34E-04 | 2.84 | down | 4.48E-04 | 3.41 | up |
| A_24_P236799 | *RAB31* | 5.27E-05 | 2.83 | down | 2.12E-06 | 5.22 | up |
| A_23_P161194 | *VIM* | 3.03E-06 | 2.81 | down | 4.13E-03 | 2.09 | up |
| A_23_P96568 | *FLNA* | 1.92E-06 | 2.81 | down | 1.09E-03 | 2.50 | up |
| A_24_P156113 | *EHD2* | 3.43E-06 | 2.81 | down | 8.89E-03 | 2.06 | up |
| A_23_P36496 | *RBMS1* | 2.85E-05 | 2.81 | down | 2.54E-03 | 2.45 | up |
| A_23_P42575 | *CALD1* | 5.76E-05 | 2.81 | down | 4.82E-05 | 3.75 | up |
| A_23_P27332 | *TCF4* | 1.08E-05 | 2.80 | down | 8.76E-03 | 2.02 | up |
| A_23_P19663 | *CTGF* | 4.06E-06 | 2.77 | down | 2.19E-05 | 2.99 | up |
| A_24_P135322 | *NRP1* | 1.21E-06 | 2.77 | down | 2.38E-05 | 3.09 | up |
| A_23_P251499 | *PCOLCE* | 1.78E-07 | 2.76 | down | 5.90E-07 | 4.02 | up |
| A_23_P421401 | *PDGFRB* | 1.32E-05 | 2.76 | down | 3.67E-07 | 5.32 | up |
| A_23_P103496 | *GBP4* | 5.93E-03 | 2.75 | down | 2.54E-03 | 3.46 | up |
| A_23_P32444 | *MXRA8* | 2.65E-06 | 2.70 | down | 1.39E-05 | 3.58 | up |
| A_32_P162520 |  | 8.43E-03 | 2.69 | down | 2.59E-03 | 3.57 | up |
| A_32_P74942 |  | 4.37E-03 | 2.69 | down | 1.26E-03 | 4.00 | up |
| A_32_P143824 |  | 5.06E-07 | 2.67 | down | 5.02E-05 | 2.42 | up |
| A_23_P86653 | *SRGN* | 7.84E-06 | 2.65 | down | 1.94E-03 | 2.19 | up |
| A_23_P129695 | *VASN* | 2.33E-05 | 2.61 | down | 5.83E-05 | 3.43 | up |
| A_23_P152876 | *RAB34* | 6.50E-06 | 2.57 | down | 2.48E-04 | 2.60 | up |
| A_23_P428129 | *CDKN1C* | 1.97E-04 | 2.54 | down | 8.14E-04 | 3.10 | up |
| A_24_P402242 | *COL3A1* | 9.75E-04 | 2.53 | down | 6.09E-06 | 5.74 | up |
| A_23_P142533 | *COL3A1* | 1.14E-04 | 2.52 | down | 4.94E-07 | 5.65 | up |
| A_23_P141688 | *RAB31* | 9.73E-05 | 2.50 | down | 6.69E-07 | 5.23 | up |
| A_24_P131522 | *ANTXR1* | 2.91E-07 | 2.49 | down | 3.71E-07 | 4.39 | up |
| A_23_P35114 | *PLEKHO1* | 2.02E-06 | 2.46 | down | 6.71E-04 | 2.05 | up |
| A_23_P74290 | *GBP5* | 9.06E-03 | 2.45 | down | 1.62E-03 | 3.13 | up |
| A_23_P72651 | *ECSCR* | 2.85E-04 | 2.41 | down | 9.09E-04 | 2.49 | up |
| A_24_P316059 |  | 9.04E-05 | 2.40 | down | 6.10E-07 | 5.25 | up |
| A_23_P102000 | *CXCR4* | 9.12E-04 | 2.39 | down | 2.50E-04 | 3.38 | up |
| A_23_P114903 | *HSPA6* | 1.74E-06 | 2.39 | down | 4.37E-05 | 2.42 | up |
| A_24_P370372 | *CBX6* | 1.77E-08 | 2.36 | down | 6.56E-05 | 2.10 | up |
| A_32_P198412 |  | 2.62E-04 | 2.35 | down | 1.31E-03 | 2.45 | up |
| A_23_P20864 | *ANGPTL2* | 5.94E-05 | 2.34 | down | 1.53E-05 | 3.69 | up |
| A_23_P394064 | *PTRF* | 3.76E-06 | 2.34 | down | 1.51E-04 | 2.45 | up |
| A_32_P32254 | *COL6A1* | 1.75E-04 | 2.33 | down | 4.68E-03 | 2.29 | up |
| A_32_P47754 | *SLC2A14* | 6.54E-04 | 2.32 | down | 4.01E-05 | 3.79 | up |
| A_23_P502142 | *FYN* | 4.23E-05 | 2.30 | down | 6.82E-04 | 2.30 | up |
| A_24_P277934 | *COL1A2* | 5.58E-04 | 2.30 | down | 6.71E-07 | 6.10 | up |
| A_23_P39465 | *BST2* | 1.84E-03 | 2.29 | down | 8.49E-04 | 3.57 | up |
| A_23_P119353 | *RASIP1* | 6.43E-05 | 2.29 | down | 2.45E-04 | 2.44 | up |
| A_23_P20566 | *TPM2* | 1.42E-05 | 2.27 | down | 1.04E-04 | 2.52 | up |
| A_23_P86390 | *NRP1* | 4.44E-03 | 2.27 | down | 2.70E-03 | 2.59 | up |
| A_23_P254271 | *TUBB6* | 9.52E-06 | 2.26 | down | 4.57E-04 | 2.14 | up |
| A_23_P127565 | *LAYN* | 4.12E-06 | 2.25 | down | 7.59E-07 | 3.16 | up |
| A_23_P51926 | *PTAFR* | 3.48E-04 | 2.24 | down | 4.28E-03 | 2.12 | up |
| A_24_P145911 | *TRA2B* | 5.17E-04 | 2.23 | down | 2.83E-04 | 2.45 | up |
| A_23_P101093 | *COPZ2* | 7.49E-06 | 2.21 | down | 8.38E-04 | 2.19 | up |
| A_23_P144959 | *VCAN* | 6.70E-04 | 2.20 | down | 4.61E-06 | 4.13 | up |
| A_23_P203475 | *PRKCDBP* | 9.08E-04 | 2.19 | down | 1.05E-03 | 2.71 | up |
| A_23_P401076 | *SUSD3* | 4.82E-04 | 2.19 | down | 7.20E-04 | 2.81 | up |
| A_23_P19226 | *DSE* | 1.89E-04 | 2.18 | down | 5.92E-03 | 2.00 | up |
| A_24_P215352 | *PRKCDBP* | 8.37E-06 | 2.15 | down | 2.94E-06 | 2.95 | up |
| A_24_P261417 | *DKK3* | 5.48E-04 | 2.11 | down | 1.43E-04 | 2.98 | up |
| A_23_P61987 | *TMEM121* | 2.20E-05 | 2.11 | down | 2.37E-04 | 2.16 | up |
| A_23_P17430 | *RBM38* | 7.89E-04 | 2.11 | down | 3.48E-03 | 2.15 | up |
| A_32_P32739 | *NAGS* | 2.79E-05 | 2.10 | down | 1.56E-03 | 2.26 | up |
| A_23_P3911 | *PLXDC1* | 5.72E-03 | 2.09 | down | 9.57E-03 | 2.41 | up |
| A_32_P70158 | *LILRB3* | 9.58E-03 | 2.09 | down | 9.49E-04 | 3.02 | up |
| A_23_P368711 | *LILRB3* | 2.82E-04 | 2.09 | down | 4.58E-03 | 2.00 | up |
| A_23_P91390 | *THBD* | 5.78E-03 | 2.06 | down | 8.86E-03 | 2.31 | up |
| A_24_P935491 | *COL3A1* | 4.19E-04 | 2.06 | down | 3.44E-07 | 5.08 | up |
| A_24_P819729 |  | 9.56E-03 | 2.02 | down | 8.66E-03 | 2.12 | up |
| A_23_P406187 | *NAGS* | 2.60E-04 | 2.01 | down | 6.06E-03 | 2.11 | up |
| A_23_P122924 | *INHBA* | 1.66E-06 | 5.72 | up | 1.22E-05 | 7.18 | up |
| A_23_P54055 | *JUB* | 2.03E-08 | 4.24 | up | 2.75E-03 | 2.07 | up |
| A_23_P210690 | *TRIB3* | 1.73E-04 | 3.17 | up | 3.10E-03 | 3.11 | up |
| A_23_P259692 | *PSAT1* | 4.48E-03 | 2.47 | up | 1.47E-03 | 2.81 | up |
| A_23_P143190 | *MYBL2* | 3.96E-05 | 2.40 | up | 2.58E-03 | 2.16 | up |
| A_23_P340909 | *SKA3* | 1.05E-04 | 2.15 | up | 3.87E-04 | 2.16 | up |
| A_23_P209978 | *VSNL1* | 7.50E-04 | 2.15 | up | 4.93E-03 | 2.59 | up |
| A_23_P62115 | *TIMP1* | 3.34E-04 | 2.11 | up | 4.62E-03 | 2.08 | up |
| A_32_P184933 | *UBE2S* | 2.74E-05 | 2.01 | up | 4.22E-05 | 2.27 | up |
