# Supplementary material for: A Gene Expression and Pre-mRNA Splicing Signature That Marks the Adenoma-Adenocarcinoma Progression in Colorectal Cancer
Source: PLoS One. 2014 Feb 6;9(2):e87761. doi: 10.1371/journal.pone.0087761 (PMC3916340; doi:10.1371/journal.pone.0087761)
Supplement: Table S11 — KEGG gene sets enriched in the gene expression signature of 265 probes. (DOC) [file pone.0087761.s017.doc]

**Table S11. KEGG gene sets enriched in the gene expression signature of 265 probes.** The KEGG pathway analysis showed eight enriched gene sets (P-value ≤ 0.05).

| Pathway in the Signature of 265 Probes | P-value | Benjamini-Hochberg | Fold  Enrichment | Number of Genes in the Pathway | Number of Deregulated Genes | Percentage of Deregulated Genes | Deregulated Genes in the Pathway |
| --- | --- | --- | --- | --- | --- | --- | --- |
| Complement and coagulation cascades | 5.4E-05 | 4.6E-03 | 7.86 | 69 | 8 | 12% | *BDKRB1, C1R, C1S, C3, CFH, SERPINA1, SERPING1, THBD* |
| Focal adhesion | 1.3E-04 | 5.6E-03 | 4.05 | 201 | 12 | 6% | *COL1A2, COL3A1, COL6A1, COL6A2, FLNA, FN1, FYN, MYL9, MYLK, PDGFRB, SHC2, VEGFC* |
| Calcium signaling pathway | 1.3E-02 | 3.2E-01 | 3.08 | 176 | 8 | 5% | *BDKRB1, CACNA1C, GNA15, MYLK, PDGFRB, PLCB4, PLN, PTAFR* |
| Vascular smooth muscle contraction | 2.3E-02 | 3.9E-01 | 3.63 | 112 | 6 | 5% | *CACNA1C, CALD1, GUCY1A3, MYL9, MYLK, PLCB4* |
| ECM-receptor interaction | 3.3E-02 | 4.4E-01 | 4.04 | 84 | 5 | 6% | *COL1A2, COL3A1, COL6A1, COL6A2, FN1* |
| TGF-*beta* signaling pathway | 3.7E-02 | 4.2E-01 | 3.90 | 87 | 5 | 6% | *DCN, ID4, INHBA, SMAD6, SMAD9* |
| Axon guidance | 3.9E-02 | 3.8E-01 | 3.15 | 129 | 6 | 5% | *ABLIM3, CXCR4, FYN, NRP1, SLIT3, UNC5B* |
| Chemokine signaling pathway | 5.4E-02 | 4.4E-01 | 2.54 | 187 | 7 | 4% | *CCL2, CCL21, CXCR4, GNB4, GNG11, PLCB4, SHC2* |
