# Supplementary material for: A Gene Expression and Pre-mRNA Splicing Signature That Marks the Adenoma-Adenocarcinoma Progression in Colorectal Cancer
Source: PLoS One. 2014 Feb 6;9(2):e87761. doi: 10.1371/journal.pone.0087761 (PMC3916340; doi:10.1371/journal.pone.0087761)
Supplement: Table S12 — List of the up- and down-regulated genes in colorectal adenomas in comparison with normal mucosae on Affymetrix™ Human Exon 1.0 ST arrays. (DOC) [file pone.0087761.s018.doc]

**Table S12. List of the up- and down-regulated genes in colorectal adenomas in comparison with normal mucosae on Affymetrix^TM^ Human Exon 1.0 ST arrays.** The results of Human Exon 1.0 ST (Affymetrix^TM^) for the deregulated genes in CRA *vs*. NOR are presented (≥ 1.5 FC, P-value ≤ 0.05 by *t*-test).

| Gene Symbol | Representative Transcript ID | EnsEMBL ID | Regulation | Fold-Change | P-Value | Intensity Normal | Intensity Adenoma | Low-specificity Probe Included? |
| --- | --- | --- | --- | --- | --- | --- | --- | --- |
| *CLDN2* | AF177340 | ENSG00000165376 | up | 9.47 | 1.75E-12 | 5.89 | 9.14 | No |
| *OLFM4* | AK000683 | ENSG00000102837 | up | 9.36 | 1.25E-11 | 9.33 | 12.55 | No |
| *DPEP1* | CR620135 | ENSG00000015413 | up | 9.23 | 6.33E-08 | 6.37 | 9.58 | No |
| *---* | AK125310 | --- | up | 8.31 | 4.22E-05 | 5.33 | 8.39 | No |
| *---* | BC065763 | --- | up | 8.08 | 1.55E-05 | 4.75 | 7.77 | No |
| *KLK11* | BC022068 | ENSG00000167757 | up | 6.84 | 9.18E-04 | 5.58 | 8.35 | No |
| *HOXB8* | AK096222 | ENSG00000120068 | up | 5.60 | 8.40E-04 | 6.29 | 8.78 | No |
| *KLK6* | AY279383 | ENSG00000167755 | up | 5.36 | 1.32E-09 | 5.47 | 7.90 | No |
| *KLK10* | BC002710 | ENSG00000129451 | up | 5.29 | 2.37E-08 | 5.88 | 8.28 | No |
| *MMP7* | EU176770 | ENSG00000137673 | up | 5.22 | 1.55E-02 | 6.70 | 9.08 | No |
| *MSX2* | D89377 | ENSG00000120149 | up | 5.04 | 2.91E-02 | 5.65 | 7.99 | No |
| *KIAA1199* | AY585237 | ENSG00000103888 | up | 4.57 | 2.52E-03 | 6.00 | 8.19 | No |
| *---* | BC050296 | --- | up | 4.42 | 1.69E-05 | 6.25 | 8.40 | No |
| *NKD1* | BC051288 | ENSG00000140807 | up | 4.00 | 2.32E-02 | 6.24 | 8.24 | No |
| *---* | AK023784 | --- | up | 3.88 | 1.83E-02 | 5.38 | 7.34 | No |
| *CLDN1* | AK225963 | ENSG00000163347 | up | 3.79 | 7.56E-03 | 7.56 | 9.48 | No |
| *GDF15* | AB000584 | ENSG00000130513 | up | 3.79 | 1.10E-03 | 8.34 | 10.27 | No |
| *QPCT* | BC047756 | ENSG00000115828 | up | 3.77 | 8.04E-04 | 6.90 | 8.82 | No |
| *CYP4X1* | AK131355 | ENSG00000186377 | up | 3.59 | 3.39E-02 | 5.71 | 7.55 | No |
| *AZGP1* | BC033830 | ENSG00000160862 | up | 3.57 | 1.11E-02 | 6.14 | 7.97 | No |
| *UCA1* | AK092401 | ENSG00000214049 | up | 3.49 | 1.50E-05 | 7.31 | 9.11 | Yes |
| *HOXB9* | AK025239 | ENSG00000170689 | up | 3.35 | 2.30E-03 | 7.29 | 9.04 | No |
| *PLCB4* | AK307837 | ENSG00000101333 | up | 3.18 | 2.56E-03 | 7.74 | 9.41 | No |
| *DGAT2* | AB048286 | ENSG00000062282 | up | 3.13 | 1.73E-12 | 6.10 | 7.75 | No |
| *---* | AK124321 | --- | up | 3.10 | 3.34E-02 | 6.32 | 7.95 | No |
| *NETO2* | BC098380 | ENSG00000171208 | up | 3.07 | 2.69E-02 | 7.48 | 9.09 | No |
| *AREG* | BC009799 | ENSG00000109321 | up | 3.06 | 9.02E-03 | 7.23 | 8.84 | No |
| *RNF43* | AB081837 | ENSG00000108375 | up | 3.03 | 5.02E-03 | 8.14 | 9.74 | No |
| *---* | BX640973 | --- | up | 3.00 | 4.20E-03 | 5.80 | 7.38 | No |
| *PITX2* | AK127829 | ENSG00000164093 | up | 2.95 | 1.12E-02 | 5.77 | 7.33 | No |
| *ASCL2* | BC028140 | ENSG00000183734 | up | 2.89 | 5.24E-03 | 6.93 | 8.46 | No |
| *PTP4A3* | AK311257 | ENSG00000184489 | up | 2.88 | 3.25E-02 | 7.24 | 8.76 | No |
| *---* | BX648498 | --- | up | 2.80 | 1.46E-03 | 6.47 | 7.96 | Yes |
| *GRM8* | AK290197 | ENSG00000179603 | up | 2.78 | 3.90E-02 | 5.67 | 7.15 | No |
| *---* | AK025743 | --- | up | 2.77 | 6.52E-03 | 6.85 | 8.33 | No |
| *VWA2* | AY572972 | ENSG00000165816 | up | 2.74 | 5.28E-03 | 6.22 | 7.67 | No |
| *OXGR1* | AK122639 | ENSG00000165621 | up | 2.72 | 1.81E-02 | 6.11 | 7.56 | No |
| *---* | BC094703 | --- | up | 2.69 | 7.68E-03 | 6.41 | 7.84 | No |
| *C13orf18* | AK125950 | ENSG00000102445 | up | 2.63 | 6.80E-03 | 6.64 | 8.04 | No |
| *GSTP1* | BC010915 | ENSG00000084207 | up | 2.63 | 2.23E-07 | 10.36 | 11.75 | No |
| *GPSM2* | AB445462 | ENSG00000121957 | up | 2.57 | 1.69E-02 | 7.20 | 8.57 | No |
| *CASP1* | AK128199 | ENSG00000137752 | up | 2.56 | 1.22E-02 | 7.38 | 8.74 | No |
| *BMP7* | AK094784 | ENSG00000101144 | up | 2.52 | 3.81E-02 | 6.28 | 7.61 | No |
| *CCNI2* | AK131553 | ENSG00000205089 | up | 2.52 | 5.65E-11 | 5.96 | 7.30 | No |
| *FAM57A* | AF477201 | ENSG00000167695 | up | 2.51 | 2.92E-03 | 7.75 | 9.08 | No |
| *LGR6* | AB049405 | ENSG00000133067 | up | 2.50 | 5.41E-10 | 6.16 | 7.49 | No |
| *REEP6* | AX775999 | ENSG00000115255 | up | 2.49 | 2.38E-03 | 7.49 | 8.80 | No |
| *LRRC6* | BC047286 | ENSG00000129295 | up | 2.49 | 5.90E-03 | 6.05 | 7.37 | No |
| *CKS2* | CR621720 | ENSG00000123975 | up | 2.47 | 2.73E-02 | 7.04 | 8.34 | No |
| *TNFRSF12A* | BC002718 | ENSG00000006327 | up | 2.44 | 8.38E-03 | 7.57 | 8.86 | No |
| *HSPH1* | BX648125 | ENSG00000120694 | up | 2.44 | 4.98E-04 | 7.73 | 9.02 | No |
| *SLC22A3* | BC172500 | ENSG00000146477 | up | 2.44 | 5.30E-03 | 5.94 | 7.23 | No |
| *PVT1* | BC041065 | ENSG00000249859 | up | 2.41 | 7.48E-03 | 6.77 | 8.04 | No |
| *FABP6* | DQ132786 | ENSG00000170231 | up | 2.38 | 6.64E-04 | 7.06 | 8.31 | No |
| *SPRYD4* | AL832247 | ENSG00000176422 | up | 2.37 | 4.12E-05 | 8.87 | 10.11 | No |
| *HSPA4L* | AK303053 | ENSG00000164070 | up | 2.36 | 2.46E-02 | 5.79 | 7.03 | No |
| *---* | AL161960 | --- | up | 2.35 | 5.59E-05 | 6.51 | 7.75 | No |
| *AEN* | BC020988 | ENSG00000181026 | up | 2.35 | 8.00E-05 | 6.75 | 7.98 | No |
| *ENC1* | AF059611 | ENSG00000171617 | up | 2.33 | 1.60E-03 | 7.71 | 8.93 | No |
| *C7orf40* | BC092459 | ENSG00000232956 | up | 2.31 | 2.85E-02 | 6.28 | 7.48 | No |
| *TBX3* | AF216750 | ENSG00000135111 | up | 2.30 | 4.29E-02 | 7.87 | 9.08 | No |
| *GTF2IRD1* | AF151354 | ENSG00000006704 | up | 2.29 | 3.46E-03 | 7.12 | 8.32 | No |
| *CXCL16* | AK027389 | ENSG00000161921 | up | 2.27 | 1.60E-03 | 8.36 | 9.54 | No |
| *QPRT* | BC005060 | ENSG00000103485 | up | 2.27 | 1.63E-02 | 7.94 | 9.12 | No |
| *ANXA3* | CR601701 | ENSG00000138772 | up | 2.26 | 5.56E-03 | 8.39 | 9.56 | No |
| *SMAD6* | AF035528 | ENSG00000137834 | up | 2.26 | 8.44E-05 | 6.76 | 7.94 | No |
| *SERPINA3 // SERPINA5* | AX747885 | ENSG00000188488 // ENSG00000196136 | up | 2.24 | 1.12E-03 | 6.50 | 7.67 | No |
| *CCND2* | D13639 | ENSG00000118971 | up | 2.24 | 9.22E-04 | 9.36 | 10.52 | No |
| *---* | AF086401 | --- | up | 2.23 | 1.91E-02 | 6.30 | 7.46 | No |
| *ID1* | CR599901 | ENSG00000125968 | up | 2.22 | 4.38E-02 | 10.01 | 11.16 | Yes |
| *SPATA18* | BC025396 | ENSG00000163071 | up | 2.21 | 2.93E-02 | 6.29 | 7.43 | No |
| *CTSH* | CR596280 | ENSG00000103811 | up | 2.21 | 1.68E-02 | 7.68 | 8.83 | No |
| *CCL24* | AK312216 | ENSG00000106178 | up | 2.21 | 1.78E-02 | 7.94 | 9.08 | No |
| *MET* | BC130420 | ENSG00000105976 | up | 2.20 | 3.26E-03 | 7.95 | 9.08 | No |
| *---* | AL157495 | --- | up | 2.20 | 1.10E-02 | 4.63 | 5.77 | No |
| *---* | AK125440 | --- | up | 2.19 | 2.79E-02 | 5.48 | 6.61 | No |
| *CXCL3* | BC016308 | ENSG00000163734 | up | 2.19 | 7.02E-07 | 8.17 | 9.30 | Yes |
| *FERMT1* | AB105105 | ENSG00000101311 | up | 2.18 | 7.54E-03 | 9.00 | 10.12 | No |
| *GPR56* | AK131550 | ENSG00000205336 | up | 2.18 | 1.00E-04 | 8.42 | 9.54 | No |
| *KCNQ1* | AF000571 | ENSG00000053918 | up | 2.18 | 8.44E-10 | 7.69 | 8.81 | No |
| *---* | CR622189 | --- | up | 2.17 | 1.37E-02 | 5.47 | 6.59 | No |
| *CCDC113* | AL136785 | ENSG00000103021 | up | 2.17 | 5.54E-03 | 5.69 | 6.80 | No |
| *---* | BC029465 | --- | up | 2.17 | 3.25E-02 | 5.27 | 6.39 | Yes |
| *SPATA20* | AK125807 | ENSG00000006282 | up | 2.15 | 7.01E-04 | 8.47 | 9.58 | No |
| *CCNA2* | CR604810 | ENSG00000145386 | up | 2.15 | 1.12E-02 | 8.26 | 9.36 | No |
| *---* | AK297320 | --- | up | 2.14 | 1.19E-04 | 5.92 | 7.02 | No |
| *RARG* | BC072462 | ENSG00000172819 | up | 2.14 | 6.48E-03 | 7.05 | 8.14 | No |
| *GRIN2B* | AB208850 | ENSG00000150086 | up | 2.14 | 1.38E-02 | 6.20 | 7.30 | No |
| *WNT5A* | AK090582 | ENSG00000114251 | up | 2.13 | 5.65E-05 | 6.03 | 7.13 | No |
| *ITGA6* | X53586 | ENSG00000091409 | up | 2.13 | 2.56E-04 | 9.88 | 10.97 | No |
| *ALG1L* | BC073816 | ENSG00000189366 | up | 2.10 | 4.64E-04 | 6.35 | 7.42 | No |
| *APCDD1* | BC053324 | ENSG00000154856 | up | 2.09 | 4.62E-04 | 8.28 | 9.35 | No |
| *FAM111B* | AY457926 | ENSG00000189057 | up | 2.09 | 2.75E-02 | 6.48 | 7.55 | No |
| *FBP1* | AK292199 | ENSG00000165140 | up | 2.09 | 3.17E-04 | 7.81 | 8.87 | No |
| *PRTFDC1* | AK021950 | ENSG00000099256 | up | 2.08 | 4.62E-03 | 6.38 | 7.43 | No |
| *WDR77* | BC016946 | ENSG00000116455 | up | 2.08 | 5.55E-04 | 7.41 | 8.46 | No |
| *ATOH8* | AK096049 | ENSG00000168874 | up | 2.06 | 4.32E-02 | 6.36 | 7.40 | No |
| *---* | BC010544 | --- | up | 2.06 | 2.52E-02 | 6.73 | 7.77 | No |
| *ZYX* | AK316227 | ENSG00000159840 | up | 2.05 | 4.68E-03 | 8.45 | 9.49 | No |
| *KCNN4* | AF022797 | ENSG00000104783 | up | 2.03 | 1.37E-02 | 7.37 | 8.40 | No |
| *SNORD1C* | AB073602 | ENSG00000200185 | up | 2.03 | 7.22E-04 | 9.05 | 10.07 | No |
| *GOLT1A* | AK124645 | ENSG00000174567 | up | 2.03 | 1.27E-02 | 6.02 | 7.05 | No |
| *SLC16A4* | BC021664 | ENSG00000168679 | up | 2.02 | 1.14E-06 | 6.08 | 7.09 | No |
| *BAX* | AK291076 | ENSG00000087088 | up | 2.02 | 3.34E-03 | 7.47 | 8.48 | No |
| *ANKRD22* | AX721211 | ENSG00000152766 | up | 2.02 | 1.64E-10 | 7.68 | 8.69 | No |
| *TBXAS1* | BC014117 | ENSG00000059377 | up | 2.02 | 2.82E-02 | 7.92 | 8.94 | No |
| *---* | AK097893 | --- | up | 2.02 | 1.74E-02 | 5.42 | 6.43 | No |
| *ABCE1* | X76388 | ENSG00000164163 | up | 2.02 | 1.01E-02 | 8.04 | 9.05 | No |
| *IL20RA* | AF184971 | ENSG00000016402 | up | 2.00 | 3.97E-02 | 6.53 | 7.54 | No |
| *GNL3* | AF191018 | ENSG00000163938 | up | 1.98 | 8.62E-03 | 7.72 | 8.71 | No |
| *SEMA3F* | AB209259 | ENSG00000001617 | up | 1.98 | 2.22E-11 | 6.97 | 7.96 | No |
| *KPNA2* | U28386 | ENSG00000182481 | up | 1.98 | 1.30E-03 | 9.53 | 10.51 | No |
| *PMEPA1* | AF305616 | ENSG00000124225 | up | 1.97 | 3.74E-02 | 7.46 | 8.43 | No |
| *---* | AK022877 | --- | up | 1.97 | 8.69E-10 | 7.56 | 8.54 | No |
| *TOMM34* | BC007423 | ENSG00000025772 | up | 1.96 | 5.26E-03 | 8.74 | 9.70 | No |
| *UHRF1* | AB177623 | ENSG00000034063 | up | 1.96 | 7.64E-10 | 6.72 | 7.69 | No |
| *G6PC3* | BC021574 | ENSG00000141349 | up | 1.96 | 4.39E-04 | 8.01 | 8.98 | No |
| *PPIL1* | AY359032 | ENSG00000137168 | up | 1.96 | 1.26E-03 | 7.42 | 8.39 | No |
| *---* | BC064478 | --- | up | 1.96 | 1.54E-03 | 7.22 | 8.18 | No |
| *BACE2* | AF188277 | ENSG00000182240 | up | 1.95 | 9.44E-04 | 10.17 | 11.13 | No |
| *---* | AK129685 | --- | up | 1.94 | 4.33E-02 | 4.87 | 5.83 | No |
| *TNFSF15* | AF520785 | ENSG00000181634 | up | 1.94 | 2.30E-02 | 7.03 | 7.98 | No |
| *PLEK2* | BC001226 | ENSG00000100558 | up | 1.93 | 2.92E-03 | 8.73 | 9.68 | No |
| *---* | BC036629 | --- | up | 1.93 | 1.01E-02 | 6.60 | 7.55 | Yes |
| *SLC11A2* | AF064484 | ENSG00000110911 | up | 1.92 | 1.61E-02 | 8.03 | 8.97 | No |
| *---* | AK025100 | --- | up | 1.92 | 3.31E-02 | 5.85 | 6.79 | No |
| *---* | AK097963 | --- | up | 1.92 | 3.62E-02 | 6.44 | 7.38 | No |
| *---* | CR608093 | --- | up | 1.91 | 7.20E-03 | 7.99 | 8.92 | Yes |
| *LMNB2* | CR606147 | ENSG00000176619 | up | 1.91 | 1.84E-03 | 8.60 | 9.54 | No |
| *KRT6C* | BC110639 | ENSG00000170465 | up | 1.91 | 3.79E-05 | 7.05 | 7.98 | Yes |
| *GLB1L2* | BC040641 | ENSG00000149328 | up | 1.91 | 3.72E-02 | 7.54 | 8.48 | No |
| *---* | BC010117 | --- | up | 1.91 | 4.85E-02 | 6.42 | 7.35 | No |
| *---* | BC062707 | --- | up | 1.90 | 1.84E-04 | 8.23 | 9.15 | No |
| *E2F5* | AB209185 | ENSG00000133740 | up | 1.90 | 3.74E-02 | 6.34 | 7.27 | No |
| *GGCT* | BC000625 | ENSG00000006625 | up | 1.90 | 3.08E-02 | 7.37 | 8.30 | No |
| *PTCD3* | BC011832 | ENSG00000132300 | up | 1.89 | 4.66E-03 | 7.61 | 8.53 | No |
| *TRIM16L* | AK293428 | ENSG00000108448 | up | 1.89 | 4.44E-03 | 8.43 | 9.35 | Yes |
| *SLC2A8* | BC019043 | ENSG00000136856 | up | 1.89 | 4.24E-03 | 7.88 | 8.80 | No |
| *S100P* | AF539739 | ENSG00000163993 | up | 1.88 | 2.59E-02 | 10.10 | 11.01 | No |
| *SLCO4A1* | BC015727 | ENSG00000101187 | up | 1.88 | 4.83E-02 | 7.58 | 8.49 | No |
| *SLC7A8* | AL365346 | ENSG00000092068 | up | 1.88 | 1.23E-02 | 8.31 | 9.23 | No |
| *DHX33* | BC050409 | ENSG00000005100 | up | 1.87 | 3.82E-03 | 6.68 | 7.59 | No |
| *ECM1* | AK097205 | ENSG00000143369 | up | 1.87 | 4.82E-02 | 7.05 | 7.95 | No |
| *---* | AY956760 | --- | up | 1.87 | 2.63E-02 | 6.70 | 7.60 | No |
| *RPH3AL* | BC005153 | ENSG00000181031 | up | 1.86 | 2.30E-03 | 6.48 | 7.37 | No |
| *---* | BC042436 | --- | up | 1.86 | 2.84E-02 | 8.93 | 9.83 | No |
| *RAD54B* | AK290437 | ENSG00000197275 | up | 1.86 | 2.26E-02 | 5.99 | 6.88 | No |
| *---* | CR620043 | --- | up | 1.86 | 2.99E-02 | 6.90 | 7.79 | Yes |
| *BYSL* | BC050645 | ENSG00000112578 | up | 1.86 | 4.40E-03 | 6.69 | 7.58 | No |
| *SMOX* | AK293485 | ENSG00000088826 | up | 1.85 | 3.57E-02 | 6.56 | 7.45 | No |
| *PDCD2L* | BC006146 | ENSG00000126249 | up | 1.85 | 1.75E-02 | 6.05 | 6.94 | No |
| *KAZALD1* | AK172864 | ENSG00000107821 | up | 1.85 | 7.81E-04 | 6.75 | 7.64 | No |
| *TGIF1* | AK291112 | ENSG00000177426 | up | 1.84 | 5.36E-03 | 9.93 | 10.81 | No |
| *---* | BC000907 | --- | up | 1.84 | 3.62E-02 | 5.93 | 6.81 | No |
| *PIGW* | AK094752 | ENSG00000184886 | up | 1.83 | 3.34E-03 | 6.10 | 6.97 | No |
| *CD68 // EIF4A1 // SENP3* | BC032613 | ENSG00000129226 // ENSG00000161956 // ENSG00000161960 | up | 1.83 | 3.76E-05 | 10.45 | 11.32 | No |
| *---* | BX537644 | --- | up | 1.83 | 1.02E-04 | 6.62 | 7.50 | No |
| *SNTB1* | AK026095 | ENSG00000172164 | up | 1.83 | 2.41E-02 | 8.15 | 9.02 | No |
| *C6orf125* | AY568085 | ENSG00000137288 | up | 1.83 | 2.32E-03 | 8.75 | 9.62 | No |
| *CMTM8* | AY598783 | ENSG00000170293 | up | 1.82 | 3.30E-03 | 6.79 | 7.65 | No |
| *MRPL4* | CR624335 | ENSG00000105364 | up | 1.82 | 6.00E-03 | 9.04 | 9.90 | No |
| *---* | AK098016 | --- | up | 1.82 | 1.02E-03 | 6.30 | 7.16 | No |
| *C1QBP* | CR607361 | ENSG00000108561 | up | 1.82 | 2.32E-02 | 8.73 | 9.59 | No |
| *TNFRSF10B* | BX538104 | ENSG00000120889 | up | 1.82 | 4.20E-03 | 7.39 | 8.26 | No |
| *SSBP1* | BC093054 | ENSG00000106028 | up | 1.82 | 2.59E-02 | 8.03 | 8.89 | No |
| *---* | AK092541 | --- | up | 1.82 | 2.28E-02 | 5.37 | 6.23 | No |
| *PLAGL2* | D83784 | ENSG00000126003 | up | 1.81 | 1.18E-02 | 6.81 | 7.67 | No |
| *PYCR1* | AK225879 | ENSG00000183010 | up | 1.81 | 3.94E-02 | 8.27 | 9.13 | No |
| *EIF5A* | CR622789 | ENSG00000132507 | up | 1.81 | 4.39E-05 | 9.74 | 10.60 | Yes |
| *LYAR* | CR609495 | ENSG00000145220 | up | 1.81 | 3.83E-02 | 6.16 | 7.02 | No |
| *---* | BC041621 | --- | up | 1.81 | 9.57E-04 | 6.62 | 7.48 | No |
| *NLE1* | AB209111 | ENSG00000073536 | up | 1.80 | 1.54E-03 | 6.87 | 7.72 | No |
| *CDRT1 // TRIM16* | AB209899 | ENSG00000221926 // ENSG00000241322 | up | 1.80 | 5.94E-03 | 9.03 | 9.88 | No |
| *SERPINA1* | BX247968 | ENSG00000197249 | up | 1.80 | 1.41E-02 | 10.89 | 11.74 | No |
| *EPHA1* | M18391 | ENSG00000146904 | up | 1.80 | 1.19E-02 | 6.86 | 7.71 | No |
| *AURKA* | BC027464 | ENSG00000087586 | up | 1.79 | 3.38E-02 | 6.73 | 7.57 | No |
| *---* | AK001612 | --- | up | 1.79 | 6.45E-11 | 5.89 | 6.73 | No |
| *TSR1* | BC110851 | ENSG00000167721 | up | 1.79 | 7.35E-04 | 6.63 | 7.47 | No |
| *TRAP1* | AK093344 | ENSG00000126602 | up | 1.79 | 1.35E-02 | 9.13 | 9.97 | No |
| *SEMA5A* | U52840 | ENSG00000112902 | up | 1.79 | 1.43E-02 | 8.08 | 8.92 | No |
| *TCF7* | BC048769 | ENSG00000081059 | up | 1.79 | 2.95E-02 | 6.65 | 7.49 | No |
| *SPNS2* | BC041772 | ENSG00000183018 | up | 1.78 | 5.98E-07 | 7.51 | 8.35 | No |
| *---* | BC045168 | --- | up | 1.78 | 2.22E-03 | 6.36 | 7.19 | Yes |
| *---* | AK024177 | --- | up | 1.78 | 9.48E-03 | 6.87 | 7.70 | Yes |
| *LACTB2* | AK290201 | ENSG00000147592 | up | 1.78 | 3.66E-02 | 7.35 | 8.18 | No |
| *NLN* | AB033052 | ENSG00000123213 | up | 1.78 | 1.85E-02 | 7.04 | 7.86 | No |
| *NCAPG* | AF331796 | ENSG00000109805 | up | 1.77 | 4.49E-02 | 6.29 | 7.12 | Yes |
| *C20orf54* | BC009750 | ENSG00000101276 | up | 1.77 | 1.80E-02 | 6.95 | 7.77 | No |
| *EIF5B* | AB018284 | ENSG00000158417 | up | 1.77 | 1.37E-02 | 7.72 | 8.54 | No |
| *---* | CR608515 | --- | up | 1.77 | 2.15E-02 | 7.96 | 8.78 | Yes |
| *TP53* | K03199 | ENSG00000141510 | up | 1.77 | 8.46E-03 | 8.70 | 9.52 | No |
| *METTL1* | BC000550 | ENSG00000037897 | up | 1.77 | 3.92E-03 | 7.15 | 7.97 | No |
| *GINS4* | AK095334 | ENSG00000147536 | up | 1.77 | 7.12E-03 | 5.44 | 6.26 | No |
| *OCIAD2* | AK298305 | ENSG00000145247 | up | 1.77 | 2.73E-02 | 7.83 | 8.65 | No |
| *RWDD2B* | AK000056 | ENSG00000156253 | up | 1.76 | 2.91E-02 | 7.40 | 8.21 | No |
| *---* | AK123617 | --- | up | 1.76 | 3.62E-02 | 6.72 | 7.54 | No |
| *FASN* | U29344 | ENSG00000169710 | up | 1.76 | 1.17E-02 | 7.06 | 7.87 | No |
| *RPGRIP1L* | CR749645 | ENSG00000103494 | up | 1.76 | 1.31E-02 | 5.70 | 6.51 | No |
| *FOXQ1* | BC053850 | ENSG00000164379 | up | 1.76 | 6.31E-07 | 7.72 | 8.54 | Yes |
| *IMPDH2* | J04208 | ENSG00000178035 | up | 1.75 | 3.40E-02 | 9.21 | 10.02 | No |
| *NIT2* | AF260334 | ENSG00000114021 | up | 1.75 | 1.46E-03 | 9.19 | 10.00 | No |
| *RANGAP1* | AB058738 | ENSG00000100401 | up | 1.75 | 3.01E-04 | 8.11 | 8.92 | No |
| *DDX52* | BC041785 | ENSG00000141141 | up | 1.75 | 6.14E-03 | 7.19 | 8.00 | No |
| *TLCD1* | EU831834 | ENSG00000160606 | up | 1.75 | 7.98E-03 | 6.42 | 7.22 | No |
| *CIRH1A* | BX647265 | ENSG00000141076 | up | 1.75 | 3.56E-03 | 9.13 | 9.94 | No |
| *TTC8* | BX247959 | ENSG00000165533 | up | 1.75 | 4.76E-03 | 6.97 | 7.78 | No |
| *DPAGT1* | AK128572 | ENSG00000172269 | up | 1.75 | 4.24E-03 | 8.91 | 9.72 | No |
| *ACY3* | BC008689 | ENSG00000132744 | up | 1.75 | 4.45E-02 | 6.99 | 7.80 | No |
| *MRPL32* | AB049649 | ENSG00000106591 | up | 1.75 | 3.48E-02 | 7.84 | 8.64 | No |
| *RCC1 // SNHG3* | BC007300 | ENSG00000180198 // ENSG00000242125 | up | 1.75 | 4.58E-03 | 7.41 | 8.21 | No |
| *CXCL2* | CR617096 | ENSG00000081041 | up | 1.75 | 1.40E-04 | 7.82 | 8.63 | Yes |
| *PLK4* | AB006972 | ENSG00000142731 | up | 1.75 | 4.37E-02 | 6.10 | 6.91 | Yes |
| *NIPSNAP1* | AJ001258 | ENSG00000184117 | up | 1.74 | 1.24E-02 | 8.43 | 9.23 | No |
| *CNN2* | AK126391 | ENSG00000064666 | up | 1.74 | 3.58E-03 | 9.04 | 9.83 | No |
| *TEAD4* | AY101179 | ENSG00000197905 | up | 1.74 | 1.18E-02 | 7.09 | 7.89 | No |
| *MRPL11* | AK127603 | ENSG00000174547 | up | 1.74 | 4.93E-02 | 8.20 | 9.00 | No |
| *CDK5* | BC005115 | ENSG00000164885 | up | 1.74 | 2.49E-02 | 7.46 | 8.25 | No |
| *TRIP13* | BC019294 | ENSG00000071539 | up | 1.74 | 3.54E-02 | 7.37 | 8.18 | No |
| *---* | BC038563 | --- | up | 1.74 | 4.17E-02 | 4.73 | 5.52 | No |
| *INPP4B* | BX649090 | ENSG00000109452 | up | 1.74 | 1.26E-02 | 6.67 | 7.47 | No |
| *NAA15* | AJ314788 | ENSG00000164134 | up | 1.74 | 3.05E-02 | 7.73 | 8.53 | No |
| *EPHB2* | AB210018 | ENSG00000133216 | up | 1.74 | 1.71E-06 | 9.66 | 10.46 | No |
| *SCO1* | AK074588 | ENSG00000133028 | up | 1.73 | 1.07E-02 | 7.28 | 8.07 | No |
| *---* | CR595760 | --- | up | 1.73 | 2.30E-03 | 9.14 | 9.93 | Yes |
| *SLC29A3* | AX775921 | ENSG00000198246 | up | 1.73 | 2.04E-02 | 6.92 | 7.72 | No |
| *WBP5* | AF125535 | ENSG00000185222 | up | 1.73 | 1.63E-02 | 6.27 | 7.06 | No |
| *PHF19* | BX640713 | ENSG00000119403 | up | 1.73 | 1.16E-02 | 6.88 | 7.67 | No |
| *FXN* | BC023633 | ENSG00000165060 | up | 1.73 | 7.83E-11 | 8.37 | 9.16 | No |
| *HIST1H3F* | BC096131 | ENSG00000112727 | up | 1.73 | 4.26E-02 | 6.32 | 7.11 | No |
| *RPL7L1* | AK021904 | ENSG00000146223 | up | 1.73 | 1.39E-02 | 7.98 | 8.76 | No |
| *SOD3* | CR595031 | ENSG00000109610 | up | 1.72 | 4.01E-04 | 8.31 | 9.09 | No |
| *TACC3* | AK225631 | ENSG00000013810 | up | 1.72 | 2.34E-02 | 6.46 | 7.25 | No |
| *COMT* | AK130031 | ENSG00000093010 | up | 1.72 | 2.86E-03 | 8.79 | 9.58 | No |
| *CSE1L* | AF053641 | ENSG00000124207 | up | 1.72 | 3.74E-02 | 8.37 | 9.15 | No |
| *PNP* | BC106074 | ENSG00000198805 | up | 1.72 | 7.66E-03 | 9.09 | 9.87 | No |
| *RACGAP1P* | AF334184 | ENSG00000161800 | up | 1.72 | 2.59E-02 | 6.98 | 7.76 | Yes |
| *FIGF // PIR* | AX740234 | ENSG00000087842 // ENSG00000165197 | up | 1.72 | 2.49E-02 | 6.62 | 7.41 | No |
| *PSMD5* | BC014478 | ENSG00000095261 | up | 1.72 | 2.41E-02 | 7.84 | 8.62 | No |
| *NSUN5 // NSUN5P2* | CR601630 | ENSG00000106133 // ENSG00000130305 | up | 1.72 | 1.30E-02 | 7.47 | 8.25 | No |
| *MEST* | AK098397 | ENSG00000106484 | up | 1.72 | 2.40E-02 | 9.31 | 10.09 | No |
| *SRPK1* | BC038292 | ENSG00000096063 | up | 1.72 | 3.08E-02 | 9.10 | 9.89 | No |
| *NT5DC2* | AK023995 | ENSG00000168268 | up | 1.71 | 3.93E-02 | 8.14 | 8.91 | No |
| *PEX10* | DQ895151 | ENSG00000157911 | up | 1.71 | 7.76E-03 | 8.38 | 9.15 | No |
| *EXOSC5* | AF285785 | ENSG00000077348 | up | 1.71 | 1.74E-02 | 8.19 | 8.96 | No |
| *NUP88* | Y08612 | ENSG00000108559 | up | 1.71 | 9.34E-03 | 8.46 | 9.23 | No |
| *NQO1* | J03934 | ENSG00000181019 | up | 1.71 | 3.53E-02 | 10.31 | 11.09 | No |
| *DLGAP5* | AB076695 | ENSG00000126787 | up | 1.71 | 3.23E-02 | 6.79 | 7.57 | No |
| *TUBA1C* | AK293589 | ENSG00000167553 | up | 1.71 | 2.83E-04 | 8.97 | 9.74 | No |
| *DDX56* | AK125595 | ENSG00000136271 | up | 1.71 | 1.30E-02 | 8.64 | 9.41 | No |
| *HIST1H4E* | BC012587 | ENSG00000198518 | up | 1.71 | 4.44E-02 | 8.71 | 9.48 | Yes |
| *NOP14* | BC017358 | ENSG00000087269 | up | 1.71 | 1.18E-03 | 8.52 | 9.29 | No |
| *NBEAL2* | AK131104 | ENSG00000160796 | up | 1.70 | 1.23E-02 | 7.74 | 8.50 | No |
| *PIGP* | AK304656 | ENSG00000185808 | up | 1.70 | 1.43E-02 | 7.95 | 8.72 | No |
| *ITPA* | AF219116 | ENSG00000125877 | up | 1.70 | 4.60E-02 | 6.61 | 7.37 | No |
| *AMMECR1L* | BC078174 | ENSG00000144233 | up | 1.70 | 2.45E-02 | 6.76 | 7.52 | No |
| *FAM84A* | AX747684 | ENSG00000162981 | up | 1.70 | 3.78E-02 | 9.75 | 10.51 | No |
| *CIAPIN1* | AF248964 | ENSG00000005194 | up | 1.70 | 5.98E-03 | 8.00 | 8.77 | No |
| *CHID1* | AK301245 | ENSG00000177830 | up | 1.70 | 6.14E-03 | 8.58 | 9.35 | No |
| *TSTA3* | BC093061 | ENSG00000104522 | up | 1.70 | 9.34E-03 | 8.76 | 9.53 | No |
| *DCAF13* | AK027799 | ENSG00000164934 | up | 1.70 | 2.07E-02 | 6.77 | 7.53 | No |
| *MCM7* | AK226175 | ENSG00000166508 | up | 1.70 | 3.38E-02 | 8.47 | 9.24 | No |
| *CCT4* | AK303082 | ENSG00000115484 | up | 1.69 | 1.66E-02 | 8.26 | 9.02 | No |
| *AGMAT* | AK130034 | ENSG00000116771 | up | 1.69 | 2.90E-02 | 8.40 | 9.16 | No |
| *GRAMD1A* | AK097215 | --- | up | 1.69 | 3.00E-03 | 8.14 | 8.90 | No |
| *CDC6* | BC025232 | ENSG00000094804 | up | 1.69 | 4.96E-02 | 6.53 | 7.29 | No |
| *NUDT14* | AK128196 | ENSG00000183828 | up | 1.69 | 1.21E-02 | 7.05 | 7.80 | No |
| *PHB2* | BC110322 | ENSG00000215021 | up | 1.69 | 4.84E-03 | 9.90 | 10.66 | No |
| *CCDC86* | AK025974 | ENSG00000110104 | up | 1.69 | 4.58E-03 | 7.53 | 8.29 | No |
| *DDX21* | BX648405 | ENSG00000165732 | up | 1.69 | 2.68E-02 | 7.94 | 8.70 | No |
| *FZD6* | BX640609 | ENSG00000164930 | up | 1.69 | 2.80E-02 | 6.86 | 7.62 | No |
| *UBXN8* | D83767 | ENSG00000104691 | up | 1.69 | 1.79E-02 | 6.83 | 7.59 | No |
| *PSMC2* | AB075520 | ENSG00000161057 | up | 1.69 | 2.39E-02 | 8.46 | 9.22 | No |
| *ILF2* | CR593428 | ENSG00000143621 | up | 1.69 | 7.86E-05 | 10.50 | 11.26 | No |
| *HMGA1* | M23617 | ENSG00000137309 | up | 1.69 | 3.90E-08 | 9.63 | 10.39 | No |
| *C20orf3* | AB033767 | ENSG00000101474 | up | 1.68 | 1.12E-02 | 7.50 | 8.25 | No |
| *SNRPB* | BC003530 | ENSG00000125835 | up | 1.68 | 1.31E-02 | 10.71 | 11.46 | No |
| *TRMT61B* | BC000952 | ENSG00000171103 | up | 1.68 | 9.06E-03 | 6.43 | 7.18 | No |
| *RHOB* | AK309991 | ENSG00000143878 | up | 1.68 | 1.64E-02 | 7.32 | 8.07 | No |
| *GOT2* | AK098313 | ENSG00000125166 | up | 1.68 | 1.49E-02 | 10.16 | 10.90 | No |
| *PRDX5* | CR608340 | ENSG00000126432 | up | 1.68 | 1.10E-07 | 10.14 | 10.88 | Yes |
| *TEX10* | AB060968 | ENSG00000136891 | up | 1.68 | 4.93E-02 | 7.70 | 8.45 | No |
| *POLD2* | CR590132 | ENSG00000106628 | up | 1.68 | 2.75E-02 | 8.69 | 9.43 | No |
| *RNF39* | AF238315 | ENSG00000204618 | up | 1.68 | 3.72E-02 | 6.72 | 7.48 | No |
| *S100A11* | D38583 | ENSG00000163191 | up | 1.68 | 5.43E-08 | 11.06 | 11.80 | No |
| *NOP16* | AF151875 | ENSG00000048162 | up | 1.68 | 2.53E-02 | 7.42 | 8.17 | No |
| *SLC19A1* | AK310797 | ENSG00000173638 | up | 1.67 | 5.74E-03 | 8.38 | 9.12 | No |
| *METTL8* | AK024046 | ENSG00000123600 | up | 1.67 | 1.83E-02 | 6.40 | 7.14 | No |
| *USP14* | BC003556 | ENSG00000101557 | up | 1.67 | 7.88E-03 | 7.67 | 8.41 | No |
| *H2AFX* | CR605072 | ENSG00000188486 | up | 1.67 | 3.82E-02 | 8.57 | 9.31 | No |
| *KCTD14* | AK313892 | ENSG00000151364 | up | 1.67 | 4.05E-02 | 8.91 | 9.65 | No |
| *SSSCA1* | AB001740 | ENSG00000173465 | up | 1.67 | 3.21E-04 | 7.79 | 8.54 | No |
| *TM2D2* | AL834224 | ENSG00000169490 | up | 1.67 | 5.66E-03 | 6.77 | 7.51 | No |
| *ADAM32* | AK300148 | ENSG00000197140 | up | 1.67 | 3.98E-02 | 5.48 | 6.22 | No |
| *LIMK1* | D26309 | ENSG00000106683 | up | 1.67 | 3.53E-02 | 7.48 | 8.22 | No |
| *---* | AK022628 | --- | up | 1.67 | 1.76E-02 | 7.18 | 7.92 | No |
| *SLC35B2* | BC024288 | ENSG00000157593 | up | 1.67 | 1.35E-02 | 8.53 | 9.27 | No |
| *HIST1H2AB* | BC125140 | ENSG00000137259 | up | 1.67 | 3.29E-02 | 6.16 | 6.90 | Yes |
| *TIGD2* | AK027653 | ENSG00000180346 | up | 1.66 | 1.85E-02 | 5.93 | 6.66 | No |
| *C3orf26* | BC006512 | ENSG00000184220 | up | 1.66 | 4.04E-02 | 7.36 | 8.09 | No |
| *ZNRF3* | AB051436 | ENSG00000183579 | up | 1.66 | 3.91E-02 | 7.18 | 7.91 | No |
| *NOP56* | BC104793 | ENSG00000101361 | up | 1.66 | 3.90E-02 | 8.23 | 8.97 | No |
| *ZRANB3* | BX647838 | ENSG00000121988 | up | 1.66 | 3.07E-02 | 6.01 | 6.74 | No |
| *NOP58* | CR618609 | ENSG00000055044 | up | 1.66 | 4.45E-02 | 7.75 | 8.48 | Yes |
| *PPAP2C* | BC002806 | ENSG00000141934 | up | 1.66 | 2.80E-02 | 9.92 | 10.65 | No |
| *GRWD1* | AB075822 | ENSG00000105447 | up | 1.66 | 8.76E-04 | 7.62 | 8.36 | No |
| *SLC27A2* | AK290262 | ENSG00000140284 | up | 1.66 | 4.80E-02 | 7.70 | 8.44 | Yes |
| *EIF2S1* | BC002513 | ENSG00000134001 | up | 1.66 | 8.78E-03 | 8.41 | 9.14 | No |
| *MIPEP* | BC009934 | ENSG00000027001 | up | 1.66 | 1.39E-02 | 6.28 | 7.01 | No |
| *CDK4* | M14505 | ENSG00000135446 | up | 1.66 | 1.08E-02 | 9.65 | 10.38 | No |
| *DDX20* | BC034953 | ENSG00000064703 | up | 1.66 | 2.43E-02 | 7.03 | 7.76 | No |
| *UNC119B* | AK126367 | ENSG00000175970 | up | 1.66 | 3.52E-03 | 6.92 | 7.65 | No |
| *G6PD* | X03674 | ENSG00000160211 | up | 1.66 | 7.14E-03 | 7.52 | 8.26 | No |
| *C9orf100* | AB528966 | ENSG00000137135 | up | 1.66 | 4.28E-02 | 7.25 | 7.98 | No |
| *VARS* | AK300033 | ENSG00000204394 // ENSG00000224264 | up | 1.66 | 9.12E-03 | 8.04 | 8.77 | No |
| *PSMD6* | AY359879 | ENSG00000163636 | up | 1.65 | 2.07E-02 | 7.63 | 8.35 | No |
| *SETMAR* | AK307537 | ENSG00000170364 | up | 1.65 | 5.68E-03 | 7.05 | 7.77 | No |
| *IFFO2* | AK024480 | ENSG00000169991 | up | 1.65 | 1.42E-02 | 6.73 | 7.46 | No |
| *POFUT1* | AF375884 | ENSG00000101346 | up | 1.65 | 2.75E-02 | 7.53 | 8.25 | No |
| *STK16* | AK292694 | ENSG00000115661 | up | 1.65 | 2.43E-02 | 6.66 | 7.38 | No |
| *SCCPDH* | AF151807 | ENSG00000143653 | up | 1.65 | 1.62E-02 | 8.33 | 9.05 | No |
| *FBXO17 // SARS2* | AK301903 | ENSG00000104835 | up | 1.65 | 9.76E-03 | 7.34 | 8.06 | No |
| *TRIM28* | BC004978 | ENSG00000130726 | up | 1.65 | 1.50E-03 | 8.80 | 9.52 | No |
| *RNF187* | BC012758 | ENSG00000168159 | up | 1.65 | 3.26E-03 | 7.67 | 8.39 | No |
| *PHB* | CR619581 | ENSG00000167085 | up | 1.65 | 1.10E-02 | 10.57 | 11.29 | No |
| *RSL1D1* | AY154473 | ENSG00000171490 | up | 1.65 | 2.98E-03 | 9.52 | 10.25 | No |
| *WDR3* | AF083217 | ENSG00000065183 | up | 1.65 | 1.80E-02 | 6.46 | 7.19 | No |
| *PPM1H* | AB084258 | ENSG00000111110 | up | 1.65 | 2.95E-02 | 6.47 | 7.19 | No |
| *SHMT2* | BC091501 | ENSG00000182199 | up | 1.65 | 2.98E-03 | 9.37 | 10.09 | No |
| *WDR74* | AK292330 | ENSG00000133316 | up | 1.65 | 1.34E-02 | 8.58 | 9.30 | No |
| *---* | AK096908 | --- | up | 1.65 | 2.60E-02 | 4.95 | 5.67 | No |
| *RPSAP15* | AF284768 | --- | up | 1.65 | 4.01E-02 | 8.72 | 9.44 | Yes |
| *CTPS* | BC009408 | ENSG00000171793 | up | 1.65 | 3.75E-02 | 6.85 | 7.57 | Yes |
| *MRRF* | AK300718 | ENSG00000148187 | up | 1.65 | 1.83E-02 | 7.47 | 8.19 | No |
| *GPR172A* | AK027888 | ENSG00000185803 | up | 1.65 | 1.00E-02 | 7.43 | 8.16 | No |
| *---* | CR619849 | --- | up | 1.65 | 1.29E-02 | 9.07 | 9.80 | No |
| *---* | AK091117 | --- | up | 1.65 | 3.72E-02 | 5.89 | 6.61 | Yes |
| *SOX4* | AJ420500 | ENSG00000124766 | up | 1.65 | 2.68E-02 | 8.94 | 9.66 | No |
| *PIGX* | BC022542 | ENSG00000163964 | up | 1.64 | 1.22E-03 | 7.99 | 8.71 | No |
| *EXOSC7* | AK130053 | ENSG00000075914 | up | 1.64 | 1.34E-02 | 6.56 | 7.27 | No |
| *---* | BC029123 | --- | up | 1.64 | 9.80E-03 | 6.20 | 6.92 | No |
| *FAM64A* | CR615844 | ENSG00000129195 | up | 1.64 | 1.86E-02 | 6.91 | 7.62 | No |
| *MRP63* | BC068492 | ENSG00000173141 | up | 1.64 | 2.39E-02 | 7.11 | 7.83 | No |
| *VANGL1* | BC065272 | ENSG00000173218 | up | 1.64 | 1.39E-09 | 7.83 | 8.54 | No |
| *CCT2* | AF026166 | ENSG00000166226 | up | 1.64 | 7.22E-03 | 8.08 | 8.79 | No |
| *RNASEH2C* | CR602154 | ENSG00000172922 | up | 1.64 | 1.52E-03 | 8.21 | 8.92 | No |
| *DDB2* | U18300 | ENSG00000134574 | up | 1.64 | 2.36E-02 | 8.24 | 8.95 | No |
| *THNSL1* | BX647989 | ENSG00000185875 | up | 1.64 | 4.35E-02 | 6.31 | 7.02 | No |
| *---* | AK024840 | --- | up | 1.64 | 1.81E-02 | 7.62 | 8.33 | Yes |
| *LSM12* | BC044587 | ENSG00000161654 | up | 1.64 | 3.72E-03 | 10.20 | 10.92 | No |
| *TMEM9* | AY359012 | ENSG00000116857 | up | 1.64 | 1.79E-02 | 7.69 | 8.41 | No |
| *RPP40* | BC017871 | ENSG00000124787 | up | 1.64 | 3.02E-02 | 5.82 | 6.53 | No |
| *NHP2* | BC006387 | ENSG00000145912 | up | 1.64 | 4.99E-02 | 6.86 | 7.57 | No |
| *GFM2* | AF367997 | ENSG00000164347 | up | 1.64 | 1.16E-02 | 7.15 | 7.86 | No |
| *MRPS27* | AK025417 | ENSG00000113048 | up | 1.64 | 8.10E-03 | 8.26 | 8.97 | No |
| *WARS2* | BC044575 | ENSG00000116874 | up | 1.64 | 4.40E-02 | 6.73 | 7.44 | No |
| *C4orf43* | CR595986 | ENSG00000198498 | up | 1.64 | 8.92E-03 | 6.00 | 6.71 | No |
| *TTLL4* | AK302728 | ENSG00000135912 | up | 1.63 | 2.58E-02 | 7.26 | 7.96 | No |
| *GPR39* | AK122643 | ENSG00000183840 | up | 1.63 | 4.58E-02 | 7.21 | 7.92 | No |
| *CAD* | BC065510 | ENSG00000084774 | up | 1.63 | 2.63E-02 | 6.87 | 7.58 | No |
| *PNPO* | AK223242 | ENSG00000108439 | up | 1.63 | 1.76E-02 | 8.76 | 9.47 | No |
| *TUBG1* | BC000619 | ENSG00000131462 | up | 1.63 | 1.30E-03 | 9.72 | 10.42 | No |
| *GPX2* | BC046164 | ENSG00000176153 | up | 1.63 | 4.02E-03 | 11.66 | 12.37 | No |
| *CDC16* | BC017244 | ENSG00000130177 | up | 1.63 | 6.56E-03 | 7.75 | 8.45 | No |
| *PRKAB1* | AF022116 | ENSG00000111725 | up | 1.63 | 2.62E-02 | 9.86 | 10.57 | No |
| *NOLC1* | BC006769 | ENSG00000166197 | up | 1.63 | 1.33E-07 | 8.36 | 9.07 | No |
| *EXOSC4* | AF281133 | ENSG00000178896 | up | 1.63 | 8.96E-03 | 8.10 | 8.80 | No |
| *UBE2V2* | AF049140 | ENSG00000169139 | up | 1.63 | 1.51E-02 | 7.16 | 7.87 | No |
| *KIAA1549* | AM989469 | ENSG00000122778 | up | 1.63 | 7.68E-03 | 6.75 | 7.46 | No |
| *C6orf108* | EU585603 | ENSG00000112667 | up | 1.63 | 1.41E-02 | 9.92 | 10.63 | No |
| *CDKN1A* | U09579 | ENSG00000124762 | up | 1.63 | 1.62E-06 | 9.34 | 10.05 | Yes |
| *CYFIP2* | AL136549 | ENSG00000055163 | up | 1.63 | 1.36E-02 | 8.00 | 8.70 | No |
| *PHTF1* | AJ011863 | ENSG00000116793 | up | 1.63 | 3.65E-02 | 6.09 | 6.80 | No |
| *PSMA5* | AK304448 | ENSG00000143106 | up | 1.62 | 4.31E-02 | 7.51 | 8.21 | No |
| *C4orf42* | AK125775 | ENSG00000196810 | up | 1.62 | 3.96E-03 | 7.04 | 7.74 | No |
| *KTI12* | AF327348 | ENSG00000198841 | up | 1.62 | 1.28E-02 | 7.45 | 8.14 | No |
| *TMEM161A* | AK096964 | ENSG00000064545 | up | 1.62 | 6.46E-03 | 7.58 | 8.28 | No |
| *VPS25* | BC006282 | ENSG00000131475 | up | 1.62 | 1.22E-02 | 9.08 | 9.77 | No |
| *C17orf81* | BC002762 | ENSG00000170291 | up | 1.62 | 1.74E-02 | 8.82 | 9.52 | No |
| *---* | BC035106 | --- | up | 1.62 | 2.81E-02 | 5.68 | 6.38 | No |
| *SDSL* | BC009849 | ENSG00000139410 | up | 1.62 | 5.30E-03 | 7.42 | 8.12 | No |
| *NUDT8* | BC018644 | ENSG00000167799 | up | 1.62 | 1.77E-02 | 6.82 | 7.51 | No |
| *EIF5AL1* | BC070048 | ENSG00000132507 | up | 1.62 | 3.22E-03 | 9.04 | 9.73 | Yes |
| *DYNLT3* | U02556 | ENSG00000165169 | up | 1.62 | 4.89E-02 | 6.85 | 7.54 | No |
| *PLS3* | BC056898 | ENSG00000102024 | up | 1.62 | 3.44E-02 | 8.07 | 8.77 | No |
| *DDX31* | AF427339 | ENSG00000125485 | up | 1.62 | 1.83E-02 | 6.69 | 7.39 | No |
| *RHEB* | AK125446 | ENSG00000106615 | up | 1.62 | 4.75E-02 | 7.92 | 8.62 | No |
| *POT1* | AK302263 | ENSG00000128513 | up | 1.62 | 4.91E-02 | 6.23 | 6.92 | No |
| *MOSPD3* | CR609640 | ENSG00000106330 | up | 1.62 | 4.97E-02 | 7.80 | 8.49 | Yes |
| *FAM189B* | BC008854 | ENSG00000160767 | up | 1.62 | 6.64E-03 | 6.52 | 7.21 | No |
| *POLH* | AK316151 | ENSG00000170734 | up | 1.62 | 3.74E-02 | 7.61 | 8.31 | No |
| *PRELID1* | BC018904 | ENSG00000169230 | up | 1.62 | 1.66E-03 | 10.18 | 10.87 | Yes |
| *CSNK1E* | AK092269 | ENSG00000213923 | up | 1.61 | 9.43E-04 | 9.49 | 10.18 | No |
| *DUSP18* | BC030987 | ENSG00000167065 | up | 1.61 | 1.53E-02 | 7.45 | 8.14 | No |
| *---* | AK297038 | --- | up | 1.61 | 4.18E-02 | 7.24 | 7.93 | No |
| *MAPRE3* | AB025186 | ENSG00000084764 | up | 1.61 | 4.30E-02 | 6.72 | 7.40 | No |
| *C19orf48* | CR591122 | ENSG00000167747 | up | 1.61 | 5.84E-07 | 7.47 | 8.16 | No |
| *LRRC8E* | BC070089 | ENSG00000171017 | up | 1.61 | 1.00E-02 | 6.37 | 7.05 | No |
| *GLOD4* | AF151908 | ENSG00000167699 | up | 1.61 | 2.82E-03 | 7.16 | 7.85 | No |
| *POLR2C* | BC003159 | ENSG00000102978 | up | 1.61 | 5.76E-04 | 7.89 | 8.58 | No |
| *RPAP1* | AK022794 | ENSG00000103932 | up | 1.61 | 3.12E-03 | 7.13 | 7.81 | No |
| *NUDT4 // NUDT4P1* | AF191652 | ENSG00000173598 | up | 1.61 | 5.74E-03 | 10.58 | 11.27 | No |
| *SPIN4* | AL833314 | ENSG00000186767 | up | 1.61 | 7.80E-03 | 7.44 | 8.13 | No |
| *PPP2R4* | DQ895349 | ENSG00000119383 | up | 1.61 | 7.46E-03 | 7.97 | 8.66 | No |
| *B3GALNT2* | BC029564 | ENSG00000162885 | up | 1.61 | 3.40E-03 | 7.26 | 7.94 | No |
| *ZNF703* | BC084581 | ENSG00000183779 | up | 1.61 | 4.23E-02 | 8.88 | 9.56 | Yes |
| *AGK* | AK001704 | ENSG00000006530 | up | 1.61 | 3.80E-02 | 6.83 | 7.52 | No |
| *PDCD2* | S78085 | ENSG00000071994 | up | 1.61 | 2.68E-02 | 7.41 | 8.10 | No |
| *TBC1D14* | BC041167 | ENSG00000132405 | up | 1.60 | 1.20E-02 | 7.89 | 8.57 | No |
| *---* | AK126297 | --- | up | 1.60 | 2.47E-02 | 6.64 | 7.32 | No |
| *DUSP14* | CR598453 | ENSG00000161326 | up | 1.60 | 4.78E-02 | 6.04 | 6.72 | No |
| *CSNK2A2* | M55268 | ENSG00000070770 | up | 1.60 | 3.86E-03 | 9.06 | 9.73 | No |
| *C15orf41* | BC006254 | ENSG00000186073 | up | 1.60 | 5.92E-08 | 7.42 | 8.10 | No |
| *C12orf66* | BC036246 | ENSG00000174206 | up | 1.60 | 1.39E-02 | 6.56 | 7.23 | No |
| *CYB561D1* | BX647509 | ENSG00000174151 | up | 1.60 | 2.50E-03 | 6.23 | 6.92 | No |
| *USP5* | U47927 | ENSG00000111667 | up | 1.60 | 7.59E-04 | 7.63 | 8.31 | No |
| *RBMX* | BX647131 | ENSG00000147274 | up | 1.60 | 2.23E-02 | 8.93 | 9.61 | Yes |
| *DKC1* | BC009928 | ENSG00000130826 | up | 1.60 | 3.65E-02 | 7.74 | 8.43 | No |
| *POLE3* | AK074629 | ENSG00000148229 | up | 1.60 | 9.62E-03 | 8.28 | 8.95 | No |
| *TMEM2* | AF137030 | ENSG00000135048 | up | 1.60 | 2.50E-02 | 7.99 | 8.66 | No |
| *PLAA* | AL133608 | ENSG00000137055 | up | 1.60 | 1.42E-02 | 7.91 | 8.58 | No |
| *---* | AK090578 | --- | up | 1.60 | 3.76E-02 | 6.66 | 7.34 | No |
| *RPS2P32* | BC026177 | ENSG00000232818 | up | 1.60 | 2.06E-03 | 7.24 | 7.92 | No |
| *ZNRD1* | BC051741 | ENSG00000066379 // ENSG00000206502 | up | 1.60 | 1.94E-02 | 7.98 | 8.66 | No |
| *FUCA2* | AY358551 | ENSG00000001036 | up | 1.60 | 9.28E-03 | 8.19 | 8.87 | No |
| *CCT3* | AK126400 | ENSG00000163468 | up | 1.60 | 7.00E-03 | 8.93 | 9.61 | No |
| *TMEM184B* | BC015489 | ENSG00000198792 | up | 1.59 | 1.90E-03 | 8.82 | 9.49 | No |
| *MRPL39* | BC107719 | ENSG00000154719 | up | 1.59 | 1.12E-02 | 8.18 | 8.85 | No |
| *ADCY3* | AK122926 | ENSG00000138031 | up | 1.59 | 3.95E-02 | 7.78 | 8.45 | No |
| *URB2* | D50923 | ENSG00000135763 | up | 1.59 | 3.28E-02 | 6.38 | 7.05 | No |
| *SOCS7* | BC128607 | ENSG00000174111 | up | 1.59 | 8.20E-03 | 6.80 | 7.47 | No |
| *DARS2* | BC045173 | ENSG00000117593 | up | 1.59 | 1.01E-02 | 7.51 | 8.17 | No |
| *G2E3* | AB037754 | ENSG00000092140 | up | 1.59 | 7.74E-03 | 6.22 | 6.89 | No |
| *IDE* | BX648462 | ENSG00000119912 | up | 1.59 | 9.46E-03 | 8.24 | 8.91 | No |
| *HSD17B10* | U96132 | ENSG00000072506 | up | 1.59 | 6.36E-03 | 7.87 | 8.54 | No |
| *C9orf69* | BC021231 | ENSG00000238227 | up | 1.59 | 4.56E-03 | 7.28 | 7.95 | No |
| *GSTCD* | BX648355 | ENSG00000138780 | up | 1.58 | 2.56E-02 | 6.33 | 6.99 | No |
| *CXCL1* | BC011976 | ENSG00000163739 | up | 1.58 | 7.18E-03 | 8.47 | 9.13 | No |
| *RRP9* | BC021032 | ENSG00000114767 | up | 1.58 | 1.24E-02 | 7.66 | 8.32 | No |
| *ODC1* | CR614398 | ENSG00000115758 | up | 1.58 | 7.81E-07 | 9.94 | 10.60 | No |
| *SRM* | CR590206 | ENSG00000116649 | up | 1.58 | 2.14E-07 | 9.42 | 10.09 | No |
| *PRMT1* | CR622298 | ENSG00000126457 | up | 1.58 | 6.90E-03 | 8.86 | 9.52 | No |
| *---* | CR611601 | --- | up | 1.58 | 1.70E-02 | 6.95 | 7.61 | No |
| *SLC43A2* | BC027923 | ENSG00000167703 | up | 1.58 | 3.74E-02 | 8.23 | 8.89 | No |
| *RNF167* | CR617000 | ENSG00000108523 | up | 1.58 | 1.52E-02 | 8.22 | 8.87 | No |
| *NUP93* | D42085 | ENSG00000102900 | up | 1.58 | 5.78E-03 | 8.19 | 8.84 | No |
| *OGFOD1* | AB046832 | ENSG00000087263 | up | 1.58 | 2.56E-03 | 7.69 | 8.35 | No |
| *C15orf44* | AK027475 | ENSG00000138614 | up | 1.58 | 2.35E-02 | 8.33 | 8.99 | No |
| *RAB25* | AF274025 | ENSG00000132698 | up | 1.58 | 2.49E-02 | 9.90 | 10.56 | No |
| *---* | AK055641 | --- | up | 1.58 | 3.48E-02 | 7.94 | 8.60 | No |
| *ORMDL2* | CR621685 | ENSG00000123353 | up | 1.58 | 1.74E-02 | 9.52 | 10.18 | No |
| *MRPS2* | CR618613 | ENSG00000122140 | up | 1.58 | 1.99E-02 | 9.44 | 10.10 | No |
| *BCAS2 // DENND2C* | BC063894 | ENSG00000116752 // ENSG00000175984 | up | 1.58 | 2.19E-02 | 7.49 | 8.15 | No |
| *PGM2* | AF109360 | ENSG00000169299 | up | 1.57 | 8.10E-03 | 8.03 | 8.69 | No |
| *SCLY* | AB209458 | ENSG00000132330 | up | 1.57 | 2.90E-02 | 7.87 | 8.53 | No |
| *STK36* | AB033104 | ENSG00000163482 | up | 1.57 | 7.84E-03 | 6.63 | 7.28 | No |
| *ATIC* | CR606023 | ENSG00000138363 | up | 1.57 | 1.85E-02 | 8.47 | 9.12 | No |
| *KAT2A* | BC039907 | ENSG00000108773 | up | 1.57 | 2.37E-02 | 7.90 | 8.55 | No |
| *YWHAE* | AK128785 | ENSG00000108953 | up | 1.57 | 1.64E-03 | 10.33 | 10.99 | No |
| *SIPA1L1* | AF090990 | ENSG00000197555 | up | 1.57 | 2.72E-03 | 8.75 | 9.40 | No |
| *PRIM1* | BC005266 | ENSG00000198056 | up | 1.57 | 1.50E-02 | 6.86 | 7.51 | No |
| *RACGAP1* | AL136794 | ENSG00000161800 | up | 1.57 | 4.52E-02 | 7.88 | 8.53 | Yes |
| *PEX5* | AK292256 | ENSG00000139197 | up | 1.57 | 7.76E-03 | 7.05 | 7.69 | No |
| *DPH2* | BC003181 | ENSG00000132768 | up | 1.57 | 1.42E-02 | 7.27 | 7.93 | No |
| *ADAP1 // COX19* | AK290517 | ENSG00000105963 // ENSG00000240230 | up | 1.57 | 3.82E-02 | 8.23 | 8.88 | No |
| *SH3PXD2B* | BC038561 | ENSG00000174705 | up | 1.57 | 2.22E-02 | 7.13 | 7.78 | No |
| *CLOCK* | BC126159 | ENSG00000134852 | up | 1.57 | 4.55E-02 | 8.04 | 8.70 | Yes |
| *---* | AK123776 | --- | up | 1.56 | 4.52E-02 | 7.60 | 8.25 | Yes |
| *UMPS* | J03626 | ENSG00000114491 | up | 1.56 | 3.24E-02 | 7.21 | 7.85 | No |
| *ABHD14A // ACY1* | AK298086 | ENSG00000114786 // ENSG00000243989 | up | 1.56 | 1.12E-02 | 8.64 | 9.28 | No |
| *BCL2L1* | BX647525 | ENSG00000171552 | up | 1.56 | 2.19E-02 | 8.91 | 9.55 | No |
| *STRA13* | U95007 | ENSG00000169689 | up | 1.56 | 1.89E-02 | 7.79 | 8.43 | Yes |
| *TRAPPC1* | AK314038 | ENSG00000170043 | up | 1.56 | 5.22E-03 | 9.90 | 10.54 | No |
| *DPH1 // OVCA2* | U34880 | ENSG00000108963 // ENSG00000214014 | up | 1.56 | 5.10E-03 | 7.41 | 8.05 | No |
| *RNMTL1* | AF177344 | ENSG00000171861 | up | 1.56 | 4.00E-03 | 6.26 | 6.90 | No |
| *H6PD* | BC081559 | ENSG00000049239 | up | 1.56 | 8.60E-04 | 7.68 | 8.32 | No |
| *GPC4* | AF030186 | ENSG00000076716 | up | 1.56 | 9.60E-03 | 7.33 | 7.98 | No |
| *INTS9* | AK225530 | ENSG00000104299 | up | 1.56 | 1.22E-02 | 7.01 | 7.65 | No |
| *PMS2* | BX537558 | ENSG00000122512 | up | 1.56 | 4.96E-02 | 6.61 | 7.25 | No |
| *POP7* | BC001430 | ENSG00000172336 | up | 1.56 | 1.56E-02 | 7.30 | 7.94 | No |
| *UBE2D4* | AK001446 | ENSG00000078967 | up | 1.56 | 8.74E-03 | 7.98 | 8.62 | No |
| *HCP5* | CR626077 | ENSG00000237105 | up | 1.56 | 4.78E-02 | 8.46 | 9.10 | No |
| *PF4* | M25897 | ENSG00000163737 | up | 1.56 | 4.76E-02 | 6.27 | 6.91 | No |
| *COX18* | AY957564 | ENSG00000163626 | up | 1.56 | 1.30E-02 | 7.99 | 8.64 | No |
| *ABHD14B* | BC071931 | ENSG00000114779 | up | 1.55 | 4.28E-02 | 8.50 | 9.14 | No |
| *PPPDE2* | CR605156 | ENSG00000100418 | up | 1.55 | 7.16E-03 | 9.45 | 10.08 | No |
| *C21orf70* | AF391114 | ENSG00000160256 | up | 1.55 | 4.40E-03 | 7.39 | 8.03 | No |
| *C20orf96* | AK097394 | ENSG00000196476 | up | 1.55 | 2.20E-02 | 6.35 | 6.99 | No |
| *CCT7* | BC088351 | ENSG00000135624 | up | 1.55 | 1.22E-02 | 9.33 | 9.96 | No |
| *TIMM50* | BC121147 | ENSG00000105197 | up | 1.55 | 9.74E-03 | 9.47 | 10.10 | No |
| *TMEM147* | CR601646 | ENSG00000105677 | up | 1.55 | 6.08E-03 | 9.93 | 10.57 | No |
| *PALB2* | BX647130 | ENSG00000083093 | up | 1.55 | 4.88E-02 | 7.21 | 7.83 | No |
| *TPRG1L* | AK096427 | ENSG00000158109 | up | 1.55 | 2.32E-02 | 8.49 | 9.12 | No |
| *PGM2L1* | AB019210 | ENSG00000165434 | up | 1.55 | 4.11E-02 | 6.67 | 7.30 | No |
| *FEN1* | BC000323 | ENSG00000168496 | up | 1.55 | 3.30E-02 | 7.07 | 7.71 | No |
| *C9orf114* | AL110193 | ENSG00000198917 | up | 1.55 | 7.70E-03 | 8.82 | 9.45 | No |
| *C9orf167* | AK000252 | ENSG00000198113 | up | 1.55 | 2.40E-02 | 7.06 | 7.70 | No |
| *METTL11A* | AK298840 | ENSG00000148335 | up | 1.55 | 2.04E-02 | 7.84 | 8.47 | No |
| *URM1* | CR594010 | ENSG00000167118 | up | 1.55 | 1.07E-02 | 7.62 | 8.25 | No |
| *---* | CR606158 | --- | up | 1.55 | 3.88E-02 | 7.29 | 7.92 | Yes |
| *MCM3* | D38073 | ENSG00000112118 | up | 1.55 | 3.81E-02 | 8.04 | 8.68 | No |
| *ACAD9* | BC007970 | ENSG00000177646 | up | 1.54 | 9.78E-03 | 8.63 | 9.25 | No |
| *RRP1* | BC000380 | ENSG00000160214 | up | 1.54 | 3.80E-02 | 7.29 | 7.92 | No |
| *ETS2* | AK096841 | ENSG00000157557 | up | 1.54 | 1.47E-02 | 10.82 | 11.43 | No |
| *HOXB5* | M92299 | ENSG00000120075 | up | 1.54 | 2.39E-05 | 6.98 | 7.61 | No |
| *POLDIP2* | BC018864 | ENSG00000004142 | up | 1.54 | 1.20E-02 | 9.66 | 10.29 | No |
| *PSMB5* | AK225888 | ENSG00000100804 | up | 1.54 | 1.28E-02 | 8.71 | 9.33 | No |
| *TSPAN8* | BC070168 | ENSG00000127324 | up | 1.54 | 4.07E-02 | 10.73 | 11.36 | No |
| *ALG8* | AK098614 | ENSG00000159063 | up | 1.54 | 3.47E-02 | 8.71 | 9.32 | No |
| *FOXRED1* | AK298807 | ENSG00000110074 | up | 1.54 | 2.52E-02 | 7.37 | 7.99 | No |
| *PDCD11* | BC172442 | ENSG00000148843 | up | 1.54 | 2.71E-02 | 7.21 | 7.84 | No |
| *HKDC1* | AK095086 | ENSG00000156510 | up | 1.54 | 2.12E-06 | 6.38 | 7.01 | Yes |
| *ZNF485* | AK074679 | ENSG00000198298 | up | 1.54 | 2.26E-02 | 6.30 | 6.93 | No |
| *NUDCD1* | BC043406 | ENSG00000120526 | up | 1.54 | 4.91E-02 | 6.76 | 7.38 | No |
| *MRPL15* | BC000891 | ENSG00000137547 | up | 1.54 | 4.78E-02 | 7.47 | 8.09 | No |
| *SLC4A2* | AB209158 | ENSG00000164889 | up | 1.54 | 1.41E-02 | 7.97 | 8.59 | No |
| *PMPCB* | AK297354 | ENSG00000105819 | up | 1.54 | 1.82E-02 | 8.33 | 8.95 | No |
| *---* | EF565086 | --- | up | 1.54 | 3.46E-02 | 6.03 | 6.65 | No |
| *TBCK* | AK074305 | ENSG00000145348 | up | 1.54 | 2.63E-02 | 7.70 | 8.32 | No |
| *GRPEL1* | BC024242 | ENSG00000109519 | up | 1.54 | 1.09E-02 | 7.89 | 8.51 | No |
| *WDR5B* | BC043494 | ENSG00000196981 | up | 1.53 | 1.31E-02 | 6.44 | 7.04 | No |
| *WDR4* | AJ243912 | ENSG00000160193 | up | 1.53 | 2.74E-02 | 6.93 | 7.54 | No |
| *ZMYND8* | BC146802 | ENSG00000101040 | up | 1.53 | 4.25E-02 | 8.01 | 8.63 | No |
| *DTYMK* | CR599450 | ENSG00000168393 | up | 1.53 | 4.67E-02 | 7.61 | 8.22 | No |
| *TMEM185B* | AF530474 | ENSG00000226479 | up | 1.53 | 1.44E-02 | 7.39 | 8.00 | No |
| *TMEM177* | AK057313 | ENSG00000144120 | up | 1.53 | 4.48E-02 | 7.25 | 7.86 | No |
| *SMYD5* | BC073806 | ENSG00000135632 | up | 1.53 | 1.74E-02 | 7.00 | 7.61 | No |
| *NDUFA7* | BC003102 | ENSG00000167774 | up | 1.53 | 6.70E-03 | 8.73 | 9.35 | Yes |
| *SLC39A3* | AK302600 | ENSG00000141873 | up | 1.53 | 2.70E-02 | 7.41 | 8.03 | Yes |
| *---* | AX747919 | --- | up | 1.53 | 3.62E-02 | 6.74 | 7.36 | No |
| *---* | AX746718 | --- | up | 1.53 | 4.33E-02 | 10.01 | 10.63 | No |
| *SHCBP1* | BC030699 | ENSG00000171241 | up | 1.53 | 3.59E-02 | 6.06 | 6.67 | No |
| *PTPLAD1* | AK074898 | ENSG00000074696 | up | 1.53 | 4.16E-02 | 8.16 | 8.77 | No |
| *PRMT5* | AF167572 | ENSG00000100462 | up | 1.53 | 5.02E-03 | 9.07 | 9.68 | No |
| *UCHL3* | CR610104 | ENSG00000118939 | up | 1.53 | 2.12E-02 | 8.34 | 8.95 | No |
| *PWP1* | AK297961 | ENSG00000136045 | up | 1.53 | 4.84E-03 | 7.54 | 8.15 | No |
| *C12orf10* | CR626228 | ENSG00000139637 | up | 1.53 | 1.00E-02 | 8.21 | 8.83 | No |
| *---* | M30627 | --- | up | 1.53 | 1.89E-02 | 9.30 | 9.91 | Yes |
| *PGAP2* | AL096753 | --- | up | 1.53 | 1.25E-08 | 8.80 | 9.42 | No |
| *SHROOM4* | BC151240 | ENSG00000158352 | up | 1.53 | 1.32E-02 | 6.69 | 7.31 | No |
| *RPL9* | CR605134 | ENSG00000163682 | up | 1.53 | 4.34E-02 | 6.27 | 6.89 | No |
| *SUV39H1* | AF019968 | ENSG00000101945 | up | 1.53 | 1.70E-02 | 6.61 | 7.22 | No |
| *KIAA1797* | AY139834 | ENSG00000188352 | up | 1.53 | 3.96E-02 | 6.79 | 7.41 | No |
| *EPHB4* | BC052804 | ENSG00000196411 | up | 1.53 | 1.25E-02 | 7.30 | 7.91 | No |
| *CHCHD2* | AF078845 | ENSG00000106153 | up | 1.53 | 4.10E-02 | 8.70 | 9.31 | No |
| *EEPD1* | AK027386 | ENSG00000122547 | up | 1.53 | 3.74E-02 | 6.98 | 7.59 | No |
| *TTYH3* | AB051478 | ENSG00000136295 | up | 1.53 | 1.55E-02 | 9.22 | 9.84 | Yes |
| *---* | AF088011 | --- | up | 1.52 | 1.78E-02 | 5.34 | 5.94 | No |
| *LARS2* | BC025989 | ENSG00000011376 | up | 1.52 | 1.20E-02 | 6.67 | 7.28 | No |
| *YDJC* | CR600451 | ENSG00000161179 | up | 1.52 | 1.92E-02 | 7.95 | 8.56 | No |
| *---* | AK124515 | --- | up | 1.52 | 2.40E-03 | 6.81 | 7.41 | Yes |
| *URB1* | AF231919 | ENSG00000142207 | up | 1.52 | 3.11E-02 | 7.01 | 7.61 | Yes |
| *EIF6* | AF047433 | ENSG00000242372 | up | 1.52 | 4.42E-02 | 10.07 | 10.67 | No |
| *SYMPK* | AK130602 | ENSG00000125755 | up | 1.52 | 5.82E-03 | 8.08 | 8.69 | No |
| *LRG1 // PLIN5* | DQ839131 | ENSG00000171236 // ENSG00000214456 | up | 1.52 | 3.28E-03 | 7.72 | 8.32 | No |
| *C18orf1* | AF009425 | ENSG00000168675 | up | 1.52 | 4.86E-02 | 7.64 | 8.25 | No |
| *EIF4A3* | CR602794 | ENSG00000141543 | up | 1.52 | 1.42E-02 | 9.22 | 9.82 | No |
| *FDXR* | BC011521 | ENSG00000161513 | up | 1.52 | 1.55E-02 | 7.23 | 7.83 | No |
| *GINS3* | BC005879 | ENSG00000181938 | up | 1.52 | 2.15E-02 | 6.55 | 7.16 | No |
| *FAM82A2* | AK123282 | ENSG00000137824 | up | 1.52 | 4.46E-02 | 8.41 | 9.02 | Yes |
| *GNPNAT1* | AK090577 | ENSG00000100522 | up | 1.52 | 4.42E-02 | 8.37 | 8.97 | Yes |
| *C12orf44* | AK021835 | ENSG00000123395 | up | 1.52 | 4.63E-02 | 7.35 | 7.96 | No |
| *SPATS2* | AL833614 | ENSG00000123352 | up | 1.52 | 2.07E-02 | 7.53 | 8.14 | No |
| *TALDO1* | BC009680 | ENSG00000177156 | up | 1.52 | 1.80E-04 | 8.70 | 9.30 | No |
| *NONO* | BC002364 | ENSG00000147140 | up | 1.52 | 3.60E-03 | 10.66 | 11.26 | No |
| *TBC1D2* | AK124772 | ENSG00000095383 | up | 1.52 | 5.16E-08 | 7.94 | 8.55 | No |
| *UBAP2* | AK302754 | ENSG00000137073 | up | 1.52 | 4.96E-02 | 8.73 | 9.34 | No |
| *NCRNA00094* | AK092667 | ENSG00000235106 | up | 1.52 | 2.77E-02 | 6.17 | 6.77 | No |
| *---* | AY359878 | --- | up | 1.52 | 1.95E-02 | 10.39 | 10.99 | Yes |
| *SLC29A1* | CR593933 | ENSG00000112759 | up | 1.52 | 7.52E-09 | 8.62 | 9.22 | No |
| *QDPR* | AK296622 | ENSG00000151552 | up | 1.52 | 2.83E-02 | 6.45 | 7.06 | No |
| *STX18* | AK310026 | ENSG00000168818 | up | 1.52 | 3.04E-03 | 8.34 | 8.95 | No |
| *POMGNT1* | AK056186 | ENSG00000085998 | up | 1.51 | 1.78E-02 | 7.77 | 8.37 | No |
| *UBE2S* | CR594048 | ENSG00000108106 | up | 1.51 | 1.34E-06 | 6.87 | 7.47 | Yes |
| *RUVBL2* | CR613453 | ENSG00000183207 | up | 1.51 | 1.74E-02 | 8.14 | 8.73 | No |
| *ZNF146* | BC065568 | ENSG00000167635 | up | 1.51 | 4.59E-02 | 8.21 | 8.81 | No |
| *C17orf49 // RNASEK* | CR600192 | ENSG00000161939 // ENSG00000219200 | up | 1.51 | 1.02E-02 | 9.58 | 10.17 | Yes |
| *C16orf80* | AF093680 | ENSG00000070761 | up | 1.51 | 9.14E-03 | 8.10 | 8.70 | No |
| *MTERFD3* | BC025984 | ENSG00000120832 | up | 1.51 | 4.47E-02 | 6.47 | 7.07 | No |
| *DCLRE1B* | CR936679 | ENSG00000118655 | up | 1.51 | 1.53E-02 | 7.28 | 7.88 | No |
| *NOP2* | AK307582 | ENSG00000111641 | up | 1.51 | 9.56E-03 | 7.76 | 8.35 | No |
| *NEU3* | AK290442 | ENSG00000162139 | up | 1.51 | 1.28E-02 | 7.16 | 7.75 | No |
| *BANF1* | AF068235 | ENSG00000175334 | up | 1.51 | 1.91E-02 | 9.87 | 10.47 | No |
| *WDR34* | CR596639 | ENSG00000119333 | up | 1.51 | 4.73E-02 | 8.47 | 9.06 | No |
| *TRUB2* | CR622527 | ENSG00000167112 | up | 1.51 | 2.25E-02 | 8.36 | 8.96 | Yes |
| *EXOSC2* | AK022460 | ENSG00000130713 | up | 1.51 | 4.17E-02 | 8.69 | 9.28 | No |
| *---* | BC085016 | --- | up | 1.51 | 2.68E-02 | 7.58 | 8.18 | Yes |
| *ERGIC3* | EF206690 | ENSG00000125991 | up | 1.50 | 2.74E-02 | 10.00 | 10.59 | No |
| *VWA1* | AK075366 | ENSG00000179403 | up | 1.50 | 9.36E-10 | 7.89 | 8.48 | Yes |
| *RWDD4A* | BC017472 | ENSG00000182552 | up | 1.50 | 4.19E-02 | 5.98 | 6.57 | No |
| *CCNC* | BC010135 | ENSG00000112237 | up | 1.50 | 4.29E-02 | 8.72 | 9.31 | No |
| *CA1* | M33987 | ENSG00000133742 | down | 41.42 | 2.60E-03 | 12.42 | 7.04 | No |
| *---* | BC038540 | --- | down | 34.22 | 1.21E-04 | 10.32 | 5.22 | No |
| *GCG* | BC005278 | ENSG00000115263 | down | 27.59 | 4.00E-06 | 10.31 | 5.52 | No |
| *---* | AK297065 | --- | down | 24.88 | 2.58E-03 | 10.89 | 6.25 | No |
| *MS4A12* | AK000224 | ENSG00000071203 | down | 24.59 | 7.63E-04 | 10.55 | 5.93 | No |
| *CLCA4* | AK000072 | ENSG00000016602 | down | 23.99 | 3.16E-04 | 10.81 | 6.23 | No |
| *CD177* | CR592446 | ENSG00000204936 | down | 22.68 | 3.02E-04 | 11.63 | 7.13 | No |
| *GUCA2B* | U34279 | ENSG00000044012 | down | 19.82 | 1.62E-12 | 10.03 | 5.72 | No |
| *OTOP2* | BC152986 | ENSG00000183034 | down | 19.34 | 4.02E-13 | 9.86 | 5.59 | No |
| *GUCA2A* | M97496 | ENSG00000197273 | down | 18.57 | 1.51E-05 | 11.11 | 6.89 | No |
| *TMIGD1* | AK172838 | ENSG00000182271 | down | 17.34 | 1.69E-11 | 8.99 | 4.87 | No |
| *ZG16* | AK125559 | ENSG00000174992 | down | 15.63 | 2.14E-03 | 10.96 | 6.99 | No |
| *CA7* | AY075019 | ENSG00000168748 | down | 15.61 | 1.79E-08 | 9.21 | 5.25 | No |
| *CCL21* | DQ895242 | ENSG00000137077 | down | 15.49 | 3.60E-04 | 10.10 | 6.15 | No |
| *CLDN8* | BC020866 | ENSG00000156284 | down | 13.28 | 3.40E-04 | 9.30 | 5.57 | No |
| *CA4* | AK298710 | ENSG00000167434 | down | 12.30 | 4.49E-05 | 9.68 | 6.06 | No |
| *BEST4* | AF440757 | ENSG00000142959 | down | 11.98 | 6.35E-11 | 9.08 | 5.49 | No |
| *SLC26A3* | BX640837 | ENSG00000091138 | down | 11.92 | 7.14E-03 | 12.28 | 8.70 | No |
| *SST* | AK312177 | ENSG00000157005 | down | 11.60 | 1.21E-09 | 8.80 | 5.27 | No |
| *MFAP5* | BC005901 | ENSG00000197614 | down | 11.16 | 1.45E-06 | 9.45 | 5.97 | No |
| *ADH1A // ADH1B* | BT019812 | ENSG00000187758 // ENSG00000196616 | down | 11.08 | 8.65E-07 | 9.51 | 6.04 | No |
| *SFRP2* | AF311912 | ENSG00000145423 | down | 10.91 | 1.67E-08 | 8.67 | 5.22 | No |
| *PLP1* | AK128782 | ENSG00000123560 | down | 10.85 | 2.88E-14 | 8.92 | 5.48 | No |
| *C21orf88* | AF426266 | ENSG00000184809 | down | 10.82 | 1.74E-07 | 8.66 | 5.23 | No |
| *XIST* | BX648323 | ENSG00000229807 | down | 10.46 | 2.20E-07 | 9.98 | 6.59 | No |
| *VIP* | M36634 | ENSG00000146469 | down | 10.15 | 5.80E-12 | 8.05 | 4.70 | No |
| *INSL5* | AY359030 | ENSG00000172410 | down | 9.74 | 1.02E-04 | 8.26 | 4.97 | No |
| *SCNN1B* | BC036352 | ENSG00000168447 | down | 9.33 | 1.58E-04 | 9.73 | 6.51 | No |
| *AQP8* | AF067797 | ENSG00000103375 | down | 9.25 | 9.61E-12 | 9.63 | 6.42 | No |
| *SLC4A4* | AF011390 | ENSG00000080493 | down | 9.06 | 1.02E-03 | 10.36 | 7.18 | No |
| *PYY* | BC041057 | ENSG00000131096 | down | 8.60 | 1.02E-06 | 9.78 | 6.67 | No |
| *OGN* | AF086912 | ENSG00000106809 | down | 8.52 | 2.01E-13 | 7.81 | 4.72 | No |
| *LYVE1* | AF127670 | ENSG00000133800 | down | 8.39 | 2.03E-04 | 8.87 | 5.80 | No |
| *SLC26A2* | BC059390 | ENSG00000155850 | down | 8.33 | 3.70E-03 | 11.46 | 8.40 | No |
| *B3GNT7* | AK126207 | ENSG00000156966 | down | 7.94 | 1.50E-03 | 10.05 | 7.06 | No |
| *SLC6A19* | AK096054 | ENSG00000174358 | down | 7.92 | 1.09E-13 | 8.60 | 5.62 | No |
| *ADAMDEC1* | AK316320 | ENSG00000134028 | down | 7.86 | 1.80E-05 | 10.97 | 7.99 | No |
| *CHGA* | BC006459 | ENSG00000100604 | down | 7.65 | 1.63E-12 | 8.33 | 5.39 | No |
| *TPH1* | X52836 | ENSG00000129167 | down | 7.43 | 1.85E-05 | 8.00 | 5.11 | No |
| *CA2* | CR613409 | ENSG00000104267 | down | 7.42 | 7.40E-03 | 11.56 | 8.67 | No |
| *CHRDL1* | BC002909 | ENSG00000101938 | down | 7.10 | 1.14E-10 | 8.04 | 5.21 | No |
| *TRPM6* | AF350881 | ENSG00000119121 | down | 7.04 | 5.75E-07 | 8.60 | 5.79 | No |
| *B3GALT5* | AY372061 | ENSG00000183778 | down | 6.64 | 1.14E-11 | 9.11 | 6.38 | No |
| *SLC30A10* | AK097467 | ENSG00000196660 | down | 6.58 | 2.16E-12 | 7.84 | 5.12 | No |
| *PCK1* | BC023978 | ENSG00000124253 | down | 6.46 | 4.26E-03 | 9.90 | 7.20 | No |
| *EFEMP1* | AK293058 | ENSG00000115380 | down | 6.09 | 8.16E-05 | 10.13 | 7.52 | No |
| *CXCL12* | DQ345520 | ENSG00000107562 | down | 6.02 | 1.65E-12 | 10.46 | 7.87 | No |
| *DSC3* | D17427 | ENSG00000134762 | down | 5.89 | 2.62E-03 | 8.20 | 5.65 | No |
| *DPT* | BC033736 | ENSG00000143196 | down | 5.73 | 1.88E-11 | 7.77 | 5.26 | No |
| *UGT2B15* | AF180322 | ENSG00000196620 | down | 5.73 | 3.44E-02 | 10.56 | 8.04 | Yes |
| *DCN* | CR616358 | ENSG00000011465 | down | 5.64 | 5.26E-03 | 10.73 | 8.23 | No |
| *ITLN1* | AY549722 | ENSG00000179914 | down | 5.53 | 4.41E-02 | 10.68 | 8.21 | No |
| *F13A1* | M14539 | ENSG00000124491 | down | 5.52 | 1.96E-03 | 9.80 | 7.33 | No |
| *C3* | BC150299 | ENSG00000125730 | down | 5.51 | 2.71E-02 | 10.24 | 7.77 | No |
| *---* | AK129601 | --- | down | 5.49 | 1.16E-03 | 9.74 | 7.28 | No |
| *BTNL3* | AY358385 | ENSG00000168903 | down | 5.40 | 5.42E-03 | 9.21 | 6.77 | No |
| *MYH11* | EU489063 | ENSG00000133392 | down | 5.37 | 1.65E-12 | 10.01 | 7.59 | No |
| *CEACAM7* | X98311 | ENSG00000007306 | down | 5.34 | 3.98E-02 | 12.28 | 9.86 | No |
| *MGP* | CR623037 | ENSG00000111341 | down | 5.30 | 2.98E-03 | 9.89 | 7.48 | No |
| *FN1* | CR749317 | ENSG00000115414 | down | 5.23 | 1.76E-02 | 9.65 | 7.26 | No |
| *---* | Y17867 | --- | down | 5.22 | 3.90E-07 | 8.01 | 5.63 | Yes |
| *---* | AF086125 | --- | down | 5.03 | 6.80E-07 | 7.65 | 5.32 | No |
| *BMP3* | M22491 | ENSG00000152785 | down | 5.00 | 1.07E-08 | 7.59 | 5.27 | No |
| *CP* | AK299272 | ENSG00000047457 | down | 4.94 | 2.34E-06 | 7.33 | 5.02 | No |
| *SFRP1* | BC036503 | ENSG00000104332 | down | 4.93 | 3.14E-09 | 7.45 | 5.15 | No |
| *CCDC80* | BC116179 | ENSG00000091986 | down | 4.88 | 4.44E-05 | 8.19 | 5.91 | No |
| *---* | AK126530 | --- | down | 4.85 | 7.37E-04 | 9.21 | 6.93 | Yes |
| *C7* | BC041807 | ENSG00000112936 | down | 4.85 | 7.16E-09 | 7.55 | 5.27 | No |
| *PRELP* | U29089 | ENSG00000188783 | down | 4.83 | 3.23E-07 | 7.97 | 5.70 | No |
| *APOD* | BC007402 | ENSG00000189058 | down | 4.81 | 3.34E-02 | 10.28 | 8.01 | No |
| *C2orf88* | AK075168 | ENSG00000187699 | down | 4.74 | 3.85E-05 | 8.43 | 6.19 | No |
| *CDR1* | BC113474 | ENSG00000184258 | down | 4.69 | 3.20E-04 | 8.18 | 5.95 | No |
| *PTPRR* | D64053 | ENSG00000153233 | down | 4.55 | 1.68E-12 | 7.92 | 5.73 | No |
| *JAM2* | AK294769 | ENSG00000154721 | down | 4.53 | 1.26E-03 | 8.54 | 6.36 | No |
| *FBLN1* | AK075566 | ENSG00000077942 | down | 4.44 | 1.88E-03 | 9.57 | 7.42 | No |
| *GREM2* | BC046632 | ENSG00000180875 | down | 4.44 | 2.38E-07 | 7.79 | 5.64 | No |
| *ABCG2* | BC092408 | ENSG00000118777 | down | 4.44 | 2.38E-08 | 7.27 | 5.12 | No |
| *PKIB* | BC036011 | ENSG00000135549 | down | 4.41 | 3.40E-03 | 8.65 | 6.51 | No |
| *GPX3* | CR623805 | ENSG00000211445 | down | 4.40 | 4.44E-05 | 10.06 | 7.93 | No |
| *ABCA8* | AB020629 | ENSG00000141338 | down | 4.33 | 9.69E-09 | 7.57 | 5.45 | No |
| *ANGPTL1* | AY358278 | ENSG00000116194 | down | 4.29 | 5.92E-10 | 7.06 | 4.95 | No |
| *SLC17A4* | AK024903 | ENSG00000146039 | down | 4.29 | 9.50E-03 | 8.69 | 6.59 | No |
| *FBLN2* | AK304777 | ENSG00000163520 | down | 4.22 | 3.23E-07 | 8.42 | 6.34 | No |
| *MFAP4* | CR613174 | ENSG00000166482 | down | 4.22 | 2.78E-03 | 9.23 | 7.15 | No |
| *NAALADL1* | BC143644 | ENSG00000168060 | down | 4.22 | 1.93E-09 | 7.48 | 5.40 | No |
| *DES* | BC032116 | ENSG00000175084 | down | 4.21 | 3.03E-12 | 9.08 | 7.00 | No |
| *---* | AK125288 | --- | down | 4.21 | 1.36E-04 | 8.51 | 6.44 | Yes |
| *EDIL3* | BC030828 | ENSG00000164176 | down | 4.17 | 3.60E-04 | 9.84 | 7.78 | No |
| *---* | AL832503 | --- | down | 4.16 | 2.36E-03 | 9.65 | 7.59 | No |
| *---* | AF146019 | --- | down | 4.10 | 2.02E-03 | 8.74 | 6.71 | No |
| *CRYAB* | BC107897 | ENSG00000109846 | down | 4.07 | 1.40E-03 | 8.64 | 6.61 | No |
| *MAMDC2* | BC045569 | ENSG00000165072 | down | 4.07 | 1.07E-07 | 7.09 | 5.06 | No |
| *---* | AK026893 | --- | down | 4.06 | 1.92E-03 | 7.95 | 5.93 | No |
| *---* | AK055059 | --- | down | 4.04 | 1.23E-02 | 8.47 | 6.46 | No |
| *HRASLS2* | AB453251 | ENSG00000133328 | down | 4.03 | 5.63E-05 | 7.34 | 5.33 | No |
| *PGM5* | AK297108 | ENSG00000154330 | down | 4.00 | 5.26E-03 | 7.66 | 5.66 | No |
| *FABP4* | BC003672 | ENSG00000170323 | down | 3.98 | 2.42E-07 | 6.42 | 4.43 | No |
| *MUC5B* | U95031 | ENSG00000117983 | down | 3.96 | 3.82E-03 | 11.36 | 9.37 | No |
| *COL14A1* | BC140893 | ENSG00000187955 | down | 3.93 | 4.88E-03 | 7.71 | 5.73 | No |
| *SFRP4* | AF026692 | ENSG00000106483 | down | 3.93 | 4.54E-07 | 7.11 | 5.14 | No |
| *---* | AK122718 | --- | down | 3.92 | 3.75E-05 | 7.34 | 5.37 | No |
| *MUC5B* | AF086604 | ENSG00000117983 | down | 3.86 | 3.68E-03 | 10.58 | 8.64 | No |
| *CADM3* | AF363367 | ENSG00000162706 | down | 3.84 | 3.94E-11 | 7.48 | 5.54 | No |
| *ZEB2* | AB056507 | ENSG00000169554 | down | 3.81 | 1.44E-03 | 9.34 | 7.41 | No |
| *ALPI* | BC132678 | ENSG00000163295 | down | 3.79 | 4.71E-09 | 8.15 | 6.23 | No |
| *IGFBP3* | AK091745 | ENSG00000146674 | down | 3.75 | 3.02E-11 | 10.17 | 8.27 | No |
| *CFH* | AK291395 | ENSG00000000971 | down | 3.70 | 7.96E-03 | 8.39 | 6.50 | No |
| *ANPEP* | AB209918 | ENSG00000166825 | down | 3.69 | 2.29E-07 | 9.61 | 7.72 | No |
| *FAM5C* | AB111893 | ENSG00000162670 | down | 3.68 | 2.04E-05 | 7.03 | 5.15 | No |
| *ST6GAL2* | AB058780 | ENSG00000144057 | down | 3.67 | 2.84E-04 | 7.56 | 5.68 | No |
| *USP2* | BC041366 | ENSG00000036672 | down | 3.67 | 1.04E-03 | 7.86 | 5.98 | No |
| *IGF2BP3* | AF117108 | ENSG00000136231 | down | 3.67 | 4.27E-07 | 7.14 | 5.27 | No |
| *ITIH5* | AY358426 | ENSG00000123243 | down | 3.66 | 9.80E-03 | 8.24 | 6.36 | No |
| *ANK2* | BX537758 | ENSG00000145362 | down | 3.62 | 4.10E-08 | 7.09 | 5.23 | No |
| *DDR2* | AK225196 | ENSG00000162733 | down | 3.61 | 1.10E-02 | 8.65 | 6.80 | No |
| *---* | BC032913 | --- | down | 3.60 | 2.09E-10 | 7.62 | 5.77 | Yes |
| *SLIT3* | BC098388 | ENSG00000184347 | down | 3.58 | 3.83E-05 | 8.02 | 6.18 | No |
| *GPNMB* | X76534 | ENSG00000136235 | down | 3.57 | 7.02E-03 | 10.42 | 8.59 | No |
| *EDN3* | BC008876 | ENSG00000124205 | down | 3.55 | 1.12E-03 | 8.39 | 6.56 | No |
| *GLDN* | AK172756 | ENSG00000186417 | down | 3.55 | 1.38E-06 | 7.12 | 5.29 | No |
| *SECTM1* | U77643 | ENSG00000141574 | down | 3.50 | 1.18E-03 | 9.29 | 7.49 | No |
| *SPARCL1* | X86693 | ENSG00000152583 | down | 3.48 | 1.52E-02 | 8.99 | 7.19 | No |
| *PRKAR2B* | BC075800 | ENSG00000005249 | down | 3.47 | 2.56E-03 | 8.22 | 6.43 | No |
| *IL1R2* | BC039031 | ENSG00000115590 | down | 3.44 | 2.42E-03 | 9.13 | 7.35 | No |
| *---* | S55736 | --- | down | 3.44 | 1.88E-09 | 9.37 | 7.59 | No |
| *BTNL8* | AY358523 | ENSG00000113303 | down | 3.44 | 2.58E-03 | 8.50 | 6.72 | No |
| *MUSK* | BC109099 | ENSG00000030304 | down | 3.43 | 4.22E-07 | 6.83 | 5.05 | No |
| *SCG2* | BC022509 | ENSG00000171951 | down | 3.41 | 1.62E-12 | 6.81 | 5.04 | No |
| *---* | Y17868 | --- | down | 3.41 | 5.53E-08 | 7.46 | 5.69 | No |
| *ISLR* | AY358871 | ENSG00000129009 | down | 3.40 | 1.16E-02 | 9.06 | 7.29 | No |
| *MEP1A* | AF478685 | ENSG00000112818 | down | 3.38 | 5.77E-10 | 10.76 | 9.01 | No |
| *SEMA6D* | BC150253 | ENSG00000137872 | down | 3.37 | 1.30E-02 | 8.71 | 6.95 | No |
| *SPIB* | AK225225 | ENSG00000142539 | down | 3.35 | 1.62E-12 | 7.33 | 5.58 | No |
| *HAPLN1* | U43328 | ENSG00000145681 | down | 3.35 | 1.38E-04 | 7.54 | 5.80 | No |
| *RERGL* | BC042888 | ENSG00000111404 | down | 3.34 | 1.93E-09 | 6.45 | 4.70 | No |
| *SCN9A* | X82835 | ENSG00000169432 | down | 3.30 | 3.00E-07 | 6.90 | 5.18 | No |
| *MFSD4* | AX746641 | ENSG00000174514 | down | 3.30 | 8.48E-03 | 8.35 | 6.63 | No |
| *ST6GALNAC6* | AY358672 | ENSG00000160408 | down | 3.30 | 2.94E-02 | 11.01 | 9.29 | No |
| *NRXN1* | EF539882 | ENSG00000179915 | down | 3.29 | 3.74E-14 | 6.55 | 4.83 | No |
| *CHRNA3* | BC006114 | ENSG00000080644 | down | 3.29 | 1.21E-08 | 7.01 | 5.29 | No |
| *LIFR* | X61615 | ENSG00000113594 | down | 3.27 | 6.11E-05 | 6.84 | 5.13 | No |
| *EPHA3* | AF213459 | ENSG00000044524 | down | 3.23 | 9.53E-08 | 7.01 | 5.32 | No |
| *DPP10* | BC030832 | ENSG00000175497 | down | 3.23 | 8.74E-03 | 6.69 | 4.99 | No |
| *ASPA* | AK312901 | ENSG00000108381 | down | 3.23 | 2.05E-06 | 6.72 | 5.03 | No |
| *MEIS1* | BC043503 | ENSG00000143995 | down | 3.22 | 1.04E-04 | 7.19 | 5.51 | No |
| *CPM* | J04970 | ENSG00000135678 | down | 3.22 | 9.24E-04 | 9.25 | 7.57 | No |
| *SRPX* | AK125542 | ENSG00000101955 | down | 3.22 | 2.00E-03 | 7.85 | 6.16 | No |
| *MMRN1* | AK125557 | ENSG00000138722 | down | 3.21 | 8.22E-04 | 7.10 | 5.41 | No |
| *PDE3A* | U36798 | ENSG00000172572 | down | 3.21 | 2.01E-02 | 8.55 | 6.86 | No |
| *TMOD2* | BC064961 | ENSG00000128872 | down | 3.20 | 5.03E-04 | 7.49 | 5.82 | No |
| *FAM23A* | BC157856 | ENSG00000148483 | down | 3.20 | 3.00E-03 | 8.79 | 7.11 | No |
| *---* | BX648635 | --- | down | 3.20 | 1.57E-04 | 7.32 | 5.65 | No |
| *TNS1* | AK001785 | ENSG00000079308 | down | 3.18 | 9.57E-04 | 8.78 | 7.11 | No |
| *THBS2* | L12350 | ENSG00000186340 | down | 3.18 | 8.47E-08 | 8.47 | 6.80 | No |
| *CHAD* | AK292177 | ENSG00000136457 | down | 3.17 | 1.77E-06 | 7.74 | 6.08 | No |
| *PCOLCE2* | AK075478 | ENSG00000163710 | down | 3.16 | 1.89E-09 | 6.79 | 5.13 | No |
| *---* | AK126393 | --- | down | 3.16 | 6.27E-05 | 6.38 | 4.72 | No |
| *---* | BC040983 | --- | down | 3.15 | 4.40E-04 | 6.76 | 5.10 | No |
| *CST1* | BC021225 | ENSG00000170373 | down | 3.14 | 4.64E-02 | 8.30 | 6.65 | Yes |
| *SYNPO2* | AL833547 | ENSG00000172403 | down | 3.13 | 3.92E-03 | 8.96 | 7.31 | No |
| *ZFPM2* | AF119334 | ENSG00000169946 | down | 3.13 | 4.14E-03 | 7.05 | 5.41 | No |
| *CFD* | M84526 | ENSG00000197766 | down | 3.12 | 1.40E-02 | 9.99 | 8.35 | No |
| *CMAH* | AK303297 | ENSG00000168405 | down | 3.12 | 5.24E-03 | 7.59 | 5.95 | No |
| *REG1A* | AK291981 | ENSG00000115386 | down | 3.11 | 1.26E-03 | 10.03 | 8.40 | Yes |
| *GPC6* | AF105267 | ENSG00000183098 | down | 3.10 | 1.36E-03 | 7.77 | 6.14 | No |
| *SULT1A2* | U28169 | ENSG00000197165 | down | 3.07 | 4.15E-04 | 8.46 | 6.84 | No |
| *TPSAB1* | BC029356 | ENSG00000172236 | down | 3.06 | 3.90E-02 | 9.98 | 8.37 | Yes |
| *MUC12* | AF147790 | ENSG00000205277 | down | 3.05 | 4.73E-02 | 10.57 | 8.96 | No |
| *ADH1C* | X04299 | ENSG00000248144 | down | 3.04 | 2.24E-05 | 10.30 | 8.70 | No |
| *---* | AK093957 | --- | down | 3.03 | 1.58E-02 | 7.64 | 6.04 | No |
| *HSPB6* | CR618274 | ENSG00000004776 | down | 3.02 | 7.84E-04 | 7.38 | 5.78 | No |
| *SERPING1* | CR614379 | ENSG00000149131 | down | 3.02 | 2.82E-03 | 9.26 | 7.66 | No |
| *---* | BX648964 | --- | down | 3.02 | 4.26E-03 | 7.14 | 5.54 | No |
| *CLDN23* | AK123547 | --- | down | 3.00 | 2.16E-05 | 8.08 | 6.50 | No |
| *SEMA3D* | AY358937 | ENSG00000153993 | down | 3.00 | 3.04E-10 | 6.47 | 4.88 | No |
| *BEST2* | AF440756 | ENSG00000039987 | down | 2.99 | 3.79E-02 | 8.27 | 6.69 | No |
| *LPAR1* | BC036034 | ENSG00000198121 | down | 2.99 | 2.67E-11 | 9.11 | 7.53 | No |
| *MCOLN2* | AX748410 | ENSG00000153898 | down | 2.98 | 9.02E-03 | 7.74 | 6.17 | No |
| *MST1P2 // MST1P9* | AY192149 | ENSG00000186715 | down | 2.98 | 9.93E-05 | 8.10 | 6.52 | No |
| *TCF21* | BC025697 | ENSG00000118526 | down | 2.98 | 2.03E-02 | 8.61 | 7.03 | No |
| *SHISA3* | CR624275 | ENSG00000178343 | down | 2.97 | 2.28E-07 | 7.24 | 5.67 | No |
| *GLI3* | AK308429 | ENSG00000106571 | down | 2.96 | 6.39E-04 | 6.90 | 5.33 | No |
| *PADI2* | AB023211 | ENSG00000117115 | down | 2.95 | 3.00E-02 | 10.59 | 9.03 | No |
| *RNF152* | AK096495 | ENSG00000176641 | down | 2.95 | 1.18E-04 | 7.54 | 5.98 | No |
| *TPSB2* | AK308208 | ENSG00000197253 | down | 2.95 | 4.30E-02 | 10.01 | 8.45 | Yes |
| *KCTD12* | AK128363 | ENSG00000178695 | down | 2.95 | 1.24E-02 | 10.00 | 8.45 | No |
| *SLC15A1* | U21936 | ENSG00000088386 | down | 2.94 | 4.22E-03 | 7.18 | 5.63 | No |
| *ITM2A* | BC034485 | ENSG00000078596 | down | 2.93 | 2.48E-03 | 7.56 | 6.01 | No |
| *CASQ2* | AK129891 | ENSG00000118729 | down | 2.93 | 4.50E-11 | 6.51 | 4.96 | No |
| *AMPD1* | AK097077 | ENSG00000116748 | down | 2.92 | 4.16E-03 | 7.02 | 5.48 | No |
| *PRPH* | AK125587 | ENSG00000135406 | down | 2.90 | 2.01E-10 | 7.48 | 5.94 | No |
| *PI16* | AK124589 | ENSG00000164530 | down | 2.90 | 2.47E-07 | 7.69 | 6.15 | No |
| *GUCY1A3* | BX649180 | ENSG00000164116 | down | 2.89 | 7.48E-03 | 7.41 | 5.88 | No |
| *TMEM47* | BC039242 | ENSG00000147027 | down | 2.86 | 1.23E-07 | 10.25 | 8.73 | No |
| *STMN2* | AK297485 | ENSG00000104435 | down | 2.86 | 8.96E-03 | 7.70 | 6.18 | No |
| *CD36* | AK096858 | ENSG00000135218 | down | 2.85 | 4.60E-03 | 7.31 | 5.80 | No |
| *CPNE8* | AK098593 | ENSG00000139117 | down | 2.84 | 3.48E-03 | 7.26 | 5.75 | No |
| *---* | AK025042 | --- | down | 2.83 | 2.47E-05 | 6.16 | 4.66 | No |
| *CYBRD1* | AL136693 | ENSG00000071967 | down | 2.83 | 1.84E-02 | 9.60 | 8.10 | No |
| *CALB2* | AK222648 | ENSG00000172137 | down | 2.82 | 7.17E-07 | 6.87 | 5.37 | No |
| *SCARA5* | AK172746 | ENSG00000168079 | down | 2.82 | 1.38E-03 | 7.74 | 6.25 | No |
| *---* | BC000926 | --- | down | 2.81 | 1.18E-04 | 10.39 | 8.90 | No |
| *SPON1* | AK074803 | ENSG00000152268 | down | 2.81 | 1.80E-07 | 8.38 | 6.90 | No |
| *---* | AK095203 | --- | down | 2.80 | 1.22E-02 | 6.91 | 5.43 | No |
| *---* | AK054989 | --- | down | 2.77 | 1.50E-03 | 6.81 | 5.34 | Yes |
| *CFHR1* | BC107771 | ENSG00000244414 | down | 2.77 | 2.38E-02 | 7.82 | 6.35 | No |
| *CTSG* | DQ896320 | ENSG00000100448 | down | 2.77 | 2.21E-02 | 7.28 | 5.80 | No |
| *CXCL13* | AF044197 | ENSG00000156234 | down | 2.75 | 4.47E-02 | 6.94 | 5.48 | No |
| *CLEC3B* | X64559 | ENSG00000163815 | down | 2.75 | 2.04E-05 | 7.92 | 6.46 | No |
| *FXYD6* | CR617323 | ENSG00000137726 | down | 2.74 | 6.31E-05 | 9.06 | 7.60 | No |
| *OR51E2* | AY033942 | ENSG00000167332 | down | 2.74 | 1.25E-02 | 7.07 | 5.62 | No |
| *PCSK5* | AK225773 | ENSG00000099139 | down | 2.74 | 4.26E-03 | 7.62 | 6.16 | No |
| *SEMG1* | BC055416 | ENSG00000124233 | down | 2.73 | 7.84E-05 | 6.35 | 4.90 | No |
| *CNN1* | BC022015 | ENSG00000130176 | down | 2.73 | 5.96E-03 | 8.38 | 6.94 | No |
| *IGF1* | X57025 | ENSG00000017427 | down | 2.72 | 1.34E-03 | 8.86 | 7.42 | No |
| *---* | AK000787 | --- | down | 2.71 | 1.79E-02 | 7.42 | 5.98 | No |
| *ACTG2* | BC012617 | ENSG00000163017 | down | 2.70 | 3.87E-02 | 10.58 | 9.14 | No |
| *HPGD* | AK058013 | ENSG00000164120 | down | 2.70 | 1.89E-02 | 9.08 | 7.65 | No |
| *AHNAK* | AX746812 | ENSG00000124942 | down | 2.69 | 1.36E-03 | 10.49 | 9.06 | No |
| *ANO5* | AL833271 | ENSG00000171714 | down | 2.69 | 2.47E-02 | 6.71 | 5.28 | No |
| *OLFML1* | BX647908 | ENSG00000183801 | down | 2.69 | 4.02E-03 | 7.28 | 5.85 | No |
| *FHL1* | BC010998 | ENSG00000022267 | down | 2.69 | 4.40E-09 | 9.41 | 7.98 | No |
| *ABCA6* | AY028898 | ENSG00000154262 | down | 2.68 | 5.42E-07 | 6.40 | 4.98 | No |
| *CES2* | BX538086 | ENSG00000172831 | down | 2.68 | 2.29E-02 | 10.61 | 9.19 | No |
| *FGL2* | BC017813 | ENSG00000127951 | down | 2.68 | 1.06E-02 | 9.03 | 7.61 | No |
| *PTGIS* | D38145 | ENSG00000124212 | down | 2.67 | 1.29E-02 | 7.62 | 6.20 | No |
| *AOC3* | U39447 | ENSG00000131471 | down | 2.65 | 1.98E-04 | 7.18 | 5.77 | No |
| *MS4A1* | AK225630 | ENSG00000156738 | down | 2.65 | 7.86E-03 | 6.91 | 5.50 | No |
| *EBF1* | BC038805 | ENSG00000164330 | down | 2.65 | 2.64E-03 | 7.56 | 6.16 | No |
| *FOLR2* | AK222539 | ENSG00000165457 | down | 2.64 | 9.38E-03 | 9.19 | 7.80 | No |
| *LMOD1* | BC080187 | ENSG00000163431 | down | 2.64 | 2.17E-04 | 8.23 | 6.83 | No |
| *HOXD10* | BC069619 | ENSG00000128710 | down | 2.63 | 9.26E-03 | 7.64 | 6.25 | No |
| *TSPAN7* | AB062057 | ENSG00000156298 | down | 2.63 | 4.67E-02 | 9.95 | 8.55 | No |
| *PNMA2* | BC036489 | ENSG00000240694 | down | 2.62 | 3.40E-04 | 7.26 | 5.87 | No |
| *VSIG4* | AK075171 | ENSG00000155659 | down | 2.61 | 1.88E-03 | 8.06 | 6.68 | No |
| *TLR7* | AF240467 | ENSG00000196664 | down | 2.61 | 4.08E-03 | 7.90 | 6.51 | No |
| *PAG1* | AK289818 | ENSG00000076641 | down | 2.61 | 2.20E-03 | 8.71 | 7.33 | No |
| *---* | CR618530 | --- | down | 2.60 | 3.05E-02 | 7.24 | 5.86 | No |
| *TMOD1* | AK314533 | ENSG00000136842 | down | 2.60 | 1.58E-03 | 7.28 | 5.90 | No |
| *MATN2* | AK289721 | ENSG00000132561 | down | 2.60 | 1.70E-02 | 8.75 | 7.37 | No |
| *THRB* | AK096628 | ENSG00000151090 | down | 2.59 | 1.20E-03 | 7.47 | 6.10 | No |
| *PLN* | BC005269 | ENSG00000198523 | down | 2.59 | 4.44E-04 | 6.78 | 5.41 | No |
| *GNG7* | BC053630 | ENSG00000176533 | down | 2.58 | 7.26E-03 | 8.12 | 6.75 | No |
| *COLEC12* | AB038518 | ENSG00000158270 | down | 2.58 | 1.19E-10 | 7.23 | 5.87 | No |
| *IFI44L* | AK223087 | ENSG00000137959 | down | 2.56 | 2.74E-02 | 7.19 | 5.83 | No |
| *NLGN4X* | AX773938 | ENSG00000146938 | down | 2.56 | 4.42E-05 | 7.18 | 5.82 | No |
| *HRCT1* | AY358408 | ENSG00000196196 | down | 2.55 | 2.37E-02 | 7.62 | 6.27 | No |
| *GFRA2* | BC041688 | ENSG00000168546 | down | 2.55 | 1.85E-04 | 7.24 | 5.89 | No |
| *KIF5C* | AB011103 | ENSG00000168280 | down | 2.54 | 8.02E-07 | 6.45 | 5.11 | No |
| *RHOV* | CR599960 | ENSG00000104140 | down | 2.54 | 8.19E-04 | 7.87 | 6.53 | No |
| *SLIT2* | AF055585 | ENSG00000145147 | down | 2.53 | 1.97E-05 | 6.63 | 5.29 | No |
| *PMP22* | D11428 | ENSG00000109099 | down | 2.53 | 2.73E-02 | 10.14 | 8.80 | No |
| *EPB41L3* | BC008377 | ENSG00000082397 | down | 2.52 | 4.73E-02 | 9.11 | 7.78 | No |
| *NR5A2* | AB019246 | ENSG00000116833 | down | 2.51 | 2.62E-02 | 9.29 | 7.96 | No |
| *CNTFR* | BC001492 | ENSG00000122756 | down | 2.51 | 5.49E-07 | 7.35 | 6.02 | No |
| *GFRA3* | AY359037 | ENSG00000146013 | down | 2.50 | 1.76E-05 | 7.10 | 5.77 | No |
| *TMEM171* | BC018083 | ENSG00000157111 | down | 2.50 | 8.56E-03 | 9.27 | 7.95 | No |
| *MYL9* | D82057 | ENSG00000101335 | down | 2.49 | 4.13E-02 | 10.39 | 9.08 | No |
| *GNAO1* | BC030027 | ENSG00000087258 | down | 2.49 | 4.42E-05 | 7.18 | 5.87 | No |
| *ADAMTSL3* | BC128390 | ENSG00000156218 | down | 2.49 | 3.83E-04 | 6.89 | 5.57 | No |
| *ROR1* | M97675 | ENSG00000185483 | down | 2.49 | 1.17E-02 | 7.21 | 5.89 | No |
| *---* | AK308167 | --- | down | 2.49 | 1.49E-02 | 8.00 | 6.69 | Yes |
| *---* | AK126481 | --- | down | 2.49 | 5.79E-05 | 7.48 | 6.16 | No |
| *CNTN4* | AK125460 | ENSG00000144619 | down | 2.48 | 3.77E-04 | 6.50 | 5.19 | No |
| *---* | AK000085 | --- | down | 2.48 | 6.16E-05 | 8.31 | 7.00 | No |
| *FRZB* | CR622855 | ENSG00000162998 | down | 2.48 | 2.59E-02 | 8.21 | 6.90 | No |
| *---* | BC030100 | --- | down | 2.48 | 1.81E-02 | 7.08 | 5.77 | No |
| *HPGDS* | AK290075 | ENSG00000163106 | down | 2.48 | 2.45E-02 | 6.68 | 5.37 | No |
| *TTLL7* | BC028350 | ENSG00000137941 | down | 2.47 | 1.22E-03 | 7.23 | 5.93 | No |
| *EFHD1* | AK310565 | ENSG00000115468 | down | 2.47 | 2.36E-03 | 8.18 | 6.88 | No |
| *IGFBP6* | BC010162 | ENSG00000167779 | down | 2.47 | 3.79E-05 | 8.24 | 6.94 | No |
| *TP53INP2* | AY422170 | ENSG00000078804 | down | 2.46 | 5.91E-05 | 8.11 | 6.81 | No |
| *SYNM* | AL833262 | ENSG00000182253 | down | 2.46 | 2.19E-05 | 7.30 | 6.00 | No |
| *PDLIM3* | AF039018 | ENSG00000154553 | down | 2.46 | 2.69E-02 | 7.61 | 6.31 | No |
| *BST2* | AK303593 | ENSG00000130303 | down | 2.45 | 2.13E-02 | 8.22 | 6.93 | No |
| *CCL23* | U58913 | ENSG00000167236 | down | 2.44 | 7.18E-04 | 6.79 | 5.51 | No |
| *DHRS11* | AK026196 | ENSG00000108272 | down | 2.44 | 2.98E-03 | 10.07 | 8.78 | No |
| *MYLK* | BC064695 | ENSG00000065534 | down | 2.43 | 2.40E-10 | 9.02 | 7.73 | No |
| *SIGLEC1* | BC141884 | ENSG00000088827 | down | 2.43 | 3.37E-04 | 7.84 | 6.55 | No |
| *ABI3BP* | BC030221 | ENSG00000154175 | down | 2.42 | 5.66E-03 | 7.70 | 6.42 | No |
| *HHLA2* | AK000692 | ENSG00000114455 | down | 2.42 | 2.37E-02 | 10.20 | 8.93 | No |
| *MALL* | AK125647 | ENSG00000144063 | down | 2.42 | 9.36E-03 | 10.99 | 9.72 | No |
| *NTM* | AY358331 | ENSG00000182667 | down | 2.42 | 1.78E-04 | 6.81 | 5.54 | No |
| *SLC37A2* | AK074207 | ENSG00000134955 | down | 2.42 | 2.75E-12 | 7.29 | 6.01 | No |
| *C2orf40* | AF325503 | ENSG00000119147 | down | 2.41 | 2.10E-05 | 6.68 | 5.42 | No |
| *PHLPP2* | AB023148 | ENSG00000040199 | down | 2.41 | 6.14E-05 | 9.31 | 8.04 | No |
| *GPM6B* | AB209525 | ENSG00000046653 | down | 2.41 | 1.62E-04 | 6.30 | 5.03 | No |
| *---* | AK025116 | --- | down | 2.41 | 2.89E-02 | 6.26 | 4.99 | No |
| *C4orf7* | AY190326 | ENSG00000181617 | down | 2.40 | 2.36E-03 | 6.56 | 5.30 | No |
| *CCL19* | AB000887 | ENSG00000172724 | down | 2.40 | 3.03E-04 | 6.96 | 5.70 | No |
| *TUSC3* | AK026149 | ENSG00000104723 | down | 2.40 | 3.01E-08 | 6.15 | 4.89 | No |
| *RSPO3* | AF251057 | ENSG00000146374 | down | 2.40 | 1.36E-02 | 6.25 | 4.99 | No |
| *SEPP1* | AK096125 | ENSG00000250722 | down | 2.40 | 2.28E-02 | 11.24 | 9.98 | No |
| *ASPN* | BC063114 | ENSG00000106819 | down | 2.39 | 8.94E-07 | 6.31 | 5.06 | No |
| *GPR63* | AK289839 | ENSG00000112218 | down | 2.39 | 1.66E-03 | 7.44 | 6.19 | No |
| *MS4A2* | D10583 | ENSG00000149534 | down | 2.38 | 2.01E-02 | 6.52 | 5.27 | No |
| *ZFHX4* | AB083343 | ENSG00000091656 | down | 2.38 | 1.65E-12 | 6.72 | 5.47 | No |
| *UGT2B17* | U59209 | ENSG00000197888 | down | 2.38 | 3.27E-02 | 6.93 | 5.68 | No |
| *TMEM72* | AB235418 | ENSG00000187783 | down | 2.37 | 2.99E-04 | 7.81 | 6.57 | No |
| *UCHL1* | BC018838 | ENSG00000154277 | down | 2.36 | 1.32E-03 | 7.02 | 5.79 | No |
| *---* | BC089413 | --- | down | 2.36 | 2.43E-05 | 11.10 | 9.86 | Yes |
| *CDA* | BC048284 | ENSG00000158825 | down | 2.36 | 3.82E-08 | 8.47 | 7.23 | No |
| *OSR1* | AK074591 | ENSG00000143867 | down | 2.36 | 1.75E-09 | 6.83 | 5.60 | No |
| *CNTN1* | Z21488 | ENSG00000018236 | down | 2.36 | 1.50E-07 | 6.08 | 4.84 | No |
| *RUNX1T1* | BC005850 | ENSG00000079102 | down | 2.36 | 5.58E-04 | 6.67 | 5.43 | No |
| *FBN1* | X63556 | ENSG00000166147 | down | 2.35 | 2.96E-02 | 7.76 | 6.53 | No |
| *RERG* | BC007997 | ENSG00000134533 | down | 2.35 | 2.45E-04 | 6.59 | 5.36 | No |
| *ECM2* | BX537976 | ENSG00000106823 | down | 2.35 | 1.03E-06 | 6.59 | 5.36 | No |
| *CHL1* | AF002246 | ENSG00000134121 | down | 2.34 | 3.52E-05 | 6.86 | 5.63 | No |
| *---* | AK055640 | --- | down | 2.34 | 3.25E-02 | 6.54 | 5.32 | No |
| *---* | AF086154 | --- | down | 2.34 | 2.44E-03 | 8.62 | 7.40 | No |
| *---* | CR618029 | --- | down | 2.34 | 4.58E-02 | 7.55 | 6.33 | No |
| *CLIC5* | AK097048 | ENSG00000112782 | down | 2.34 | 4.78E-04 | 10.52 | 9.29 | No |
| *---* | AK096234 | --- | down | 2.33 | 1.34E-03 | 7.08 | 5.85 | No |
| *GGTA1* | AF378123 | ENSG00000204136 | down | 2.33 | 9.56E-04 | 7.00 | 5.78 | No |
| *GPM6A* | AK226176 | ENSG00000150625 | down | 2.33 | 3.59E-05 | 6.64 | 5.43 | No |
| *---* | AF085831 | --- | down | 2.32 | 8.34E-03 | 7.39 | 6.18 | Yes |
| *CES1* | AK292209 | ENSG00000198848 | down | 2.32 | 7.68E-05 | 7.52 | 6.30 | No |
| *ANO1* | AY728143 | ENSG00000131620 | down | 2.32 | 4.83E-02 | 8.99 | 7.77 | Yes |
| *NRP1* | CR749333 | ENSG00000099250 | down | 2.32 | 2.53E-02 | 8.39 | 7.18 | No |
| *SCGN* | BC003036 | ENSG00000079689 | down | 2.32 | 4.48E-06 | 6.26 | 5.05 | No |
| *---* | AF143876 | --- | down | 2.32 | 1.20E-04 | 6.02 | 4.81 | No |
| *RECK* | D50406 | ENSG00000122707 | down | 2.31 | 4.48E-03 | 7.01 | 5.80 | No |
| *FMN2* | BC112361 | ENSG00000155816 | down | 2.30 | 4.63E-04 | 5.82 | 4.62 | No |
| *---* | DQ786197 | --- | down | 2.30 | 2.83E-04 | 6.76 | 5.55 | No |
| *LMO3* | AK095595 | ENSG00000048540 | down | 2.30 | 2.03E-02 | 7.09 | 5.89 | No |
| *AEBP1* | BC038588 | ENSG00000106624 | down | 2.30 | 4.21E-02 | 9.08 | 7.88 | No |
| *FBLN5* | AY358898 | ENSG00000140092 | down | 2.29 | 2.50E-02 | 7.89 | 6.69 | No |
| *CSRP2* | CR598239 | ENSG00000175183 | down | 2.29 | 8.04E-03 | 7.85 | 6.66 | No |
| *---* | AK311733 | --- | down | 2.29 | 7.63E-09 | 6.28 | 5.08 | No |
| *ADHFE1* | BC047492 | ENSG00000147576 | down | 2.29 | 2.56E-03 | 7.03 | 5.84 | No |
| *HOXB13* | AY937237 | ENSG00000159184 | down | 2.28 | 1.58E-05 | 9.00 | 7.82 | No |
| *CDHR5* | AF258675 | ENSG00000099834 | down | 2.28 | 2.43E-09 | 10.42 | 9.23 | No |
| *FAM70A* | AK056403 | ENSG00000125355 | down | 2.28 | 3.30E-06 | 6.73 | 5.54 | No |
| *VIM* | BC066956 | ENSG00000026025 | down | 2.28 | 2.26E-07 | 10.41 | 9.22 | No |
| *PGM5P2* | BC007887 | ENSG00000227558 | down | 2.28 | 1.20E-02 | 7.41 | 6.22 | Yes |
| *FGFR1* | FJ809917 | ENSG00000077782 | down | 2.28 | 3.21E-02 | 8.04 | 6.85 | No |
| *MAP1B* | BC172388 | ENSG00000131711 | down | 2.28 | 1.71E-02 | 7.35 | 6.16 | No |
| *DPYD* | U20938 | ENSG00000188641 | down | 2.26 | 1.14E-02 | 7.48 | 6.30 | No |
| *TIMP3* | U14394 | ENSG00000100234 | down | 2.26 | 1.57E-07 | 9.96 | 8.78 | No |
| *A2M* | CR749334 | ENSG00000175899 | down | 2.26 | 3.40E-08 | 10.33 | 9.16 | No |
| *PLXDC2* | AY358486 | ENSG00000120594 | down | 2.26 | 1.99E-02 | 9.40 | 8.23 | No |
| *---* | AK095177 | --- | down | 2.26 | 3.79E-04 | 7.64 | 6.47 | No |
| *RTN1* | BC068510 | ENSG00000139970 | down | 2.25 | 1.02E-03 | 6.17 | 5.00 | No |
| *MS4A4A* | CR605689 | ENSG00000110079 | down | 2.25 | 2.91E-02 | 7.52 | 6.35 | No |
| *IFIT1* | AK308688 | ENSG00000185745 | down | 2.25 | 3.54E-03 | 6.73 | 5.57 | No |
| *CACNB1* | M92303 | ENSG00000067191 | down | 2.24 | 9.36E-04 | 8.06 | 6.89 | No |
| *KLF12* | AK291397 | ENSG00000118922 | down | 2.24 | 6.32E-03 | 8.34 | 7.18 | No |
| *PAMR1* | AK027841 | ENSG00000149090 | down | 2.24 | 1.13E-02 | 7.09 | 5.93 | No |
| *CLEC10A* | BC039011 | ENSG00000132514 | down | 2.23 | 7.38E-04 | 6.97 | 5.81 | No |
| *---* | AK021709 | --- | down | 2.23 | 8.41E-04 | 6.46 | 5.31 | No |
| *LRRN2* | BC068541 | ENSG00000170382 | down | 2.23 | 2.27E-06 | 7.98 | 6.82 | No |
| *JAZF1* | AX746909 | ENSG00000153814 | down | 2.23 | 2.73E-02 | 7.83 | 6.68 | No |
| *BMP5* | BC027958 | ENSG00000112175 | down | 2.23 | 5.20E-03 | 6.99 | 5.84 | No |
| *TIMP2* | BC071586 | ENSG00000035862 | down | 2.22 | 2.12E-02 | 9.35 | 8.20 | No |
| *---* | AK128305 | --- | down | 2.22 | 2.86E-03 | 6.65 | 5.51 | Yes |
| *GPR34* | BC020678 | ENSG00000171659 | down | 2.22 | 2.42E-02 | 7.75 | 6.60 | No |
| *AKAP12* | U81607 | ENSG00000131016 | down | 2.22 | 6.06E-03 | 7.06 | 5.91 | No |
| *FMO5* | AK225739 | ENSG00000131781 | down | 2.22 | 1.63E-02 | 8.76 | 7.61 | No |
| *GHR* | X06562 | ENSG00000112964 | down | 2.22 | 4.34E-02 | 6.91 | 5.76 | No |
| *---* | AK124396 | --- | down | 2.22 | 5.26E-03 | 6.64 | 5.49 | No |
| *FLRT2* | BC143936 | ENSG00000185070 | down | 2.21 | 7.22E-03 | 7.06 | 5.91 | No |
| *GSTM2* | BC017836 | ENSG00000213366 | down | 2.21 | 7.09E-09 | 8.28 | 7.13 | No |
| *---* | AK000795 | --- | down | 2.21 | 4.10E-03 | 5.63 | 4.48 | No |
| *CD48* | BC016182 | ENSG00000117091 | down | 2.21 | 3.37E-02 | 8.41 | 7.27 | No |
| *PPAP2B* | AF480883 | ENSG00000162407 | down | 2.20 | 1.24E-02 | 9.86 | 8.72 | No |
| *---* | U63836 | --- | down | 2.20 | 4.65E-02 | 12.97 | 11.84 | No |
| *FAM126A* | AL833296 | ENSG00000122591 | down | 2.20 | 4.19E-02 | 7.06 | 5.92 | No |
| *SLCO4C1* | BX647349 | ENSG00000173930 | down | 2.20 | 1.41E-02 | 6.56 | 5.42 | No |
| *---* | AF379613 | --- | down | 2.19 | 2.17E-02 | 6.34 | 5.20 | No |
| *NAT8B* | AF185571 | ENSG00000144035 | down | 2.19 | 3.35E-12 | 7.24 | 6.11 | No |
| *OMD* | AB000114 | ENSG00000127083 | down | 2.19 | 1.14E-06 | 6.01 | 4.88 | No |
| *BMP6* | M60315 | ENSG00000153162 | down | 2.19 | 2.11E-02 | 7.01 | 5.88 | No |
| *---* | BC039414 | --- | down | 2.19 | 4.14E-02 | 6.56 | 5.43 | No |
| *LRIG1* | BC071561 | ENSG00000144749 | down | 2.18 | 3.98E-03 | 8.76 | 7.64 | No |
| *ANTXR1* | AF279145 | ENSG00000169604 | down | 2.18 | 4.76E-02 | 9.45 | 8.33 | No |
| *MPEG1* | AK301210 | ENSG00000197629 | down | 2.18 | 1.61E-02 | 10.76 | 9.64 | No |
| *---* | AK127644 | --- | down | 2.18 | 1.02E-02 | 7.71 | 6.59 | No |
| *PER3* | BC146781 | ENSG00000049246 | down | 2.17 | 1.07E-02 | 8.05 | 6.93 | No |
| *MS4A6A* | AK291870 | ENSG00000110077 | down | 2.17 | 2.07E-02 | 9.44 | 8.32 | No |
| *TUBAL3* | AK025318 | ENSG00000178462 | down | 2.17 | 1.70E-02 | 7.07 | 5.96 | No |
| *NR3C2* | M16801 | ENSG00000151623 | down | 2.17 | 1.54E-03 | 8.83 | 7.71 | No |
| *SLPI* | BC020708 | ENSG00000124107 | down | 2.16 | 4.89E-02 | 10.41 | 9.30 | No |
| *UBE2QL1* | AK057805 | ENSG00000215218 | down | 2.16 | 2.20E-03 | 6.64 | 5.53 | No |
| *ARHGAP15* | BC029346 | ENSG00000075884 | down | 2.15 | 3.79E-02 | 6.74 | 5.63 | No |
| *MAPK10* | BC051731 | ENSG00000109339 | down | 2.15 | 1.65E-02 | 7.04 | 5.94 | No |
| *MAB21L2* | AF262032 | ENSG00000181541 | down | 2.15 | 1.80E-10 | 6.70 | 5.60 | No |
| *PLEKHH2* | AL832207 | ENSG00000152527 | down | 2.14 | 2.53E-02 | 7.33 | 6.23 | No |
| *PTGFR* | BC035694 | ENSG00000122420 | down | 2.14 | 2.48E-02 | 6.69 | 5.59 | No |
| *GLIPR2* | AK074488 | ENSG00000122694 | down | 2.14 | 1.37E-02 | 8.69 | 7.59 | No |
| *---* | AB209012 | --- | down | 2.14 | 9.44E-08 | 7.58 | 6.48 | No |
| *SULT1A1* | CR601418 | ENSG00000196502 | down | 2.13 | 1.78E-03 | 8.33 | 7.24 | No |
| *GDPD2* | AB048363 | ENSG00000130055 | down | 2.13 | 6.11E-10 | 6.82 | 5.73 | No |
| *ENPP2* | BC034961 | ENSG00000136960 | down | 2.13 | 5.65E-07 | 8.54 | 7.45 | No |
| *DOCK10* | AB014594 | ENSG00000135905 | down | 2.12 | 5.70E-03 | 7.19 | 6.10 | No |
| *---* | BC071787 | --- | down | 2.12 | 1.10E-02 | 6.44 | 5.35 | No |
| *---* | AF116647 | --- | down | 2.12 | 1.28E-03 | 6.39 | 5.31 | Yes |
| *CDHR2* | AK315055 | ENSG00000074276 | down | 2.12 | 1.29E-02 | 9.22 | 8.14 | No |
| *VEPH1* | AB051479 | ENSG00000197415 | down | 2.11 | 3.23E-02 | 6.69 | 5.62 | No |
| *FAM13C* | AK316542 | ENSG00000148541 | down | 2.11 | 2.81E-04 | 6.87 | 5.79 | No |
| *FBXO32* | EF143260 | ENSG00000156804 | down | 2.11 | 2.93E-02 | 8.41 | 7.33 | No |
| *AHCYL2* | BC024325 | ENSG00000158467 | down | 2.11 | 1.46E-02 | 11.03 | 9.95 | No |
| *CCL28* | AF110384 | ENSG00000151882 | down | 2.11 | 1.70E-02 | 10.54 | 9.46 | No |
| *CCDC68* | AF273051 | ENSG00000166510 | down | 2.10 | 2.20E-03 | 7.34 | 6.26 | No |
| *MEIS2* | BC007202 | ENSG00000134138 | down | 2.10 | 1.19E-02 | 6.92 | 5.86 | No |
| *BCAT1* | AK128527 | ENSG00000060982 | down | 2.10 | 1.65E-02 | 6.52 | 5.45 | No |
| *ADAM28* | AK225921 | ENSG00000042980 | down | 2.10 | 2.76E-02 | 7.77 | 6.70 | No |
| *SCIN* | AB067492 | ENSG00000006747 | down | 2.10 | 2.98E-02 | 7.57 | 6.50 | No |
| *TRANK1* | AB002340 | ENSG00000168016 | down | 2.09 | 2.05E-04 | 7.35 | 6.29 | No |
| *SGSM1* | AB275761 | ENSG00000167037 | down | 2.09 | 4.11E-02 | 7.19 | 6.13 | No |
| *---* | AK055942 | --- | down | 2.09 | 9.62E-03 | 8.34 | 7.28 | No |
| *AHNAK2* | BC004283 | ENSG00000185567 | down | 2.09 | 9.98E-03 | 6.86 | 5.80 | No |
| *WASF3* | BC050283 | ENSG00000132970 | down | 2.09 | 6.20E-03 | 6.62 | 5.56 | No |
| *CD163L1* | AF264014 | ENSG00000177675 | down | 2.09 | 6.12E-03 | 7.61 | 6.54 | No |
| *---* | AK056919 | --- | down | 2.09 | 1.64E-02 | 6.64 | 5.57 | No |
| *---* | BC073771 | --- | down | 2.08 | 1.82E-04 | 10.11 | 9.06 | No |
| *NOVA1* | AK226114 | ENSG00000139910 | down | 2.08 | 1.58E-03 | 7.00 | 5.95 | No |
| *C10orf72* | BC041414 | ENSG00000165633 | down | 2.08 | 3.77E-02 | 7.78 | 6.73 | No |
| *---* | AK056897 | --- | down | 2.08 | 3.61E-02 | 6.70 | 5.65 | No |
| *WISP2* | CR603485 | ENSG00000064205 | down | 2.07 | 2.04E-05 | 7.62 | 6.57 | No |
| *VN1R1* | AK094452 | ENSG00000178201 | down | 2.07 | 1.58E-03 | 6.94 | 5.89 | No |
| *CDKL1* | AF390028 | ENSG00000100490 | down | 2.07 | 1.62E-03 | 7.49 | 6.43 | No |
| *DCLK1* | BC152456 | ENSG00000133083 | down | 2.07 | 5.65E-05 | 6.47 | 5.42 | No |
| *AIF1* | BC009474 | ENSG00000204472 // ENSG00000237727 | down | 2.07 | 3.01E-07 | 8.92 | 7.87 | No |
| *---* | AL117237 | --- | down | 2.07 | 8.44E-03 | 8.01 | 6.96 | No |
| *---* | AY343891 | --- | down | 2.06 | 4.48E-03 | 5.82 | 4.77 | Yes |
| *---* | CR613436 | --- | down | 2.06 | 3.84E-04 | 6.65 | 5.61 | No |
| *TBX10* | AY229977 | ENSG00000167800 | down | 2.06 | 4.13E-02 | 7.80 | 6.76 | No |
| *CYR61* | Y11307 | ENSG00000142871 | down | 2.06 | 4.50E-02 | 7.84 | 6.80 | No |
| *RIMKLA* | BC039737 | ENSG00000177181 | down | 2.06 | 1.18E-02 | 8.05 | 7.01 | No |
| *KLF9* | D31716 | ENSG00000119138 | down | 2.06 | 3.16E-03 | 8.11 | 7.06 | No |
| *---* | AY726568 | --- | down | 2.06 | 2.79E-02 | 6.40 | 5.35 | No |
| *TTC22* | AK000626 | ENSG00000006555 | down | 2.05 | 1.07E-02 | 8.37 | 7.33 | No |
| *MX1* | AK096355 | ENSG00000157601 | down | 2.05 | 3.07E-02 | 7.68 | 6.65 | No |
| *CCL13* | U59808 | ENSG00000181374 | down | 2.05 | 1.24E-02 | 7.05 | 6.01 | No |
| *SOSTDC1* | AX748107 | ENSG00000171243 | down | 2.05 | 4.43E-02 | 7.31 | 6.28 | No |
| *PDE5A* | D89094 | ENSG00000138735 | down | 2.05 | 3.67E-02 | 7.50 | 6.46 | No |
| *C20orf194* | AK226100 | ENSG00000088854 | down | 2.04 | 6.83E-10 | 6.96 | 5.93 | No |
| *PCSK2* | BC040546 | ENSG00000125851 | down | 2.04 | 7.39E-04 | 6.58 | 5.56 | No |
| *TCF4* | BC031056 | ENSG00000196628 | down | 2.04 | 8.56E-07 | 9.30 | 8.27 | No |
| *IL16* | AL833554 | ENSG00000172349 | down | 2.04 | 3.75E-02 | 7.74 | 6.71 | No |
| *ITGBL1* | AK291340 | ENSG00000198542 | down | 2.04 | 6.31E-05 | 6.41 | 5.38 | No |
| *INA* | BC006359 | ENSG00000148798 | down | 2.04 | 2.78E-04 | 6.15 | 5.13 | No |
| *LGI1* | BC022500 | ENSG00000108231 | down | 2.04 | 1.54E-07 | 5.98 | 4.95 | No |
| *BVES* | BC040502 | ENSG00000112276 | down | 2.04 | 3.75E-02 | 7.11 | 6.08 | No |
| *EPHA7* | L36642 | ENSG00000135333 | down | 2.04 | 1.68E-03 | 6.56 | 5.52 | No |
| *---* | AK025818 | --- | down | 2.03 | 2.32E-02 | 9.04 | 8.02 | No |
| *---* | AK000735 | --- | down | 2.03 | 3.97E-02 | 8.08 | 7.05 | No |
| *SGCE* | AJ000534 | ENSG00000127990 | down | 2.03 | 9.86E-03 | 7.49 | 6.47 | No |
| *GNB4* | AK001890 | ENSG00000114450 | down | 2.02 | 3.31E-02 | 7.07 | 6.06 | No |
| *MITF* | AB006909 | ENSG00000187098 | down | 2.02 | 3.38E-02 | 7.38 | 6.37 | No |
| *MT1F* | BC108722 | ENSG00000198417 | down | 2.02 | 3.92E-05 | 10.80 | 9.79 | No |
| *---* | BX537651 | --- | down | 2.02 | 3.63E-02 | 6.63 | 5.61 | Yes |
| *SFTA1P* | AY102069 | ENSG00000225383 | down | 2.02 | 1.01E-04 | 5.84 | 4.83 | No |
| *SLC10A5* | AK095808 | ENSG00000205184 | down | 2.02 | 3.61E-02 | 7.92 | 6.91 | No |
| *STAP1* | BC014958 | ENSG00000035720 | down | 2.01 | 2.90E-02 | 6.23 | 5.23 | No |
| *RAB3B* | AK291653 | ENSG00000169213 | down | 2.01 | 3.61E-02 | 6.91 | 5.91 | No |
| *---* | AF116637 | --- | down | 2.01 | 4.75E-02 | 8.93 | 7.92 | No |
| *---* | BC110411 | --- | down | 2.01 | 9.86E-03 | 6.95 | 5.95 | No |
| *CDH2* | S42303 | ENSG00000170558 | down | 2.01 | 1.18E-07 | 6.32 | 5.31 | No |
| *SV2B* | AK294902 | ENSG00000185518 | down | 2.01 | 5.71E-07 | 6.24 | 5.23 | No |
| *SVEP1* | AY243503 | ENSG00000165124 | down | 2.01 | 2.34E-02 | 6.82 | 5.81 | No |
| *---* | AK130718 | --- | down | 2.01 | 5.77E-05 | 6.12 | 5.12 | No |
| *NPY* | BC029497 | ENSG00000122585 | down | 2.01 | 5.61E-04 | 6.94 | 5.93 | No |
| *ADAMTS1* | BX647388 | ENSG00000154734 | down | 2.00 | 4.61E-02 | 7.28 | 6.28 | No |
| *NBPF1* | BC093404 | ENSG00000219481 | down | 2.00 | 2.17E-04 | 9.34 | 8.34 | No |
| *MMP28* | AK122604 | ENSG00000129270 | down | 2.00 | 1.07E-07 | 8.07 | 7.07 | No |
| *LRRC15* | AK125112 | ENSG00000172061 | down | 1.99 | 1.48E-02 | 8.01 | 7.02 | Yes |
| *---* | BC065843 | --- | down | 1.99 | 2.36E-02 | 6.76 | 5.76 | Yes |
| *SLC13A2* | AK298388 | ENSG00000007216 | down | 1.99 | 2.79E-04 | 8.28 | 7.28 | No |
| *LDHD* | BC040279 | ENSG00000166816 | down | 1.99 | 3.48E-07 | 8.47 | 7.48 | No |
| *NCAM1* | BC047244 | ENSG00000149294 | down | 1.99 | 1.98E-03 | 7.50 | 6.51 | No |
| *---* | AL389956 | --- | down | 1.99 | 3.53E-02 | 6.59 | 5.60 | Yes |
| *SAMD9* | AF445355 | ENSG00000205413 | down | 1.99 | 3.09E-02 | 7.71 | 6.72 | No |
| *---* | AK093863 | --- | down | 1.98 | 2.65E-08 | 7.23 | 6.25 | No |
| *CHODL* | AK022689 | ENSG00000154645 | down | 1.98 | 8.04E-05 | 6.11 | 5.12 | No |
| *CMA1* | BC103975 | ENSG00000092009 | down | 1.98 | 2.26E-02 | 6.30 | 5.32 | No |
| *FAM30A // KIAA0125* | AF161538 | ENSG00000226777 | down | 1.98 | 6.14E-07 | 7.55 | 6.56 | Yes |
| *TMCC3* | BC040535 | ENSG00000057704 | down | 1.98 | 6.14E-08 | 9.23 | 8.24 | No |
| *XPNPEP2* | U90724 | ENSG00000122121 | down | 1.98 | 2.59E-02 | 6.70 | 5.71 | No |
| *SSBP2* | AK294864 | ENSG00000145687 | down | 1.98 | 4.41E-02 | 8.49 | 7.50 | No |
| *TRPC1* | X89066 | ENSG00000144935 | down | 1.97 | 1.14E-02 | 6.34 | 5.36 | No |
| *---* | CR611171 | --- | down | 1.97 | 4.37E-02 | 7.02 | 6.05 | No |
| *BCAS1* | AK225656 | ENSG00000064787 | down | 1.97 | 9.72E-03 | 10.21 | 9.24 | No |
| *CNRIP1* | AY144596 | ENSG00000119865 | down | 1.97 | 3.71E-02 | 7.58 | 6.60 | No |
| *ALPPL2* | J04948 | ENSG00000163286 | down | 1.97 | 2.84E-03 | 7.59 | 6.61 | No |
| *FCGR2A // HSPA6* | DQ895657 | ENSG00000143226 // ENSG00000173110 | down | 1.97 | 1.66E-02 | 7.44 | 6.46 | No |
| *CD248* | AJ295846 | ENSG00000174807 | down | 1.97 | 2.80E-02 | 7.40 | 6.42 | No |
| *---* | AK022347 | --- | down | 1.97 | 2.44E-02 | 6.79 | 5.81 | No |
| *BGN* | AX747825 | ENSG00000182492 | down | 1.97 | 3.54E-06 | 9.65 | 8.68 | Yes |
| *PDE7B* | AJ251860 | ENSG00000171408 | down | 1.97 | 4.47E-02 | 7.26 | 6.29 | No |
| *KAT2B* | BC070075 | ENSG00000114166 | down | 1.96 | 2.33E-02 | 8.05 | 7.08 | No |
| *---* | BC013942 | --- | down | 1.96 | 1.43E-02 | 6.46 | 5.49 | No |
| *PTRF* | BC066123 | ENSG00000177469 | down | 1.95 | 2.59E-02 | 8.03 | 7.07 | No |
| *---* | AX747263 | --- | down | 1.95 | 1.66E-02 | 6.75 | 5.79 | No |
| *ADCY5* | BC156217 | ENSG00000173175 | down | 1.94 | 1.88E-03 | 7.50 | 6.54 | No |
| *ABCA9* | AY028899 | ENSG00000154258 | down | 1.94 | 2.26E-07 | 5.95 | 5.00 | No |
| *FCGR2B* | AK308150 | ENSG00000072694 | down | 1.94 | 2.91E-02 | 7.11 | 6.15 | No |
| *---* | BC150525 | --- | down | 1.94 | 6.24E-04 | 6.44 | 5.48 | Yes |
| *RASSF4* | AB209446 | ENSG00000107551 | down | 1.94 | 4.53E-02 | 7.62 | 6.66 | No |
| *BNC2* | CR933649 | ENSG00000173068 | down | 1.94 | 4.46E-02 | 7.09 | 6.13 | No |
| *PEG10* | AB049150 | ENSG00000242265 | down | 1.94 | 2.29E-02 | 6.57 | 5.61 | No |
| *HSPA1A* | AK298056 | ENSG00000204389 | down | 1.94 | 6.18E-03 | 11.68 | 10.72 | Yes |
| *C1QB* | CR595684 | ENSG00000173369 | down | 1.93 | 4.41E-02 | 10.46 | 9.51 | No |
| *---* | AK126410 | --- | down | 1.93 | 3.17E-02 | 8.91 | 7.96 | No |
| *RGL1* | AF186780 | ENSG00000143344 | down | 1.93 | 1.69E-02 | 7.69 | 6.74 | No |
| *AMN* | AF328788 | ENSG00000166126 | down | 1.93 | 3.94E-07 | 8.52 | 7.57 | No |
| *OR6C3* | AB463979 | ENSG00000205329 | down | 1.93 | 6.70E-03 | 5.36 | 4.41 | No |
| *MS4A8B* | AF237905 | ENSG00000166959 | down | 1.93 | 2.22E-07 | 6.94 | 5.99 | No |
| *SFMBT2* | BC167860 | ENSG00000198879 | down | 1.93 | 4.02E-02 | 7.31 | 6.37 | No |
| *SMPDL3A* | CR620022 | ENSG00000172594 | down | 1.93 | 2.87E-02 | 8.27 | 7.33 | No |
| *P2RX1* | BC027949 | ENSG00000108405 | down | 1.92 | 1.25E-02 | 7.36 | 6.42 | No |
| *---* | CR749529 | --- | down | 1.92 | 2.38E-02 | 6.58 | 5.64 | No |
| *MBOAT1* | BC150652 | ENSG00000172197 | down | 1.92 | 1.08E-02 | 9.87 | 8.93 | No |
| *GCNT2* | AK307337 | ENSG00000111846 | down | 1.92 | 2.24E-05 | 6.04 | 5.10 | No |
| *UNC5C* | AF055634 | ENSG00000182168 | down | 1.92 | 8.74E-03 | 6.86 | 5.92 | No |
| *KIAA1211* | AK126014 | ENSG00000109265 // ENSG00000109323 | down | 1.91 | 1.36E-02 | 7.79 | 6.86 | No |
| *TSHZ2* | BX640770 | ENSG00000182463 | down | 1.91 | 2.11E-02 | 7.12 | 6.19 | No |
| *---* | L37727 | --- | down | 1.91 | 7.86E-03 | 11.29 | 10.35 | Yes |
| *PDCD4* | BC031049 | ENSG00000150593 | down | 1.91 | 9.82E-03 | 9.02 | 8.09 | No |
| *MXI1* | BC035128 | ENSG00000119950 | down | 1.91 | 1.98E-03 | 8.76 | 7.83 | No |
| *---* | AK024937 | --- | down | 1.91 | 3.57E-02 | 6.89 | 5.95 | No |
| *RUNDC3B* | AK055233 | ENSG00000105784 | down | 1.91 | 1.28E-02 | 6.26 | 5.32 | No |
| *---* | BC031342 | --- | down | 1.91 | 1.57E-02 | 6.90 | 5.97 | No |
| *HSPA1B* | AK303967 | ENSG00000232804 | down | 1.91 | 3.94E-03 | 9.99 | 9.05 | No |
| *C1QTNF2* | BC011699 | ENSG00000145861 | down | 1.91 | 2.46E-05 | 7.16 | 6.23 | Yes |
| *NAAA* | M92449 | ENSG00000138744 | down | 1.91 | 1.60E-02 | 9.57 | 8.64 | No |
| *---* | AK097806 | --- | down | 1.90 | 7.14E-03 | 6.44 | 5.51 | No |
| *MXRA8* | AB052096 | ENSG00000162576 | down | 1.90 | 2.49E-02 | 7.84 | 6.92 | No |
| *CCDC102B* | CR749520 | ENSG00000150636 | down | 1.90 | 9.50E-03 | 6.12 | 5.19 | No |
| *MXRA7* | BC053983 | ENSG00000182534 | down | 1.90 | 4.42E-02 | 9.45 | 8.53 | No |
| *GAS7* | AY327406 | ENSG00000007237 | down | 1.90 | 8.44E-04 | 7.26 | 6.33 | No |
| *TNFAIP8L3* | AY517501 | ENSG00000183578 | down | 1.90 | 3.04E-03 | 6.77 | 5.84 | No |
| *ACAT1* | D90228 | ENSG00000075239 | down | 1.90 | 6.10E-03 | 8.41 | 7.49 | No |
| *CTSW* | BC048255 | ENSG00000172543 | down | 1.90 | 5.94E-03 | 6.92 | 5.99 | No |
| *---* | AK091103 | --- | down | 1.90 | 2.26E-02 | 7.79 | 6.86 | No |
| *PLCL2* | AK309946 | ENSG00000154822 | down | 1.89 | 8.26E-03 | 6.84 | 5.92 | No |
| *UGP2* | CR602817 | ENSG00000169764 | down | 1.89 | 1.06E-03 | 9.42 | 8.50 | No |
| *---* | BC062632 | --- | down | 1.89 | 7.92E-03 | 7.37 | 6.46 | No |
| *KLRD1* | BC042884 | ENSG00000134539 | down | 1.89 | 1.48E-03 | 6.23 | 5.31 | No |
| *CNNM2* | AF216962 | ENSG00000148842 | down | 1.89 | 1.65E-12 | 7.73 | 6.81 | No |
| *NCAM2* | U75330 | ENSG00000154654 | down | 1.88 | 2.46E-06 | 6.41 | 5.50 | No |
| *---* | AK094277 | --- | down | 1.88 | 4.98E-03 | 6.42 | 5.51 | No |
| *ZDBF2* | AK127271 | ENSG00000204186 | down | 1.88 | 4.79E-02 | 6.11 | 5.19 | No |
| *CNNM4* | BC063295 | ENSG00000158158 | down | 1.88 | 1.50E-03 | 9.27 | 8.36 | No |
| *CD37* | AK058093 | ENSG00000104894 | down | 1.88 | 1.87E-05 | 8.24 | 7.33 | No |
| *---* | AK023905 | --- | down | 1.88 | 2.63E-02 | 5.40 | 4.48 | No |
| *HMCN1* | AF156100 | ENSG00000143341 | down | 1.88 | 7.15E-04 | 6.23 | 5.32 | No |
| *MAF* | AF055377 | ENSG00000178573 | down | 1.88 | 1.59E-02 | 6.73 | 5.81 | No |
| *PRDX6* | CR604144 | ENSG00000117592 | down | 1.88 | 7.43E-04 | 9.90 | 8.99 | No |
| *IL6R* | X12830 | ENSG00000160712 | down | 1.88 | 1.96E-02 | 7.75 | 6.84 | No |
| *GFRA1* | AF038421 | ENSG00000151892 | down | 1.88 | 2.43E-02 | 6.59 | 5.68 | No |
| *FLNA* | X53416 | ENSG00000196924 | down | 1.88 | 2.54E-08 | 9.14 | 8.23 | No |
| *GSN* | AK125819 | ENSG00000148180 | down | 1.88 | 1.92E-02 | 10.47 | 9.57 | No |
| *---* | BX537743 | --- | down | 1.88 | 3.12E-03 | 6.03 | 5.11 | No |
| *---* | M28204 | --- | down | 1.88 | 3.86E-02 | 12.51 | 11.60 | No |
| *MOXD1* | CR624520 | ENSG00000079931 | down | 1.88 | 1.48E-02 | 6.56 | 5.65 | No |
| *C6orf204* | BC064835 | ENSG00000111860 | down | 1.88 | 1.68E-02 | 6.34 | 5.43 | No |
| *RFX6* | BC039248 | ENSG00000185002 | down | 1.88 | 2.44E-03 | 5.99 | 5.08 | No |
| *MCC* | AK128596 | ENSG00000171444 | down | 1.88 | 2.94E-02 | 7.09 | 6.18 | No |
| *GZMA* | BC015739 | ENSG00000145649 | down | 1.88 | 2.91E-02 | 6.64 | 5.73 | No |
| *FAT4* | AY356402 | ENSG00000196159 | down | 1.88 | 3.56E-02 | 6.91 | 6.00 | No |
| *GCET2* | AY212246 | ENSG00000174500 | down | 1.87 | 1.91E-02 | 6.83 | 5.93 | No |
| *OSBPL6* | AB208898 | ENSG00000079156 | down | 1.87 | 7.71E-05 | 6.52 | 5.62 | No |
| *RNASE1* | CR593433 | ENSG00000129538 | down | 1.87 | 2.98E-02 | 12.14 | 11.23 | No |
| *NBEA* | AF467288 | ENSG00000172915 | down | 1.87 | 2.93E-02 | 6.67 | 5.76 | No |
| *PLCE1* | BC151854 | ENSG00000138193 | down | 1.87 | 2.45E-02 | 8.70 | 7.79 | No |
| *HDAC9* | AK304298 | ENSG00000048052 | down | 1.87 | 3.66E-02 | 7.00 | 6.09 | No |
| *STAB1* | BC150250 | ENSG00000010327 | down | 1.86 | 6.96E-03 | 8.11 | 7.21 | No |
| *ZNF135* | AK095796 | ENSG00000176293 | down | 1.86 | 2.12E-03 | 7.10 | 6.20 | No |
| *TTR* | BC020791 | ENSG00000118271 | down | 1.86 | 2.14E-03 | 6.66 | 5.76 | No |
| *FAM19A2* | BC050347 | ENSG00000198673 | down | 1.86 | 8.22E-03 | 5.93 | 5.03 | No |
| *CYP7B1* | AF127090 | ENSG00000172817 | down | 1.86 | 4.95E-02 | 6.67 | 5.78 | No |
| *XKR4* | AY534241 | ENSG00000206579 | down | 1.86 | 1.74E-05 | 6.65 | 5.76 | No |
| *HIGD1A* | AL110233 | ENSG00000181061 | down | 1.85 | 2.27E-02 | 9.37 | 8.48 | Yes |
| *FRMD6* | AL833158 | ENSG00000139926 | down | 1.85 | 4.99E-02 | 6.61 | 5.72 | No |
| *SSPN* | EU433933 | ENSG00000123096 | down | 1.85 | 2.00E-02 | 7.40 | 6.52 | No |
| *CLIC2* | AK292785 | ENSG00000155962 | down | 1.85 | 3.72E-02 | 6.37 | 5.49 | No |
| *KLF4* | AF105036 | ENSG00000136826 | down | 1.85 | 2.58E-02 | 9.32 | 8.43 | No |
| *---* | AK024861 | --- | down | 1.85 | 8.54E-03 | 6.94 | 6.05 | No |
| *SPRR2A // SPRR2C* | BC128049 | ENSG00000229035 // ENSG00000241794 | down | 1.85 | 3.06E-02 | 6.66 | 5.77 | No |
| *---* | AK094685 | --- | down | 1.85 | 1.53E-02 | 6.47 | 5.58 | No |
| *---* | BC094872 | --- | down | 1.84 | 4.59E-02 | 8.81 | 7.93 | Yes |
| *SOX5* | AK096569 | ENSG00000134532 | down | 1.84 | 7.88E-03 | 6.81 | 5.93 | No |
| *---* | AK022113 | --- | down | 1.84 | 3.47E-02 | 5.59 | 4.71 | No |
| *FMOD* | X75546 | ENSG00000122176 | down | 1.84 | 2.70E-02 | 9.80 | 8.92 | No |
| *---* | AK126009 | --- | down | 1.84 | 1.36E-03 | 6.08 | 5.21 | No |
| *---* | AK023999 | --- | down | 1.84 | 4.72E-02 | 6.75 | 5.87 | No |
| *FAM7A2 // FAM7A3* | BC023564 | ENSG00000178081 // ENSG00000215312 | down | 1.83 | 1.88E-12 | 7.51 | 6.64 | Yes |
| *---* | AF085861 | --- | down | 1.83 | 2.82E-03 | 5.88 | 5.01 | No |
| *ENDOD1* | AB020637 | ENSG00000149218 | down | 1.83 | 1.52E-02 | 8.88 | 8.01 | No |
| *---* | AK098511 | --- | down | 1.83 | 3.03E-02 | 6.29 | 5.42 | Yes |
| *FAIM3* | BC006401 | ENSG00000162894 | down | 1.83 | 4.97E-02 | 7.28 | 6.40 | No |
| *CHRM2* | AL832585 | ENSG00000181072 | down | 1.83 | 9.90E-05 | 6.65 | 5.79 | No |
| *CITED2* | AF109161 | ENSG00000164442 | down | 1.83 | 2.17E-02 | 9.51 | 8.64 | No |
| *RRAGD* | AL137502 | ENSG00000025039 | down | 1.83 | 1.63E-05 | 6.82 | 5.95 | No |
| *---* | AK021932 | --- | down | 1.83 | 3.96E-02 | 5.67 | 4.80 | No |
| *PRSS12* | AJ001531 | ENSG00000164099 | down | 1.83 | 8.90E-03 | 7.63 | 6.76 | No |
| *DMXL2* | AF389880 | ENSG00000104093 | down | 1.82 | 1.26E-02 | 8.29 | 7.42 | No |
| *---* | AK054930 | --- | down | 1.82 | 4.85E-02 | 5.44 | 4.58 | No |
| *MEG3* | CR626265 | ENSG00000214548 | down | 1.82 | 1.41E-02 | 7.40 | 6.53 | No |
| *SORBS1* | BC152463 | ENSG00000095637 | down | 1.82 | 2.06E-02 | 7.87 | 7.01 | No |
| *GPR124* | AK027296 | ENSG00000020181 | down | 1.82 | 2.27E-02 | 8.04 | 7.18 | No |
| *RNF150* | AK130520 | ENSG00000170153 | down | 1.82 | 3.20E-04 | 6.36 | 5.50 | No |
| *MXD1* | BC098396 | ENSG00000059728 | down | 1.81 | 2.73E-02 | 9.38 | 8.52 | No |
| *CLIP3* | AK094738 | ENSG00000105270 | down | 1.81 | 5.62E-09 | 7.72 | 6.87 | No |
| *AXL* | BC032229 | ENSG00000167601 | down | 1.81 | 1.64E-02 | 8.31 | 7.46 | No |
| *---* | AK022263 | --- | down | 1.81 | 2.40E-07 | 6.84 | 5.98 | No |
| *---* | AK124033 | --- | down | 1.81 | 4.36E-02 | 7.27 | 6.41 | No |
| *---* | AF116643 | --- | down | 1.81 | 1.22E-04 | 6.57 | 5.71 | No |
| *LRRC19* | AK024955 | ENSG00000184434 | down | 1.81 | 2.81E-02 | 7.92 | 7.06 | No |
| *RNF180* | AL832580 | ENSG00000164197 | down | 1.81 | 1.52E-02 | 6.35 | 5.50 | No |
| *---* | AK057984 | --- | down | 1.80 | 2.56E-03 | 6.16 | 5.31 | Yes |
| *C16orf89* | AY358483 | ENSG00000153446 | down | 1.80 | 3.14E-02 | 7.33 | 6.48 | No |
| *---* | AK055850 | --- | down | 1.80 | 4.18E-02 | 6.70 | 5.85 | No |
| *---* | CR603086 | --- | down | 1.80 | 1.44E-02 | 6.14 | 5.29 | No |
| *SMO* | BC009989 | ENSG00000128602 | down | 1.80 | 3.54E-02 | 7.42 | 6.57 | No |
| *C7orf10* | AK299133 | ENSG00000175600 | down | 1.80 | 1.36E-04 | 6.04 | 5.19 | No |
| *---* | BC031092 | --- | down | 1.80 | 4.70E-03 | 6.50 | 5.66 | No |
| *---* | AK022264 | --- | down | 1.79 | 1.28E-03 | 6.76 | 5.92 | No |
| *PKP1* | BC114571 | ENSG00000081277 | down | 1.79 | 6.23E-04 | 7.18 | 6.33 | No |
| *TRPS1* | AF183810 | ENSG00000104447 | down | 1.79 | 9.35E-07 | 7.44 | 6.60 | No |
| *GUCY1B3* | BC047620 | ENSG00000061918 | down | 1.79 | 6.81E-07 | 6.95 | 6.11 | No |
| *VIPR1* | AK056819 | ENSG00000114812 | down | 1.78 | 3.08E-10 | 8.73 | 7.89 | No |
| *---* | AK098245 | --- | down | 1.78 | 3.68E-03 | 6.16 | 5.33 | No |
| *---* | AK124907 | --- | down | 1.78 | 7.42E-04 | 6.48 | 5.65 | No |
| *TXNIP* | BX537824 | ENSG00000117289 | down | 1.78 | 2.05E-02 | 11.65 | 10.82 | No |
| *MPDZ* | AB210041 | ENSG00000107186 | down | 1.78 | 2.80E-02 | 6.38 | 5.55 | No |
| *TMEM140* | AK001862 | ENSG00000146859 | down | 1.78 | 3.72E-02 | 7.51 | 6.68 | No |
| *CSF1R* | X03663 | ENSG00000182578 | down | 1.78 | 5.81E-07 | 9.58 | 8.75 | No |
| *---* | BC030253 | --- | down | 1.77 | 1.69E-02 | 6.02 | 5.20 | Yes |
| *ACSS2* | AK000162 | ENSG00000131069 | down | 1.77 | 2.23E-02 | 9.44 | 8.62 | No |
| *PLEKHG5 // TNFRSF25* | BC036671 | ENSG00000171680 // ENSG00000215788 | down | 1.77 | 2.37E-02 | 8.54 | 7.72 | No |
| *---* | AK123486 | --- | down | 1.77 | 3.86E-02 | 7.04 | 6.21 | No |
| *FCER1A* | X06948 | ENSG00000179639 | down | 1.77 | 3.77E-02 | 6.69 | 5.86 | No |
| *SLAIN1* | AK294201 | ENSG00000139737 | down | 1.77 | 1.65E-05 | 7.93 | 7.11 | No |
| *SHE* | CR936667 | ENSG00000169291 | down | 1.77 | 2.93E-02 | 6.41 | 5.59 | No |
| *BAG2* | AK023735 | ENSG00000112208 | down | 1.77 | 2.56E-06 | 7.28 | 6.45 | No |
| *RBM24* | AK095016 | ENSG00000112183 | down | 1.77 | 2.08E-02 | 6.40 | 5.58 | No |
| *TLR3* | BC094737 | ENSG00000164342 | down | 1.77 | 3.71E-02 | 8.65 | 7.83 | No |
| *---* | AK095925 | --- | down | 1.76 | 2.73E-02 | 6.06 | 5.24 | No |
| *SLC30A4* | BC026089 | ENSG00000104154 | down | 1.76 | 1.06E-03 | 7.67 | 6.86 | No |
| *TSPAN11* | AK293947 | ENSG00000110900 | down | 1.76 | 9.75E-07 | 8.20 | 7.38 | No |
| *---* | AB116553 | --- | down | 1.76 | 9.68E-03 | 5.79 | 4.97 | No |
| *ABP1* | BX648159 | ENSG00000002726 | down | 1.76 | 3.07E-02 | 11.11 | 10.29 | No |
| *SYNE1* | AF495910 | ENSG00000131018 | down | 1.76 | 1.23E-02 | 7.59 | 6.77 | No |
| *---* | BX538191 | --- | down | 1.76 | 2.22E-03 | 6.02 | 5.21 | No |
| *MARCH3* | BC047569 | ENSG00000173926 | down | 1.76 | 3.79E-02 | 9.65 | 8.84 | No |
| *MIER3* | BX648294 | ENSG00000155545 | down | 1.76 | 1.72E-03 | 8.63 | 7.81 | No |
| *ABCC9* | BC172883 | ENSG00000069431 | down | 1.75 | 9.10E-03 | 6.32 | 5.51 | No |
| *---* | BC086865 | --- | down | 1.75 | 1.00E-03 | 6.17 | 5.36 | Yes |
| *GIMAP8* | AL834361 | ENSG00000171115 | down | 1.75 | 1.40E-02 | 6.61 | 5.81 | No |
| *---* | AK098358 | --- | down | 1.75 | 2.88E-02 | 7.16 | 6.35 | No |
| *---* | AK127627 | --- | down | 1.74 | 6.42E-11 | 6.54 | 5.74 | No |
| *GVIN1* | BX538318 | --- | down | 1.74 | 1.30E-02 | 6.38 | 5.58 | No |
| *DDX26B* | BX648113 | ENSG00000165359 | down | 1.74 | 4.52E-03 | 7.02 | 6.22 | No |
| *---* | AK129811 | --- | down | 1.74 | 1.02E-04 | 6.59 | 5.79 | No |
| *---* | CR612038 | --- | down | 1.74 | 4.85E-02 | 6.58 | 5.78 | No |
| *SGCD* | BX537948 | ENSG00000170624 | down | 1.74 | 1.44E-02 | 6.82 | 6.02 | No |
| *---* | AB062480 | --- | down | 1.74 | 2.69E-02 | 5.21 | 4.42 | No |
| *WDR17* | AK098748 | ENSG00000150627 | down | 1.74 | 1.21E-04 | 5.78 | 4.99 | No |
| *---* | AF075056 | --- | down | 1.73 | 3.71E-11 | 6.50 | 5.71 | Yes |
| *PTPRH* | D15049 | ENSG00000080031 | down | 1.73 | 1.20E-03 | 8.53 | 7.74 | No |
| *---* | AK095738 | --- | down | 1.73 | 3.43E-02 | 7.34 | 6.55 | No |
| *---* | AF339795 | --- | down | 1.73 | 3.35E-06 | 6.24 | 5.45 | No |
| *KLRC4 // KLRK1* | AF461811 | ENSG00000183542 // ENSG00000213809 | down | 1.73 | 4.05E-02 | 6.32 | 5.53 | No |
| *---* | AK000819 | --- | down | 1.72 | 2.76E-02 | 7.81 | 7.03 | No |
| *---* | BC047481 | --- | down | 1.72 | 4.36E-02 | 7.29 | 6.51 | No |
| *---* | AK057621 | --- | down | 1.72 | 1.21E-02 | 5.81 | 5.03 | No |
| *FAM7A2* | BC070492 | ENSG00000215312 | down | 1.72 | 1.98E-05 | 6.75 | 5.96 | No |
| *---* | AF075007 | --- | down | 1.72 | 2.33E-02 | 5.56 | 4.78 | No |
| *ROR2* | BC033697 | ENSG00000169071 | down | 1.72 | 1.42E-02 | 7.40 | 6.62 | No |
| *MEOX2* | BC017021 | ENSG00000106511 | down | 1.72 | 3.74E-02 | 7.15 | 6.37 | No |
| *---* | BC040308 | --- | down | 1.72 | 1.48E-03 | 7.66 | 6.88 | No |
| *---* | AK024998 | --- | down | 1.72 | 2.38E-05 | 6.63 | 5.85 | Yes |
| *UST* | AB020316 | ENSG00000111962 | down | 1.72 | 3.41E-02 | 6.77 | 5.99 | No |
| *---* | AL050141 | --- | down | 1.72 | 3.17E-02 | 10.10 | 9.31 | No |
| *---* | AK026659 | --- | down | 1.71 | 1.89E-02 | 7.42 | 6.64 | No |
| *---* | AK022198 | --- | down | 1.71 | 3.79E-02 | 9.32 | 8.54 | No |
| *TTLL6* | AL834151 | ENSG00000170703 | down | 1.71 | 4.50E-02 | 6.95 | 6.18 | No |
| *TMEM220* | AY550194 | ENSG00000187824 | down | 1.71 | 4.85E-02 | 6.32 | 5.54 | No |
| *GLP2R* | AK295765 | ENSG00000065325 | down | 1.71 | 9.24E-04 | 6.53 | 5.76 | No |
| *P2RX7* | BC007679 | ENSG00000089041 | down | 1.71 | 7.60E-03 | 6.46 | 5.69 | No |
| *---* | AK309962 | --- | down | 1.71 | 2.10E-02 | 5.82 | 5.04 | No |
| *VLDLR* | L20470 | ENSG00000147852 | down | 1.71 | 3.80E-02 | 6.50 | 5.73 | No |
| *SLC22A23* | BC128581 | ENSG00000137266 | down | 1.71 | 4.37E-02 | 9.27 | 8.50 | No |
| *---* | AK124401 | --- | down | 1.71 | 4.69E-02 | 6.07 | 5.30 | No |
| *C5orf56* | AK309191 | ENSG00000197536 | down | 1.71 | 1.27E-02 | 8.17 | 7.39 | No |
| *SLC9A9* | BC035779 | ENSG00000181804 | down | 1.70 | 3.02E-02 | 6.69 | 5.92 | No |
| *ZIC5* | AF378304 | ENSG00000139800 | down | 1.70 | 1.67E-02 | 6.91 | 6.14 | No |
| *SLC2A13* | AJ315644 | ENSG00000151229 | down | 1.70 | 3.20E-03 | 8.20 | 7.43 | No |
| *PIK3AP1* | BX648550 | ENSG00000155629 | down | 1.70 | 1.81E-02 | 7.93 | 7.16 | No |
| *SCARA3* | BC060811 | ENSG00000168077 | down | 1.70 | 3.83E-06 | 7.41 | 6.64 | No |
| *HEY2* | AK303890 | ENSG00000135547 | down | 1.70 | 2.70E-02 | 6.46 | 5.69 | No |
| *PCDHB4* | AK308946 | ENSG00000081818 | down | 1.70 | 2.55E-02 | 6.21 | 5.45 | No |
| *---* | AK001007 | --- | down | 1.70 | 1.12E-02 | 6.76 | 6.00 | No |
| *---* | AK094703 | --- | down | 1.69 | 1.10E-02 | 6.47 | 5.72 | No |
| *C1QTNF7* | BX647781 | ENSG00000163145 | down | 1.69 | 1.94E-02 | 6.08 | 5.33 | No |
| *NEGR1* | BX538014 | ENSG00000172260 | down | 1.69 | 2.50E-02 | 6.62 | 5.86 | No |
| *RBMS3* | AK097311 | ENSG00000144642 | down | 1.69 | 4.44E-02 | 6.31 | 5.55 | No |
| *---* | AK022220 | --- | down | 1.69 | 1.02E-02 | 6.81 | 6.06 | Yes |
| *---* | AK128535 | --- | down | 1.69 | 4.86E-02 | 5.80 | 5.05 | No |
| *---* | BC104435 | --- | down | 1.69 | 7.60E-05 | 7.00 | 6.24 | No |
| *---* | AK022008 | --- | down | 1.69 | 2.03E-05 | 8.08 | 7.32 | Yes |
| *MRVI1* | AK127209 | ENSG00000072952 | down | 1.69 | 1.94E-02 | 7.07 | 6.32 | No |
| *PREX2* | AJ437636 | ENSG00000046889 | down | 1.69 | 2.41E-02 | 6.06 | 5.30 | No |
| *---* | AK098461 | --- | down | 1.69 | 3.69E-02 | 6.05 | 5.29 | No |
| *EIF4E3* | BC068443 | ENSG00000163412 | down | 1.68 | 3.96E-02 | 8.13 | 7.38 | No |
| *APOL3* | AY014907 | ENSG00000128284 | down | 1.68 | 2.59E-06 | 7.39 | 6.64 | Yes |
| *---* | AY926481 | --- | down | 1.68 | 1.63E-02 | 6.65 | 5.90 | No |
| *LTBP4* | AF051345 | ENSG00000090006 | down | 1.68 | 1.53E-06 | 9.06 | 8.31 | Yes |
| *PLA2G10* | U95301 | ENSG00000069764 | down | 1.68 | 3.34E-06 | 8.86 | 8.11 | No |
| *---* | AF147388 | --- | down | 1.68 | 2.27E-02 | 5.55 | 4.80 | No |
| *---* | AK025589 | --- | down | 1.68 | 6.15E-04 | 6.22 | 5.47 | No |
| *---* | AK023481 | --- | down | 1.68 | 8.20E-03 | 7.91 | 7.16 | No |
| *ARHGAP20* | AY496267 | ENSG00000137727 | down | 1.68 | 2.98E-02 | 6.17 | 5.42 | No |
| *AMOTL1* | AK074084 | ENSG00000166025 | down | 1.68 | 1.13E-02 | 7.18 | 6.43 | No |
| *FNBP1* | AB011126 | ENSG00000187239 | down | 1.68 | 4.04E-02 | 8.19 | 7.44 | No |
| *PDLIM2* | AY217349 | ENSG00000120913 | down | 1.68 | 1.09E-02 | 7.64 | 6.89 | No |
| *HLA-DPA1* | CR617728 | ENSG00000206291 // ENSG00000231389 | down | 1.68 | 9.95E-07 | 11.44 | 10.69 | No |
| *---* | AK096549 | --- | down | 1.68 | 7.68E-03 | 6.62 | 5.87 | No |
| *DOCK2* | D86964 | ENSG00000134516 | down | 1.68 | 1.16E-06 | 7.45 | 6.70 | No |
| *SLC22A5* | AF057164 | ENSG00000197375 | down | 1.68 | 4.60E-03 | 9.05 | 8.29 | No |
| *CD160* | AK128370 | ENSG00000117281 | down | 1.68 | 1.26E-03 | 6.38 | 5.63 | No |
| *---* | BC039540 | --- | down | 1.68 | 3.52E-02 | 8.64 | 7.89 | No |
| *RBM38* | AF432218 | ENSG00000132819 | down | 1.67 | 2.55E-02 | 8.09 | 7.35 | No |
| *DEGS1* | CR623303 | ENSG00000143753 | down | 1.67 | 2.44E-05 | 8.25 | 7.51 | No |
| *SLC26A10* | AK057416 | ENSG00000135502 | down | 1.67 | 2.69E-02 | 6.69 | 5.94 | No |
| *EGLN1* | AF229245 | ENSG00000135766 | down | 1.67 | 1.92E-02 | 9.52 | 8.77 | No |
| *---* | AK023631 | --- | down | 1.67 | 2.92E-02 | 6.16 | 5.42 | No |
| *HLA-DPB1* | CR602959 | ENSG00000223865 // ENSG00000236693 | down | 1.67 | 1.51E-05 | 11.13 | 10.39 | Yes |
| *SLC36A1* | AK057340 | ENSG00000123643 | down | 1.67 | 1.10E-02 | 8.66 | 7.92 | No |
| *---* | AL049449 | --- | down | 1.67 | 3.57E-02 | 6.18 | 5.44 | Yes |
| *CLCN2* | BC072004 | ENSG00000114859 | down | 1.66 | 1.41E-02 | 7.64 | 6.91 | No |
| *PLSCR4* | BC028354 | ENSG00000114698 | down | 1.66 | 3.34E-02 | 7.76 | 7.03 | No |
| *SLC25A23* | BC001656 | ENSG00000125648 | down | 1.66 | 4.26E-03 | 8.28 | 7.54 | No |
| *C14orf49* | BC146604 | ENSG00000176438 | down | 1.66 | 3.00E-03 | 7.03 | 6.30 | No |
| *FLVCR2* | AY260577 | ENSG00000119686 | down | 1.66 | 1.31E-07 | 7.45 | 6.72 | No |
| *---* | BC036614 | --- | down | 1.66 | 2.79E-02 | 6.52 | 5.78 | No |
| *---* | AK024580 | --- | down | 1.66 | 4.61E-02 | 6.10 | 5.37 | No |
| *---* | AF190162 | --- | down | 1.66 | 5.98E-03 | 5.95 | 5.22 | No |
| *FAM163B* | BC146961 | ENSG00000196990 | down | 1.66 | 6.63E-03 | 7.07 | 6.35 | No |
| *---* | GU014834 | --- | down | 1.66 | 4.14E-02 | 8.03 | 7.30 | No |
| *ATP13A4* | AY823162 | ENSG00000127249 | down | 1.65 | 4.89E-02 | 6.83 | 6.11 | No |
| *PPP2R3A* | L07590 | ENSG00000073711 | down | 1.65 | 2.31E-02 | 6.50 | 5.78 | No |
| *RETSAT* | AK075261 | ENSG00000042445 | down | 1.65 | 8.14E-03 | 8.98 | 8.25 | No |
| *C1orf115* | AK125403 | ENSG00000162817 | down | 1.65 | 3.50E-02 | 7.99 | 7.27 | No |
| *---* | AY726599 | --- | down | 1.65 | 2.17E-02 | 9.42 | 8.70 | No |
| *STON2* | AB208948 | ENSG00000140022 | down | 1.65 | 1.09E-02 | 6.77 | 6.05 | No |
| *---* | BC148183 | --- | down | 1.65 | 5.78E-03 | 6.10 | 5.39 | No |
| *ANK3* | BX648574 | ENSG00000151150 | down | 1.65 | 3.97E-02 | 8.23 | 7.50 | No |
| *QRFP* | BC101128 | ENSG00000188710 | down | 1.65 | 1.13E-02 | 8.88 | 8.16 | No |
| *HOXA11AS* | BC025338 | ENSG00000240990 | down | 1.65 | 3.70E-02 | 7.50 | 6.77 | No |
| *BRP44L* | AF151887 | ENSG00000060762 | down | 1.65 | 1.63E-02 | 9.05 | 8.33 | No |
| *FAM8A1* | AF097027 | ENSG00000137414 | down | 1.65 | 3.40E-02 | 8.89 | 8.16 | Yes |
| *BCAR3* | BC028477 | ENSG00000137936 | down | 1.64 | 2.34E-03 | 7.85 | 7.14 | No |
| *---* | BC035193 | --- | down | 1.64 | 3.20E-02 | 6.68 | 5.97 | No |
| *---* | AX748190 | --- | down | 1.64 | 1.98E-02 | 6.60 | 5.89 | No |
| *STYK1* | AF251059 | ENSG00000060140 | down | 1.64 | 1.38E-02 | 7.12 | 6.41 | No |
| *ADC* | AK095127 | ENSG00000142920 | down | 1.64 | 1.98E-03 | 7.23 | 6.52 | No |
| *CASD1* | BC063284 | ENSG00000127995 | down | 1.64 | 4.32E-02 | 7.93 | 7.21 | No |
| *CNKSR3* | AY328891 | ENSG00000153721 | down | 1.64 | 2.42E-02 | 7.50 | 6.79 | No |
| *MAN1C1* | AK128560 | ENSG00000117643 | down | 1.64 | 5.68E-03 | 7.14 | 6.42 | No |
| *---* | AL117656 | --- | down | 1.64 | 3.04E-02 | 6.56 | 5.85 | Yes |
| *ABCA5 // ABCA10* | AY247065 | ENSG00000154263 // ENSG00000154265 | down | 1.63 | 3.88E-02 | 6.50 | 5.80 | No |
| *SLFN12L* | AK172761 | ENSG00000205045 | down | 1.63 | 2.32E-02 | 6.72 | 6.01 | No |
| *---* | AY358241 | --- | down | 1.63 | 4.50E-03 | 6.06 | 5.36 | No |
| *KCNMB4* | CR613566 | ENSG00000135643 | down | 1.63 | 1.26E-03 | 7.49 | 6.79 | No |
| *---* | AK094737 | --- | down | 1.63 | 4.41E-02 | 7.68 | 6.98 | No |
| *HOXA2* | BC136500 | ENSG00000105996 | down | 1.63 | 3.84E-02 | 6.99 | 6.29 | No |
| *COL1A2* | J03464 | --- | down | 1.63 | 2.21E-04 | 8.55 | 7.85 | No |
| *NBPF11* | AL832622 | ENSG00000152042 // ENSG00000203836 | down | 1.63 | 2.99E-02 | 8.80 | 8.09 | Yes |
| *---* | BC022048 | --- | down | 1.63 | 2.24E-04 | 6.02 | 5.31 | No |
| *CAMK2N2* | AY037149 | ENSG00000163888 | down | 1.62 | 8.68E-03 | 9.22 | 8.52 | Yes |
| *---* | AK095172 | --- | down | 1.62 | 2.86E-02 | 6.71 | 6.02 | No |
| *ASAP3* | BC023519 | ENSG00000088280 | down | 1.62 | 4.72E-02 | 7.09 | 6.40 | No |
| *---* | AK097810 | --- | down | 1.62 | 1.35E-04 | 6.71 | 6.02 | Yes |
| *---* | AF075022 | --- | down | 1.62 | 4.66E-02 | 5.92 | 5.22 | No |
| *---* | BC042520 | --- | down | 1.62 | 5.92E-03 | 6.99 | 6.29 | No |
| *---* | CR627426 | --- | down | 1.62 | 1.60E-02 | 6.34 | 5.64 | No |
| *---* | AK055118 | --- | down | 1.62 | 7.30E-03 | 5.69 | 4.99 | Yes |
| *PTGDS* | BC041463 | ENSG00000107317 | down | 1.62 | 1.25E-02 | 8.72 | 8.03 | Yes |
| *AGPAT9* | DQ345298 | ENSG00000138678 | down | 1.61 | 4.37E-02 | 7.00 | 6.32 | No |
| *PARM1* | AK022311 | ENSG00000169116 | down | 1.61 | 2.02E-02 | 11.12 | 10.43 | No |
| *---* | AK123104 | --- | down | 1.61 | 3.96E-03 | 5.89 | 5.20 | No |
| *SIRPA* | AB023430 | ENSG00000198053 | down | 1.61 | 6.06E-05 | 8.32 | 7.63 | Yes |
| *---* | CR626349 | --- | down | 1.61 | 4.50E-03 | 6.73 | 6.04 | No |
| *---* | J05550 | --- | down | 1.61 | 1.31E-02 | 6.31 | 5.63 | No |
| *IGSF9* | AY358128 | ENSG00000085552 | down | 1.61 | 1.52E-02 | 7.54 | 6.85 | No |
| *MT1P3* | BC133653 | --- | down | 1.60 | 1.57E-04 | 10.88 | 10.20 | No |
| *---* | BC131754 | --- | down | 1.60 | 5.92E-03 | 6.75 | 6.08 | No |
| *PNMA1* | AF037364 | ENSG00000176903 | down | 1.60 | 3.58E-02 | 7.30 | 6.63 | No |
| *KLRG1* | BC012621 | ENSG00000139187 | down | 1.60 | 2.83E-02 | 6.45 | 5.77 | No |
| *RGS10* | AK290773 | ENSG00000148908 | down | 1.60 | 4.48E-09 | 8.16 | 7.49 | No |
| *CALHM2* | AK022195 | ENSG00000138172 | down | 1.60 | 2.54E-02 | 8.28 | 7.60 | No |
| *BTK* | AK309851 | ENSG00000010671 | down | 1.60 | 9.35E-08 | 6.31 | 5.63 | No |
| *OPTN* | AK055403 | ENSG00000123240 | down | 1.60 | 1.68E-02 | 8.09 | 7.41 | No |
| *---* | AF261143 | --- | down | 1.60 | 3.93E-02 | 6.79 | 6.11 | No |
| *VSTM2A* | CR613464 | ENSG00000170419 | down | 1.60 | 9.43E-07 | 7.98 | 7.30 | No |
| *---* | AK123506 | --- | down | 1.59 | 1.53E-02 | 10.23 | 9.56 | No |
| *CECR1* | AK304818 | ENSG00000093072 | down | 1.59 | 3.17E-04 | 7.94 | 7.27 | No |
| *TIAM1* | U16296 | ENSG00000156299 | down | 1.59 | 1.18E-02 | 6.66 | 5.99 | No |
| *---* | BC169317 | --- | down | 1.59 | 3.60E-02 | 7.88 | 7.21 | No |
| *RMND5A* | BC047668 | ENSG00000153561 | down | 1.59 | 4.67E-02 | 8.66 | 7.99 | No |
| *FCGBP* | D84239 | ENSG00000090920 | down | 1.59 | 2.68E-03 | 11.03 | 10.36 | No |
| *---* | AF086240 | --- | down | 1.59 | 4.46E-02 | 6.46 | 5.79 | No |
| *ANKRD12* | AY373757 | ENSG00000101745 | down | 1.59 | 3.24E-02 | 8.94 | 8.27 | No |
| *GLTP* | AY372530 | ENSG00000139433 | down | 1.59 | 1.83E-08 | 9.30 | 8.63 | No |
| *---* | AF090887 | --- | down | 1.59 | 3.90E-02 | 5.92 | 5.25 | No |
| *DAAM2* | AB002379 | ENSG00000146122 | down | 1.59 | 2.07E-07 | 7.02 | 6.34 | No |
| *ZBTB20* | AK225788 | ENSG00000181722 | down | 1.58 | 2.74E-02 | 8.31 | 7.65 | No |
| *TCF7L1* | AB031046 | ENSG00000152284 | down | 1.58 | 3.08E-02 | 7.44 | 6.77 | No |
| *---* | AK127502 | --- | down | 1.58 | 8.36E-03 | 6.64 | 5.98 | No |
| *---* | AK126118 | --- | down | 1.58 | 3.96E-02 | 5.90 | 5.24 | No |
| *---* | AK092342 | --- | down | 1.58 | 4.88E-02 | 6.06 | 5.40 | No |
| *NXPH3* | AK308928 | ENSG00000182575 | down | 1.58 | 1.22E-02 | 7.08 | 6.42 | No |
| *DYRK2* | Y13493 | ENSG00000127334 | down | 1.58 | 1.04E-02 | 9.01 | 8.36 | No |
| *ATOH1* | EU446465 | ENSG00000172238 | down | 1.57 | 4.68E-03 | 10.84 | 10.20 | No |
| *---* | AK128410 | --- | down | 1.57 | 2.62E-03 | 6.15 | 5.49 | No |
| *---* | AK130945 | --- | down | 1.57 | 3.67E-02 | 6.16 | 5.51 | No |
| *---* | BC026124 | --- | down | 1.57 | 8.90E-03 | 6.29 | 5.64 | No |
| *AAK1* | AK056043 | ENSG00000115977 | down | 1.57 | 1.76E-08 | 9.43 | 8.77 | No |
| *---* | L23850 | --- | down | 1.57 | 3.36E-02 | 5.39 | 4.74 | Yes |
| *NBEAL1* | AK127772 | ENSG00000144426 | down | 1.57 | 3.18E-02 | 7.88 | 7.22 | No |
| *---* | AB082533 | --- | down | 1.57 | 1.33E-02 | 8.80 | 8.15 | No |
| *APPL2* | AY113704 | ENSG00000136044 | down | 1.57 | 9.08E-03 | 9.02 | 8.36 | No |
| *---* | AK096048 | --- | down | 1.57 | 2.93E-02 | 6.22 | 5.56 | No |
| *---* | AK098218 | --- | down | 1.57 | 1.31E-02 | 6.03 | 5.37 | Yes |
| *AKAP9* | AJ131693 | ENSG00000127914 | down | 1.57 | 3.87E-02 | 8.38 | 7.72 | No |
| *FAM162B* | BC038997 | ENSG00000183807 | down | 1.57 | 6.68E-03 | 7.39 | 6.74 | Yes |
| *---* | AF380582 | --- | down | 1.57 | 1.12E-02 | 12.17 | 11.52 | Yes |
| *DSC2* | BC063291 | ENSG00000134755 | down | 1.56 | 4.21E-02 | 10.67 | 10.03 | No |
| *SETBP1* | BC146776 | ENSG00000152217 | down | 1.56 | 2.98E-03 | 7.32 | 6.68 | No |
| *---* | AK055340 | --- | down | 1.56 | 1.24E-02 | 5.88 | 5.24 | No |
| *NUPR1* | AF069073 | ENSG00000176046 | down | 1.56 | 2.56E-03 | 10.16 | 9.51 | Yes |
| *---* | BC040875 | --- | down | 1.56 | 3.06E-02 | 5.98 | 5.34 | Yes |
| *RAB27A* | AK312836 | ENSG00000069974 | down | 1.56 | 4.97E-02 | 8.42 | 7.78 | No |
| *SCAND2* | BC063836 | ENSG00000176700 | down | 1.56 | 1.50E-03 | 6.98 | 6.34 | No |
| *CTSF* | AF136279 | ENSG00000174080 | down | 1.56 | 3.23E-02 | 6.52 | 5.88 | No |
| *LY6E* | CR597286 | ENSG00000160932 | down | 1.56 | 1.08E-03 | 8.31 | 7.67 | Yes |
| *TEF* | AK091916 | ENSG00000167074 | down | 1.55 | 1.29E-02 | 7.03 | 6.40 | No |
| *DEFB121* | AF479700 | ENSG00000204548 | down | 1.55 | 4.42E-03 | 6.07 | 5.44 | No |
| *PDE1A* | AK294239 | ENSG00000115252 | down | 1.55 | 1.93E-02 | 5.98 | 5.34 | No |
| *CLIP4* | AF433661 | ENSG00000115295 | down | 1.55 | 4.60E-02 | 6.17 | 5.54 | No |
| *CCL14 // CCL15* | Z70293 | ENSG00000161574 // ENSG00000213494 | down | 1.55 | 4.83E-02 | 9.19 | 8.55 | No |
| *SMPD3* | AK289651 | ENSG00000103056 | down | 1.55 | 1.27E-02 | 8.00 | 7.36 | No |
| *FOXF1* | U13219 | ENSG00000103241 | down | 1.55 | 2.64E-04 | 8.06 | 7.43 | No |
| *EEF2K* | AK308757 | ENSG00000103319 | down | 1.55 | 7.64E-04 | 8.04 | 7.41 | No |
| *TSPAN3* | AK001326 | ENSG00000140391 | down | 1.55 | 2.05E-02 | 11.08 | 10.45 | No |
| *RBPMS2* | AK127873 | ENSG00000166831 | down | 1.55 | 1.75E-02 | 7.47 | 6.84 | No |
| *---* | AK129925 | --- | down | 1.55 | 4.69E-02 | 5.79 | 5.16 | Yes |
| *C10orf10* | AB022718 | ENSG00000165507 | down | 1.55 | 4.70E-02 | 7.61 | 6.97 | No |
| *SRGN* | CR626092 | ENSG00000122862 | down | 1.55 | 2.98E-03 | 10.06 | 9.43 | No |
| *---* | AK123238 | --- | down | 1.55 | 2.96E-02 | 5.96 | 5.32 | No |
| *---* | BC010432 | --- | down | 1.55 | 8.50E-05 | 7.57 | 6.95 | No |
| *FRAS1* | BC131821 | ENSG00000138759 | down | 1.54 | 3.33E-02 | 6.45 | 5.82 | No |
| *---* | AK025205 | --- | down | 1.54 | 5.02E-07 | 8.95 | 8.32 | Yes |
| *COX6B2 // FAM71E2* | AK128284 | ENSG00000160471 // ENSG00000180043 | down | 1.54 | 7.92E-03 | 8.08 | 7.46 | Yes |
| *ATP2B1* | J04027 | ENSG00000070961 | down | 1.54 | 1.46E-02 | 9.74 | 9.12 | No |
| *PCSK1N* | CR605647 | ENSG00000102109 | down | 1.54 | 9.98E-03 | 8.63 | 8.00 | No |
| *TCEAL3* | BC008703 | ENSG00000196507 | down | 1.54 | 4.55E-02 | 7.18 | 6.56 | Yes |
| *MT1P2* | AF333388 | ENSG00000162840 // ENSG00000244020 | down | 1.54 | 1.70E-03 | 11.55 | 10.92 | Yes |
| *XCR1* | AK314277 | ENSG00000173578 | down | 1.53 | 2.21E-02 | 7.03 | 6.41 | No |
| *CBX7* | BC051773 | ENSG00000100307 | down | 1.53 | 5.80E-03 | 8.06 | 7.45 | No |
| *LARGE* | AK295636 | ENSG00000133424 | down | 1.53 | 4.03E-12 | 8.36 | 7.74 | No |
| *ABCC13* | AF418600 | ENSG00000243064 | down | 1.53 | 3.21E-02 | 5.68 | 5.07 | No |
| *MAL* | BC003006 | ENSG00000172005 | down | 1.53 | 7.50E-03 | 6.77 | 6.15 | No |
| *ATP2C2* | BX648333 | ENSG00000064270 | down | 1.53 | 1.82E-05 | 10.16 | 9.55 | No |
| *---* | BC160029 | --- | down | 1.53 | 2.37E-07 | 8.22 | 7.60 | Yes |
| *RCSD1* | AF545852 | ENSG00000198771 | down | 1.53 | 4.48E-02 | 7.15 | 6.53 | No |
| *---* | X61123 | --- | down | 1.53 | 2.93E-02 | 9.77 | 9.15 | No |
| *MICALCL* | BC168365 | ENSG00000133808 | down | 1.53 | 2.18E-04 | 6.52 | 5.91 | No |
| *STOM* | CR623514 | ENSG00000148175 | down | 1.53 | 1.60E-03 | 9.65 | 9.04 | No |
| *---* | AK309314 | --- | down | 1.52 | 1.45E-02 | 6.29 | 5.68 | No |
| *FLNB* | AF043045 | ENSG00000136068 | down | 1.52 | 2.47E-02 | 10.18 | 9.58 | No |
| *CCBP2* | BC018716 | ENSG00000144648 | down | 1.52 | 1.90E-03 | 6.47 | 5.86 | No |
| *---* | BC033170 | --- | down | 1.52 | 4.86E-02 | 6.25 | 5.65 | Yes |
| *ADCY9* | BC151229 | ENSG00000162104 | down | 1.52 | 3.50E-03 | 7.52 | 6.92 | No |
| *SYTL4* | BX537410 | ENSG00000102362 | down | 1.52 | 1.02E-02 | 7.22 | 6.61 | No |
| *RARRES2* | CR591132 | ENSG00000106538 | down | 1.52 | 1.42E-04 | 7.38 | 6.77 | No |
| *---* | AK091967 | --- | down | 1.51 | 4.43E-02 | 6.26 | 5.67 | No |
| *CD209* | AK293089 | ENSG00000090659 | down | 1.51 | 1.12E-06 | 7.39 | 6.79 | Yes |
| *EVL* | BC023997 | ENSG00000196405 | down | 1.51 | 3.86E-02 | 7.10 | 6.51 | No |
| *P2RX4* | U83993 | ENSG00000135124 | down | 1.51 | 8.21E-07 | 8.94 | 8.34 | No |
| *ACACB* | AY382667 | ENSG00000076555 | down | 1.51 | 1.75E-02 | 7.61 | 7.02 | No |
| *---* | AK130760 | --- | down | 1.51 | 8.30E-03 | 6.22 | 5.63 | No |
| *FRMD1* | AK125963 | ENSG00000153303 | down | 1.51 | 1.17E-02 | 7.18 | 6.59 | No |
| *---* | AX747750 | --- | down | 1.51 | 1.34E-02 | 5.99 | 5.40 | No |
| *NBPF15* | AK097180 | ENSG00000243452 | down | 1.51 | 3.26E-02 | 9.90 | 9.30 | Yes |
| *---* | BX641108 | --- | down | 1.50 | 2.04E-02 | 9.78 | 9.19 | No |
| *SDPR* | BC016475 | ENSG00000168497 | down | 1.50 | 3.05E-02 | 7.24 | 6.66 | No |
| *---* | CR623291 | --- | down | 1.50 | 1.40E-02 | 6.76 | 6.17 | Yes |
| *TENC1* | AF417490 | ENSG00000111077 | down | 1.50 | 4.49E-02 | 8.02 | 7.43 | No |
| *MAP6* | AK123340 | ENSG00000171533 | down | 1.50 | 4.05E-02 | 6.14 | 5.55 | No |
