# Supplementary material for: A Gene Expression and Pre-mRNA Splicing Signature That Marks the Adenoma-Adenocarcinoma Progression in Colorectal Cancer
Source: PLoS One. 2014 Feb 6;9(2):e87761. doi: 10.1371/journal.pone.0087761 (PMC3916340; doi:10.1371/journal.pone.0087761)
Supplement: Table S13 — List of the up- and down-regulated exons in colorectal adenomas in comparison with normal mucosae on Affymetrix Human Exon 1.0 ST arrays. (DOC) [file pone.0087761.s019.doc]

**Table S13. List of the up- and down-regulated exons in colorectal adenomas in comparison with normal mucosae on Affymetrix Human Exon 1.0 ST arrays.** The results of Human Exon 1.0 ST (Affymetrix) for the deregulated exons from genes deregulated or not in CRA *vs*. NOR are presented (≥ 1.5 FC, P-value ≤ 0.05 by *t*-test).

| Gene Symbol | Representative Transcript ID | EnsEMBL ID | Detected Exon | Known Alternative Event | Regulation Splicing Index | Fold-Change Splicing Index | P-Value Splicing Index | Regulation Gene | Fold-Change Gene | P-value Gene | Low-specificity Probe Included? |
| --- | --- | --- | --- | --- | --- | --- | --- | --- | --- | --- | --- |
| *---* | BC029465 | --- | e2 | Unknown | up | 2.88 | 1.10E-03 | up | 2.17 | 3.25E-02 | Yes |
| *KRT6C* | BC110639 | ENSG00000170465 | e8 | Unknown | up | 2.69 | 1.69E-12 | up | 1.91 | 3.79E-05 | Yes |
| *KRT6C* | BC110639 | ENSG00000170465 | e4 | Unknown | up | 2.43 | 1.24E-03 | up | 1.91 | 3.79E-05 | Yes |
| *RNF39* | AF238315 | ENSG00000204618 | ae4 | intron_retention,alternative_last_exon | up | 2.32 | 4.63E-04 | up | 1.68 | 3.72E-02 | No |
| *KIAA1797* | AY139834 | ENSG00000188352 | e37 | Unknown | up | 2.15 | 4.99E-06 | up | 1.53 | 3.96E-02 | No |
| *TBX3* | AF216750 | ENSG00000135111 | e3 | exon_skipping | up | 1.98 | 3.21E-04 | up | 2.30 | 4.29E-02 | No |
| *DPEP1* | CR620135 | ENSG00000015413 | e11 | Unknown | up | 1.92 | 3.46E-03 | up | 9.23 | 6.33E-08 | No |
| *KIAA1199* | AY585237 | ENSG00000103888 | e7 | Unknown | up | 1.82 | 6.36E-05 | up | 4.57 | 2.52E-03 | No |
| *KIAA1797* | AY139834 | ENSG00000188352 | e34 | Unknown | up | 1.80 | 1.36E-03 | up | 1.53 | 3.96E-02 | No |
| *KIAA1199* | AY585237 | ENSG00000103888 | e10 | Unknown | up | 1.76 | 1.72E-03 | up | 4.57 | 2.52E-03 | No |
| *NKD1* | BC051288 | ENSG00000140807 | e9 | Unknown | up | 1.76 | 3.25E-02 | up | 4.00 | 2.32E-02 | No |
| *KIAA1199* | AY585237 | ENSG00000103888 | e15 | Unknown | up | 1.74 | 7.59E-05 | up | 4.57 | 2.52E-03 | No |
| *KIAA1199* | AY585237 | ENSG00000103888 | e14 | Unknown | up | 1.73 | 6.25E-07 | up | 4.57 | 2.52E-03 | No |
| *FDXR* | BC011521 | ENSG00000161513 | e14 | Unknown | up | 1.72 | 1.70E-02 | up | 1.52 | 1.55E-02 | No |
| *OLFM4* | AK000683 | ENSG00000102837 | ae1 | intron_retention,alternative_first_exon | up | 1.67 | 8.30E-03 | up | 9.36 | 1.25E-11 | No |
| *WDR3* | AF083217 | ENSG00000065183 | e5 | exon_skipping | up | 1.64 | 4.36E-02 | up | 1.65 | 1.80E-02 | No |
| *KIAA1199* | AY585237 | ENSG00000103888 | e16 | Unknown | up | 1.61 | 1.79E-05 | up | 4.57 | 2.52E-03 | No |
| *ZNRF3* | AB051436 | ENSG00000183579 | e5 | Unknown | up | 1.60 | 2.96E-03 | up | 1.66 | 3.91E-02 | No |
| *URB1* | AF231919 | ENSG00000142207 | e7 | Unknown | up | 1.59 | 2.51E-02 | up | 1.52 | 3.11E-02 | Yes |
| *ADAM32* | AK300148 | ENSG00000197140 | e14 | alternative_first_exon | up | 1.58 | 2.43E-04 | up | 1.67 | 3.98E-02 | No |
| *KIAA1199* | AY585237 | ENSG00000103888 | e21 | exon_skipping | up | 1.56 | 7.80E-05 | up | 4.57 | 2.52E-03 | No |
| *KIAA1199* | AY585237 | ENSG00000103888 | e28 | exon_skipping | up | 1.55 | 1.87E-05 | up | 4.57 | 2.52E-03 | No |
| *OGFOD1* | AB046832 | ENSG00000087263 | e9 | Unknown | up | 1.55 | 8.00E-03 | up | 1.58 | 2.56E-03 | No |
| *TCF7* | BC048769 | ENSG00000081059 | e2 | Unknown | up | 1.55 | 2.34E-02 | up | 1.79 | 2.95E-02 | No |
| *ATOH8* | AK096049 | ENSG00000168874 | e3 | Unknown | up | 1.55 | 3.36E-03 | up | 2.06 | 4.32E-02 | No |
| *KIAA1199* | AY585237 | ENSG00000103888 | e27 | exon_skipping | up | 1.54 | 7.02E-03 | up | 4.57 | 2.52E-03 | No |
| *CYP4X1* | AK131355 | ENSG00000186377 | e7 | Unknown | up | 1.54 | 4.28E-03 | up | 3.59 | 3.39E-02 | No |
| *KRT6C* | BC110639 | ENSG00000170465 | e9 | alternative_last_exon | up | 1.53 | 5.81E-12 | up | 1.91 | 3.79E-05 | Yes |
| *FASN* | U29344 | ENSG00000169710 | e36 | exon_skipping | up | 1.53 | 1.86E-03 | up | 1.76 | 1.17E-02 | No |
| *SPNS2* | BC041772 | ENSG00000183018 | e7 | Unknown | up | 1.52 | 3.20E-03 | up | 1.78 | 5.98E-07 | No |
| *KRT6C* | BC110639 | ENSG00000170465 | e2 | Unknown | up | 1.52 | 8.12E-03 | up | 1.91 | 3.79E-05 | Yes |
| *ZNRF3* | AB051436 | ENSG00000183579 | e7 | Unknown | up | 1.51 | 1.64E-02 | up | 1.66 | 3.91E-02 | No |
| *LGR6* | AB049405 | ENSG00000133067 | e5 | Unknown | up | 1.50 | 8.66E-03 | up | 2.50 | 5.41E-10 | No |
| *KIAA1797* | AY139834 | ENSG00000188352 | e8 | Unknown | up | 1.50 | 1.19E-02 | up | 1.53 | 3.96E-02 | No |
| *KLK6* | AY279383 | ENSG00000167755 | e2 | alternative_first_exon | down | 5.61 | 1.18E-02 | up | 5.36 | 1.32E-09 | Yes |
| *FIGF // PIR* | AX740234 | ENSG00000087842 // ENSG00000165197 | e18 | Unknown | down | 4.42 | 1.56E-03 | up | 1.72 | 2.49E-02 | No |
| *FIGF // PIR* | AX740234 | ENSG00000087842 // ENSG00000165197 | e15 | Unknown | down | 4.02 | 1.34E-03 | up | 1.72 | 2.49E-02 | No |
| *PLCB4* | AK307837 | ENSG00000101333 | e3 | exon_skipping,alternative_first_exon | down | 3.48 | 2.18E-03 | up | 3.18 | 2.56E-03 | No |
| *PLCB4* | AK307837 | ENSG00000101333 | e1 | alternative_first_exon | down | 3.29 | 1.89E-02 | up | 3.18 | 2.56E-03 | Yes |
| *FIGF // PIR* | AX740234 | ENSG00000087842 // ENSG00000165197 | e16 | Unknown | down | 3.15 | 1.54E-03 | up | 1.72 | 2.49E-02 | No |
| *GPR56* | AK131550 | ENSG00000205336 | e3 | exon_skipping,alternative_first_exon | down | 2.82 | 1.76E-06 | up | 2.18 | 1.00E-04 | No |
| *ANXA3* | CR601701 | ENSG00000138772 | e3 | exon_skipping | down | 2.77 | 2.78E-03 | up | 2.26 | 5.56E-03 | No |
| *GSTP1* | BC010915 | ENSG00000084207 | e1 | alternative_first_exon | down | 2.65 | 2.91E-06 | up | 2.63 | 2.23E-07 | No |
| *DPEP1* | CR620135 | ENSG00000015413 | e7 | Unknown | down | 2.57 | 6.38E-03 | up | 9.23 | 6.33E-08 | No |
| *SPATA20* | AK125807 | ENSG00000006282 | e1 | alternative_first_exon | down | 2.53 | 7.44E-04 | up | 2.15 | 7.01E-04 | No |
| *TMEM9* | AY359012 | ENSG00000116857 | ae3 | alternative_donor_splice_site | down | 2.49 | 7.54E-03 | up | 1.64 | 1.79E-02 | No |
| *TBXAS1* | BC014117 | ENSG00000059377 | e4 | Unknown | down | 2.42 | 6.20E-03 | up | 2.07 | 2.35E-02 | Yes |
| *DPEP1* | CR620135 | ENSG00000015413 | e3 | exon_skipping,alternative_first_exon | down | 2.41 | 5.96E-04 | up | 9.23 | 6.33E-08 | No |
| *TBC1D14* | BC041167 | ENSG00000132405 | ae8 | alternative_acceptor_splice_site | down | 2.41 | 2.36E-02 | up | 1.60 | 1.20E-02 | Yes |
| *FBXO17 // SARS2* | AK301903 | ENSG00000104835 | e5 | Unknown | down | 2.33 | 3.51E-12 | up | 1.65 | 9.76E-03 | No |
| *FBXO17 // SARS2* | AK301903 | ENSG00000104835 | e1 | alternative_first_exon | down | 2.28 | 1.38E-02 | up | 1.65 | 9.76E-03 | Yes |
| *NKD1* | BC051288 | ENSG00000140807 | e8 | Unknown | down | 2.28 | 7.00E-03 | up | 4.00 | 2.32E-02 | No |
| *SHMT2* | BC091501 | ENSG00000182199 | e2 | exon_skipping,alternative_first_exon | down | 2.24 | 1.38E-03 | up | 1.65 | 2.98E-03 | No |
| *SLC2A8* | BC019043 | ENSG00000136856 | ae7 | intron_retention | down | 2.23 | 1.26E-11 | up | 1.90 | 3.42E-03 | Yes |
| *BAX* | AK291076 | ENSG00000087088 | e5 | exon_skipping | down | 2.22 | 1.61E-02 | up | 2.02 | 3.34E-03 | No |
| *AGMAT* | AK130034 | ENSG00000116771 | e8 | alternative_last_exon | down | 2.22 | 1.85E-02 | up | 1.65 | 3.59E-02 | Yes |
| *TRIM16L* | AK293428 | ENSG00000108448 | ae7 | alternative_first_exon | down | 2.20 | 9.92E-03 | up | 1.89 | 4.44E-03 | Yes |
| *PLCB4* | AK307837 | ENSG00000101333 | e4 | Unknown | down | 2.18 | 3.96E-02 | up | 3.18 | 2.56E-03 | No |
| *DPEP1* | CR620135 | ENSG00000015413 | e8 | Unknown | down | 2.17 | 5.86E-03 | up | 9.23 | 6.33E-08 | No |
| *PHTF1* | AJ011863 | ENSG00000116793 | ae14 | alternative_last_exon | down | 2.16 | 6.54E-03 | up | 1.63 | 3.65E-02 | No |
| *KLK6* | AY279383 | ENSG00000167755 | e5 | exon_skipping | down | 2.14 | 3.96E-03 | up | 5.36 | 1.32E-09 | No |
| *CD68 // EIF4A1 // SENP3* | BC032613 | ENSG00000129226 // ENSG00000161956 // ENSG00000161960 | e28 | Unknown | down | 2.14 | 2.24E-03 | up | 1.83 | 3.76E-05 | No |
| *CDRT1 // TRIM16* | AB209899 | ENSG00000221926 // ENSG00000241322 | e17 | Unknown | down | 2.14 | 6.76E-03 | up | 1.72 | 4.58E-03 | Yes |
| *TMEM177* | AK057313 | ENSG00000144120 | e3 | alternative_last_exon | down | 2.14 | 1.74E-02 | up | 1.53 | 4.48E-02 | No |
| *KIAA1199* | AY585237 | ENSG00000103888 | e19 | Unknown | down | 2.13 | 2.14E-03 | up | 4.56 | 2.26E-03 | Yes |
| *TMEM177* | AK057313 | ENSG00000144120 | e12 | Unknown | down | 2.13 | 3.96E-02 | up | 1.53 | 4.48E-02 | No |
| *CD68 // EIF4A1 // SENP3* | BC032613 | ENSG00000129226 // ENSG00000161956 // ENSG00000161960 | e26 | Unknown | down | 2.12 | 5.98E-03 | up | 1.83 | 3.76E-05 | No |
| *TMEM184B* | BC015489 | ENSG00000198792 | e4 | exon_skipping | down | 2.09 | 3.54E-03 | up | 1.59 | 1.90E-03 | No |
| *KLK11* | BC022068 | ENSG00000167757 | e7 | Unknown | down | 2.08 | 8.41E-05 | up | 6.84 | 9.18E-04 | No |
| *AZGP1* | BC033830 | ENSG00000160862 | e1 | alternative_first_exon | down | 2.08 | 1.97E-02 | up | 3.57 | 1.11E-02 | No |
| *ZRANB3* | BX647838 | ENSG00000121988 | e25 | Unknown | down | 2.08 | 1.81E-02 | up | 1.66 | 3.07E-02 | No |
| *CYP4X1* | AK131355 | ENSG00000186377 | e4 | Unknown | down | 2.06 | 1.11E-02 | up | 3.59 | 3.39E-02 | No |
| *LIMK1* | D26309 | ENSG00000106683 | e4 | exon_skipping,alternative_first_exon | down | 2.06 | 2.17E-02 | up | 1.67 | 3.53E-02 | No |
| *ZNRF3* | AB051436 | ENSG00000183579 | e2 | exon_skipping,alternative_first_exon | down | 2.05 | 4.86E-03 | up | 1.66 | 3.91E-02 | No |
| *CD68 // EIF4A1 // SENP3* | BC032613 | ENSG00000129226 // ENSG00000161956 // ENSG00000161960 | e29 | Unknown | down | 2.03 | 1.62E-02 | up | 1.83 | 3.76E-05 | No |
| *CD68 // EIF4A1 // SENP3* | BC032613 | ENSG00000129226 // ENSG00000161956 // ENSG00000161960 | e27 | Unknown | down | 2.02 | 1.01E-02 | up | 1.83 | 4.25E-05 | Yes |
| *CSNK1E* | AK092269 | ENSG00000213923 | e7 | Unknown | down | 2.01 | 1.68E-02 | up | 1.61 | 9.43E-04 | Yes |
| *GRWD1* | AB075822 | ENSG00000105447 | e11 | exon_skipping | down | 2.01 | 1.04E-02 | up | 1.64 | 1.74E-03 | Yes |
| *GRM8* | AK290197 | ENSG00000179603 | ae15 | alternative_donor_splice_site,alternative_last_exon | down | 2.01 | 9.38E-09 | up | 2.78 | 3.90E-02 | Yes |
| *KRT6C* | BC110639 | ENSG00000170465 | e3 | Unknown | down | 1.98 | 1.16E-02 | up | 1.91 | 3.79E-05 | Yes |
| *DDX52* | BC041785 | ENSG00000141141 | e4 | exon_skipping | down | 1.96 | 8.23E-04 | up | 1.75 | 6.14E-03 | Yes |
| *FASN* | U29344 | ENSG00000169710 | e21 | Unknown | down | 1.96 | 6.28E-10 | up | 1.76 | 1.17E-02 | No |
| *CASP1* | AK128199 | ENSG00000137752 | ae11 | intron_retention | down | 1.95 | 4.03E-02 | up | 3.04 | 6.96E-03 | Yes |
| *RAD54B* | AK290437 | ENSG00000197275 | e1 | alternative_first_exon | down | 1.95 | 1.78E-02 | up | 1.86 | 2.26E-02 | Yes |
| *BAX* | AK291076 | ENSG00000087088 | e1 | alternative_first_exon | down | 1.94 | 1.04E-02 | up | 2.02 | 3.34E-03 | Yes |
| *IDE* | BX648462 | ENSG00000119912 | e4 | exon_skipping | down | 1.94 | 1.74E-02 | up | 1.59 | 9.46E-03 | Yes |
| *RNF43* | AB081837 | ENSG00000108375 | e8 | Unknown | down | 1.93 | 3.17E-02 | up | 3.03 | 5.02E-03 | No |
| *BCAS2 // DENND2C* | BC063894 | ENSG00000116752 // ENSG00000175984 | e13 | Unknown | down | 1.91 | 5.98E-03 | up | 1.58 | 2.19E-02 | No |
| *CD68 // EIF4A1 // SENP3* | BC032613 | ENSG00000129226 // ENSG00000161956 // ENSG00000161960 | e25 | Unknown | down | 1.90 | 1.63E-02 | up | 1.83 | 4.25E-05 | Yes |
| *C13orf18* | AK125950 | ENSG00000102445 | e12 | exon_skipping | down | 1.90 | 1.32E-02 | up | 2.63 | 6.80E-03 | No |
| *GFM2* | AF367997 | ENSG00000164347 | e21 | Unknown | down | 1.90 | 7.56E-05 | up | 1.64 | 1.16E-02 | No |
| *DLGAP5* | AB076695 | ENSG00000126787 | e19 | exon_skipping | down | 1.90 | 2.54E-02 | up | 1.71 | 3.23E-02 | Yes |
| *PLS3* | BC056898 | ENSG00000102024 | e5 | exon_skipping | down | 1.90 | 2.04E-02 | up | 1.62 | 3.44E-02 | No |
| *IL20RA* | AF184971 | ENSG00000016402 | e1 | alternative_first_exon | down | 1.90 | 3.35E-02 | up | 2.00 | 3.97E-02 | Yes |
| *C12orf10* | CR626228 | ENSG00000139637 | ae3 | alternative_last_exon | down | 1.89 | 6.60E-03 | up | 1.56 | 5.74E-03 | Yes |
| *HSPH1* | BX648125 | ENSG00000120694 | e3 | Unknown | down | 1.88 | 7.08E-07 | up | 2.44 | 4.98E-04 | No |
| *SHMT2* | BC091501 | ENSG00000182199 | ae7 | intron_retention,alternative_acceptor_splice_site | down | 1.88 | 2.14E-03 | up | 1.65 | 2.98E-03 | Yes |
| *PHF19* | BX640713 | ENSG00000119403 | ae6 | alternative_last_exon | down | 1.88 | 2.65E-02 | up | 1.73 | 1.16E-02 | Yes |
| *RPH3AL* | BC005153 | ENSG00000181031 | e7 | Unknown | down | 1.87 | 5.84E-04 | up | 1.90 | 1.68E-03 | Yes |
| *TNFRSF12A* | BC002718 | ENSG00000006327 | e1 | alternative_first_exon | down | 1.87 | 5.24E-03 | up | 2.25 | 3.06E-03 | Yes |
| *RNF167* | CR617000 | ENSG00000108523 | ae3 | alternative_first_exon | down | 1.87 | 7.30E-03 | up | 1.58 | 1.52E-02 | Yes |
| *PLS3* | BC056898 | ENSG00000102024 | e1 | alternative_first_exon | down | 1.87 | 2.56E-02 | up | 1.62 | 3.44E-02 | No |
| *LYAR* | CR609495 | ENSG00000145220 | e9 | Unknown | down | 1.87 | 3.92E-03 | up | 1.81 | 3.83E-02 | No |
| *CD68 // EIF4A1 // SENP3* | BC032613 | ENSG00000129226 // ENSG00000161956 // ENSG00000161960 | e30 | alternative_last_exon | down | 1.86 | 5.64E-03 | up | 1.83 | 4.25E-05 | Yes |
| *USP14* | BC003556 | ENSG00000101557 | e1 | alternative_first_exon | down | 1.86 | 2.75E-02 | up | 1.67 | 7.88E-03 | Yes |
| *YWHAE* | AK128785 | ENSG00000108953 | e2 | exon_skipping | down | 1.85 | 4.37E-04 | up | 1.62 | 8.22E-04 | Yes |
| *CXCL16* | AK027389 | ENSG00000161921 | ae6 | intron_retention,alternative_last_exon | down | 1.85 | 1.52E-03 | up | 2.27 | 1.60E-03 | No |
| *KIAA1199* | AY585237 | ENSG00000103888 | e20 | exon_skipping | down | 1.85 | 7.62E-04 | up | 4.57 | 2.52E-03 | No |
| *NAA15* | AJ314788 | ENSG00000164134 | e22 | Unknown | down | 1.85 | 1.86E-02 | up | 1.74 | 3.05E-02 | Yes |
| *B3GALNT2* | BC029564 | ENSG00000162885 | e2 | exon_skipping | down | 1.84 | 1.80E-03 | up | 1.61 | 3.40E-03 | Yes |
| *CTSH* | CR596280 | ENSG00000103811 | e1 | alternative_first_exon | down | 1.83 | 1.71E-02 | up | 2.15 | 1.47E-02 | Yes |
| *SHCBP1* | BC030699 | ENSG00000171241 | e1 | alternative_first_exon | down | 1.83 | 3.33E-02 | up | 1.53 | 3.59E-02 | Yes |
| *CCT4* | AK303082 | ENSG00000115484 | e2 | exon_skipping | down | 1.82 | 4.20E-03 | up | 1.69 | 1.66E-02 | Yes |
| *PMPCB* | AK297354 | ENSG00000105819 | e14 | alternative_last_exon | down | 1.81 | 4.79E-04 | up | 1.54 | 1.82E-02 | Yes |
| *DPEP1* | CR620135 | ENSG00000015413 | e4 | Unknown | down | 1.80 | 4.98E-03 | up | 9.23 | 6.33E-08 | No |
| *MRRF* | AK300718 | ENSG00000148187 | e7 | exon_skipping | down | 1.80 | 3.28E-02 | up | 1.65 | 1.83E-02 | Yes |
| *PLCB4* | AK307837 | ENSG00000101333 | e15 | Unknown | down | 1.79 | 2.62E-02 | up | 3.18 | 2.56E-03 | No |
| *DDX52* | BC041785 | ENSG00000141141 | e2 | exon_skipping,alternative_first_exon | down | 1.79 | 1.60E-02 | up | 1.75 | 6.14E-03 | Yes |
| *INPP4B* | BX649090 | ENSG00000109452 | e23 | Unknown | down | 1.79 | 4.21E-04 | up | 1.74 | 1.26E-02 | Yes |
| *BCAS2 // DENND2C* | BC063894 | ENSG00000116752 // ENSG00000175984 | e11 | Unknown | down | 1.79 | 3.10E-02 | up | 1.58 | 2.19E-02 | Yes |
| *SPATA20* | AK125807 | ENSG00000006282 | ae15 | intron_retention,alternative_last_exon | down | 1.77 | 2.37E-02 | up | 2.16 | 6.64E-04 | Yes |
| *DDX20* | BC034953 | ENSG00000064703 | ae4 | alternative_first_exon | down | 1.77 | 7.80E-03 | up | 1.66 | 2.43E-02 | No |
| *HSPH1* | BX648125 | ENSG00000120694 | ae2 | alternative_first_exon | down | 1.76 | 9.22E-03 | up | 2.44 | 4.98E-04 | No |
| *HMGA1* | M23617 | ENSG00000137309 | e1 | alternative_first_exon | down | 1.75 | 2.57E-04 | up | 1.72 | 1.88E-08 | Yes |
| *PDCD11* | BC172442 | ENSG00000148843 | e29 | Unknown | down | 1.75 | 1.62E-02 | up | 1.54 | 2.71E-02 | No |
| *E2F5* | AB209185 | ENSG00000133740 | e2 | exon_skipping,alternative_first_exon | down | 1.75 | 3.98E-02 | up | 1.90 | 3.74E-02 | Yes |
| *CTPS* | BC009408 | ENSG00000171793 | ae9 | alternative_last_exon | down | 1.75 | 4.67E-02 | up | 1.65 | 3.75E-02 | Yes |
| *BMP7* | AK094784 | ENSG00000101144 | e4 | exon_skipping | down | 1.75 | 6.70E-03 | up | 2.52 | 3.81E-02 | Yes |
| *SYMPK* | AK130602 | ENSG00000125755 | e2 | exon_skipping,alternative_first_exon | down | 1.74 | 8.58E-03 | up | 1.52 | 5.82E-03 | No |
| *RARG* | BC072462 | ENSG00000172819 | e1 | alternative_first_exon | down | 1.74 | 1.84E-03 | up | 2.14 | 6.48E-03 | Yes |
| *SOCS7* | BC128607 | ENSG00000174111 | ae2 | alternative_acceptor_splice_site,alternative_first_exon | down | 1.74 | 2.63E-02 | up | 1.59 | 8.20E-03 | No |
| *BCAS2 // DENND2C* | BC063894 | ENSG00000116752 // ENSG00000175984 | e33 | Unknown | down | 1.74 | 2.28E-02 | up | 1.58 | 2.19E-02 | Yes |
| *SPATA18* | BC025396 | ENSG00000163071 | e6 | Unknown | down | 1.74 | 2.58E-02 | up | 2.21 | 2.93E-02 | No |
| *SMOX* | AK293485 | ENSG00000088826 | e9 | alternative_last_exon | down | 1.74 | 1.50E-03 | up | 1.85 | 3.57E-02 | Yes |
| *TSR1* | BC110851 | ENSG00000167721 | e14 | Unknown | down | 1.72 | 1.76E-04 | up | 1.79 | 7.35E-04 | Yes |
| *CCDC86* | AK025974 | ENSG00000110104 | e3 | Unknown | down | 1.72 | 1.88E-03 | up | 1.69 | 4.58E-03 | Yes |
| *MRRF* | AK300718 | ENSG00000148187 | ae5 | alternative_last_exon,intron_retention | down | 1.72 | 3.52E-02 | up | 1.65 | 1.83E-02 | Yes |
| *GPR56* | AK131550 | ENSG00000205336 | e5 | exon_skipping | down | 1.71 | 1.48E-03 | up | 2.18 | 8.17E-05 | Yes |
| *RPAP1* | AK022794 | ENSG00000103932 | e7 | exon_skipping | down | 1.71 | 5.92E-03 | up | 1.61 | 3.12E-03 | Yes |
| *VWA2* | AY572972 | ENSG00000165816 | e9 | Unknown | down | 1.71 | 1.96E-03 | up | 2.83 | 3.92E-03 | Yes |
| *MMP7* | EU176770 | ENSG00000137673 | e1 | alternative_first_exon | down | 1.71 | 5.98E-03 | up | 5.22 | 1.55E-02 | No |
| *CCT4* | AK303082 | ENSG00000115484 | e8 | Unknown | down | 1.71 | 8.58E-03 | up | 1.69 | 1.66E-02 | Yes |
| *VWA2* | AY572972 | ENSG00000165816 | e6 | Unknown | down | 1.70 | 8.76E-03 | up | 2.74 | 5.28E-03 | No |
| *IDE* | BX648462 | ENSG00000119912 | e22 | exon_skipping | down | 1.70 | 5.18E-03 | up | 1.59 | 9.46E-03 | Yes |
| *RAD54B* | AK290437 | ENSG00000197275 | e12 | Unknown | down | 1.70 | 2.68E-03 | up | 1.86 | 2.26E-02 | Yes |
| *TBCK* | AK074305 | ENSG00000145348 | e7 | exon_skipping | down | 1.70 | 3.31E-02 | up | 1.54 | 2.63E-02 | Yes |
| *MRRF* | AK300718 | ENSG00000148187 | e1 | alternative_first_exon | down | 1.69 | 1.59E-02 | up | 1.65 | 1.83E-02 | Yes |
| *POLR2C* | BC003159 | ENSG00000102978 | ae9 | intron_retention | down | 1.68 | 1.37E-02 | up | 1.61 | 5.76E-04 | Yes |
| *UNC119B* | AK126367 | ENSG00000175970 | e3 | Unknown | down | 1.68 | 6.00E-03 | up | 1.66 | 3.56E-03 | Yes |
| *PIGP* | AK304656 | ENSG00000185808 | e8 | Unknown | down | 1.68 | 3.42E-03 | up | 1.70 | 1.43E-02 | Yes |
| *PLCB4* | AK307837 | ENSG00000101333 | e20 | Unknown | down | 1.67 | 6.24E-03 | up | 3.18 | 2.56E-03 | No |
| *INTS9* | AK225530 | ENSG00000104299 | e2 | Unknown | down | 1.67 | 6.22E-03 | up | 1.56 | 1.22E-02 | Yes |
| *CLDN2* | AF177340 | ENSG00000165376 | ae4 | intron_retention | down | 1.66 | 4.96E-04 | up | 9.47 | 1.75E-12 | No |
| *C15orf41* | BC006254 | ENSG00000186073 | e4 | Unknown | down | 1.66 | 3.15E-02 | up | 1.60 | 5.92E-08 | No |
| *CLDN1* | AK225963 | ENSG00000163347 | e5 | alternative_last_exon | down | 1.66 | 1.87E-02 | up | 3.79 | 7.56E-03 | Yes |
| *PYCR1* | AK225879 | ENSG00000183010 | e6 | exon_skipping | down | 1.66 | 3.94E-02 | up | 1.81 | 3.08E-02 | Yes |
| *KLK11* | BC022068 | ENSG00000167757 | e6 | Unknown | down | 1.65 | 1.00E-03 | up | 6.84 | 9.18E-04 | No |
| *SYMPK* | AK130602 | ENSG00000125755 | e27 | Unknown | down | 1.65 | 2.16E-04 | up | 1.52 | 5.82E-03 | No |
| *CASP1* | AK128199 | ENSG00000137752 | e9 | Unknown | down | 1.65 | 3.78E-03 | up | 3.04 | 6.96E-03 | Yes |
| *ABCE1* | X76388 | ENSG00000164163 | e6 | Unknown | down | 1.65 | 2.63E-02 | up | 2.02 | 1.01E-02 | Yes |
| *NUDT14* | AK128196 | ENSG00000183828 | e1 | alternative_first_exon | down | 1.65 | 2.38E-03 | up | 1.69 | 1.21E-02 | No |
| *METTL11A* | AK298840 | ENSG00000148335 | e1 | alternative_first_exon | down | 1.64 | 3.92E-02 | up | 1.55 | 2.04E-02 | No |
| *TACC3* | AK225631 | ENSG00000013810 | e6 | exon_skipping | down | 1.64 | 1.10E-02 | up | 1.72 | 2.34E-02 | Yes |
| *KAT2A* | BC039907 | ENSG00000108773 | e16 | Unknown | down | 1.64 | 3.65E-07 | up | 1.57 | 2.37E-02 | No |
| *HSPA4L* | AK303053 | ENSG00000164070 | e21 | alternative_last_exon | down | 1.64 | 4.40E-03 | up | 2.36 | 2.46E-02 | No |
| *CKS2* | CR621720 | ENSG00000123975 | e3 | alternative_last_exon | down | 1.64 | 3.59E-02 | up | 2.47 | 2.73E-02 | No |
| *POLD2* | CR590132 | ENSG00000106628 | e11 | alternative_last_exon | down | 1.64 | 2.48E-02 | up | 1.65 | 3.16E-02 | Yes |
| *S100A11* | D38583 | ENSG00000163191 | e1 | alternative_first_exon | down | 1.64 | 1.90E-02 | up | 1.56 | 4.96E-02 | Yes |
| *G6PD* | X03674 | ENSG00000160211 | e3 | Unknown | down | 1.63 | 5.42E-03 | up | 1.66 | 7.14E-03 | Yes |
| *PEX10* | DQ895151 | ENSG00000157911 | e6 | Unknown | down | 1.63 | 7.80E-04 | up | 1.63 | 1.16E-02 | Yes |
| *C20orf96* | AK097394 | ENSG00000196476 | e5 | Unknown | down | 1.63 | 4.36E-04 | up | 1.55 | 2.20E-02 | No |
| *SH3PXD2B* | BC038561 | ENSG00000174705 | e7 | Unknown | down | 1.63 | 4.67E-02 | up | 1.57 | 2.22E-02 | No |
| *PTP4A3* | AK311257 | ENSG00000184489 | e5 | exon_skipping | down | 1.63 | 2.25E-02 | up | 2.88 | 3.25E-02 | No |
| *ADAP1 // COX19* | AK290517 | ENSG00000105963 // ENSG00000240230 | e6 | Unknown | down | 1.63 | 3.40E-03 | up | 1.57 | 3.82E-02 | Yes |
| *AREG* | BC009799 | ENSG00000109321 | e1 | alternative_first_exon | down | 1.62 | 4.83E-02 | up | 3.06 | 7.02E-10 | Yes |
| *RCC1 // SNHG3* | BC007300 | ENSG00000180198 // ENSG00000242125 | e4 | Unknown | down | 1.62 | 3.40E-02 | up | 1.75 | 4.38E-03 | Yes |
| *RARG* | BC072462 | ENSG00000172819 | e10 | Unknown | down | 1.62 | 1.62E-12 | up | 2.14 | 6.48E-03 | No |
| *SLC29A3* | AX775921 | ENSG00000198246 | e1 | alternative_first_exon | down | 1.62 | 4.90E-02 | up | 1.73 | 2.04E-02 | Yes |
| *FOXRED1* | AK298807 | ENSG00000110074 | e1 | alternative_first_exon | down | 1.62 | 2.40E-04 | up | 1.54 | 2.52E-02 | Yes |
| *PDCD11* | BC172442 | ENSG00000148843 | e1 | alternative_first_exon | down | 1.62 | 1.23E-02 | up | 1.54 | 2.71E-02 | Yes |
| *---* | AK098044 | --- | e9 | alternative_last_exon | down | 1.61 | 1.74E-03 | up | 1.50 | 5.36E-03 | Yes |
| *C7orf40* | BC092459 | ENSG00000232956 | ae3 | intron_retention | down | 1.61 | 4.07E-02 | up | 2.12 | 3.07E-02 | Yes |
| *CCNC* | BC010135 | ENSG00000112237 | ae5 | alternative_last_exon | down | 1.61 | 3.64E-02 | up | 1.50 | 4.29E-02 | No |
| *CCNA2* | CR604810 | ENSG00000145386 | e1 | alternative_first_exon | down | 1.60 | 4.62E-03 | up | 2.15 | 1.12E-02 | No |
| *HSPA4L* | AK303053 | ENSG00000164070 | e8 | Unknown | down | 1.60 | 2.60E-04 | up | 2.36 | 2.46E-02 | No |
| *MCM3* | D38073 | ENSG00000112118 | e1 | alternative_first_exon | down | 1.60 | 7.18E-03 | up | 1.55 | 3.81E-02 | Yes |
| *CDC16* | BC017244 | ENSG00000130177 | ae6 | intron_retention | down | 1.59 | 1.55E-02 | up | 1.63 | 6.56E-03 | No |
| *PITX2* | AK127829 | ENSG00000164093 | e4 | Unknown | down | 1.59 | 6.14E-03 | up | 2.95 | 1.12E-02 | No |
| *EPHA1* | M18391 | ENSG00000146904 | e2 | Unknown | down | 1.59 | 4.62E-02 | up | 1.83 | 1.18E-02 | Yes |
| *PLCB4* | AK307837 | ENSG00000101333 | e37 | Unknown | down | 1.58 | 1.44E-03 | up | 3.18 | 2.56E-03 | No |
| *PEX5* | AK292256 | ENSG00000139197 | e2 | exon_skipping,alternative_first_exon | down | 1.58 | 2.18E-02 | up | 1.57 | 7.76E-03 | Yes |
| *FASN* | U29344 | ENSG00000169710 | e24 | Unknown | down | 1.58 | 1.07E-02 | up | 1.74 | 1.15E-02 | Yes |
| *C20orf96* | AK097394 | ENSG00000196476 | e6 | Unknown | down | 1.58 | 4.66E-02 | up | 1.55 | 2.20E-02 | Yes |
| *TMEM177* | AK057313 | ENSG00000144120 | e4 | Unknown | down | 1.58 | 3.36E-02 | up | 1.53 | 4.48E-02 | No |
| *PPIL1* | AY359032 | ENSG00000137168 | e1 | alternative_first_exon | down | 1.57 | 2.01E-02 | up | 1.96 | 1.26E-03 | Yes |
| *RPAP1* | AK022794 | ENSG00000103932 | e2 | exon_skipping | down | 1.56 | 4.58E-03 | up | 1.61 | 3.12E-03 | Yes |
| *PVT1* | BC041065 | ENSG00000249859 | e11 | Unknown | down | 1.56 | 8.82E-03 | up | 2.41 | 7.48E-03 | Yes |
| *EIF2S1* | BC002513 | ENSG00000134001 | e9 | alternative_last_exon | down | 1.56 | 1.64E-03 | up | 1.69 | 8.38E-03 | Yes |
| *FIGF // PIR* | AX740234 | ENSG00000087842 // ENSG00000165197 | e9 | Unknown | down | 1.56 | 1.28E-02 | up | 1.72 | 2.49E-02 | No |
| *ZRANB3* | BX647838 | ENSG00000121988 | e1 | alternative_first_exon | down | 1.56 | 3.38E-02 | up | 1.66 | 3.07E-02 | Yes |
| *NCAPG* | AF331796 | ENSG00000109805 | e21 | alternative_last_exon | down | 1.56 | 2.02E-03 | up | 1.77 | 4.49E-02 | Yes |
| *RPAP1* | AK022794 | ENSG00000103932 | e5 | exon_skipping | down | 1.55 | 3.41E-04 | up | 1.61 | 3.12E-03 | Yes |
| *C12orf66* | BC036246 | ENSG00000174206 | e4 | alternative_last_exon | down | 1.55 | 1.06E-03 | up | 1.60 | 1.39E-02 | Yes |
| *CSE1L* | AF053641 | ENSG00000124207 | ae25 | alternative_acceptor_splice_site,internal_exon_deletion | down | 1.55 | 2.92E-02 | up | 1.72 | 3.74E-02 | Yes |
| *TTC8* | BX247959 | ENSG00000165533 | e2 | Unknown | down | 1.54 | 2.30E-02 | up | 1.75 | 4.76E-03 | No |
| *BANF1* | AF068235 | ENSG00000175334 | e1 | alternative_first_exon | down | 1.54 | 2.42E-02 | up | 1.51 | 1.91E-02 | Yes |
| *PSMD6* | AY359879 | ENSG00000163636 | e3 | exon_skipping | down | 1.54 | 1.34E-07 | up | 1.65 | 2.07E-02 | Yes |
| *ITPA* | AF219116 | ENSG00000125877 | e3 | Unknown | down | 1.54 | 9.50E-03 | up | 1.70 | 4.60E-02 | Yes |
| *SLC22A3* | BC172500 | ENSG00000146477 | e9 | Unknown | down | 1.53 | 5.68E-09 | up | 2.44 | 5.30E-03 | No |
| *WDR74* | AK292330 | ENSG00000133316 | e1 | alternative_first_exon | down | 1.53 | 2.65E-07 | up | 1.63 | 1.68E-02 | Yes |
| *WDR74* | AK292330 | ENSG00000133316 | e3 | Unknown | down | 1.53 | 1.32E-02 | up | 1.63 | 1.68E-02 | Yes |
| *PSMC2* | AB075520 | ENSG00000161057 | e1 | alternative_first_exon | down | 1.53 | 1.36E-03 | up | 1.72 | 2.20E-02 | Yes |
| *C19orf48* | CR591122 | ENSG00000167747 | e4 | Unknown | down | 1.52 | 5.12E-03 | up | 1.61 | 5.84E-07 | Yes |
| *AEN* | BC020988 | ENSG00000181026 | e4 | alternative_last_exon | down | 1.52 | 3.00E-03 | up | 2.42 | 2.99E-04 | Yes |
| *SNORD1C* | AB073602 | ENSG00000200185 | ae4 | intron_retention,alternative_acceptor_splice_site | down | 1.52 | 2.90E-03 | up | 2.03 | 7.22E-04 | No |
| *STK36* | AB033104 | ENSG00000163482 | e16 | Unknown | down | 1.52 | 7.54E-03 | up | 1.57 | 7.84E-03 | Yes |
| *INPP4B* | BX649090 | ENSG00000109452 | e15 | Unknown | down | 1.52 | 4.34E-03 | up | 1.74 | 1.26E-02 | No |
| *LIMK1* | D26309 | ENSG00000106683 | e14 | Unknown | down | 1.52 | 8.00E-04 | up | 1.67 | 3.53E-02 | No |
| *ADAP1 // COX19* | AK290517 | ENSG00000105963 // ENSG00000240230 | e8 | Unknown | down | 1.52 | 9.02E-03 | up | 1.57 | 3.82E-02 | Yes |
| *SLCO4A1* | BC015727 | ENSG00000101187 | e5 | exon_skipping | down | 1.52 | 2.48E-03 | up | 1.88 | 4.83E-02 | No |
| *TEX10* | AB060968 | ENSG00000136891 | e13 | Unknown | down | 1.52 | 2.36E-02 | up | 1.68 | 4.93E-02 | No |
| *CDC16* | BC017244 | ENSG00000130177 | e1 | alternative_first_exon | down | 1.51 | 4.75E-02 | up | 1.63 | 6.56E-03 | Yes |
| *STK36* | AB033104 | ENSG00000163482 | e11 | Unknown | down | 1.51 | 2.97E-02 | up | 1.57 | 7.84E-03 | Yes |
| *GFM2* | AF367997 | ENSG00000164347 | e20 | Unknown | down | 1.51 | 5.06E-03 | up | 1.64 | 1.16E-02 | No |
| *EXOSC7* | AK130053 | ENSG00000075914 | e2 | exon_skipping,alternative_first_exon | down | 1.51 | 2.84E-02 | up | 1.64 | 1.34E-02 | Yes |
| *CCT4* | AK303082 | ENSG00000115484 | e1 | alternative_first_exon | down | 1.51 | 1.90E-02 | up | 1.69 | 1.66E-02 | No |
| *GGCT* | BC000625 | ENSG00000006625 | e4 | exon_skipping | down | 1.51 | 3.05E-02 | up | 1.90 | 3.08E-02 | No |
| *PLS3* | BC056898 | ENSG00000102024 | e9 | exon_skipping | down | 1.51 | 4.90E-03 | up | 1.62 | 3.44E-02 | No |
| *PGAP2* | AL096753 | --- | e8 | exon_skipping | down | 1.50 | 3.79E-02 | up | 1.53 | 1.25E-08 | Yes |
| *HSPH1* | BX648125 | ENSG00000120694 | e2 | alternative_first_exon,exon_skipping | down | 1.50 | 4.22E-03 | up | 2.44 | 4.98E-04 | Yes |
| *TMEM161A* | AK096964 | ENSG00000064545 | e9 | Unknown | down | 1.50 | 3.02E-04 | up | 1.70 | 2.34E-03 | Yes |
| *CASP1* | AK128199 | ENSG00000137752 | e10 | exon_skipping | down | 1.50 | 2.67E-02 | up | 3.04 | 6.96E-03 | Yes |
| *EXOSC7* | AK130053 | ENSG00000075914 | e9 | alternative_last_exon | down | 1.50 | 2.09E-02 | up | 1.64 | 1.34E-02 | Yes |
| *SLCO4A1* | BC015727 | ENSG00000101187 | e6 | Unknown | down | 1.50 | 2.41E-04 | up | 1.88 | 4.83E-02 | No |
| *SLCO4A1* | BC015727 | ENSG00000101187 | e3 | exon_skipping | down | 1.50 | 1.38E-03 | up | 1.88 | 4.83E-02 | No |
| *NUDCD1* | BC043406 | ENSG00000120526 | e9 | Unknown | down | 1.50 | 8.37E-04 | up | 1.54 | 4.91E-02 | Yes |
| *GUCA2B* | U34279 | ENSG00000044012 | e1 | alternative_first_exon | up | 15.08 | 3.33E-06 | down | 16.99 | 1.62E-12 | Yes |
| *CLCA4* | AK000072 | ENSG00000016602 | ae9 | alternative_last_exon | up | 11.74 | 1.66E-03 | down | 23.99 | 3.16E-04 | Yes |
| *---* | AK297065 | --- | e1 | alternative_first_exon | up | 8.28 | 1.35E-02 | down | 24.88 | 2.58E-03 | No |
| *CHRDL1* | BC002909 | ENSG00000101938 | ae1 | alternative_donor_splice_site | up | 6.97 | 1.99E-08 | down | 7.10 | 1.14E-10 | Yes |
| *SLC4A4* | AF011390 | ENSG00000080493 | ae14 | alternative_last_exon | up | 5.34 | 8.98E-11 | down | 9.06 | 1.02E-03 | No |
| *SLC4A4* | AF011390 | ENSG00000080493 | e1 | alternative_first_exon | up | 5.29 | 1.74E-03 | down | 9.06 | 1.02E-03 | No |
| *NAALADL1* | BC143644 | ENSG00000168060 | ae7 | intron_retention | up | 5.25 | 1.63E-12 | down | 4.22 | 1.93E-09 | No |
| *CD177* | CR592446 | ENSG00000204936 | e9 | Unknown | up | 4.92 | 1.45E-02 | down | 22.68 | 3.02E-04 | No |
| *PLP1* | AK128782 | ENSG00000123560 | e6 | alternative_last_exon | up | 4.40 | 8.71E-09 | down | 10.85 | 2.88E-14 | No |
| *GUCA2B* | U34279 | ENSG00000044012 | e2 | Unknown | up | 4.36 | 4.38E-04 | down | 16.99 | 1.62E-12 | Yes |
| *SCNN1B* | BC036352 | ENSG00000168447 | e15 | alternative_last_exon | up | 4.32 | 1.32E-03 | down | 9.36 | 1.55E-04 | Yes |
| *OTOP2* | BC152986 | ENSG00000183034 | e8 | alternative_last_exon | up | 4.29 | 2.27E-08 | down | 19.34 | 4.02E-13 | No |
| *CCL21* | DQ895242 | ENSG00000137077 | ae2 | alternative_first_exon | up | 4.23 | 6.12E-03 | down | 15.49 | 3.60E-04 | No |
| *FBLN1* | AK075566 | ENSG00000077942 | e3 | exon_skipping | up | 4.19 | 3.80E-03 | down | 4.49 | 1.62E-03 | Yes |
| *TRPM6* | AF350881 | ENSG00000119121 | ae20 | alternative_first_exon | up | 4.14 | 2.18E-05 | down | 7.04 | 5.75E-07 | No |
| *MFAP5* | BC005901 | ENSG00000197614 | e1 | alternative_first_exon | up | 4.05 | 1.87E-05 | down | 11.16 | 1.45E-06 | No |
| *VIP* | M36634 | ENSG00000146469 | e1 | alternative_first_exon | up | 4.00 | 6.94E-09 | down | 10.15 | 5.80E-12 | No |
| *GCG* | BC005278 | ENSG00000115263 | e6 | alternative_last_exon | up | 3.93 | 1.80E-03 | down | 27.59 | 4.00E-06 | No |
| *TPH1* | X52836 | ENSG00000129167 | e1 | alternative_first_exon | up | 3.90 | 2.40E-04 | down | 7.43 | 1.85E-05 | No |
| *SCNN1B* | BC036352 | ENSG00000168447 | e2 | exon_skipping,alternative_first_exon | up | 3.77 | 1.62E-02 | down | 9.33 | 1.58E-04 | No |
| *FN1* | CR749317 | ENSG00000115414 | ae33 | intron_retention | up | 3.64 | 9.26E-11 | down | 5.23 | 1.76E-02 | No |
| *PCK1* | BC023978 | ENSG00000124253 | ae7 | intron_retention | up | 3.61 | 5.44E-03 | down | 6.46 | 4.26E-03 | No |
| *UGT2B15* | AF180322 | ENSG00000196620 | e1 | alternative_first_exon | up | 3.61 | 7.58E-03 | down | 5.73 | 3.44E-02 | Yes |
| *ADH1A // ADH1B* | BT019812 | ENSG00000187758 // ENSG00000196616 | e15 | Unknown | up | 3.58 | 2.00E-02 | down | 11.08 | 8.65E-07 | No |
| *CP* | AK299272 | ENSG00000047457 | e21 | alternative_last_exon | up | 3.55 | 4.88E-03 | down | 4.94 | 2.34E-06 | No |
| *CXCL12* | DQ345520 | ENSG00000107562 | e1 | alternative_first_exon | up | 3.42 | 1.84E-03 | down | 6.02 | 1.65E-12 | No |
| *NAALADL1* | BC143644 | ENSG00000168060 | e8 | Unknown | up | 3.34 | 9.18E-08 | down | 4.22 | 1.93E-09 | No |
| *TMIGD1* | AK172838 | ENSG00000182271 | e7 | alternative_last_exon | up | 3.33 | 1.76E-05 | down | 17.34 | 1.69E-11 | No |
| *SFRP2* | AF311912 | ENSG00000145423 | e4 | alternative_last_exon | up | 3.33 | 3.43E-04 | down | 10.91 | 1.67E-08 | No |
| *SLC17A4* | AK024903 | ENSG00000146039 | ae10 | intron_retention | up | 3.27 | 1.22E-02 | down | 4.29 | 9.50E-03 | No |
| *ADH1A // ADH1B* | BT019812 | ENSG00000187758 // ENSG00000196616 | e2 | exon_skipping | up | 3.26 | 2.42E-04 | down | 11.08 | 8.65E-07 | No |
| *CXCL12* | DQ345520 | ENSG00000107562 | e6 | exon_skipping,alternative_last_exon | up | 3.19 | 1.38E-04 | down | 6.02 | 1.65E-12 | No |
| *CA1* | M33987 | ENSG00000133742 | e9 | alternative_last_exon | up | 3.19 | 4.47E-02 | down | 41.42 | 2.60E-03 | No |
| *ITIH5* | AY358426 | ENSG00000123243 | ae13 | alternative_last_exon | up | 3.19 | 3.87E-02 | down | 3.66 | 9.80E-03 | No |
| *ADH1A // ADH1B* | BT019812 | ENSG00000187758 // ENSG00000196616 | e17 | Unknown | up | 3.16 | 1.71E-02 | down | 11.08 | 8.65E-07 | No |
| *COL14A1* | BC140893 | ENSG00000187955 | e55 | alternative_last_exon | up | 3.15 | 3.60E-03 | down | 3.93 | 4.88E-03 | No |
| *CLCA4* | AK000072 | ENSG00000016602 | e14 | alternative_last_exon | up | 3.12 | 9.04E-03 | down | 23.99 | 3.16E-04 | No |
| *FBLN1* | AK075566 | ENSG00000077942 | e16 | exon_skipping,alternative_last_exon | up | 3.12 | 2.47E-02 | down | 4.44 | 1.88E-03 | No |
| *CP* | AK299272 | ENSG00000047457 | e19 | exon_skipping,alternative_last_exon | up | 3.07 | 8.24E-05 | down | 4.94 | 2.34E-06 | No |
| *ITIH5* | AY358426 | ENSG00000123243 | e14 | exon_skipping,alternative_last_exon | up | 3.07 | 2.84E-02 | down | 3.66 | 9.80E-03 | No |
| *TRPM6* | AF350881 | ENSG00000119121 | ae26 | intron_retention | up | 2.99 | 1.18E-03 | down | 7.04 | 5.75E-07 | No |
| *CCL21* | DQ895242 | ENSG00000137077 | e5 | alternative_last_exon | up | 2.97 | 8.18E-03 | down | 15.49 | 3.60E-04 | No |
| *ITIH5* | AY358426 | ENSG00000123243 | e18 | alternative_last_exon | up | 2.95 | 3.36E-02 | down | 3.66 | 9.80E-03 | Yes |
| *SULT1A1* | CR601418 | ENSG00000196502 | e3 | exon_skipping | up | 2.91 | 6.82E-03 | down | 2.40 | 1.52E-02 | Yes |
| *CTSG* | DQ896320 | ENSG00000100448 | ae2 | alternative_first_exon | up | 2.91 | 3.39E-02 | down | 2.77 | 2.21E-02 | Yes |
| *PTPRR* | D64053 | ENSG00000153233 | ae15 | alternative_acceptor_splice_site | up | 2.90 | 4.98E-03 | down | 4.55 | 1.68E-12 | No |
| *CP* | AK299272 | ENSG00000047457 | e20 | Unknown | up | 2.89 | 5.57E-04 | down | 4.94 | 2.34E-06 | No |
| *NAALADL1* | BC143644 | ENSG00000168060 | e4 | Unknown | up | 2.87 | 6.12E-05 | down | 4.22 | 1.93E-09 | Yes |
| *CD36* | AK096858 | ENSG00000135218 | e2 | Unknown | up | 2.87 | 5.70E-03 | down | 2.85 | 4.60E-03 | Yes |
| *TRPM6* | AF350881 | ENSG00000119121 | e41 | exon_skipping | up | 2.86 | 6.17E-04 | down | 7.04 | 5.75E-07 | No |
| *OGN* | AF086912 | ENSG00000106809 | e3 | Unknown | up | 2.83 | 1.85E-06 | down | 8.52 | 2.01E-13 | No |
| *NAALADL1* | BC143644 | ENSG00000168060 | e1 | alternative_first_exon | up | 2.81 | 1.74E-07 | down | 4.22 | 1.93E-09 | No |
| *TRPM6* | AF350881 | ENSG00000119121 | ae28 | alternative_first_exon | up | 2.79 | 3.76E-03 | down | 7.04 | 5.75E-07 | No |
| *ADH1A // ADH1B* | BT019812 | ENSG00000187758 // ENSG00000196616 | ae2 | intron_retention | up | 2.79 | 2.66E-03 | down | 11.08 | 8.65E-07 | No |
| *SRPX* | AK125542 | ENSG00000101955 | e1 | alternative_first_exon | up | 2.78 | 3.26E-03 | down | 3.22 | 1.84E-03 | Yes |
| *AEBP1* | BC038588 | ENSG00000106624 | ae13 | intron_retention | up | 2.77 | 3.28E-02 | down | 2.35 | 3.39E-02 | Yes |
| *TNS1* | AK001785 | ENSG00000079308 | e39 | alternative_last_exon | up | 2.73 | 1.36E-03 | down | 3.18 | 9.57E-04 | Yes |
| *TNS1* | AK001785 | ENSG00000079308 | e27 | exon_skipping | up | 2.73 | 1.79E-02 | down | 3.18 | 9.57E-04 | No |
| *USP2* | BC041366 | ENSG00000036672 | e6 | Unknown | up | 2.73 | 4.16E-03 | down | 3.67 | 1.04E-03 | No |
| *ALPI* | BC132678 | ENSG00000163295 | e4 | Unknown | up | 2.71 | 4.77E-08 | down | 3.79 | 4.71E-09 | No |
| *PLEKHG5 // TNFRSF25* | BC036671 | ENSG00000171680 // ENSG00000215788 | e29 | alternative_last_exon | up | 2.71 | 4.10E-03 | down | 1.77 | 2.37E-02 | No |
| *PLP1* | AK128782 | ENSG00000123560 | e4 | Unknown | up | 2.70 | 5.40E-07 | down | 10.85 | 2.88E-14 | No |
| *BEST4* | AF440757 | ENSG00000142959 | e1 | alternative_first_exon | up | 2.70 | 5.02E-04 | down | 11.98 | 6.35E-11 | No |
| *CASQ2* | AK129891 | ENSG00000118729 | e7 | exon_skipping | up | 2.69 | 6.43E-07 | down | 2.93 | 4.50E-11 | Yes |
| *CES2* | BX538086 | ENSG00000172831 | e1 | alternative_first_exon | up | 2.69 | 3.06E-03 | down | 2.68 | 2.29E-02 | Yes |
| *FN1* | CR749317 | ENSG00000115414 | e1 | alternative_first_exon | up | 2.68 | 2.42E-10 | down | 5.23 | 1.76E-02 | No |
| *ABCG2* | BC092408 | ENSG00000118777 | e6 | Unknown | up | 2.65 | 1.85E-05 | down | 4.44 | 2.38E-08 | No |
| *PLP1* | AK128782 | ENSG00000123560 | e9 | Unknown | up | 2.64 | 2.33E-06 | down | 10.85 | 2.88E-14 | No |
| *MAMDC2* | BC045569 | ENSG00000165072 | e1 | alternative_first_exon | up | 2.64 | 7.63E-05 | down | 4.07 | 1.07E-07 | No |
| *CA7* | AY075019 | ENSG00000168748 | e4 | Unknown | up | 2.63 | 1.82E-04 | down | 15.61 | 1.79E-08 | No |
| *EFEMP1* | AK293058 | ENSG00000115380 | e2 | exon_skipping | up | 2.63 | 2.16E-03 | down | 6.09 | 8.16E-05 | No |
| *SCNN1B* | BC036352 | ENSG00000168447 | e14 | Unknown | up | 2.59 | 2.36E-02 | down | 9.33 | 1.58E-04 | No |
| *FHL1* | BC010998 | ENSG00000022267 | e4 | exon_skipping,alternative_first_exon | up | 2.59 | 2.21E-02 | down | 2.68 | 1.48E-02 | Yes |
| *MYH11* | EU489063 | ENSG00000133392 | e32 | exon_skipping | up | 2.57 | 7.32E-03 | down | 5.37 | 1.65E-12 | No |
| *DPP10* | BC030832 | ENSG00000175497 | e4 | Unknown | up | 2.56 | 1.10E-02 | down | 3.23 | 8.74E-03 | No |
| *FN1* | CR749317 | ENSG00000115414 | ae40 | intron_retention | up | 2.53 | 3.12E-02 | down | 5.23 | 1.76E-02 | No |
| *CHGA* | BC006459 | ENSG00000100604 | e4 | Unknown | up | 2.50 | 1.14E-02 | down | 7.65 | 1.63E-12 | No |
| *---* | AK129601 | --- | e4 | alternative_last_exon | up | 2.50 | 3.20E-03 | down | 5.49 | 1.16E-03 | No |
| *CRYAB* | BC107897 | ENSG00000109846 | ae1 | alternative_first_exon,intron_retention | up | 2.50 | 5.28E-03 | down | 4.07 | 1.40E-03 | No |
| *TRPM6* | AF350881 | ENSG00000119121 | e11 | exon_skipping,alternative_first_exon | up | 2.48 | 1.08E-02 | down | 7.04 | 5.75E-07 | Yes |
| *ABCA8* | AB020629 | ENSG00000141338 | e37 | exon_skipping | up | 2.47 | 3.10E-06 | down | 4.33 | 9.69E-09 | No |
| *ZG16* | AK125559 | ENSG00000174992 | e1 | alternative_first_exon | up | 2.47 | 4.36E-02 | down | 15.63 | 2.14E-03 | No |
| *SLC4A4* | AF011390 | ENSG00000080493 | e4 | alternative_first_exon | up | 2.46 | 9.92E-03 | down | 9.06 | 1.02E-03 | No |
| *MYH11* | EU489063 | ENSG00000133392 | e20 | Unknown | up | 2.44 | 6.70E-03 | down | 5.37 | 1.65E-12 | No |
| *IGFBP3* | AK091745 | ENSG00000146674 | e2 | exon_skipping | up | 2.43 | 1.28E-08 | down | 3.75 | 3.02E-11 | No |
| *OTOP2* | BC152986 | ENSG00000183034 | e5 | Unknown | up | 2.41 | 4.13E-06 | down | 19.34 | 4.02E-13 | No |
| *PYY* | BC041057 | ENSG00000131096 | e6 | Unknown | up | 2.41 | 1.68E-02 | down | 8.60 | 1.02E-06 | No |
| *IL1R2* | BC039031 | ENSG00000115590 | e2 | exon_skipping,alternative_first_exon | up | 2.41 | 6.31E-12 | down | 3.44 | 2.42E-03 | No |
| *OGN* | AF086912 | ENSG00000106809 | e2 | alternative_first_exon | up | 2.38 | 1.47E-07 | down | 8.52 | 2.01E-13 | No |
| *TRPM6* | AF350881 | ENSG00000119121 | e4 | Unknown | up | 2.38 | 1.37E-07 | down | 7.04 | 5.75E-07 | No |
| *MYH11* | EU489063 | ENSG00000133392 | e15 | Unknown | up | 2.35 | 2.43E-02 | down | 5.37 | 1.65E-12 | No |
| *CP* | AK299272 | ENSG00000047457 | e12 | Unknown | up | 2.34 | 2.37E-05 | down | 4.94 | 2.34E-06 | No |
| *CFHR1* | BC107771 | ENSG00000244414 | e3 | Unknown | up | 2.34 | 4.42E-02 | down | 2.67 | 2.25E-02 | Yes |
| *GLI3* | AK308429 | ENSG00000106571 | e16 | Unknown | up | 2.33 | 4.65E-12 | down | 2.96 | 6.39E-04 | Yes |
| *MYH11* | EU489063 | ENSG00000133392 | e19 | Unknown | up | 2.30 | 6.24E-03 | down | 5.37 | 1.65E-12 | No |
| *GSTM2* | BC017836 | ENSG00000213366 | e4 | Unknown | up | 2.29 | 2.43E-02 | down | 2.21 | 7.09E-09 | No |
| *ABCA8* | AB020629 | ENSG00000141338 | e26 | exon_skipping,alternative_last_exon | up | 2.28 | 3.95E-05 | down | 4.33 | 9.69E-09 | No |
| *FBLN2* | AK304777 | ENSG00000163520 | e9 | Unknown | up | 2.28 | 8.19E-05 | down | 4.22 | 3.23E-07 | No |
| *CD177* | CR592446 | ENSG00000204936 | e1 | alternative_first_exon | up | 2.28 | 7.80E-05 | down | 22.68 | 3.02E-04 | No |
| *ADAMTSL3* | BC128390 | ENSG00000156218 | e18 | Unknown | up | 2.28 | 2.22E-03 | down | 2.40 | 3.79E-04 | Yes |
| *TMOD1* | AK314533 | ENSG00000136842 | e2 | exon_skipping,alternative_first_exon | up | 2.28 | 6.54E-03 | down | 2.60 | 1.58E-03 | Yes |
| *COL14A1* | BC140893 | ENSG00000187955 | e31 | Unknown | up | 2.28 | 7.36E-03 | down | 3.93 | 4.88E-03 | No |
| *SLC6A19* | AK096054 | ENSG00000174358 | e2 | Unknown | up | 2.27 | 2.46E-07 | down | 7.92 | 1.09E-13 | No |
| *ANK2* | BX537758 | ENSG00000145362 | e54 | exon_skipping | up | 2.27 | 3.69E-05 | down | 3.62 | 4.10E-08 | No |
| *MS4A12* | AK000224 | ENSG00000071203 | e8 | alternative_last_exon | up | 2.26 | 3.11E-02 | down | 24.59 | 7.63E-04 | No |
| *GUCY1A3* | BX649180 | ENSG00000164116 | ae1 | intron_retention | up | 2.26 | 3.36E-02 | down | 2.89 | 7.48E-03 | No |
| *FGFR1* | FJ809917 | ENSG00000077782 | e2 | exon_skipping,alternative_first_exon | up | 2.26 | 4.01E-02 | down | 2.28 | 3.21E-02 | Yes |
| *MYH11* | EU489063 | ENSG00000133392 | e33 | exon_skipping | up | 2.25 | 5.76E-03 | down | 5.37 | 1.65E-12 | No |
| *SCIN* | AB067492 | ENSG00000006747 | e4 | exon_skipping,alternative_first_exon | up | 2.25 | 2.96E-02 | down | 2.10 | 2.98E-02 | Yes |
| *ABCA8* | AB020629 | ENSG00000141338 | e38 | exon_skipping | up | 2.23 | 3.05E-12 | down | 4.33 | 9.69E-09 | No |
| *GPM6A* | AK226176 | ENSG00000150625 | e1 | alternative_first_exon | up | 2.22 | 8.16E-03 | down | 2.33 | 3.59E-05 | Yes |
| *ANK2* | BX537758 | ENSG00000145362 | e17 | Unknown | up | 2.21 | 4.31E-05 | down | 3.67 | 3.70E-08 | Yes |
| *MUSK* | BC109099 | ENSG00000030304 | e6 | exon_skipping | up | 2.21 | 1.66E-03 | down | 3.43 | 4.22E-07 | No |
| *ADH1A // ADH1B* | BT019812 | ENSG00000187758 // ENSG00000196616 | e10 | exon_skipping,alternative_last_exon | up | 2.21 | 1.55E-04 | down | 11.08 | 8.65E-07 | No |
| *SLC4A4* | AF011390 | ENSG00000080493 | e11 | Unknown | up | 2.21 | 4.33E-02 | down | 9.06 | 1.02E-03 | No |
| *SEMG1* | BC055416 | ENSG00000124233 | e1 | alternative_first_exon | up | 2.20 | 9.60E-03 | down | 2.73 | 7.84E-05 | No |
| *CHRDL1* | BC002909 | ENSG00000101938 | e8 | Unknown | up | 2.19 | 1.50E-03 | down | 7.10 | 1.14E-10 | Yes |
| *MXI1* | BC035128 | ENSG00000119950 | e4 | exon_skipping | up | 2.19 | 2.97E-04 | down | 1.91 | 1.98E-03 | No |
| *MYH11* | EU489063 | ENSG00000133392 | e22 | Unknown | up | 2.18 | 2.74E-02 | down | 5.37 | 1.65E-12 | No |
| *EDIL3* | BC030828 | ENSG00000164176 | e7 | exon_skipping,alternative_first_exon | up | 2.18 | 3.01E-02 | down | 4.17 | 3.60E-04 | No |
| *IGF2BP3* | AF117108 | ENSG00000136231 | e3 | Unknown | up | 2.16 | 1.97E-05 | down | 3.80 | 1.57E-10 | Yes |
| *SLC4A4* | AF011390 | ENSG00000080493 | e2 | alternative_first_exon | up | 2.16 | 4.30E-03 | down | 9.06 | 1.02E-03 | No |
| *TTLL7* | BC028350 | ENSG00000137941 | e23 | exon_skipping | up | 2.16 | 4.92E-03 | down | 2.47 | 1.22E-03 | No |
| *MYH11* | EU489063 | ENSG00000133392 | e21 | Unknown | up | 2.15 | 1.34E-02 | down | 5.37 | 1.65E-12 | No |
| *MYH11* | EU489063 | ENSG00000133392 | e28 | exon_skipping | up | 2.15 | 1.09E-02 | down | 5.37 | 1.65E-12 | No |
| *CA4* | AK298710 | ENSG00000167434 | e7 | Unknown | up | 2.15 | 3.88E-03 | down | 12.30 | 4.49E-05 | No |
| *THBS2* | L12350 | ENSG00000186340 | e12 | Unknown | up | 2.13 | 1.80E-02 | down | 3.18 | 8.47E-08 | No |
| *---* | BC038540 | --- | e1 | alternative_first_exon | up | 2.13 | 2.82E-02 | down | 26.89 | 1.19E-04 | Yes |
| *CALB2* | AK222648 | ENSG00000172137 | e1 | alternative_first_exon | up | 2.12 | 2.78E-04 | down | 2.57 | 1.88E-06 | Yes |
| *PLEKHG5 // TNFRSF25* | BC036671 | ENSG00000171680 // ENSG00000215788 | e26 | Unknown | up | 2.12 | 5.68E-03 | down | 1.77 | 2.37E-02 | No |
| *CXCL12* | DQ345520 | ENSG00000107562 | e4 | exon_skipping,alternative_last_exon | up | 2.11 | 1.87E-02 | down | 6.02 | 1.65E-12 | No |
| *PHLPP2* | AB023148 | ENSG00000040199 | e24 | exon_skipping | up | 2.10 | 1.50E-03 | down | 2.41 | 6.14E-05 | No |
| *NBEAL1* | AK127772 | ENSG00000144426 | ae19 | alternative_last_exon | up | 2.10 | 1.48E-03 | down | 1.57 | 3.18E-02 | Yes |
| *FMO5* | AK225739 | ENSG00000131781 | ae3 | alternative_last_exon | up | 2.08 | 2.44E-02 | down | 2.22 | 1.63E-02 | No |
| *MYLK* | BC064695 | ENSG00000065534 | e2 | Unknown | up | 2.06 | 3.02E-02 | down | 2.43 | 2.40E-10 | No |
| *ABCA8* | AB020629 | ENSG00000141338 | e27 | exon_skipping | up | 2.06 | 4.82E-04 | down | 4.33 | 9.69E-09 | No |
| *EPHA3* | AF213459 | ENSG00000044524 | e1 | alternative_first_exon | up | 2.06 | 7.62E-05 | down | 3.23 | 9.53E-08 | No |
| *CP* | AK299272 | ENSG00000047457 | e15 | Unknown | up | 2.06 | 3.89E-05 | down | 4.94 | 2.34E-06 | No |
| *GPM6B* | AB209525 | ENSG00000046653 | e1 | alternative_first_exon | up | 2.06 | 8.57E-04 | down | 2.41 | 1.62E-04 | No |
| *TMIGD1* | AK172838 | ENSG00000182271 | e6 | Unknown | up | 2.05 | 2.57E-04 | down | 17.34 | 1.69E-11 | No |
| *SLC6A19* | AK096054 | ENSG00000174358 | e9 | Unknown | up | 2.04 | 2.20E-04 | down | 7.92 | 1.09E-13 | No |
| *CHRNA3* | BC006114 | ENSG00000080644 | e6 | exon_skipping,alternative_last_exon | up | 2.04 | 5.62E-05 | down | 3.29 | 1.21E-08 | Yes |
| *EFHD1* | AK310565 | ENSG00000115468 | e4 | alternative_first_exon | up | 2.04 | 3.92E-02 | down | 2.47 | 2.36E-03 | No |
| *CHRDL1* | BC002909 | ENSG00000101938 | e10 | Unknown | up | 2.03 | 1.86E-12 | down | 7.10 | 1.14E-10 | No |
| *ANK2* | BX537758 | ENSG00000145362 | e26 | Unknown | up | 2.02 | 3.74E-03 | down | 3.62 | 4.10E-08 | No |
| *MUSK* | BC109099 | ENSG00000030304 | e13 | Unknown | up | 2.02 | 1.65E-12 | down | 3.43 | 4.22E-07 | No |
| *MFAP4* | CR613174 | ENSG00000166482 | e2 | alternative_first_exon | up | 2.02 | 4.99E-02 | down | 4.22 | 2.78E-03 | No |
| *VIP* | M36634 | ENSG00000146469 | e2 | Unknown | up | 2.00 | 8.81E-04 | down | 10.15 | 5.80E-12 | No |
| *ZEB2* | AB056507 | ENSG00000169554 | ae6 | alternative_last_exon | up | 2.00 | 1.24E-02 | down | 3.81 | 1.44E-03 | No |
| *SIGLEC1* | BC141884 | ENSG00000088827 | ae9 | intron_retention | up | 1.99 | 7.90E-03 | down | 2.43 | 3.37E-04 | No |
| *PLEKHG5 // TNFRSF25* | BC036671 | ENSG00000171680 // ENSG00000215788 | ae20 | intron_retention | up | 1.99 | 8.22E-03 | down | 2.03 | 1.40E-03 | Yes |
| *CFH* | AK291395 | ENSG00000000971 | e17 | Unknown | up | 1.98 | 3.13E-02 | down | 3.70 | 7.96E-03 | No |
| *JAZF1* | AX746909 | ENSG00000153814 | e3 | Unknown | up | 1.98 | 4.54E-03 | down | 2.23 | 2.73E-02 | Yes |
| *PCK1* | BC023978 | ENSG00000124253 | e5 | exon_skipping | up | 1.97 | 2.03E-02 | down | 6.46 | 4.26E-03 | No |
| *SLIT3* | BC098388 | ENSG00000184347 | e24 | Unknown | up | 1.96 | 6.44E-03 | down | 3.58 | 3.83E-05 | No |
| *SLIT3* | BC098388 | ENSG00000184347 | e31 | Unknown | up | 1.96 | 9.01E-04 | down | 3.58 | 3.83E-05 | No |
| *CLIC5* | AK097048 | ENSG00000112782 | e6 | exon_skipping,alternative_first_exon | up | 1.96 | 1.54E-02 | down | 2.34 | 4.78E-04 | Yes |
| *NRXN1* | EF539882 | ENSG00000179915 | e22 | Unknown | up | 1.95 | 6.29E-05 | down | 3.29 | 3.74E-14 | No |
| *ENPP2* | BC034961 | ENSG00000136960 | e22 | exon_skipping | up | 1.95 | 1.02E-03 | down | 2.13 | 5.65E-07 | Yes |
| *CCDC102B* | CR749520 | ENSG00000150636 | e3 | exon_skipping | up | 1.95 | 3.84E-02 | down | 1.90 | 9.50E-03 | Yes |
| *---* | Y17868 | --- | e6 | Unknown | up | 1.93 | 4.02E-03 | down | 3.41 | 5.53E-08 | No |
| *GLI3* | AK308429 | ENSG00000106571 | e3 | Unknown | up | 1.93 | 2.80E-03 | down | 2.96 | 6.39E-04 | No |
| *PLEKHG5 // TNFRSF25* | BC036671 | ENSG00000171680 // ENSG00000215788 | e22 | Unknown | up | 1.93 | 1.71E-02 | down | 1.77 | 2.37E-02 | No |
| *ABCA8* | AB020629 | ENSG00000141338 | e39 | exon_skipping | up | 1.92 | 6.78E-03 | down | 4.33 | 9.69E-09 | No |
| *SCN9A* | X82835 | ENSG00000169432 | e1 | alternative_first_exon | up | 1.92 | 1.78E-03 | down | 3.30 | 3.00E-07 | No |
| *ABCA6* | AY028898 | ENSG00000154262 | e17 | Unknown | up | 1.92 | 2.63E-04 | down | 2.68 | 5.42E-07 | No |
| *KIF5C* | AB011103 | ENSG00000168280 | e13 | Unknown | up | 1.92 | 1.22E-03 | down | 2.54 | 8.02E-07 | Yes |
| *SIGLEC1* | BC141884 | ENSG00000088827 | e19 | Unknown | up | 1.92 | 1.12E-02 | down | 2.43 | 3.37E-04 | No |
| *MAPK10* | BC051731 | ENSG00000109339 | e1 | alternative_first_exon | up | 1.92 | 4.88E-02 | down | 2.15 | 1.65E-02 | No |
| *MYH11* | EU489063 | ENSG00000133392 | e31 | exon_skipping,alternative_first_exon | up | 1.91 | 3.70E-03 | down | 5.37 | 1.65E-12 | No |
| *PTPRR* | D64053 | ENSG00000153233 | ae6 | alternative_first_exon | up | 1.90 | 9.68E-03 | down | 4.55 | 1.68E-12 | No |
| *THBS2* | L12350 | ENSG00000186340 | e8 | Unknown | up | 1.90 | 3.75E-02 | down | 3.26 | 4.95E-08 | Yes |
| *GPNMB* | X76534 | ENSG00000136235 | e6 | Unknown | up | 1.89 | 2.63E-04 | down | 3.57 | 7.02E-03 | No |
| *SYNE1* | AF495910 | ENSG00000131018 | e24 | Unknown | up | 1.89 | 1.26E-03 | down | 1.76 | 1.23E-02 | Yes |
| *GSN* | AK125819 | ENSG00000148180 | e5 | Unknown | up | 1.89 | 4.25E-02 | down | 1.88 | 1.92E-02 | Yes |
| *ANK3* | BX648574 | ENSG00000151150 | e27 | exon_skipping | up | 1.89 | 2.28E-03 | down | 1.65 | 3.97E-02 | Yes |
| *DES* | BC032116 | ENSG00000175084 | e9 | alternative_last_exon | up | 1.88 | 1.22E-02 | down | 4.21 | 3.03E-12 | No |
| *GNAO1* | BC030027 | ENSG00000087258 | e8 | exon_skipping,alternative_last_exon | up | 1.88 | 3.62E-05 | down | 2.49 | 4.42E-05 | No |
| *PLEKHG5 // TNFRSF25* | BC036671 | ENSG00000171680 // ENSG00000215788 | e11 | exon_skipping | up | 1.88 | 7.66E-03 | down | 2.03 | 1.40E-03 | Yes |
| *SVEP1* | AY243503 | ENSG00000165124 | e36 | exon_skipping | up | 1.88 | 4.89E-02 | down | 2.01 | 2.34E-02 | Yes |
| *ALPI* | BC132678 | ENSG00000163295 | e10 | Unknown | up | 1.87 | 1.00E-03 | down | 3.79 | 4.71E-09 | No |
| *GPM6B* | AB209525 | ENSG00000046653 | e9 | Unknown | up | 1.87 | 3.36E-02 | down | 2.41 | 1.62E-04 | Yes |
| *GGTA1* | AF378123 | ENSG00000204136 | e8 | exon_skipping | up | 1.87 | 1.24E-02 | down | 2.25 | 1.52E-03 | Yes |
| *JAM2* | AK294769 | ENSG00000154721 | e12 | alternative_last_exon | up | 1.86 | 1.19E-02 | down | 4.53 | 1.26E-03 | No |
| *PLEKHG5 // TNFRSF25* | BC036671 | ENSG00000171680 // ENSG00000215788 | e25 | Unknown | up | 1.86 | 1.29E-02 | down | 1.77 | 2.37E-02 | No |
| *C7* | BC041807 | ENSG00000112936 | e12 | Unknown | up | 1.85 | 1.02E-03 | down | 4.85 | 7.16E-09 | No |
| *BMP3* | M22491 | ENSG00000152785 | e1 | alternative_first_exon | up | 1.85 | 2.46E-03 | down | 5.00 | 1.07E-08 | No |
| *FMO5* | AK225739 | ENSG00000131781 | ae1 | alternative_donor_splice_site,intron_retention | up | 1.85 | 4.69E-02 | down | 2.22 | 1.63E-02 | Yes |
| *GSTM2* | BC017836 | ENSG00000213366 | e1 | alternative_first_exon | up | 1.84 | 3.96E-02 | down | 1.97 | 2.42E-08 | Yes |
| *PLEKHG5 // TNFRSF25* | BC036671 | ENSG00000171680 // ENSG00000215788 | e30 | Unknown | up | 1.84 | 7.74E-03 | down | 2.03 | 1.40E-03 | Yes |
| *SLC15A1* | U21936 | ENSG00000088386 | e23 | Unknown | up | 1.84 | 2.71E-02 | down | 2.94 | 4.22E-03 | No |
| *SCIN* | AB067492 | ENSG00000006747 | e8 | Unknown | up | 1.84 | 1.15E-02 | down | 2.10 | 2.98E-02 | No |
| *SCN9A* | X82835 | ENSG00000169432 | e4 | exon_skipping | up | 1.83 | 3.38E-04 | down | 3.30 | 3.00E-07 | No |
| *ADH1A // ADH1B* | BT019812 | ENSG00000187758 // ENSG00000196616 | e13 | Unknown | up | 1.83 | 3.15E-02 | down | 11.08 | 8.65E-07 | No |
| *OSBPL6* | AB208898 | ENSG00000079156 | e21 | exon_skipping | up | 1.83 | 2.41E-05 | down | 1.87 | 7.71E-05 | Yes |
| *STAB1* | BC150250 | ENSG00000010327 | e50 | Unknown | up | 1.83 | 2.63E-02 | down | 1.86 | 6.96E-03 | Yes |
| *SLC6A19* | AK096054 | ENSG00000174358 | e12 | alternative_last_exon | up | 1.82 | 2.79E-04 | down | 7.92 | 1.09E-13 | No |
| *KIF5C* | AB011103 | ENSG00000168280 | e26 | Unknown | up | 1.82 | 3.96E-05 | down | 2.54 | 8.02E-07 | Yes |
| *CHL1* | AF002246 | ENSG00000134121 | e29 | Unknown | up | 1.82 | 1.16E-02 | down | 2.34 | 3.52E-05 | Yes |
| *NTM* | AY358331 | ENSG00000182667 | e11 | exon_skipping | up | 1.82 | 5.70E-03 | down | 2.42 | 1.78E-04 | No |
| *THRB* | AK096628 | ENSG00000151090 | ae4 | alternative_acceptor_splice_site | up | 1.82 | 2.19E-02 | down | 2.59 | 1.20E-03 | No |
| *CCL13* | U59808 | ENSG00000181374 | e1 | alternative_first_exon | up | 1.82 | 2.45E-02 | down | 2.05 | 1.24E-02 | Yes |
| *AXL* | BC032229 | ENSG00000167601 | e11 | exon_skipping | up | 1.82 | 2.65E-02 | down | 1.79 | 1.44E-02 | Yes |
| *RGL1* | AF186780 | ENSG00000143344 | e3 | Unknown | up | 1.82 | 9.20E-03 | down | 1.93 | 1.69E-02 | Yes |
| *CADM3* | AF363367 | ENSG00000162706 | e9 | Unknown | up | 1.81 | 4.60E-03 | down | 3.84 | 3.94E-11 | No |
| *SV2B* | AK294902 | ENSG00000185518 | e13 | Unknown | up | 1.81 | 2.82E-04 | down | 2.01 | 5.71E-07 | No |
| *ZFHX4* | AB083343 | ENSG00000091656 | e6 | Unknown | up | 1.81 | 5.94E-03 | down | 2.18 | 2.23E-04 | Yes |
| *WDR17* | AK098748 | ENSG00000150627 | e31 | alternative_last_exon,exon_skipping | up | 1.80 | 7.50E-03 | down | 1.74 | 1.21E-04 | No |
| *DSC3* | D17427 | ENSG00000134762 | e13 | Unknown | up | 1.80 | 5.70E-03 | down | 5.89 | 2.62E-03 | No |
| *---* | CR618029 | --- | e8 | exon_skipping | up | 1.80 | 2.30E-02 | down | 2.34 | 4.58E-02 | No |
| *TMIGD1* | AK172838 | ENSG00000182271 | e3 | Unknown | up | 1.79 | 6.03E-05 | down | 17.34 | 1.69E-11 | No |
| *GLDN* | AK172756 | ENSG00000186417 | e1 | alternative_first_exon | up | 1.79 | 4.81E-04 | down | 3.55 | 1.38E-06 | Yes |
| *EBF1* | BC038805 | ENSG00000164330 | e5 | Unknown | up | 1.79 | 5.28E-03 | down | 2.73 | 1.82E-03 | Yes |
| *FAM70A* | AK056403 | ENSG00000125355 | e10 | Unknown | up | 1.78 | 3.81E-04 | down | 2.28 | 3.30E-06 | No |
| *CA4* | AK298710 | ENSG00000167434 | e3 | Unknown | up | 1.78 | 2.06E-03 | down | 12.30 | 4.49E-05 | No |
| *MEIS1* | BC043503 | ENSG00000143995 | e4 | Unknown | up | 1.78 | 4.14E-03 | down | 3.22 | 1.04E-04 | No |
| *SULT1A2* | U28169 | ENSG00000197165 | e6 | exon_skipping | up | 1.78 | 3.76E-04 | down | 3.07 | 4.15E-04 | No |
| *EDN3* | BC008876 | ENSG00000124205 | e1 | alternative_first_exon | up | 1.78 | 4.60E-02 | down | 3.55 | 1.12E-03 | No |
| *UGT2B17* | U59209 | ENSG00000197888 | e1 | alternative_first_exon | up | 1.78 | 4.54E-02 | down | 6.55 | 3.10E-02 | Yes |
| *SCN9A* | X82835 | ENSG00000169432 | e20 | exon_skipping | up | 1.77 | 3.86E-03 | down | 3.30 | 3.00E-07 | No |
| *MUSK* | BC109099 | ENSG00000030304 | e10 | Unknown | up | 1.77 | 3.68E-03 | down | 3.43 | 4.22E-07 | No |
| *STAB1* | BC150250 | ENSG00000010327 | e27 | Unknown | up | 1.77 | 3.78E-02 | down | 1.86 | 6.96E-03 | Yes |
| *PER3* | BC146781 | ENSG00000049246 | e12 | Unknown | up | 1.77 | 3.13E-02 | down | 2.17 | 1.07E-02 | No |
| *IGF2BP3* | AF117108 | ENSG00000136231 | e12 | Unknown | up | 1.76 | 8.55E-04 | down | 3.80 | 1.57E-10 | Yes |
| *ABCA8* | AB020629 | ENSG00000141338 | e24 | exon_skipping | up | 1.76 | 2.33E-02 | down | 4.33 | 9.69E-09 | No |
| *ANPEP* | AB209918 | ENSG00000166825 | e2 | Unknown | up | 1.76 | 1.23E-02 | down | 3.69 | 2.29E-07 | No |
| *ABCA6* | AY028898 | ENSG00000154262 | e31 | Unknown | up | 1.76 | 6.44E-03 | down | 2.68 | 5.42E-07 | No |
| *TRPM6* | AF350881 | ENSG00000119121 | e19 | exon_skipping | up | 1.76 | 3.59E-02 | down | 7.04 | 5.75E-07 | No |
| *SLIT3* | BC098388 | ENSG00000184347 | e34 | Unknown | up | 1.76 | 2.88E-03 | down | 3.58 | 3.83E-05 | No |
| *MEIS1* | BC043503 | ENSG00000143995 | e14 | exon_skipping | up | 1.76 | 5.24E-04 | down | 3.22 | 1.04E-04 | No |
| *BTNL8* | AY358523 | ENSG00000113303 | ae6 | alternative_donor_splice_site,alternative_last_exon | up | 1.76 | 4.66E-02 | down | 3.44 | 2.58E-03 | No |
| *COL14A1* | BC140893 | ENSG00000187955 | e39 | Unknown | up | 1.76 | 3.30E-02 | down | 3.93 | 4.88E-03 | No |
| *SPIB* | AK225225 | ENSG00000142539 | e2 | Unknown | up | 1.75 | 5.00E-03 | down | 3.35 | 1.62E-12 | No |
| *PCOLCE2* | AK075478 | ENSG00000163710 | e7 | Unknown | up | 1.75 | 1.02E-03 | down | 3.16 | 1.89E-09 | No |
| *ABCA8* | AB020629 | ENSG00000141338 | ae40 | intron_retention | up | 1.75 | 3.63E-02 | down | 4.33 | 9.69E-09 | No |
| *SLIT3* | BC098388 | ENSG00000184347 | e32 | Unknown | up | 1.75 | 1.18E-03 | down | 3.58 | 3.83E-05 | No |
| *ST6GAL2* | AB058780 | ENSG00000144057 | e4 | Unknown | up | 1.75 | 8.84E-04 | down | 3.67 | 2.84E-04 | No |
| *CLCA4* | AK000072 | ENSG00000016602 | e7 | Unknown | up | 1.75 | 1.96E-02 | down | 23.99 | 3.16E-04 | No |
| *UGP2* | CR602817 | ENSG00000169764 | e1 | alternative_first_exon | up | 1.75 | 2.42E-03 | down | 1.89 | 1.06E-03 | No |
| *ADCY5* | BC156217 | ENSG00000173175 | e4 | alternative_first_exon,exon_skipping,exon_skipping | up | 1.75 | 1.70E-02 | down | 1.94 | 1.88E-03 | Yes |
| *---* | Y17868 | --- | e4 | Unknown | up | 1.74 | 2.16E-03 | down | 3.41 | 5.53E-08 | No |
| *PCSK2* | BC040546 | ENSG00000125851 | e8 | Unknown | up | 1.74 | 8.94E-03 | down | 2.04 | 7.39E-04 | Yes |
| *MCOLN2* | AX748410 | ENSG00000153898 | e1 | alternative_first_exon | up | 1.74 | 3.80E-02 | down | 2.98 | 9.02E-03 | No |
| *OTOP2* | BC152986 | ENSG00000183034 | e2 | Unknown | up | 1.73 | 4.21E-04 | down | 19.34 | 4.02E-13 | No |
| *NAALADL1* | BC143644 | ENSG00000168060 | e16 | Unknown | up | 1.73 | 1.01E-04 | down | 4.22 | 1.93E-09 | No |
| *FAM5C* | AB111893 | ENSG00000162670 | e3 | exon_skipping,alternative_first_exon | up | 1.73 | 4.82E-03 | down | 3.68 | 2.04E-05 | No |
| *CA4* | AK298710 | ENSG00000167434 | e2 | Unknown | up | 1.73 | 3.76E-03 | down | 12.30 | 4.49E-05 | No |
| *---* | AK126481 | --- | e1 | alternative_first_exon | up | 1.73 | 2.04E-05 | down | 2.49 | 5.79E-05 | No |
| *JAM2* | AK294769 | ENSG00000154721 | e2 | Unknown | up | 1.73 | 3.43E-02 | down | 4.53 | 1.26E-03 | No |
| *RGL1* | AF186780 | ENSG00000143344 | e20 | Unknown | up | 1.73 | 9.06E-03 | down | 1.93 | 1.69E-02 | No |
| *PCOLCE2* | AK075478 | ENSG00000163710 | e8 | Unknown | up | 1.72 | 5.14E-03 | down | 3.16 | 1.89E-09 | No |
| *ANK2* | BX537758 | ENSG00000145362 | e18 | Unknown | up | 1.72 | 7.28E-03 | down | 3.62 | 4.10E-08 | No |
| *ABCA6* | AY028898 | ENSG00000154262 | e22 | Unknown | up | 1.72 | 1.55E-02 | down | 2.68 | 5.42E-07 | No |
| *MST1P2 // MST1P9* | AY192149 | ENSG00000186715 | ae7 | intron_retention | up | 1.72 | 8.64E-03 | down | 2.35 | 6.37E-05 | Yes |
| *CES1* | AK292209 | ENSG00000198848 | e3 | Unknown | up | 1.72 | 5.57E-04 | down | 2.37 | 8.31E-05 | Yes |
| *ACTG2* | BC012617 | ENSG00000163017 | e10 | Unknown | up | 1.72 | 1.80E-02 | down | 2.70 | 3.87E-02 | No |
| *CHGA* | BC006459 | ENSG00000100604 | e6 | exon_skipping | up | 1.71 | 2.72E-02 | down | 7.65 | 1.63E-12 | No |
| *CA7* | AY075019 | ENSG00000168748 | e8 | alternative_last_exon | up | 1.71 | 6.18E-03 | down | 15.61 | 1.79E-08 | No |
| *TRPM6* | AF350881 | ENSG00000119121 | e39 | Unknown | up | 1.71 | 1.46E-02 | down | 7.04 | 5.75E-07 | No |
| *SCN9A* | X82835 | ENSG00000169432 | e7 | exon_skipping | up | 1.71 | 1.22E-02 | down | 2.74 | 2.67E-06 | Yes |
| *SCARA3* | BC060811 | ENSG00000168077 | e4 | Unknown | up | 1.71 | 1.10E-03 | down | 1.70 | 3.83E-06 | Yes |
| *MST1P2 // MST1P9* | AY192149 | ENSG00000186715 | e11 | exon_skipping | up | 1.71 | 1.68E-02 | down | 2.35 | 6.37E-05 | Yes |
| *LYVE1* | AF127670 | ENSG00000133800 | e6 | alternative_last_exon | up | 1.71 | 1.45E-02 | down | 8.39 | 2.03E-04 | No |
| *CNTN4* | AK125460 | ENSG00000144619 | e8 | Unknown | up | 1.71 | 2.84E-03 | down | 2.48 | 3.77E-04 | No |
| *SLC4A4* | AF011390 | ENSG00000080493 | e9 | Unknown | up | 1.71 | 9.60E-03 | down | 9.06 | 1.02E-03 | No |
| *---* | AK297065 | --- | e2 | Unknown | up | 1.71 | 4.16E-05 | down | 24.88 | 2.58E-03 | No |
| *---* | AK308167 | --- | e31 | alternative_last_exon | up | 1.71 | 8.36E-08 | down | 2.49 | 1.49E-02 | Yes |
| *ABCA8* | AB020629 | ENSG00000141338 | e36 | exon_skipping | up | 1.70 | 2.11E-02 | down | 4.33 | 9.69E-09 | No |
| *FABP4* | BC003672 | ENSG00000170323 | e3 | Unknown | up | 1.70 | 2.02E-03 | down | 3.98 | 2.42E-07 | No |
| *CP* | AK299272 | ENSG00000047457 | e18 | exon_skipping | up | 1.70 | 3.78E-04 | down | 4.12 | 1.74E-06 | Yes |
| *MEIS1* | BC043503 | ENSG00000143995 | ae13 | intron_retention | up | 1.70 | 3.74E-02 | down | 3.22 | 1.04E-04 | No |
| *ADC* | AK095127 | ENSG00000142920 | ae11 | alternative_acceptor_splice_site | up | 1.70 | 1.30E-02 | down | 1.64 | 1.98E-03 | Yes |
| *EBF1* | BC038805 | ENSG00000164330 | e3 | alternative_first_exon | up | 1.70 | 4.01E-02 | down | 2.65 | 2.64E-03 | No |
| *SLC26A3* | BX640837 | ENSG00000091138 | e12 | Unknown | up | 1.70 | 3.41E-04 | down | 11.92 | 7.14E-03 | No |
| *CLCA4* | AK000072 | ENSG00000016602 | e3 | exon_skipping | up | 1.69 | 3.24E-02 | down | 23.99 | 3.16E-04 | No |
| *GPC6* | AF105267 | ENSG00000183098 | e4 | Unknown | up | 1.69 | 6.36E-03 | down | 3.10 | 1.36E-03 | No |
| *C4orf7* | AY190326 | ENSG00000181617 | e5 | alternative_last_exon | up | 1.69 | 1.86E-02 | down | 2.40 | 2.36E-03 | No |
| *MCOLN2* | AX748410 | ENSG00000153898 | e15 | alternative_last_exon | up | 1.69 | 1.17E-02 | down | 2.98 | 9.02E-03 | No |
| *SLC17A4* | AK024903 | ENSG00000146039 | e8 | exon_skipping | up | 1.69 | 2.72E-07 | down | 4.29 | 9.50E-03 | No |
| *CFHR1* | BC107771 | ENSG00000244414 | e4 | Unknown | up | 1.69 | 4.64E-02 | down | 2.77 | 2.38E-02 | No |
| *IFI44L* | AK223087 | ENSG00000137959 | e1 | alternative_first_exon | up | 1.69 | 3.94E-03 | down | 2.56 | 2.74E-02 | No |
| *ADAMDEC1* | AK316320 | ENSG00000134028 | e9 | Unknown | up | 1.68 | 1.55E-04 | down | 7.86 | 1.80E-05 | No |
| *SLIT3* | BC098388 | ENSG00000184347 | e13 | alternative_last_exon | up | 1.68 | 4.38E-03 | down | 3.58 | 3.83E-05 | No |
| *HIGD1A* | AL110233 | ENSG00000181061 | e1 | alternative_first_exon | up | 1.68 | 4.46E-02 | down | 1.85 | 2.27E-02 | Yes |
| *IGF2BP3* | AF117108 | ENSG00000136231 | e17 | Unknown | up | 1.67 | 1.85E-02 | down | 3.67 | 4.27E-07 | No |
| *ASPA* | AK312901 | ENSG00000108381 | e6 | Unknown | up | 1.67 | 6.04E-03 | down | 3.23 | 2.05E-06 | No |
| *MUC5B* | AF086604 | ENSG00000117983 | e13 | Unknown | up | 1.67 | 3.06E-03 | down | 3.86 | 3.68E-03 | No |
| *MFSD4* | AX746641 | ENSG00000174514 | e8 | exon_skipping | up | 1.67 | 3.55E-02 | down | 3.30 | 8.48E-03 | No |
| *SYNE1* | AF495910 | ENSG00000131018 | e19 | Unknown | up | 1.67 | 4.31E-02 | down | 1.76 | 1.23E-02 | No |
| *ROR2* | BC033697 | ENSG00000169071 | e14 | Unknown | up | 1.67 | 2.99E-08 | down | 1.72 | 1.42E-02 | No |
| *C7* | BC041807 | ENSG00000112936 | e3 | Unknown | up | 1.66 | 4.08E-03 | down | 4.85 | 7.16E-09 | No |
| *---* | Y17868 | --- | e3 | Unknown | up | 1.66 | 4.21E-02 | down | 3.41 | 5.53E-08 | No |
| *DOCK2* | D86964 | ENSG00000134516 | e50 | Unknown | up | 1.66 | 3.74E-02 | down | 1.68 | 1.16E-06 | Yes |
| *ADAMDEC1* | AK316320 | ENSG00000134028 | e4 | Unknown | up | 1.66 | 2.09E-02 | down | 7.86 | 1.80E-05 | No |
| *ADAMTSL3* | BC128390 | ENSG00000156218 | e30 | Unknown | up | 1.66 | 2.67E-02 | down | 2.49 | 3.83E-04 | No |
| *TMOD2* | BC064961 | ENSG00000128872 | e11 | Unknown | up | 1.66 | 2.52E-12 | down | 3.20 | 5.03E-04 | No |
| *ITIH5* | AY358426 | ENSG00000123243 | e5 | Unknown | up | 1.66 | 1.69E-02 | down | 3.66 | 9.80E-03 | No |
| *AKAP9* | AJ131693 | ENSG00000127914 | e13 | Unknown | up | 1.66 | 1.25E-02 | down | 1.57 | 3.87E-02 | Yes |
| *GLDN* | AK172756 | ENSG00000186417 | e7 | Unknown | up | 1.65 | 1.31E-02 | down | 3.55 | 1.38E-06 | No |
| *GLDN* | AK172756 | ENSG00000186417 | e6 | Unknown | up | 1.65 | 3.44E-03 | down | 3.55 | 1.38E-06 | No |
| *COL1A2* | J03464 | --- | e16 | exon_skipping | up | 1.65 | 4.14E-06 | down | 1.62 | 2.80E-04 | Yes |
| *MS4A12* | AK000224 | ENSG00000071203 | e5 | Unknown | up | 1.65 | 2.78E-02 | down | 24.59 | 7.63E-04 | No |
| *FN1* | CR749317 | ENSG00000115414 | e42 | exon_skipping | up | 1.65 | 5.70E-07 | down | 5.23 | 1.76E-02 | No |
| *NR5A2* | AB019246 | ENSG00000116833 | e2 | exon_skipping | up | 1.65 | 3.38E-08 | down | 2.51 | 2.62E-02 | No |
| *HOXA11AS* | BC025338 | ENSG00000240990 | e3 | alternative_last_exon | up | 1.65 | 4.40E-03 | down | 1.65 | 3.70E-02 | Yes |
| *OTOP2* | BC152986 | ENSG00000183034 | e7 | Unknown | up | 1.64 | 4.46E-03 | down | 19.34 | 4.02E-13 | No |
| *GDPD2* | AB048363 | ENSG00000130055 | e5 | Unknown | up | 1.64 | 1.79E-02 | down | 2.13 | 6.11E-10 | No |
| *SFRP2* | AF311912 | ENSG00000145423 | e3 | Unknown | up | 1.64 | 1.20E-03 | down | 12.43 | 9.32E-09 | Yes |
| *CA7* | AY075019 | ENSG00000168748 | e7 | Unknown | up | 1.64 | 1.63E-02 | down | 15.61 | 1.79E-08 | No |
| *ANK2* | BX537758 | ENSG00000145362 | e51 | exon_skipping | up | 1.64 | 2.26E-02 | down | 3.62 | 4.10E-08 | No |
| *IGF2BP3* | AF117108 | ENSG00000136231 | e16 | Unknown | up | 1.64 | 1.85E-02 | down | 3.67 | 4.27E-07 | No |
| *GCG* | BC005278 | ENSG00000115263 | e4 | Unknown | up | 1.64 | 3.44E-04 | down | 27.59 | 4.00E-06 | No |
| *SLIT2* | AF055585 | ENSG00000145147 | e22 | Unknown | up | 1.64 | 1.43E-02 | down | 2.53 | 1.97E-05 | No |
| *SCNN1B* | BC036352 | ENSG00000168447 | e4 | Unknown | up | 1.64 | 2.62E-03 | down | 9.33 | 1.58E-04 | No |
| *ADAMTSL3* | BC128390 | ENSG00000156218 | e28 | Unknown | up | 1.64 | 5.70E-03 | down | 2.49 | 3.83E-04 | No |
| *PLEKHH2* | AL832207 | ENSG00000152527 | e24 | Unknown | up | 1.64 | 3.45E-02 | down | 2.14 | 2.53E-02 | Yes |
| *PDLIM3* | AF039018 | ENSG00000154553 | e5 | exon_skipping,alternative_last_exon | up | 1.64 | 1.40E-04 | down | 2.46 | 2.69E-02 | No |
| *EPHA3* | AF213459 | ENSG00000044524 | e7 | Unknown | up | 1.63 | 1.01E-02 | down | 3.23 | 9.53E-08 | No |
| *SCNN1B* | BC036352 | ENSG00000168447 | e13 | Unknown | up | 1.63 | 1.15E-02 | down | 9.33 | 1.58E-04 | No |
| *STON2* | AB208948 | ENSG00000140022 | e9 | alternative_last_exon | up | 1.63 | 2.91E-02 | down | 1.65 | 1.09E-02 | Yes |
| *ADAM28* | AK225921 | ENSG00000042980 | e1 | alternative_first_exon | up | 1.63 | 2.78E-02 | down | 2.10 | 2.76E-02 | No |
| *FGFR1* | FJ809917 | ENSG00000077782 | e22 | Unknown | up | 1.63 | 4.99E-02 | down | 2.28 | 3.21E-02 | No |
| *FBLN2* | AK304777 | ENSG00000163520 | e11 | exon_skipping | up | 1.62 | 2.71E-02 | down | 4.22 | 3.23E-07 | No |
| *ANK2* | BX537758 | ENSG00000145362 | e14 | Unknown | up | 1.61 | 3.76E-03 | down | 3.62 | 4.10E-08 | No |
| *SV2B* | AK294902 | ENSG00000185518 | e8 | Unknown | up | 1.61 | 1.66E-03 | down | 2.01 | 5.71E-07 | No |
| *CP* | AK299272 | ENSG00000047457 | e16 | Unknown | up | 1.61 | 2.81E-04 | down | 4.94 | 2.34E-06 | No |
| *FAM5C* | AB111893 | ENSG00000162670 | e10 | alternative_last_exon | up | 1.61 | 9.96E-04 | down | 3.68 | 2.04E-05 | No |
| *---* | AK122718 | --- | e1 | alternative_first_exon | up | 1.61 | 3.22E-02 | down | 3.92 | 3.75E-05 | No |
| *LYVE1* | AF127670 | ENSG00000133800 | e5 | Unknown | up | 1.61 | 1.17E-02 | down | 8.39 | 2.03E-04 | No |
| *CACNB1* | M92303 | ENSG00000067191 | e16 | alternative_last_exon | up | 1.61 | 1.10E-02 | down | 2.24 | 9.36E-04 | No |
| *PLEKHG5 // TNFRSF25* | BC036671 | ENSG00000171680 // ENSG00000215788 | e36 | exon_skipping,exon_skipping,exon_skipping | up | 1.61 | 2.06E-03 | down | 2.03 | 1.40E-03 | Yes |
| *MGP* | CR623037 | ENSG00000111341 | e4 | alternative_last_exon | up | 1.61 | 9.90E-03 | down | 5.30 | 2.98E-03 | No |
| *STON2* | AB208948 | ENSG00000140022 | e7 | Unknown | up | 1.61 | 4.25E-02 | down | 1.65 | 1.09E-02 | Yes |
| *SULT1A1* | CR601418 | ENSG00000196502 | e11 | alternative_last_exon | up | 1.61 | 2.07E-02 | down | 2.40 | 1.52E-02 | Yes |
| *MS4A6A* | AK291870 | ENSG00000110077 | ae8 | intron_retention,alternative_last_exon | up | 1.61 | 1.52E-03 | down | 2.17 | 2.07E-02 | No |
| *SORBS1* | BC152463 | ENSG00000095637 | e5 | Unknown | up | 1.61 | 1.76E-02 | down | 1.79 | 2.35E-02 | Yes |
| *DPT* | BC033736 | ENSG00000143196 | e1 | alternative_first_exon | up | 1.60 | 1.70E-12 | down | 5.73 | 1.88E-11 | No |
| *A2M* | CR749334 | ENSG00000175899 | e4 | exon_skipping | up | 1.60 | 3.32E-03 | down | 2.26 | 3.40E-08 | Yes |
| *ABCA9* | AY028899 | ENSG00000154258 | e20 | Unknown | up | 1.60 | 2.99E-02 | down | 2.13 | 4.49E-08 | Yes |
| *THBS2* | L12350 | ENSG00000186340 | e15 | Unknown | up | 1.60 | 4.31E-02 | down | 3.26 | 4.95E-08 | Yes |
| *---* | BC160029 | --- | e5 | Unknown | up | 1.60 | 2.09E-02 | down | 1.53 | 2.37E-07 | Yes |
| *---* | BC089413 | --- | ae37 | alternative_first_exon | up | 1.60 | 2.22E-05 | down | 2.36 | 2.43E-05 | Yes |
| *FBLN1* | AK075566 | ENSG00000077942 | e7 | Unknown | up | 1.60 | 2.29E-02 | down | 4.44 | 1.88E-03 | No |
| *ADC* | AK095127 | ENSG00000142920 | e12 | exon_skipping,alternative_last_exon | up | 1.60 | 9.28E-03 | down | 1.64 | 1.98E-03 | Yes |
| *ALPPL2* | J04948 | ENSG00000163286 | e11 | alternative_last_exon | up | 1.60 | 3.12E-03 | down | 1.97 | 2.84E-03 | No |
| *MAPK10* | BC051731 | ENSG00000109339 | e9 | exon_skipping | up | 1.60 | 3.49E-02 | down | 2.15 | 1.65E-02 | No |
| *ABCA8* | AB020629 | ENSG00000141338 | e29 | exon_skipping | up | 1.59 | 2.42E-02 | down | 4.33 | 9.69E-09 | No |
| *DAAM2* | AB002379 | ENSG00000146122 | e27 | exon_skipping,alternative_last_exon | up | 1.59 | 1.62E-02 | down | 1.59 | 2.07E-07 | No |
| *CHL1* | AF002246 | ENSG00000134121 | e11 | Unknown | up | 1.59 | 7.64E-03 | down | 2.34 | 3.52E-05 | No |
| *IGFBP6* | BC010162 | ENSG00000167779 | e1 | alternative_first_exon | up | 1.59 | 2.92E-03 | down | 2.47 | 3.79E-05 | No |
| *---* | BC073771 | --- | e146 | exon_skipping | up | 1.59 | 1.56E-03 | down | 2.08 | 1.82E-04 | No |
| *LYVE1* | AF127670 | ENSG00000133800 | ae6 | alternative_last_exon | up | 1.59 | 6.28E-03 | down | 8.39 | 2.03E-04 | No |
| *COL1A2* | J03464 | --- | e13 | exon_skipping | up | 1.59 | 1.54E-02 | down | 1.62 | 2.80E-04 | Yes |
| *GPNMB* | X76534 | ENSG00000136235 | e8 | Unknown | up | 1.59 | 4.30E-02 | down | 3.57 | 7.02E-03 | No |
| *SYNE1* | AF495910 | ENSG00000131018 | e88 | Unknown | up | 1.59 | 2.18E-02 | down | 1.76 | 1.23E-02 | Yes |
| *STAP1* | BC014958 | ENSG00000035720 | e8 | Unknown | up | 1.59 | 4.44E-02 | down | 2.01 | 2.90E-02 | Yes |
| *BMP8B* | AY303955 | ENSG00000116985 | e4 | exon_skipping | up | 1.59 | 4.69E-02 | down | 1.50 | 3.66E-02 | Yes |
| *C20orf194* | AK226100 | ENSG00000088854 | e4 | Unknown | up | 1.58 | 3.75E-02 | down | 2.04 | 6.83E-10 | No |
| *MMRN1* | AK125557 | ENSG00000138722 | e3 | Unknown | up | 1.58 | 9.40E-03 | down | 3.21 | 8.22E-04 | No |
| *KLRD1* | BC042884 | ENSG00000134539 | e1 | alternative_first_exon | up | 1.58 | 3.54E-02 | down | 1.89 | 1.48E-03 | Yes |
| *GPNMB* | X76534 | ENSG00000136235 | e7 | Unknown | up | 1.58 | 3.81E-02 | down | 3.57 | 7.02E-03 | No |
| *DPYD* | U20938 | ENSG00000188641 | e17 | Unknown | up | 1.58 | 1.96E-02 | down | 2.26 | 1.14E-02 | No |
| *C16orf89* | AY358483 | ENSG00000153446 | e4 | Unknown | up | 1.58 | 1.02E-02 | down | 1.80 | 3.14E-02 | No |
| *ANK3* | BX648574 | ENSG00000151150 | e7 | exon_skipping | up | 1.58 | 2.61E-02 | down | 1.65 | 3.97E-02 | No |
| *MYH11* | EU489063 | ENSG00000133392 | e27 | exon_skipping | up | 1.57 | 1.32E-02 | down | 5.37 | 1.65E-12 | No |
| *ECM2* | BX537976 | ENSG00000106823 | e12 | exon_skipping,alternative_last_exon | up | 1.57 | 7.79E-04 | down | 2.35 | 1.03E-06 | No |
| *MST1P2 // MST1P9* | AY192149 | ENSG00000186715 | e12 | Unknown | up | 1.57 | 6.99E-04 | down | 2.35 | 6.37E-05 | Yes |
| *OSBPL6* | AB208898 | ENSG00000079156 | e8 | Unknown | up | 1.57 | 5.18E-03 | down | 1.87 | 7.71E-05 | Yes |
| *CCL19* | AB000887 | ENSG00000172724 | e1 | alternative_first_exon | up | 1.57 | 2.89E-02 | down | 2.40 | 3.03E-04 | No |
| *ZEB2* | AB056507 | ENSG00000169554 | e13 | exon_skipping,alternative_last_exon | up | 1.57 | 2.15E-02 | down | 3.81 | 1.44E-03 | No |
| *PKIB* | BC036011 | ENSG00000135549 | e8 | alternative_last_exon | up | 1.57 | 2.64E-02 | down | 4.41 | 3.40E-03 | Yes |
| *COL14A1* | BC140893 | ENSG00000187955 | e38 | Unknown | up | 1.57 | 2.82E-02 | down | 3.93 | 4.88E-03 | No |
| *CFH* | AK291395 | ENSG00000000971 | e20 | Unknown | up | 1.57 | 4.26E-02 | down | 3.67 | 7.20E-03 | Yes |
| *FRZB* | CR622855 | ENSG00000162998 | e2 | Unknown | up | 1.57 | 3.07E-02 | down | 2.48 | 2.59E-02 | No |
| *CADM3* | AF363367 | ENSG00000162706 | e1 | alternative_first_exon | up | 1.56 | 3.84E-03 | down | 3.84 | 3.94E-11 | No |
| *MEP1A* | AF478685 | ENSG00000112818 | e1 | alternative_first_exon | up | 1.56 | 1.98E-02 | down | 3.38 | 5.77E-10 | No |
| *PCOLCE2* | AK075478 | ENSG00000163710 | e6 | Unknown | up | 1.56 | 4.44E-03 | down | 3.16 | 1.89E-09 | No |
| *---* | AK129601 | --- | e1 | alternative_first_exon | up | 1.56 | 1.88E-02 | down | 5.49 | 1.16E-03 | No |
| *NR3C2* | M16801 | ENSG00000151623 | e1 | alternative_first_exon | up | 1.56 | 4.36E-03 | down | 2.17 | 1.54E-03 | Yes |
| *NBPF11* | AL832622 | ENSG00000152042 // ENSG00000203836 | e3 | exon_skipping | up | 1.56 | 8.25E-04 | down | 1.63 | 2.99E-02 | Yes |
| *MAMDC2* | BC045569 | ENSG00000165072 | e12 | Unknown | up | 1.55 | 3.14E-03 | down | 4.07 | 1.07E-07 | No |
| *C21orf88* | AF426266 | ENSG00000184809 | e1 | alternative_first_exon | up | 1.55 | 7.71E-05 | down | 10.82 | 1.74E-07 | No |
| *CHGA* | BC006459 | ENSG00000100604 | e8 | alternative_last_exon | up | 1.55 | 2.60E-03 | down | 7.78 | 1.66E-05 | Yes |
| *GNAO1* | BC030027 | ENSG00000087258 | e2 | Unknown | up | 1.55 | 3.61E-02 | down | 2.49 | 4.42E-05 | No |
| *SLC4A4* | AF011390 | ENSG00000080493 | e23 | Unknown | up | 1.55 | 3.57E-09 | down | 9.06 | 1.02E-03 | No |
| *PLEKHH2* | AL832207 | ENSG00000152527 | e31 | Unknown | up | 1.55 | 2.02E-02 | down | 2.14 | 2.53E-02 | No |
| *SCN9A* | X82835 | ENSG00000169432 | ae28 | alternative_acceptor_splice_site | up | 1.54 | 1.79E-02 | down | 3.30 | 3.00E-07 | No |
| *CSF1R* | X03663 | ENSG00000182578 | e13 | Unknown | up | 1.54 | 1.72E-02 | down | 1.78 | 5.81E-07 | Yes |
| *CP* | AK299272 | ENSG00000047457 | e11 | exon_skipping,alternative_last_exon | up | 1.54 | 1.53E-02 | down | 4.94 | 2.34E-06 | No |
| *GUCA2A* | M97496 | ENSG00000197273 | e3 | alternative_last_exon | up | 1.54 | 1.36E-03 | down | 18.57 | 1.51E-05 | No |
| *SLIT3* | BC098388 | ENSG00000184347 | e18 | Unknown | up | 1.54 | 4.50E-03 | down | 3.58 | 3.83E-05 | No |
| *CA4* | AK298710 | ENSG00000167434 | e8 | alternative_last_exon,exon_skipping | up | 1.54 | 2.18E-02 | down | 12.30 | 4.49E-05 | No |
| *COL14A1* | BC140893 | ENSG00000187955 | e7 | Unknown | up | 1.54 | 1.34E-02 | down | 3.93 | 4.88E-03 | No |
| *ADH1A // ADH1B* | BT019812 | ENSG00000187758 // ENSG00000196616 | e12 | Unknown | up | 1.54 | 1.83E-05 | down | 6.22 | 1.12E-02 | Yes |
| *ROR1* | M97675 | ENSG00000185483 | e7 | Unknown | up | 1.54 | 5.74E-03 | down | 2.49 | 1.17E-02 | No |
| *ANK3* | BX648574 | ENSG00000151150 | e12 | exon_skipping | up | 1.54 | 2.57E-02 | down | 1.65 | 3.97E-02 | Yes |
| *ANK3* | BX648574 | ENSG00000151150 | e26 | exon_skipping | up | 1.54 | 4.50E-02 | down | 1.65 | 3.97E-02 | Yes |
| *ANK2* | BX537758 | ENSG00000145362 | e52 | Unknown | up | 1.53 | 5.46E-03 | down | 3.62 | 4.10E-08 | No |
| *CES1* | AK292209 | ENSG00000198848 | e4 | exon_skipping | up | 1.53 | 3.45E-02 | down | 2.32 | 7.68E-05 | No |
| *HMCN1* | AF156100 | ENSG00000143341 | e44 | Unknown | up | 1.53 | 2.46E-02 | down | 1.88 | 7.15E-04 | No |
| *EBF1* | BC038805 | ENSG00000164330 | e11 | Unknown | up | 1.53 | 6.55E-10 | down | 2.73 | 1.82E-03 | Yes |
| *PRKAR2B* | BC075800 | ENSG00000005249 | e4 | Unknown | up | 1.53 | 1.89E-02 | down | 3.47 | 2.56E-03 | No |
| *DSC3* | D17427 | ENSG00000134762 | e14 | Unknown | up | 1.53 | 3.36E-02 | down | 5.89 | 2.62E-03 | No |
| *ADH1A // ADH1B* | BT019812 | ENSG00000187758 // ENSG00000196616 | e8 | exon_skipping | up | 1.53 | 2.93E-02 | down | 6.22 | 1.12E-02 | Yes |
| *KCTD12* | AK128363 | ENSG00000178695 | e2 | alternative_last_exon | up | 1.53 | 1.52E-02 | down | 2.95 | 1.24E-02 | No |
| *ANK3* | BX648574 | ENSG00000151150 | e2 | exon_skipping | up | 1.53 | 4.58E-02 | down | 1.65 | 3.97E-02 | Yes |
| *ABCA8* | AB020629 | ENSG00000141338 | e35 | exon_skipping | up | 1.52 | 4.20E-02 | down | 4.33 | 9.69E-09 | Yes |
| *ABCG2* | BC092408 | ENSG00000118777 | e15 | Unknown | up | 1.52 | 1.60E-02 | down | 4.44 | 2.38E-08 | No |
| *ABCA9* | AY028899 | ENSG00000154258 | e9 | Unknown | up | 1.52 | 2.09E-02 | down | 2.13 | 4.49E-08 | Yes |
| *MFAP5* | BC005901 | ENSG00000197614 | e3 | Unknown | up | 1.52 | 3.84E-02 | down | 11.16 | 1.45E-06 | No |
| *MST1P2 // MST1P9* | AY192149 | ENSG00000186715 | e7 | Unknown | up | 1.52 | 1.06E-02 | down | 2.35 | 6.37E-05 | Yes |
| *NBPF1* | BC093404 | ENSG00000219481 | e4 | exon_skipping,alternative_last_exon | up | 1.52 | 7.43E-04 | down | 2.06 | 5.05E-04 | Yes |
| *TMOD1* | AK314533 | ENSG00000136842 | e11 | Unknown | up | 1.52 | 1.82E-02 | down | 2.60 | 1.58E-03 | No |
| *SRPX* | AK125542 | ENSG00000101955 | e2 | exon_skipping | up | 1.52 | 2.49E-02 | down | 3.22 | 1.84E-03 | Yes |
| *ADH1A // ADH1B* | BT019812 | ENSG00000187758 // ENSG00000196616 | e7 | exon_skipping | up | 1.52 | 7.02E-03 | down | 6.22 | 1.12E-02 | Yes |
| *FN1* | CR749317 | ENSG00000115414 | e46 | Unknown | up | 1.52 | 1.21E-02 | down | 5.23 | 1.76E-02 | No |
| *SFMBT2* | BC167860 | ENSG00000198879 | e17 | exon_skipping | up | 1.52 | 3.45E-02 | down | 1.93 | 4.02E-02 | Yes |
| *PDE7B* | AJ251860 | ENSG00000171408 | e9 | Unknown | up | 1.52 | 2.06E-02 | down | 1.97 | 4.47E-02 | No |
| *ANK2* | BX537758 | ENSG00000145362 | e21 | Unknown | up | 1.51 | 6.80E-03 | down | 3.62 | 4.10E-08 | No |
| *ANPEP* | AB209918 | ENSG00000166825 | e6 | exon_skipping | up | 1.51 | 2.49E-02 | down | 3.76 | 2.43E-07 | Yes |
| *SCN9A* | X82835 | ENSG00000169432 | e5 | exon_skipping | up | 1.51 | 7.88E-03 | down | 3.30 | 3.00E-07 | No |
| *SLIT3* | BC098388 | ENSG00000184347 | e20 | Unknown | up | 1.51 | 4.23E-02 | down | 3.58 | 3.83E-05 | No |
| *CCL23* | U58913 | ENSG00000167236 | e1 | alternative_first_exon | up | 1.51 | 3.06E-10 | down | 2.39 | 5.86E-05 | Yes |
| *FAM13C* | AK316542 | ENSG00000148541 | e4 | Unknown | up | 1.51 | 1.40E-02 | down | 2.11 | 2.81E-04 | No |
| *IGF1* | X57025 | ENSG00000017427 | e1 | alternative_first_exon | up | 1.51 | 3.19E-02 | down | 2.72 | 1.34E-03 | No |
| *IGF1* | X57025 | ENSG00000017427 | e4 | Unknown | up | 1.51 | 6.61E-04 | down | 2.72 | 1.34E-03 | No |
| *RECK* | D50406 | ENSG00000122707 | e15 | Unknown | up | 1.51 | 3.27E-02 | down | 2.21 | 9.12E-03 | Yes |
| *---* | AK308167 | --- | e26 | Unknown | up | 1.51 | 2.18E-02 | down | 2.49 | 1.49E-02 | Yes |
| *PLEKHH2* | AL832207 | ENSG00000152527 | e16 | alternative_last_exon | up | 1.51 | 2.54E-02 | down | 2.14 | 2.53E-02 | No |
| *AKAP9* | AJ131693 | ENSG00000127914 | e12 | Unknown | up | 1.51 | 3.65E-02 | down | 1.57 | 3.87E-02 | No |
| *RERGL* | BC042888 | ENSG00000111404 | e1 | alternative_first_exon | up | 1.50 | 3.31E-02 | down | 3.34 | 1.93E-09 | No |
| *LDHD* | BC040279 | ENSG00000166816 | e3 | Unknown | up | 1.50 | 4.32E-02 | down | 1.99 | 3.48E-07 | No |
| *ADAMDEC1* | AK316320 | ENSG00000134028 | e1 | alternative_first_exon | up | 1.50 | 4.17E-02 | down | 7.86 | 1.80E-05 | No |
| *---* | AK122718 | --- | e9 | Unknown | up | 1.50 | 4.65E-02 | down | 3.92 | 3.75E-05 | No |
| *ADAMTSL3* | BC128390 | ENSG00000156218 | e14 | Unknown | up | 1.50 | 4.44E-02 | down | 2.49 | 3.83E-04 | No |
| *---* | BC073771 | --- | e111 | exon_skipping | down | 7.13 | 8.73E-03 | down | 2.08 | 1.82E-04 | No |
| *ABI3BP* | BC030221 | ENSG00000154175 | e41 | exon_skipping | down | 5.37 | 1.03E-07 | down | 2.42 | 5.66E-03 | No |
| *ABI3BP* | BC030221 | ENSG00000154175 | e37 | exon_skipping | down | 4.83 | 1.52E-05 | down | 2.42 | 5.66E-03 | No |
| *ABI3BP* | BC030221 | ENSG00000154175 | e45 | exon_skipping | down | 4.19 | 1.60E-05 | down | 2.42 | 5.66E-03 | No |
| *---* | AB209012 | --- | e7 | Unknown | down | 4.12 | 1.62E-12 | down | 2.14 | 9.44E-08 | No |
| *ABI3BP* | BC030221 | ENSG00000154175 | e42 | exon_skipping | down | 4.08 | 4.07E-08 | down | 2.42 | 5.66E-03 | No |
| *ABI3BP* | BC030221 | ENSG00000154175 | e38 | exon_skipping | down | 3.72 | 1.62E-12 | down | 2.42 | 5.66E-03 | No |
| *ABI3BP* | BC030221 | ENSG00000154175 | e24 | exon_skipping | down | 3.39 | 1.62E-12 | down | 2.42 | 5.66E-03 | No |
| *ABI3BP* | BC030221 | ENSG00000154175 | e46 | exon_skipping | down | 3.39 | 2.00E-05 | down | 2.42 | 5.66E-03 | No |
| *ABCA8* | AB020629 | ENSG00000141338 | e3 | exon_skipping | down | 3.32 | 9.60E-05 | down | 4.33 | 9.69E-09 | No |
| *ABCA8* | AB020629 | ENSG00000141338 | e14 | Unknown | down | 3.30 | 1.85E-04 | down | 4.33 | 9.69E-09 | No |
| *ABI3BP* | BC030221 | ENSG00000154175 | e30 | exon_skipping | down | 3.24 | 2.98E-04 | down | 2.42 | 5.66E-03 | No |
| *ABCA8* | AB020629 | ENSG00000141338 | e13 | Unknown | down | 3.13 | 7.17E-04 | down | 4.33 | 9.69E-09 | No |
| *CHGA* | BC006459 | ENSG00000100604 | e3 | Unknown | down | 3.09 | 1.22E-04 | down | 7.65 | 1.63E-12 | No |
| *OTOP2* | BC152986 | ENSG00000183034 | e4 | Unknown | down | 2.95 | 1.86E-05 | down | 19.34 | 4.02E-13 | No |
| *ABI3BP* | BC030221 | ENSG00000154175 | e33 | exon_skipping | down | 2.86 | 1.62E-05 | down | 2.42 | 5.66E-03 | No |
| *OTOP2* | BC152986 | ENSG00000183034 | e3 | Unknown | down | 2.69 | 1.42E-04 | down | 19.34 | 4.02E-13 | No |
| *SLIT2* | AF055585 | ENSG00000145147 | e11 | Unknown | down | 2.63 | 7.84E-03 | down | 2.53 | 1.97E-05 | No |
| *ABI3BP* | BC030221 | ENSG00000154175 | e39 | exon_skipping | down | 2.63 | 1.66E-12 | down | 2.42 | 5.66E-03 | No |
| *ABI3BP* | BC030221 | ENSG00000154175 | e53 | Unknown | down | 2.63 | 1.38E-03 | down | 2.42 | 5.66E-03 | No |
| *ABCA8* | AB020629 | ENSG00000141338 | e18 | Unknown | down | 2.55 | 1.84E-04 | down | 4.33 | 9.69E-09 | No |
| *---* | BC073771 | --- | e110 | exon_skipping,alternative_last_exon | down | 2.53 | 1.12E-07 | down | 1.81 | 1.20E-03 | Yes |
| *ABI3BP* | BC030221 | ENSG00000154175 | e27 | exon_skipping,alternative_first_exon | down | 2.51 | 1.70E-05 | down | 2.42 | 5.66E-03 | No |
| *ABI3BP* | BC030221 | ENSG00000154175 | e52 | exon_skipping | down | 2.45 | 2.92E-03 | down | 2.42 | 5.66E-03 | No |
| *ABCA9* | AY028899 | ENSG00000154258 | e34 | Unknown | down | 2.44 | 8.35E-05 | down | 2.13 | 4.49E-08 | Yes |
| *ABI3BP* | BC030221 | ENSG00000154175 | e28 | exon_skipping | down | 2.44 | 1.60E-04 | down | 2.42 | 5.66E-03 | No |
| *DPT* | BC033736 | ENSG00000143196 | e3 | Unknown | down | 2.42 | 5.65E-04 | down | 5.73 | 1.88E-11 | No |
| *---* | AK096234 | --- | e4 | Unknown | down | 2.41 | 1.64E-03 | down | 2.33 | 1.34E-03 | No |
| *SEMA3D* | AY358937 | ENSG00000153993 | e8 | Unknown | down | 2.39 | 2.76E-04 | down | 3.00 | 3.04E-10 | No |
| *PYY* | BC041057 | ENSG00000131096 | ae6 | intron_retention | down | 2.37 | 1.07E-11 | down | 8.60 | 1.02E-06 | No |
| *ABI3BP* | BC030221 | ENSG00000154175 | e34 | exon_skipping | down | 2.35 | 6.64E-04 | down | 2.42 | 5.66E-03 | No |
| *ABI3BP* | BC030221 | ENSG00000154175 | e14 | exon_skipping | down | 2.33 | 3.58E-03 | down | 2.42 | 5.66E-03 | No |
| *ABI3BP* | BC030221 | ENSG00000154175 | e9 | exon_skipping | down | 2.32 | 1.34E-02 | down | 2.42 | 5.66E-03 | No |
| *PKP1* | BC114571 | ENSG00000081277 | e7 | Unknown | down | 2.31 | 4.32E-02 | down | 1.79 | 6.23E-04 | No |
| *CHRDL1* | BC002909 | ENSG00000101938 | e3 | Unknown | down | 2.30 | 1.72E-05 | down | 7.10 | 1.14E-10 | No |
| *TMIGD1* | AK172838 | ENSG00000182271 | e2 | Unknown | down | 2.29 | 6.34E-05 | down | 17.34 | 1.69E-11 | No |
| *TRPM6* | AF350881 | ENSG00000119121 | e27 | exon_skipping,alternative_last_exon | down | 2.29 | 9.88E-03 | down | 7.04 | 5.75E-07 | No |
| *ABI3BP* | BC030221 | ENSG00000154175 | e43 | exon_skipping,alternative_last_exon | down | 2.29 | 2.00E-03 | down | 2.42 | 5.66E-03 | No |
| *ABI3BP* | BC030221 | ENSG00000154175 | e3 | Unknown | down | 2.28 | 2.20E-03 | down | 2.42 | 5.66E-03 | No |
| *CHGA* | BC006459 | ENSG00000100604 | e2 | Unknown | down | 2.27 | 6.83E-04 | down | 7.65 | 1.63E-12 | No |
| *---* | AK096234 | --- | e5 | alternative_last_exon | down | 2.27 | 2.92E-03 | down | 2.91 | 2.41E-04 | Yes |
| *TRPM6* | AF350881 | ENSG00000119121 | e15 | exon_skipping | down | 2.26 | 3.57E-02 | down | 7.04 | 5.75E-07 | No |
| *BEST4* | AF440757 | ENSG00000142959 | e4 | Unknown | down | 2.25 | 1.61E-05 | down | 11.98 | 6.35E-11 | No |
| *ABI3BP* | BC030221 | ENSG00000154175 | e16 | exon_skipping | down | 2.24 | 1.18E-02 | down | 2.42 | 5.66E-03 | No |
| *ABI3BP* | BC030221 | ENSG00000154175 | ae23 | alternative_acceptor_splice_site | down | 2.24 | 2.38E-03 | down | 2.42 | 5.66E-03 | No |
| *ABI3BP* | BC030221 | ENSG00000154175 | e51 | Unknown | down | 2.22 | 3.14E-03 | down | 2.42 | 5.66E-03 | No |
| *ABCA8* | AB020629 | ENSG00000141338 | e10 | Unknown | down | 2.19 | 4.02E-05 | down | 4.33 | 9.69E-09 | No |
| *ABI3BP* | BC030221 | ENSG00000154175 | e15 | exon_skipping | down | 2.19 | 1.20E-02 | down | 2.42 | 5.66E-03 | No |
| *TRPM6* | AF350881 | ENSG00000119121 | e17 | exon_skipping | down | 2.17 | 3.69E-02 | down | 7.04 | 5.75E-07 | No |
| *---* | BC073771 | --- | e72 | exon_skipping,alternative_first_exon | down | 2.15 | 4.05E-05 | down | 2.08 | 1.82E-04 | No |
| *OGN* | AF086912 | ENSG00000106809 | e4 | Unknown | down | 2.13 | 1.63E-12 | down | 8.52 | 2.01E-13 | No |
| *RERGL* | BC042888 | ENSG00000111404 | e5 | Unknown | down | 2.13 | 6.17E-05 | down | 3.34 | 1.93E-09 | No |
| *ABI3BP* | BC030221 | ENSG00000154175 | e6 | Unknown | down | 2.11 | 5.68E-03 | down | 2.42 | 5.66E-03 | No |
| *CHRDL1* | BC002909 | ENSG00000101938 | e4 | Unknown | down | 2.10 | 2.68E-03 | down | 7.10 | 1.14E-10 | No |
| *ABCA6* | AY028898 | ENSG00000154262 | e35 | Unknown | down | 2.10 | 2.54E-03 | down | 2.68 | 5.42E-07 | No |
| *CADM3* | AF363367 | ENSG00000162706 | e4 | Unknown | down | 2.09 | 1.58E-05 | down | 3.84 | 3.94E-11 | No |
| *PRPH* | AK125587 | ENSG00000135406 | e8 | Unknown | down | 2.07 | 5.62E-05 | down | 2.90 | 2.01E-10 | No |
| *ABI3BP* | BC030221 | ENSG00000154175 | e29 | exon_skipping | down | 2.07 | 2.59E-04 | down | 2.42 | 5.66E-03 | No |
| *FCGR2B* | AK308150 | ENSG00000072694 | e7 | Unknown | down | 2.07 | 6.14E-03 | down | 1.94 | 2.91E-02 | No |
| *---* | BC073771 | --- | e77 | exon_skipping | down | 2.06 | 3.06E-02 | down | 1.81 | 1.20E-03 | Yes |
| *ABCA8* | AB020629 | ENSG00000141338 | e11 | Unknown | down | 2.05 | 6.58E-04 | down | 4.33 | 9.69E-09 | No |
| *ABCA8* | AB020629 | ENSG00000141338 | e20 | Unknown | down | 2.05 | 3.80E-04 | down | 4.33 | 9.69E-09 | No |
| *SLIT3* | BC098388 | ENSG00000184347 | e12 | Unknown | down | 2.04 | 3.14E-03 | down | 3.58 | 3.83E-05 | No |
| *---* | AK096234 | --- | e3 | exon_skipping | down | 2.04 | 1.20E-04 | down | 2.33 | 1.34E-03 | No |
| *ABI3BP* | BC030221 | ENSG00000154175 | e10 | exon_skipping | down | 2.03 | 7.30E-03 | down | 2.42 | 5.66E-03 | No |
| *TRPM6* | AF350881 | ENSG00000119121 | e14 | exon_skipping | down | 2.01 | 9.54E-09 | down | 7.04 | 5.75E-07 | No |
| *PCSK5* | AK225773 | ENSG00000099139 | e21 | Unknown | down | 2.00 | 1.46E-02 | down | 2.74 | 4.26E-03 | No |
| *MAMDC2* | BC045569 | ENSG00000165072 | e7 | Unknown | down | 1.99 | 2.25E-05 | down | 4.07 | 1.07E-07 | No |
| *XIST* | BX648323 | ENSG00000229807 | e5 | Unknown | down | 1.98 | 1.57E-05 | down | 10.46 | 2.20E-07 | No |
| *PMP22* | D11428 | ENSG00000109099 | e1 | alternative_first_exon | down | 1.98 | 3.92E-02 | down | 2.53 | 2.73E-02 | No |
| *ABI3BP* | BC030221 | ENSG00000154175 | e25 | exon_skipping | down | 1.95 | 2.14E-03 | down | 2.42 | 5.66E-03 | No |
| *TMEM72* | AB235418 | ENSG00000187783 | e3 | Unknown | down | 1.94 | 2.13E-02 | down | 2.37 | 2.99E-04 | No |
| *SULT1A2* | U28169 | ENSG00000197165 | e7 | Unknown | down | 1.94 | 5.90E-03 | down | 3.07 | 4.15E-04 | No |
| *UNC5C* | AF055634 | ENSG00000182168 | e11 | Unknown | down | 1.94 | 5.99E-05 | down | 1.92 | 8.74E-03 | No |
| *TRPM6* | AF350881 | ENSG00000119121 | e25 | exon_skipping | down | 1.93 | 3.57E-02 | down | 7.04 | 5.75E-07 | No |
| *DSC3* | D17427 | ENSG00000134762 | e5 | Unknown | down | 1.93 | 3.70E-02 | down | 5.89 | 2.62E-03 | No |
| *DES* | BC032116 | ENSG00000175084 | e3 | Unknown | down | 1.92 | 5.66E-03 | down | 4.21 | 3.03E-12 | No |
| *NAALADL1* | BC143644 | ENSG00000168060 | e10 | Unknown | down | 1.92 | 1.73E-12 | down | 4.22 | 1.93E-09 | No |
| *---* | BC073771 | --- | ae99 | alternative_acceptor_splice_site | down | 1.92 | 6.61E-04 | down | 1.81 | 1.20E-03 | Yes |
| *EPHA7* | L36642 | ENSG00000135333 | e16 | Unknown | down | 1.91 | 2.38E-03 | down | 2.04 | 1.68E-03 | No |
| *CHRDL1* | BC002909 | ENSG00000101938 | e5 | exon_skipping | down | 1.90 | 7.85E-04 | down | 7.10 | 1.14E-10 | No |
| *TRPM6* | AF350881 | ENSG00000119121 | e35 | exon_skipping | down | 1.90 | 5.68E-03 | down | 7.04 | 5.75E-07 | No |
| *FBLN1* | AK075566 | ENSG00000077942 | e20 | Unknown | down | 1.89 | 9.76E-04 | down | 4.44 | 1.88E-03 | No |
| *ABI3BP* | BC030221 | ENSG00000154175 | e50 | Unknown | down | 1.89 | 1.14E-03 | down | 2.42 | 5.66E-03 | No |
| *---* | Y17867 | --- | e3 | Unknown | down | 1.88 | 2.00E-06 | down | 5.22 | 3.90E-07 | Yes |
| *ABI3BP* | BC030221 | ENSG00000154175 | e5 | Unknown | down | 1.88 | 3.58E-03 | down | 2.42 | 5.66E-03 | No |
| *TRPM6* | AF350881 | ENSG00000119121 | e34 | exon_skipping | down | 1.87 | 1.78E-02 | down | 7.04 | 5.75E-07 | No |
| *ABI3BP* | BC030221 | ENSG00000154175 | e19 | exon_skipping | down | 1.87 | 3.27E-02 | down | 2.42 | 5.66E-03 | No |
| *FBLN1* | AK075566 | ENSG00000077942 | e11 | Unknown | down | 1.85 | 1.37E-02 | down | 4.44 | 1.88E-03 | No |
| *ABI3BP* | BC030221 | ENSG00000154175 | e44 | exon_skipping | down | 1.85 | 5.86E-03 | down | 2.42 | 5.66E-03 | No |
| *MAMDC2* | BC045569 | ENSG00000165072 | e4 | Unknown | down | 1.84 | 9.40E-04 | down | 4.07 | 1.07E-07 | No |
| *C4orf7* | AY190326 | ENSG00000181617 | e3 | Unknown | down | 1.84 | 2.12E-03 | down | 2.40 | 2.36E-03 | No |
| *ABI3BP* | BC030221 | ENSG00000154175 | e8 | Unknown | down | 1.84 | 2.08E-03 | down | 2.42 | 5.66E-03 | No |
| *ABI3BP* | BC030221 | ENSG00000154175 | e58 | Unknown | down | 1.84 | 1.41E-04 | down | 2.42 | 5.66E-03 | No |
| *SLIT2* | AF055585 | ENSG00000145147 | e13 | Unknown | down | 1.83 | 1.61E-04 | down | 2.53 | 1.97E-05 | No |
| *ABI3BP* | BC030221 | ENSG00000154175 | e54 | Unknown | down | 1.83 | 5.76E-04 | down | 2.42 | 5.66E-03 | No |
| *ABCA8* | AB020629 | ENSG00000141338 | e15 | exon_skipping | down | 1.82 | 5.84E-05 | down | 4.33 | 9.69E-09 | No |
| *MST1P2 // MST1P9* | AY192149 | ENSG00000186715 | e8 | Unknown | down | 1.82 | 1.06E-02 | down | 2.35 | 6.37E-05 | Yes |
| *ABI3BP* | BC030221 | ENSG00000154175 | e17 | exon_skipping | down | 1.81 | 9.50E-03 | down | 2.42 | 5.66E-03 | No |
| *C7* | BC041807 | ENSG00000112936 | e8 | Unknown | down | 1.80 | 2.14E-03 | down | 4.85 | 7.16E-09 | No |
| *HMCN1* | AF156100 | ENSG00000143341 | e77 | Unknown | down | 1.80 | 1.97E-11 | down | 1.88 | 7.15E-04 | No |
| *ABI3BP* | BC030221 | ENSG00000154175 | e60 | Unknown | down | 1.80 | 1.20E-02 | down | 2.42 | 5.66E-03 | No |
| *ABCA8* | AB020629 | ENSG00000141338 | e17 | Unknown | down | 1.78 | 2.00E-12 | down | 4.33 | 9.69E-09 | No |
| *CP* | AK299272 | ENSG00000047457 | e17 | alternative_last_exon | down | 1.78 | 3.22E-12 | down | 4.12 | 1.74E-06 | Yes |
| *ABI3BP* | BC030221 | ENSG00000154175 | e4 | Unknown | down | 1.77 | 6.77E-04 | down | 2.42 | 5.66E-03 | No |
| *---* | BC148183 | --- | e19 | Unknown | down | 1.77 | 8.16E-03 | down | 1.65 | 5.78E-03 | No |
| *BEST4* | AF440757 | ENSG00000142959 | e5 | Unknown | down | 1.76 | 4.30E-03 | down | 11.98 | 6.35E-11 | No |
| *C7* | BC041807 | ENSG00000112936 | e9 | Unknown | down | 1.75 | 1.63E-04 | down | 4.85 | 7.16E-09 | No |
| *CP* | AK299272 | ENSG00000047457 | e7 | Unknown | down | 1.75 | 9.84E-04 | down | 4.12 | 1.74E-06 | Yes |
| *HMCN1* | AF156100 | ENSG00000143341 | e78 | Unknown | down | 1.75 | 4.02E-03 | down | 1.88 | 7.15E-04 | No |
| *SRPX* | AK125542 | ENSG00000101955 | e4 | exon_skipping | down | 1.75 | 7.41E-04 | down | 3.22 | 2.00E-03 | No |
| *EBF1* | BC038805 | ENSG00000164330 | e7 | Unknown | down | 1.75 | 2.04E-02 | down | 2.65 | 2.64E-03 | No |
| *ABI3BP* | BC030221 | ENSG00000154175 | e2 | Unknown | down | 1.75 | 6.74E-03 | down | 2.42 | 5.66E-03 | No |
| *ABI3BP* | BC030221 | ENSG00000154175 | e7 | Unknown | down | 1.75 | 1.54E-02 | down | 2.42 | 5.66E-03 | No |
| *PDE1A* | AK294239 | ENSG00000115252 | e10 | Unknown | down | 1.75 | 7.26E-03 | down | 1.55 | 1.93E-02 | No |
| *ABCA8* | AB020629 | ENSG00000141338 | e16 | Unknown | down | 1.74 | 7.34E-03 | down | 4.33 | 9.69E-09 | No |
| *---* | BC031092 | --- | e10 | Unknown | down | 1.74 | 1.86E-02 | down | 1.80 | 4.70E-03 | No |
| *ABI3BP* | BC030221 | ENSG00000154175 | e22 | exon_skipping | down | 1.74 | 2.13E-02 | down | 2.42 | 5.66E-03 | No |
| *SLIT2* | AF055585 | ENSG00000145147 | e7 | Unknown | down | 1.73 | 5.98E-03 | down | 2.53 | 1.97E-05 | No |
| *SLIT2* | AF055585 | ENSG00000145147 | e6 | Unknown | down | 1.73 | 3.46E-11 | down | 2.53 | 1.97E-05 | No |
| *PLEKHG5 // TNFRSF25* | BC036671 | ENSG00000171680 // ENSG00000215788 | e31 | Unknown | down | 1.73 | 7.59E-04 | down | 1.77 | 2.37E-02 | No |
| *ATP13A4* | AY823162 | ENSG00000127249 | e25 | Unknown | down | 1.73 | 4.56E-02 | down | 1.65 | 4.89E-02 | No |
| *BMP3* | M22491 | ENSG00000152785 | e3 | alternative_last_exon | down | 1.72 | 2.34E-03 | down | 5.00 | 1.07E-08 | No |
| *---* | BC073771 | --- | e104 | exon_skipping,alternative_first_exon | down | 1.72 | 2.43E-05 | down | 1.81 | 1.20E-03 | Yes |
| *CMAH* | AK303297 | ENSG00000168405 | ae12 | alternative_first_exon | down | 1.72 | 4.03E-02 | down | 3.12 | 5.24E-03 | Yes |
| *ABI3BP* | BC030221 | ENSG00000154175 | e47 | exon_skipping | down | 1.72 | 4.84E-02 | down | 2.42 | 5.66E-03 | No |
| *DES* | BC032116 | ENSG00000175084 | e8 | Unknown | down | 1.71 | 4.48E-03 | down | 4.21 | 3.03E-12 | No |
| *---* | Y17867 | --- | e4 | Unknown | down | 1.71 | 9.24E-03 | down | 5.22 | 3.90E-07 | Yes |
| *SCN9A* | X82835 | ENSG00000169432 | e24 | exon_skipping | down | 1.70 | 4.94E-03 | down | 3.30 | 3.00E-07 | No |
| *SLIT2* | AF055585 | ENSG00000145147 | e10 | Unknown | down | 1.70 | 1.10E-03 | down | 2.53 | 1.97E-05 | No |
| *---* | BC073771 | --- | e5 | alternative_first_exon,exon_skipping | down | 1.70 | 2.80E-03 | down | 1.81 | 1.20E-03 | Yes |
| *ABCA9* | AY028899 | ENSG00000154258 | e35 | Unknown | down | 1.69 | 1.43E-02 | down | 1.94 | 2.26E-07 | No |
| *FBLN2* | AK304777 | ENSG00000163520 | e19 | Unknown | down | 1.69 | 5.59E-04 | down | 4.22 | 3.23E-07 | No |
| *FBLN2* | AK304777 | ENSG00000163520 | e18 | Unknown | down | 1.69 | 1.60E-03 | down | 4.22 | 3.23E-07 | No |
| *CLEC3B* | X64559 | ENSG00000163815 | e3 | Unknown | down | 1.69 | 2.16E-03 | down | 2.75 | 2.04E-05 | No |
| *ITGBL1* | AK291340 | ENSG00000198542 | e11 | Unknown | down | 1.69 | 3.72E-03 | down | 2.04 | 6.31E-05 | No |
| *MYLK* | BC064695 | ENSG00000065534 | e10 | Unknown | down | 1.68 | 3.46E-02 | down | 2.43 | 2.40E-10 | No |
| *DSC3* | D17427 | ENSG00000134762 | e7 | Unknown | down | 1.68 | 2.18E-03 | down | 5.89 | 2.62E-03 | No |
| *ABI3BP* | BC030221 | ENSG00000154175 | e55 | Unknown | down | 1.68 | 1.04E-03 | down | 2.42 | 5.66E-03 | No |
| *ATP13A4* | AY823162 | ENSG00000127249 | e19 | Unknown | down | 1.68 | 4.16E-02 | down | 1.65 | 4.89E-02 | No |
| *NCAM2* | U75330 | ENSG00000154654 | e11 | Unknown | down | 1.67 | 3.00E-04 | down | 1.88 | 2.46E-06 | No |
| *SCGN* | BC003036 | ENSG00000079689 | e4 | Unknown | down | 1.67 | 1.86E-03 | down | 2.32 | 4.48E-06 | No |
| *SLIT2* | AF055585 | ENSG00000145147 | e21 | Unknown | down | 1.67 | 7.44E-03 | down | 2.53 | 1.97E-05 | No |
| *SLIT2* | AF055585 | ENSG00000145147 | e17 | Unknown | down | 1.67 | 1.06E-02 | down | 2.53 | 1.97E-05 | No |
| *SLIT3* | BC098388 | ENSG00000184347 | e15 | Unknown | down | 1.67 | 2.98E-03 | down | 3.58 | 3.83E-05 | No |
| *CFHR1* | BC107771 | ENSG00000244414 | e5 | Unknown | down | 1.67 | 1.70E-02 | down | 2.67 | 2.25E-02 | Yes |
| *NTM* | AY358331 | ENSG00000182667 | e4 | Unknown | down | 1.66 | 1.56E-02 | down | 2.42 | 1.78E-04 | No |
| *IGFBP6* | BC010162 | ENSG00000167779 | e3 | Unknown | down | 1.66 | 1.94E-02 | down | 2.25 | 1.79E-04 | Yes |
| *HSPB6* | CR618274 | ENSG00000004776 | e3 | alternative_last_exon | down | 1.66 | 3.14E-02 | down | 3.02 | 7.84E-04 | No |
| *LGI1* | BC022500 | ENSG00000108231 | e1 | alternative_first_exon | down | 1.65 | 2.39E-04 | down | 2.04 | 1.54E-07 | No |
| *DCLK1* | BC152456 | ENSG00000133083 | e15 | Unknown | down | 1.65 | 4.28E-03 | down | 2.07 | 5.65E-05 | No |
| *MS4A1* | AK225630 | ENSG00000156738 | e7 | Unknown | down | 1.65 | 7.25E-07 | down | 2.65 | 7.86E-03 | No |
| *AGPAT9* | DQ345298 | ENSG00000138678 | e8 | Unknown | down | 1.65 | 2.22E-02 | down | 1.61 | 4.37E-02 | No |
| *DCLK1* | BC152456 | ENSG00000133083 | e12 | exon_skipping | down | 1.64 | 2.16E-03 | down | 2.07 | 5.65E-05 | No |
| *SLC15A1* | U21936 | ENSG00000088386 | e6 | Unknown | down | 1.63 | 3.44E-02 | down | 2.94 | 4.22E-03 | No |
| *ROR1* | M97675 | ENSG00000185483 | e2 | exon_skipping | down | 1.63 | 4.63E-02 | down | 2.49 | 1.17E-02 | No |
| *CADM3* | AF363367 | ENSG00000162706 | e3 | Unknown | down | 1.62 | 5.54E-03 | down | 3.84 | 3.94E-11 | No |
| *ABCA9* | AY028899 | ENSG00000154258 | e26 | Unknown | down | 1.62 | 1.82E-04 | down | 1.94 | 2.26E-07 | No |
| *FAM70A* | AK056403 | ENSG00000125355 | e4 | Unknown | down | 1.62 | 4.70E-03 | down | 2.28 | 3.30E-06 | No |
| *SLIT3* | BC098388 | ENSG00000184347 | e5 | Unknown | down | 1.62 | 5.30E-03 | down | 3.58 | 3.83E-05 | No |
| *RNF152* | AK096495 | ENSG00000176641 | e3 | alternative_last_exon | down | 1.62 | 1.75E-02 | down | 2.95 | 1.18E-04 | No |
| *SLC15A1* | U21936 | ENSG00000088386 | e7 | Unknown | down | 1.62 | 9.81E-04 | down | 2.94 | 4.22E-03 | No |
| *SEMA3D* | AY358937 | ENSG00000153993 | e5 | Unknown | down | 1.61 | 4.57E-04 | down | 3.00 | 3.04E-10 | No |
| *PCOLCE2* | AK075478 | ENSG00000163710 | e5 | Unknown | down | 1.61 | 2.83E-04 | down | 3.16 | 1.89E-09 | No |
| *CALB2* | AK222648 | ENSG00000172137 | e5 | Unknown | down | 1.61 | 1.98E-02 | down | 2.82 | 7.17E-07 | No |
| *XKR4* | AY534241 | ENSG00000206579 | e3 | alternative_last_exon | down | 1.61 | 7.58E-03 | down | 1.86 | 1.74E-05 | No |
| *FAM7A2* | BC070492 | ENSG00000215312 | e6 | exon_skipping | down | 1.61 | 4.14E-02 | down | 1.70 | 9.00E-03 | Yes |
| *SFRP2* | AF311912 | ENSG00000145423 | e2 | Unknown | down | 1.60 | 6.14E-03 | down | 10.91 | 1.67E-08 | No |
| *ANK2* | BX537758 | ENSG00000145362 | e7 | Unknown | down | 1.60 | 4.28E-03 | down | 3.62 | 4.10E-08 | No |
| *LGI1* | BC022500 | ENSG00000108231 | e2 | Unknown | down | 1.60 | 1.76E-12 | down | 2.04 | 1.54E-07 | No |
| *IGF2BP3* | AF117108 | ENSG00000136231 | e18 | Unknown | down | 1.60 | 2.39E-04 | down | 3.67 | 4.27E-07 | No |
| *LIFR* | X61615 | ENSG00000113594 | e13 | Unknown | down | 1.60 | 2.77E-02 | down | 3.27 | 6.11E-05 | No |
| *GPM6B* | AB209525 | ENSG00000046653 | e7 | Unknown | down | 1.60 | 1.25E-02 | down | 2.41 | 1.62E-04 | No |
| *GLI3* | AK308429 | ENSG00000106571 | e9 | Unknown | down | 1.60 | 1.82E-04 | down | 2.96 | 6.39E-04 | No |
| *SLC9A9* | BC035779 | ENSG00000181804 | e2 | exon_skipping | down | 1.60 | 3.62E-03 | down | 1.70 | 3.02E-02 | No |
| *SLIT2* | AF055585 | ENSG00000145147 | e9 | Unknown | down | 1.59 | 3.16E-03 | down | 2.53 | 1.97E-05 | No |
| *HMCN1* | AF156100 | ENSG00000143341 | e31 | Unknown | down | 1.59 | 1.11E-02 | down | 1.88 | 7.15E-04 | No |
| *MYLK* | BC064695 | ENSG00000065534 | e24 | Unknown | down | 1.58 | 1.46E-02 | down | 2.43 | 2.40E-10 | No |
| *ABCG2* | BC092408 | ENSG00000118777 | e17 | Unknown | down | 1.58 | 1.50E-03 | down | 4.44 | 2.38E-08 | No |
| *ST6GAL2* | AB058780 | ENSG00000144057 | e5 | Unknown | down | 1.58 | 4.24E-03 | down | 3.67 | 2.84E-04 | No |
| *FBLN1* | AK075566 | ENSG00000077942 | e13 | Unknown | down | 1.58 | 2.38E-02 | down | 4.49 | 1.62E-03 | Yes |
| *NCAM1* | BC047244 | ENSG00000149294 | e13 | Unknown | down | 1.58 | 1.60E-03 | down | 1.99 | 1.98E-03 | No |
| *LMO3* | AK095595 | ENSG00000048540 | e11 | Unknown | down | 1.58 | 1.19E-09 | down | 2.30 | 2.03E-02 | No |
| *SLC6A19* | AK096054 | ENSG00000174358 | e10 | Unknown | down | 1.57 | 1.18E-02 | down | 7.92 | 1.09E-13 | No |
| *SLC6A19* | AK096054 | ENSG00000174358 | e7 | Unknown | down | 1.57 | 2.65E-02 | down | 7.92 | 1.09E-13 | No |
| *SLIT2* | AF055585 | ENSG00000145147 | e3 | Unknown | down | 1.57 | 1.06E-02 | down | 2.53 | 1.97E-05 | No |
| *EPHA7* | L36642 | ENSG00000135333 | e7 | Unknown | down | 1.57 | 7.44E-03 | down | 2.04 | 1.68E-03 | No |
| *GUCY1A3* | BX649180 | ENSG00000164116 | e12 | alternative_last_exon | down | 1.57 | 1.92E-02 | down | 2.89 | 7.48E-03 | No |
| *ABCA6* | AY028898 | ENSG00000154262 | e5 | Unknown | down | 1.56 | 7.55E-04 | down | 2.68 | 5.42E-07 | No |
| *TRPM6* | AF350881 | ENSG00000119121 | e38 | Unknown | down | 1.56 | 3.45E-02 | down | 7.04 | 5.75E-07 | No |
| *SLIT2* | AF055585 | ENSG00000145147 | e4 | Unknown | down | 1.56 | 1.03E-02 | down | 2.53 | 1.97E-05 | No |
| *CNN1* | BC022015 | ENSG00000130176 | e5 | Unknown | down | 1.56 | 1.44E-02 | down | 2.73 | 5.96E-03 | No |
| *SLC9A9* | BC035779 | ENSG00000181804 | e6 | Unknown | down | 1.56 | 3.60E-03 | down | 1.70 | 3.02E-02 | No |
| *ARHGAP15* | BC029346 | ENSG00000075884 | e10 | Unknown | down | 1.56 | 2.16E-03 | down | 2.15 | 3.79E-02 | No |
| *CXCL13* | AF044197 | ENSG00000156234 | e4 | Unknown | down | 1.56 | 7.44E-03 | down | 2.75 | 4.47E-02 | No |
| *DPT* | BC033736 | ENSG00000143196 | e2 | Unknown | down | 1.55 | 2.10E-02 | down | 5.73 | 1.88E-11 | No |
| *ABCA8* | AB020629 | ENSG00000141338 | e21 | Unknown | down | 1.55 | 5.78E-03 | down | 4.33 | 9.69E-09 | No |
| *CP* | AK299272 | ENSG00000047457 | e4 | Unknown | down | 1.55 | 1.61E-02 | down | 4.12 | 1.74E-06 | Yes |
| *SLIT3* | BC098388 | ENSG00000184347 | e17 | Unknown | down | 1.55 | 9.18E-03 | down | 3.58 | 3.83E-05 | No |
| *FXYD6* | CR617323 | ENSG00000137726 | e7 | exon_skipping | down | 1.55 | 1.04E-02 | down | 2.74 | 6.31E-05 | No |
| *ADAMTSL3* | BC128390 | ENSG00000156218 | e8 | Unknown | down | 1.55 | 1.03E-02 | down | 2.49 | 3.83E-04 | No |
| *EDN3* | BC008876 | ENSG00000124205 | e5 | Unknown | down | 1.55 | 2.46E-03 | down | 3.55 | 1.12E-03 | No |
| *UNC5C* | AF055634 | ENSG00000182168 | e10 | Unknown | down | 1.55 | 5.63E-04 | down | 1.92 | 8.74E-03 | No |
| *---* | BC110411 | --- | ae1 | alternative_donor_splice_site | down | 1.55 | 4.12E-08 | down | 2.01 | 9.86E-03 | No |
| *COLEC12* | AB038518 | ENSG00000158270 | e2 | exon_skipping | down | 1.54 | 4.56E-03 | down | 2.58 | 1.19E-10 | No |
| *COLEC12* | AB038518 | ENSG00000158270 | e5 | Unknown | down | 1.54 | 2.23E-02 | down | 2.58 | 1.19E-10 | No |
| *SLC9A9* | BC035779 | ENSG00000181804 | e1 | alternative_first_exon | down | 1.54 | 9.38E-04 | down | 1.70 | 3.02E-02 | No |
| *CNTN1* | Z21488 | ENSG00000018236 | e25 | Unknown | down | 1.53 | 2.97E-02 | down | 2.36 | 1.50E-07 | No |
| *ABCA6* | AY028898 | ENSG00000154262 | e2 | Unknown | down | 1.53 | 4.81E-06 | down | 2.68 | 5.42E-07 | No |
| *C14orf49* | BC146604 | ENSG00000176438 | e5 | Unknown | down | 1.53 | 1.11E-02 | down | 1.66 | 3.00E-03 | No |
| *AMPD1* | AK097077 | ENSG00000116748 | e5 | Unknown | down | 1.53 | 2.51E-02 | down | 2.92 | 4.16E-03 | No |
| *CNN1* | BC022015 | ENSG00000130176 | e7 | Unknown | down | 1.53 | 7.43E-04 | down | 2.73 | 5.96E-03 | No |
| *SLC9A9* | BC035779 | ENSG00000181804 | e7 | Unknown | down | 1.53 | 4.18E-03 | down | 1.70 | 3.02E-02 | No |
| *SFRP1* | BC036503 | ENSG00000104332 | e3 | Unknown | down | 1.52 | 7.66E-03 | down | 4.45 | 1.91E-09 | Yes |
| *SCN9A* | X82835 | ENSG00000169432 | e2 | Unknown | down | 1.52 | 1.08E-02 | down | 2.74 | 2.67E-06 | Yes |
| *FMN2* | BC112361 | ENSG00000155816 | e3 | exon_skipping | down | 1.52 | 4.39E-02 | down | 2.30 | 4.63E-04 | No |
| *RFX6* | BC039248 | ENSG00000185002 | e13 | Unknown | down | 1.52 | 4.39E-10 | down | 1.88 | 2.44E-03 | No |
| *CNN1* | BC022015 | ENSG00000130176 | e6 | Unknown | down | 1.52 | 1.78E-03 | down | 2.73 | 5.96E-03 | No |
| *TBX10* | AY229977 | ENSG00000167800 | e5 | Unknown | down | 1.52 | 7.62E-04 | down | 2.06 | 4.13E-02 | No |
| *CA7* | AY075019 | ENSG00000168748 | e6 | Unknown | down | 1.51 | 4.92E-03 | down | 15.61 | 1.79E-08 | No |
| *WASF3* | BC050283 | ENSG00000132970 | e3 | Unknown | down | 1.51 | 3.60E-02 | down | 2.09 | 6.20E-03 | No |
| *SYNE1* | AF495910 | ENSG00000131018 | e150 | exon_skipping | down | 1.51 | 4.12E-03 | down | 1.76 | 1.23E-02 | No |
| *PTGIS* | D38145 | ENSG00000124212 | e3 | exon_skipping | down | 1.51 | 3.94E-02 | down | 2.67 | 1.29E-02 | No |
| *SLIT3* | BC098388 | ENSG00000184347 | e8 | Unknown | down | 1.50 | 3.73E-02 | down | 3.55 | 3.97E-05 | Yes |
| *ABCA13* | AY204751 | ENSG00000179869 | e15 | Unknown | up | 4.42 | 3.04E-04 | down | 1.34 | 3.62E-03 | No |
| *C6orf130 // UNC5CL* | AK310594 | ENSG00000124596 // ENSG00000124602 | e13 | Unknown | up | 3.84 | 3.14E-03 | up | 1.06 | 7.69E-01 | No |
| *AQP1 // FAM188B // INMT* | AK308762 | ENSG00000106125 // ENSG00000240583 // ENSG00000241644 | e11 | alternative_last_exon | up | 3.62 | 1.59E-02 | down | 2.07 | 1.26E-01 | No |
| *C1S // EMG1* | AK055183 | ENSG00000126749 // ENSG00000182326 | e2 | Unknown | up | 3.58 | 2.36E-02 | down | 2.22 | 7.81E-02 | No |
| *AQP1 // FAM188B // INMT* | AK308762 | ENSG00000106125 // ENSG00000240583 // ENSG00000241644 | e18 | Unknown | up | 3.57 | 3.09E-02 | down | 2.07 | 1.26E-01 | No |
| *C1S // EMG1* | AK055183 | ENSG00000126749 // ENSG00000182326 | e3 | exon_skipping | up | 3.49 | 2.17E-02 | down | 2.22 | 7.81E-02 | No |
| *C1S // EMG1* | AK055183 | ENSG00000126749 // ENSG00000182326 | e5 | alternative_last_exon,exon_skipping | up | 3.45 | 1.98E-02 | down | 2.22 | 7.81E-02 | No |
| *AQP1 // FAM188B // INMT* | AK308762 | ENSG00000106125 // ENSG00000240583 // ENSG00000241644 | e14 | Unknown | up | 3.41 | 3.79E-02 | down | 2.07 | 1.26E-01 | No |
| *C1S // EMG1* | AK055183 | ENSG00000126749 // ENSG00000182326 | e6 | exon_skipping | up | 3.26 | 1.85E-02 | down | 2.22 | 7.81E-02 | No |
| *FAS // STAMBPL1* | AB037794 | ENSG00000026103 // ENSG00000138134 | e8 | Unknown | up | 3.19 | 2.17E-04 | down | 1.36 | 1.43E-01 | No |
| *FAS // STAMBPL1* | AB037794 | ENSG00000026103 // ENSG00000138134 | e9 | Unknown | up | 3.09 | 3.42E-04 | down | 1.36 | 1.43E-01 | No |
| *FAS // STAMBPL1* | AB037794 | ENSG00000026103 // ENSG00000138134 | e4 | Unknown | up | 3.09 | 3.21E-04 | down | 1.36 | 1.43E-01 | No |
| *AQP1 // FAM188B // INMT* | AK308762 | ENSG00000106125 // ENSG00000240583 // ENSG00000241644 | e13 | Unknown | up | 3.06 | 3.11E-08 | down | 2.07 | 1.26E-01 | No |
| *CYP2B7P1* | BC041174 | --- | e3 | Unknown | up | 3.06 | 6.46E-03 | down | 1.23 | 6.00E-01 | No |
| *AQP1 // FAM188B // INMT* | AK308762 | ENSG00000106125 // ENSG00000240583 // ENSG00000241644 | e22 | Unknown | up | 3.02 | 4.39E-02 | down | 2.07 | 1.26E-01 | No |
| *AQP1 // FAM188B // INMT* | AK308762 | ENSG00000106125 // ENSG00000240583 // ENSG00000241644 | e9 | Unknown | up | 3.01 | 4.14E-02 | down | 2.07 | 1.26E-01 | No |
| *OAS1* | AJ629455 | ENSG00000089127 | ae5 | alternative_last_exon | up | 2.99 | 1.41E-02 | up | 1.00 | 9.99E-01 | No |
| *FAS // STAMBPL1* | AB037794 | ENSG00000026103 // ENSG00000138134 | e11 | exon_skipping,alternative_last_exon | up | 2.97 | 1.42E-03 | down | 1.36 | 1.43E-01 | No |
| *FDFT1* | AK057726 | ENSG00000079459 | e1 | alternative_first_exon | up | 2.90 | 1.56E-02 | up | 1.17 | 3.65E-01 | Yes |
| *FAS // STAMBPL1* | AB037794 | ENSG00000026103 // ENSG00000138134 | e6 | alternative_first_exon | up | 2.81 | 5.37E-04 | down | 1.36 | 1.43E-01 | No |
| *ABCA13* | AY204751 | ENSG00000179869 | e4 | Unknown | up | 2.80 | 7.12E-03 | down | 1.34 | 3.62E-03 | No |
| *C1S // EMG1* | AK055183 | ENSG00000126749 // ENSG00000182326 | e4 | exon_skipping | up | 2.80 | 4.92E-02 | down | 2.22 | 7.81E-02 | No |
| *FAS // STAMBPL1* | AB037794 | ENSG00000026103 // ENSG00000138134 | e7 | Unknown | up | 2.78 | 1.16E-03 | down | 1.36 | 1.43E-01 | No |
| *HKDC1* | AK095086 | ENSG00000156510 | e3 | Unknown | up | 2.77 | 6.57E-11 | up | 1.56 | 1.04E-01 | No |
| *AXIN2 // CCDC46* | AK299075 | ENSG00000154240 // ENSG00000168646 | e35 | exon_skipping | up | 2.76 | 3.30E-02 | up | 1.30 | 2.24E-01 | No |
| *RPL27* | AK124148 | ENSG00000131469 | ae1 | intron_retention,alternative_first_exon | up | 2.74 | 4.46E-03 | up | 1.08 | 8.05E-01 | No |
| *KRT6B* | BC034535 | ENSG00000185479 | e8 | Unknown | up | 2.73 | 2.16E-03 | up | 1.78 | 2.04E-01 | Yes |
| *AXIN2 // CCDC46* | AK299075 | ENSG00000154240 // ENSG00000168646 | e34 | Unknown | up | 2.69 | 1.24E-02 | up | 1.30 | 2.24E-01 | No |
| *AXIN2 // CCDC46* | AK299075 | ENSG00000154240 // ENSG00000168646 | e43 | exon_skipping,alternative_last_exon | up | 2.67 | 2.50E-02 | up | 1.30 | 2.24E-01 | No |
| *FAS // STAMBPL1* | AB037794 | ENSG00000026103 // ENSG00000138134 | e2 | alternative_first_exon | up | 2.66 | 3.24E-03 | down | 1.36 | 1.43E-01 | No |
| *AXIN2 // CCDC46* | AK299075 | ENSG00000154240 // ENSG00000168646 | ae43 | intron_retention | up | 2.60 | 3.52E-02 | up | 1.30 | 2.24E-01 | No |
| *COL1A1* | Z74615 | --- | e19 | Unknown | up | 2.60 | 2.45E-02 | down | 1.30 | 4.76E-01 | No |
| *ABCA13* | AY204751 | ENSG00000179869 | e38 | Unknown | up | 2.59 | 2.63E-04 | down | 1.34 | 3.62E-03 | No |
| *UGT1A1 // UGT1A3 // UGT1A4 // UGT1A7 // UGT1A8 // UGT1A9 // UGT1A10* | AF462268 | ENSG00000241119 // ENSG00000241635 // ENSG00000242366 // ENSG00000242515 // ENSG00000243135 // ENSG00000244122 // ENSG00000244474 | e6 | exon_skipping,alternative_first_exon | up | 2.52 | 2.86E-02 | up | 1.01 | 9.72E-01 | No |
| *AXIN2 // CCDC46* | AK299075 | ENSG00000154240 // ENSG00000168646 | e36 | exon_skipping | up | 2.49 | 3.36E-02 | up | 1.30 | 2.24E-01 | No |
| *FAS // STAMBPL1* | AB037794 | ENSG00000026103 // ENSG00000138134 | e3 | Unknown | up | 2.49 | 1.64E-03 | down | 1.36 | 1.43E-01 | No |
| *---* | AK098764 | --- | ae1 | alternative_donor_splice_site | up | 2.45 | 4.29E-02 | up | 1.10 | 6.27E-01 | Yes |
| *KRT6A* | BC014152 | ENSG00000205420 | e8 | Unknown | up | 2.42 | 1.04E-03 | up | 1.92 | 1.53E-01 | Yes |
| *KRT6B* | BC034535 | ENSG00000185479 | e4 | Unknown | up | 2.42 | 3.50E-03 | up | 1.78 | 2.04E-01 | Yes |
| *KRT6A* | BC014152 | ENSG00000205420 | e4 | Unknown | up | 2.41 | 3.28E-03 | up | 1.92 | 1.53E-01 | Yes |
| *AXIN2 // CCDC46* | AK299075 | ENSG00000154240 // ENSG00000168646 | e38 | exon_skipping | up | 2.35 | 1.49E-02 | up | 1.30 | 2.24E-01 | No |
| *AXIN2 // CCDC46* | AK299075 | ENSG00000154240 // ENSG00000168646 | e42 | exon_skipping | up | 2.35 | 9.92E-03 | up | 1.30 | 2.24E-01 | No |
| *FAS // STAMBPL1* | AB037794 | ENSG00000026103 // ENSG00000138134 | e5 | Unknown | up | 2.31 | 2.76E-03 | down | 1.36 | 1.43E-01 | Yes |
| *KRI1* | AL833189 | ENSG00000129347 | e2 | Unknown | up | 2.30 | 1.56E-03 | up | 1.00 | 9.94E-01 | Yes |
| *AXIN2 // CCDC46* | AK299075 | ENSG00000154240 // ENSG00000168646 | e41 | exon_skipping | up | 2.29 | 3.26E-02 | up | 1.30 | 2.24E-01 | No |
| *AXIN2 // CCDC46* | AK299075 | ENSG00000154240 // ENSG00000168646 | e33 | alternative_first_exon,exon_skipping | up | 2.28 | 2.96E-03 | up | 1.30 | 2.24E-01 | No |
| *HNRNPUL1* | AK225850 | ENSG00000105323 | ae4 | alternative_donor_splice_site | up | 2.28 | 8.24E-04 | up | 1.05 | 5.72E-01 | No |
| *CHST5* | BC063677 | ENSG00000135702 | e6 | exon_skipping | up | 2.27 | 1.35E-04 | down | 1.49 | 1.64E-01 | No |
| *UGT1A1 // UGT1A3 // UGT1A4 // UGT1A7 // UGT1A8 // UGT1A9 // UGT1A10* | AF462268 | ENSG00000241119 // ENSG00000241635 // ENSG00000242366 // ENSG00000242515 // ENSG00000243135 // ENSG00000244122 // ENSG00000244474 | ae6 | alternative_first_exon | up | 2.24 | 1.76E-02 | up | 1.01 | 9.72E-01 | No |
| *HKDC1* | AK095086 | ENSG00000156510 | e2 | Unknown | up | 2.21 | 4.33E-02 | up | 1.56 | 1.04E-01 | No |
| *DGAT1* | BC023565 | ENSG00000185000 | e10 | exon_skipping | up | 2.21 | 1.40E-04 | up | 1.06 | 6.62E-01 | No |
| *ABCA13* | AY204751 | ENSG00000179869 | e29 | Unknown | up | 2.20 | 5.44E-03 | down | 1.34 | 3.62E-03 | No |
| *---* | AF480461 | --- | e20 | Unknown | up | 2.18 | 4.26E-05 | up | 1.15 | 6.58E-01 | No |
| *ABCA13* | AY204751 | ENSG00000179869 | e22 | Unknown | up | 2.17 | 3.05E-09 | down | 1.34 | 3.62E-03 | Yes |
| *FAS // STAMBPL1* | AB037794 | ENSG00000026103 // ENSG00000138134 | e10 | Unknown | up | 2.15 | 6.04E-03 | down | 1.36 | 1.43E-01 | No |
| *CYP2B7P1* | BC041174 | --- | e9 | alternative_last_exon | up | 2.13 | 1.45E-02 | down | 1.23 | 6.00E-01 | No |
| *ZBTB38* | BC015444 | ENSG00000177311 | e3 | Unknown | up | 2.12 | 2.21E-04 | up | 1.21 | 1.59E-04 | No |
| *PRKCD* | BC043350 | ENSG00000163932 | ae12 | alternative_donor_splice_site | up | 2.12 | 2.12E-02 | down | 1.37 | 9.59E-02 | No |
| *FAM13A* | AX747234 | ENSG00000138640 | e6 | exon_skipping,alternative_last_exon | up | 2.10 | 3.55E-02 | up | 1.05 | 8.71E-01 | No |
| *CCNDBP1 // TMEM62* | BC009981 | ENSG00000137842 // ENSG00000166946 | e12 | exon_skipping | up | 2.08 | 1.72E-02 | down | 1.24 | 2.79E-01 | No |
| *RDX* | DQ916742 | ENSG00000137710 | e11 | exon_skipping | up | 2.06 | 2.05E-02 | down | 1.86 | 1.04E-01 | No |
| *HKDC1* | AK095086 | ENSG00000156510 | e1 | alternative_first_exon | up | 2.05 | 1.63E-02 | up | 1.56 | 1.04E-01 | No |
| *ARID3B* | BC045545 | ENSG00000179361 | ae6 | intron_retention | up | 2.05 | 1.64E-03 | down | 1.15 | 1.67E-01 | Yes |
| *ABTB1* | BC011858 | ENSG00000114626 | ae7 | intron_retention | up | 2.05 | 1.88E-02 | down | 1.15 | 3.95E-01 | No |
| *C6orf130 // UNC5CL* | AK310594 | ENSG00000124596 // ENSG00000124602 | e10 | alternative_last_exon | up | 2.04 | 3.33E-02 | up | 1.06 | 7.69E-01 | No |
| *ABCA13* | AY204751 | ENSG00000179869 | e30 | Unknown | up | 2.03 | 1.12E-02 | down | 1.34 | 3.62E-03 | Yes |
| *PLXNA1* | AB385178 | ENSG00000114554 | e4 | Unknown | up | 2.02 | 5.76E-05 | up | 1.27 | 1.49E-01 | No |
| *CAMK2G* | AK094774 | ENSG00000148660 | e16 | exon_skipping | up | 2.02 | 9.72E-03 | down | 1.20 | 1.22E-01 | No |
| *OSBPL1A* | AF392450 | ENSG00000141447 | ae23 | alternative_last_exon | up | 2.01 | 4.04E-02 | down | 1.81 | 5.76E-02 | Yes |
| *DISC1 // TSNAX* | FJ804217 | ENSG00000116918 // ENSG00000162946 | e3 | exon_skipping | up | 2.00 | 2.87E-02 | down | 1.62 | 5.30E-02 | Yes |
| *ARL2 // SNX15* | AK096447 | ENSG00000110025 // ENSG00000213465 | e5 | alternative_last_exon,exon_skipping | up | 2.00 | 3.83E-10 | down | 1.26 | 2.15E-01 | No |
| *ARHGEF10L* | AB046846 | ENSG00000074964 | e1 | alternative_first_exon | up | 2.00 | 2.00E-03 | down | 1.03 | 7.72E-01 | No |
| *MNT* | X96401 | ENSG00000070444 | ae6 | intron_retention | up | 1.98 | 1.30E-03 | down | 1.17 | 1.18E-01 | No |
| *ARL2 // SNX15* | AK096447 | ENSG00000110025 // ENSG00000213465 | e4 | exon_skipping | up | 1.98 | 1.28E-02 | down | 1.26 | 2.15E-01 | No |
| *RPL9* | CR605134 | ENSG00000163682 | ae1 | intron_retention | up | 1.97 | 2.88E-03 | down | 1.03 | 8.72E-01 | Yes |
| *DISC1 // TSNAX* | FJ804217 | ENSG00000116918 // ENSG00000162946 | e2 | Unknown | up | 1.96 | 1.62E-02 | down | 1.62 | 5.30E-02 | Yes |
| *ARL2 // SNX15* | AK096447 | ENSG00000110025 // ENSG00000213465 | e2 | Unknown | up | 1.95 | 2.66E-03 | down | 1.26 | 2.15E-01 | No |
| *FAM129A* | AF288391 | ENSG00000135842 | e15 | Unknown | up | 1.94 | 6.76E-03 | down | 2.67 | 5.24E-02 | No |
| *AXIN2 // CCDC46* | AK299075 | ENSG00000154240 // ENSG00000168646 | e37 | exon_skipping,alternative_last_exon | up | 1.93 | 1.89E-02 | up | 1.30 | 2.24E-01 | No |
| *HTR4 // SH3TC2* | AB070620 | ENSG00000164270 // ENSG00000169247 | e9 | Unknown | up | 1.93 | 1.02E-04 | down | 1.26 | 5.65E-01 | No |
| *SCN7A* | M91556 | ENSG00000136546 | e12 | Unknown | up | 1.92 | 1.27E-06 | down | 2.73 | 1.42E-01 | No |
| *SH3RF3* | AK074131 | ENSG00000172985 | e4 | Unknown | up | 1.92 | 4.68E-03 | down | 1.28 | 2.76E-01 | Yes |
| *RPS7* | BC061901 | ENSG00000171863 | e1 | alternative_first_exon | up | 1.91 | 6.01E-04 | up | 1.37 | 3.19E-06 | No |
| *ZBTB38* | BC015444 | ENSG00000177311 | e4 | Unknown | up | 1.90 | 2.92E-03 | up | 1.21 | 1.59E-04 | No |
| *ZBTB38* | BC015444 | ENSG00000177311 | e2 | alternative_first_exon | up | 1.90 | 3.62E-03 | up | 1.21 | 1.59E-04 | No |
| *DISC1 // TSNAX* | FJ804217 | ENSG00000116918 // ENSG00000162946 | e4 | exon_skipping | up | 1.90 | 2.02E-02 | down | 1.62 | 5.30E-02 | Yes |
| *OSBPL1A* | AF392450 | ENSG00000141447 | e8 | Unknown | up | 1.90 | 1.38E-02 | down | 1.81 | 5.76E-02 | Yes |
| *GET4 // SUN1* | AK022816 | ENSG00000164828 // ENSG00000239857 | e28 | alternative_first_exon | up | 1.90 | 3.52E-05 | down | 1.18 | 3.12E-01 | No |
| *---* | AJ294733 | --- | e2 | Unknown | up | 1.89 | 1.40E-03 | down | 1.21 | 7.95E-01 | Yes |
| *DISC1 // TSNAX* | FJ804217 | ENSG00000116918 // ENSG00000162946 | e5 | exon_skipping | up | 1.88 | 3.86E-02 | down | 1.62 | 5.30E-02 | Yes |
| *COL6A2* | BC065509 | ENSG00000142173 | e14 | exon_skipping | up | 1.88 | 6.36E-03 | down | 1.77 | 1.39E-01 | Yes |
| *OSBPL1A* | AF392450 | ENSG00000141447 | e12 | Unknown | up | 1.87 | 3.18E-02 | down | 1.81 | 5.76E-02 | No |
| *C15orf21* | AK096745 | ENSG00000179362 | e9 | alternative_last_exon | up | 1.87 | 1.69E-02 | down | 1.91 | 6.30E-02 | Yes |
| *C15orf21* | AK096745 | ENSG00000179362 | e5 | exon_skipping,alternative_last_exon | up | 1.87 | 1.29E-02 | down | 1.91 | 6.30E-02 | Yes |
| *LTBP4* | AF051345 | ENSG00000090006 | e14 | exon_skipping | up | 1.87 | 3.09E-02 | down | 1.63 | 1.20E-01 | No |
| *RPS6KA4* | AK223561 | ENSG00000162302 | e2 | Unknown | up | 1.86 | 4.97E-04 | up | 1.17 | 1.39E-01 | No |
| *ZNF266* | BC112919 | ENSG00000174652 | e8 | exon_skipping | up | 1.86 | 1.60E-03 | up | 1.05 | 8.54E-01 | No |
| *N4BP2* | AB371584 | ENSG00000078177 | e12 | Unknown | up | 1.86 | 5.39E-04 | down | 1.02 | 9.04E-01 | No |
| *FGFRL1* | BC036769 | ENSG00000127418 | e9 | Unknown | up | 1.85 | 4.98E-03 | up | 1.51 | 5.25E-02 | No |
| *MLXIPL* | AF156603 | ENSG00000009950 | e4 | Unknown | up | 1.85 | 2.52E-03 | up | 1.65 | 1.58E-01 | No |
| *FAIM2* | AB023167 | ENSG00000135472 | e12 | Unknown | up | 1.85 | 5.76E-03 | up | 1.67 | 1.79E-01 | No |
| *PTPRC* | Y00062 | ENSG00000081237 | e17 | Unknown | up | 1.85 | 5.68E-03 | down | 1.87 | 1.80E-01 | Yes |
| *CYP4F2* | U02388 | ENSG00000186115 | e9 | Unknown | up | 1.85 | 2.44E-02 | down | 1.40 | 3.84E-01 | Yes |
| *APIP* | AK302225 | ENSG00000149089 | e7 | Unknown | up | 1.84 | 5.56E-03 | up | 1.37 | 1.12E-01 | No |
| *CCNDBP1 // TMEM62* | BC009981 | ENSG00000137842 // ENSG00000166946 | e11 | Unknown | up | 1.84 | 9.24E-03 | down | 1.24 | 2.79E-01 | No |
| *SCMH1* | BX640721 | ENSG00000010803 | e6 | exon_skipping | up | 1.83 | 5.24E-03 | up | 1.22 | 1.83E-01 | No |
| *VCAN* | X15998 | ENSG00000038427 | e7 | exon_skipping | up | 1.83 | 4.96E-02 | down | 2.29 | 7.09E-02 | Yes |
| *C2orf64 // MGAT4A* | AK314024 | ENSG00000071073 // ENSG00000183513 | e21 | exon_skipping | up | 1.83 | 2.66E-03 | down | 1.56 | 7.43E-02 | No |
| *ARHGEF9* | AL832116 | ENSG00000131089 | e3 | exon_skipping | up | 1.82 | 1.76E-02 | down | 1.47 | 1.41E-01 | No |
| *WDR44* | AK127556 | ENSG00000131725 | e2 | exon_skipping | up | 1.82 | 2.54E-02 | down | 1.07 | 7.50E-01 | No |
| *HKDC1* | AK095086 | ENSG00000156510 | e8 | Unknown | up | 1.81 | 2.20E-04 | up | 1.56 | 1.04E-01 | No |
| *ARL2 // SNX15* | AK096447 | ENSG00000110025 // ENSG00000213465 | e3 | alternative_last_exon | up | 1.81 | 1.40E-02 | down | 1.26 | 2.15E-01 | No |
| *SDHAP1* | BC071730 | ENSG00000185485 | e16 | alternative_last_exon | up | 1.80 | 3.84E-02 | down | 1.61 | 6.84E-02 | No |
| *C2orf64 // MGAT4A* | AK314024 | ENSG00000071073 // ENSG00000183513 | e20 | alternative_first_exon,exon_skipping | up | 1.80 | 1.81E-02 | down | 1.56 | 7.43E-02 | No |
| *CCNDBP1 // TMEM62* | BC009981 | ENSG00000137842 // ENSG00000166946 | e10 | alternative_first_exon | up | 1.80 | 3.90E-02 | down | 1.27 | 2.17E-01 | Yes |
| *TNC* | X78565 | ENSG00000041982 | e13 | exon_skipping | up | 1.80 | 2.57E-04 | down | 1.06 | 9.21E-01 | No |
| *HYI* | AF284751 | ENSG00000178922 | ae2 | alternative_first_exon | up | 1.79 | 1.10E-03 | down | 1.07 | 8.01E-01 | No |
| *OSBPL1A* | AF392450 | ENSG00000141447 | e10 | Unknown | up | 1.78 | 2.01E-02 | down | 1.81 | 5.76E-02 | Yes |
| *RBM47* | BC071585 | ENSG00000163694 | e2 | alternative_first_exon,exon_skipping | up | 1.78 | 2.83E-02 | down | 1.21 | 1.27E-01 | No |
| *ZBTB38* | BC015444 | ENSG00000177311 | e5 | Unknown | up | 1.77 | 1.09E-02 | up | 1.21 | 1.59E-04 | No |
| *OSBPL1A* | AF392450 | ENSG00000141447 | e14 | Unknown | up | 1.76 | 2.78E-02 | down | 1.81 | 5.76E-02 | Yes |
| *---* | BC071595 | --- | e22 | exon_skipping | up | 1.76 | 2.48E-02 | down | 1.22 | 1.12E-01 | No |
| *---* | AK001299 | --- | e3 | exon_skipping | up | 1.76 | 2.41E-02 | down | 1.63 | 2.20E-01 | Yes |
| *PGAM5 // PXMP2* | AK301831 | ENSG00000176894 | e12 | Unknown | up | 1.75 | 2.66E-03 | down | 1.12 | 4.91E-02 | No |
| *DISC1 // TSNAX* | FJ804217 | ENSG00000116918 // ENSG00000162946 | e6 | exon_skipping,alternative_last_exon | up | 1.75 | 2.31E-02 | down | 1.62 | 5.30E-02 | Yes |
| *NHEJ1 // SLC23A3* | AK125121 | ENSG00000187736 // ENSG00000213901 | e15 | Unknown | up | 1.75 | 6.02E-03 | down | 1.36 | 2.20E-01 | No |
| *AKAP2 // PALM2 // PALM2-AKAP2* | AJ303079 | ENSG00000157654 // ENSG00000241978 // ENSG00000243444 | e7 | exon_skipping | up | 1.74 | 8.32E-03 | down | 1.50 | 2.74E-01 | Yes |
| *CCNDBP1 // TMEM62* | BC009981 | ENSG00000137842 // ENSG00000166946 | e13 | Unknown | up | 1.74 | 2.95E-02 | down | 1.24 | 2.79E-01 | No |
| *ARHGAP32* | EF127492 | ENSG00000134909 | e16 | Unknown | up | 1.74 | 2.28E-03 | down | 1.31 | 2.86E-01 | No |
| *SHISA5* | AK056328 | ENSG00000164054 | e4 | Unknown | up | 1.73 | 8.14E-10 | up | 1.12 | 2.46E-01 | No |
| *NEBL* | AB209311 | ENSG00000078114 | e14 | exon_skipping | up | 1.73 | 1.66E-02 | up | 1.04 | 8.27E-01 | No |
| *---* | BC016359 | --- | e3 | Unknown | up | 1.73 | 5.78E-04 | up | 1.01 | 9.50E-01 | No |
| *ARL2 // SNX15* | AK096447 | ENSG00000110025 // ENSG00000213465 | e1 | alternative_first_exon | up | 1.73 | 1.43E-09 | down | 1.26 | 2.15E-01 | No |
| *MAP7D3* | AL832120 | ENSG00000129680 | e4 | Unknown | up | 1.73 | 3.54E-02 | down | 1.65 | 2.38E-01 | Yes |
| *DOCK5 // PPP2R2A* | AK130123 | ENSG00000147459 // ENSG00000221914 | e59 | exon_skipping | up | 1.73 | 3.37E-02 | down | 1.17 | 3.84E-01 | No |
| *HAVCR1* | AK298259 | ENSG00000113249 | e2 | alternative_first_exon | up | 1.73 | 5.69E-05 | down | 1.04 | 9.63E-01 | No |
| *B4GALT2* | BC096821 | ENSG00000117411 | e6 | Unknown | up | 1.72 | 2.85E-02 | up | 1.18 | 2.16E-01 | No |
| *TOP2A* | BC140791 | ENSG00000131747 | e18 | Unknown | up | 1.72 | 4.32E-03 | up | 1.18 | 5.99E-01 | No |
| *LPP* | AK304035 | ENSG00000145012 | e5 | exon_skipping | up | 1.72 | 2.36E-04 | down | 1.38 | 4.47E-02 | No |
| *CAPN13* | BX640793 | ENSG00000162949 | e2 | exon_skipping | up | 1.72 | 4.52E-03 | down | 2.19 | 5.92E-02 | No |
| *RTTN* | BC143723 | ENSG00000176225 | e43 | Unknown | up | 1.72 | 2.17E-04 | down | 1.06 | 6.75E-01 | No |
| *SLC38A9* | AK122957 | ENSG00000177058 | e7 | Unknown | up | 1.72 | 1.08E-03 | down | 1.07 | 7.18E-01 | Yes |
| *MLXIPL* | AF156603 | ENSG00000009950 | e6 | alternative_first_exon | up | 1.71 | 3.66E-02 | up | 1.65 | 1.58E-01 | No |
| *MUC3A* | AB038784 | ENSG00000169894 | e12 | alternative_last_exon | up | 1.71 | 2.24E-02 | down | 1.39 | 2.10E-02 | No |
| *SIAE* | AF300796 | ENSG00000110013 | e3 | Unknown | up | 1.71 | 2.22E-02 | down | 1.42 | 1.51E-01 | No |
| *TNC* | X78565 | ENSG00000041982 | e16 | exon_skipping | up | 1.71 | 3.54E-03 | down | 1.06 | 9.21E-01 | No |
| *PTK7* | U33635 | ENSG00000112655 | e8 | alternative_last_exon | up | 1.70 | 1.30E-02 | up | 1.45 | 2.43E-01 | No |
| *DHX37* | BC037964 | ENSG00000150990 | e15 | Unknown | up | 1.70 | 3.33E-02 | up | 1.11 | 4.21E-01 | No |
| *KLK1* | AY429508 | ENSG00000167748 | e5 | Unknown | up | 1.70 | 5.34E-03 | down | 1.48 | 3.49E-01 | No |
| *EIF3E* | CR615385 | ENSG00000104408 | e6 | alternative_last_exon | up | 1.69 | 1.04E-02 | up | 1.33 | 3.24E-01 | No |
| *PGAM5 // PXMP2* | AK301831 | ENSG00000176894 | e8 | Unknown | up | 1.69 | 3.56E-03 | down | 1.12 | 4.91E-02 | No |
| *LSP1* | CR595099 | ENSG00000130592 | e1 | alternative_first_exon | up | 1.69 | 3.76E-05 | down | 1.95 | 9.46E-02 | Yes |
| *MYH9* | AB191263 | ENSG00000100345 | ae43 | intron_retention | up | 1.69 | 1.88E-03 | down | 1.09 | 3.52E-01 | Yes |
| *---* | BC026300 | --- | e2 | exon_skipping | up | 1.69 | 1.87E-02 | down | 1.09 | 7.58E-01 | Yes |
| *C14orf149* | AK058165 | ENSG00000126790 | ae1 | alternative_donor_splice_site | up | 1.69 | 8.40E-03 | down | 1.06 | 8.18E-01 | No |
| *GCHFR* | AK223430 | ENSG00000137880 | e2 | Unknown | up | 1.69 | 7.01E-04 | down | 1.00 | 9.97E-01 | No |
| *ZBTB38* | BC015444 | ENSG00000177311 | e7 | exon_skipping | up | 1.68 | 3.98E-02 | up | 1.21 | 1.59E-04 | No |
| *CSNK1G1 // KIAA0101* | AK301858 | ENSG00000166803 // ENSG00000169118 | e5 | exon_skipping,alternative_last_exon | up | 1.68 | 3.07E-02 | up | 1.01 | 9.63E-01 | No |
| *PGAM5 // PXMP2* | AK301831 | ENSG00000176894 | e9 | Unknown | up | 1.68 | 1.10E-02 | down | 1.12 | 4.91E-02 | No |
| *ZNF320* | AK307375 | ENSG00000182986 | ae10 | alternative_last_exon | up | 1.68 | 1.88E-02 | down | 1.24 | 3.46E-01 | Yes |
| *HOOK3* | BC056146 | ENSG00000168172 | e17 | Unknown | up | 1.68 | 2.18E-02 | down | 1.25 | 4.48E-01 | No |
| *TRIM29* | L24203 | ENSG00000137699 | e5 | Unknown | up | 1.67 | 1.70E-03 | up | 2.11 | 1.56E-01 | No |
| *PRPF8* | BC064370 | ENSG00000174231 | e26 | exon_skipping | up | 1.67 | 1.90E-02 | up | 1.06 | 7.24E-01 | No |
| *MNT* | X96401 | ENSG00000070444 | e7 | alternative_last_exon | up | 1.67 | 3.30E-03 | down | 1.17 | 1.18E-01 | No |
| *FOXP2* | AK131266 | ENSG00000128573 | e10 | Unknown | up | 1.67 | 3.88E-02 | down | 2.50 | 1.47E-01 | No |
| *TFEB* | AJ608786 | ENSG00000112561 | e9 | exon_skipping | up | 1.67 | 2.32E-02 | down | 1.20 | 2.12E-01 | Yes |
| *SIPA1* | AF029789 | ENSG00000213445 | e12 | Unknown | up | 1.67 | 1.99E-02 | down | 1.26 | 3.01E-01 | Yes |
| *PMS2CL* | BC041364 | ENSG00000187953 | e13 | Unknown | up | 1.67 | 6.50E-03 | down | 1.11 | 6.01E-01 | Yes |
| *PRPF31* | AY040822 | ENSG00000105618 | e13 | exon_skipping | up | 1.66 | 1.44E-02 | up | 1.03 | 9.01E-01 | No |
| *PGAM5 // PXMP2* | AK301831 | ENSG00000176894 | e10 | Unknown | up | 1.66 | 2.10E-02 | down | 1.12 | 4.91E-02 | No |
| *NHEJ1 // SLC23A3* | AK125121 | ENSG00000187736 // ENSG00000213901 | e16 | Unknown | up | 1.66 | 4.13E-02 | down | 1.36 | 2.20E-01 | No |
| *CCNDBP1 // TMEM62* | BC009981 | ENSG00000137842 // ENSG00000166946 | e8 | Unknown | up | 1.66 | 1.60E-02 | down | 1.24 | 2.79E-01 | No |
| *CHKB // CPT1B* | AB051457 | ENSG00000100288 // ENSG00000205560 | e24 | Unknown | up | 1.66 | 4.82E-03 | down | 1.04 | 6.52E-01 | No |
| *TNPO2* | AK056183 | ENSG00000105576 | ae1 | intron_retention | up | 1.65 | 3.63E-02 | up | 1.42 | 1.10E-02 | No |
| *ZNF232* | AK316369 | ENSG00000167840 | e5 | Unknown | up | 1.65 | 4.44E-02 | up | 1.26 | 2.19E-01 | No |
| *LIN9* | BC065302 | ENSG00000183814 | e8 | Unknown | up | 1.65 | 5.21E-04 | up | 1.18 | 3.77E-01 | No |
| *CYB561* | AK095244 | ENSG00000008283 | e7 | Unknown | up | 1.65 | 4.60E-04 | up | 1.03 | 9.01E-01 | No |
| *---* | AB014514 | --- | e24 | Unknown | up | 1.65 | 2.37E-02 | down | 1.16 | 3.48E-01 | No |
| *SVIL* | AF051850 | ENSG00000197321 | e2 | Unknown | up | 1.65 | 6.52E-03 | down | 1.12 | 4.24E-01 | No |
| *ALDH3A1* | BC004102 | ENSG00000108602 | e10 | Unknown | up | 1.64 | 2.74E-03 | up | 1.90 | 6.71E-02 | No |
| *DNMT3A* | AB208833 | ENSG00000119772 | e25 | Unknown | up | 1.64 | 2.97E-04 | up | 1.24 | 2.79E-01 | No |
| *ADORA3* | BC064411 | ENSG00000121933 | e8 | alternative_last_exon | up | 1.64 | 4.36E-06 | down | 1.63 | 1.48E-01 | Yes |
| *RAB40B* | CR598763 | ENSG00000141542 | e5 | Unknown | up | 1.64 | 3.09E-06 | down | 1.32 | 2.96E-01 | No |
| *PPP3CA* | EU192653 | ENSG00000138814 | e9 | exon_skipping | up | 1.64 | 1.48E-03 | down | 1.08 | 7.37E-01 | No |
| *MYBBP1A* | AK303150 | ENSG00000132382 | e5 | Unknown | up | 1.63 | 2.76E-03 | up | 1.31 | 1.50E-01 | No |
| *MYBBP1A* | AK303150 | ENSG00000132382 | e24 | exon_skipping | up | 1.63 | 6.62E-03 | up | 1.31 | 1.50E-01 | No |
| *DNMT3A* | AB208833 | ENSG00000119772 | e26 | Unknown | up | 1.63 | 8.16E-03 | up | 1.24 | 2.79E-01 | No |
| *ASAP2* | AB007860 | ENSG00000151693 | e18 | Unknown | up | 1.63 | 2.59E-02 | down | 1.21 | 1.69E-01 | Yes |
| *PRDM15* | AB051815 | ENSG00000141956 | e9 | Unknown | up | 1.63 | 1.32E-02 | down | 1.12 | 1.79E-01 | No |
| *FAM21C* | AB011164 | ENSG00000172661 | e24 | Unknown | up | 1.63 | 3.61E-02 | down | 1.15 | 3.44E-01 | No |
| *SLC8A1* | BX537482 | ENSG00000183023 | e14 | exon_skipping | up | 1.63 | 8.06E-03 | down | 1.30 | 3.73E-01 | No |
| *C19orf54* | AK123126 | ENSG00000188493 | ae8 | intron_retention,alternative_acceptor_splice_site | up | 1.63 | 2.69E-02 | down | 1.08 | 6.59E-01 | No |
| *TNPO2* | AK056183 | ENSG00000105576 | e2 | Unknown | up | 1.62 | 5.00E-03 | up | 1.42 | 1.10E-02 | No |
| *CUL7* | BC033647 | ENSG00000044090 | e3 | exon_skipping | up | 1.62 | 5.68E-03 | up | 1.34 | 2.32E-02 | No |
| *CADPS* | AB032947 | ENSG00000163618 | e29 | Unknown | up | 1.62 | 1.55E-02 | up | 1.86 | 1.45E-01 | No |
| *---* | AK309777 | --- | e11 | exon_skipping | up | 1.62 | 4.34E-02 | up | 1.17 | 2.93E-01 | Yes |
| *FHIT* | BC057223 | ENSG00000189283 | e4 | Unknown | up | 1.62 | 2.77E-02 | up | 1.17 | 4.78E-01 | No |
| *DNM2 // QTRT1* | AB209213 | ENSG00000079805 // ENSG00000213339 | e5 | Unknown | up | 1.62 | 7.41E-04 | up | 1.05 | 6.17E-01 | No |
| *INF2* | BX248757 | ENSG00000203485 | e4 | Unknown | up | 1.62 | 3.16E-03 | up | 1.06 | 6.39E-01 | No |
| *NBPF3* | AK095602 | ENSG00000142794 | e12 | Unknown | up | 1.62 | 1.42E-02 | up | 1.04 | 8.24E-01 | No |
| *ARID5B* | BC156570 | ENSG00000150347 | e4 | Unknown | up | 1.62 | 3.61E-11 | up | 1.02 | 9.38E-01 | No |
| *OSBPL1A* | AF392450 | ENSG00000141447 | e18 | Unknown | up | 1.62 | 3.65E-02 | down | 1.81 | 5.76E-02 | No |
| *CACNA2D1* | AK299838 | ENSG00000153956 | e6 | Unknown | up | 1.62 | 3.49E-02 | down | 1.74 | 6.49E-02 | Yes |
| *ZNF430* | AY269787 | ENSG00000118620 | e3 | Unknown | up | 1.62 | 1.56E-03 | down | 1.26 | 1.68E-01 | Yes |
| *UBR2* | AY061884 | ENSG00000024048 | e17 | exon_skipping | up | 1.62 | 2.21E-04 | down | 1.27 | 2.31E-01 | No |
| *ABCA1* | AK022254 | ENSG00000165029 | e19 | exon_skipping | up | 1.62 | 8.80E-03 | down | 1.20 | 5.19E-01 | No |
| *PMS2CL* | BC041364 | ENSG00000187953 | e11 | Unknown | up | 1.62 | 6.20E-04 | down | 1.11 | 6.01E-01 | Yes |
| *PROM1* | AK027422 | ENSG00000007062 | e5 | Unknown | up | 1.62 | 4.15E-02 | down | 1.04 | 9.47E-01 | No |
| *BBS1 // DPP3* | AL833475 | ENSG00000174483 | e17 | Unknown | up | 1.61 | 6.78E-03 | up | 1.33 | 1.84E-02 | No |
| *THNSL2* | CR620866 | ENSG00000144115 | e7 | exon_skipping | up | 1.61 | 3.50E-02 | up | 2.07 | 9.32E-02 | No |
| *CUEDC2* | BC000262 | ENSG00000107874 | e7 | Unknown | up | 1.61 | 5.17E-04 | up | 1.22 | 1.06E-01 | No |
| *TCTN2* | DQ278870 | ENSG00000168778 | e7 | Unknown | up | 1.61 | 2.14E-03 | up | 1.15 | 4.94E-01 | No |
| *IFI30 // PIK3R2* | BC063240 | ENSG00000105647 // ENSG00000216490 | e13 | Unknown | up | 1.61 | 2.58E-04 | down | 1.30 | 8.84E-03 | No |
| *PGAM5 // PXMP2* | AK301831 | ENSG00000176894 | e11 | Unknown | up | 1.61 | 4.82E-03 | down | 1.12 | 4.91E-02 | No |
| *CDK11A // CDK11B // GNB1* | BC171773 | ENSG00000008128 // ENSG00000078369 | e5 | exon_skipping | up | 1.61 | 3.29E-02 | down | 1.37 | 1.09E-01 | Yes |
| *SCN7A* | M91556 | ENSG00000136546 | e24 | Unknown | up | 1.61 | 2.30E-03 | down | 2.73 | 1.42E-01 | No |
| *CCNDBP1 // TMEM62* | BC009981 | ENSG00000137842 // ENSG00000166946 | e9 | Unknown | up | 1.61 | 4.04E-02 | down | 1.27 | 2.17E-01 | Yes |
| *CAST* | AB208816 | ENSG00000153113 | e3 | exon_skipping,alternative_first_exon | up | 1.61 | 1.80E-02 | down | 1.09 | 5.28E-01 | No |
| *PMS2CL* | BC041364 | ENSG00000187953 | e14 | Unknown | up | 1.61 | 2.38E-03 | down | 1.11 | 6.01E-01 | Yes |
| *ZNF790* | BC057245 | ENSG00000197863 | e2 | Unknown | up | 1.61 | 4.65E-02 | down | 1.10 | 6.62E-01 | No |
| *FGGY* | BC014947 | ENSG00000172456 | e16 | Unknown | up | 1.60 | 1.16E-04 | up | 2.26 | 5.03E-02 | No |
| *SHQ1* | AK001401 | ENSG00000144736 | e5 | Unknown | up | 1.60 | 1.60E-03 | up | 1.07 | 7.32E-01 | No |
| *ARHGEF2* | AM393305 | ENSG00000116584 | e27 | exon_skipping | up | 1.60 | 3.18E-03 | up | 1.03 | 8.98E-01 | No |
| *TAF1C* | AK304261 | ENSG00000103168 | e7 | exon_skipping | up | 1.60 | 1.96E-03 | down | 1.10 | 3.83E-04 | No |
| *ZCCHC10* | AK000101 | ENSG00000155329 | e2 | exon_skipping | up | 1.60 | 1.39E-02 | down | 1.32 | 1.43E-01 | No |
| *PTPRC* | Y00062 | ENSG00000081237 | e25 | exon_skipping | up | 1.60 | 3.37E-02 | down | 1.87 | 1.80E-01 | No |
| *C11orf74* | AK095997 | ENSG00000166352 | e5 | Unknown | up | 1.60 | 5.36E-04 | down | 1.29 | 2.44E-01 | No |
| *CCNDBP1 // TMEM62* | BC009981 | ENSG00000137842 // ENSG00000166946 | e7 | Unknown | up | 1.60 | 1.08E-08 | down | 1.24 | 2.79E-01 | No |
| *ABCB6 // ATG9A* | AB209054 | ENSG00000115657 // ENSG00000198925 | e29 | Unknown | up | 1.59 | 1.63E-12 | up | 1.40 | 3.78E-03 | No |
| *CUL7* | BC033647 | ENSG00000044090 | e8 | exon_skipping | up | 1.59 | 1.90E-03 | up | 1.34 | 2.32E-02 | No |
| *CACNA2D2* | AF042793 | ENSG00000007402 | e16 | Unknown | up | 1.59 | 3.88E-03 | up | 1.69 | 1.21E-01 | No |
| *SLC25A6* | CR616158 | ENSG00000169100 | e2 | exon_skipping | up | 1.59 | 4.93E-02 | up | 1.22 | 1.35E-01 | Yes |
| *PLXNA1* | AB385178 | ENSG00000114554 | e7 | Unknown | up | 1.59 | 4.68E-03 | up | 1.27 | 1.49E-01 | No |
| *FTSJD2* | D43949 | ENSG00000137200 | e15 | Unknown | up | 1.59 | 7.30E-03 | up | 1.15 | 3.47E-01 | No |
| *USP38* | AL833976 | ENSG00000170185 | e5 | Unknown | up | 1.59 | 3.49E-02 | up | 1.16 | 3.89E-01 | No |
| *ZNF185* | AY997296 | ENSG00000147394 | e22 | Unknown | up | 1.59 | 1.64E-03 | up | 1.24 | 5.07E-01 | No |
| *CYP2B7P1* | BC041174 | --- | e5 | alternative_last_exon | up | 1.59 | 5.56E-05 | up | 1.32 | 5.86E-01 | Yes |
| *NISCH* | AB023192 | ENSG00000010322 | ae16 | alternative_first_exon | up | 1.59 | 1.38E-02 | down | 1.24 | 3.18E-02 | No |
| *MYO5A* | Y07759 | ENSG00000197535 | e17 | Unknown | up | 1.59 | 1.77E-02 | down | 1.88 | 5.01E-02 | No |
| *MAP1S* | AK292555 | ENSG00000130479 | e3 | exon_skipping | up | 1.59 | 4.17E-04 | down | 1.13 | 1.69E-01 | No |
| *GGT6* | AK074646 | ENSG00000167741 | e1 | alternative_first_exon | up | 1.59 | 2.19E-04 | down | 1.11 | 2.50E-01 | No |
| *FURIN* | X17094 | ENSG00000140564 | e14 | Unknown | up | 1.59 | 8.95E-04 | down | 1.16 | 4.02E-01 | No |
| *NOL11* | AK299483 | ENSG00000130935 | e17 | Unknown | up | 1.58 | 3.26E-03 | up | 1.19 | 4.00E-01 | No |
| *ANKRD26* | AB028997 | ENSG00000107890 | e2 | Unknown | up | 1.58 | 6.58E-03 | up | 1.02 | 9.02E-01 | No |
| *MAST2* | AB047005 | ENSG00000086015 | e5 | exon_skipping | up | 1.58 | 1.94E-02 | down | 1.35 | 1.41E-08 | No |
| *RNFT2* | BC011878 | ENSG00000135119 | e6 | Unknown | up | 1.58 | 1.47E-02 | down | 1.22 | 9.95E-04 | No |
| *IFIH1* | AK292941 | ENSG00000115267 | e14 | Unknown | up | 1.58 | 6.12E-03 | down | 1.57 | 5.04E-02 | Yes |
| *LUM* | CR591759 | ENSG00000139329 | e1 | alternative_first_exon | up | 1.58 | 3.71E-02 | down | 2.69 | 9.26E-02 | No |
| *RBM47* | BC071585 | ENSG00000163694 | e3 | exon_skipping,alternative_last_exon | up | 1.58 | 9.98E-03 | down | 1.21 | 1.27E-01 | No |
| *BBX* | AF454941 | ENSG00000114439 | e11 | Unknown | up | 1.58 | 3.54E-03 | down | 1.46 | 1.81E-01 | No |
| *ZNF235* | BC002800 | ENSG00000159917 | e6 | exon_skipping,alternative_last_exon | up | 1.58 | 3.24E-02 | down | 1.13 | 4.56E-01 | Yes |
| *ATF6B* | U52696 | ENSG00000213676 | e14 | Unknown | up | 1.58 | 2.44E-03 | down | 1.03 | 8.09E-01 | Yes |
| *DUS2L* | AK000406 | ENSG00000167264 | e8 | Unknown | up | 1.57 | 3.12E-02 | up | 1.28 | 3.34E-02 | No |
| *SEC16B* | AB067515 | ENSG00000120341 | e33 | exon_skipping | up | 1.57 | 4.60E-03 | up | 1.78 | 7.69E-02 | No |
| *FGFR4* | BC011847 | ENSG00000160867 | e12 | Unknown | up | 1.57 | 1.16E-03 | up | 1.63 | 8.54E-02 | No |
| *C6orf94 // LTV1* | AK311477 | ENSG00000118491 // ENSG00000135521 | e10 | exon_skipping | up | 1.57 | 1.16E-03 | up | 1.62 | 1.30E-01 | No |
| *LYPLA1* | AF081281 | ENSG00000120992 | e6 | alternative_last_exon | up | 1.57 | 4.18E-02 | up | 1.15 | 3.67E-01 | No |
| *AUTS2* | BC064693 | ENSG00000158321 | e11 | Unknown | up | 1.57 | 1.39E-02 | up | 1.12 | 5.91E-01 | Yes |
| *ACVR2A* | M93415 | ENSG00000121989 | ae2 | alternative_first_exon | up | 1.57 | 1.74E-03 | down | 1.41 | 8.98E-02 | Yes |
| *PRDM15* | AB051815 | ENSG00000141956 | e15 | Unknown | up | 1.57 | 4.41E-02 | down | 1.12 | 1.79E-01 | No |
| *ARHGAP32* | EF127492 | ENSG00000134909 | e4 | Unknown | up | 1.57 | 2.55E-02 | down | 1.31 | 2.86E-01 | No |
| *ASNS* | BC030024 | ENSG00000070669 | e4 | exon_skipping,alternative_last_exon | up | 1.57 | 1.46E-03 | down | 1.35 | 5.16E-01 | No |
| *---* | BC073170 | --- | e3 | alternative_last_exon | up | 1.57 | 3.23E-02 | down | 1.07 | 7.20E-01 | Yes |
| *SRF* | BC052572 | ENSG00000112658 | e7 | Unknown | up | 1.57 | 3.87E-02 | down | 1.03 | 8.15E-01 | No |
| *SEMA3A* | AK289954 | ENSG00000075213 | e13 | Unknown | up | 1.57 | 6.18E-03 | down | 1.01 | 9.59E-01 | No |
| *CUL7* | BC033647 | ENSG00000044090 | e13 | exon_skipping | up | 1.56 | 1.53E-02 | up | 1.34 | 2.32E-02 | No |
| *FAM40A* | AK125054 | ENSG00000143093 | e21 | Unknown | up | 1.56 | 5.40E-03 | up | 1.33 | 1.24E-01 | No |
| *FAIM2* | AB023167 | ENSG00000135472 | e11 | Unknown | up | 1.56 | 6.27E-12 | up | 1.67 | 1.79E-01 | No |
| *DIAPH3* | AY750055 | ENSG00000139734 | e20 | Unknown | up | 1.56 | 4.86E-03 | up | 1.38 | 2.14E-01 | No |
| *UBA2* | AK023120 | ENSG00000126261 | e8 | Unknown | up | 1.56 | 2.56E-04 | up | 1.18 | 4.10E-01 | No |
| *DHX37* | BC037964 | ENSG00000150990 | e3 | Unknown | up | 1.56 | 4.58E-04 | up | 1.11 | 4.21E-01 | No |
| *UBA7* | L13852 | ENSG00000182179 | e7 | Unknown | up | 1.56 | 1.11E-07 | up | 1.07 | 5.35E-01 | No |
| *PLEKHM1P* | BC041324 | ENSG00000159266 | e25 | Unknown | up | 1.56 | 1.53E-02 | up | 1.04 | 8.40E-01 | No |
| *DNAH1* | AB290163 | ENSG00000114841 | e48 | Unknown | up | 1.56 | 3.87E-02 | down | 1.33 | 1.12E-01 | No |
| *IFT172* | BC144491 | ENSG00000138002 | e47 | Unknown | up | 1.56 | 3.60E-02 | down | 1.10 | 4.62E-01 | Yes |
| *ACBD6* | CR626583 | ENSG00000135847 | e7 | exon_skipping | up | 1.56 | 1.34E-03 | down | 1.06 | 7.33E-01 | No |
| *C11orf9* | AB023171 | ENSG00000124920 | e6 | Unknown | up | 1.55 | 1.70E-02 | up | 1.20 | 4.78E-03 | No |
| *EXOSC1* | AF151866 | ENSG00000171311 | e1 | alternative_first_exon | up | 1.55 | 5.44E-03 | up | 1.36 | 5.68E-02 | No |
| *ZC3HAV1L* | BC020784 | ENSG00000146858 | e5 | exon_skipping,alternative_last_exon | up | 1.55 | 1.30E-02 | up | 1.36 | 2.48E-01 | No |
| *TMEM150A* | AK074505 | ENSG00000168890 | e3 | Unknown | up | 1.55 | 2.87E-02 | up | 1.08 | 5.04E-01 | No |
| *FDPS* | CR590457 | ENSG00000160752 | e8 | Unknown | up | 1.55 | 2.22E-02 | up | 1.10 | 5.53E-01 | Yes |
| *EIF2S2* | CR624977 | ENSG00000125977 | e1 | alternative_first_exon | up | 1.55 | 6.98E-03 | up | 1.11 | 6.22E-01 | No |
| *---* | AF480461 | --- | e15 | Unknown | up | 1.55 | 2.77E-02 | up | 1.15 | 6.58E-01 | No |
| *---* | AK124332 | --- | e7 | Unknown | up | 1.55 | 4.64E-04 | up | 1.08 | 7.47E-01 | No |
| *CCNYL1* | BC067253 | ENSG00000163249 | e8 | exon_skipping,alternative_first_exon | up | 1.55 | 1.52E-03 | down | 1.45 | 3.93E-02 | Yes |
| *IQGAP2* | U51903 | ENSG00000145703 | e14 | Unknown | up | 1.55 | 2.13E-02 | down | 1.73 | 5.67E-02 | Yes |
| *SYTL2* | AB046817 | ENSG00000137501 | e17 | exon_skipping | up | 1.55 | 2.02E-05 | down | 1.66 | 6.50E-02 | Yes |
| *PALMD* | AJ312214 | ENSG00000099260 | e2 | Unknown | up | 1.55 | 8.90E-03 | down | 1.89 | 8.88E-02 | No |
| *ZNF442* | AK024418 | ENSG00000198342 | e4 | exon_skipping | up | 1.55 | 1.59E-02 | down | 1.32 | 1.09E-01 | No |
| *IL6ST* | M57230 | ENSG00000134352 | e2 | Unknown | up | 1.55 | 2.72E-03 | down | 1.67 | 1.21E-01 | Yes |
| *KLK1* | AY429508 | ENSG00000167748 | e6 | alternative_last_exon | up | 1.55 | 1.07E-02 | down | 1.48 | 3.49E-01 | No |
| *MAP2K6* | U39657 | ENSG00000108984 | e6 | Unknown | up | 1.55 | 4.08E-02 | down | 1.19 | 5.56E-01 | No |
| *CCDC93* | AB209993 | ENSG00000125633 | e23 | Unknown | up | 1.55 | 2.00E-02 | down | 1.11 | 5.59E-01 | No |
| *PLEKHM1P* | BC041324 | ENSG00000159266 | e29 | Unknown | up | 1.55 | 1.64E-02 | down | 1.07 | 6.82E-01 | Yes |
| *NEK1* | AL050385 | ENSG00000137601 | e9 | Unknown | up | 1.55 | 4.20E-02 | down | 1.05 | 7.74E-01 | Yes |
| *---* | AF465484 | --- | e39 | Unknown | up | 1.54 | 3.06E-03 | up | 1.93 | 7.85E-02 | No |
| *CACNA2D2* | AF042793 | ENSG00000007402 | e21 | Unknown | up | 1.54 | 3.89E-02 | up | 1.69 | 1.21E-01 | No |
| *CACNA2D2* | AF042793 | ENSG00000007402 | e23 | Unknown | up | 1.54 | 2.54E-03 | up | 1.69 | 1.21E-01 | No |
| *BLM* | BC034480 | ENSG00000197299 | e7 | Unknown | up | 1.54 | 2.07E-02 | up | 1.30 | 1.59E-01 | No |
| *ADAMTS9* | BC171764 | ENSG00000163638 | e7 | Unknown | up | 1.54 | 1.10E-02 | up | 1.64 | 1.77E-01 | No |
| *DHX57* | BC153875 | ENSG00000163214 | e18 | Unknown | up | 1.54 | 5.20E-03 | up | 1.27 | 2.28E-01 | No |
| *---* | AK126662 | --- | e3 | Unknown | up | 1.54 | 2.00E-03 | up | 1.25 | 2.37E-01 | No |
| *SMC2* | AL833191 | ENSG00000136824 | e7 | Unknown | up | 1.54 | 4.20E-02 | up | 1.24 | 3.74E-01 | No |
| *POMZP3* | U10099 | ENSG00000146707 | e5 | Unknown | up | 1.54 | 2.50E-02 | up | 1.14 | 5.75E-01 | Yes |
| *PLA2G4A* | M68874 | ENSG00000116711 | e11 | Unknown | up | 1.54 | 7.42E-04 | up | 1.10 | 7.97E-01 | No |
| *THOP1* | BC002391 | ENSG00000172009 | e5 | Unknown | up | 1.54 | 3.22E-02 | up | 1.01 | 9.40E-01 | No |
| *MRAS* | AK124954 | ENSG00000158186 | e4 | exon_skipping,alternative_first_exon | up | 1.54 | 2.44E-02 | down | 1.56 | 1.33E-01 | Yes |
| *CD4* | BC025782 | ENSG00000010610 | e3 | exon_skipping | up | 1.54 | 4.78E-02 | down | 1.72 | 1.57E-01 | Yes |
| *CCNDBP1 // TMEM62* | BC009981 | ENSG00000137842 // ENSG00000166946 | e6 | Unknown | up | 1.54 | 4.51E-02 | down | 1.27 | 2.17E-01 | Yes |
| *ARHGAP32* | EF127492 | ENSG00000134909 | e7 | Unknown | up | 1.54 | 4.94E-02 | down | 1.31 | 2.86E-01 | No |
| *CPB1* | CR623808 | ENSG00000153002 | e8 | Unknown | up | 1.54 | 2.73E-02 | down | 2.09 | 3.44E-01 | Yes |
| *ASS1* | CR605897 | ENSG00000130707 | e3 | exon_skipping | up | 1.54 | 8.58E-03 | down | 1.20 | 3.95E-01 | No |
| *ZNF638* | AK303723 | ENSG00000075292 | e7 | Unknown | up | 1.54 | 1.44E-02 | down | 1.13 | 6.51E-01 | No |
| *POLR3B* | BX537447 | ENSG00000013503 | e13 | Unknown | up | 1.54 | 1.50E-02 | down | 1.02 | 8.80E-01 | No |
| *GLMN* | AK295971 | ENSG00000174842 | e17 | Unknown | up | 1.53 | 1.10E-02 | up | 1.41 | 1.67E-01 | No |
| *DIAPH1* | DQ067453 | ENSG00000131504 | e20 | Unknown | up | 1.53 | 1.51E-02 | up | 1.18 | 1.99E-01 | No |
| *KCTD18* | BC067755 | ENSG00000155729 | e4 | Unknown | up | 1.53 | 7.08E-03 | up | 1.25 | 2.36E-01 | No |
| *C12orf48* | AK000648 | ENSG00000185480 | e13 | exon_skipping | up | 1.53 | 1.57E-02 | up | 1.31 | 3.52E-01 | No |
| *NR4A2* | AK223625 | ENSG00000153234 | e2 | exon_skipping | up | 1.53 | 4.95E-02 | up | 1.34 | 5.00E-01 | No |
| *SPAG9* | AY850123 | ENSG00000008294 | e7 | exon_skipping,alternative_last_exon | up | 1.53 | 8.06E-03 | up | 1.00 | 9.97E-01 | No |
| *C2orf64 // MGAT4A* | AK314024 | ENSG00000071073 // ENSG00000183513 | e22 | alternative_last_exon | up | 1.53 | 2.97E-02 | down | 1.56 | 7.43E-02 | Yes |
| *CDK11A // CDK11B // GNB1* | BC171773 | ENSG00000008128 // ENSG00000078369 | e17 | exon_skipping | up | 1.53 | 3.28E-02 | down | 1.37 | 1.09E-01 | Yes |
| *ARL3* | BC009841 | ENSG00000138175 | e6 | alternative_last_exon | up | 1.53 | 3.38E-02 | down | 1.25 | 1.84E-01 | Yes |
| *CCNDBP1 // TMEM62* | BC009981 | ENSG00000137842 // ENSG00000166946 | e5 | Unknown | up | 1.53 | 4.40E-02 | down | 1.27 | 2.17E-01 | Yes |
| *CCNDBP1 // TMEM62* | BC009981 | ENSG00000137842 // ENSG00000166946 | e2 | Unknown | up | 1.53 | 4.63E-02 | down | 1.27 | 2.17E-01 | Yes |
| *GET4 // SUN1* | AK022816 | ENSG00000164828 // ENSG00000239857 | e30 | Unknown | up | 1.53 | 3.16E-03 | down | 1.18 | 3.12E-01 | No |
| *DOCK5 // PPP2R2A* | AK130123 | ENSG00000147459 // ENSG00000221914 | e63 | Unknown | up | 1.53 | 1.41E-06 | down | 1.17 | 3.84E-01 | No |
| *TOR1AIP2* | BC094724 | ENSG00000169905 | e6 | exon_skipping | up | 1.53 | 1.70E-02 | down | 1.17 | 4.23E-01 | Yes |
| *HIP1* | BC110545 | ENSG00000127946 | e23 | Unknown | up | 1.53 | 3.08E-06 | down | 1.19 | 5.62E-01 | Yes |
| *ZNF468* | CR936695 | ENSG00000204604 | e4 | exon_skipping | up | 1.53 | 1.40E-02 | down | 1.06 | 7.57E-01 | No |
| *APITD1 // CORT* | AF521016 | ENSG00000175279 // ENSG00000241563 | e2 | exon_skipping,alternative_first_exon | up | 1.53 | 2.36E-07 | down | 1.02 | 8.76E-01 | Yes |
| *CHMP4A // IPO4 // MDP1 // TM9SF1* | AK300118 | ENSG00000100926 // ENSG00000100931 // ENSG00000196497 // ENSG00000213920 | e31 | Unknown | up | 1.52 | 1.65E-04 | up | 1.48 | 7.70E-03 | No |
| *RRP12* | AK022496 | ENSG00000052749 | e5 | exon_skipping | up | 1.52 | 4.52E-02 | up | 1.34 | 7.35E-02 | No |
| *PNPLA6* | BC050553 | ENSG00000032444 | e28 | exon_skipping | up | 1.52 | 2.92E-03 | up | 1.21 | 1.81E-01 | No |
| *---* | X66893 | --- | e9 | Unknown | up | 1.52 | 5.82E-03 | up | 1.21 | 3.10E-01 | No |
| *LRP11* | BC043141 | ENSG00000120256 | e8 | alternative_last_exon | up | 1.52 | 2.25E-02 | up | 1.16 | 3.12E-01 | No |
| *GRHL1* | AF198489 | ENSG00000134317 | e3 | Unknown | up | 1.52 | 1.55E-02 | up | 1.23 | 3.75E-01 | No |
| *C18orf22* | AK307212 | ENSG00000101546 | e5 | exon_skipping,alternative_first_exon | up | 1.52 | 2.20E-03 | up | 1.09 | 3.75E-01 | No |
| *DHX37* | BC037964 | ENSG00000150990 | e18 | Unknown | up | 1.52 | 2.20E-02 | up | 1.11 | 4.21E-01 | No |
| *ERCC8* | AK056931 | ENSG00000049167 | e15 | Unknown | up | 1.52 | 2.83E-04 | up | 1.16 | 5.01E-01 | No |
| *XRRA1* | AK056364 | ENSG00000166435 | e8 | Unknown | up | 1.52 | 4.14E-03 | up | 1.17 | 5.29E-01 | No |
| *---* | AF480461 | --- | e19 | Unknown | up | 1.52 | 9.90E-03 | up | 1.15 | 6.58E-01 | No |
| *HSD3B7* | EU176408 | ENSG00000099377 | ae7 | alternative_last_exon | up | 1.52 | 4.36E-02 | up | 1.03 | 8.50E-01 | No |
| *C11orf17* | AF493785 | ENSG00000166452 | e3 | exon_skipping | up | 1.52 | 3.23E-02 | up | 1.03 | 8.83E-01 | No |
| *RNFT2* | BC011878 | ENSG00000135119 | e12 | exon_skipping | up | 1.52 | 3.10E-03 | down | 1.22 | 9.95E-04 | No |
| *GTF3C1* | CR627430 | ENSG00000077235 | e6 | Unknown | up | 1.52 | 2.53E-02 | down | 1.24 | 9.95E-02 | Yes |
| *CDK11A // CDK11B // GNB1* | BC171773 | ENSG00000008128 // ENSG00000078369 | ae19 | alternative_first_exon | up | 1.52 | 3.86E-02 | down | 1.37 | 1.09E-01 | Yes |
| *PRKCB* | X06318 | ENSG00000166501 | e8 | Unknown | up | 1.52 | 8.04E-04 | down | 1.61 | 2.28E-01 | Yes |
| *SIPA1* | AF029789 | ENSG00000213445 | e15 | Unknown | up | 1.52 | 7.72E-03 | down | 1.26 | 3.04E-01 | No |
| *HP1BP3* | AK023129 | ENSG00000127483 | ae2 | alternative_donor_splice_site | up | 1.52 | 4.61E-02 | down | 1.09 | 5.94E-01 | No |
| *SSBP4* | BC000274 | ENSG00000130511 | e2 | Unknown | up | 1.52 | 1.62E-02 | down | 1.08 | 6.06E-01 | No |
| *---* | BC033072 | --- | e3 | Unknown | up | 1.52 | 3.72E-03 | down | 1.08 | 6.42E-01 | Yes |
| *PARP1* | AK225654 | ENSG00000143799 | e8 | Unknown | up | 1.52 | 2.17E-02 | down | 1.01 | 9.61E-01 | No |
| *---* | AF465484 | --- | e48 | Unknown | up | 1.51 | 1.07E-08 | up | 1.93 | 7.85E-02 | No |
| *HKDC1* | AK095086 | ENSG00000156510 | e9 | Unknown | up | 1.51 | 4.38E-02 | up | 1.56 | 1.04E-01 | No |
| *MCM6* | BC032374 | ENSG00000076003 | e14 | Unknown | up | 1.51 | 1.41E-02 | up | 1.42 | 1.17E-01 | No |
| *NHS* | AY436752 | ENSG00000188158 | e7 | Unknown | up | 1.51 | 6.26E-03 | up | 1.43 | 1.81E-01 | No |
| *MAP7* | BC025777 | ENSG00000135525 | e14 | exon_skipping | up | 1.51 | 4.13E-02 | up | 1.20 | 3.48E-01 | Yes |
| *CENPI* | AK302986 | ENSG00000102384 | e21 | Unknown | up | 1.51 | 3.54E-03 | up | 1.24 | 4.02E-01 | No |
| *---* | BC014373 | --- | ae1 | alternative_donor_splice_site | up | 1.51 | 2.14E-02 | up | 1.14 | 4.07E-01 | Yes |
| *MAPKAPK3* | BC068497 | ENSG00000114738 | e10 | Unknown | up | 1.51 | 3.98E-03 | up | 1.12 | 4.60E-01 | No |
| *NPM3* | AY049737 | ENSG00000107833 | e3 | Unknown | up | 1.51 | 6.70E-03 | up | 1.06 | 8.19E-01 | No |
| *PRMT7* | AK001502 | ENSG00000132600 | e10 | Unknown | up | 1.51 | 1.72E-03 | up | 1.02 | 8.50E-01 | No |
| *SLC10A3* | CR622264 | ENSG00000126903 | ae3 | intron_retention | up | 1.51 | 1.66E-02 | up | 1.02 | 9.04E-01 | No |
| *PSEN2* | AK292299 | ENSG00000143801 | e2 | Unknown | up | 1.51 | 1.42E-02 | up | 1.01 | 9.37E-01 | No |
| *CARS* | BC002880 | ENSG00000110619 | e23 | exon_skipping | up | 1.51 | 9.70E-03 | down | 1.45 | 2.13E-02 | No |
| *CD33* | AK090470 | ENSG00000105383 | e8 | alternative_last_exon | up | 1.51 | 4.17E-02 | down | 1.47 | 6.56E-02 | Yes |
| *SI* | X63597 | ENSG00000090402 | e6 | Unknown | up | 1.51 | 3.43E-02 | down | 3.69 | 9.42E-02 | No |
| *VPS13D* | AJ608775 | ENSG00000048707 | e63 | Unknown | up | 1.51 | 5.40E-04 | down | 1.29 | 1.06E-01 | No |
| *SORBS2* | AB018320 | ENSG00000154556 | e27 | Unknown | up | 1.51 | 3.09E-02 | down | 1.50 | 1.22E-01 | Yes |
| *DLG1* | EF553524 | ENSG00000075711 | e3 | exon_skipping,alternative_first_exon | up | 1.51 | 4.39E-02 | down | 1.24 | 1.71E-01 | Yes |
| *CAB39* | BC020570 | ENSG00000135932 | e6 | Unknown | up | 1.51 | 3.76E-12 | down | 1.28 | 1.85E-01 | Yes |
| *ARHGAP32* | EF127492 | ENSG00000134909 | e3 | Unknown | up | 1.51 | 1.17E-02 | down | 1.31 | 2.86E-01 | No |
| *GET4 // SUN1* | AK022816 | ENSG00000164828 // ENSG00000239857 | e33 | Unknown | up | 1.51 | 3.02E-03 | down | 1.18 | 3.12E-01 | No |
| *CENPJ* | AK292406 | ENSG00000151849 | e6 | Unknown | up | 1.51 | 3.95E-02 | down | 1.24 | 3.20E-01 | No |
| *EP400* | AY044869 | ENSG00000183495 | e42 | Unknown | up | 1.51 | 1.22E-02 | down | 1.16 | 3.84E-01 | Yes |
| *EP400* | AY044869 | ENSG00000183495 | e38 | Unknown | up | 1.51 | 4.20E-02 | down | 1.16 | 3.84E-01 | Yes |
| *RGPD5* | U64675 | ENSG00000015568 | e8 | Unknown | up | 1.51 | 1.35E-04 | down | 1.14 | 5.49E-01 | Yes |
| *ARHGAP29* | AK310491 | ENSG00000137962 | e7 | Unknown | up | 1.51 | 7.63E-04 | down | 1.23 | 5.54E-01 | Yes |
| *ARHGAP19 // SLIT1* | BC146761 | ENSG00000187122 // ENSG00000213390 | e24 | Unknown | up | 1.51 | 3.01E-02 | down | 1.07 | 5.66E-01 | No |
| *AKAP7* | AK300587 | ENSG00000118507 | e6 | Unknown | up | 1.51 | 4.43E-02 | down | 1.15 | 5.89E-01 | No |
| *COL12A1* | BC146868 | ENSG00000111799 | e8 | exon_skipping | up | 1.51 | 1.11E-02 | down | 1.05 | 9.23E-01 | Yes |
| *KIAA0146* | D63480 | ENSG00000164808 | e11 | exon_skipping,exon_skipping | up | 1.51 | 3.30E-03 | down | 1.01 | 9.52E-01 | No |
| *DUS1L* | CR614184 | ENSG00000169718 | e4 | exon_skipping | up | 1.50 | 5.80E-03 | up | 1.33 | 4.97E-08 | No |
| *SMARCA5* | AK302841 | ENSG00000153147 | e8 | Unknown | up | 1.50 | 6.58E-03 | up | 1.48 | 6.18E-02 | No |
| *NCAPG* | AF331796 | ENSG00000109805 | e2 | Unknown | up | 1.50 | 2.60E-03 | up | 1.70 | 6.84E-02 | No |
| *PRPF6* | AK225993 | ENSG00000101161 | e7 | Unknown | up | 1.50 | 4.10E-03 | up | 1.21 | 2.46E-01 | No |
| *C12orf73* | BC044618 | ENSG00000204954 | e3 | exon_skipping | up | 1.50 | 1.68E-02 | up | 1.21 | 3.79E-01 | No |
| *POLRMT* | AK302877 | ENSG00000099821 | e14 | exon_skipping | up | 1.50 | 3.03E-04 | up | 1.05 | 6.10E-01 | No |
| *ZNF273* | AX746558 | ENSG00000198039 | e5 | exon_skipping,alternative_first_exon | up | 1.50 | 3.74E-02 | up | 1.06 | 6.19E-01 | No |
| *FOSB* | L49169 | ENSG00000125740 | e1 | alternative_first_exon | up | 1.50 | 3.65E-02 | up | 1.09 | 8.06E-01 | No |
| *CYP20A1* | AK021770 | ENSG00000119004 | e5 | Unknown | up | 1.50 | 3.29E-02 | down | 1.10 | 6.65E-01 | No |
| *FBXL20* | AK291844 | ENSG00000108306 | e7 | exon_skipping | up | 1.50 | 1.73E-02 | down | 1.06 | 7.88E-01 | Yes |
| *ATF6B* | U52696 | ENSG00000213676 | e22 | Unknown | down | 5.79 | 1.03E-07 | up | 1.08 | 6.15E-01 | No |
| *ATF6B* | U52696 | ENSG00000213676 | e27 | Unknown | down | 4.83 | 2.66E-08 | up | 1.08 | 6.15E-01 | No |
| *HSPA8* | AK129885 | ENSG00000109971 | ae6 | intron_retention | down | 4.29 | 2.21E-04 | up | 1.21 | 1.84E-02 | No |
| *FAIM2* | AB023167 | ENSG00000135472 | e4 | Unknown | down | 3.83 | 1.44E-02 | up | 1.67 | 1.79E-01 | No |
| *C2 // CFB* | AK096258 | ENSG00000204359 // ENSG00000206372 | e9 | exon_skipping | down | 3.63 | 2.22E-04 | up | 2.68 | 9.04E-02 | No |
| *C2 // CFB* | AK096258 | ENSG00000204359 // ENSG00000206372 | e8 | exon_skipping,alternative_last_exon | down | 3.52 | 2.97E-04 | up | 2.68 | 9.04E-02 | No |
| *C2 // CFB* | AK096258 | ENSG00000204359 // ENSG00000206372 | e6 | Unknown | down | 3.50 | 9.42E-04 | up | 2.68 | 9.04E-02 | No |
| *C2 // CFB* | AK096258 | ENSG00000204359 // ENSG00000206372 | e19 | exon_skipping | down | 3.47 | 4.20E-04 | up | 2.68 | 9.04E-02 | No |
| *C2 // CFB* | AK096258 | ENSG00000204359 // ENSG00000206372 | e11 | Unknown | down | 3.43 | 3.36E-04 | up | 2.68 | 9.04E-02 | No |
| *MAST2* | AB047005 | ENSG00000086015 | e7 | exon_skipping | down | 3.41 | 1.87E-05 | down | 1.35 | 1.41E-08 | No |
| *---* | AF465484 | --- | e18 | Unknown | down | 3.37 | 3.30E-03 | up | 1.93 | 7.85E-02 | No |
| *C2 // CFB* | AK096258 | ENSG00000204359 // ENSG00000206372 | e18 | Unknown | down | 3.28 | 4.83E-04 | up | 2.68 | 9.04E-02 | No |
| *C2 // CFB* | AK096258 | ENSG00000204359 // ENSG00000206372 | e10 | Unknown | down | 3.27 | 4.22E-04 | up | 2.68 | 9.04E-02 | No |
| *C2 // CFB* | AK096258 | ENSG00000204359 // ENSG00000206372 | e15 | Unknown | down | 3.16 | 2.24E-03 | up | 2.68 | 9.04E-02 | Yes |
| *HSP90AA1* | AK056446 | ENSG00000080824 | ae11 | alternative_last_exon | down | 3.14 | 3.18E-04 | up | 1.38 | 2.70E-02 | No |
| *FRMD4B* | AK091076 | ENSG00000114541 | e9 | alternative_first_exon,exon_skipping | down | 3.14 | 1.68E-02 | up | 1.07 | 7.68E-01 | No |
| *---* | AF465484 | --- | e22 | Unknown | down | 3.12 | 1.14E-02 | up | 1.93 | 7.85E-02 | No |
| *C2 // CFB* | AK096258 | ENSG00000204359 // ENSG00000206372 | e17 | Unknown | down | 3.09 | 2.99E-04 | up | 2.68 | 9.04E-02 | No |
| *SPTBN1* | AB371586 | ENSG00000115306 | e3 | Unknown | down | 3.06 | 8.28E-03 | down | 1.15 | 3.64E-01 | No |
| *FAIM2* | AB023167 | ENSG00000135472 | e5 | Unknown | down | 3.04 | 7.54E-03 | up | 1.67 | 1.79E-01 | No |
| *---* | AF465484 | --- | e26 | Unknown | down | 3.02 | 9.90E-03 | up | 1.93 | 7.85E-02 | No |
| *C2 // CFB* | AK096258 | ENSG00000204359 // ENSG00000206372 | e20 | alternative_last_exon,exon_skipping | down | 2.98 | 5.46E-03 | up | 2.68 | 9.04E-02 | No |
| *C2 // CFB* | AK096258 | ENSG00000204359 // ENSG00000206372 | e7 | exon_skipping | down | 2.98 | 1.90E-03 | up | 2.68 | 9.04E-02 | No |
| *TACC1* | AK303596 | ENSG00000147526 | e2 | exon_skipping | down | 2.97 | 4.00E-04 | down | 1.00 | 9.83E-01 | No |
| *C5orf4* | BC007216 | ENSG00000170271 | e2 | exon_skipping | down | 2.95 | 9.38E-03 | down | 1.40 | 3.14E-01 | No |
| *NEDD4L* | AB071179 | ENSG00000049759 | e5 | exon_skipping,alternative_first_exon | down | 2.94 | 3.08E-10 | down | 1.30 | 2.57E-01 | No |
| *---* | BC026300 | --- | e1 | alternative_first_exon | down | 2.94 | 1.23E-02 | down | 1.09 | 7.58E-01 | Yes |
| *C2 // CFB* | AK096258 | ENSG00000204359 // ENSG00000206372 | e5 | exon_skipping | down | 2.89 | 2.12E-03 | up | 2.68 | 9.04E-02 | No |
| *AMACR // C1QTNF3* | AB209261 | ENSG00000082196 // ENSG00000242110 | e7 | alternative_first_exon | down | 2.89 | 6.00E-03 | up | 1.20 | 3.53E-01 | No |
| *EPB41L2* | AB209522 | ENSG00000079819 | e18 | exon_skipping | down | 2.86 | 4.83E-04 | up | 1.42 | 1.61E-01 | No |
| *SCN3A* | AF225987 | ENSG00000153253 | e18 | Unknown | down | 2.85 | 2.42E-04 | down | 1.19 | 1.57E-01 | Yes |
| *FAIM2* | AB023167 | ENSG00000135472 | e6 | Unknown | down | 2.78 | 2.13E-02 | up | 1.67 | 1.79E-01 | No |
| *AMACR // C1QTNF3* | AB209261 | ENSG00000082196 // ENSG00000242110 | e8 | alternative_last_exon | down | 2.77 | 3.84E-03 | up | 1.20 | 3.53E-01 | No |
| *GGT1* | M24903 | ENSG00000100031 | e17 | Unknown | down | 2.74 | 3.90E-03 | up | 1.50 | 2.14E-01 | No |
| *FANCG* | AJ007669 | ENSG00000221829 | e11 | exon_skipping | down | 2.72 | 4.03E-04 | up | 1.41 | 3.19E-02 | No |
| *---* | BC089413 | --- | e5 | exon_skipping | down | 2.72 | 2.58E-07 | down | 2.07 | 1.83E-01 | No |
| *AMACR // C1QTNF3* | AB209261 | ENSG00000082196 // ENSG00000242110 | e6 | alternative_first_exon | down | 2.68 | 1.70E-03 | up | 1.20 | 3.53E-01 | No |
| *PTPRD* | L38929 | ENSG00000153707 | e15 | exon_skipping | down | 2.67 | 1.73E-02 | up | 1.23 | 5.23E-01 | No |
| *C2 // CFB* | AK096258 | ENSG00000204359 // ENSG00000206372 | e16 | Unknown | down | 2.63 | 1.16E-02 | up | 2.68 | 9.04E-02 | Yes |
| *HSPA8* | AK129885 | ENSG00000109971 | ae5 | intron_retention | down | 2.61 | 3.88E-03 | up | 1.21 | 1.84E-02 | No |
| *C2 // CFB* | AK096258 | ENSG00000204359 // ENSG00000206372 | e13 | Unknown | down | 2.61 | 1.53E-02 | up | 2.68 | 9.04E-02 | Yes |
| *EPB41L2* | AB209522 | ENSG00000079819 | e17 | exon_skipping | down | 2.59 | 9.94E-03 | up | 1.42 | 1.61E-01 | No |
| *FAM107A* | AK054720 | ENSG00000168309 | e8 | alternative_last_exon | down | 2.59 | 6.32E-05 | down | 1.34 | 6.64E-03 | No |
| *---* | BC073889 | --- | e7 | Unknown | down | 2.56 | 4.17E-02 | down | 1.17 | 5.02E-01 | Yes |
| *---* | AF465484 | --- | e21 | Unknown | down | 2.54 | 2.46E-02 | up | 1.93 | 7.85E-02 | No |
| *C2 // CFB* | AK096258 | ENSG00000204359 // ENSG00000206372 | e3 | exon_skipping,alternative_first_exon | down | 2.49 | 2.14E-02 | up | 2.68 | 9.04E-02 | No |
| *---* | AF465484 | --- | e33 | alternative_last_exon | down | 2.48 | 1.06E-02 | up | 1.93 | 7.85E-02 | No |
| *AMACR // C1QTNF3* | AB209261 | ENSG00000082196 // ENSG00000242110 | e2 | exon_skipping,alternative_first_exon | down | 2.44 | 2.74E-03 | up | 1.20 | 3.53E-01 | No |
| *---* | AF465484 | --- | e28 | Unknown | down | 2.43 | 1.40E-02 | up | 1.93 | 7.85E-02 | No |
| *IL33* | BC047085 | ENSG00000137033 | e1 | alternative_first_exon | down | 2.39 | 4.37E-02 | up | 1.91 | 2.23E-01 | Yes |
| *---* | M29874 | --- | e1 | alternative_first_exon | down | 2.38 | 4.97E-02 | up | 3.17 | 5.80E-02 | No |
| *---* | AK001299 | --- | ae3 | intron_retention | down | 2.38 | 7.97E-05 | down | 1.63 | 2.20E-01 | Yes |
| *MAST2* | AB047005 | ENSG00000086015 | e6 | exon_skipping,alternative_first_exon | down | 2.37 | 1.96E-03 | down | 1.35 | 1.41E-08 | No |
| *FLNC* | AB371585 | ENSG00000128591 | e7 | Unknown | down | 2.37 | 1.48E-03 | down | 1.37 | 1.24E-01 | No |
| *RNFT2* | BC011878 | ENSG00000135119 | e7 | exon_skipping,alternative_first_exon | down | 2.34 | 9.92E-03 | down | 1.22 | 9.95E-04 | No |
| *---* | BC071971 | --- | e2 | Unknown | down | 2.32 | 5.43E-07 | up | 1.38 | 1.54E-01 | Yes |
| *VCL* | BX537994 | ENSG00000035403 | e23 | exon_skipping | down | 2.32 | 1.94E-03 | down | 1.07 | 1.17E-01 | No |
| *LRRFIP2* | AK291514 | ENSG00000093167 | e8 | exon_skipping | down | 2.31 | 1.13E-02 | down | 1.22 | 2.60E-01 | No |
| *---* | AK291987 | --- | e1 | alternative_first_exon | down | 2.29 | 3.84E-02 | up | 3.59 | 2.20E-01 | No |
| *PDE4DIP* | AB007923 | ENSG00000178104 | e4 | Unknown | down | 2.29 | 8.58E-03 | down | 1.14 | 4.88E-01 | No |
| *DCTN1 // SLC4A5* | AF207661 | ENSG00000188687 // ENSG00000204843 | e62 | exon_skipping | down | 2.28 | 3.92E-03 | up | 1.02 | 7.96E-01 | No |
| *CYP2B7P1* | BC041174 | --- | e2 | Unknown | down | 2.25 | 4.42E-03 | down | 1.23 | 6.00E-01 | No |
| *SBF2* | AY234241 | ENSG00000133812 | ae27 | alternative_last_exon | down | 2.21 | 2.74E-02 | up | 1.16 | 2.30E-01 | No |
| *ATP5SL* | AK298018 | ENSG00000105341 | e3 | exon_skipping | down | 2.20 | 6.92E-08 | up | 1.24 | 9.06E-02 | No |
| *SESN1* | AF033122 | ENSG00000080546 | e1 | alternative_first_exon | down | 2.20 | 2.05E-02 | up | 1.84 | 1.37E-01 | Yes |
| *EDEM2* | AK296878 | ENSG00000088298 | e4 | Unknown | down | 2.19 | 4.32E-02 | up | 1.20 | 2.77E-01 | No |
| *LAT // SPNS1* | AB209402 | ENSG00000169682 // ENSG00000213658 | e16 | Unknown | down | 2.16 | 3.31E-02 | up | 1.40 | 3.77E-02 | No |
| *AMACR // C1QTNF3* | AB209261 | ENSG00000082196 // ENSG00000242110 | e4 | Unknown | down | 2.16 | 7.74E-03 | up | 1.20 | 3.53E-01 | No |
| *MMP3* | AK223291 | ENSG00000149968 | e10 | alternative_last_exon | down | 2.15 | 6.96E-03 | up | 4.52 | 5.70E-02 | No |
| *VPS53* | BC040223 | ENSG00000141252 | ae22 | intron_retention,alternative_last_exon | down | 2.15 | 2.22E-02 | up | 1.30 | 7.23E-02 | No |
| *---* | AF020640 | --- | e13 | exon_skipping | down | 2.15 | 4.22E-03 | down | 1.17 | 3.20E-01 | No |
| *HNF4A* | AY680697 | ENSG00000101076 | e3 | exon_skipping,alternative_first_exon | down | 2.15 | 4.84E-02 | down | 1.24 | 3.54E-01 | No |
| *ERCC1* | AX747328 | ENSG00000012061 | ae10 | alternative_last_exon | down | 2.14 | 1.62E-12 | up | 1.28 | 1.34E-01 | No |
| *SLC39A14* | BC015770 | ENSG00000104635 | e5 | exon_skipping | down | 2.14 | 5.64E-03 | up | 1.20 | 3.08E-01 | No |
| *CARS2* | AK301907 | ENSG00000134905 | e17 | exon_skipping | down | 2.12 | 8.58E-03 | up | 1.32 | 6.49E-02 | No |
| *C7orf49* | BC050676 | ENSG00000122783 | e6 | alternative_last_exon | down | 2.12 | 4.39E-04 | up | 1.34 | 1.32E-01 | No |
| *C1orf175 // TTC4* | CR749830 | ENSG00000184313 // ENSG00000243725 | e24 | Unknown | down | 2.12 | 3.50E-03 | up | 1.24 | 1.49E-01 | No |
| *THOC1* | BC010381 | ENSG00000079134 | e12 | alternative_first_exon,exon_skipping | down | 2.12 | 2.13E-02 | up | 1.26 | 2.45E-01 | No |
| *OSBP2* | AK131374 | ENSG00000184792 | e10 | exon_skipping,alternative_first_exon | down | 2.10 | 2.08E-02 | up | 1.36 | 2.06E-02 | No |
| *MMADHC* | AF161510 | ENSG00000168288 | e5 | Unknown | down | 2.10 | 2.12E-02 | up | 1.42 | 1.12E-01 | Yes |
| *ACTN1* | AK098203 | ENSG00000072110 | e22 | exon_skipping | down | 2.09 | 1.39E-02 | up | 1.43 | 3.80E-04 | No |
| *PSPH* | BC063614 | ENSG00000146733 | e4 | exon_skipping | down | 2.08 | 2.84E-02 | up | 1.51 | 1.83E-01 | No |
| *WDR13* | AF329819 | ENSG00000101940 | ae6 | alternative_last_exon | down | 2.07 | 5.76E-03 | up | 1.20 | 2.41E-01 | No |
| *MSH4 // RABGGTB* | BC033030 | ENSG00000057468 // ENSG00000137955 | e31 | Unknown | down | 2.06 | 1.18E-02 | up | 1.47 | 1.23E-01 | No |
| *FRMD4B* | AK091076 | ENSG00000114541 | e7 | exon_skipping | down | 2.06 | 3.04E-02 | up | 1.07 | 7.68E-01 | No |
| *DCP1B* | AK299203 | ENSG00000151065 | ae12 | alternative_last_exon | down | 2.05 | 1.50E-03 | up | 1.36 | 5.58E-02 | No |
| *PRDX5* | CR608340 | ENSG00000126432 | e7 | alternative_last_exon | down | 2.05 | 2.71E-02 | up | 1.62 | 7.35E-02 | No |
| *NUDCD3* | AB028991 | ENSG00000015676 | e6 | exon_skipping | down | 2.05 | 4.64E-02 | up | 1.47 | 7.61E-02 | No |
| *NEU1 // SLC44A4* | AF466766 | ENSG00000204385 // ENSG00000204386 // ENSG00000227129 // ENSG00000228263 | e3 | Unknown | down | 2.05 | 4.10E-02 | up | 1.51 | 1.83E-01 | No |
| *HTRA2* | AF141305 | ENSG00000115317 | ae6 | intron_retention | down | 2.05 | 2.16E-02 | up | 1.23 | 1.97E-01 | No |
| *EGFL8 // PPT2* | AF020544 | ENSG00000227600 // ENSG00000240389 // ENSG00000241404 | e16 | Unknown | down | 2.05 | 3.29E-02 | up | 1.28 | 2.94E-01 | No |
| *TACC1* | AK303596 | ENSG00000147526 | ae4 | alternative_last_exon | down | 2.05 | 1.21E-02 | down | 1.00 | 9.83E-01 | Yes |
| *MYO18A // TIAF1* | D86970 | ENSG00000196535 // ENSG00000221995 | e48 | exon_skipping | down | 2.04 | 1.85E-02 | up | 1.07 | 6.83E-01 | No |
| *---* | AK311167 | --- | e7 | alternative_last_exon,exon_skipping | down | 2.03 | 3.71E-02 | up | 1.48 | 7.16E-02 | Yes |
| *GIMAP1 // GIMAP2* | CR608105 | ENSG00000106560 // ENSG00000213203 | e6 | alternative_last_exon | down | 2.03 | 3.20E-02 | down | 1.22 | 4.23E-01 | No |
| *DMKN* | BC035311 | ENSG00000161249 | e20 | exon_skipping,alternative_last_exon | down | 2.02 | 3.93E-05 | up | 1.85 | 2.16E-01 | No |
| *GOPC // ROS1* | X51619 | ENSG00000047932 // ENSG00000047936 | e24 | exon_skipping | down | 2.02 | 3.51E-02 | up | 1.13 | 5.73E-01 | No |
| *GPN1 // ZNF512* | AK303566 | ENSG00000198522 // ENSG00000243943 | e3 | exon_skipping | down | 2.01 | 3.44E-02 | up | 1.26 | 2.09E-01 | No |
| *AXIN2 // CCDC46* | AK299075 | ENSG00000154240 // ENSG00000168646 | e15 | Unknown | down | 2.01 | 1.46E-02 | up | 1.30 | 2.24E-01 | No |
| *PANK1* | BC156381 | ENSG00000152782 | e3 | exon_skipping,alternative_first_exon | down | 2.01 | 3.51E-02 | up | 1.34 | 3.99E-01 | No |
| *MKL2* | AK294765 | ENSG00000186260 | e3 | exon_skipping,alternative_first_exon | down | 2.00 | 4.85E-10 | down | 1.13 | 3.05E-01 | No |
| *CYP4F2* | U02388 | ENSG00000186115 | e4 | Unknown | down | 2.00 | 1.99E-02 | down | 1.40 | 3.84E-01 | Yes |
| *HS2ST1* | AB007917 | ENSG00000153936 | e12 | Unknown | down | 2.00 | 7.96E-03 | down | 1.07 | 7.69E-01 | No |
| *FHL2* | U29332 | ENSG00000115641 | e1 | alternative_first_exon | down | 1.99 | 2.77E-02 | up | 1.49 | 8.37E-02 | No |
| *CEP57* | AK098647 | ENSG00000166037 | ae9 | alternative_last_exon | down | 1.99 | 6.12E-03 | up | 1.30 | 2.00E-01 | No |
| *SLC5A6* | AF069307 | ENSG00000138074 | ae18 | intron_retention | down | 1.98 | 2.74E-02 | up | 1.75 | 5.11E-02 | Yes |
| *FRMD4B* | AK091076 | ENSG00000114541 | e8 | exon_skipping,alternative_last_exon | down | 1.98 | 2.75E-02 | up | 1.07 | 7.68E-01 | No |
| *MANBAL* | BC016822 | ENSG00000101363 | ae4 | alternative_acceptor_splice_site | down | 1.96 | 1.11E-02 | up | 1.40 | 5.01E-02 | No |
| *C3orf31* | AX747723 | ENSG00000144559 | ae12 | alternative_last_exon | down | 1.96 | 2.86E-02 | up | 1.68 | 7.04E-02 | No |
| *AXIN2 // CCDC46* | AK299075 | ENSG00000154240 // ENSG00000168646 | e7 | Unknown | down | 1.96 | 1.42E-02 | up | 1.30 | 2.24E-01 | No |
| *PLCB1* | AB011153 | ENSG00000182621 | e5 | exon_skipping | down | 1.96 | 4.81E-02 | up | 1.56 | 3.17E-01 | No |
| *FKBP1A // SDCBP2* | AK309026 | ENSG00000088832 // ENSG00000125775 | e8 | Unknown | down | 1.96 | 2.14E-02 | down | 1.30 | 2.19E-01 | No |
| *HS2ST1* | AB007917 | ENSG00000153936 | e14 | alternative_last_exon | down | 1.96 | 4.22E-07 | down | 1.07 | 7.69E-01 | No |
| *CDC14B* | AF064105 | ENSG00000081377 | e2 | exon_skipping,alternative_first_exon | down | 1.95 | 1.98E-02 | up | 1.15 | 5.77E-01 | No |
| *ITK* | AB209622 | ENSG00000113263 | e7 | exon_skipping | down | 1.95 | 2.24E-03 | up | 1.02 | 9.42E-01 | No |
| *NSUN7* | BC042401 | ENSG00000179299 | e7 | Unknown | down | 1.94 | 5.12E-03 | up | 1.66 | 6.44E-02 | Yes |
| *SGOL2* | BX647433 | ENSG00000163535 | e1 | alternative_first_exon | down | 1.94 | 1.59E-02 | up | 1.59 | 7.03E-02 | Yes |
| *SCLT1* | AK055217 | ENSG00000151466 | e12 | exon_skipping | down | 1.94 | 1.66E-02 | up | 1.51 | 7.71E-02 | No |
| *DCXR* | AK226040 | ENSG00000169738 | ae6 | intron_retention | down | 1.94 | 3.36E-02 | up | 1.33 | 2.32E-01 | No |
| *ZNF761* | AB107355 | ENSG00000160336 | ae5 | alternative_last_exon | down | 1.94 | 1.53E-02 | up | 1.17 | 3.98E-01 | No |
| *TTPA* | BC041784 | ENSG00000137561 | e2 | Unknown | down | 1.93 | 5.92E-03 | up | 1.76 | 6.80E-02 | Yes |
| *ODF2* | AY366499 | ENSG00000136811 | e1 | alternative_first_exon | down | 1.93 | 1.27E-02 | up | 1.33 | 7.46E-02 | No |
| *C10orf81* | AK126354 | ENSG00000148735 | e9 | exon_skipping,alternative_first_exon | down | 1.93 | 2.48E-06 | up | 1.76 | 1.09E-01 | Yes |
| *AMACR // C1QTNF3* | AB209261 | ENSG00000082196 // ENSG00000242110 | e5 | Unknown | down | 1.93 | 5.66E-03 | up | 1.20 | 3.53E-01 | No |
| *PKM2* | BC094767 | ENSG00000067225 | e13 | exon_skipping | down | 1.92 | 6.92E-03 | up | 1.35 | 2.07E-08 | No |
| *AXIN2 // CCDC46* | AK299075 | ENSG00000154240 // ENSG00000168646 | e8 | exon_skipping | down | 1.91 | 2.18E-02 | up | 1.30 | 2.24E-01 | No |
| *EIF4G2* | AK223548 | ENSG00000110321 | ae2 | alternative_first_exon | down | 1.91 | 8.42E-03 | up | 1.08 | 5.46E-01 | No |
| *---* | BC035792 | --- | e11 | alternative_last_exon | down | 1.90 | 1.98E-02 | up | 1.46 | 5.28E-02 | Yes |
| *C2orf15 // MRPL30* | AK126402 | ENSG00000185414 // ENSG00000241962 | e15 | Unknown | down | 1.90 | 1.88E-02 | up | 1.66 | 8.21E-02 | No |
| *ATF5* | AB073613 | ENSG00000169136 | e1 | alternative_first_exon | down | 1.90 | 7.58E-03 | up | 1.34 | 9.44E-02 | No |
| *SEC22A* | AF100749 | ENSG00000121542 | e6 | Unknown | down | 1.90 | 1.22E-03 | up | 1.25 | 2.09E-01 | No |
| *TMC6* | BC023597 | ENSG00000141524 | e2 | alternative_first_exon,exon_skipping | down | 1.89 | 5.96E-04 | up | 1.33 | 1.21E-02 | No |
| *ATP6V0A2* | BC068531 | ENSG00000185344 | e10 | exon_skipping,alternative_last_exon | down | 1.89 | 3.44E-02 | up | 1.18 | 2.00E-01 | No |
| *NDUFAF2* | BC070357 | ENSG00000164182 | e2 | Unknown | down | 1.89 | 1.52E-02 | up | 1.19 | 4.01E-01 | No |
| *UBA6* | AL832015 | ENSG00000033178 | ae12 | alternative_last_exon | down | 1.88 | 4.30E-03 | up | 1.39 | 1.11E-01 | No |
| *FAM98B* | AK095745 | ENSG00000171262 | e3 | Unknown | down | 1.88 | 2.16E-03 | up | 1.45 | 1.60E-01 | No |
| *LYG1 // TXNDC9* | AK301722 | ENSG00000115514 // ENSG00000144214 | e9 | Unknown | down | 1.88 | 2.79E-02 | up | 1.37 | 1.99E-01 | No |
| *KIAA0895* | BC028678 | ENSG00000164542 | ae3 | alternative_first_exon | down | 1.88 | 3.95E-02 | up | 1.36 | 2.88E-01 | No |
| *GPR133* | AY532280 | ENSG00000111452 | e10 | Unknown | down | 1.88 | 1.26E-02 | down | 1.47 | 9.48E-03 | No |
| *C3orf31* | AX747723 | ENSG00000144559 | e5 | exon_skipping | down | 1.87 | 3.96E-03 | up | 1.68 | 7.04E-02 | No |
| *PTPRB* | BX648245 | ENSG00000127329 | e3 | Unknown | down | 1.87 | 5.55E-08 | up | 1.16 | 3.51E-01 | No |
| *SCLT1* | AK055217 | ENSG00000151466 | e10 | exon_skipping | down | 1.86 | 2.18E-03 | up | 1.51 | 7.71E-02 | No |
| *PAPPA2* | BC152552 | ENSG00000116183 | e12 | Unknown | down | 1.86 | 4.24E-03 | down | 1.37 | 7.12E-03 | No |
| *ELK4 // SLC45A3* | FJ805261 | ENSG00000158711 // ENSG00000158715 | e13 | Unknown | down | 1.86 | 4.64E-03 | down | 1.05 | 6.76E-01 | No |
| *CRCP* | BX647867 | ENSG00000241258 | e3 | exon_skipping | down | 1.85 | 9.00E-03 | up | 1.36 | 4.14E-02 | No |
| *---* | BC067803 | --- | ae14 | alternative_acceptor_splice_site | down | 1.85 | 4.36E-02 | up | 1.59 | 7.56E-02 | Yes |
| *TECR* | BC005384 | ENSG00000099797 | ae11 | intron_retention | down | 1.85 | 4.84E-02 | up | 1.49 | 1.10E-01 | No |
| *FAIM2* | AB023167 | ENSG00000135472 | e7 | Unknown | down | 1.85 | 7.59E-04 | up | 1.67 | 1.79E-01 | No |
| *MAGED1* | AF258554 | ENSG00000179222 | ae8 | intron_retention | down | 1.84 | 2.13E-02 | up | 1.26 | 1.77E-05 | No |
| *MLF2* | AK313998 | ENSG00000089693 | ae2 | alternative_first_exon,alternative_donor_splice_site | down | 1.84 | 1.33E-02 | up | 1.45 | 5.68E-03 | Yes |
| *TUBBP5* | AK308459 | ENSG00000159247 | e5 | Unknown | down | 1.84 | 5.76E-03 | up | 1.45 | 2.53E-02 | No |
| *KANK1* | BC037495 | ENSG00000107104 | e5 | exon_skipping | down | 1.84 | 4.24E-02 | up | 1.68 | 6.16E-02 | Yes |
| *C3orf31* | AX747723 | ENSG00000144559 | e11 | exon_skipping | down | 1.84 | 8.90E-03 | up | 1.68 | 7.04E-02 | Yes |
| *MTHFD1L* | AY374130 | ENSG00000120254 | e31 | Unknown | down | 1.84 | 3.50E-02 | up | 1.55 | 1.13E-01 | No |
| *MTAP* | AF109294 | ENSG00000099810 | e1 | alternative_first_exon | down | 1.84 | 4.30E-02 | up | 1.38 | 1.37E-01 | Yes |
| *RFWD3* | BC059371 | ENSG00000168411 | e2 | exon_skipping | down | 1.83 | 2.38E-02 | up | 1.24 | 2.90E-01 | No |
| *---* | AK023040 | --- | ae3 | alternative_first_exon | down | 1.83 | 2.92E-02 | up | 1.13 | 5.09E-01 | No |
| *SVIL* | AF051850 | ENSG00000197321 | e4 | exon_skipping,alternative_first_exon | down | 1.83 | 9.46E-03 | down | 1.12 | 4.24E-01 | No |
| *SVIL* | AF051850 | ENSG00000197321 | e12 | exon_skipping | down | 1.83 | 2.38E-02 | down | 1.12 | 4.24E-01 | No |
| *PSMB2* | AB209470 | ENSG00000126067 | e9 | exon_skipping,alternative_last_exon | down | 1.82 | 1.10E-02 | up | 1.39 | 1.73E-02 | Yes |
| *GDPD1* | AY271346 | ENSG00000153982 | e8 | Unknown | down | 1.82 | 6.74E-03 | up | 1.22 | 3.21E-01 | No |
| *FER1L6* | AB196633 | ENSG00000214814 | e35 | Unknown | down | 1.82 | 1.66E-12 | down | 1.84 | 8.87E-02 | No |
| *GPN1 // ZNF512* | AK303566 | ENSG00000198522 // ENSG00000243943 | e5 | Unknown | down | 1.81 | 3.31E-02 | up | 1.26 | 2.09E-01 | No |
| *FAM120AOS* | CR618537 | ENSG00000188938 | e1 | alternative_first_exon | down | 1.81 | 5.80E-03 | up | 1.06 | 7.04E-01 | No |
| *CHMP4A // IPO4 // MDP1 // TM9SF1* | AK300118 | ENSG00000100926 // ENSG00000100931 // ENSG00000196497 // ENSG00000213920 | ae1 | intron_retention | down | 1.80 | 1.71E-02 | up | 1.48 | 7.70E-03 | No |
| *SCLT1* | AK055217 | ENSG00000151466 | e9 | exon_skipping | down | 1.80 | 1.24E-02 | up | 1.51 | 7.71E-02 | No |
| *C9orf86 // KIAA1984* | AF318367 | ENSG00000196642 // ENSG00000213213 | e14 | exon_skipping | down | 1.80 | 4.88E-02 | up | 1.47 | 9.61E-02 | Yes |
| *PSPC1* | BC014184 | ENSG00000121390 | e11 | Unknown | down | 1.80 | 1.96E-03 | up | 1.28 | 2.29E-01 | No |
| *NCRNA00081* | BC073157 | ENSG00000214413 | e1 | alternative_first_exon | down | 1.80 | 1.85E-02 | up | 1.17 | 4.81E-01 | No |
| *KBTBD4* | BC018974 | ENSG00000123444 | ae1 | alternative_donor_splice_site | down | 1.79 | 2.11E-02 | up | 1.33 | 9.33E-02 | No |
| *ACOT13* | AK309738 | ENSG00000112304 | e2 | exon_skipping | down | 1.79 | 1.14E-02 | up | 1.17 | 5.20E-01 | No |
| *REG3G* | AK292595 | ENSG00000143954 | e5 | Unknown | down | 1.79 | 3.69E-02 | down | 2.08 | 1.01E-01 | Yes |
| *MBNL1* | AB007888 | ENSG00000152601 | e13 | exon_skipping | down | 1.79 | 1.85E-02 | down | 1.12 | 2.52E-01 | No |
| *C15orf21* | AK096745 | ENSG00000179362 | e2 | exon_skipping | down | 1.79 | 2.02E-02 | down | 1.18 | 5.89E-01 | No |
| *---* | CR598972 | --- | e3 | alternative_last_exon | down | 1.79 | 1.38E-06 | down | 1.07 | 7.31E-01 | No |
| *HAVCR1* | AK298259 | ENSG00000113249 | e4 | Unknown | down | 1.79 | 8.58E-03 | down | 1.04 | 9.63E-01 | No |
| *KIF23* | BC051826 | ENSG00000137807 | e25 | Unknown | down | 1.78 | 8.94E-03 | up | 1.51 | 1.17E-01 | No |
| *ZNF484* | AK309617 | ENSG00000127081 | e3 | exon_skipping | down | 1.78 | 3.06E-02 | up | 1.28 | 1.64E-01 | No |
| *AXIN2 // CCDC46* | AK299075 | ENSG00000154240 // ENSG00000168646 | e11 | Unknown | down | 1.78 | 2.57E-02 | up | 1.30 | 2.24E-01 | No |
| *PRKAG2* | AJ249976 | ENSG00000106617 | e4 | Unknown | down | 1.78 | 4.99E-02 | up | 1.19 | 3.77E-01 | No |
| *LARP1B* | AL137759 | ENSG00000138709 | e10 | exon_skipping,alternative_last_exon | down | 1.77 | 7.10E-03 | up | 1.57 | 5.86E-02 | Yes |
| *SMTN* | AK128719 | ENSG00000183963 | e15 | exon_skipping | down | 1.77 | 4.23E-02 | up | 1.30 | 1.00E-01 | No |
| *C1orf175 // TTC4* | CR749830 | ENSG00000184313 // ENSG00000243725 | e19 | exon_skipping | down | 1.77 | 4.20E-03 | up | 1.24 | 1.49E-01 | No |
| *---* | AL832582 | --- | e1 | alternative_first_exon | down | 1.77 | 9.20E-04 | up | 1.34 | 1.54E-01 | Yes |
| *CASP8* | U60520 | ENSG00000064012 | ae13 | alternative_donor_splice_site | down | 1.77 | 2.06E-02 | up | 1.34 | 2.00E-01 | Yes |
| *PRKAG2* | AJ249976 | ENSG00000106617 | e6 | alternative_last_exon | down | 1.77 | 2.95E-02 | up | 1.19 | 3.77E-01 | No |
| *PROK2* | AF333025 | ENSG00000163421 | e3 | exon_skipping | down | 1.77 | 4.39E-02 | up | 1.24 | 5.92E-01 | Yes |
| *SEZ6L2* | BC000567 | ENSG00000174938 | e3 | Unknown | down | 1.77 | 4.60E-03 | up | 1.12 | 6.23E-01 | No |
| *CST2* | BC062679 | ENSG00000170369 | e2 | Unknown | down | 1.77 | 3.09E-02 | down | 1.68 | 1.51E-01 | No |
| *STARD13* | BX647695 | ENSG00000133121 | e2 | Unknown | down | 1.77 | 4.17E-02 | down | 1.16 | 5.09E-01 | No |
| *CHMP4A // IPO4 // MDP1 // TM9SF1* | AK300118 | ENSG00000100926 // ENSG00000100931 // ENSG00000196497 // ENSG00000213920 | e3 | Unknown | down | 1.76 | 2.70E-02 | up | 1.48 | 7.70E-03 | No |
| *NR2C1* | BC026074 | ENSG00000120798 | e1 | alternative_first_exon | down | 1.76 | 7.80E-03 | up | 1.52 | 5.11E-02 | Yes |
| *THEM4* | BC017339 | ENSG00000159445 | e3 | exon_skipping,alternative_first_exon | down | 1.76 | 2.55E-02 | up | 1.54 | 8.06E-02 | Yes |
| *MYO19* | AK293903 | ENSG00000141140 | e1 | alternative_first_exon | down | 1.76 | 1.15E-02 | up | 1.33 | 1.14E-01 | Yes |
| *ADAM15* | AB209157 | ENSG00000143537 | e22 | exon_skipping,alternative_last_exon | down | 1.76 | 3.72E-02 | up | 1.29 | 1.18E-01 | No |
| *KIF20B* | AB033337 | ENSG00000138182 | e29 | Unknown | down | 1.76 | 1.48E-03 | up | 1.40 | 2.14E-01 | No |
| *TCTN1* | DQ278868 | ENSG00000204852 | e1 | alternative_first_exon | down | 1.76 | 1.75E-02 | up | 1.30 | 2.23E-01 | No |
| *GYG2* | AK098046 | ENSG00000056998 | e3 | exon_skipping | down | 1.76 | 8.52E-07 | up | 1.49 | 2.27E-01 | Yes |
| *TRA@ // TRAC // TRD@* | FJ009444 | ENSG00000211889 // ENSG00000229164 | e125 | exon_skipping,alternative_last_exon | down | 1.76 | 3.91E-08 | down | 1.32 | 6.11E-02 | No |
| *RFC5* | BC001866 | ENSG00000111445 | e2 | exon_skipping | down | 1.75 | 2.79E-02 | up | 1.40 | 1.25E-01 | No |
| *CARD16* | AY885669 | ENSG00000204397 | e1 | alternative_first_exon | down | 1.75 | 1.28E-02 | up | 1.31 | 2.34E-01 | Yes |
| *EGFL8 // PPT2* | AF020544 | ENSG00000227600 // ENSG00000240389 // ENSG00000241404 | e18 | Unknown | down | 1.75 | 7.81E-11 | up | 1.28 | 2.94E-01 | No |
| *DDHD2* | AK125904 | ENSG00000085788 | e6 | alternative_last_exon,exon_skipping | down | 1.75 | 2.00E-04 | up | 1.06 | 7.88E-01 | No |
| *CCT5* | AK301760 | ENSG00000150753 | ae10 | alternative_last_exon | down | 1.74 | 3.54E-02 | up | 1.47 | 1.82E-02 | Yes |
| *TECPR2* | BC151230 | ENSG00000196663 | e1 | alternative_first_exon | down | 1.74 | 1.73E-02 | up | 1.27 | 2.35E-02 | Yes |
| *TOP3B* | AK290997 | ENSG00000100038 | e6 | exon_skipping | down | 1.74 | 5.92E-03 | up | 1.29 | 3.04E-02 | No |
| *FXR2* | BC067272 | ENSG00000129245 | e1 | alternative_first_exon | down | 1.74 | 1.08E-02 | up | 1.47 | 3.19E-02 | No |
| *ABCC10* | AK000002 | ENSG00000124574 | e7 | Unknown | down | 1.74 | 8.15E-05 | up | 1.35 | 3.64E-02 | No |
| *TUBBP5* | AK308459 | ENSG00000159247 | e3 | exon_skipping,alternative_first_exon | down | 1.74 | 2.43E-02 | up | 1.39 | 3.76E-02 | Yes |
| *NIPA1* | BX537997 | ENSG00000170113 | e3 | Unknown | down | 1.74 | 2.95E-02 | up | 1.26 | 1.07E-01 | Yes |
| *KRT6A* | BC014152 | ENSG00000205420 | e3 | Unknown | down | 1.74 | 2.87E-02 | up | 1.92 | 1.53E-01 | Yes |
| *DNMT3A* | AB208833 | ENSG00000119772 | e7 | Unknown | down | 1.74 | 5.86E-08 | up | 1.24 | 2.79E-01 | Yes |
| *ATP8B2* | BC140441 | ENSG00000143515 | e5 | Unknown | down | 1.74 | 2.55E-02 | down | 1.28 | 1.08E-03 | No |
| *FLNC* | AB371585 | ENSG00000128591 | e12 | Unknown | down | 1.74 | 2.55E-02 | down | 1.37 | 1.24E-01 | No |
| *GPD2* | AB209399 | ENSG00000115159 | e3 | exon_skipping,alternative_first_exon | down | 1.74 | 5.54E-08 | down | 1.05 | 6.97E-01 | No |
| *COPS7A* | AK128792 | ENSG00000111652 | ae2 | alternative_first_exon,internal_exon_deletion | down | 1.73 | 1.19E-02 | up | 1.40 | 2.47E-02 | No |
| *RBX1 // XPNPEP3* | AK301758 | ENSG00000100387 // ENSG00000196236 | ae3 | alternative_last_exon | down | 1.73 | 2.66E-02 | up | 1.39 | 8.18E-02 | Yes |
| *TYW1* | AK001762 | ENSG00000198874 | e3 | Unknown | down | 1.73 | 4.78E-02 | up | 1.40 | 1.21E-01 | No |
| *USP21* | AB208899 | ENSG00000143258 | ae9 | intron_retention | down | 1.73 | 7.52E-03 | up | 1.20 | 2.24E-01 | No |
| *TDG* | BC037557 | ENSG00000139372 | ae4 | alternative_last_exon | down | 1.73 | 3.67E-02 | up | 1.25 | 2.47E-01 | No |
| *SAFB2* | D50928 | ENSG00000130254 | ae6 | alternative_first_exon,alternative_last_exon | down | 1.73 | 4.10E-02 | up | 1.20 | 3.31E-01 | No |
| *GLTSCR2* | DQ895973 | ENSG00000105373 | e16 | alternative_last_exon | down | 1.73 | 8.31E-08 | up | 1.09 | 6.34E-01 | No |
| *CLSTN2* | AJ278018 | ENSG00000158258 | e3 | Unknown | down | 1.73 | 3.28E-02 | down | 1.29 | 5.72E-02 | No |
| *RAB37* | AK127073 | ENSG00000172794 | e8 | Unknown | down | 1.73 | 3.72E-02 | down | 1.62 | 1.28E-01 | No |
| *KPNB1* | AK316421 | ENSG00000108424 | ae2 | alternative_first_exon | down | 1.72 | 4.72E-02 | up | 1.41 | 1.81E-02 | Yes |
| *RAB11FIP4* | AJ314646 | ENSG00000131242 | e3 | Unknown | down | 1.72 | 1.08E-02 | up | 1.40 | 3.53E-02 | No |
| *MMP3* | AK223291 | ENSG00000149968 | e8 | Unknown | down | 1.72 | 4.26E-03 | up | 4.52 | 5.70E-02 | No |
| *MARK3* | AF465413 | ENSG00000075413 | e5 | Unknown | down | 1.72 | 5.77E-07 | up | 1.32 | 7.06E-02 | No |
| *C9orf86 // KIAA1984* | AF318367 | ENSG00000196642 // ENSG00000213213 | ae23 | intron_retention | down | 1.72 | 3.43E-02 | up | 1.47 | 9.61E-02 | No |
| *KIAA1841* | BC070104 | ENSG00000162929 | e23 | exon_skipping | down | 1.72 | 1.34E-02 | up | 1.31 | 1.50E-01 | No |
| *XPO1* | BX647758 | ENSG00000082898 | e6 | exon_skipping | down | 1.72 | 1.01E-02 | up | 1.40 | 2.14E-01 | No |
| *DNAJC8* | CR619944 | ENSG00000126698 | e1 | alternative_first_exon | down | 1.72 | 6.44E-03 | up | 1.35 | 3.31E-01 | No |
| *NOL12 // TRIOBP* | DQ278603 | ENSG00000100101 // ENSG00000100106 | e14 | alternative_last_exon | down | 1.72 | 2.89E-02 | up | 1.07 | 4.50E-01 | Yes |
| *GSTM3* | CR614504 | ENSG00000134202 | e9 | alternative_last_exon | down | 1.72 | 1.54E-02 | up | 1.30 | 5.87E-01 | Yes |
| *SLC25A34* | BC027998 | ENSG00000162461 | ae5 | intron_retention | down | 1.72 | 1.31E-02 | down | 1.41 | 1.75E-02 | No |
| *RERE* | AB036737 | ENSG00000142599 | ae10 | alternative_first_exon | down | 1.72 | 1.39E-02 | down | 1.06 | 6.65E-01 | No |
| *CD52* | CR607239 | ENSG00000169442 | e1 | alternative_first_exon | down | 1.72 | 3.06E-02 | down | 1.03 | 8.77E-01 | No |
| *BCL2L13* | AL831982 | ENSG00000099968 | e7 | exon_skipping | down | 1.71 | 2.08E-02 | up | 1.43 | 1.54E-02 | No |
| *RPL23AP7* | BC000596 | ENSG00000228003 | e1 | alternative_first_exon | down | 1.71 | 4.19E-02 | up | 1.27 | 4.71E-02 | Yes |
| *ESPL1* | D79987 | ENSG00000135476 | e22 | Unknown | down | 1.71 | 4.13E-02 | up | 1.45 | 9.53E-02 | No |
| *CUL2* | AK095217 | ENSG00000108094 | e3 | exon_skipping,alternative_first_exon | down | 1.71 | 1.62E-02 | up | 1.29 | 1.52E-01 | No |
| *DNMT3A* | AB208833 | ENSG00000119772 | ae5 | alternative_last_exon | down | 1.71 | 2.95E-02 | up | 1.24 | 2.79E-01 | No |
| *RRN3P2* | BC042920 | ENSG00000103472 | e17 | alternative_last_exon | down | 1.71 | 4.57E-02 | up | 1.16 | 3.93E-01 | Yes |
| *ATP8B2* | BC140441 | ENSG00000143515 | e8 | Unknown | down | 1.71 | 2.97E-02 | down | 1.28 | 1.08E-03 | No |
| *ARHGAP8 // PRR5 // PRR5-ARHGAP8* | CR597658 | ENSG00000186654 // ENSG00000241484 // ENSG00000248405 | e3 | alternative_first_exon | down | 1.70 | 6.30E-03 | up | 1.39 | 2.31E-02 | Yes |
| *LAT // SPNS1* | AB209402 | ENSG00000169682 // ENSG00000213658 | e20 | Unknown | down | 1.70 | 1.21E-02 | up | 1.40 | 3.77E-02 | No |
| *FHL2* | U29332 | ENSG00000115641 | e4 | exon_skipping | down | 1.70 | 1.22E-02 | up | 1.49 | 8.37E-02 | Yes |
| *EXOSC10* | BC028687 | ENSG00000171824 | ae16 | intron_retention | down | 1.70 | 3.89E-02 | up | 1.24 | 9.05E-02 | Yes |
| *IFT74* | AK225570 | ENSG00000096872 | e7 | Unknown | down | 1.70 | 3.51E-03 | up | 1.35 | 2.22E-01 | No |
| *ZNF33A* | AK027057 | ENSG00000189180 | e5 | exon_skipping,alternative_last_exon | down | 1.70 | 1.82E-02 | up | 1.25 | 2.28E-01 | No |
| *SLC30A6* | AL832755 | ENSG00000152683 | e5 | exon_skipping | down | 1.70 | 1.26E-02 | up | 1.20 | 3.42E-01 | No |
| *VAV3* | AF067817 | ENSG00000134215 | e31 | exon_skipping | down | 1.70 | 1.31E-06 | up | 1.15 | 6.53E-01 | No |
| *YARS* | AK125213 | ENSG00000134684 | ae9 | intron_retention | down | 1.70 | 2.25E-05 | up | 1.07 | 7.56E-01 | Yes |
| *MUC3A* | AB038784 | ENSG00000169894 | e2 | Unknown | down | 1.70 | 3.13E-02 | down | 1.39 | 2.10E-02 | No |
| *SLC35F1* | BC028615 | ENSG00000196376 | ae10 | intron_retention | down | 1.70 | 7.16E-03 | down | 1.42 | 2.74E-02 | No |
| *F8* | AK295164 | ENSG00000185010 | e14 | exon_skipping | down | 1.70 | 1.69E-09 | down | 1.47 | 6.83E-02 | No |
| *FKBP1A // SDCBP2* | AK309026 | ENSG00000088832 // ENSG00000125775 | e12 | Unknown | down | 1.70 | 4.25E-08 | down | 1.30 | 2.19E-01 | No |
| *XAF1* | BX649188 | ENSG00000132530 | e5 | exon_skipping | down | 1.70 | 5.20E-04 | down | 1.02 | 9.36E-01 | No |
| *HIATL2* | BC005058 | ENSG00000196312 | e5 | exon_skipping,alternative_last_exon | down | 1.69 | 1.77E-04 | up | 1.41 | 4.84E-03 | Yes |
| *SPIRE2* | AL834408 | ENSG00000204991 | e3 | exon_skipping,alternative_last_exon | down | 1.69 | 5.55E-07 | up | 1.24 | 7.08E-03 | Yes |
| *NEDD8* | BC104664 | ENSG00000129559 | e3 | exon_skipping,alternative_last_exon | down | 1.69 | 1.87E-02 | up | 1.44 | 1.01E-02 | Yes |
| *TARBP2* | BC002419 | ENSG00000139546 | ae8 | alternative_acceptor_splice_site | down | 1.69 | 1.76E-02 | up | 1.41 | 2.43E-02 | No |
| *TMEM120B* | BC035884 | ENSG00000188735 | e15 | exon_skipping | down | 1.69 | 3.76E-02 | up | 1.35 | 5.11E-02 | No |
| *KLHL36* | BC033821 | ENSG00000135686 | e1 | alternative_first_exon | down | 1.69 | 1.87E-02 | up | 1.27 | 5.63E-02 | Yes |
| *C11orf73* | BC021621 | ENSG00000149196 | ae7 | intron_retention | down | 1.69 | 1.91E-02 | up | 1.31 | 8.20E-02 | No |
| *UTP14A* | AK298578 | ENSG00000156697 | e15 | alternative_last_exon | down | 1.69 | 3.04E-02 | up | 1.41 | 9.45E-02 | No |
| *MSH2* | AK223284 | ENSG00000095002 | e18 | alternative_last_exon | down | 1.69 | 1.54E-02 | up | 1.35 | 1.57E-01 | Yes |
| *NIPAL2* | AK024017 | ENSG00000104361 | e13 | exon_skipping | down | 1.69 | 1.70E-02 | up | 1.13 | 3.28E-01 | No |
| *---* | AK310526 | --- | e4 | Unknown | down | 1.69 | 9.72E-03 | down | 1.26 | 1.52E-02 | Yes |
| *CEP110* | BC137286 | ENSG00000119397 | e8 | Unknown | down | 1.68 | 6.80E-03 | up | 1.16 | 2.03E-02 | No |
| *UTP14A* | AK298578 | ENSG00000156697 | e7 | Unknown | down | 1.68 | 1.26E-03 | up | 1.48 | 2.75E-02 | Yes |
| *HSPBP1* | CR615211 | ENSG00000133265 | ae3 | alternative_first_exon | down | 1.68 | 3.19E-02 | up | 1.38 | 3.03E-02 | Yes |
| *HARS* | AK000498 | ENSG00000170445 | ae7 | intron_retention | down | 1.68 | 2.91E-02 | up | 1.39 | 3.76E-02 | Yes |
| *C3orf31* | AX747723 | ENSG00000144559 | e12 | exon_skipping,alternative_last_exon | down | 1.68 | 2.95E-02 | up | 1.68 | 7.04E-02 | Yes |
| *C9orf86 // KIAA1984* | AF318367 | ENSG00000196642 // ENSG00000213213 | e1 | alternative_first_exon | down | 1.68 | 2.89E-02 | up | 1.47 | 9.61E-02 | Yes |
| *CEP78* | BC128058 | ENSG00000148019 | ae16 | alternative_last_exon | down | 1.68 | 3.79E-02 | up | 1.41 | 1.43E-01 | No |
| *BRCA1* | AK293762 | ENSG00000012048 | e23 | exon_skipping | down | 1.68 | 1.48E-02 | up | 1.48 | 1.59E-01 | No |
| *IFT74* | AK225570 | ENSG00000096872 | e21 | alternative_last_exon | down | 1.68 | 6.52E-03 | up | 1.35 | 2.22E-01 | No |
| *CENPH* | BC015355 | ENSG00000153044 | e8 | Unknown | down | 1.68 | 9.32E-03 | up | 1.33 | 2.77E-01 | No |
| *EGFL8 // PPT2* | AF020544 | ENSG00000227600 // ENSG00000240389 // ENSG00000241404 | e21 | alternative_last_exon | down | 1.68 | 8.88E-03 | up | 1.28 | 2.94E-01 | No |
| *EPB41L5* | BC054508 | ENSG00000115109 | e19 | Unknown | down | 1.68 | 4.44E-02 | up | 1.13 | 6.48E-01 | No |
| *NBPF3* | AK095602 | ENSG00000142794 | e4 | exon_skipping | down | 1.68 | 6.80E-03 | up | 1.04 | 8.24E-01 | No |
| *MXRA5* | AF245505 | ENSG00000101825 | ae5 | intron_retention | down | 1.68 | 2.98E-03 | down | 1.86 | 1.04E-01 | No |
| *LCAT // SLC12A4* | BC021193 | ENSG00000124067 // ENSG00000213398 | e31 | Unknown | down | 1.68 | 1.28E-02 | down | 1.07 | 7.44E-01 | No |
| *TMC6* | BC023597 | ENSG00000141524 | ae13 | intron_retention | down | 1.67 | 1.69E-02 | up | 1.33 | 1.21E-02 | No |
| *MRPL9* | AK315459 | ENSG00000143436 | ae4 | intron_retention,alternative_last_exon | down | 1.67 | 2.18E-02 | up | 1.44 | 1.68E-02 | No |
| *ZBTB40* | AB007947 | ENSG00000184677 | e16 | Unknown | down | 1.67 | 1.06E-03 | up | 1.46 | 1.71E-02 | No |
| *INO80B // WBP1* | AK055333 | ENSG00000115274 // ENSG00000239779 | e1 | alternative_first_exon | down | 1.67 | 3.46E-03 | up | 1.30 | 2.81E-02 | Yes |
| *GBAS* | AF029786 | ENSG00000146729 | ae4 | alternative_first_exon | down | 1.67 | 2.65E-06 | up | 1.55 | 5.46E-02 | Yes |
| *CACNA2D2* | AF042793 | ENSG00000007402 | e31 | Unknown | down | 1.67 | 2.37E-02 | up | 1.73 | 1.05E-01 | Yes |
| *ACACA* | BC137287 | ENSG00000132142 | e3 | exon_skipping | down | 1.67 | 4.37E-02 | up | 1.31 | 1.62E-01 | No |
| *PPP1R9A* | AB033048 | ENSG00000158528 | e8 | Unknown | down | 1.67 | 1.03E-04 | up | 1.28 | 2.39E-01 | Yes |
| *THADA* | AK290023 | ENSG00000115970 | e29 | exon_skipping | down | 1.67 | 3.88E-02 | up | 1.25 | 3.00E-01 | Yes |
| *CDADC1* | AY027525 | ENSG00000102543 | e5 | exon_skipping | down | 1.67 | 4.78E-03 | up | 1.19 | 3.38E-01 | No |
| *C5orf42* | BC150594 | ENSG00000197603 | e28 | exon_skipping | down | 1.67 | 1.29E-02 | up | 1.06 | 8.17E-01 | No |
| *SCN7A* | M91556 | ENSG00000136546 | e15 | Unknown | down | 1.67 | 1.48E-03 | down | 2.73 | 1.42E-01 | No |
| *NBPF9* | AF419616 | ENSG00000168614 | e5 | exon_skipping | down | 1.67 | 2.31E-02 | down | 1.19 | 4.18E-01 | No |
| *FIBP* | CR622949 | ENSG00000172500 | ae3 | alternative_last_exon | down | 1.66 | 3.98E-03 | up | 1.43 | 2.47E-02 | Yes |
| *---* | BC067758 | --- | e8 | alternative_last_exon | down | 1.66 | 1.70E-02 | up | 1.41 | 3.13E-02 | Yes |
| *TBCE* | CR599260 | ENSG00000116957 | e8 | exon_skipping | down | 1.66 | 2.65E-02 | up | 1.41 | 5.10E-02 | No |
| *IDH3B* | BC001960 | ENSG00000101365 | ae6 | intron_retention | down | 1.66 | 3.37E-02 | up | 1.36 | 8.43E-02 | Yes |
| *BICD1* | BC136372 | ENSG00000151746 | e1 | alternative_first_exon | down | 1.66 | 7.76E-03 | up | 1.28 | 1.66E-01 | Yes |
| *SURF6* | BC006197 | ENSG00000148296 | e2 | Unknown | down | 1.66 | 1.34E-02 | up | 1.27 | 2.01E-01 | Yes |
| *DOCK7* | DQ341187 | ENSG00000116641 | e16 | exon_skipping,alternative_last_exon | down | 1.66 | 2.69E-02 | up | 1.07 | 7.62E-01 | Yes |
| *ALG13* | AK316522 | ENSG00000101901 | e14 | Unknown | down | 1.66 | 1.38E-02 | up | 1.06 | 7.85E-01 | No |
| *CD209* | AK293089 | ENSG00000090659 | e2 | exon_skipping | down | 1.66 | 2.69E-02 | down | 1.48 | 6.20E-02 | No |
| *PHF20L1* | BC144654 | ENSG00000129292 | ae9 | intron_retention | down | 1.65 | 4.21E-02 | up | 1.45 | 7.47E-02 | Yes |
| *SCLT1* | AK055217 | ENSG00000151466 | e8 | exon_skipping | down | 1.65 | 2.13E-02 | up | 1.51 | 7.71E-02 | No |
| *KIF23* | BC051826 | ENSG00000137807 | e21 | exon_skipping | down | 1.65 | 3.86E-02 | up | 1.51 | 1.17E-01 | Yes |
| *OGG1* | U88620 | ENSG00000114026 | ae8 | alternative_acceptor_splice_site | down | 1.65 | 2.54E-02 | up | 1.30 | 1.35E-01 | Yes |
| *FBXW8* | AK289621 | ENSG00000174989 | e11 | alternative_last_exon | down | 1.65 | 6.00E-04 | up | 1.26 | 1.60E-01 | Yes |
| *KRT6B* | BC034535 | ENSG00000185479 | e3 | exon_skipping | down | 1.65 | 2.08E-02 | up | 1.78 | 2.04E-01 | Yes |
| *MAP2K5* | CR592618 | ENSG00000137764 | e12 | exon_skipping | down | 1.65 | 2.23E-02 | up | 1.23 | 3.88E-01 | No |
| *RRN3P1* | BC068999 | --- | e2 | Unknown | down | 1.65 | 2.22E-02 | up | 1.17 | 5.12E-01 | Yes |
| *EVC* | AK025394 | ENSG00000072840 | e22 | alternative_last_exon | down | 1.65 | 1.72E-02 | down | 1.40 | 4.57E-02 | No |
| *NT5C2* | D38524 | ENSG00000076685 | e12 | exon_skipping | down | 1.64 | 1.22E-02 | up | 1.32 | 4.38E-03 | No |
| *ZBTB40* | AB007947 | ENSG00000184677 | e14 | Unknown | down | 1.64 | 3.56E-04 | up | 1.46 | 1.71E-02 | No |
| *B4GALT7* | AF142675 | ENSG00000027847 | ae4 | alternative_first_exon | down | 1.64 | 2.19E-02 | up | 1.32 | 8.20E-02 | Yes |
| *SLC25A15* | AF112968 | ENSG00000102743 | ae7 | alternative_last_exon | down | 1.64 | 4.42E-02 | up | 1.48 | 8.84E-02 | Yes |
| *TULP4* | AF219946 | ENSG00000130338 | e1 | alternative_first_exon | down | 1.64 | 4.97E-04 | up | 1.25 | 2.03E-01 | Yes |
| *GJA9 // MYCBP // RRAGC* | BC044622 | ENSG00000116954 // ENSG00000131233 // ENSG00000214114 | e1 | alternative_first_exon | down | 1.64 | 9.57E-07 | up | 1.18 | 3.27E-01 | Yes |
| *ZNF41* | AK294858 | ENSG00000147124 | e2 | exon_skipping,alternative_first_exon | down | 1.64 | 3.47E-02 | up | 1.11 | 3.55E-01 | Yes |
| *EMCN* | AK291831 | ENSG00000164035 | e1 | alternative_first_exon | down | 1.64 | 7.94E-03 | down | 1.67 | 8.45E-02 | No |
| *CRYBA2* | BC006285 | ENSG00000163499 | e4 | Unknown | down | 1.64 | 4.78E-02 | down | 1.39 | 1.21E-01 | No |
| *HMGA2* | X92518 | ENSG00000149948 | e5 | exon_skipping,alternative_last_exon | down | 1.64 | 4.88E-02 | down | 1.24 | 3.61E-01 | Yes |
| *PHKG2* | AK293551 | ENSG00000156873 | e2 | Unknown | down | 1.63 | 1.21E-02 | up | 1.34 | 4.33E-02 | No |
| *PAK1IP1* | AK225516 | ENSG00000111845 | e9 | Unknown | down | 1.63 | 2.69E-02 | up | 1.62 | 6.98E-02 | Yes |
| *ZNF280C* | BC051728 | ENSG00000056277 | e15 | Unknown | down | 1.63 | 1.04E-02 | up | 1.51 | 8.20E-02 | Yes |
| *MIR17HG* | AB176708 | ENSG00000215417 | ae2 | intron_retention | down | 1.63 | 3.54E-02 | up | 1.84 | 1.51E-01 | Yes |
| *BRCA1* | AK293762 | ENSG00000012048 | e8 | exon_skipping,exon_skipping,exon_skipping | down | 1.63 | 1.83E-02 | up | 1.48 | 1.59E-01 | Yes |
| *DUS3L* | EU832718 | ENSG00000141994 | e9 | Unknown | down | 1.63 | 1.86E-02 | up | 1.14 | 2.40E-01 | Yes |
| *TIMP1* | DQ894301 | ENSG00000102265 | ae3 | intron_retention | down | 1.63 | 4.55E-02 | up | 1.36 | 2.57E-01 | No |
| *ATP8B2* | BC140441 | ENSG00000143515 | e26 | Unknown | down | 1.63 | 8.01E-07 | down | 1.28 | 1.08E-03 | No |
| *TTN* | AF321609 | ENSG00000155657 | e166 | exon_skipping | down | 1.63 | 4.77E-02 | down | 1.26 | 2.10E-03 | Yes |
| *ITSN1* | AF064244 | ENSG00000205726 | e35 | Unknown | down | 1.63 | 8.76E-03 | down | 1.23 | 3.00E-01 | No |
| *ERCC2* | BC110523 | ENSG00000104884 | e13 | alternative_first_exon,exon_skipping | down | 1.62 | 1.67E-02 | up | 1.45 | 2.40E-02 | Yes |
| *FARSB* | D84430 | ENSG00000116120 | e3 | exon_skipping | down | 1.62 | 4.03E-02 | up | 1.34 | 1.96E-01 | No |
| *FAM190B* | AK298966 | ENSG00000107771 | e12 | exon_skipping | down | 1.62 | 3.13E-02 | up | 1.16 | 3.19E-01 | No |
| *LRBA* | AF216648 | ENSG00000198589 | e48 | Unknown | down | 1.62 | 7.16E-04 | up | 1.14 | 4.51E-01 | No |
| *UBE2E2* | AK307075 | ENSG00000182247 | e3 | Unknown | down | 1.62 | 5.60E-03 | down | 1.85 | 6.67E-02 | No |
| *BAZ2A* | CR749379 | ENSG00000076108 | e1 | alternative_first_exon | down | 1.62 | 2.38E-02 | down | 1.11 | 4.76E-01 | No |
| *HAVCR1* | AK298259 | ENSG00000113249 | e5 | Unknown | down | 1.62 | 4.96E-03 | down | 1.04 | 9.63E-01 | No |
| *AP2B1* | M34175 | ENSG00000006125 | e1 | alternative_first_exon | down | 1.61 | 3.04E-03 | up | 1.41 | 7.76E-03 | Yes |
| *ZBTB40* | AB007947 | ENSG00000184677 | e1 | alternative_first_exon | down | 1.61 | 2.15E-02 | up | 1.46 | 1.71E-02 | No |
| *MARK3* | AF465413 | ENSG00000075413 | e3 | Unknown | down | 1.61 | 3.89E-02 | up | 1.32 | 7.06E-02 | Yes |
| *MTIF2* | AF494407 | ENSG00000085760 | e14 | Unknown | down | 1.61 | 1.77E-02 | up | 1.49 | 7.88E-02 | Yes |
| *ANKLE1 // C19orf62* | AK299493 | ENSG00000105393 // ENSG00000160117 | ae16 | alternative_donor_splice_site | down | 1.61 | 3.12E-02 | up | 1.26 | 1.01E-01 | No |
| *SNRPA1* | BC067846 | ENSG00000131876 | ae6 | intron_retention | down | 1.61 | 4.71E-02 | up | 1.32 | 1.81E-01 | Yes |
| *RRP15* | BC020641 | ENSG00000067533 | e4 | Unknown | down | 1.61 | 4.23E-04 | up | 1.28 | 1.96E-01 | Yes |
| *C19orf63* | AY194293 | ENSG00000161671 | e11 | exon_skipping | down | 1.61 | 1.04E-02 | up | 1.25 | 2.09E-01 | Yes |
| *PION* | BC110797 | ENSG00000186088 | ae20 | intron_retention,alternative_last_exon | down | 1.61 | 4.86E-03 | up | 1.31 | 2.86E-01 | No |
| *KLHL18* | BX537953 | ENSG00000114648 | ae8 | alternative_last_exon | down | 1.61 | 1.18E-02 | up | 1.19 | 2.92E-01 | No |
| *SLC39A13* | BX647491 | ENSG00000165915 | ae1 | alternative_donor_splice_site | down | 1.61 | 6.45E-08 | up | 1.19 | 3.82E-01 | Yes |
| *PEX16* | CR612893 | ENSG00000121680 | e10 | Unknown | down | 1.61 | 2.14E-03 | up | 1.08 | 4.39E-01 | No |
| *FIP1L1 // PDGFRA* | AY229892 | ENSG00000134853 // ENSG00000145216 | e25 | exon_skipping | down | 1.61 | 8.35E-05 | up | 1.03 | 8.73E-01 | No |
| *KLHL5* | BC053860 | ENSG00000109790 | e3 | exon_skipping | down | 1.61 | 1.52E-02 | up | 1.06 | 9.02E-01 | No |
| *PLXNC1* | AF030339 | ENSG00000136040 | e10 | Unknown | down | 1.61 | 7.38E-03 | down | 1.84 | 9.59E-02 | No |
| *FKBP1A // SDCBP2* | AK309026 | ENSG00000088832 // ENSG00000125775 | e14 | Unknown | down | 1.61 | 7.92E-07 | down | 1.34 | 1.56E-01 | Yes |
| *CALCRL* | L76380 | ENSG00000064989 | e4 | Unknown | down | 1.61 | 1.76E-05 | down | 1.76 | 1.90E-01 | No |
| *XRN1* | BX640905 | ENSG00000114127 | ae24 | alternative_last_exon | down | 1.61 | 1.77E-02 | down | 1.15 | 5.64E-01 | No |
| *ELK4 // SLC45A3* | FJ805261 | ENSG00000158711 // ENSG00000158715 | e16 | alternative_last_exon | down | 1.61 | 4.33E-02 | down | 1.05 | 6.76E-01 | No |
| *MACF1* | AB029290 | ENSG00000127603 | e40 | exon_skipping,alternative_first_exon | down | 1.61 | 2.60E-02 | down | 1.06 | 8.09E-01 | No |
| *GPC3* | U50410 | ENSG00000147257 | e5 | Unknown | down | 1.61 | 6.92E-11 | down | 1.01 | 9.82E-01 | No |
| *SMAGP* | BC045658 | ENSG00000170545 | e2 | exon_skipping,alternative_first_exon | down | 1.60 | 4.93E-02 | up | 1.47 | 3.95E-07 | Yes |
| *GIGYF1* | AY176044 | ENSG00000146830 | ae2 | intron_retention,alternative_first_exon | down | 1.60 | 2.43E-04 | up | 1.20 | 4.54E-03 | No |
| *NUP85* | AF514995 | ENSG00000125450 | e13 | exon_skipping,alternative_first_exon | down | 1.60 | 2.88E-02 | up | 1.38 | 4.64E-02 | Yes |
| *FGGY* | BC014947 | ENSG00000172456 | e6 | exon_skipping | down | 1.60 | 7.96E-05 | up | 2.26 | 5.03E-02 | No |
| *DHPS* | AK300498 | ENSG00000095059 | e2 | exon_skipping,alternative_first_exon | down | 1.60 | 2.48E-02 | up | 1.32 | 7.53E-02 | Yes |
| *TRMT5* | BC136607 | ENSG00000126814 | e5 | alternative_last_exon | down | 1.60 | 2.58E-02 | up | 1.52 | 7.74E-02 | No |
| *SULT2B1* | U92315 | ENSG00000088002 | e8 | alternative_last_exon | down | 1.60 | 3.41E-02 | up | 1.99 | 8.52E-02 | No |
| *C1orf175 // TTC4* | CR749830 | ENSG00000184313 // ENSG00000243725 | e27 | Unknown | down | 1.60 | 4.88E-02 | up | 1.24 | 1.49E-01 | No |
| *---* | BC157846 | --- | e34 | alternative_last_exon | down | 1.60 | 5.48E-03 | up | 1.38 | 1.67E-01 | Yes |
| *HPS1* | U65676 | ENSG00000107521 | e4 | exon_skipping,alternative_last_exon | down | 1.60 | 3.93E-02 | up | 1.13 | 1.85E-01 | Yes |
| *HELQ* | AF436845 | ENSG00000163312 | e17 | Unknown | down | 1.60 | 2.69E-02 | up | 1.17 | 3.89E-01 | No |
| *OSGEP* | CR597694 | ENSG00000092094 | ae5 | alternative_donor_splice_site | down | 1.60 | 8.06E-03 | up | 1.11 | 4.31E-01 | No |
| *AKT3* | AY005799 | ENSG00000117020 | e10 | Unknown | down | 1.60 | 1.16E-02 | down | 1.88 | 8.00E-02 | No |
| *---* | AK092605 | --- | e3 | alternative_last_exon | down | 1.60 | 1.97E-02 | down | 1.11 | 5.61E-01 | No |
| *PSMG4* | AK095683 | ENSG00000180822 | e3 | alternative_last_exon | down | 1.59 | 4.37E-02 | up | 1.32 | 1.86E-05 | No |
| *PIGO* | AK074064 | ENSG00000165282 | e2 | exon_skipping | down | 1.59 | 3.65E-02 | up | 1.34 | 3.35E-02 | No |
| *ATR* | Y09077 | ENSG00000175054 | e41 | Unknown | down | 1.59 | 4.54E-03 | up | 1.43 | 1.36E-01 | Yes |
| *KIAA1841* | BC070104 | ENSG00000162929 | e22 | exon_skipping | down | 1.59 | 1.45E-02 | up | 1.31 | 1.50E-01 | No |
| *LRWD1* | AL133057 | ENSG00000161036 | e14 | Unknown | down | 1.59 | 2.25E-02 | up | 1.24 | 1.54E-01 | Yes |
| *PSTPIP2* | BC035395 | ENSG00000152229 | e13 | Unknown | down | 1.59 | 2.27E-02 | up | 1.49 | 1.87E-01 | Yes |
| *USP45* | BC157838 | ENSG00000123552 | e2 | Unknown | down | 1.59 | 1.73E-02 | up | 1.28 | 3.54E-01 | No |
| *LMNA* | AK056143 | ENSG00000160789 | e7 | exon_skipping | down | 1.59 | 2.68E-02 | up | 1.02 | 8.94E-01 | No |
| *XDH* | D11456 | ENSG00000158125 | e1 | alternative_first_exon | down | 1.59 | 5.02E-03 | down | 1.40 | 2.21E-01 | No |
| *SH2D3C* | AK096983 | ENSG00000095370 | e6 | exon_skipping | down | 1.59 | 4.70E-02 | down | 1.28 | 4.15E-01 | No |
| *OSBPL9* | AK128043 | ENSG00000117859 | e1 | alternative_first_exon | down | 1.59 | 3.34E-02 | down | 1.01 | 9.63E-01 | Yes |
| *C15orf63 // SERF2* | BC009869 | ENSG00000140264 // ENSG00000242028 | e7 | exon_skipping,alternative_first_exon | down | 1.59 | 4.36E-02 | down | 1.00 | 9.94E-01 | No |
| *TBC1D17* | BC003516 | ENSG00000104946 | ae18 | alternative_acceptor_splice_site | down | 1.58 | 2.00E-02 | up | 1.31 | 3.21E-02 | Yes |
| *CHMP4A // IPO4 // MDP1 // TM9SF1* | AK300118 | ENSG00000100926 // ENSG00000100931 // ENSG00000196497 // ENSG00000213920 | e8 | Unknown | down | 1.58 | 2.64E-02 | up | 1.48 | 7.70E-03 | No |
| *DNTTIP2* | AY336729 | ENSG00000067334 | e5 | Unknown | down | 1.58 | 5.32E-03 | up | 1.58 | 6.56E-02 | Yes |
| *ERCC4* | AK289726 | ENSG00000175595 | e10 | Unknown | down | 1.58 | 2.59E-02 | up | 1.32 | 7.85E-02 | Yes |
| *---* | BC028086 | --- | e2 | alternative_first_exon | down | 1.58 | 3.28E-02 | up | 1.60 | 8.18E-02 | Yes |
| *CACNA2D2* | AF042793 | ENSG00000007402 | e40 | Unknown | down | 1.58 | 4.06E-02 | up | 1.73 | 1.05E-01 | Yes |
| *MTHFD1L* | AY374130 | ENSG00000120254 | e16 | Unknown | down | 1.58 | 1.83E-02 | up | 1.55 | 1.13E-01 | No |
| *AUP1* | EU831868 | ENSG00000115307 | ae12 | alternative_last_exon | down | 1.58 | 3.91E-02 | up | 1.21 | 2.21E-01 | No |
| *EXOG* | AB020735 | ENSG00000157036 | e12 | alternative_last_exon | down | 1.58 | 3.70E-03 | up | 1.23 | 3.18E-01 | Yes |
| *BCAS3* | AK225757 | ENSG00000141376 | e26 | exon_skipping,alternative_first_exon | down | 1.58 | 4.31E-02 | up | 1.17 | 4.20E-01 | No |
| *ZDHHC21* | AF161360 | ENSG00000175893 | ae8 | alternative_first_exon | down | 1.58 | 3.61E-06 | up | 1.08 | 7.34E-01 | No |
| *CCNH* | BX537673 | ENSG00000134480 | e10 | alternative_last_exon | down | 1.58 | 2.59E-02 | up | 1.06 | 7.95E-01 | No |
| *GNB5* | AF300650 | ENSG00000069966 | e3 | Unknown | down | 1.58 | 6.86E-03 | down | 1.45 | 2.63E-02 | No |
| *CHST5* | BC063677 | ENSG00000135702 | e9 | Unknown | down | 1.58 | 1.25E-04 | down | 1.49 | 1.64E-01 | No |
| *LDB2* | AF052389 | ENSG00000169744 | e2 | Unknown | down | 1.58 | 2.11E-02 | down | 1.26 | 3.66E-01 | No |
| *WSB1* | AL110269 | ENSG00000109046 | e6 | exon_skipping | down | 1.58 | 7.32E-03 | down | 1.10 | 6.71E-01 | No |
| *MACF1* | AB029290 | ENSG00000127603 | e27 | Unknown | down | 1.58 | 1.28E-02 | down | 1.06 | 8.09E-01 | No |
| *XAF1* | BX649188 | ENSG00000132530 | e6 | exon_skipping | down | 1.58 | 1.52E-02 | down | 1.02 | 9.36E-01 | No |
| *MTMR15* | AL833089 | ENSG00000198690 | e12 | Unknown | down | 1.57 | 1.59E-02 | up | 1.35 | 3.73E-02 | No |
| *TMEM120B* | BC035884 | ENSG00000188735 | e10 | exon_skipping | down | 1.57 | 1.17E-07 | up | 1.35 | 5.00E-02 | Yes |
| *ABHD2* | AF546700 | ENSG00000140526 | e1 | alternative_first_exon | down | 1.57 | 2.70E-03 | up | 1.69 | 6.09E-02 | Yes |
| *ZCCHC4* | AY629351 | ENSG00000168228 | e1 | alternative_first_exon | down | 1.57 | 2.32E-02 | up | 1.49 | 7.09E-02 | Yes |
| *STRBP* | AL136866 | ENSG00000165209 | e14 | Unknown | down | 1.57 | 2.98E-03 | up | 1.44 | 1.15E-01 | Yes |
| *ABLIM2* | AK094754 | ENSG00000163995 | e23 | exon_skipping,alternative_last_exon | down | 1.57 | 3.44E-10 | up | 1.50 | 1.59E-01 | Yes |
| *COX7A2L* | AK092911 | ENSG00000115944 | e1 | alternative_first_exon | down | 1.57 | 3.25E-02 | up | 1.19 | 2.59E-01 | Yes |
| *FANCD2* | BC038666 | ENSG00000144554 | e10 | Unknown | down | 1.57 | 1.00E-04 | up | 1.36 | 2.70E-01 | Yes |
| *IL1RAP* | EF591790 | ENSG00000196083 | e13 | alternative_last_exon,exon_skipping | down | 1.57 | 2.22E-02 | up | 1.29 | 4.36E-01 | No |
| *YIPF3* | CR590752 | ENSG00000137207 | e1 | alternative_first_exon | down | 1.57 | 2.97E-02 | up | 1.17 | 4.45E-01 | No |
| *HDAC4* | AB006626 | ENSG00000068024 | e26 | Unknown | down | 1.57 | 1.96E-02 | up | 1.10 | 4.63E-01 | No |
| *MKNK1 // MOBKL2C* | AK096423 | ENSG00000079277 // ENSG00000142961 | e8 | exon_skipping | down | 1.57 | 8.90E-03 | up | 1.09 | 5.59E-01 | No |
| *GPR126* | BC075798 | ENSG00000112414 | e24 | exon_skipping | down | 1.57 | 1.76E-05 | up | 1.22 | 5.74E-01 | No |
| *CEP135* | BC136536 | ENSG00000174799 | e2 | Unknown | down | 1.57 | 1.11E-02 | up | 1.02 | 9.39E-01 | No |
| *PSG6 // PSG8* | CR604837 | ENSG00000124467 // ENSG00000170848 | e3 | alternative_last_exon | down | 1.57 | 2.84E-02 | down | 1.09 | 2.77E-01 | Yes |
| *SCUBE2* | AK131552 | ENSG00000175356 | e10 | Unknown | down | 1.57 | 4.52E-02 | down | 1.39 | 4.68E-01 | No |
| *AKAP8L* | AK295956 | ENSG00000011243 | e10 | exon_skipping | down | 1.56 | 8.38E-03 | up | 1.22 | 4.10E-05 | No |
| *ARHGAP8 // PRR5 // PRR5-ARHGAP8* | CR597658 | ENSG00000186654 // ENSG00000241484 // ENSG00000248405 | e16 | exon_skipping,alternative_last_exon | down | 1.56 | 2.98E-02 | up | 1.43 | 1.37E-02 | No |
| *FPGS* | BC064393 | ENSG00000136877 | e12 | Unknown | down | 1.56 | 8.48E-03 | up | 1.47 | 1.54E-02 | Yes |
| *---* | AK094715 | --- | e6 | Unknown | down | 1.56 | 2.51E-08 | up | 2.02 | 1.71E-01 | No |
| *NEU1 // SLC44A4* | AF466766 | ENSG00000204385 // ENSG00000204386 // ENSG00000227129 // ENSG00000228263 | e11 | Unknown | down | 1.56 | 6.44E-05 | up | 1.51 | 1.83E-01 | Yes |
| *CREB3* | AF211848 | ENSG00000107175 | ae2 | intron_retention | down | 1.56 | 3.65E-02 | up | 1.22 | 1.84E-01 | No |
| *C14orf118* | AK000696 | ENSG00000089916 | e1 | alternative_first_exon | down | 1.56 | 2.89E-02 | up | 1.16 | 2.44E-01 | No |
| *PRUNE2* | AB535152 | ENSG00000106772 | ae21 | intron_retention | down | 1.56 | 4.58E-02 | up | 1.36 | 3.57E-01 | Yes |
| *FUT4 // PIWIL4* | CR749642 | ENSG00000134627 // ENSG00000196371 | e15 | exon_skipping | down | 1.56 | 4.92E-03 | up | 1.19 | 4.52E-01 | Yes |
| *ZBED5* | AK126323 | ENSG00000236287 | e5 | Unknown | down | 1.56 | 4.15E-02 | up | 1.01 | 9.78E-01 | Yes |
| *TTN* | AF321609 | ENSG00000155657 | e174 | exon_skipping | down | 1.56 | 4.15E-02 | down | 1.26 | 2.10E-03 | Yes |
| *F8* | AK295164 | ENSG00000185010 | e13 | exon_skipping | down | 1.56 | 2.81E-02 | down | 1.47 | 6.83E-02 | No |
| *ERG* | M21535 | ENSG00000157554 | e3 | exon_skipping | down | 1.56 | 1.99E-02 | down | 1.44 | 1.96E-01 | No |
| *GATM* | AK098393 | ENSG00000171766 | e2 | alternative_first_exon | down | 1.56 | 2.17E-05 | down | 1.26 | 6.21E-01 | No |
| *BCL2L13* | AL831982 | ENSG00000099968 | ae10 | alternative_last_exon | down | 1.55 | 3.46E-02 | up | 1.47 | 7.30E-03 | Yes |
| *TBC1D17* | BC003516 | ENSG00000104946 | ae3 | intron_retention | down | 1.55 | 1.33E-06 | up | 1.28 | 7.78E-02 | No |
| *ZDHHC24* | CR616301 | ENSG00000174165 | e7 | alternative_last_exon | down | 1.55 | 3.17E-02 | up | 1.26 | 1.36E-01 | No |
| *---* | AB002330 | --- | e1 | alternative_first_exon | down | 1.55 | 1.80E-02 | up | 1.40 | 2.14E-01 | Yes |
| *C6orf26 // MSH5* | AJ245661 | ENSG00000204410 // ENSG00000227861 // ENSG00000228727 | e34 | alternative_last_exon | down | 1.55 | 9.80E-03 | up | 1.47 | 2.29E-01 | Yes |
| *KIAA0284* | BC112928 | ENSG00000099814 | ae19 | intron_retention | down | 1.55 | 1.96E-03 | up | 1.12 | 2.33E-01 | No |
| *TFDP1* | BC011685 | ENSG00000198176 | e4 | exon_skipping,alternative_last_exon | down | 1.55 | 3.12E-02 | up | 1.18 | 2.58E-01 | No |
| *EGFL8 // PPT2* | AF020544 | ENSG00000227600 // ENSG00000240389 // ENSG00000241404 | e20 | Unknown | down | 1.55 | 2.87E-02 | up | 1.28 | 2.94E-01 | No |
| *KIF14* | D26361 | ENSG00000118193 | e20 | Unknown | down | 1.55 | 2.45E-02 | up | 1.25 | 3.35E-01 | Yes |
| *RANBP17* | AJ288955 | ENSG00000204764 | e2 | Unknown | down | 1.55 | 8.35E-11 | up | 1.26 | 3.53E-01 | No |
| *PVRIG // STAG3* | AJ007798 | ENSG00000066923 // ENSG00000213413 | e38 | Unknown | down | 1.55 | 3.32E-02 | up | 1.06 | 5.60E-01 | Yes |
| *CCDC132* | AL833112 | ENSG00000004766 | e21 | Unknown | down | 1.55 | 1.27E-02 | up | 1.14 | 5.98E-01 | No |
| *C8orf76 // ZHX1* | BC012379 | ENSG00000165156 // ENSG00000189376 | e2 | alternative_first_exon,exon_skipping | down | 1.55 | 2.60E-02 | up | 1.15 | 7.45E-01 | No |
| *TRA@ // TRAC // TRD@* | FJ009444 | ENSG00000211889 // ENSG00000229164 | e128 | exon_skipping,alternative_last_exon | down | 1.55 | 2.72E-03 | down | 1.32 | 6.11E-02 | No |
| *TLR10* | AY358300 | ENSG00000174123 | e2 | exon_skipping,alternative_first_exon | down | 1.55 | 2.25E-02 | down | 1.19 | 4.11E-01 | No |
| *SMARCA2* | BC068252 | ENSG00000080503 | e1 | alternative_first_exon | down | 1.55 | 8.70E-03 | down | 1.01 | 9.58E-01 | No |
| *CLDN15 // FIS1* | AK310968 | ENSG00000106404 // ENSG00000214253 | e8 | alternative_first_exon | down | 1.54 | 2.86E-03 | up | 1.18 | 7.28E-03 | No |
| *---* | BC067758 | --- | e4 | exon_skipping | down | 1.54 | 1.76E-02 | up | 1.41 | 3.13E-02 | Yes |
| *PMS2* | BX537558 | ENSG00000122512 | e15 | exon_skipping | down | 1.54 | 1.52E-02 | up | 1.54 | 5.51E-02 | Yes |
| *TRAF5* | AB000509 | ENSG00000082512 | ae6 | intron_retention,alternative_acceptor_splice_site | down | 1.54 | 1.87E-06 | up | 1.72 | 5.64E-02 | Yes |
| *LARP1B* | AL137759 | ENSG00000138709 | ae22 | alternative_last_exon | down | 1.54 | 5.94E-03 | up | 1.57 | 5.86E-02 | Yes |
| *RALGAPA2* | BC171842 | ENSG00000188559 | e3 | exon_skipping | down | 1.54 | 3.63E-10 | up | 1.43 | 1.14E-01 | Yes |
| *FAM111A* | BC071759 | ENSG00000166801 | ae3 | intron_retention,alternative_first_exon | down | 1.54 | 2.28E-02 | up | 1.41 | 1.35E-01 | Yes |
| *---* | BC157846 | --- | e5 | Unknown | down | 1.54 | 9.10E-03 | up | 1.38 | 1.67E-01 | No |
| *GSTK1* | AK095263 | ENSG00000197448 | e9 | alternative_last_exon | down | 1.54 | 1.22E-02 | up | 1.24 | 1.89E-01 | No |
| *KIF4A* | AF071592 | ENSG00000090889 | e33 | Unknown | down | 1.54 | 1.64E-03 | up | 1.32 | 2.11E-01 | Yes |
| *EDN1* | CR602522 | ENSG00000078401 | e2 | alternative_last_exon | down | 1.54 | 5.86E-05 | up | 1.75 | 2.18E-01 | No |
| *CCDC94* | AK001236 | ENSG00000105248 | e7 | Unknown | down | 1.54 | 5.26E-03 | up | 1.12 | 3.36E-01 | No |
| *MCCC1* | BC036395 | ENSG00000078070 | e22 | alternative_last_exon | down | 1.54 | 2.94E-03 | up | 1.19 | 3.77E-01 | Yes |
| *---* | AK095188 | --- | e2 | Unknown | down | 1.54 | 8.70E-03 | up | 1.28 | 3.78E-01 | Yes |
| *UBR4* | AF348492 | ENSG00000127481 | e36 | Unknown | down | 1.54 | 4.28E-02 | up | 1.15 | 5.66E-01 | No |
| *SERPINB6* | AK094983 | ENSG00000124570 | e3 | exon_skipping,alternative_first_exon | down | 1.54 | 4.20E-04 | up | 1.14 | 7.13E-01 | No |
| *LTBP1* | AB208801 | ENSG00000049323 | e1 | alternative_first_exon | down | 1.54 | 1.99E-02 | up | 1.08 | 7.48E-01 | No |
| *STRN3* | U17989 | ENSG00000196792 | e2 | Unknown | down | 1.54 | 1.50E-02 | up | 1.04 | 7.96E-01 | No |
| *LRP4* | AB084910 | ENSG00000134569 | e22 | Unknown | down | 1.54 | 3.80E-03 | up | 1.09 | 8.21E-01 | No |
| *FCGBP* | D84239 | ENSG00000090920 | e35 | Unknown | down | 1.54 | 7.18E-03 | down | 1.48 | 5.40E-03 | Yes |
| *FBXL7* | AB020647 | ENSG00000183580 | e5 | Unknown | down | 1.54 | 1.28E-02 | down | 1.56 | 5.18E-02 | No |
| *SCAMP5* | AK126940 | ENSG00000198794 | e8 | Unknown | down | 1.54 | 1.32E-02 | down | 1.95 | 7.93E-02 | No |
| *ACSS3* | BC015769 | ENSG00000111058 | e4 | Unknown | down | 1.54 | 1.73E-02 | down | 1.30 | 1.55E-01 | No |
| *DAB2IP* | AK096391 | ENSG00000136848 | e3 | Unknown | down | 1.53 | 1.04E-03 | up | 1.19 | 3.51E-05 | No |
| *HSPBP1* | CR615211 | ENSG00000133265 | e8 | exon_skipping | down | 1.53 | 2.49E-02 | up | 1.38 | 3.03E-02 | Yes |
| *AIFM3 // LZTR1* | AK127080 | ENSG00000099949 // ENSG00000183773 | e32 | Unknown | down | 1.53 | 6.86E-03 | up | 1.24 | 3.60E-02 | Yes |
| *BOK* | BC017214 | ENSG00000176720 | e2 | Unknown | down | 1.53 | 2.89E-02 | up | 1.45 | 5.11E-02 | No |
| *BRE* | AK000097 | ENSG00000158019 | e13 | exon_skipping | down | 1.53 | 4.82E-02 | up | 1.51 | 6.50E-02 | Yes |
| *ZC3H3* | BC038670 | ENSG00000014164 | e3 | Unknown | down | 1.53 | 1.18E-02 | up | 1.22 | 6.52E-02 | Yes |
| *SNX11* | AK091852 | ENSG00000002919 | e4 | exon_skipping | down | 1.53 | 3.57E-04 | up | 1.23 | 1.10E-01 | No |
| *C2orf3* | EF158468 | ENSG00000005436 | e14 | Unknown | down | 1.53 | 8.54E-03 | up | 1.46 | 1.85E-01 | Yes |
| *EIF2B5* | AK310207 | ENSG00000145191 | e6 | exon_skipping | down | 1.53 | 2.44E-02 | up | 1.17 | 1.91E-01 | No |
| *LRRC23* | CR590063 | ENSG00000010626 | e12 | alternative_last_exon | down | 1.53 | 1.41E-02 | up | 1.17 | 3.34E-01 | No |
| *KBTBD6* | CR625002 | ENSG00000165572 | e1 | alternative_first_exon | down | 1.53 | 7.72E-03 | up | 1.11 | 4.36E-01 | Yes |
| *ZNF510* | AB023189 | ENSG00000081386 | e3 | Unknown | down | 1.53 | 2.84E-02 | up | 1.14 | 4.79E-01 | No |
| *STXBP4* | AK122865 | ENSG00000166263 | e10 | Unknown | down | 1.53 | 1.67E-02 | up | 1.06 | 7.50E-01 | No |
| *EXOC3* | AK074086 | ENSG00000180104 | e16 | Unknown | down | 1.53 | 4.36E-02 | up | 1.02 | 7.90E-01 | No |
| *PARD3B* | AX765687 | ENSG00000116117 | e12 | Unknown | down | 1.53 | 1.30E-03 | up | 1.04 | 8.02E-01 | No |
| *CACHD1* | BX537603 | ENSG00000158966 | e13 | Unknown | down | 1.53 | 4.31E-02 | down | 1.16 | 5.89E-01 | No |
| *TMC6* | BC023597 | ENSG00000141524 | e5 | alternative_last_exon | down | 1.52 | 2.80E-03 | up | 1.35 | 9.38E-03 | Yes |
| *CDC42BPB* | AF128625 | ENSG00000121388 // ENSG00000198752 | e20 | exon_skipping | down | 1.52 | 1.14E-03 | up | 1.37 | 3.02E-02 | Yes |
| *AURKA* | BC027464 | ENSG00000087586 | e10 | Unknown | down | 1.52 | 3.45E-02 | up | 1.68 | 6.12E-02 | Yes |
| *PCCB* | AB209009 | ENSG00000114054 | e5 | exon_skipping | down | 1.52 | 4.94E-02 | up | 1.45 | 6.16E-02 | Yes |
| *MED24* | AK291040 | ENSG00000008838 | e28 | exon_skipping,exon_skipping | down | 1.52 | 1.09E-02 | up | 1.34 | 6.16E-02 | Yes |
| *TTPA* | BC041784 | ENSG00000137561 | e1 | alternative_first_exon | down | 1.52 | 4.44E-02 | up | 1.76 | 6.80E-02 | No |
| *PHF20L1* | BC144654 | ENSG00000129292 | e16 | Unknown | down | 1.52 | 3.78E-02 | up | 1.45 | 7.47E-02 | Yes |
| *ENGASE* | AK025518 | ENSG00000167280 | e1 | alternative_first_exon | down | 1.52 | 3.28E-02 | up | 1.55 | 7.72E-02 | Yes |
| *MTIF2* | AF494407 | ENSG00000085760 | e13 | Unknown | down | 1.52 | 3.05E-02 | up | 1.49 | 7.88E-02 | Yes |
| *LPHN1* | AB020628 | ENSG00000072071 | e2 | Unknown | down | 1.52 | 4.32E-03 | up | 1.35 | 8.39E-02 | No |
| *CDK7* | X77743 | ENSG00000134058 | e8 | Unknown | down | 1.52 | 4.07E-02 | up | 1.54 | 8.69E-02 | Yes |
| *PIAS2* | AK308143 | ENSG00000078043 | e7 | Unknown | down | 1.52 | 9.77E-04 | up | 1.34 | 9.00E-02 | Yes |
| *PPP6R2* | AB014585 | ENSG00000100239 | e15 | exon_skipping | down | 1.52 | 1.11E-02 | up | 1.27 | 1.03E-01 | No |
| *FIG4* | BC041338 | ENSG00000112367 | e1 | alternative_first_exon | down | 1.52 | 2.58E-02 | up | 1.44 | 1.18E-01 | Yes |
| *OSCP1* | BC018069 | ENSG00000116885 | e9 | Unknown | down | 1.52 | 6.94E-03 | up | 1.27 | 1.35E-01 | Yes |
| *NCAPG2* | BC043404 | ENSG00000146918 | e28 | exon_skipping | down | 1.52 | 4.30E-03 | up | 1.47 | 1.53E-01 | Yes |
| *ZNF484* | AK309617 | ENSG00000127081 | e2 | exon_skipping,exon_skipping | down | 1.52 | 8.28E-03 | up | 1.28 | 1.64E-01 | No |
| *CDK18* | BC011526 | ENSG00000117266 | e3 | exon_skipping,alternative_first_exon | down | 1.52 | 8.48E-03 | up | 1.23 | 2.34E-01 | No |
| *ALS2* | AF391100 | ENSG00000003393 | e31 | Unknown | down | 1.52 | 3.72E-02 | up | 1.27 | 2.44E-01 | No |
| *HELQ* | AF436845 | ENSG00000163312 | e4 | exon_skipping | down | 1.52 | 4.81E-02 | up | 1.17 | 3.89E-01 | No |
| *RRN3P3* | AK055742 | ENSG00000232297 | e6 | exon_skipping | down | 1.52 | 2.02E-02 | up | 1.06 | 6.42E-01 | Yes |
| *ZNF586 // ZNF587 // ZNF776* | AK055448 | ENSG00000083828 // ENSG00000152443 // ENSG00000198466 | e7 | exon_skipping | down | 1.52 | 2.92E-02 | up | 1.08 | 6.54E-01 | Yes |
| *NFKB1* | BC051765 | ENSG00000109320 | e5 | Unknown | down | 1.52 | 2.73E-02 | up | 1.06 | 7.25E-01 | No |
| *FRMD4B* | AK091076 | ENSG00000114541 | e23 | exon_skipping | down | 1.52 | 1.37E-02 | up | 1.07 | 7.68E-01 | No |
| *KDM5C* | EF613277 | ENSG00000126012 | e28 | Unknown | down | 1.52 | 2.14E-02 | up | 1.03 | 8.51E-01 | No |
| *FIP1L1 // PDGFRA* | AY229892 | ENSG00000134853 // ENSG00000145216 | e26 | exon_skipping | down | 1.52 | 9.84E-04 | up | 1.03 | 8.73E-01 | No |
| *GRAMD3* | AK316288 | ENSG00000155324 | ae2 | alternative_first_exon | down | 1.52 | 6.34E-03 | up | 1.03 | 8.86E-01 | No |
| *TPM1* | AX747334 | ENSG00000140416 | e2 | exon_skipping | down | 1.52 | 4.33E-02 | down | 1.37 | 7.16E-02 | No |
| *AKT3* | AY005799 | ENSG00000117020 | e12 | Unknown | down | 1.52 | 2.39E-02 | down | 1.88 | 8.00E-02 | No |
| *SIAE* | AF300796 | ENSG00000110013 | e13 | alternative_last_exon | down | 1.52 | 1.40E-04 | down | 1.42 | 1.51E-01 | No |
| *ADAMTS2* | BC111459 | ENSG00000087116 | e9 | Unknown | down | 1.52 | 1.33E-02 | down | 1.53 | 1.61E-01 | No |
| *FLI1* | AK294279 | ENSG00000151702 | e2 | exon_skipping,alternative_first_exon | down | 1.52 | 3.18E-03 | down | 1.55 | 2.76E-01 | No |
| *CELF2* | AF090693 | ENSG00000048740 | ae16 | intron_retention | down | 1.51 | 3.26E-02 | up | 1.16 | 6.02E-03 | No |
| *AURKAIP1* | BC062333 | ENSG00000175756 | ae1 | alternative_donor_splice_site | down | 1.51 | 2.72E-02 | up | 1.42 | 2.09E-02 | No |
| *ISY1 // RAB43* | AB032986 | ENSG00000172780 // ENSG00000240682 | e15 | exon_skipping | down | 1.51 | 4.57E-02 | up | 1.25 | 4.44E-02 | Yes |
| *FGGY* | BC014947 | ENSG00000172456 | e17 | Unknown | down | 1.51 | 2.68E-03 | up | 2.26 | 5.03E-02 | No |
| *RINT1* | BC068483 | ENSG00000135249 | e5 | exon_skipping | down | 1.51 | 8.16E-03 | up | 1.53 | 5.06E-02 | Yes |
| *PPP2R1B* | AK301705 | ENSG00000137713 | e19 | alternative_last_exon | down | 1.51 | 3.06E-02 | up | 1.33 | 5.25E-02 | No |
| *PMS2* | BX537558 | ENSG00000122512 | e3 | Unknown | down | 1.51 | 1.07E-02 | up | 1.54 | 5.51E-02 | Yes |
| *ZNF202* | AK226047 | ENSG00000166261 | e1 | alternative_first_exon | down | 1.51 | 1.06E-02 | up | 1.33 | 6.54E-02 | Yes |
| *SEC16B* | AB067515 | ENSG00000120341 | e8 | exon_skipping | down | 1.51 | 2.40E-02 | up | 1.78 | 7.69E-02 | No |
| *MCM3AP* | AJ010089 | ENSG00000160294 | e19 | Unknown | down | 1.51 | 3.56E-02 | up | 1.20 | 9.85E-02 | Yes |
| *MLKL* | AX747141 | ENSG00000168404 | e4 | exon_skipping | down | 1.51 | 2.72E-02 | up | 1.39 | 1.04E-01 | Yes |
| *C10orf81* | AK126354 | ENSG00000148735 | ae16 | intron_retention | down | 1.51 | 2.18E-02 | up | 1.76 | 1.09E-01 | Yes |
| *CENPL* | BC033154 | ENSG00000120334 | e5 | exon_skipping | down | 1.51 | 4.65E-02 | up | 1.24 | 1.81E-01 | Yes |
| *EPRS* | CR933648 | ENSG00000136628 | e33 | alternative_last_exon | down | 1.51 | 1.63E-02 | up | 1.37 | 2.08E-01 | Yes |
| *---* | AK302871 | --- | e18 | Unknown | down | 1.51 | 4.65E-02 | up | 1.15 | 5.39E-01 | No |
| *UTY* | AF000994 | ENSG00000183878 | e11 | Unknown | down | 1.51 | 4.17E-02 | up | 1.60 | 5.46E-01 | Yes |
| *---* | AK096680 | --- | e1 | alternative_first_exon | down | 1.51 | 8.40E-03 | up | 1.08 | 6.44E-01 | No |
| *C8orf76 // ZHX1* | BC012379 | ENSG00000165156 // ENSG00000189376 | e5 | exon_skipping,alternative_last_exon | down | 1.51 | 1.10E-02 | up | 1.15 | 7.45E-01 | No |
| *TRRAP* | AF076974 | ENSG00000196367 | e75 | Unknown | down | 1.51 | 3.98E-02 | up | 1.07 | 7.61E-01 | No |
| *SNRNP35* | BC020829 | ENSG00000184209 | e4 | exon_skipping | down | 1.51 | 3.10E-02 | up | 1.01 | 9.52E-01 | Yes |
| *PDZD3* | AB094096 | ENSG00000172367 | e3 | Unknown | down | 1.51 | 3.42E-02 | down | 1.64 | 6.22E-02 | No |
| *GIMAP5* | AK002158 | ENSG00000196329 | e2 | exon_skipping | down | 1.51 | 2.51E-02 | down | 1.75 | 6.99E-02 | Yes |
| *FLNC* | AB371585 | ENSG00000128591 | e42 | Unknown | down | 1.51 | 1.35E-02 | down | 1.37 | 1.24E-01 | No |
| *ABLIM3* | AB020650 | ENSG00000173210 | ae24 | intron_retention | down | 1.51 | 3.78E-02 | down | 1.37 | 2.29E-01 | No |
| *NEDD4L* | AB071179 | ENSG00000049759 | e6 | exon_skipping | down | 1.51 | 3.91E-02 | down | 1.30 | 2.57E-01 | No |
| *LGALS9B* | AK290263 | ENSG00000170298 | e3 | Unknown | down | 1.51 | 3.19E-02 | down | 1.19 | 4.32E-01 | Yes |
| *SECISBP2L* | D87445 | ENSG00000138593 | e15 | Unknown | down | 1.51 | 2.48E-02 | down | 1.11 | 5.87E-01 | No |
| *GPC3* | U50410 | ENSG00000147257 | e2 | exon_skipping | down | 1.51 | 2.58E-02 | down | 1.01 | 9.82E-01 | No |
| *SAE1* | AK055481 | ENSG00000142230 | e1 | alternative_first_exon | down | 1.50 | 1.53E-02 | up | 1.48 | 1.49E-02 | Yes |
| *ZBTB40* | AB007947 | ENSG00000184677 | e9 | Unknown | down | 1.50 | 2.54E-03 | up | 1.46 | 1.71E-02 | Yes |
| *PMPCA* | BC111399 | ENSG00000165688 | e1 | alternative_first_exon | down | 1.50 | 4.39E-02 | up | 1.44 | 2.03E-02 | Yes |
| *HTT* | AB016794 | ENSG00000197386 | e52 | Unknown | down | 1.50 | 4.72E-03 | up | 1.28 | 4.14E-02 | No |
| *---* | BX649060 | --- | ae6 | intron_retention | down | 1.50 | 3.15E-06 | up | 1.26 | 4.62E-02 | No |
| *RGS12* | BC028154 | ENSG00000159788 | e13 | Unknown | down | 1.50 | 5.62E-03 | up | 1.24 | 8.76E-02 | No |
| *RUNX1* | D43969 | ENSG00000159216 | e2 | alternative_first_exon | down | 1.50 | 4.61E-02 | up | 1.45 | 1.19E-01 | Yes |
| *ABLIM2* | AK094754 | ENSG00000163995 | e22 | exon_skipping,alternative_first_exon | down | 1.50 | 1.39E-02 | up | 1.50 | 1.59E-01 | No |
| *DAGLB // KDELR2* | AK127440 | ENSG00000136240 // ENSG00000164535 | e18 | exon_skipping | down | 1.50 | 3.09E-02 | up | 1.05 | 7.37E-01 | Yes |
| *C8orf76 // ZHX1* | BC012379 | ENSG00000165156 // ENSG00000189376 | e4 | exon_skipping | down | 1.50 | 1.26E-02 | up | 1.15 | 7.45E-01 | No |
| *KIAA1826* | AB058729 | ENSG00000170903 | ae1 | intron_retention | down | 1.50 | 4.52E-02 | up | 1.04 | 8.28E-01 | No |
| *ITGAM* | BC099660 | ENSG00000169896 | e18 | Unknown | down | 1.50 | 6.38E-03 | down | 1.53 | 1.90E-01 | No |
