# Supplementary material for: A Gene Expression and Pre-mRNA Splicing Signature That Marks the Adenoma-Adenocarcinoma Progression in Colorectal Cancer
Source: PLoS One. 2014 Feb 6;9(2):e87761. doi: 10.1371/journal.pone.0087761 (PMC3916340; doi:10.1371/journal.pone.0087761)
Supplement: Table S14 — List of the deregulated exons in colorectal adenomas in comparison with normal mucosae, for the genes from the Agilent™ gene expression signature of 44 probes. (DOC) [file pone.0087761.s020.doc]

**Table S14. List of the deregulated exons in colorectal adenomas in comparison with normal mucosae, for the genes from the Agilent^TM^ gene expression signature of 44 probes.** The results of Human Exon 1.0 ST (Affymetrix^TM^) for the deregulated exons in CRA *vs*. NOR (≥ 1.5 FC, P-value ≤ 0.05 by *t*-test), for the genes of the Agilent^TM^ gene expression signature of 44 probes, are presented.

| Gene Symbol | Detected Exon | Known  Alternative Event | Regulation  Splicing Index | Fold-Change  Splicing Index | P-Value  Splicing Index | Regulation  Gene | Fold-Change  Gene | P-value  Gene |
| --- | --- | --- | --- | --- | --- | --- | --- | --- |
| *NR3C2* | e1 | alternative_first_exon | up | 1.56 | 4.36E-03 | down | 2.17 | 1.54E-03 |
| *CFH* | e17 | Unknown | up | 1.98 | 3.13E-02 | down | 3.70 | 7.96E-03 |
| *CFH* | e20 | Unknown | up | 1.57 | 4.26E-02 | down | 3.67 | 7.20E-03 |
| *DPT* | e2 | Unknown | down | 1.55 | 2.10E-02 | down | 5.73 | 1.88E-11 |
| *DPT* | e3 | Unknown | down | 2.42 | 5.65E-04 | down | 5.73 | 1.88E-11 |
| *DPT* | e1 | alternative_first_exon | up | 1.60 | 1.70E-12 | down | 5.73 | 1.88E-11 |
| *CRYAB* | ae1 | alternative_first_exon,intron_retention | up | 2.50 | 5.28E-03 | down | 4.07 | 1.40E-03 |
| *FBLN1* | e11 | Unknown | down | 1.85 | 1.37E-02 | down | 4.44 | 1.88E-03 |
| *FBLN1* | e20 | Unknown | down | 1.89 | 9.76E-04 | down | 4.44 | 1.88E-03 |
| *FBLN1* | e16 | exon_skipping,alternative_last_exon | up | 3.12 | 2.47E-02 | down | 4.44 | 1.88E-03 |
| *FBLN1* | e7 | Unknown | up | 1.60 | 2.29E-02 | down | 4.44 | 1.88E-03 |
| *FBLN1* | e13 | Unknown | down | 1.58 | 2.38E-02 | down | 4.49 | 1.62E-03 |
| *FBLN1* | e3 | exon_skipping | up | 4.19 | 3.80E-03 | down | 4.49 | 1.62E-03 |
| *ITIH5* | ae13 | alternative_last_exon | up | 3.19 | 3.87E-02 | down | 3.66 | 9.80E-03 |
| *ITIH5* | e14 | exon_skipping,alternative_last_exon | up | 3.07 | 2.84E-02 | down | 3.66 | 9.80E-03 |
| *ITIH5* | e18 | alternative_last_exon | up | 2.95 | 3.36E-02 | down | 3.66 | 9.80E-03 |
| *ITIH5* | e5 | Unknown | up | 1.66 | 1.69E-02 | down | 3.66 | 9.80E-03 |
| *SLIT3* | e12 | Unknown | down | 2.04 | 3.14E-03 | down | 3.58 | 3.83E-05 |
| *SLIT3* | e15 | Unknown | down | 1.67 | 2.98E-03 | down | 3.58 | 3.83E-05 |
| *SLIT3* | e17 | Unknown | down | 1.55 | 9.18E-03 | down | 3.58 | 3.83E-05 |
| *SLIT3* | e5 | Unknown | down | 1.62 | 5.30E-03 | down | 3.58 | 3.83E-05 |
| *SLIT3* | e8 | Unknown | down | 1.50 | 3.73E-02 | down | 3.58 | 3.83E-05 |
| *SLIT3* | e13 | alternative_last_exon | up | 1.68 | 4.38E-03 | down | 3.58 | 3.83E-05 |
| *SLIT3* | e18 | Unknown | up | 1.54 | 4.50E-03 | down | 3.58 | 3.83E-05 |
| *SLIT3* | e20 | Unknown | up | 1.51 | 4.23E-02 | down | 3.58 | 3.83E-05 |
| *SLIT3* | e24 | Unknown | up | 1.96 | 6.44E-03 | down | 3.58 | 3.83E-05 |
| *SLIT3* | e31 | Unknown | up | 1.96 | 9.01E-04 | down | 3.58 | 3.83E-05 |
| *SLIT3* | e32 | Unknown | up | 1.75 | 1.18E-03 | down | 3.58 | 3.83E-05 |
| *SLIT3* | e34 | Unknown | up | 1.76 | 2.88E-03 | down | 3.58 | 3.83E-05 |
| *TIMP1* | ae3 | intron_retention | down | 1.63 | 4.55E-02 | up | 1.36 | 2.57E-01 |
