# Supplementary material for: A Gene Expression and Pre-mRNA Splicing Signature That Marks the Adenoma-Adenocarcinoma Progression in Colorectal Cancer
Source: PLoS One. 2014 Feb 6;9(2):e87761. doi: 10.1371/journal.pone.0087761 (PMC3916340; doi:10.1371/journal.pone.0087761)
Supplement: File S1 — Supplementary Methods. MSI, mutation and protein analysis methods. (DOC) [file pone.0087761.s021.doc]

**SUPPLEMENTARY METHODS**

**SNaPshot Multiplex Assays – *KRAS* and *BRAF* Missense Mutations Screening**

*KRAS* and *BRAF* genes were analyzed in 56 samples derived from colorectal biopsies (14 NOR, 30 CRAs and 12 CRCs), for the presence of missense mutations using the ABI PRISM SNaPShot Multiplex kit (Applied Biosystems, Courtabœuf, France). Briefly, two multiplex PCR were designed, the first for *KRAS* exons 2 and 3 and *BRAF* exon 15 and the second for *KRAS* exons 3 and 4. The multiplex PCR were performed using the Qiagen^TM^ Multiplex PCR kit in a total volume of 20 µL containing 400 ng genomic DNA. The PCR products were treated with Exonuclease I (ExoI) and Shrimp Alkaline Phosphatase (SAP) (USB, Cleveland, Ohio USA). Each extension primer (SNaPshot primer) was designed to anneal to the reverse strand of its targeted PCR product adjacent to the mutation site of interest. SNaPshot primers contained an additional tail at their 5’ end allowing their simultaneous detection. The mutation detection reactions were performed in a total volume of 5 µL, containing 1.5 µL SAP/ExoI treated PCR product, 2 µL SNaPshot Multiplex Ready Reaction mix, and 1.5 µL of a SNaPShot primers mix (each primer at a final concentration from 0.5 to 1.5 µM). The products were treated with SAP before being analyzed on an automatic sequencer (ABI PRISM 3500 Dx Genetic Analyzer, Applied Biosystems). Data were analyzed using GeneMapper Analysis Software version 4.0 (Applied Biosystems).

**Multiplex Sizing Assay – *EGFR* and *HER2* Indels Screening**

A fragment length analysis method was used to screen for deletions and insertions in *EGFR* exons 19 and 20 and in *HER2* exon 20. Genomic tumor DNA of 27 samples derived from colorectal biopsies (nine NOR, 11 CRAs and seven CRCs) was amplified using the Qiagen^TM^ Multiplex PCR kit and the following primers: 5′-N-CTG-GAT-CCC-AGA-AGG-TGA-GA-3′ and 5′-GAT-TTC-CTT-GTT-GGC-TTT-CG-3′ (*EGFR* exon 19), 5′-N-CTC-CAG-GAA-GCC-TAC-GTG-AT-3′ and 5′-CTG-CGT-GAT-GAG-CTG-CAC-3′ (*EGFR* exon 20), 5′-N-CCT-CTC-AGC-GTA-CCC-TTG-TC-3′ and 5′-AGG-GCA-TAA-GCT-GTG-TCA-CC-3′ (*HER2* exon 20). For universal labeling, the forward primers were tailed with a short nucleotide sequence (N) that matches to a universal FAM-labeled probe. The labeled PCR products were subjected to capillary electrophoresis on an ABI PRISM 3100 XL Genetic Analyzer (Applied Biosystems) and compared with the wild-type PCR product to determine whether differences in length were present and whether the differences represented a deletion or insertion. Positive samples were re-amplified and sequenced using the BigDye Terminator v3.1 cycle sequencing kit (Applied Biosystems), according to the manufacturer’s protocol. Sequence electrophoregrams were interpreted using SeqPatient analysis Sofware version 3.5.2 (JSI medial system).

**MSI status determination**

Instability of microsatellites NR21, BAT 25, BAT26, NR24 and MONO 27 was analyzed in 12 CRA samples (4 from each morphological subgroup, A1, A2 and A3) with the MSI analysis system, version 1.2 (Promega, Madison, USA). Amplification was performed with the Gold Taq DNA polymerase (Applied Biosystems) and analyzed with a genetic analyzer 3130 (Applied Biosystems). A sample was considered MSI if at least 3/5 microsatellites were instable, according to the manufacturer.

**Protein Extraction and Western Blotting Analysis**

Only a few colorectal biopsy samples were available to the protein extraction: four NOR, seven CRAs and four CRCs. Tissue samples (20 mg) were lysed in boiling buffer [10 mM Tris-HCl pH 7.4, 1% SDS, 1 mM Na_3_VO_4_] containing protease inhibitor cocktail (Roche). Protein concentration was determined using the Bio-Rad DC protein assay. Fifty micrograms of proteins were boiled in Laemmli sample buffer (Bio-Rad), separated by SDS-PAGE using 12% polyacrylamide gels and blotted onto hydrophobic polyvinyl difluoride membranes for fluorescent western blotting (Hybond-LFP membranes, GE Healthcare). Non-specific binding sites were blocked for one hour at room temperature by SEA-BLOCK blocking buffer (Thermo Scientific) before incubation for three hours at room temperature or overnight incubation at 4°C with specific rabbit or mouse primary antibodies: Dermatopontin (ab112600, Abcam), HSD11B2 (ab89669), RDH5 (ab101457), SMPDL3A (ab68533), TRIB3 (ab50516) or Hsc70 as a loading control. Antibodies were used at the following dilutions: 1:50 (Dermatopontin), 1:500 (HSD11B2, RDH5 and SMPDL3A) and 1:2 000 (Hsc70). Primary antibodies were detected with secondary antibodies labeling with IR-Dye (IR-Dye 680 donkey anti-mouse or IR-Dye 800 goat anti-rabbit). Blots were revealed using the Odyssey^TM^ system.
