# Supplementary material for: A Gene Expression and Pre-mRNA Splicing Signature That Marks the Adenoma-Adenocarcinoma Progression in Colorectal Cancer
Source: PLoS One. 2014 Feb 6;9(2):e87761. doi: 10.1371/journal.pone.0087761 (PMC3916340; doi:10.1371/journal.pone.0087761)
Supplement: File S2 — Supplementary Results. MSI status and mutation analyses. (DOC) [file pone.0087761.s022.doc]

**SUPPLEMENTARY RESULTS**

**MSI status analysis**

MSI was analyzed in 12 CRA samples (4 from each morphological subgroup, A1, A2 and A3), but none was positive.

**Mutation Analyses**

Alterations of *KRAS* can occur relatively early in CRC formation, but do not appear necessary for the malignant conversion of CRA to CRC [54]. These mutations typically affect codons 12 and 13 in exon 2, and to a lesser extent codon 61 in exon 3, leading to a constitutively active protein. We showed that *KRAS* mutations occurred in 50% (15 out of 30) CRAs; the G12D mutation was the most common (20%), whereas the G12A, G12R, G12V, G13D, and A146T were the less frequent (3%). *KRAS* mutations occurred in 25% (three out of 12) CRCs and in none of the 14 normal mucosa (NOR) samples. Hence, *KRAS* mutations were somewhat enriched in CRA as compared to CRC (50% in CRA *vs*. 25% in CRC), as previously reported [55,56]. *BRAF* mutations (V600E) occurred in 17% (2 out of 12) CRC samples, and in none of the CRA and NOR samples. *EGFR* and *HER2* did not show any mutations in the nine NOR, 11 CRA and seven CRC samples tested. *PI3K* mutations in codons 542, 545, 546 and 1047 were also analyzed in 12 CRAs (data not shown), and only 17% (2 out of 12) CRAs showed a *PI3K* mutation (E545Q and E545K). The mutation analysis data are presented in Supplementary Table 1.

**Table S15.** ***KRAS* and *BRAF* mutation analyses in colorectal biopsy samples.** The results of SNaPshot Multiplex assay are presented.

Abbreviations: NOR: colorectal normal mucosa; CRA: colorectal adenoma; CRC: colorectal cancer.

|  | | | *KRAS* Exon 2 | | *KRAS* Exon 3 | *KRAS* Exon 4 |  |
| --- | --- | --- | --- | --- | --- | --- | --- |
| Sample | Group | Subgroup | Codon 12 | Codon 13 | Codon 61 | Codon 146 | *BRAF* (V600E) |
| 81 | CRA | Out-of-Class | NEG | NEG | NEG | NEG | NEG |
| 58 | CRA | A1 | G12V | NEG | NEG | NEG | NEG |
| 60 | CRA | A1 | G12D | NEG | NEG | NEG | NEG |
| 74 | CRA | A1 | NEG | NEG | NEG | NEG | NEG |
| 79 | CRA | A1 | NEG | NEG | NEG | NEG | NEG |
| 94 | CRA | A1 | G12A | NEG | NEG | NEG | NEG |
| 162 | CRA | A1 | NEG | NEG | Q61H | NEG | NEG |
| 78 | CRA | A2 | NEG | NEG | NEG | A146T | NEG |
| 82 | CRA | A2 | NEG | NEG | NEG | NEG | NEG |
| 84A | CRA | A2 | NEG | NEG | NEG | NEG | NEG |
| 84B | CRA | A2 | NEG | NEG | NEG | NEG | NEG |
| 88 | CRA | A2 | NEG | NEG | NEG | NEG | NEG |
| 146 | CRA | A2 | G12D | NEG | NEG | NEG | NEG |
| 157A | NOR | - | NEG | NEG | NEG | NEG | NEG |
| 157C | CRA | A2 | NEG | G13D | NEG | NEG | NEG |
| 157B | CRC | - | G12D | NEG | NEG | NEG | NEG |
| 71 | CRA | A3 | NEG | NEG | NEG | NEG | NEG |
| 73 | CRA | A3 | G12S | NEG | NEG | NEG | NEG |
| 76 | CRA | A3 | NEG | NEG | NEG | NEG | NEG |
| 83 | CRA | A3 | G12S | NEG | NEG | NEG | NEG |
| 86A | CRA | A3 | NEG | NEG | NEG | NEG | NEG |
| 91 | CRA | A3 | NEG | NEG | Q61H | NEG | NEG |
| 95 | CRA | A3 | NEG | NEG | NEG | NEG | NEG |
| 139 | CRA | A3 | G12R | NEG | NEG | NEG | NEG |
| 141 | CRA | A3 | NEG | NEG | NEG | NEG | NEG |
| 144 | CRA | A3 | NEG | NEG | NEG | NEG | NEG |
| 153 | CRA | A3 | G12D | NEG | NEG | NEG | NEG |
| 163 | CRA | A3 | G12D | NEG | NEG | NEG | NEG |
| 129B | CRA | A3 | NEG | NEG | NEG | NEG | NEG |
| 140A | NOR | - | NEG | NEG | NEG | NEG | NEG |
| 140B | CRA | A3 | G12D | NEG | NEG | NEG | NEG |
| 155A | NOR | - | NEG | NEG | NEG | NEG | NEG |
| 155B | CRA | A3 | G12D | NEG | NEG | NEG | NEG |
| 165A | NOR | - | NEG | NEG | NEG | NEG | NEG |
| 165C | CRA | A3 | NEG | NEG | NEG | NEG | NEG |
| 165B | CRC | - | NEG | NEG | NEG | NEG | NEG |
| 132A | NOR | - | NEG | NEG | NEG | NEG | NEG |
| 132B | CRC | - | NEG | NEG | NEG | NEG | NEG |
| 133A | NOR | - | NEG | NEG | NEG | NEG | NEG |
| 133B | CRC | - | NEG | NEG | NEG | NEG | NEG |
| 134A | NOR | - | NEG | NEG | NEG | NEG | NEG |
| 134B | CRC | - | NEG | NEG | NEG | NEG | POS |
| 135A | NOR | - | NEG | NEG | NEG | NEG | NEG |
| 135B | CRC | - | NEG | NEG | NEG | NEG | NEG |
| 136A | NOR | - | NEG | NEG | NEG | NEG | NEG |
| 136B | CRC | - | NEG | NEG | NEG | NEG | NEG |
| 142A | NOR | - | NEG | NEG | NEG | NEG | NEG |
| 142B | CRC | - | NEG | NEG | NEG | NEG | POS |
| 147A | NOR | - | NEG | NEG | NEG | NEG | NEG |
| 147B | CRC | - | G12C | NEG | NEG | NEG | NEG |
| 150A | NOR | - | NEG | NEG | NEG | NEG | NEG |
| 150B | CRC | - | NEG | NEG | NEG | NEG | NEG |
| 156A | NOR | - | NEG | NEG | NEG | NEG | NEG |
| 156B | CRC | - | G12V | NEG | NEG | NEG | NEG |
| 164A | NOR | - | NEG | NEG | NEG | NEG | NEG |
| 164B | CRC | - | NEG | NEG | NEG | NEG | NEG |

**References**

54. Grady WM, Markowitz SD (2002) Genetic and epigenetic alterations in colon cancer. Annual Review of Genomics and Human Genetics 3: 101-128.

55. Yadamsuren E-A, Nagy S, Pajor L, Lacza A, Bogner B (2012) Characteristics of advanced- and non advanced sporadic polypoid colorectal adenomas: correlation to KRAS mutations. Pathology & Oncology Research 18: 1077-1084.

56. Vogelstein B, Fearon ER, Hamilton SR, Kern SE, Preisinger AC, et al. (1988) Genetic alterations during colorectal-tumor development. New England Journal of Medicine 319: 525-532.
